# Chronic Alcohol Consumption Reprograms Hepatic Metabolism Through Organelle-Specific Acetylation in Mice

**DOI:** 10.1016/j.mcpro.2025.100990

**Published:** 2025-05-12

**Authors:** Mirjavid Aghayev, Megan R. McMullen, Serguei Ilchenko, Andrea Arias-Alvarado, Victor Lufi, Jack Mathis, Hannah Marchuk, Tsung-Heng Tsai, Guo-Fang Zhang, Laura E. Nagy, Takhar Kasumov

**Affiliations:** 1 Department of Pharmaceutical Sciences, College of Pharmacy, Northeast Ohio Medical University, Rootstown, Ohio, USA; 2 Departments of Inflammation and Immunity and Gastroenterology/Hepatology, Northern Ohio Alcohol Center, Cleveland Clinic, Cleveland, Ohio, USA; 3 Division of Endocrinology, Metabolism and Nutrition, Duke Molecular Physiology Institute, Department of Medicine, Duke University, Durham North Carolina, USA; 4 Department of Mathematical Sciences, Kent State University, Kent, Ohio, USA

**Keywords:** acetylation, alcohol, acetylome dynamics, heavy water, high-resolution liquid chromatography-mass spectrometry, liver injury, mitochondria, metabolic labeling, proteopathy, turnover

## Abstract

Posttranslational acetylation of proteins by acetyl-CoA is a crucial regulator of proteostasis and substrate metabolism. Ethanol metabolism in the liver induces protein accumulation, acetylation, and metabolic disruption. Although acetylation impacts enzyme activity and stability, its role in ethanol-related protein accumulation and metabolic dysfunction remains unclear. Using stable isotope-based proteomics, acetylomics, and metabolic profiling in a mouse model of chronic ethanol-induced liver injury, we demonstrate that ethanol induces hepatic steatosis, inflammation, oxidative stress, and proteinopathy linked to altered protein turnover. Ethanol increased the cytosolic protein turnover related to oxidative stress and detoxification, while reducing turnover of mitochondrial metabolic enzymes. It also elevated the acetylation of mitochondrial enzymes and nuclear histones with minimal cytosolic changes, impairing mitochondrial protein degradation. These changes were associated with altered levels of acyl-CoAs and acyl-carnitines, amino acids, and tricarboxylic acid cycle intermediates, reflecting impaired fatty acid oxidation, nitrogen disposal and tricarboxylic acid cycle activities. These results suggest that ethanol-induced acetylation contributes to liver injury and that targeting acetylation may offer treatment for alcohol-induced liver diseases.

## Highlights

• The impact of ethanol on mouse liver acetylome dynamics was assessed using ²H₂O.
• Ethanol increased cytosolic protein turnover and decreased mitochondrial turnover.
• Ethanol enhanced acetylation in the nucleus and mitochondria but not in the cytosol.
• Acetylation impaired mitophagy, leading to mitochondrial protein accumulation.
• These changes were associated with disrupted mitochondrial metabolism.

## Graphical Abstract

**Figure:**
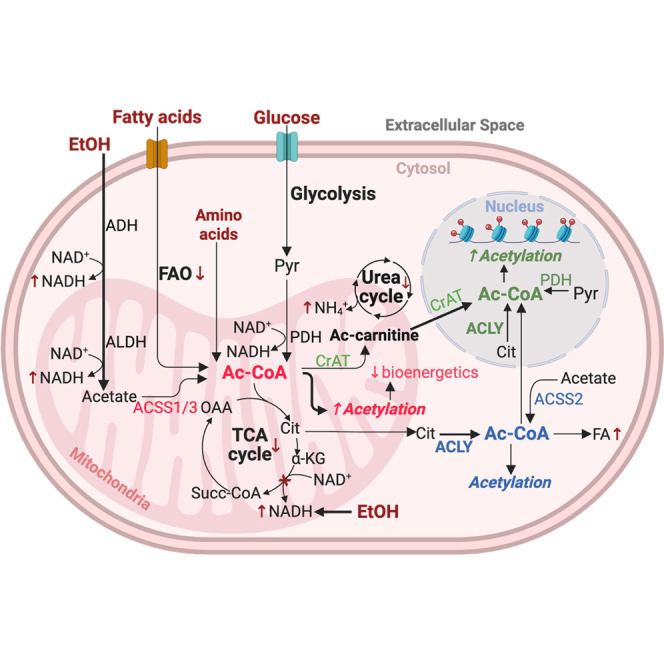

## In Brief

Alcohol contributes to liver disease, neurological disorders, and cancer. This study uses high-throughput proteomics and acetylomics with ²H₂O labeling in mice to assess chronic alcohol's effects on hepatic injury. Alcohol reprograms liver proteostasis by altering subcellular acetylation and protein turnover—enhancing histone and cytosolic protein dynamics while reducing mitochondrial protein degradation. Alcohol-induced acetylation impairs autophagy and mitophagy, leading to mitochondrial protein accumulation and metabolic disruptions. These findings reveal mechanisms of alcohol-induced liver injury and identify acetylation as a potential therapeutic target.

Chronic alcohol consumption leads to severe health issues, including neurological disorders, various cancers, and alcohol-associated liver diseases (1). The liver is the primary site for alcohol metabolism and is susceptible to alcohol-induced injury, including liver enlargement (hepatomegaly) (2). Apart from fat accumulation, alcohol-induced hepatomegaly is linked to an expansion of hepatic proteins (3). Alcohol disrupts hepatic metabolic pathways, affecting the metabolism of proteins, carbohydrates, and fat (4). However, the exact mechanisms of alcohol-mediated protein accumulation and their role in metabolic rewiring remain incompletely understood.

Hepatic alcohol oxidation to acetaldehyde is primarily driven by cytosolic alcohol dehydrogenase, microsomal CYP2E1, and to a lesser extent, peroxisomal catalase. Mitochondrial acetaldehyde dehydrogenase 2 then converts acetaldehyde to acetate, which is further processed into acetyl-CoA by mitochondrial Acss1/3 and nuclear/cytosolic Acss2, members of acyl-CoA synthetase short-chain family (5). This alcohol metabolism alters the NADH/NAD^+^ ratio and acetyl-CoA levels, influencing substrate metabolism. Acetyl-CoA, essential for mitochondrial energy production and cytosolic lipid biosynthesis, is also the exclusive substrate for the reversible acetylation of lysine side chains in proteins (6). NADH/NAD^+^ ratio, behind regulating redox reactions, controls NAD^+^ -dependent sirtuins (Sirts)-catalyzed deacetylation reactions (7). Like phosphorylation, acetylation regulates metabolic pathways by modifying histone and non-histone proteins, affecting their transcription and activity (8). Acetylation also controls proteostasis, including protein synthesis, folding, trafficking, and degradation (9), crucial for cellular health and function. Yet, the impact of alcohol-induced acetylation in metabolism and proteostasis across cell compartments remains unclear.

Chronic alcohol consumption causes hyperacetylation of hepatic mitochondrial proteins involved in energy metabolism due to predominant production of acetyl-CoA in the mitochondria (10, 11). Alcohol also contributes to acetylation of cytosolic proteins and histones in the nuclear (2, 12, 13). Cytosolic acetyl-CoA, derived from mitochondria-exported citrate cleavage by ATP citrate lyase (Acly) (14) and free acetate activation via Acss2 (15), can freely enter the nucleus through pores in the nuclear membrane. The presence of pyruvate dehydrogenase, Acly, and Acss2 in the nucleus suggests their role in generating a local acetyl-CoA pool specifically for histone acetylation (16, 17). Recent studies demonstrated that carnitine acetyl transferase (CrAT) also transports mitochondrial acetyl-CoA to nuclear-cytosolic compartments, influencing histone acetylation (18, 19). Additionally, acetyl-CoA produced from peroxisomal fatty acid oxidation can contribute to the acetylation of cytosolic proteins and histones (18, 20). The influence of alcohol on acetylation across cellular compartments remains ambiguous and may vary with acute or chronic exposure. While acute alcohol intake does not impact subcellular acetylation, chronic alcohol administration leads to cytosolic hypoacetylation and mitochondrial hyperacetylation in mouse liver (21). Conversely, histone H3 is hyperacetylated in isolated hepatocytes and in rat liver following both acute and chronic alcohol intake (22, 23), suggesting alcohol-induced partitioning nuclear and cytosolic acetylation. The reasons behind this compartment-specific acetylation are unknown; however, understanding them is critical for elucidating the role of acetylation in metabolism and hepatic injury.

Current methods studying alcohol’s role on protein metabolism and acetylation rely on static measurements (24). Studies on alcohol’s effect on protein turnover have focused on total liver protein rather than individual proteins with distinct functions, producing inconsistent results, with reports of decreased, increased, or unchanged protein synthesis rates (25, 26, 27, 28). In addition, the influence of alcohol-induced acetylation (10, 29, 30) on hepatic protein turnover remains unknown. We as well as others have developed a heavy water (^2^H_2_O)–based metabolic labeling approach to assess global proteome dynamics (31, 32, 33, 34, 35). This method revealed how posttranslational acetylation affects hepatic mitochondrial proteins and histones in a diet-induced mouse model of metabolic dysfunction-associated steatotic liver disease (36, 37). Additionally, we found that alcohol-induced acetylation increases histone turnover in the developing rat brain (38). Here, we used this method to evaluate how chronic alcohol consumption affects liver proteostasis and metabolism in mice. We hypothesized that alcohol disrupts acetylation dynamics and enzyme stability, leading to impaired substrate oxidation and oxidative stress in the liver. Our results show that chronic alcohol exposure distinctly impacts acetyl-CoA metabolism in different cell compartments, increasing acetylation of histones and mitochondrial proteins, but not cytosolic proteins. This was linked to increased turnover of histones and cytosolic proteins but decreased turnover and accumulation of mitochondrial proteins. Alcohol-induced mitochondrial acetylation altered acyl-CoA and acyl-carnitine, and tricarboxylic acid (TCA) cycle intermediates, reflecting impaired fatty acid oxidation and TCA cycle activity. Elevated hepatic amino acids suggest urea cycle dysfunction, while stable glycine and cysteine levels are likely to reflect their increased use in glutathione synthesis to combat alcohol-induced oxidative stress.

## Experimental Procedures

Detailed procedures are described in the Supporting Information.

### Experimental Design and Statistical Rationale

In the chronic ethanol exposure experiment, a total of 18 mice (pair-fed (PF): n = 9, ethanol-fed (EF): n = 9) were used to evaluate the impact of alcohol on hepatic proteostasis. Based on the variability of ^2^H labeling and turnover rates of native proteins and acetylated sites estimated from our published data (36), we performed a power analysis, considering a typical scenario of using three peptides to estimate the turnover rate of a native protein and one peptide for the turnover rate of an acetylated site. For fold changes of 1.3 and 1.5 in the turnover rate of acetylated site, the sample size would be sufficient to provide a power of 82.4% and 99.4%, respectively, at a significance level of 0.05. In consideration of the same fold changes in native protein turnover rate, the power would be 99.2% and 99.9%. All liquid chromatography-tandem mass spectrometry (LC-MS/MS)–based proteomic analyses were conducted with two technical replicates for each sample.

### Animal Experiments

All animal experiments were approved by the Northeast Ohio Medical University and Cleveland Clinic Institutional Animal Care and Use Committee and performed in accordance with the National Institutes of Health guidelines on humane care of experimental animals (https://olaw.nih.gov/policies-laws/phs-policy.htm). Studies were conducted in wild type (WT) CB57BL/6 mice under our approved Institutional Animal Care and Use Committee protocol (#22–01–311). Given that females are more susceptible to alcohol-related liver injury compared to males (39), we examined the effects of chronic alcohol exposure on hepatic protein metabolism in female mice. In parallel, mechanistic studies focused on alcohol metabolism, not aimed at liver injury, were conducted using male mice.

#### Chronic Alcohol Exposure and ^2^H_2_O–Metabolic Labeling

Eight-week-old weight-matched female mice were randomized into control PF and EF groups (n = 9/group) and fed their respective liquid diets for 25 days. In the EF group, ethanol levels gradually increased from 1% to 6% v/v over the study period. In the PF group, maltose-dextrin replaced ethanol isocalorically. To assess proteome and acetylome dynamics, ^2^H_2_O was administered at specific intervals, ensuring steady-state body water labeling and consistent ethanol exposure. Mice were not fasted, and euthanasia occurred between 12 and 2 PM to avoid circadian variability in acetylation (21). After pentobarbital anesthesia, blood was collected via cardiac puncture, with serum and liver tissues preserved for future analysis.

#### Tracing Alcohol Metabolites and Acetylation With Ethanol-d6

To assess the impact of ethanol-derived acetate on liver protein acetylation and lipid biosynthesis, we administered ethanol-d6 (2.0 μl/g body weight) or saline intraperitoneally to nonfasted male mice. This ethanol dose doubles blood acetate (∼0.4 mM) within 30 min (13). Serum and freeze-clamped tissue samples were collected at baseline, 0.5, 1, 2, 4, and 6 h post injection under pentobarbital anesthesia and stored at −80 °C. We measured ^2^H-labeling of acetate in serum, acetyl-CoA, and acetyl-carnitine in liver tissue to trace ethanol-d6 metabolism. Liver samples were also evaluated for ^2^H-acetate incorporation into histones, palmitate, and cholesterol. Isotopically labeled metabolites and acetylated histones were quantified using quantitative LC-MS/MS and GC-MS.

### Analytical Procedures

#### Liver Enzymes, Lipids, and Lipid Peroxidation Products

Aspartate aminotransferase and alanine aminotransferase were measured using commercial kits (Diagnostic Chemicals, Ltd). Hepatic triglyceride levels were determined by using triglyceride glycerol 3-phosphate oxidase (GPO) reagent (Pointe Scientific Inc). Lipid peroxidation products, including malonyl dialdehyde, were quantified as markers of hepatic oxidative stress using a TBARS assay kit (Cayman Chemical Co).

#### Liver Histology

Formalin-fixed, paraffin-embedded liver tissue was sectioned and stained with H&E and Masson’s trichrome. Liver histology was evaluated blindly in deidentified liver slides. The alcohol-induced steatosis, lobular inflammation, and fibrosis were evaluated using the standard criteria as described (40).

#### Protease Activity Assay

Hepatic protease activity in the liver homogenate was measured using fluorometric assay kit (UBPBio) according to the manufacturer’s protocol. Fluorescence-labeled peptide substrates, succinyl-LLVY-7-amido-4-methylcoumatin (AMC), Boc-LRR-AMC, and Z-LLE-AMC were used for chymotrypsin-, trypsin- and caspase-like activity assays, respectively (41).

#### *Sirt3* Enzymatic Activity

Hepatic Sirt3 enzymatic activity was assayed using the manufacturer’s instructions with slight modifications (Cayman Chemical). Briefly, 40 μg of hepatic protein from whole tissue lysate was incubated at 37 °C for 45 min with specific substrates (42 μM QPKK(ε-acetyl)-AMC and 1 mM NAD^+^) followed by the addition of 5 μl of developer containing peptidase. Sirt3 activity was measured by reading fluorescence intensity every minute for 60 min using a fluorometric microplate reader at 350 nm/450 nm.

#### Immunoblot Analysis

Liver homogenates were prepared using radioimmunoprecipitation assay buffer for whole-cell protein lysates. Equal protein amounts (20–30 μg) were separated by SDS-PAGE on 4 to 20% gradient gels (Bio-Rad) at 100 V for 50 min under denaturing conditions, transferred to polyvinylidene difluoride membranes (Bio-Rad), and incubated overnight at 4 °C with primary antibodies. Afterward, membranes were incubated for 1 h at room temperature with corresponding HRP-linked anti-mouse IgG (7076P2) or anti-rabbit IgG (7074S) secondary antibodies from Cell Signaling Technology. Signal detection and quantification were performed using FluorChem imager and Alphaview software (ProteinSimple). The primary antibodies (including their sources and dilution factors) are listed in the Supplementary Key Resources Table.

### Metabolic Profiling by GC-MS and LC-MS/MS

#### TCA Cycle Intermediates and 2-Hydroxybutyrate

For the detailed protocol, refer to the online supplement. Briefly, ∼30 mg of tissue was spiked with mixed internal standards and extracted using the Folch method. The methoxylamine and TBDMS (1% tert-butylchlorodimethylsilane) derivatized samples were analyzed by GC-MS as described (42).

#### Amino Acids

Hepatic amino acids were analyzed as described (43). Briefly, 30 to 50 mg liver tissue samples were spiked with 50 nmol of [^13^C_6_]leucine and homogenized in 1 ml of 6% formic acid. Amino acids were isolated using an ion-exchange column (AG 50W-X8 resin, hydrogen form). The trimethylsilyl derivatives of amino acids were analyzed using GC-MS (Agilent Technologies) as described (43).

#### Palmitate and Cholesterol

To quantify hepatic lipogenesis, liver samples (∼30–40 mg), spiked with heptadecanoic acid (C17) solutions, homogenized in 0.5 ml of 1N NaCl and extracted by the Bligh-Dyer method (44). After derivatization with bis(trimethylsilyl) trifluoroacetamide + 1% trimethylchlorosilane, cholesterol and palmitate were analyzed using a GC-MS system as described (45).

#### Acetate

Ethanol-derived acetate in plasma was analyzed as described (46). Briefly, a 30-μl plasma sample was mixed with 30 μl of 200 μM [2,2,2-^2^H_3_-1,2-^13^C_2_]acetate (M + 5 acetate) internal standard. M0 acetate, M + 3 acetate, and M + 5 acetate were analyzed using a Sciex QTRAP 6500+ LC-MS/MS system connected with a Sciex AD UHPLC (Sciex).

#### Acylcarnitines

Acylcarnitines and free carnitine in liver tissue were analyzed as described (47). Briefly, powdered liver tissue samples (∼20 mg) were extracted and methylated with 3 M HCl methanol solution. The derivatized samples were analyzed by LC-QTRAP 6500^+^-MS/MS (Sciex).

#### Acyl-CoAs

Acyl-CoAs and free CoA were analyzed in liver tissue (∼75 mg) spiked with 0.2 nmol [^2^H_9_]pentanoyl-CoA (internal standard) after homogenization in 1.5 ml extraction buffer (5% acetic acid in 50:50 MeOH/H_2_O). The supernatant was processed on a 1 ml ion exchange cartridge and analyzed using a LC-QTRAP 6500^+^-MS/MS (Sciex) as described (48, 49).

### LC-MS/MS-Based Proteomics Analyses

#### Histone Sample Preparation

Histones were extracted and prepared for proteomic analysis according to Evertts *et al*. (50) with modifications. Initial chemical CD_3_-acetylation with acetic anhydride-d6, using 30 μg of acid-extracted histone proteins, was carried out to block nonmodified lysine sites. The acetylation process included two rounds to ensure near-complete acetylation, followed by hydrolysis of O-acetyl esters formed during chemical acetylation. Trypsin and gluC digestions were performed sequentially at pH 8.0, with subsequent CD_3_-acetylation of terminal amino acids. Samples were purified and analyzed by LC-MS/MS using Q Exactive mass spectrometer coupled to a Dionex HPLC setup (Thermo Fisher Scientific).

#### Total Proteome and Acetylome Sample Preparation

Briefly, hepatic tissue proteins (50 μg) underwent reduction with 4.5 mM DTT and alkylation with 10 mM iodoacetamide and in solution digestion (43). Tryptic peptides were desalted using C18 solid-phase extraction columns (Supelco DSC-18) and analyzed by nanospray LC-MS/MS.

Acetylated peptides from whole tissue lysates were prepared as described (51). In summary, acetylated peptides were immunoenriched from 2.5 mg of protein isolated from approximately 150 mg of liver tissue, utilizing the PTMScan kit for acetyl-lysine motifs [Ac-K] (#13416, Cell Signaling Technologies). This process followed the reduction and alkylation procedures previously described. Immuno-isolated acetylated peptides were analyzed by nanospray LC-MS/MS (36).

#### High-Resolution Mass Spectrometry Measurement of Proteins

Proteomics analysis employed an Ultimate 3000 UHPLC (Thermo Fisher Scientific) coupled online to a Q Exactive Plus Hybrid Quadrupole-Orbitrap Mass Spectrometer (Thermo Fisher Scientific,). Samples were desalted on an Acclaim PepMap 100 precolumn (300 μm × 5 mm, C18, 5 μm, 100 Å, Thermo Fisher Scientific) and separated on an Acclaim PepMap RSLC reverse-phase nanocolumn (75 μm × 15 cm, C18, 2 μm, 100 Å, Thermo Fisher Scientific) at 300 nl/min using mobile phases A (0.1% formic acid in water) and B (20% water in acetonitrile with 0.1% formic acid). The gradient progressed from 2% B to 40% over 100 min, ramped to 90% in 5 min, held at 90% B for 10 min, decreased to 2% in 2 min, and equilibrated for 13 min with 2% B.

The mass spectrometer was operated in data-dependent acquisition mode, conducting a full profile MS scan at 70,000 resolution (200 m/z) from 380 to 1300 m/z. MS/MS spectra were collected for the 19 most abundant product ions with an isolation window of 2.6 and offset of 0.3 m/z at 17,500 resolution (200 m/z). Higher-energy collisional dissociation used a normalized collision energy of 25 to 35%. Precursor ion masses were dynamically excluded from MS/MS analyses for 20 s. Ions with charge states of 1 and greater than 6 were excluded from MS/MS analyses. MS and MS/MS spectra were acquired for 100 ms with automatic gain control targets set at 1.0 × 10^6^ and 2.0 × 10^4^ ions, respectively.

#### Protein/Peptide Identification

Mascot software (Matrix Science, Version 2.5.1) searched all MS/MS spectra against the UniProt protein database released on June 29th, 2016 (149,730 entries) with an automatically generated decoy database of reversed sequences. A precursor mass tolerance of 10 ppm (MS1 full-scan spectra) and a product ion tolerance of 0.1 Da (MS/MS fragmentation spectra) were applied. This narrow mass window minimizes peptide identification errors and reduces the false discovery rate (FDR). Searches were conducted using carbamidomethyl as a fixed modification for cysteine, with optional modifications for methionine oxidation, lysine and N-terminal acetylation, and methylation, allowing for two missed cleavages. An ion scores greater than 35 was deemed significant. The interpretation process was further supported by additional searches using the BLAST program (http://blast.ncbi.nlm.nih.gov/Blast.cgi) when necessary. The majority of proteins were identified using at least two unique peptides, achieving 99% confidence with an FDR of 1%. The source data also include annotated MS/MS spectra for several proteins that were identified based on a single unique peptide, providing additional insights into their characterization.

Acetylated peptides were identified through exact masses of precursor molecular ions (MS1 spectra) and fragmentation patterns (MS/MS spectra), validated by manual inspection focusing on acetylation-specific MS2 fragments (+42.0106 Da). The annotated spectra for all identified acetylated peptides are included in the source data. We used UniProt (http://www.uniprot.org) to assign protein subcellular localization and identified mitochondrial proteins via the MitoCarta database.

### Data Analysis

#### Label-Free Quantification of Native and Acetylated Peptides

Label-free quantification (LFQ) of hepatic proteins and their acetylated forms was conducted on the same mice used for the kinetics studies as we previously described (36). Equal total protein amounts were used to address sample preparation variations. Alcohol-induced changes in expression of individual hepatic proteins were assessed with MaxQuant software (V2.6.7.0) with the integrated Andromeda search engine (52, 53). The following search parameters were utilized: a mass tolerance of 4.5 ppm for parent ions and 20 ppm for collision-induced fragment ions, allowing for two missed cleavages. Carbamidomethyl of cysteine was set as a fixed modification with optional modifications for methionine oxidation, lysine and N-terminal acetylation, and methylation. Acetylated peptides were filtered for a minimum Andromeda score of 40, adhering to the default setting for modified peptides. Proteins were identified using at least two unique peptides, with a 1% FDR from a reversed sequence database. The “match-between run” feature in MaxQuant performs retention time alignment across LC-MS/MS runs during LFQ. The MaxLFQ algorithm (52, 54) measures the area under the curve of high-intensity paired peptides' signals from multiple samples in each group. Peptide intensity is quantified as the summed extracted ion current (XIC), representing the total signal intensity from all isotopic clusters associated with the identified amino acid sequence. In labeling experiments, this measurement accounts for the combined signal from all isotopomers within the labeling cluster, ensuring accurate quantification of labeled peptides. Selected MaxQuant results were manually validated using Xcalibur software (version 3.1, Thermo Fisher Scientific).

Relative quantification of native and acetylated peptides between EF and PF groups was evaluated separately from the manually quantified data set, focusing on the significance of their fold changes (on a log_2_ scale), which indicates an EF-induced relative change.

#### Calculation of the Site-Specific Histone Acetylation Stoichiometry

Due to chemical deuteroacetylation, histone peptides with multiple acetylation sites became fully acetylated, leading to similar chromatographic properties that hindered quantification of positional isomers based on their molecular ions. Therefore, the relative abundances of endogenous acetylated positional isomers were calculated from their fragment y and b ions in the MS/MS scans.

For site-specific quantification of isobaric H3 (18, 19, 20, 21, 22, 23, 24, 25, 26) monoacetylated species, including H3K18ac and H3K23ac, the relative MS2 abundance of each isobaric species is multiplied by the corresponding MS1 abundance. For the site-specific quantification of single acetylated H4 (4, 5, 6, 7, 8, 9, 10, 11, 12, 13, 14, 15, 16, 17) forms (H4K5ac, H4K8ac, H4K12ac, and H4K16ac), the intensities of the y5, y7, and y12 ions from each specific acetylated precursor were quantified. Site-specific acetylation stoichiometry was quantified by matching light (endogenous) and heavy (chemically acetylated) ion pairs. The intensity of each positional isomer was divided by the total intensity of all quantified forms, following the methodology outlined by Feller *et al* (55), as detailed in the Supplementary materials.

The resolution of diacetylated species, such as H4K5acK12ac, H4K5acK16ac, H4K8acK12ac, and H4K8acK16ac, necessitates a three-step MS1-MS2-MS3 analysis (55), which was not conducted in this study. As a result, we were unable to distinguish these diacetylated H4 species, and thus, we presented their combined stoichiometry.

#### Protein Turnover Analyses

Peptide turnover rates, both acetylated and native, were quantified using *in vivo* ^2^H_2_O–metabolic labeling approach (36). Turnover rates measured via ^2^H_2_O metabolic labeling are sensitive to the spectral accuracy including both monoisotopic and heavy isotopomer signals, with increased measurement errors in lower-intensity ions. For kinetic analysis, we focused on data from unique peptides with relative higher ion intensities (>10^5^). Selection criteria for acetylated peptides included optimal chromatographic properties, sufficient separation from isobaric peptides, consistent elution times, and Gaussian peak shapes, ensuring accurate quantification of acetylated peptides. Consequently, only quantifiable modification sites were selected to analyze turnover rates of acetylated proteins.

With ^2^H_2_O metabolic labeling, during protein synthesis, ^2^H labeled amino acids incorporation during protein synthesis redistributes the isotope pattern, increasing heavy isotopomers (*M*_*1*_*, M*_*2*_*, M*_*3*_, and so on) relative to the light monoisotopic isotopomer (*M*_*0*_). The custom software d2Ome analyzes spectral data (mzML) and search results (mzIdentML) to detect, integrate, and extract relative isotopomer abundances (34). The relative abundance of *M*_*0*_ at a given time point *t* (*E*_*0*_*(t)*) is calculated as the ratio of *M*_*0*_ intensity to the total intensity of all isotopomers:

(1) $$\begin{array}{c} E_{0}\left(t\right)=M_{0}\left(t\right)/\sum_{j=0}^{n}M_{j}\left(t\right) \end{array}$$

Since the increase in heavy isotopomers resulting from ^*2*^*H* incorporation is directly linked to the reduction in monoisotopic peak intensity, the total ^*2*^*H* labeling, *E(t)*, can be expressed as:

(2) $$\begin{array}{c} E\left(t\right)=1-E_{0}\left(t\right)=\sum_{j=1}^{n}M_{j}\left(t\right)/\sum_{j=0}^{n}M_{j}\left(t\right) \end{array}$$

The turnover rate constant (*k*) was estimated using a one-compartment model by fitting an exponential growth curve to the ^*2*^*H* labeling values of tryptic peptides, *E(t)*, over time (*t*):

(3) $$\begin{array}{c} E\left(t\right)=E^{\left(0\right)}+\left(E^{\left(ss\right)}-E^{\left(0\right)}\right)\times \left(1-e^{-kt}\right) \end{array}$$

where $E^{\left(0\right)}$ and $E^{\left(ss\right)}$ are the baseline and plateau enrichments, respectively. The turnover rates of native peptides are treated as repeated measures representing the turnover rate of their corresponding protein. Therefore, the time-course data of native peptides from the same protein were modeled collectively to estimate the protein's turnover rate. Details of the kinetic model are provided in the Statistical Analysis of Kinetic Data section of the Supplementary Materials. The half-life (*t*_*1/2*_) of a peptide was calculated based on the turnover rate constant *k* as:

(4) $$\begin{array}{c} t_{1/2}=\frac{\ln\left(2\right)}{k} \end{array}$$

Acetylation's impact on protein stability was evaluated by comparing turnover rates between native and acetylated peptides in a site-specific manner. Each acetylated lysine site was analyzed individually and the time-course data of acetylated peptides covering the same site were modeled jointly as in the analysis of native proteins. Estimation of turnover rate was performed individually for each site. Acetylation's impact on protein stability was evaluated by comparing turnover rates between native and acetylated peptides.

#### Statistical Analysis

To assess the alcohol-induced relative change in EF mice based on non-MS measurements, results were normalized to the mean in the control (PF) mice, and the significance of the change was evaluated using two-sample *t* test. For the analysis of LFQ data, differences in total protein abundance or acetylated peptide abundance (on log_2_ scale) between the PF and EF groups were analyzed using two-sample *t* test. For kinetic analysis, a time course of the total ^2^H-labeling based on experiments with six mice was constructed for each peptide. The kinetic data were used to evaluate the effect of alcohol on 1) the turnover rate of the native proteins and 2) the turnover rate of an acetylated peptide at the specific lysine site, where changes in turnover rates were assessed using an *F*-test (36) (see details in Supplementary Methods). Both differential abundance and turnover rate analyses conducted multiple testing correction, using the Benjamini–Hochberg procedure (56) to control the FDR. A change with an FDR-adjusted *p*-value <0.05 was considered statistically significant. The statistical analyses were performed using R (57). The R script for reproducing all the analysis results and figures reported in this manuscript is available at https://github.com/tsunghengtsai/ald-turnover.

#### Functional Enrichment Analysis

Following each significance analysis, functional enrichment analysis was performed using clusterProfiler (58, 59), where proteins with statistically significant changes (adjusted *p*-value <0.05) were used as a query to search for enriched Gene Ontology (GO) terms of biological process. Proteins with increased and decreased abundance (or turnover) were searched separately against a custom background of liver tissue proteins from two recent studies measuring protein expressions (60) (n = 6160) and turnover rates (61) (downloaded from the MassIVE identifier number MSV000086426). Overrepresented terms were determined by the hypergeometric test, followed by a multiple testing correction procedure with an FDR cutoff of 0.05. Redundant GO terms were assessed and removed using GOSemSim (62). In addition to the separate searches, we performed the functional enrichment analysis using all the significant proteins, regardless of their fold change directions, to identify significantly enriched GO terms and explore their relationship to one another and with associated proteins.

## Results

### Biochemical characteristics

**Fig. 1:**
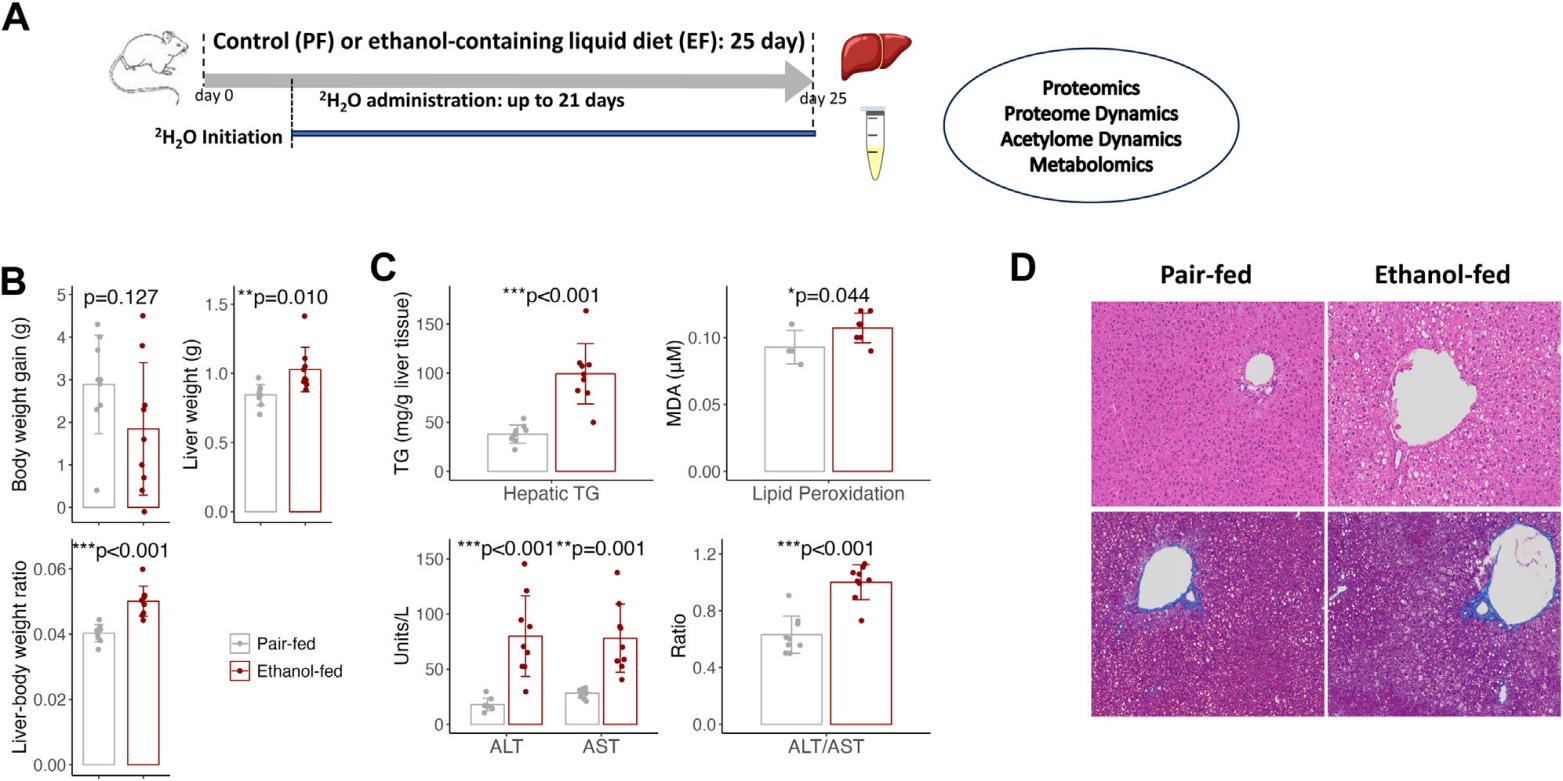
**Chronic alcohol (ethanol) intake results in hepatic steatosis and liver injury.***A*, study design for the alcohol-feeding paradigm and ^2^H_2_O–based turnover analysis. Eight-week-old female mice (n = 9/group) were fed an ethanol-containing (EF group) or isocaloric (PF group) liquid diet for 25 days. A ^2^H_2_O–based turnover study was initiated with a bolus ^2^H_2_O saline (20 μl/g of body weight) injection, followed by 6% ^2^H_2_O in drinking water. At the end of study, mice were euthanized, and liver and terminal plasma samples were analyzed (see “Experimental Procedures”). *B*, chronic alcohol consumption increases liver weight and liver-to-body weight ratio without affecting overall body weight gain compared to the pair-fed group. *C*, chronic alcohol intake induces triglyceride (TG) accumulation, oxidative stress in the liver, and increased ALT, AST levels, and ALT/AST ratio in plasma. Along with individual data points (n = 9/group for TG, ALT, AST; n = 7/group for MDA), bar graphs represent mean ± SD. *D*, representative H&E (*top*)- and trichrome (*bottom*)-stained liver sections (20x). Livers from EF mice show fat accumulation and mild inflammation, absent in PF mice. ALT, alanine aminotransferase; AST, aspartate aminotransferase; EF, ethanol-fed; MDA, malondialdehyde; PF, pair-fed.

Figure 1*A* illustrates the chronic ethanol-feeding protocol and the ^2^H_2_O–based metabolic labeling experiments in mice. Female mice were pair-fed an isocaloric liquid diet with (EF) or without ethanol (PF). After an 18-day escalation to 5% ethanol, EF mice were fed a 6% ethanol diet for 1 week to achieve a blood alcohol level approximately 50 mM, providing 32% of their caloric intake (63). EF mice had significantly higher liver weight (*p = 0.010*) and liver-to-body weight ratio (*p < 0.001*) despite similar weight gain. Chronic ethanol exposure led to hepatic triglyceride accumulation, oxidative stress, increased alanine aminotransferase and aspartate aminotransferase, steatosis, and mild inflammation (Fig. 1, *B*–*D*).

### Chronic Alcohol Consumption Selectively Impacts Protein Expression and Dynamics Across Cell Compartments

**Fig. 2:**
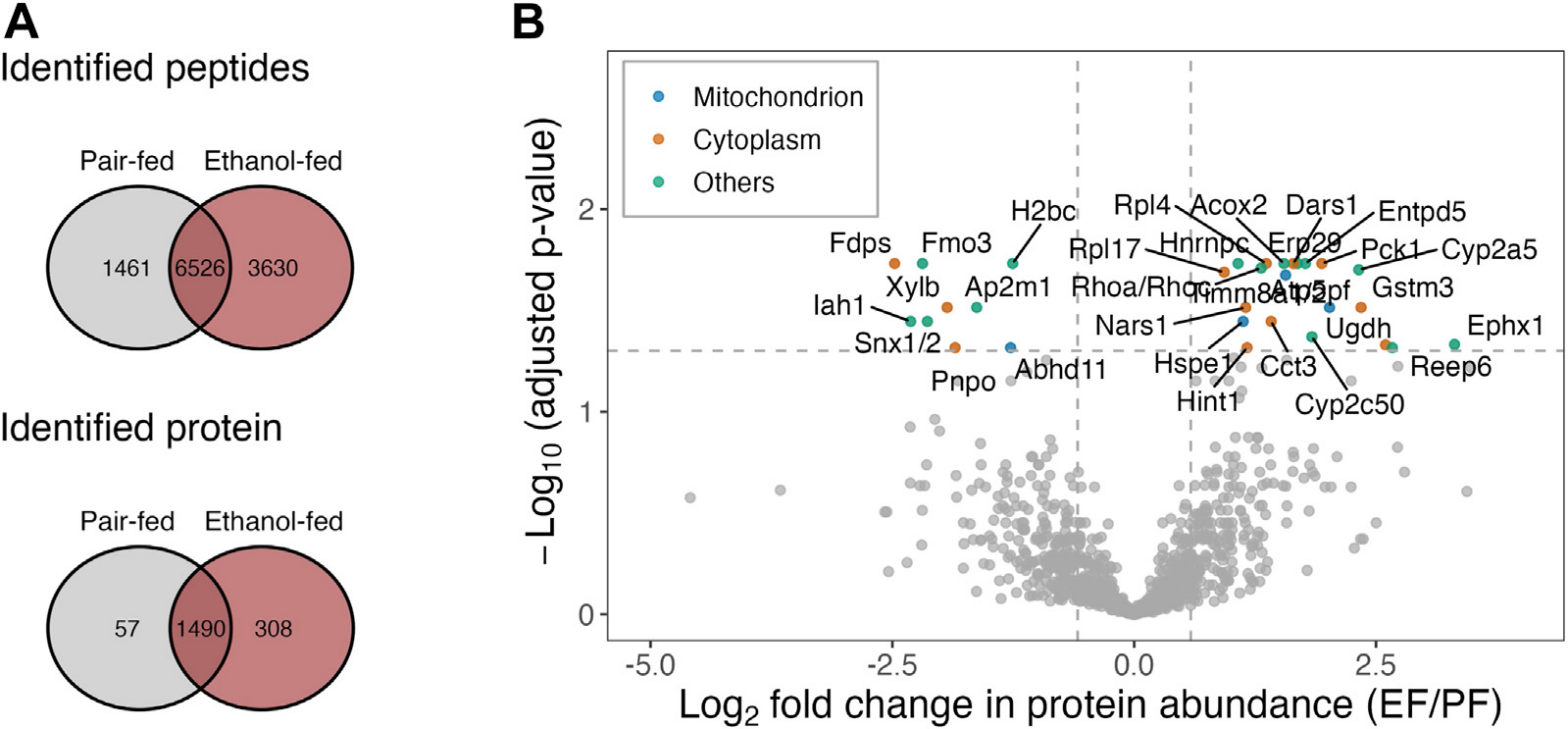
**Effect of chronic alcohol consumption on hepatic proteome expression.***A*, Venn diagram showing the number of common and unique peptides and proteins identified in EF and PF mouse livers. *B*, volcano plot of the label-free quantification (LFQ) illustrating differential protein expression between EF and PF groups, with log_2_ fold changes (*x*-axis) and −log_10_ adjusted *p*-values (*y*-axis). Each *dot* represents a protein. The horizontal *dashed line* indicates the FDR-adjusted *p-*value 0.05 threshold and vertical *dashed lines* indicate a 50% increase or decrease. Upregulated and downregulated proteins from mitochondria, cytosol, and other organelles with an adjusted *p*-value <0.05 are shown in *blue*, *orange*, and *green* colors, respectively. EF, ethanol-fed; FDR, false discovery rate; PF, pair-fed.

The accumulation of intracellular proteins, along with fat and water, contributes to alcohol-induced liver enlargement (2). We used differential and dynamic proteomics to assess alcohol's impact on hepatic proteostasis. We identified 1547 proteins in the PF group and 1798 in the EF group, with 1490 shared (6526 peptides, Figure 2*A*). LFQ of 1355 proteins (supplemental Table S1*A*) showed alcohol significantly altered 30 proteins (adjusted *p* < 0.05), with more upregulated than downregulated (21 *versus* 9) (Fig. 2*B*, supplemental Table S1*B*). Upregulated proteins from various cell compartments—cytosol, endoplasmic reticulum, peroxisome, nucleus, and mitochondria—were primarily associated with protein synthesis, folding, and secretion (Rpl4, Rpl17, Dars1, Nars1, Tom8a, Ccct3, Erp29, and Hnrnpc), fatty acid oxidation (Acox2), gluconeogenesis (Pck1), and xenobiotic and oxidative stress (Gstm3 and Ugdh). In contrast, downregulated proteins, primarily cytoplasmic and microsomal, were linked to nitrogen compound oxygenation (Fmo3), intracellular and membrane trafficking (Snx1/2 and Ap2m1), and lipid metabolism (Fdps, Iah1, and Xylb).

**Fig. 3:**
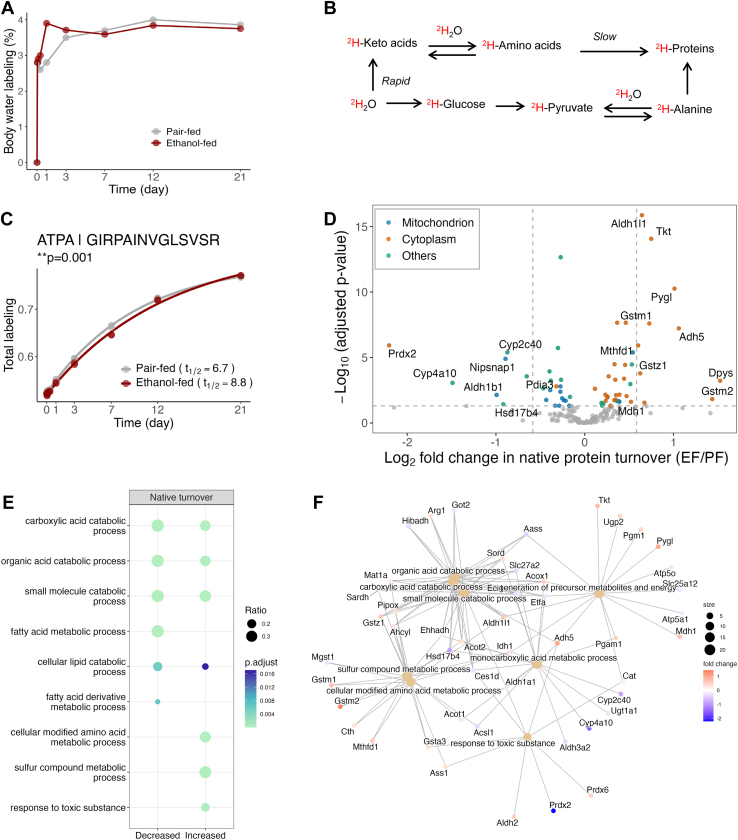
**Effect of chronic alcohol consumption on hepatic proteome dynamics with ^2^H_2_O metabolic labeling approach.***A*, time-course ^2^H_2_O labeling of body water in pair-fed (PF) and ethanol-fed (EF) mice. *B*, ^2^H_2_O rapidly labels proteogenic amino acids with deuterium (^2^H), enabling quantification of hepatic protein turnover. *C*, time-course ^2^H labeling of the GIRPAINVGLSVSR peptide, unique to ATPA, an ATP synthase subunit, allows calculation of its turnover rate in mouse liver. ∗*p* < 0.05, *F*-test (see statistical analysis). *D*, volcano plot showing differential turnover rates of hepatic proteins between EF and PF groups, with log_2_ fold changes (*x*-axis) and −log_10_ adjusted *p*-values (*y*-axis). Proteins with significantly altered turnover rates (*p* < 0.05) are colored. Significant proteins with at least 50% change labeled by gene name. *E*, gene Ontology (GO) enrichment analysis of proteins with alcohol-induced altered turnover changes. Significantly enriched GO terms associated with native proteins with increased or decreased turnover rates are shown separately in the dot plot, with circle size representing the ratio of significant proteins in each term. *F*, gene-concept network of the significantly enriched GO terms and associated proteins with significant turnover rate changes. Proteins are color-coded based on their log_2_ fold changes.

To investigate alcohol-induced changes in the hepatic proteome, we measured protein turnover rates using a ^2^H_2_O–metabolic labeling approach (64) (Fig. 1*A*). Mice received a ^2^H_2_O–saline injection and 6% ^2^H_2_O in drinking water, achieving 3.5 ± 0.6% and 3.5 ± 0.5% body water enrichment in the PF and EF groups, respectively (Fig. 3*A*). ^2^H_2_O rapidly labels proteogenic amino acids (Fig. 3*B*) (43), allowing synthesis rates of individual proteins to be determined via mass spectrometry (supplemental Fig. S1*A*). For example, time-course labeling of the ATP Synthase subunit A peptide shows a 25% decrease (*p < 0.05*) in its turnover with alcohol exposure (Fig. 3*C*). Using this method, we quantified turnover rates for 198 proteins, with 185 in both groups (supplemental Table S2*A*). Alcohol did not significantly alter the average protein turnover rate (supplemental Fig. S1*B*), despite significant changes in multiple individual proteins (Fig. 3*D*, supplemental Table S2*B*). The turnover method identified more proteins with altered turnover (∼31.8%) compared to LFQ (∼2.2%), demonstrating higher sensitivity. Of the 63 significantly changed proteins, 38 showed increased and 25 decreased turnover rates (Fig. 3*D*, supplemental Table S2*B*).

To understand alcohol-induced changes in protein turnover, we classified proteins by subcellular localization. Alcohol exposure had contrasting effects on mitochondrial and cytosolic proteins: reduced turnover of mitochondrial proteins involved in fatty acid β-oxidation (Acsl5 and Acsm1) and ATP synthesis (Atp5f1a and Atp5po) and increased turnover of cytosolic enzymes in amino acid (Arg1), alcohol (ADH1 and ADH5) and one-carbon metabolism (Mat1a and Mthfd1) (Fig. 3*E* and supplemental Fig. S2). Functional analysis linked decreased turnover to carboxylic acid and small molecule catabolism, and energy metabolism while increased turnover was associated with alcohol, amino acid, sulfur metabolism, and toxin response (Fig. 3, *E* and *F*, supplemental Table S2*C*).

### Chronic Alcohol Consumption Uniquely Alters Protein Acetylation in Specific Organelles

**Fig. 4:**
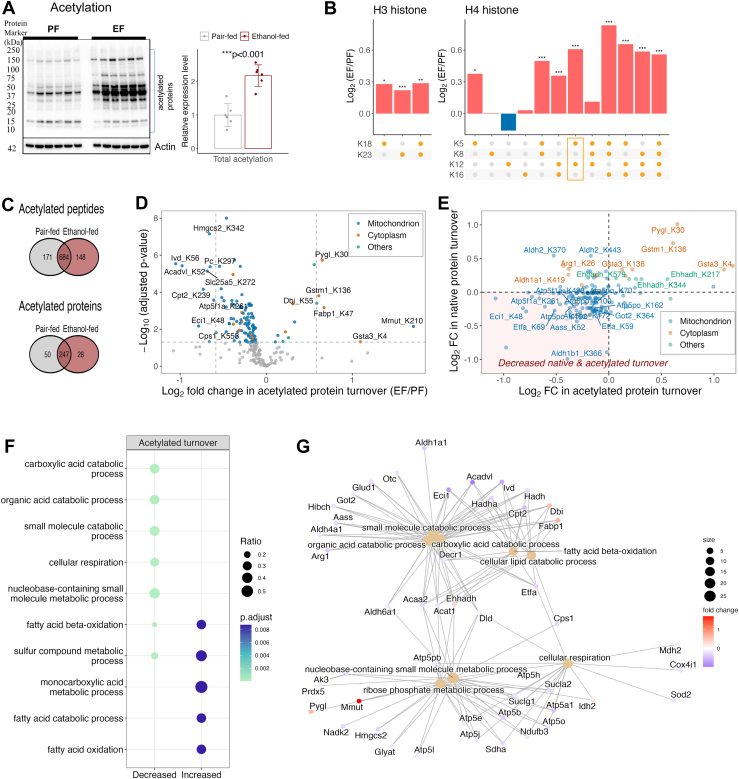
**Chronic alcohol intake uniquely impacts acetylation in different cell compartments.***A*, global acetylation of hepatic proteins in mice. Lysine-acetylated proteins from PF and EF liver homogenates were extracted and probed with pan acetyl-lysine antibody. Gel images were quantified by densitometry using ImageJ 1.41 software, with normalized intensities shown in bar graphs for all replicates (n = 6) where the error bar represents SD. *B*, alcohol increases the acetylation stoichiometry of H3 and H4 histones. Data were analyzed by two sample *t* tests comparing PF and EF groups (n = 6), with statistical significance annotated as ∗*p* < 0.05, ∗∗*p* < 0.005, ∗∗∗*p* < 0.001. An acetylation form of H4, nondistinguishable among K5acK12ac, K5acK16ac, K8acK12ac, and K8acK16ac, is highlighted in the panel of acetylation code. *C*, Venn diagrams show acetylated non-histone proteins and peptides identified in PF and EF groups: 855 acetylated peptides (297 proteins) in PF and 832 peptides (275 proteins) in EF, with 684 common peptides (247 proteins). *D*, volcano plot of alcohol-related changes in turnover rates for 223 acetylated sites of non-histone proteins. Each colored dot represents a significantly altered acetylated site with color indicating the subcellular localization. Significant acetylated sites with at least 50% change are labeled with their gene names and site locations. The *x*-axis shows the fold change in EF turnover rate relative to PF (log2 scale), and the *y*-axis shows the FDR-adjusted *p*-value (−log10 scale). The *p*-values are based on the *F*-test to evaluate the change in turnover rate of the acetylated sites between EF and PF groups. Alcohol significantly altered 116 of 223 quantified site-specific acetylated peptide turnover rates (adjusted *p* < 0.05): 15 increased (10 proteins) and 101 decreased (58 proteins). *E*, association between alcohol-induced changes in acetylated and native protein turnover rates. *Dots* represent acetylated sites; 24 sites on 14 proteins show significant changes in both acetylated and native protein turnover rates. These sites are labeled with their gene names and site locations. The *x*-axis shows the fold change in acetylated protein turnover in the EF group relative to PF control (log_2_ scale), and the *y*-axis shows the alcohol-related fold change in native protein turnover (log_2_ scale). *F*, gene Ontology (GO) enrichment of proteins with alcohol-induced altered acetylated protein turnover rates, displayed in a dot plot with an *x*-axis represents increased and decreased turnover rates of acetylated proteins. The size of each *circle* represents a ratio of the number of significant proteins in the associated term. *G*, gene-concept network of significantly enriched GO terms and associated proteins. EF, ethanol-fed; FDR, false discovery rate; PF, pair-fed.

Because alcohol consumption induces posttranslational acetylation (10, 12), which affects proteostasis (36, 65), we assessed global acetylation of hepatic proteins using acetyl-specific immunoblots. Chronic alcohol intake more than doubled global acetylation in EF mouse livers (Fig. 4*A*). To identify proteins with altered acetylation, we analyzed histone and non-histone proteins separately by mass spectrometry. Due to high acetylation, acetylated histones were analyzed with native peptides, while low-abundant acetylated non-histone peptides were immunoenriched. Histones were acid-extracted from the nuclear fraction, and nonmodified lysins were derivatized with acetic anhydride-d6 for mass spectrometry analysis (66). This strategy enhances precision by providing comparable ionization characteristics and boosts confidence in identifying low-abundant acetylation sites using isotopic mass shifts. However, it also resulted in the co-elution of endogenously acetylated and native peptides during chromatography, complicating their quantification based on MS1 ions (supplemental Fig. S3, *A* and *B*). We identified multiple acetylated peptides from core (H2A, H2B, H3, and H4), linker (H1) histones and their variants. H2B was acetylated at K13, and H3 was acetylated at K18 and K23, including diacetylation at both sites (supplemental Fig. S3, *A* and *C*). H4 showed multiacetylated forms at K5, K8, K12, and K16 (supplemental Fig. S3, *B* and *D*).

The MS2 spectrum of H3 peptide (18KQLATKAAR26) isoforms effectively distinguished between the single-acetylated forms of H3K18ac and H3K23ac, allowing quantification of acetylation stoichiometry by comparing the intensities of b2 ions from the native (^2^H_3_C-acetyl) and endogenous (H_3_C-acetyl) peptides (supplemental Fig. S4). MS2 peptide sequencing also facilitated the quantification of all positional isomers of H4 (4GKGGKGLGKGGAKR17) peptide based on the y5, y7, and y12 ions, as well as the corresponding monoacetylated, diacetylated, and triacetylated forms (supplemental Fig. S5, *A*–*C*). However, the diacetylated species H4K5acK12ac, H4K5acK16ac, H4K8acK12ac, and H4K8acK16ac could not be individually quantified, for which we present their combined abundance values. Alcohol exposure increased all quantified monoacetylated and diacetylated H3 forms and significantly elevated acetylation at multiple H4 lysine sites, except monoacetylated K8, K12, and K16 and triacetylated K5K8K12 forms, which remained unchanged (supplemental Table S3*A*, Fig. 4*B*).

We also identified 325 acetylated non-histone proteins, with 247 shared between PF and EF groups (Fig. 4*C*). These proteins comprised 1003 peptides, of which 684 were common to both groups. It is important to note that our study detected fewer acetylated peptides and proteins compared to previous studies that achieved deep coverage of the lysine acetylome (67, 68). This discrepancy is most likely due to the smaller amount of starting material, the absence of offline fractionation, and the shorter LC-MS acquisition time. Consistent with the immunoblot findings (Fig. 4*A*), label-free quantification of 172 acetylated peptides (supplemental Table S4*A*) revealed hyperacetylation of 112 peptides from 40 proteins in the EF group, primarily in mitochondria (supplemental Fig. S6*A*, supplemental Table S4*B*), though many changes were not statistically significant. Additionally, decreased acetylation was observed at 60 sites on 38 proteins, most of which were also insignificant (supplemental Fig. S6*A*, supplemental Table S4*B*). Alcohol significantly increased acetylation of the mitochondrial proteins, CPT2 and HADHa, at lysine residues 418 and 540, respectively (adjusted *p* < 0.05), while decreasing acetylation of carbamoyl phosphate synthase 1 (CPS1), at lysine 307 in EF compared to PF mice (supplemental Fig. S6*A*, supplemental Table S4*B*). Many acetylated proteins are critical for metabolic processes like the urea cycle, TCA cycle, amino acid metabolism, fatty acid oxidation, and oxidative phosphorylation. Similar to our recent study high-fat diet effects on mitochondrial acetylation (36), we found that CPS1, a urea cycle enzyme, was acetylated at multiple lysine sites (supplemental Table S4*B*). A weak, positive correlation between acetylation and protein abundance was observed (supplemental Fig. S6*B*), where many mitochondrial proteins showed increased abundance with higher acetylation. There appeared also a positive association between the detection of acetylation modifications and total protein quantification (supplemental Fig. S6*C*), which suggests that the quantitative analysis of acetylation in this study may be overrepresented by high-abundance proteins, likely due to the challenge in acetylation quantification.

### Alcohol-Induced Changes in Organelle-Specific Protein Turnover Are Linked to Compartmentalized Acetylation

To assess the impact of alcohol-induced acetylation changes on proteostasis, we examined the effect of acetylation on protein turnover with ^2^H_2_O-labeling approach (36). Turnover rates for acetylated peptides were determined using time-course ^2^H-labeling and nonlinear regression, allowing us to quantify kinetics of 263 acetylated peptides from 96 non-histone proteins and core histones (H3 and H4). To evaluate the effect of alcohol-related acetylation on non-histone protein stability, we measured the turnover rates of acetylated and native peptides from the same proteins. For example, acetylation reduced the turnover rates of ATPA, shown by slower labeling of the acetylated peptide STVAQLV^Ac^kR compared to the native peptide STVAQLVKR in both PF and EF groups (supplemental Fig. S7*A*). This resulted in a significantly longer half-life for acetylated ATPA, especially in PF mice (*t*_*1/2*_ = 3.9 days for native *versus t*_*1/2*_ = 9.6 days for acetylated, *p <* 0.01). Acetylation also slowed the turnover of many other non-histone proteins (supplemental Fig. S7*B*), particularly mitochondrial ones, in the EF group compared to PF controls (Fig. 4*D*, supplemental Table S5*B*). In contrast, alcohol significantly increased the turnover rates of acetylated cytosolic peptides from acyl-CoA binding protein (Dbi1K55), fatty acid binding protein 1 (Fabp1K47), glycogen phosphorylase L (PyglK30), glutathione S-transferase 3 (Gsta3K4), and glutathione S-transferase mu 1 (Gstm1K136), proteins involved in glycogen and lipid metabolism and cellular defense (Fig. 4*D*). Similarly, alcohol enhanced the turnover of acetylated peptides from enoyl-CoA hydratase and 3-hydroxyacyl CoA dehydrogenase (Ehhadh), a peroxisomal fatty acid oxidation enzyme, which also showed an increased turnover rate (Fig. 4*D*, supplemental Table S5*B*). Consistent with previous reports (37, 38), acetylation increased turnover rates of histone proteins in both groups (supplemental Table S3*B*, supplemental Fig. S8). Notably, multiple acetylation on the same H3 (18KQLATKAAR26) and H4 (4GKGGKGLGKGGAKR17) peptides had an additive effect, accelerating turnover. Alcohol reduced turnover rates for mono- (K23) and di- (K18K23) acetylated H3 (supplemental Fig. S8*A*), as well as for mono- (K12), di- (K12K16), and tetra- (H4K5K8K12K16-4Ac) acetylated forms of H4 (supplemental Fig. S8*B*).

Given that acetylation altered stability (*t*_*1/2*_) of many proteins, we tested whether alcohol-induced changes in protein levels and turnover in EF mice were related to acetylation. For this purpose, we correlated EF-related changes in 1) native protein turnover (Fig. 3*D*, supplemental Table S2*B*) with acetylated protein turnover (Fig. 4*D*, supplemental Table S5*B*), 2) acetylated protein turnover with acetylation levels (supplemental Fig. S6*A*, supplemental Table S4*B*), 3) acetylated protein turnover with protein abundance (Fig. 2*B*, supplemental Table S1*B*), and 4) acetylation levels with protein abundance. Figure 4*E* shows that alcohol decreased turnover in both acetylated and native proteins for 31 of 55 quantified acetylated proteins, primarily mitochondrial (n = 28). This suggests alcohol-induced reductions in mitochondrial protein turnover are linked to acetylation, confirmed by correlations between decreased turnover, increased acetylation (supplemental Fig. S9*A*), and protein abundance (supplemental Fig. S9*B*). Although the correlation between acetylation and protein abundance was weaker (supplemental Fig. S6*B*), many mitochondrial proteins showed increased abundance with higher acetylation. Conversely, the increased turnover of cytosolic acetylated peptides (PyglK30, Gsta3K4, and Gstm1K136) associated with higher turnover rates of their native proteins (top-right corner in Fig. 4*E*). These findings indicate that alcohol-induced changes in protein turnover and levels are related to acetylation, highlighting acetylation's role in proteostasis and its subcellular-specific effects (Fig. 4*E* and supplemental Fig. S9, *A* and *B*). The heat map in Figure S9C illustrates that ethanol-induced acetylation impacts turnover rates and causes of mitochondrial protein accumulation in EF mouse livers.

To determine if acetylation had similar stabilizing effects on metabolic enzymes with distinct functions, we grouped them by metabolic roles and performed functional enrichment analysis using clusterProfiler. Consistent with similar analysis on total protein expression and native turnover, mitochondrial proteins with decreased turnover of acetylated peptides were significantly enriched in carboxylic acid catabolism and cellular respiration, while proteins with increased turnover of acetylated peptides were enriched in peroxisomal fatty acid β-oxidation and cytosolic sulfur compound metabolism (Fig. 4*F*, supplemental Table S5*C*). Many of these acetylated proteins participate in cellular respiration, nucleotide metabolism, and fatty acid β-oxidation, as shown in the gene-concept network (Fig. 4*G*).

### Impaired Proteasomal and Lysosomal Degradation Lead to Alcohol-Induced Hepatic Protein Accumulation

**Fig. 5:**
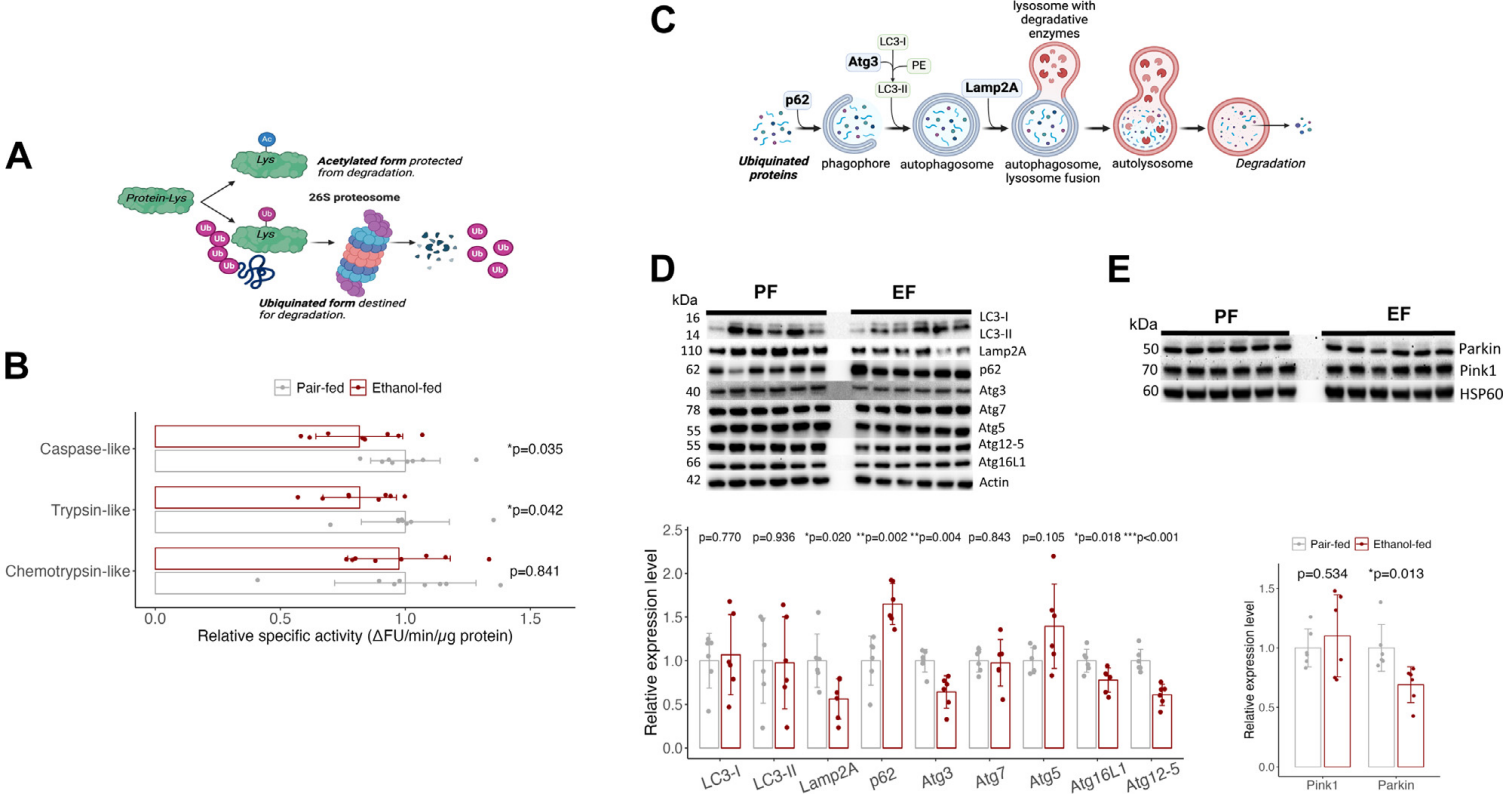
**Chronic alcohol-induced hepatic protein accumulation is related to impaired ubiquitin-proteasome system (UPS) activity and impaired autophagy/mitophagy.***A*, schematic illustrating potential competition between acetylation and ubiquitination at the same lysine site, possibly hindering protein degradation by the UPS (created with Biorender.com). *B*, proteasome activity in liver homogenates from PF and EF mice was assessed using fluorescent-tagged peptide substrates for trypsin, chymotrypsin, and caspase, normalized to total tissue protein. *C*, simplified autophagy pathway diagram (created with Biorender.com). *D*, western blot analysis of autophagy markers (P62, LC3-I/II, Atg3, Atg5, Atg7, Atg12, and Atg16L1) in liver homogenates from PF and EF mice, with bar graphs showing normalized quantification to β-actin. *E*, western blot analysis of mitophagy markers (Parkin and Pink1) in PF and EF mouse liver, normalized to HSP60. *B*, *D*, and *E*, Bar graphs show individual data points and mean ± SD. n = 6, Data were analyzed by two sample *t* tests (∗*p* < 0.05, ∗∗*p* < 0.005, ∗∗∗*p* < 0.001). EF, ethanol-fed; PF, pair-fed.

Chronic ethanol consumption inhibits proteolysis in rat livers (69). Since posttranslational acetylation impacts protein stability (36), we investigated the roles of different protein degradation pathways in this process. The ubiquitin-proteasome system (UPS) targets damaged soluble proteins for degradation, while lysosomal autophagy degrades long-lived proteins, organelles, and protein aggregates. Since acetylation may enhance protein stability by competing with ubiquitination for degradation (Fig. 5*A*) (9), we evaluated total hepatic protein ubiquitination using an anti-ubiquitin antibody to assess the UPS involvement. Alcohol exposure did not significantly affect ubiquitinated proteins or free ubiquitin (supplemental Fig. S10*A*). This may reflect either decreased proteasomal degradation due to impaired ubiquitination and proteolytic activity (70, 71) or increased flux in the pathway with efficient monoubiquitin recycling (72, 73). To further investigate the role of UPS in protein accumulation in EF mouse liver, we measured proteasome activity using chymotrypsin-like, trypsin-like, and caspase-like substrates, with and without the proteasome inhibitor MG132 (100 μM), which completely inhibited all activities (supplemental Fig. S10*B*). Alcohol intake significantly reduced trypsin and caspase activities (Fig. 5*B*), suggesting that impaired proteasomal degradation contributes to hepatic protein accumulation.

To evaluate the role of autophagy in alcohol-induced protein accumulation, we measured several autophagy markers: p62, Atg3, Atg5, Atg7, LC3, and Lamp2A. p62 binds ubiquitinated cargo for degradation and interacts with LC3, which is crucial for autophagosome formation. Atg3 is vital for converting LC3-I to LC3-II, aiding autophagosome membrane expansion and protein engulfment, while Lamp2A facilitates autophagosome-lysosome fusion and degrades damaged proteins via chaperone-mediated autophagy (Fig. 5*C*). Although alcohol did not significantly alter Atg5, Atg7, LC3-I, or LC3-II levels, we observed significant decreases in Atg3 and Lamp2A expression (Fig. 5*D*), suggesting impaired autophagy flux in EF mice, with p62 accumulation due to reduced autophagic activity.

Given that alcohol increased acetylation and decreased the turnover of primarily mitochondrial proteins, we assessed Pink1 and Parkin, critical regulators of mitochondrial quality control through mitophagy. Pink1 accumulates on the mitochondrial outer membrane in response to depolarization or damaged proteins, phosphorylating Parkin, which then ubiquitinates outer membrane proteins to mark mitochondria for degradation (74). Chronic alcohol exposure significantly reduced Parkin levels, while Pink1 levels remained unchanged (Fig. 5*E*). These results suggest that, in addition to impaired UPS activity, reduced autophagy and mitophagy contribute to alcohol-induced hepatic proteopathy.

### Alcohol Metabolism Contributes to Acetyl-CoA Pool for Acetylation

**Fig. 6:**
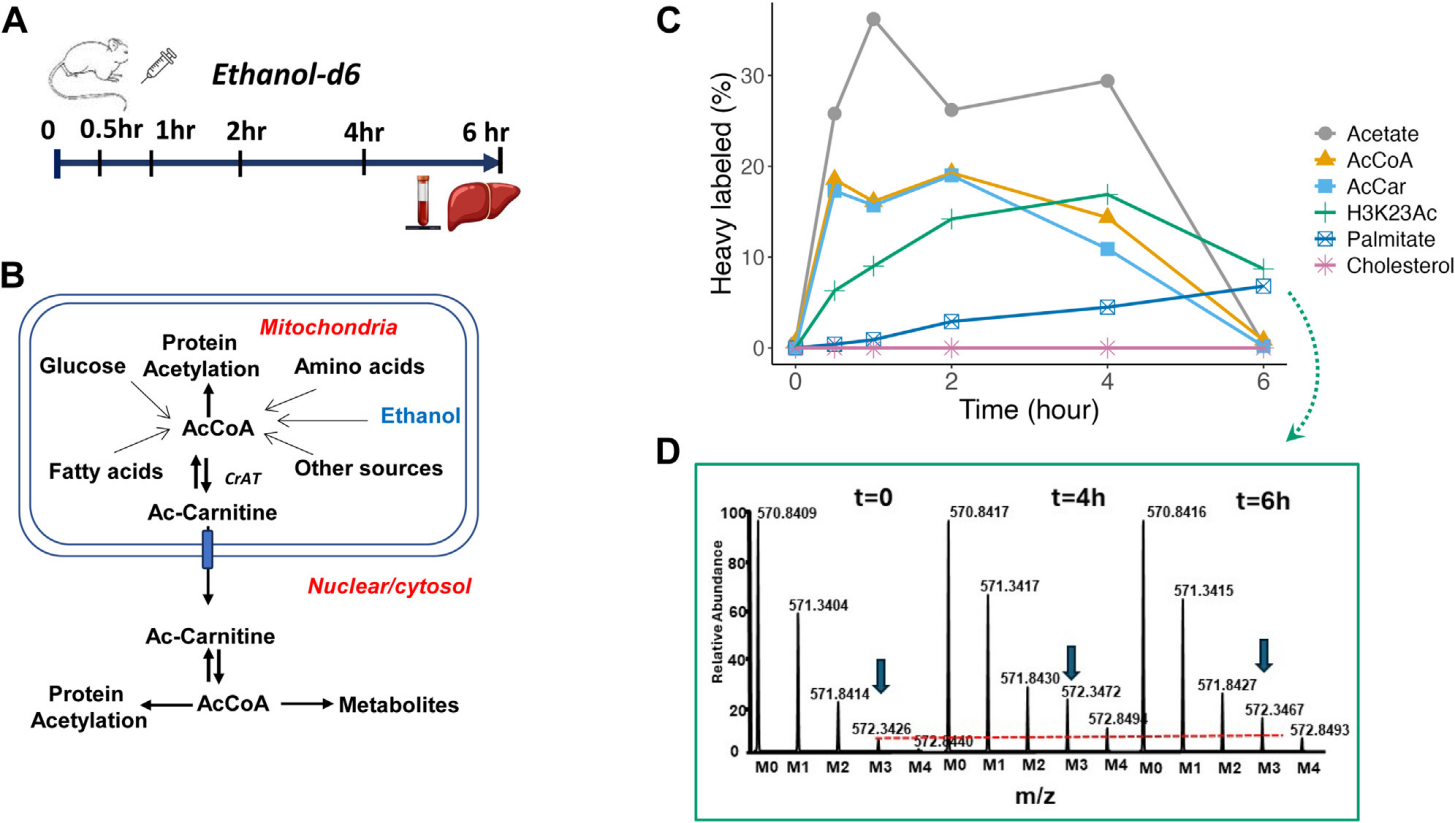
**Ethanol metabolism contributes to histone acetylation and fatty acids synthesis in mouse liver.***A*, experimental design for *in vivo* isotope tracing of alcohol metabolism using ethanol-d6 in mouse liver. Mice were injected intraperitoneally with ethanol-d6 (2.0 μl/g body weight) or saline and euthanized at various time points to collect liver and plasma samples. *B*, metabolic scheme illustrates acetyl-CoA generation from various substrates, including ethanol, in mitochondria and its role in anabolic pathways and protein acetylation. *C*, time-course labeling of acetate and downstream metabolites from ethanol-d6. The decreasing trend in labeling from acetate to acetyl-CoA/acetylcarnitine and acetyl histone at early time points indicate that ethanol-d6-derived acetate-d3 contributes to acetyl-d3-histone formation. The slow labeling of palmitate and the lack of cholesterol labeling suggest that ethanol-derived acetyl-CoA is primarily used for acetylation and fatty acid synthesis (n = 6, each data point represents 1 mouse). *D*, representative high-resolution mass spectra of H3 histone peptide 18KQLATkAAR26, acetylated at lysine 23 with acetyl-d3 from ethanol-d6 metabolism in mouse liver. *Arrows* indicate changes in the M3 isotopomer due to d3-label incorporation, reflecting the acetyl-d3 form of the peptide.

Given that lysine acetylation regulates protein degradation and acetyl-CoA availability is essential for this process (6, 65), we explored the impact of alcohol-derived acetyl-CoA. We administered ethanol-d6 to WT mice and analyzed the labeling of acetate, acetyl-CoA, acetyl-carnitine, acetyl-histone, and lipid metabolites such as palmitate and cholesterol using mass spectrometry (Fig. 6*A*). Ethanol metabolism contributes to the mitochondrial acetyl-CoA pool, while CrAT converts excess acetyl-CoA to acetyl-carnitine, which can cross the mitochondrial membrane, reducing mitochondrial acetyl-CoA levels and supporting anabolic pathways and protein acetylation (Fig. 6*B*).

Ethanol-d6 rapidly metabolized in the liver, contributing 25% of total acetate after 30 min and peaking at 37% after one-hour (Fig. 6*C*). The isotopic ^2^H-labeling of acetyl-CoA and acetyl-carnitine had nearly identical enrichment at 20% from 0.5-h-post administration to 2-h post-administration, declining by 4 h and ceasing by 6 h. We also traced ^2^H-labeling in acetylated histones. No ^2^H-label was detected in native peptides (supplemental Fig. S8). However, consistent with previous report (13), ^2^H-labeling gradually increased in multiple acetylated peptides, including H3 histone peptide KQLATkAAR (Fig. 6*C*, green line, Fig. 6*D* and supplemental Fig. S11). ^2^H-labeling gradually increased, peaking at ∼18% after 4 h and retaining approximately 10% after 6 h. Furthermore, ethanol-d6-derived acetyl-CoA was slowly incorporated into palmitate, aligning with its slow turnover rate. No ^2^H-label was found in cholesterol, suggesting that alcohol-derived acetyl-CoA is primarily diverted toward acetylation and fatty acid synthesis rather than cholesterol biosynthesis. These results indicate that alcohol-derived acetyl-CoA equilibrates with the mitochondrial acetyl-carnitine pool, supporting cytosolic and nuclear acetyl-CoA pools crucial for lipogenesis and acetylation.

### Acetylcarnitine Shuttling Links Alcohol Metabolism to Histone Acetylation

**Fig. 7:**
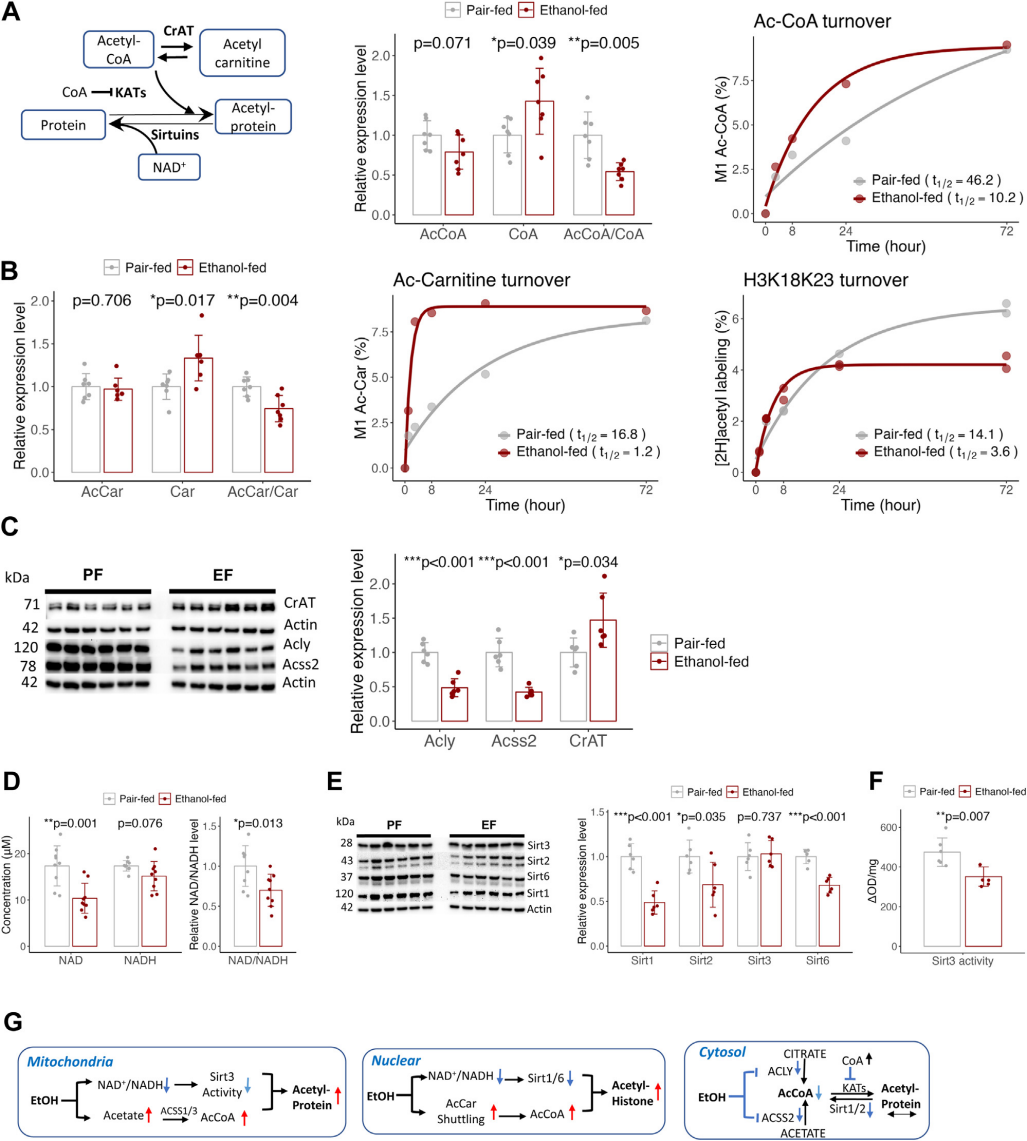
**The causes of chronic alcohol-induced acetylation in different cellular compartments.***A*, schematic of acetylation and deacetylation (*left*). Acetyl-CoA and NAD^+^ availability stimulate KAT-catalyzed and NAD^+^-dependent sirtuins-catalyzed deacetylation, respectively. Free CoA inhibits KATs. The *middle* panel shows the impact of chronic alcohol intake on hepatic acetyl-CoA, free CoA levels, and the acetyl-CoA/CoA ratio, along with acetyl-CoA turnover quantified by ^2^H-labeling from ^2^H_2_O (*right*). *B*, chronic alcohol effect on hepatic acetylcarnitine, free carnitine levels, and the acetylcarnitine/carnitine ratio (*left*), as well as acetylcarnitine turnover via ^2^H-labeling from ^2^H_2_O (*middle*). The right figure shows turnover of H3 histone acetylation, based on ^2^H-labeling of acetyl moiety of acetylated peptide 18KQLARKAAR26. *C*, western blot analysis of CrAT, Acly, and Acss2 in PF and EF mouse livers. *D*, NAD^+^, NADH levels, and NAD^+^/NADH ratio in PF and EF mouse livers. *E*, western blot analysis of NAD^+^-dependent sirtuins: Sirt1, Sirt2, Sirt3, and Sirt6. *F*, Sirt3 activity measured in mice livers. *G*, schematic depicting acetylation regulation in different cell compartments. *A*–*F*, error bars represent SD n = 6. Data were analyzed by two sample *t* tests (∗*p* < 0.05, ∗∗*p* < 0.005, ∗∗∗*p* < 0.001). Acly, ATP citrate lyase; Acss2, acyl-CoA synthase short-chain family member 2; CrAT, carnitine acetyl transferase; EF, ethanol-fed; KAT, lysine acetyl transferase; PF, pair-fed.

Next, we explored the origins of diverse acetylation patterns in specific cell compartments during chronic alcohol exposure. Alcohol may affect acetylation by altering acetyl-CoA and NAD^+^ levels, cofactors in acetylation and sirtuin-dependent deacetylation (Fig. 7*A*). Mitochondrial acetyl-CoA in addition to being a common intermediate in fuel metabolism pathways, is in CrAT-catalyzed equilibrium with acetyl-carnitine, and a precursor for acetylation. Acetyl-CoA levels influences both nonenzymatic acetylation through mass action and enzymatic acetylation via lysine acetyl transferases (KATs). The ratio of acetyl-CoA to free CoA can affect KAT activity, as free CoA inhibits KATs through competitive binding (6). Despite alcohol metabolism contributing to acetyl-CoA production (Fig. 6, *C* and *D*), we observed a nonsignificant decrease in total acetyl-CoA levels in the EF group (*p = 0.071*), alongside a significant increase in free CoA and a marked decrease in the acetyl-CoA/CoA ratio (Fig. 7*A*). The decreasing trend in total acetyl-CoA in alcohol-fed mice likely stems from decreased cytosolic levels, as the cytosol has a larger volume and higher acetyl-CoA concentrations than other compartments, including mitochondria (75, 76). Alcohol also increased acetyl-CoA turnover nearly fivefold (Fig. 7*A*), suggesting enhanced utilization in various pathways and a potential reduction in the overall acetyl-CoA pool.

Mitochondrial acetyl-CoA is transferred to the cytosol via citrate and acetylcarnitine shuttles during excess nutrient availability (14, 19), with Acss2 also contributing to cytosolic acetyl-CoA from free acetate. Alcohol significantly reduced the expression of Acly and Acss2, key enzymes in cytosolic acetyl-CoA production. Recent evidences suggest that distinct acetyl-CoA pools exist in the nucleus and cytosol (76), with complementary acetylcarnitine shuttling linking mitochondrial acetyl-CoA to histone acetylation (19, 77). To evaluate this, we quantified acetylcarnitine, free carnitine levels, the acetylcarnitine/carnitine ratio, and acetylcarnitine turnover over 72 h. To test the role of acetyl-carnitine in acetylation, we also measured ^2^H-labeling of the acetylated histone H3 peptide KQLATKAAR. Given the slow turnover of histones (37) (supplemental Fig. S8), rapid ^2^H-labeling of an acetylated peptide within 72 h reflects the acetyl group rather than the peptide backbone.

Alcohol did not affect acetylcarnitine levels but significantly increased free carnitine and decreased the acetylcarnitine/carnitine ratio. In the EF group, there was a tenfold increase in acetylcarnitine turnover, with rapid acetylcarnitine ^2^H-labeling (Fig. 7*B*, middle). While acetylcarnitine labeling plateaued at around 8.3% after 8 h of ^2^H_2_O exposure in the EF group, it reached a similar enrichment only after 72 h in the PF group. The ^2^H-labeling profile of acetylcarnitine mirrored that of the acetylated H3 peptide ^Ac^KQLAT^Ac^KAAR, though ^2^H-acetyl labeling was lower in both EF (4%) and PF (6%) groups compared to acetylcarnitine (7.5–8.3%) and showed delayed plateau levels (Fig. 7*B*, right). Based on the precursor/product isotope enrichment relationship in tracer studies (78), these results suggest that alcohol-derived mitochondrial acetyl-CoA, shuttled via acetylcarnitine, contributes to nuclear histone acetylation. Chronic alcohol-induced changes in acetylcarnitine turnover were associated with enhanced CrAT expression (Fig. 7*C*), further supporting CrAT’s role in mediating acetyl-CoA transfer from mitochondria to the nucleus for histone acetylation.

In addition to acetyl-CoA, protein acetylation is regulated by NAD^+^ availability, a cofactor for NAD^+^-dependent sirtuins (Sirt1-7), class III HDACs. Sirt3, Sirt4, and Sirt5 are mitochondrial—Sirt3 having the highest affinity for acetylated proteins (79). Sirt2 is localized in the cytosol and plays a significant role in cytosolic protein deacetylation. Sirt1 and Sirt6 are in the cytosol and nucleus, participating in site-specific deacetylation of some histones and cytosolic proteins. Alcohol significantly reduced liver NAD^+^ levels and the NAD^+^/NADH ratio without altering NADH levels (Fig. 7*D*), and decreased expressions of Sirt1, Sirt2, and Sirt6 (Fig. 7*E*). Although Sirt3 expression remained unchanged, its activity decreased (Fig. 7*F*). These results indicate that alcohol-induced mitochondrial acetyl-CoA production, coupled with reduced Sirt3 activity, increases mitochondrial acetylation (Fig. 7*G*). Although we could not directly measure compartmentalized acetyl-CoA pools, the upregulation of CrAT and downregulation of Acly and Acss2 likely led to distinct regulation of nuclear and cytosolic acetyl-CoA pools. The reduced cytosolic acetyl-CoA production by Acly and Acss2, along with free CoA inhibiting cytosolic KATs and decreased expression of NAD^+^-dependent Sirt1 and Sirt2, may explain the relatively unchanged acetylation of cytosolic proteins. Additionally, impaired Sirt1-and Sirt6-dependent deacetylation, combined with enhanced CrAT-mediated transport of alcohol-derived acetyl-CoA from mitochondria to the nucleus, may have contributed to increased histone acetylation. (Fig. 7*G*).

### Metabolic Consequences of Alcohol-Related Altered Proteostasis

**Fig. 8:**
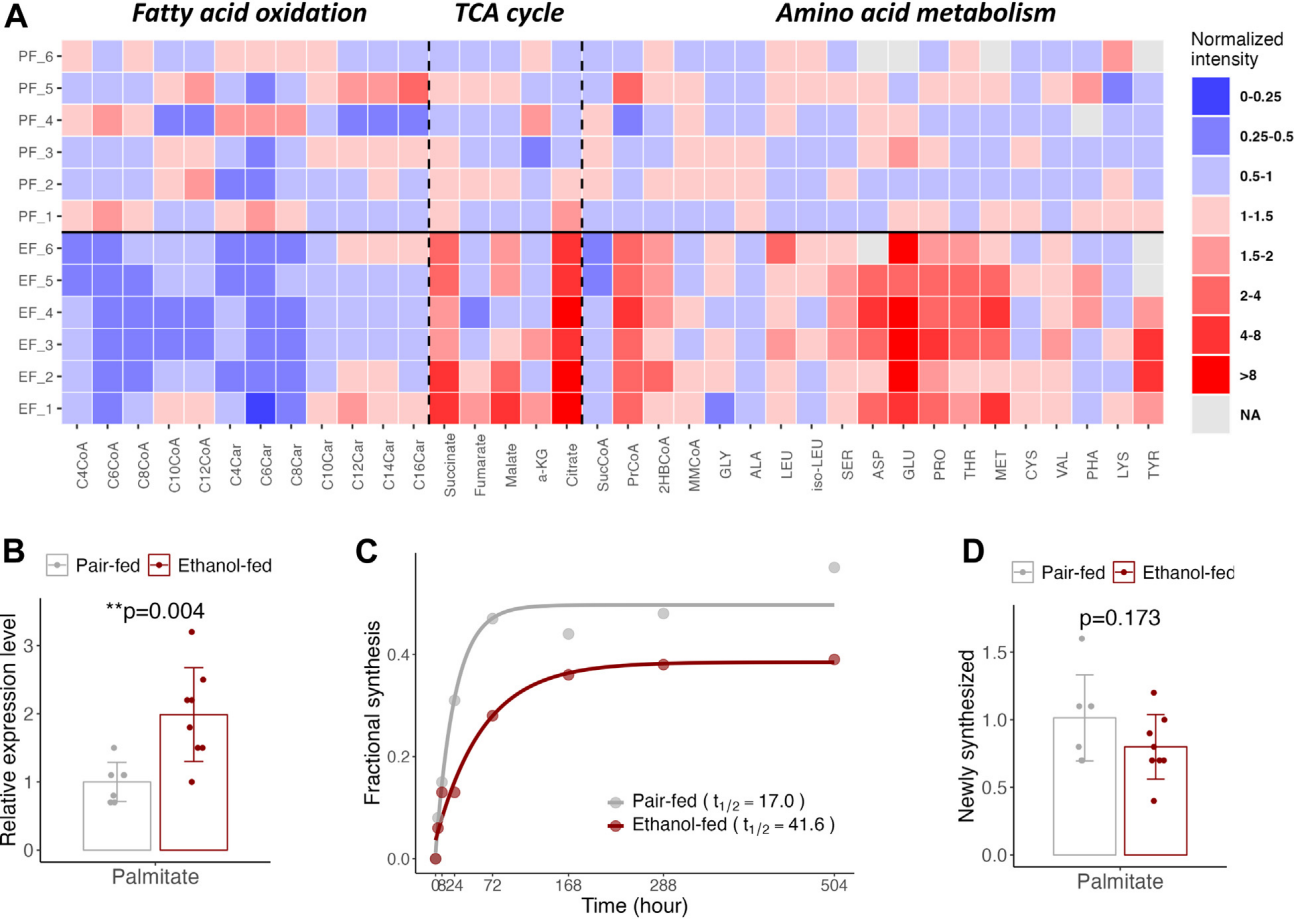
**Metabolic consequences of alcohol-induced disruption of hepatic proteostasis.***A*, heatmap displaying the normalized expression values of metabolites involved in fatty acid oxidation, the TCA cycle, and amino acid metabolism. Normalization is performed such that the mean value in control (PF) mice is set to one. *B*, the effect of ethanol consumption on total hepatic palmitate levels. *C*, palmitate turnover quantified using a ^2^H_2_O–metabolic labeling approach. *D*, fraction of newly synthesized palmitate calculated based on palmitate levels and ^2^H labeling. PF, pair-fed; TCA, tricarboxylic acid.

Chronic alcohol consumption primarily affected the acetylation and turnover of proteins involved in metabolic processes, prompting us to use targeted metabolomics to quantify relevant metabolites. Alcohol-induced alterations in long-chain acyl-CoA ligases (ACSL1 and ACSL5), essential enzymes in mitochondrial fatty acid oxidation were associated with reduced short- and medium-chain acyl-CoAs and acyl-carnitines, suggesting impaired fatty acid β-oxidation (Fig. 8*A*, supplemental Fig. S12*A*). This finding corroborates previous reports that chronic alcohol intake reduces mitochondrial fatty acid oxidation (80). Chronic alcohol consumption also altered other mitochondrial processes, elevating TCA cycle intermediates like succinate, citrate, and malate in the EF mouse livers, although malate narrowly missed significance (Fig. 8*A*, supplemental Fig. S12*B*). However, succinyl-CoA was reduced (Fig. 8*A*, supplemental Fig. S12*C*), suggesting alcohol may inhibit TCA flux due to a mitochondrial redox shift. Additionally, alcohol caused a twofold increase in 2-hydroxybutyryl-CoA (2HBCoA) and a threefold increase in propionyl-CoA (supplemental Fig. S12*C*), intermediates in methionine and branched-chain amino acid metabolism. The 2-hydroxybutyryl-CoA increase was associated with higher 2-hydroxybutyrate (2HB) levels (supplemental Fig. S12*D*), a marker of mitochondrial disorders and hepatic reductive stress (81). This suggests the alcohol-induced rise in the NADH/NAD^+^ ratio shifted 2-ketobutyrate (2KB) metabolism from the TCA cycle to 2HB, a byproduct of threonine and methionine catabolism.

To further assess alcohol's impact on protein and amino acid metabolism, we profiled hepatic amino acids. Alanine decreased in the EF group, likely due to the alcohol-induced redox shift diverting pyruvate to lactate. However, alcohol significantly increased several amino acids, including leucine, serine, aspartate, proline, threonine, methionine, tyrosine, and glutamate, with glutamate rising over fivefold (Fig. 8*A*, supplemental Fig. S12*C*), suggesting restricted proteolysis and inhibited urea synthesis. Glycine and cysteine, key precursors for glutathione, showed no changes, suggesting they may be continuously utilized in glutathione synthesis to combat alcohol-induced oxidative stress. In addition, increased 2HB and 2HB-CoA production (supplemental Fig. S12, *C* and *D*), linked to cysteine synthesis, may support this process. These findings align with restricted acetylation-related increased turnover of cytosolic proteins in one-carbon metabolism, upstream of glutathione biosynthesis. In the cytosol, alcohol also increased palmitate levels (Fig. 8*B*) and reduced its turnover (Fig. 8*C*) without affecting glucose-driven newly synthesized palmitate (Fig. 8*D*). Interestingly, alcohol consumption was linked to a reduction in the plateau labeling of palmitate (Fig. 8*C*), suggesting that ethanol may dilute the glycolytic ^2^H-labeled acetyl-CoA by producing "cold" acetyl-CoA precursors for palmitate synthesis. These findings, along with evidence of acetyl-CoA derived from deuterated ethanol (ethanol-d6) being incorporated into palmitate (Fig. 6*C*), indicate that alcohol contributes to hepatic fat accumulation by serving as a carbon source for fatty acid synthesis.

## Discussion

This study reveals that chronic alcohol consumption disrupts hepatic proteostasis and metabolism in a mouse model of liver injury. Through biochemical and metabolic profiling, combined with ^2^H_2_O–based proteome and acetylome analysis, we identified alcohol-induced proteinopathy linked to organelle-specific alterations in hepatic acetylation. Alcohol enhanced acetylation of histones and mitochondrial enzymes while restricting cytosolic acetylation, resembling a fasted state. These metabolic shifts occurred despite consistent caloric intake in the EF group, suggesting alcohol-driven acetylation imbalances contribute to liver injury, including elevated liver weight from triglyceride and protein accumulation. Furthermore, acetylation-mediated reduced UPS activity (36) and impaired autophagy and mitophagy exacerbated hepatic protein accumulation (82).

Although the impact of posttranslational modifications on protein turnover is well established in cell culture (83, 84), measuring the kinetics of modified proteins *in vivo* remains challenging. The ^2^H_2_O–metabolic labeling method has been used to study the turnover rates of thousands of proteins (31, 34, 85, 86). In this study, we applied ^2^H_2_O metabolic labeling combined with immunoaffinity LC-MS/MS to quantify the kinetics of acetylated peptides in live animals (36, 37). Similar to previous studies using stable isotope labeling with amino acids in cell culture (83, 84), our approach with ^2^H_2_O metabolic labeling enables the measurement of turnover rates for both acetylated and native peptide forms. This method reveals site-specific differences in turnover rates attributable to acetylation *in vivo*. The scope of ^2^H_2_O method for protein turnover studies is influenced by several factors, including the duration and level of ^2^H_2_O exposure, the number of time points sampled, total protein input, sample preparation methods (e.g., proteolytic digestion and offline peptide fractionation), and data analysis tools. Turnover analysis of acetylated peptides is further complicated by the typically low stoichiometry of acetylation in non-histone proteins. In this proof-of-concept study, the relatively lower ^2^H_2_O enrichment (6% in drinking water), shorter labeling duration (21 days), limited sample collection (six time points for protein turnover studies), and lower total protein input (30 μg) constrained the number of quantifiable proteins. Despite these limitations, by integrating ^2^H_2_O metabolic labeling with differential proteomics and proteome dynamics, and metabolic profiling, we demonstrate that ethanol-induced alterations in hepatic protein turnover are associated with acetylation and that these altered acetylome dynamics contribute to ethanol-induced metabolic changes.

Reversal acetylation is regulated by the acetyl-CoA-dependent KATs and NAD^+^-dependent sirtuins. Acetyl-CoA is generated within mitochondria via catabolic pathways and produced in the nucleus and cytosol through acetate activation. The inner mitochondrial membrane's impermeability isolates mitochondrial acetyl-CoA from other cell compartments. Distinct acetylation patterns in the mitochondria, cytosol, and nucleus following chronic alcohol consumption suggest alcohol disrupts subcellular metabolism of acetyl-CoA and NAD^+^, key cosubstrates for acetylation and deacetylation. Our results indicate that alcohol-induced shifts in the NADH/NAD^+^ ratio affect redox-dependent metabolic processes, including deacetylation. Additionally, alcohol metabolism increases mitochondrial acetyl-CoA pool, driving enhanced mitochondrial acetylation during chronic alcohol consumption (Fig. 7*G*).

Our study shows distinct acetylation patterns between histones and cytosolic proteins, challenging the conventional view of a shared nuclear-cytosolic acetyl-CoA pool (14). Recently, an alternative pathway linking mitochondrial metabolism to histone acetylation via CrAT-mediated acetyl-CoA shuttling has been proposed (19, 77). While nuclear localized Acly, Acss2, and pyruvate dehydrogenase are known to generate a distinct nuclear acetyl-CoA pool (16), CrAT's role has remained unclear. CrAT maintains equilibrium between mitochondrial acetyl-CoA and acetylcarnitine, exporting excess mitochondrial acetyl-CoA in the form of acetyl-carnitine. Although we could not directly measure subcellular acetyl-CoA pools, our findings shows that chronic alcohol intake suppresses Acly and Acss2 while stimulating CrAT to buffer mitochondrial acetyl-CoA to acetylcarnitine (Fig. 7*G*). These results, combined with the synchronous turnover of acetylcarnitine and histone acetylation (Fig. 7*B*) suggest that acetylcarnitine shuttles mitochondrial acetyl-CoA directly for histone acetylation, bypassing and limiting cytosolic acetylation. Additionally, local differences in NAD^+^ metabolism may further influence sirtuin-dependent deacetylation differently in the cytosol and nucleus (7).

Our results also provide insights into alcohol-mediated metabolic reprogramming arising from compartmentalized acetyl-CoA metabolism and acetylation. Acetyl-CoA, a nexus of catabolic and anabolic pathways, is a signaling molecule that allows cells to adapt to stress via acetylation-dependent transcriptional and posttranslational changes. The reversible acetylation of metabolic enzymes coordinates fuel utilization and metabolic flux (8) through enzymatic control (87). Acetylation also may regulate metabolism through altered stability of metabolic enzymes and transcription factors. Although some instances of acetylation activating transcription factors and enzymes exist, hyperacetylation generally inhibits metabolic enzymes, particularly impacting mitochondrial function and substrate metabolism (13, 88). In line with previous reports (89), we found that subunits of oxidative phosphorylation involved in NADH/FADH_2_ oxidation and ATP synthesis, as well as mitochondrial NAD^+^-dependent dehydrogenases in fatty acid oxidation and the TCA cycle, were acetylated in the livers of EF mice. Our metabolic profiling indicates that alcohol-induced enhanced acetylation of mitochondrial proteins was associated with the inhibition of fatty acid oxidation, the TCA cycle, and nitrogen disposal. Thus, increased mitochondrial acetylation may disrupt substrate metabolism, contributing to liver injury.

In contrast, restricted cytosolic acetylation due to alcohol was correlated with increased hepatic triglyceride accumulation, a known consequence of alcohol consumption. Alcohol suppressed lipogenic enzymes Acly and Acss2 and did not impact the newly synthesized ^2^H-labeled palmitate fraction (Fig. 8*C*) despite increased total palmitate (Fig. 8*B*) and triglyceride levels in the liver (Fig. 1, *C* and *D*). This suggests fatty acids influx from the diet or adipose tissue contributed to steatosis in the alcohol-fed mouse liver, consistent with alcohol’s effects on adipose tissue lipolysis (90). However, a reduction in the plateau labeling of palmitate (Fig. 8*C*), combined with evidence of acetyl-CoA derived from deuterated ethanol (ethanol-d6) incorporated into palmitate (Fig. 6*C*), suggest that ethanol also serves as a carbon source for hepatic fatty acid synthesis. Previous studies have reported that histone acetylation and lipogenesis compete for the same pools of acetyl-CoA (91), positioning acetyl-CoA as a critical junction between epigenetic regulation and metabolic processes. Our results indicate a similar competition between cytosolic acetylation and lipogenesis. The diversion of ethanol-derived acetate for lipogenesis, in conjunction with histone acetylation, likely further limits cytosolic protein acetylation, affecting proteins involved in the methionine cycle and glutathione metabolism. While further investigation is needed to fully understand the ethanol-induced changes in these pathways, the observed increase in 2HB-CoA and 2HB production—byproducts associated with cysteine synthesis from methionine in transsulfuration pathway—may suggest the activation of this metabolic pathway (92). Additionally, the stable levels of glycine and cysteine, despite elevated hepatic amino acids resulting from impaired nitrogen disposal in urea cycle, likely reflect increased utilization of glycine and cysteine for glutathione synthesis. Consequently, while acetylation of mitochondrial enzymes hindered mitochondrial substrate metabolism and the urea cycle, restricted acetylation of cytosolic enzymes involved in glutathione synthesis and metabolism may have helped mitigate alcohol-induced oxidative stress.

Despite restricted cytosolic acetylation, alcohol increased nuclear histone acetylation, suggesting that alcohol-driven substrate utilization for triglyceride synthesis and constrained cytosolic acetylation preserved acetyl-CoA for maintaining histone acetylation. Histone acetylation in the nucleus enhances metabolism by promoting transcription, potentially leading to alcohol-related epigenetic changes (93). Increased acetylation of H3K18 and H3K23 enhance transcriptional activation and chromatin accessibility. Our results with ethanol-d6 tracing confirm that ethanol metabolism provides acetyl-CoA for histone acetylation. The acetylcarnitine shuttling of alcohol-derived mitochondrial acetyl-CoA to the nucleus likely amplifies chromatin remodeling, contributing to alcohol-induced epigenetic modifications.

In conclusion, our study reveals that chronic alcohol consumption disrupts hepatic metabolism and induces proteinopathy by altering the acetylation of histones and non-histone proteins. These findings highlight the intricate relationship between compartmentalized acetylation, protein turnover, and metabolic regulation, enhancing our understanding of alcohol-induced liver injury. Further research is needed to investigate the functional implications of site-specific alcohol-mediated acetylation and its effects on hepatic proteostasis and metabolism.

## Data Availability

All data needed to evaluate the conclusions in the article are present in the paper and the supplementary materials. The mass spectrometry proteomics data have been deposited to the ProteomeXchange Consortium via the PRIDE partner repository with the ProteomeXchange (accession number: PXD055349).

All scripts used for data analysis and plotting are available in the following GitHub repository: https://github.com/tsunghengtsai/ald-turnover. This repository also contains sample code for simulating labeling data and analyzing the simulated results.

Annotated spectra that support the identification of proteins with a single unique peptide, including acetylated peptides, are available in the source data section of this submission.

## Supplemental data

This article contains supplemental data (13, 21, 34, 36, 41, 42, 43, 44, 45, 46, 48, 49, 50, 51, 55, 63, 94, 95, 96, 97, 98).

## Conflict of Interest

The authors declare no competing interests.

## Supplemental data

Quant_Files_From_D2Ome.7z

List_of_raw_files_with_descriptions

Annotated_MSMS_Spectra_Signle_Unique_Peptides

Annotated_MSMS_Spectra_Acetylated_Peptides

Key Resources Table

Supplemental Material

Non-proteomics_Source Data
