# Supplementary material for: Chronic Alcohol Consumption Reprograms Hepatic Metabolism Through Organelle-Specific Acetylation in Mice
Source: Mol Cell Proteomics. 2025 May 12;24(6):100990. doi: 10.1016/j.mcpro.2025.100990 (PMC12289531; doi:10.1016/j.mcpro.2025.100990)

## Slide 1
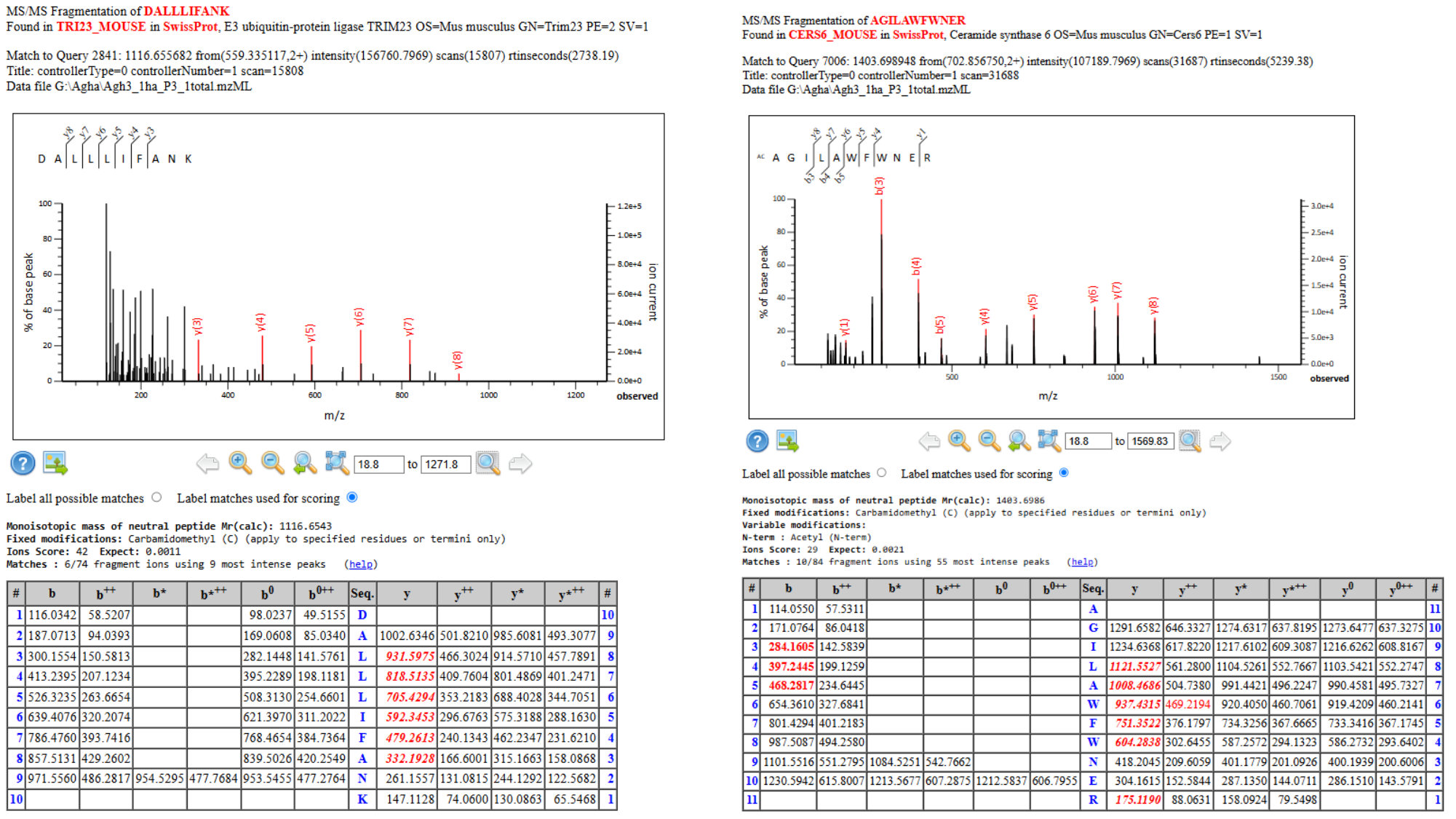

## Slide 2
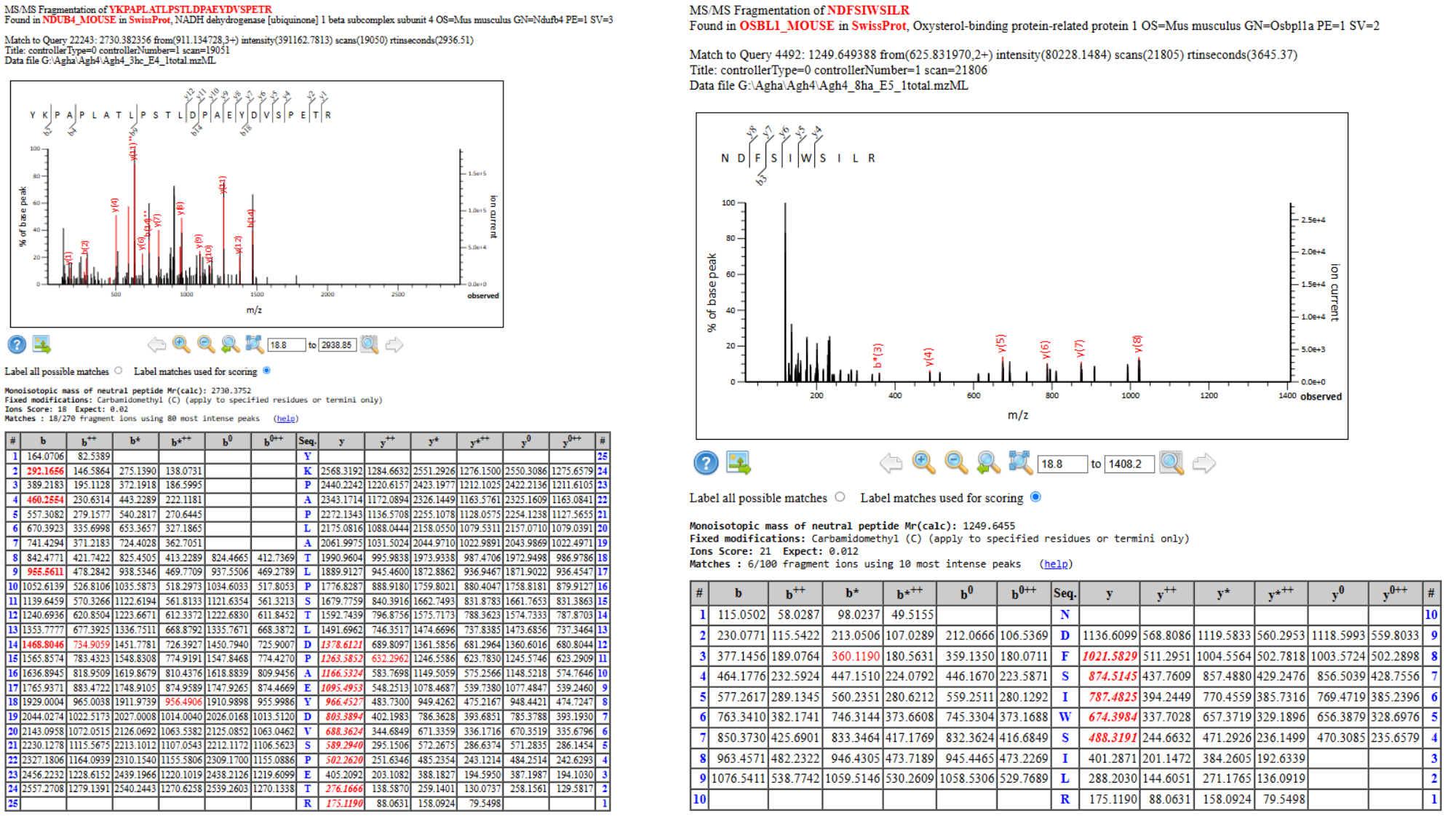

## Slide 3
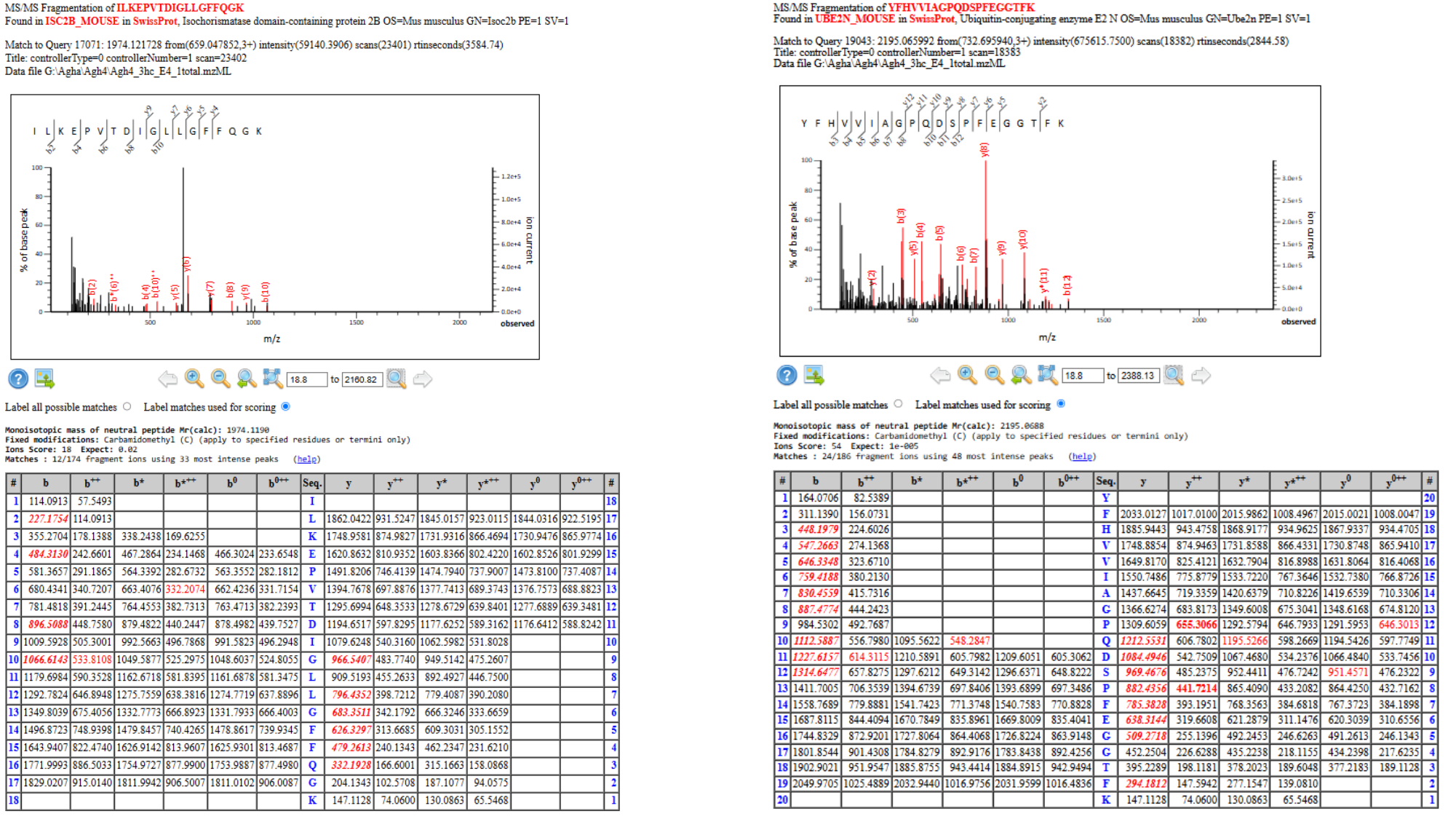

## Slide 4
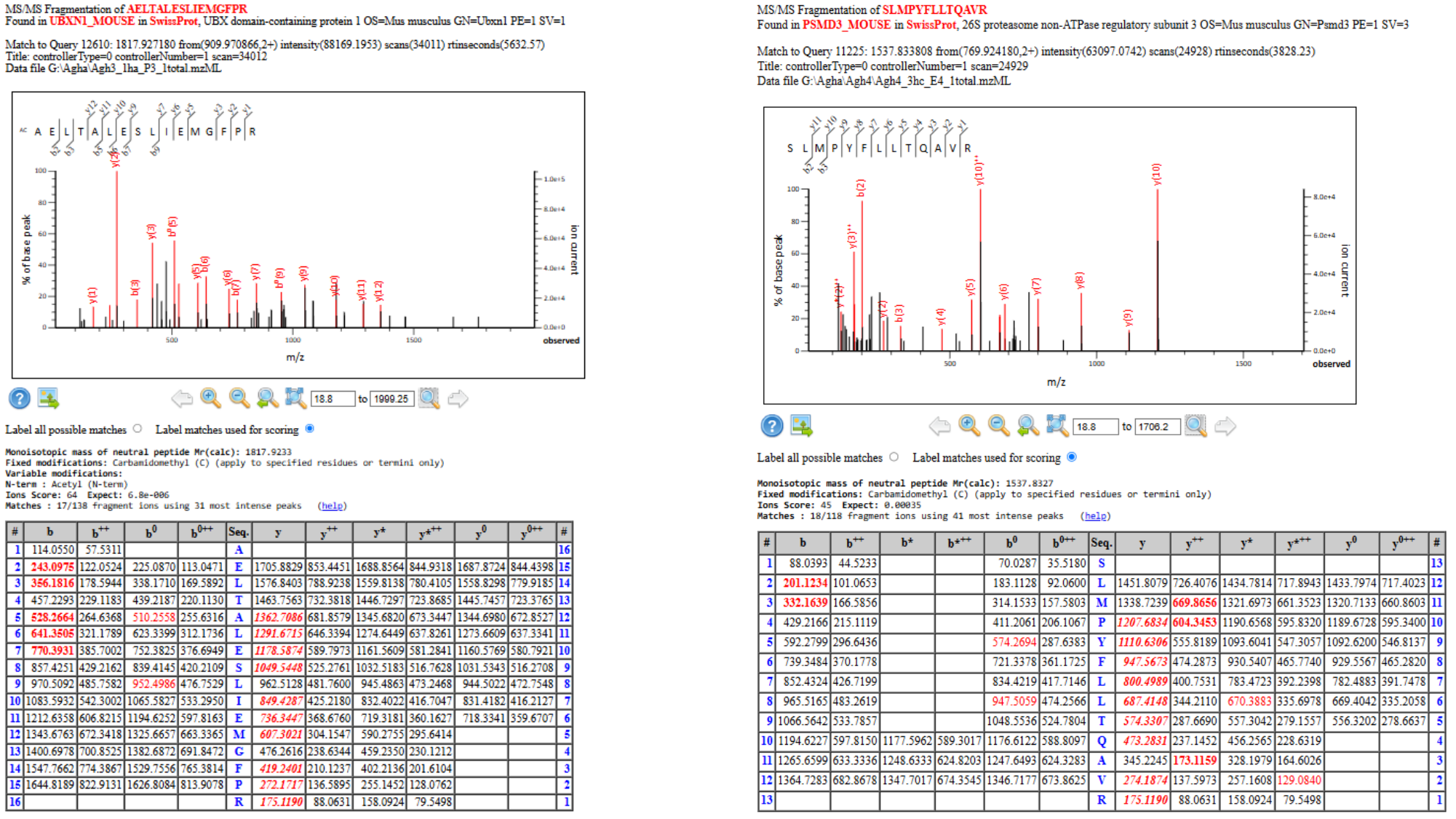

## Slide 5
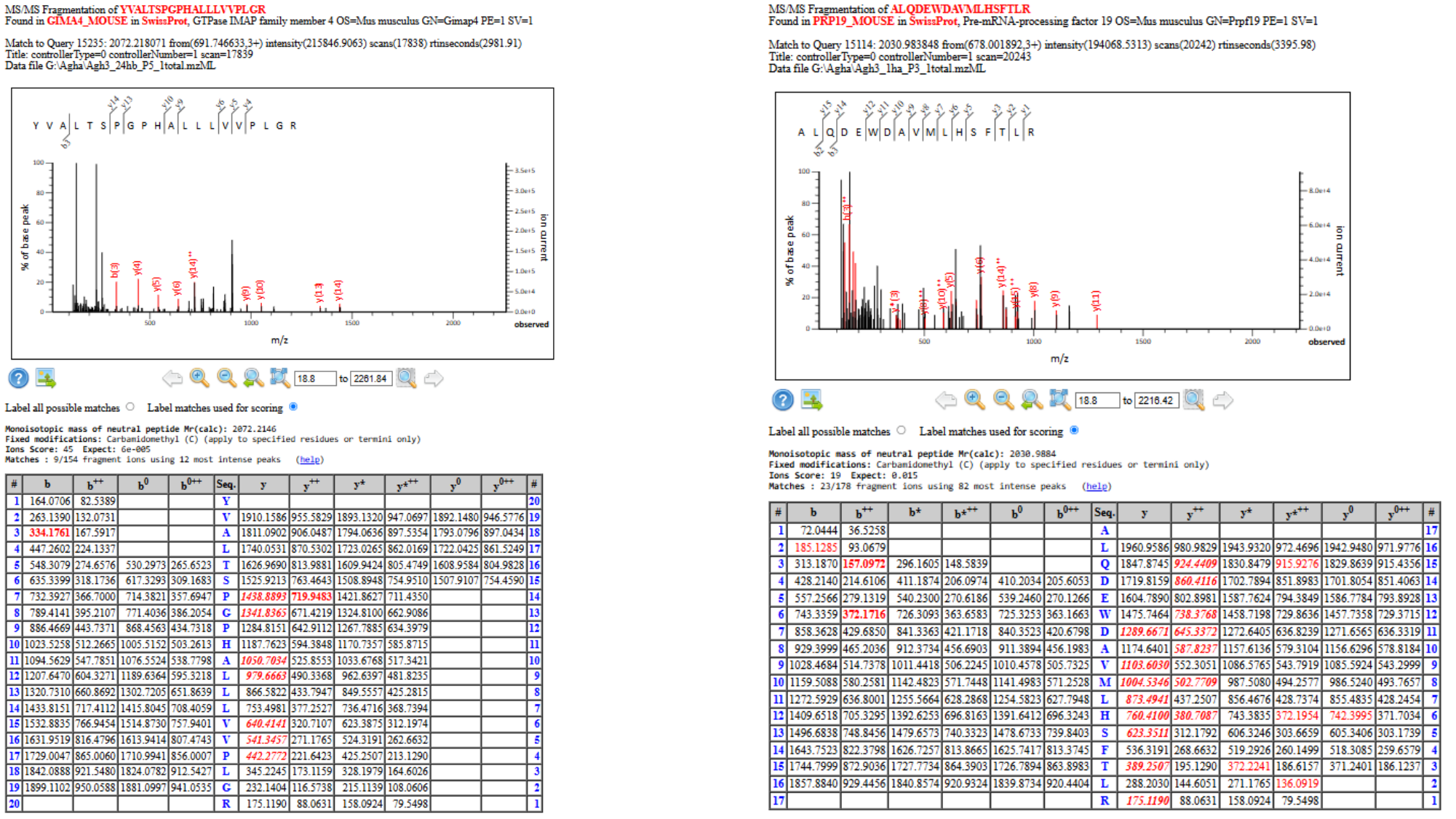

## Slide 6
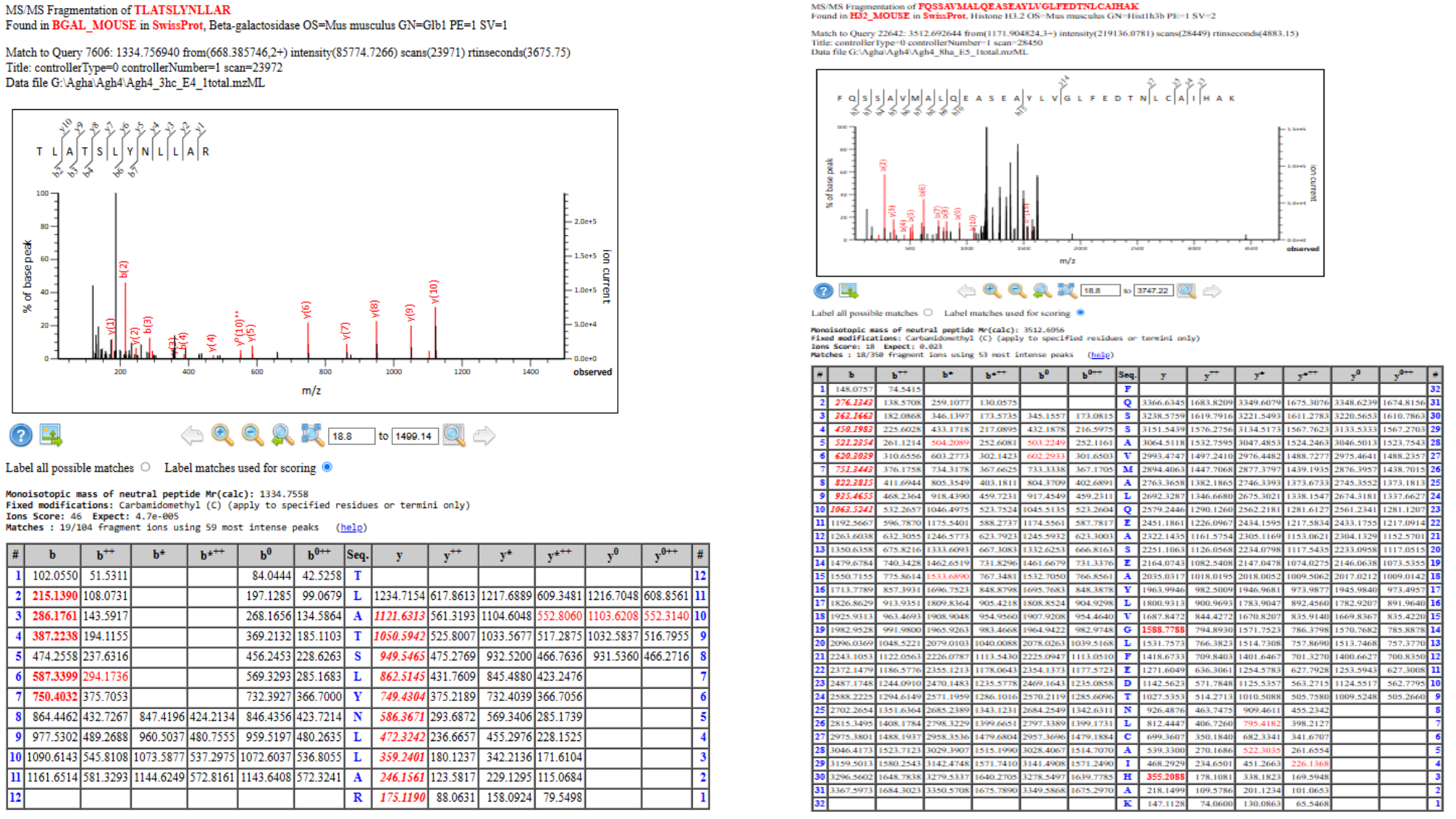

## Slide 7
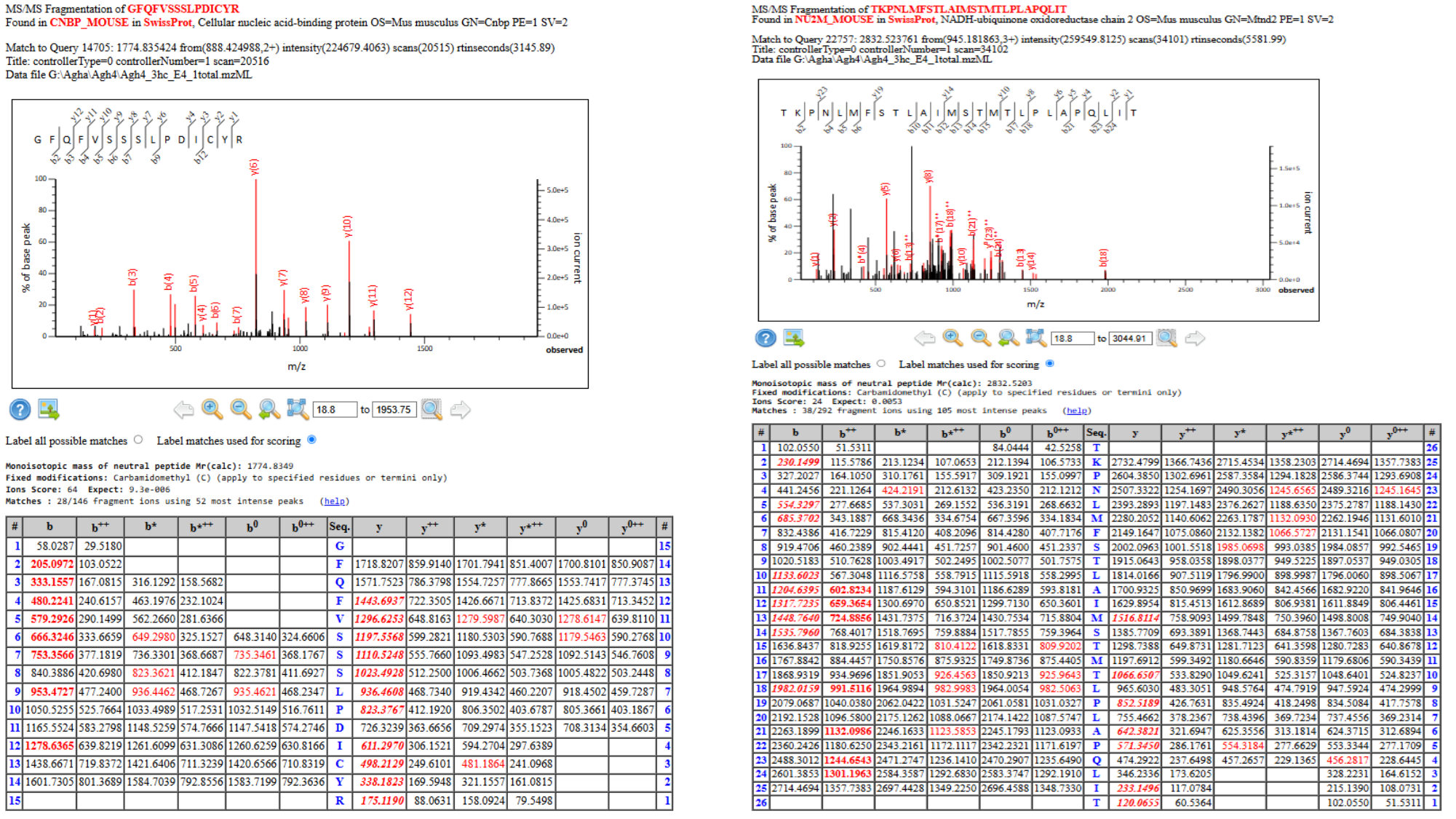

## Slide 8
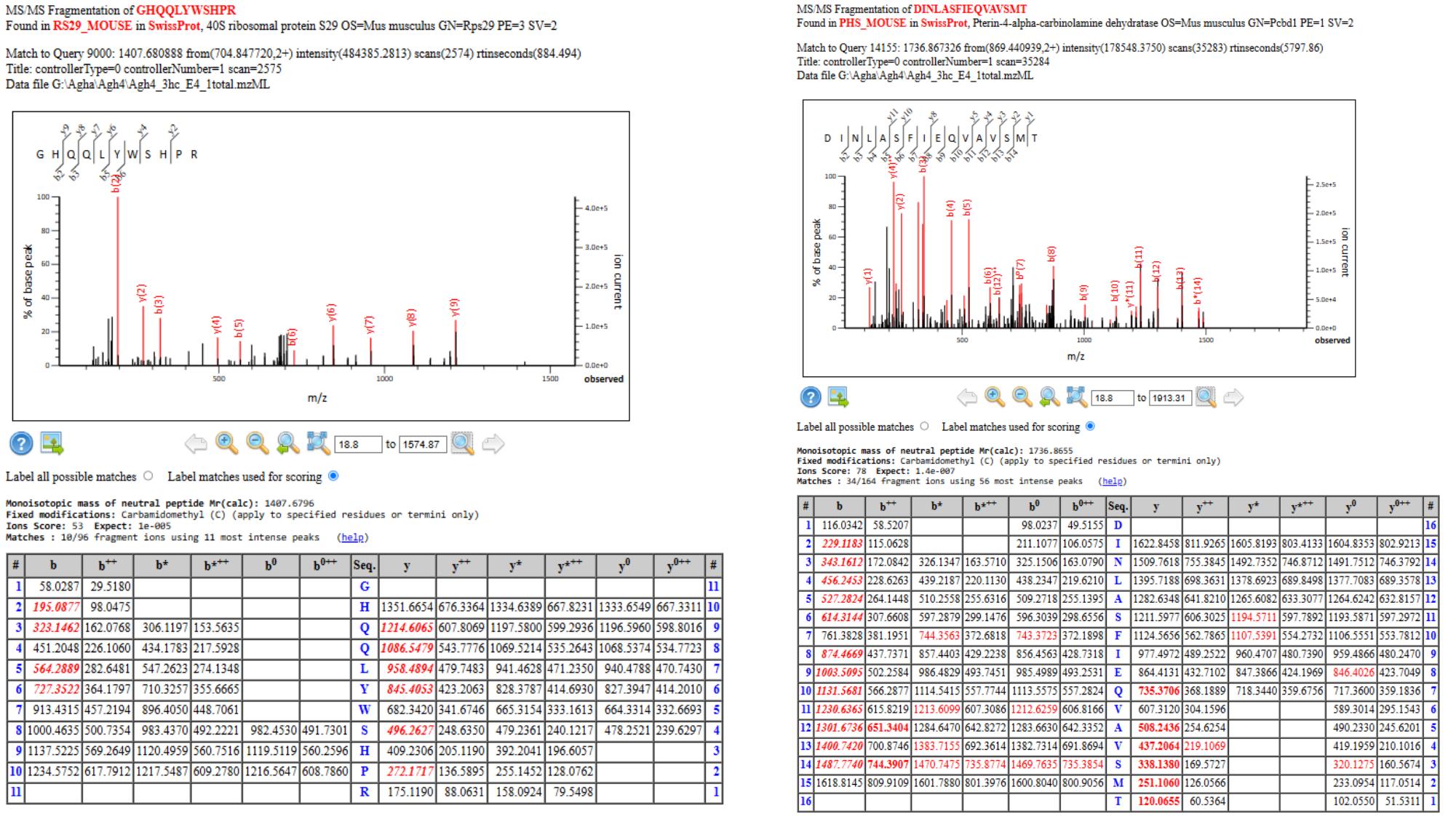

## Slide 9
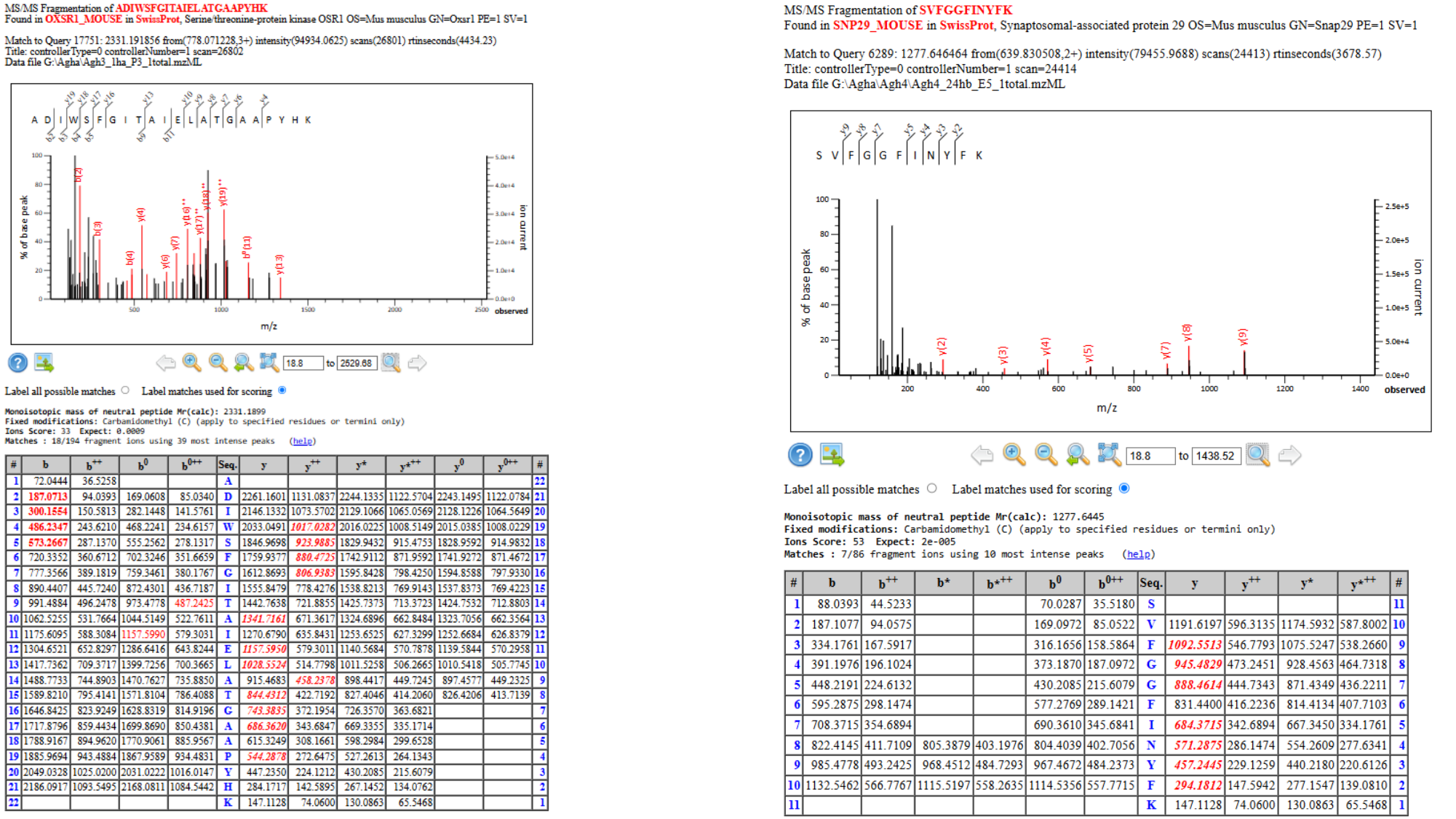

## Slide 10
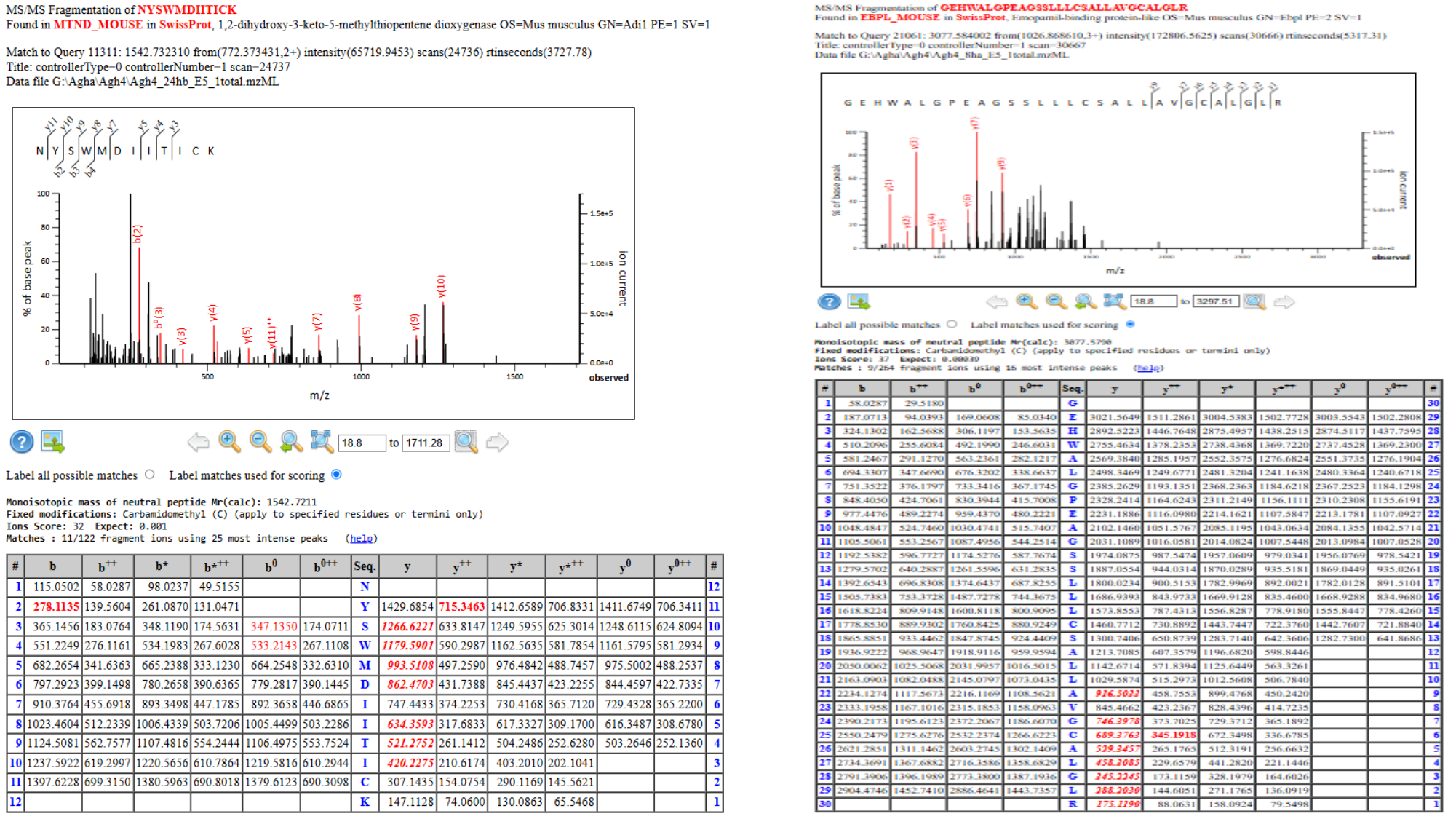

## Slide 11
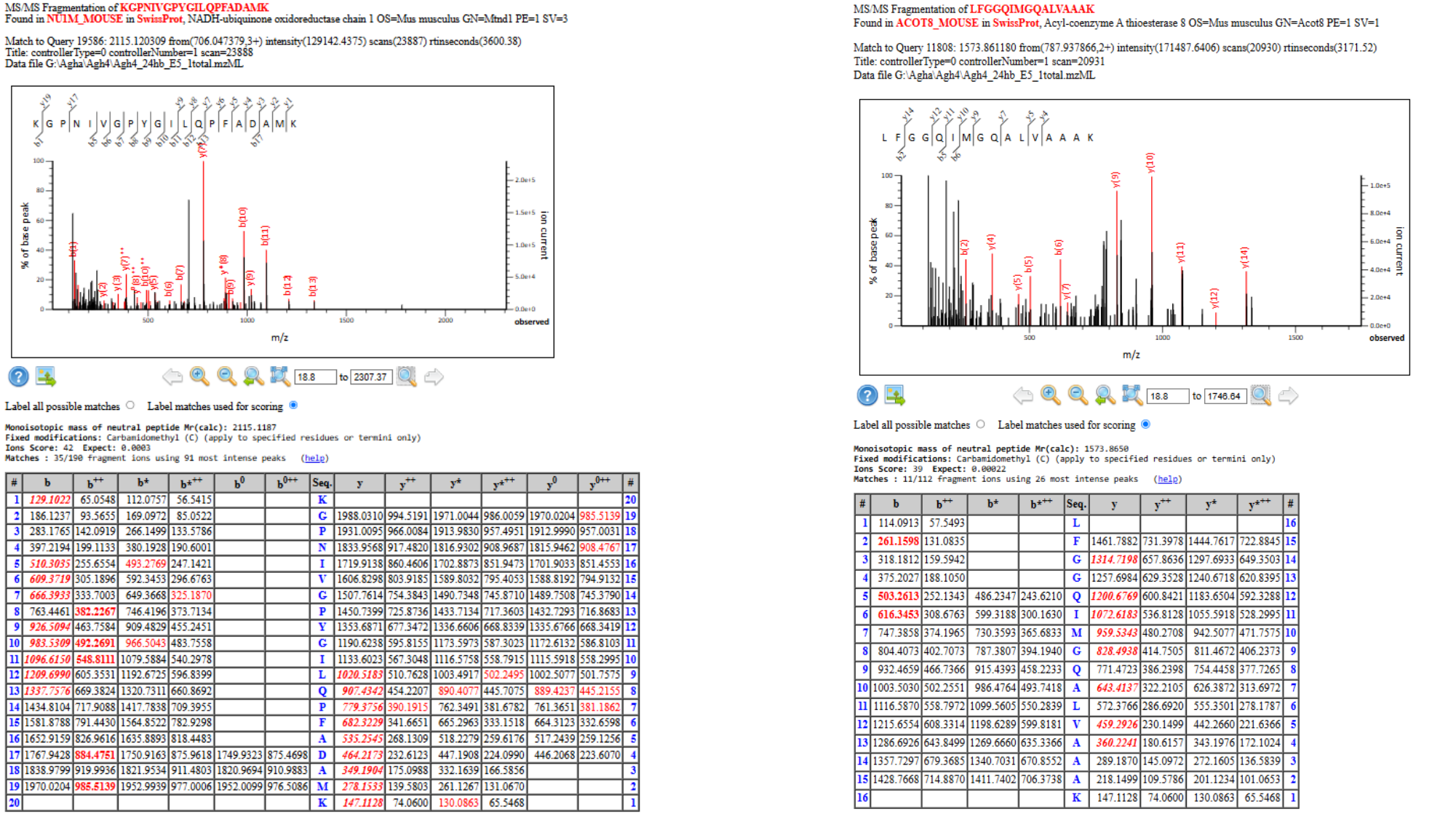

## Slide 12
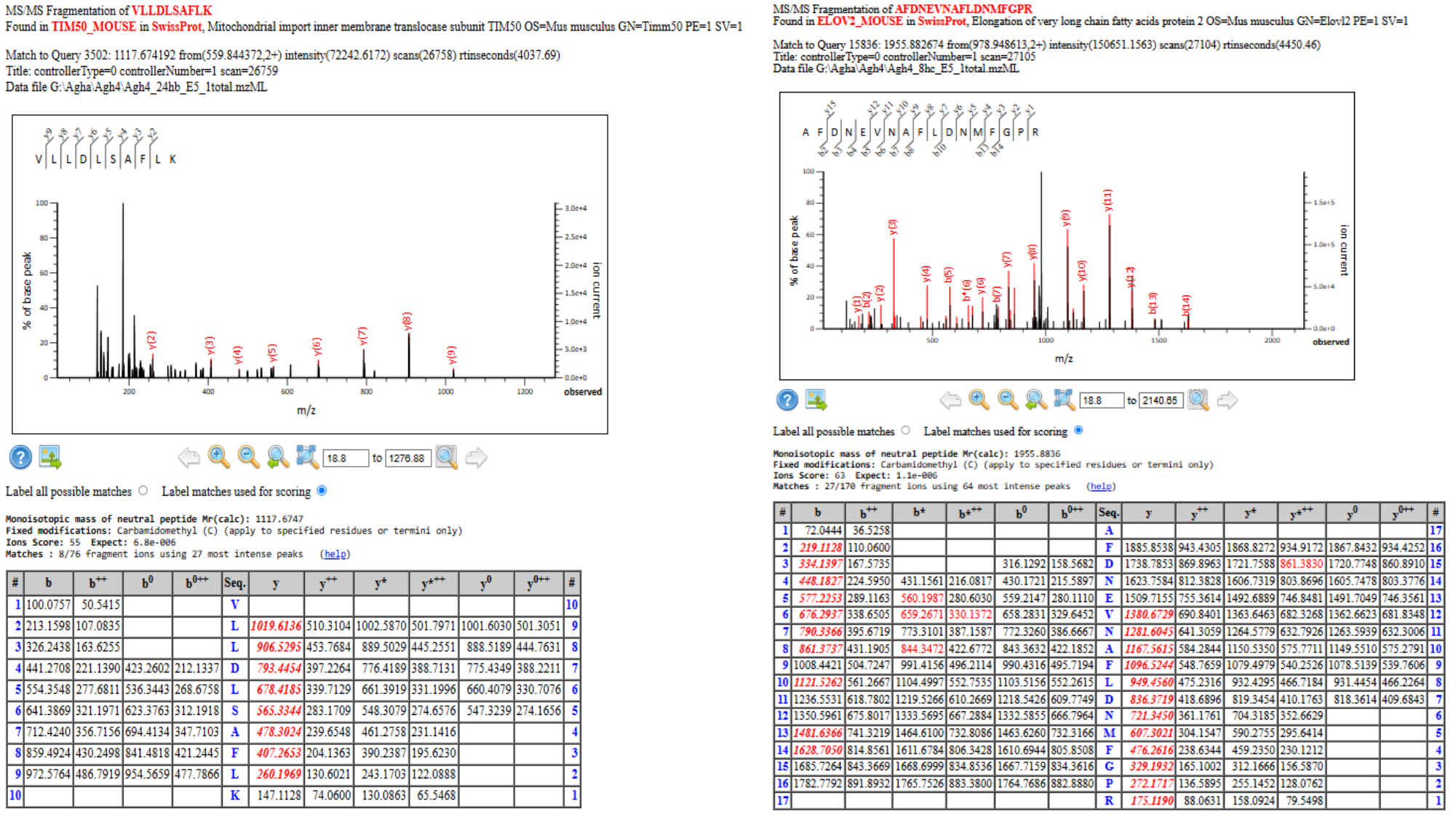

## Slide 13
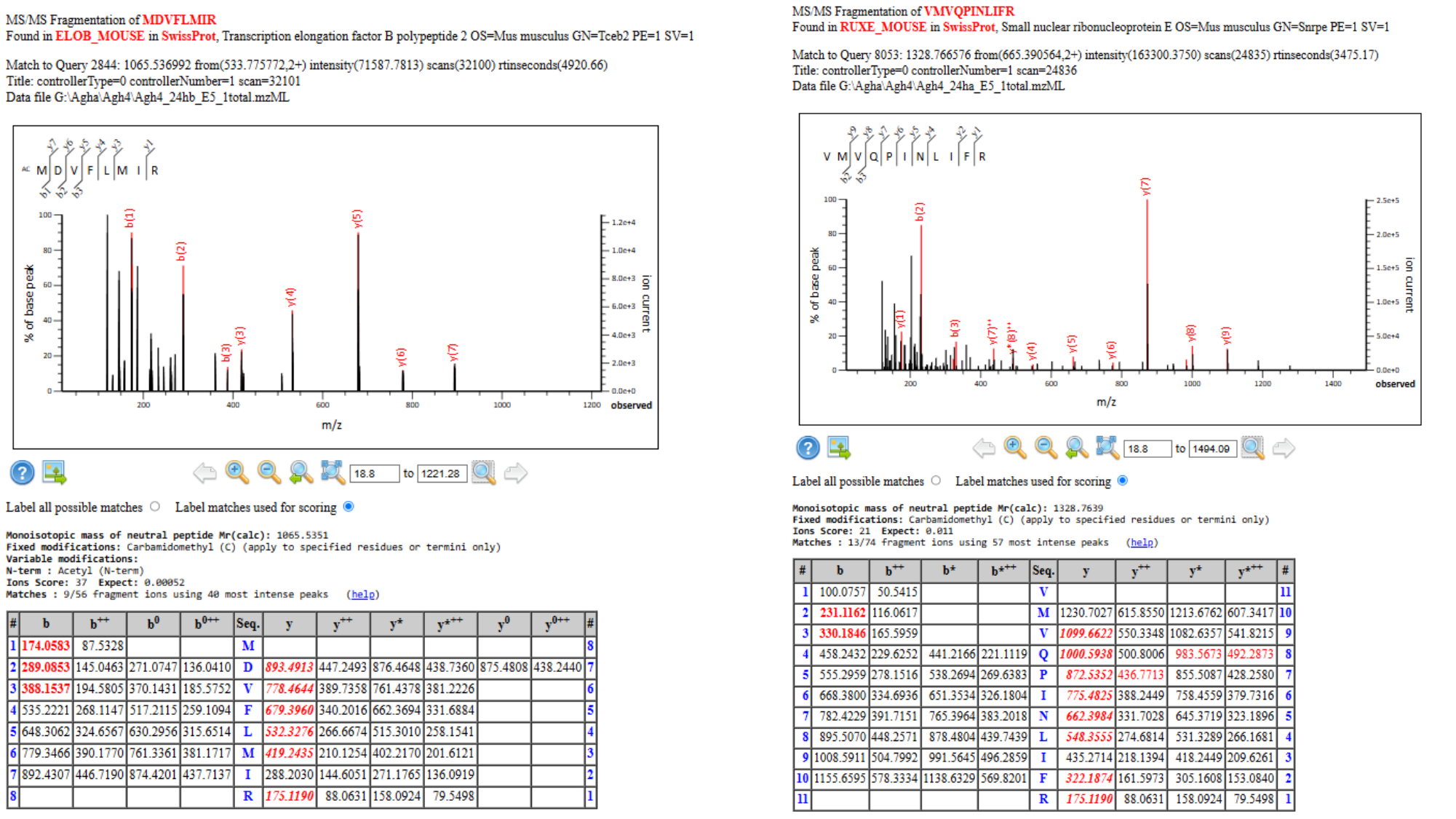

## Slide 14
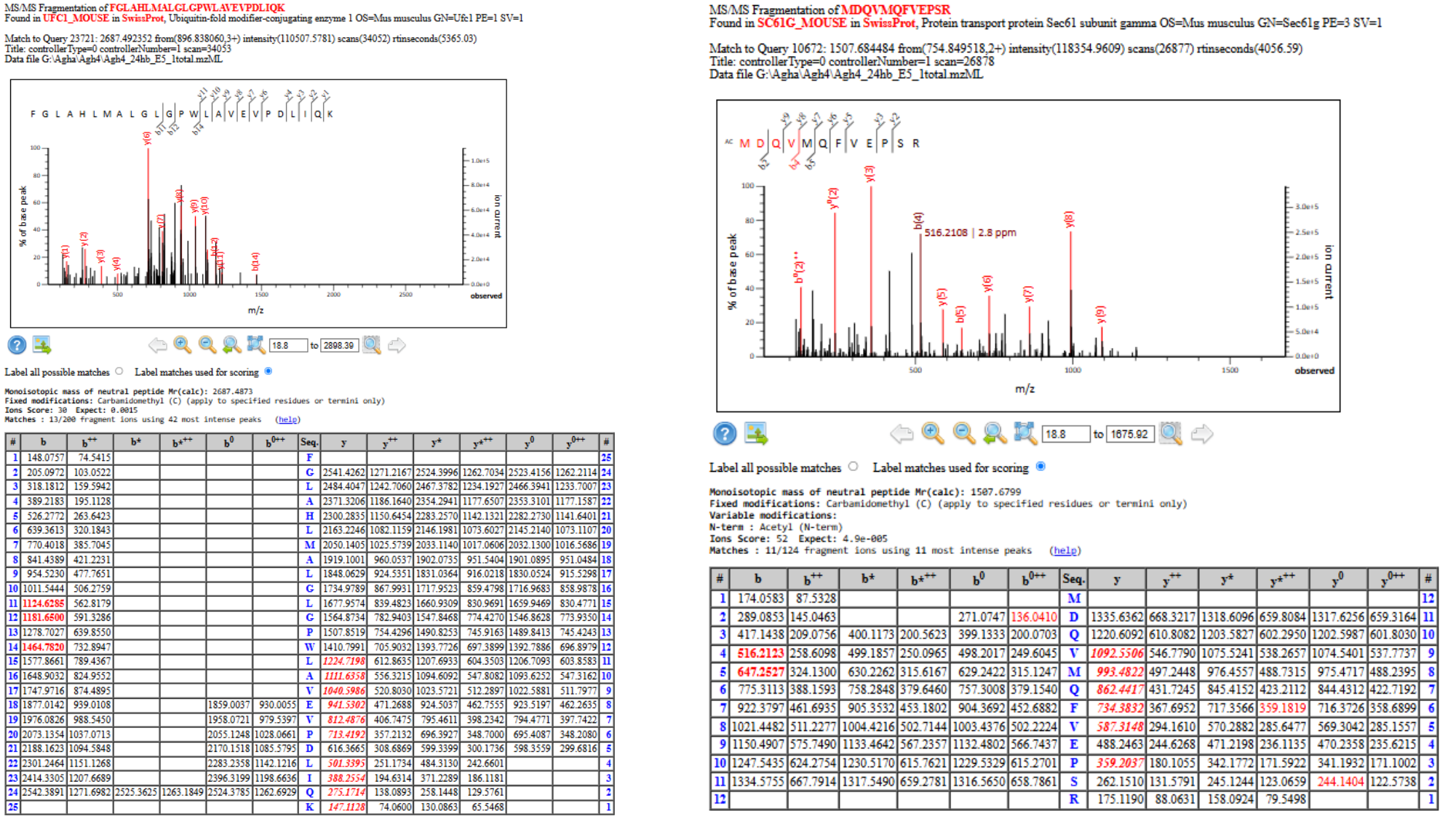

## Slide 15
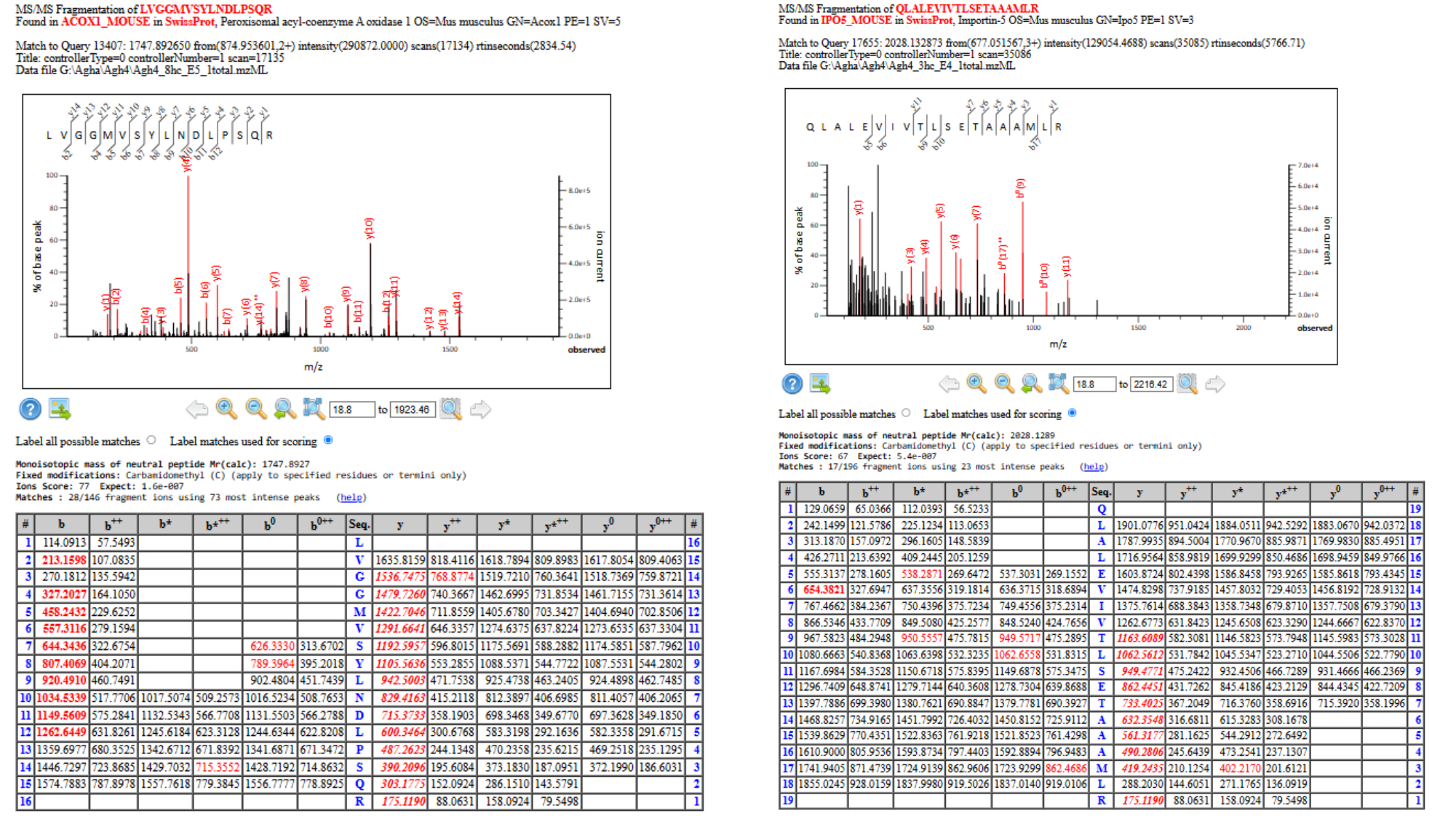

## Slide 16
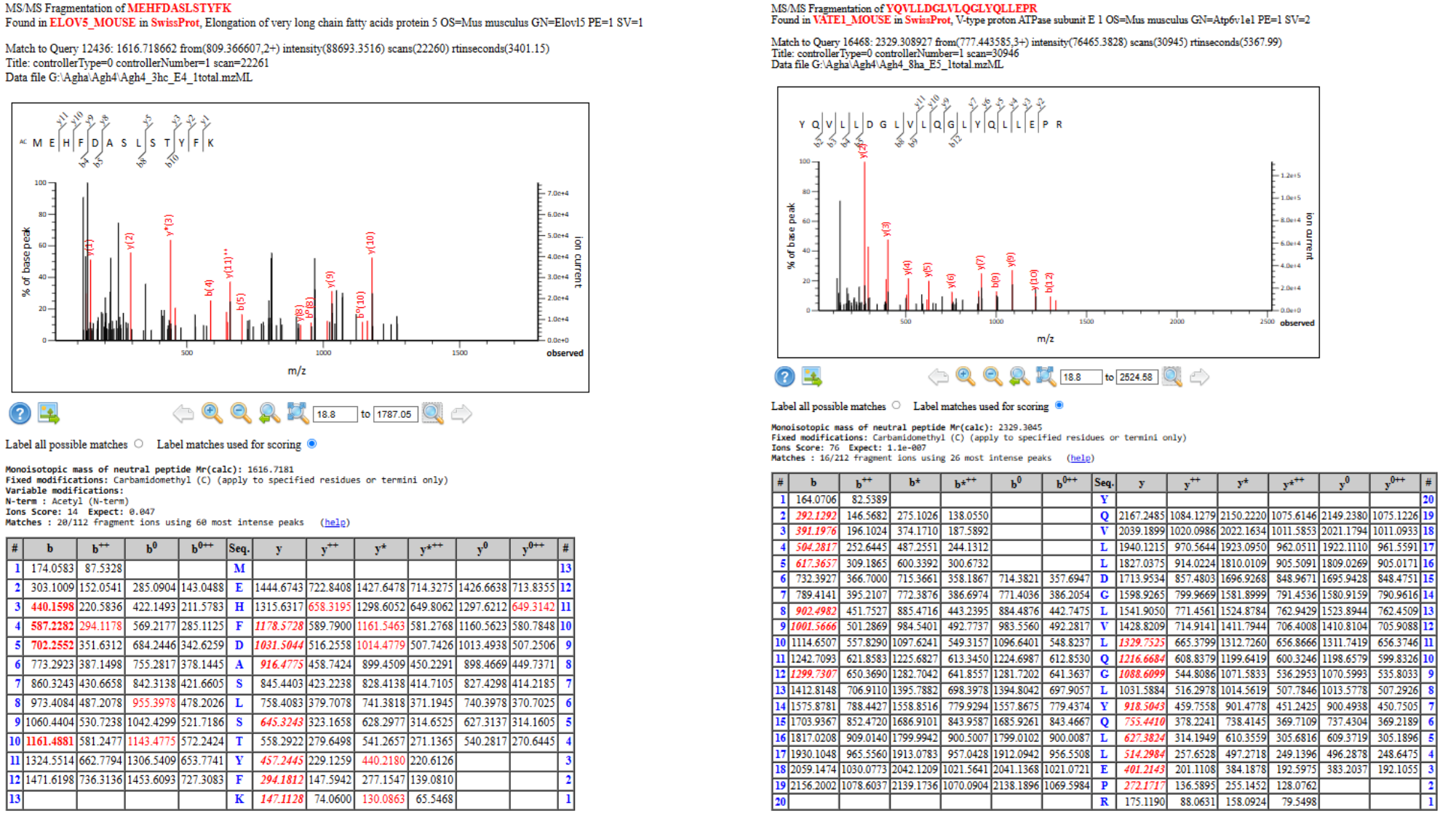

Supplement: Annotated_MSMS_Spectra_Signle_Unique_Peptides [file mmc3.pptx]
