# Supplementary material for: Chronic Alcohol Consumption Reprograms Hepatic Metabolism Through Organelle-Specific Acetylation in Mice
Source: Mol Cell Proteomics. 2025 May 12;24(6):100990. doi: 10.1016/j.mcpro.2025.100990 (PMC12289531; doi:10.1016/j.mcpro.2025.100990)
Supplement: Annotated_MSMS_Spectra_Acetylated_Peptides [file mmc4.pptx]

## Slide 1
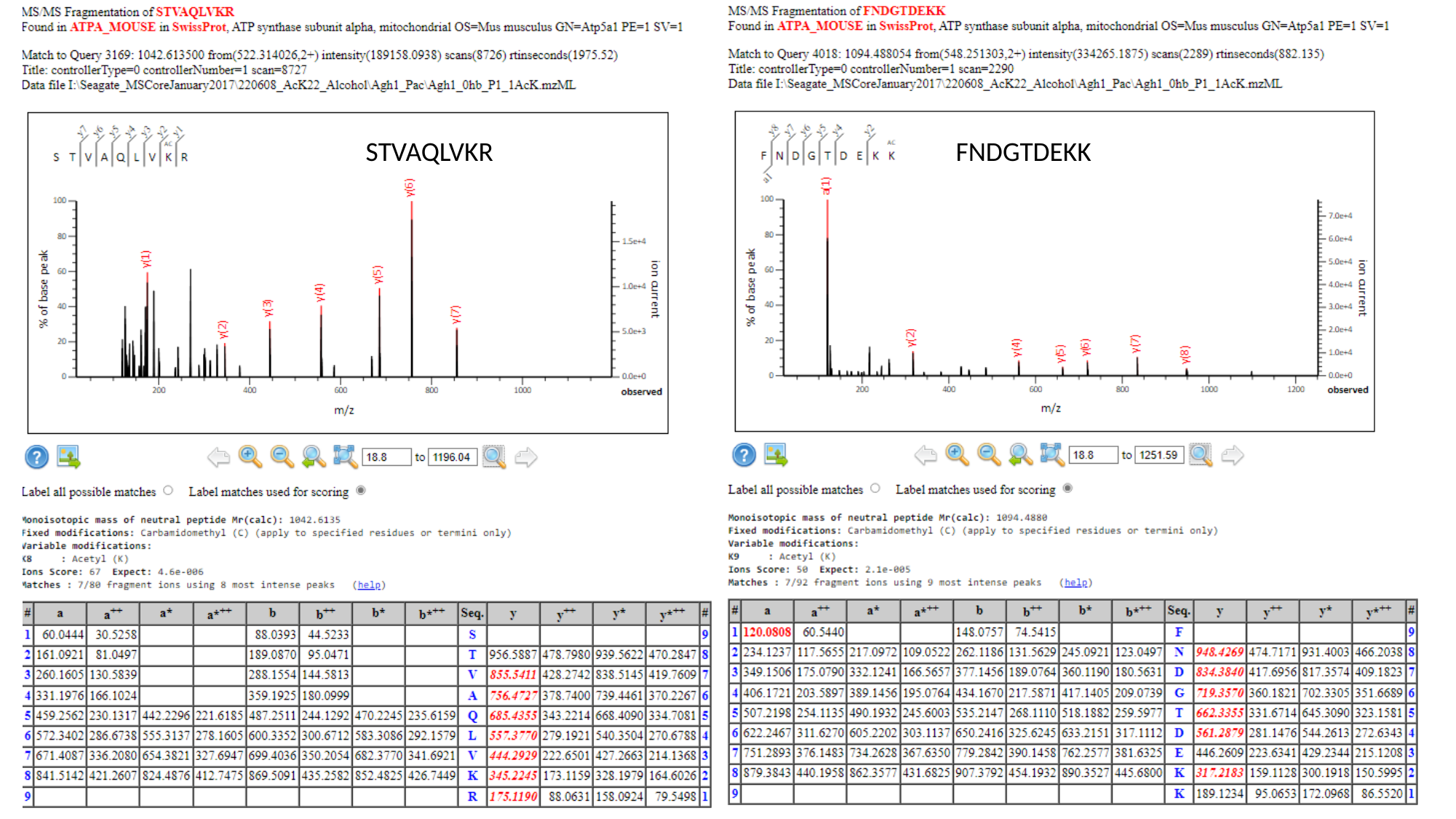

STVAQLVKR FNDGTDEKK

## Slide 2
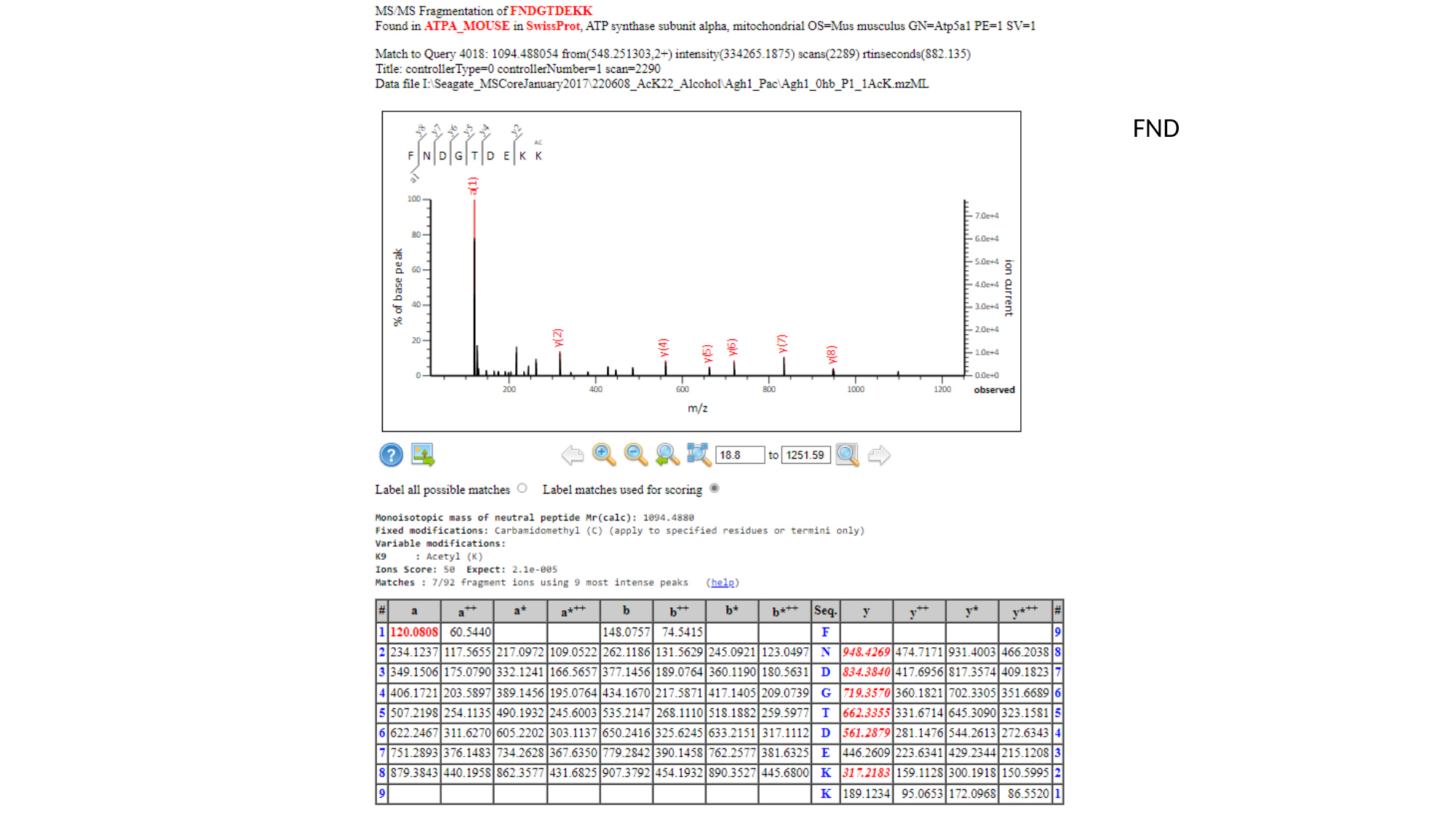

FND

## Slide 3
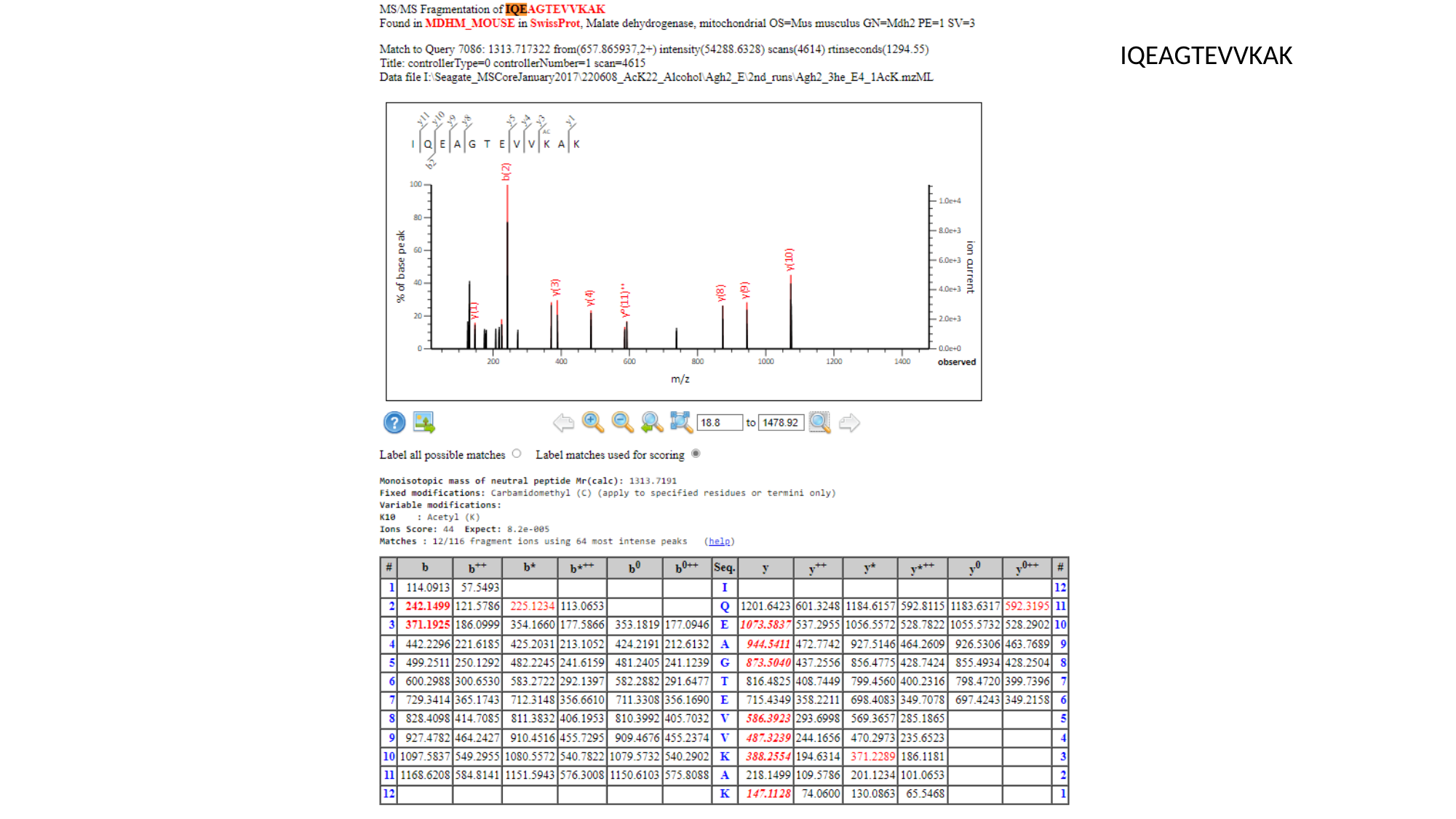

IQEAGTEVVKAK

## Slide 4
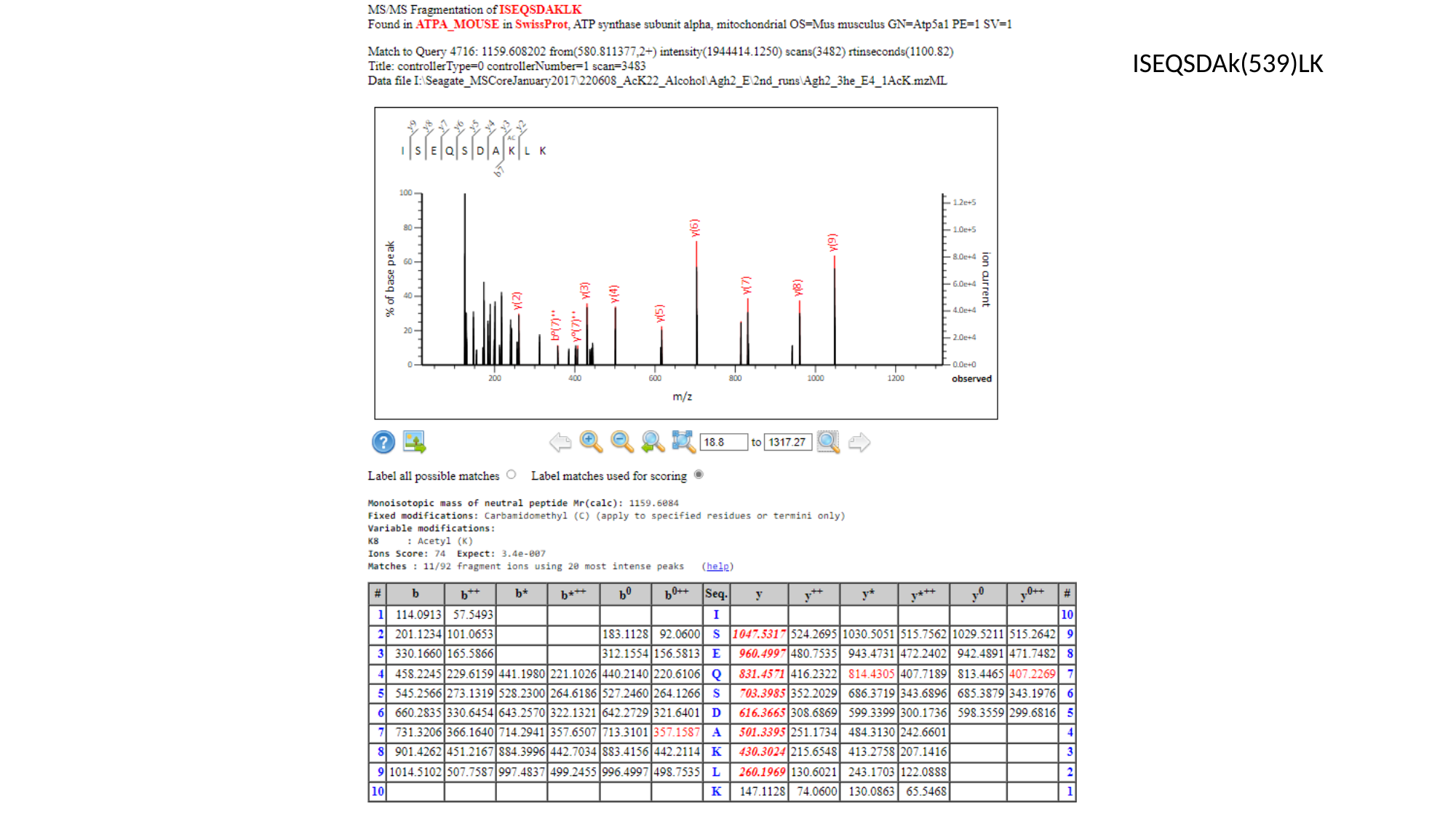

ISEQSDAk(539)LK

## Slide 5
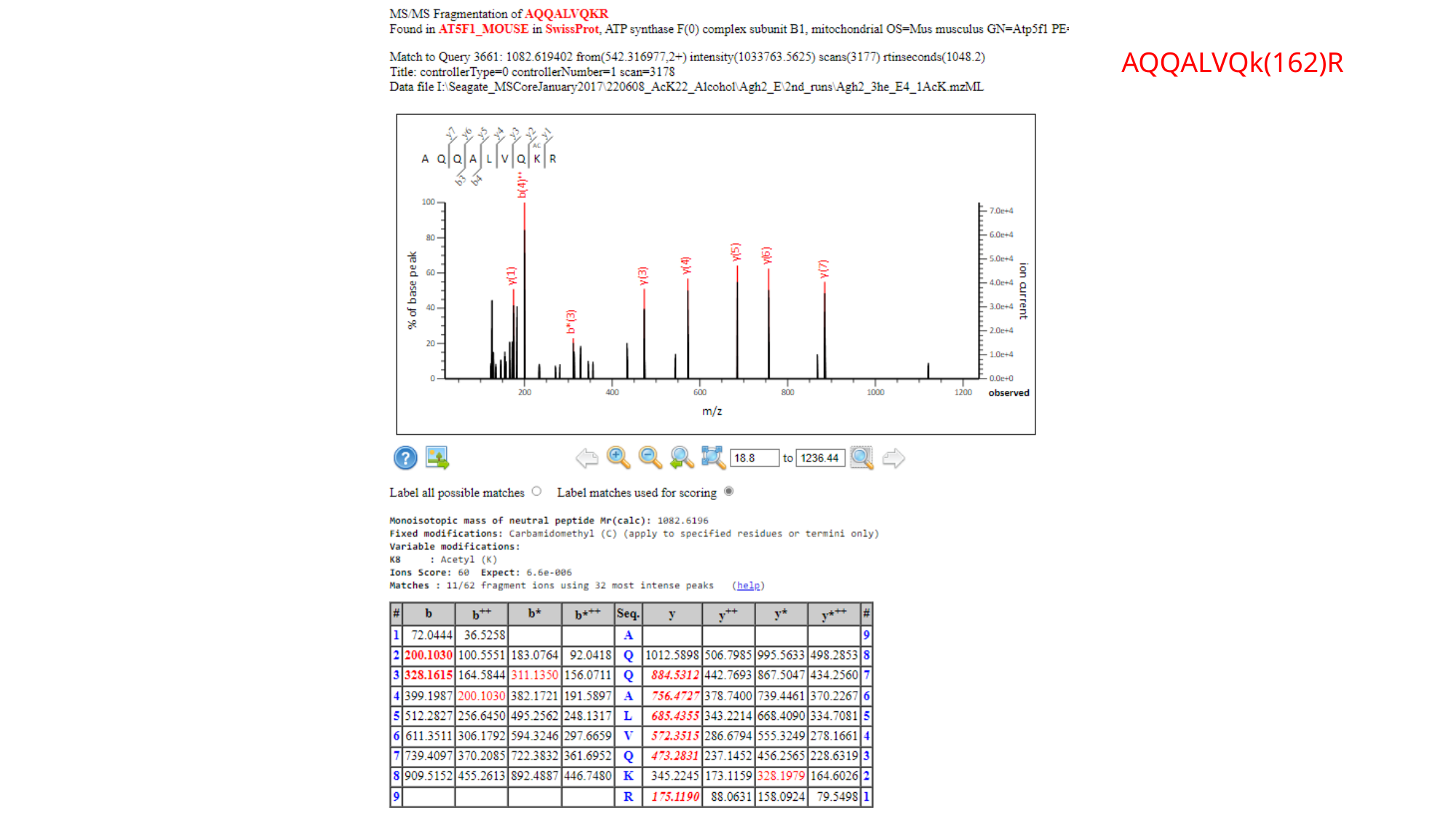

AQQALVQk(162)R

## Slide 6
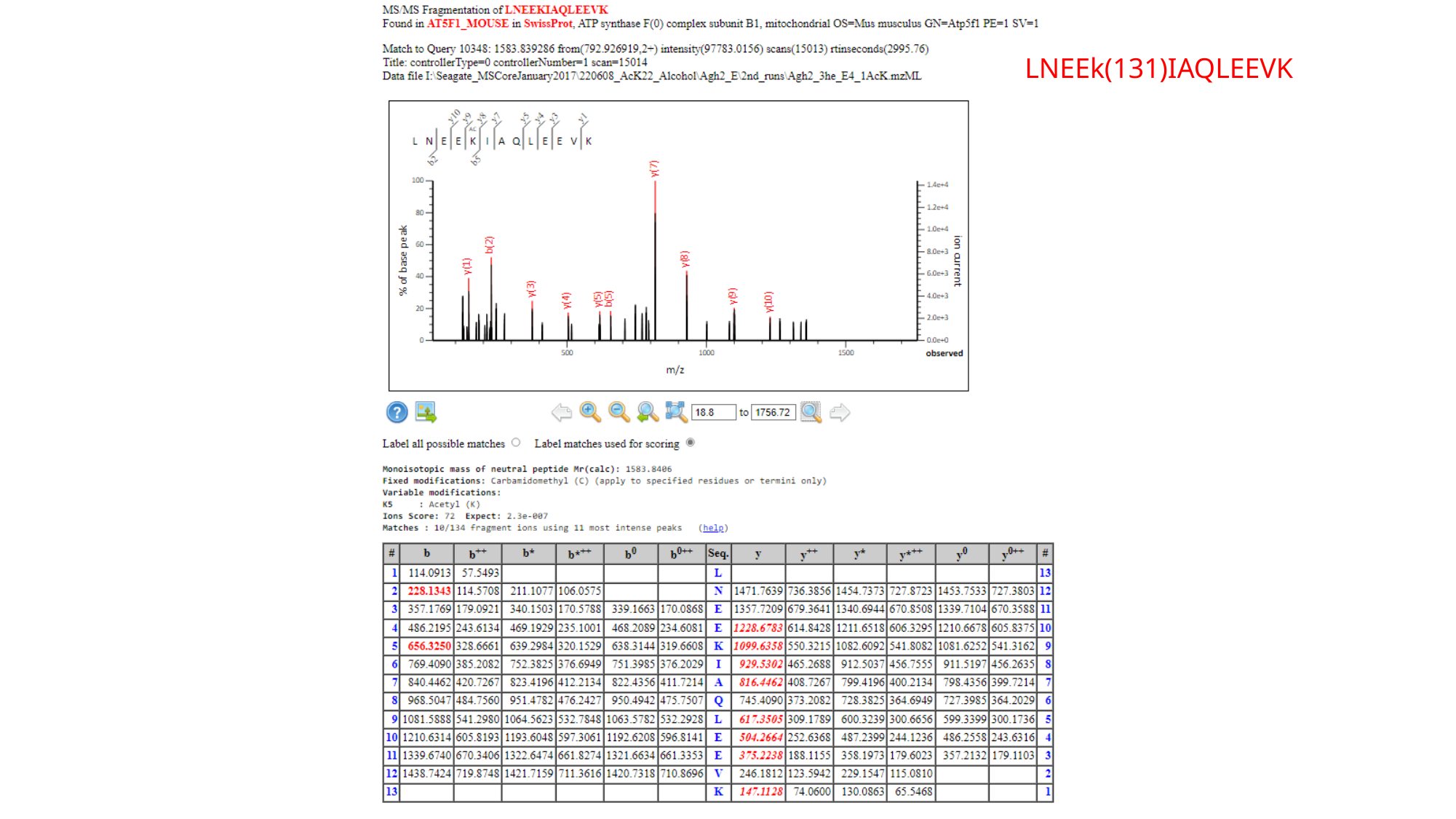

LNEEk(131)IAQLEEVK

## Slide 7
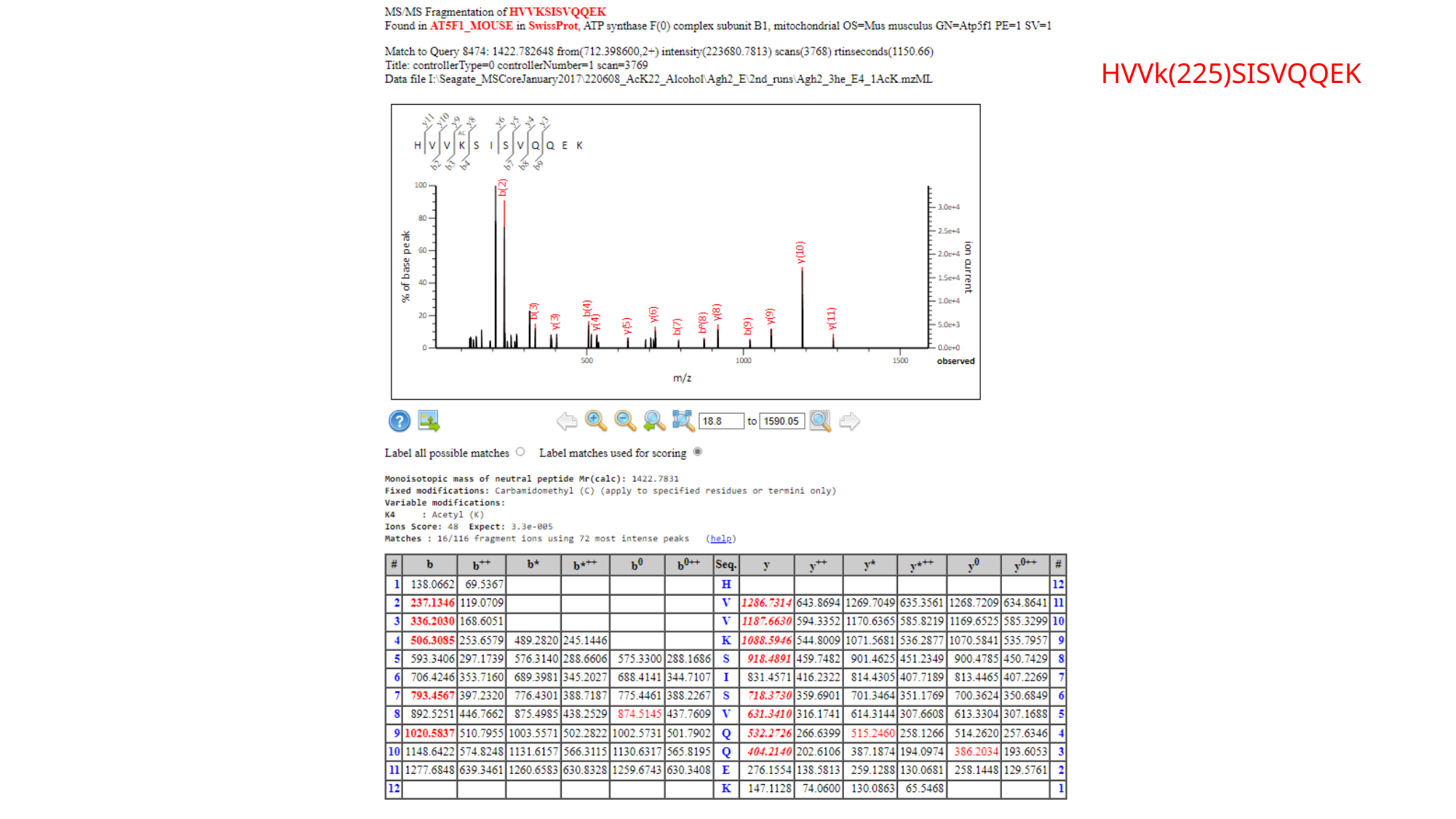

HVVk(225)SISVQQEK

## Slide 8
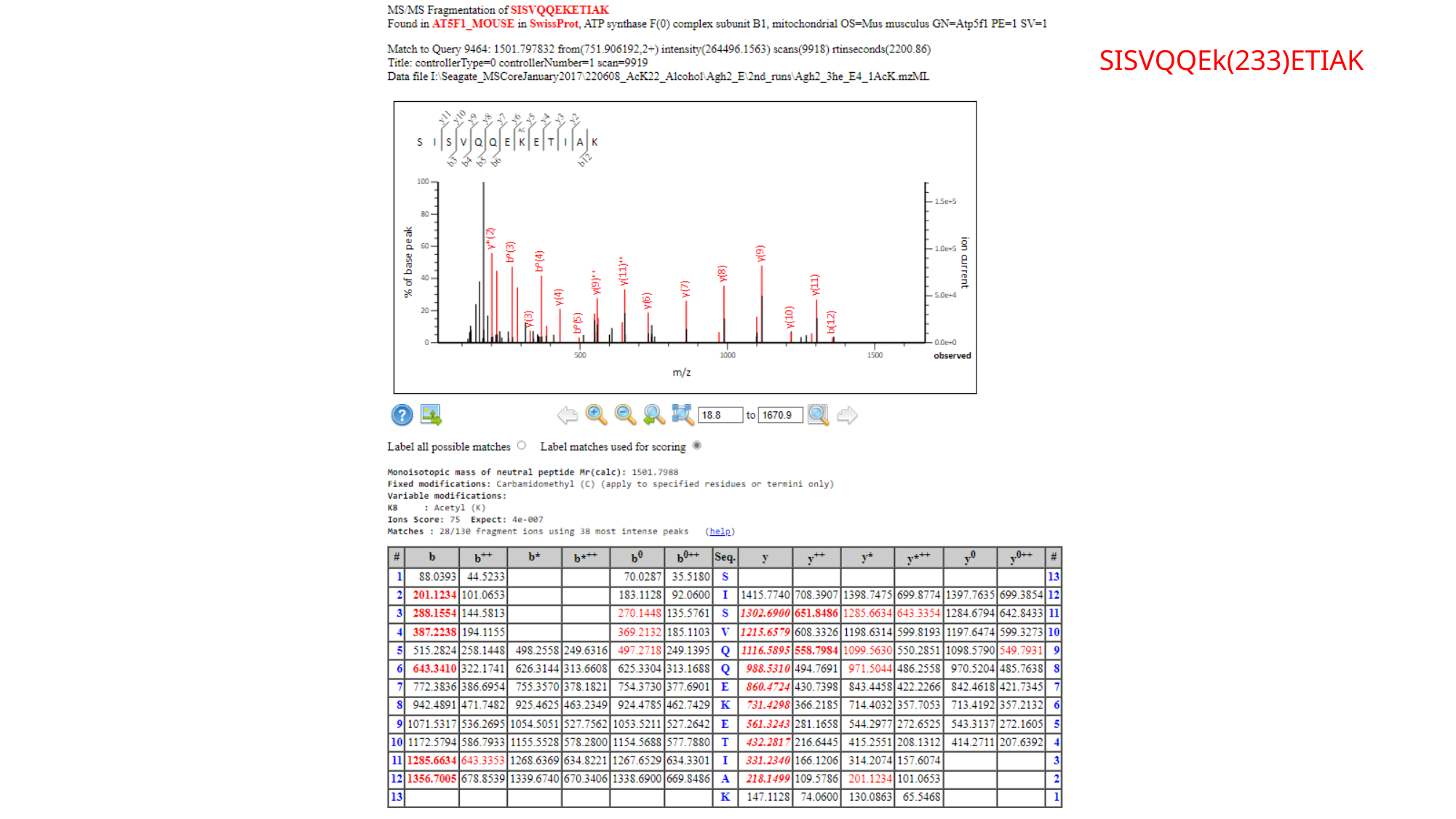

SISVQQEk(233)ETIAK

## Slide 9
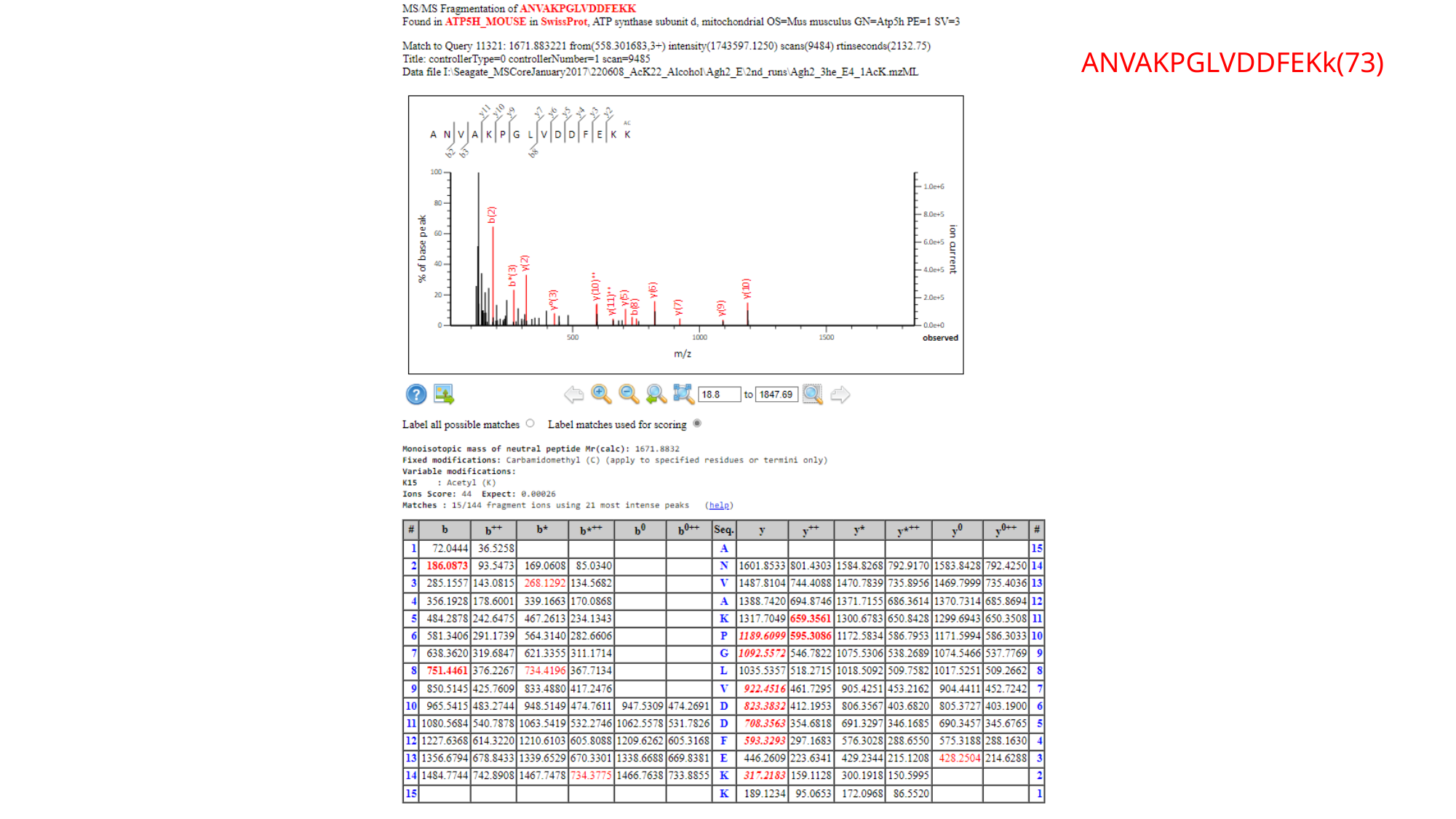

ANVAKPGLVDDFEKk(73)

## Slide 10
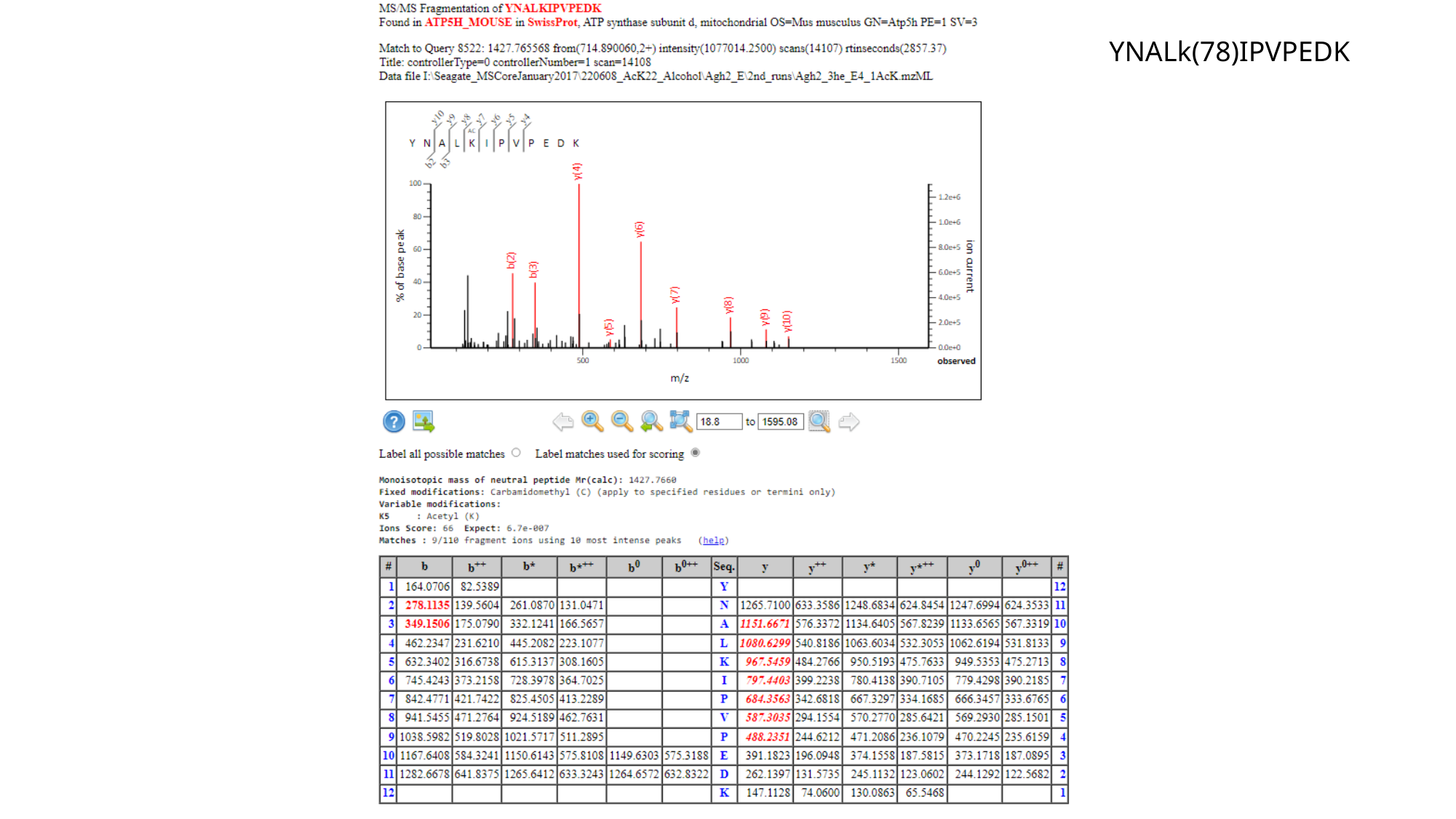

YNALk(78)IPVPEDK

## Slide 11
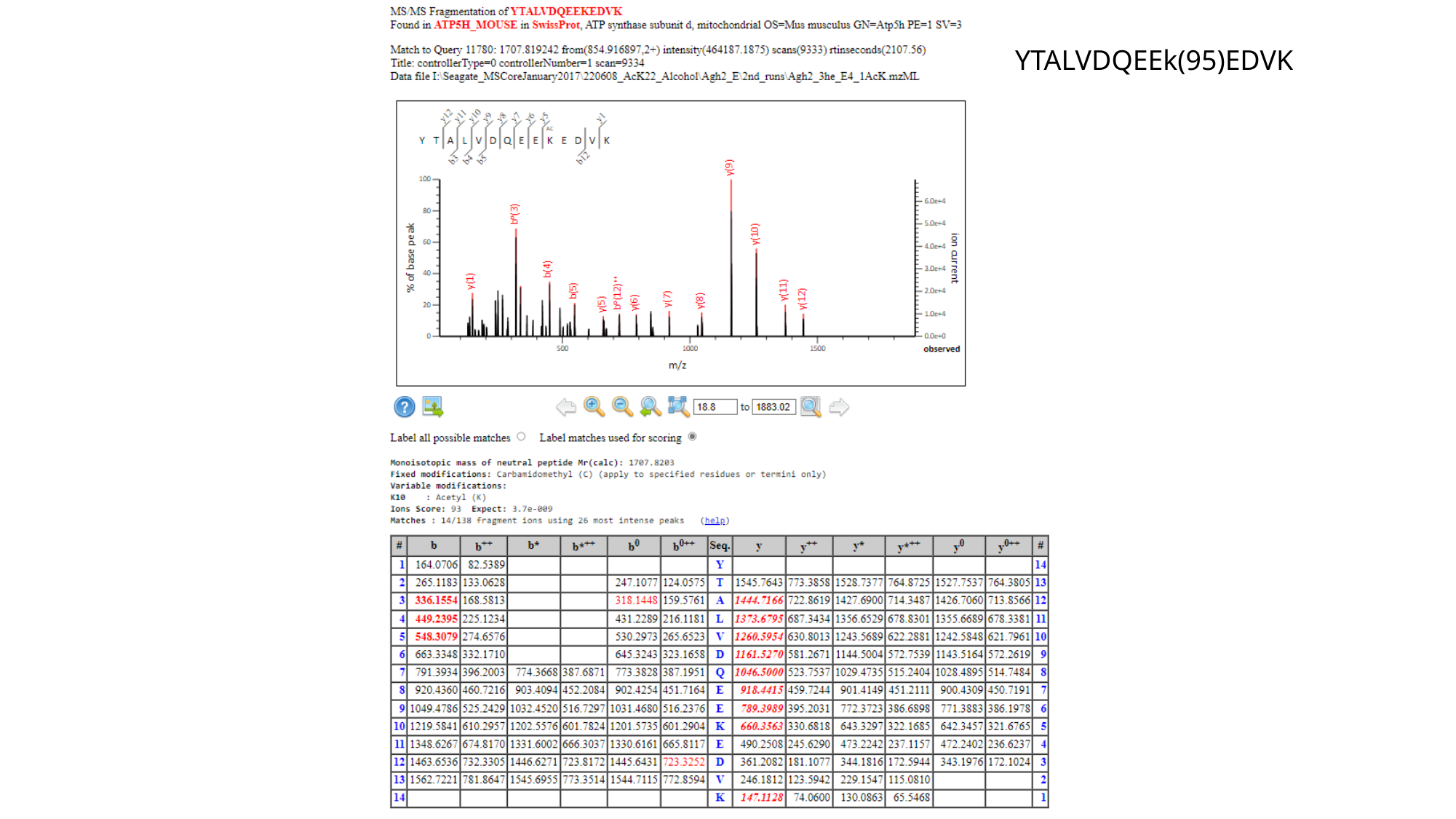

YTALVDQEEk(95)EDVK

## Slide 12
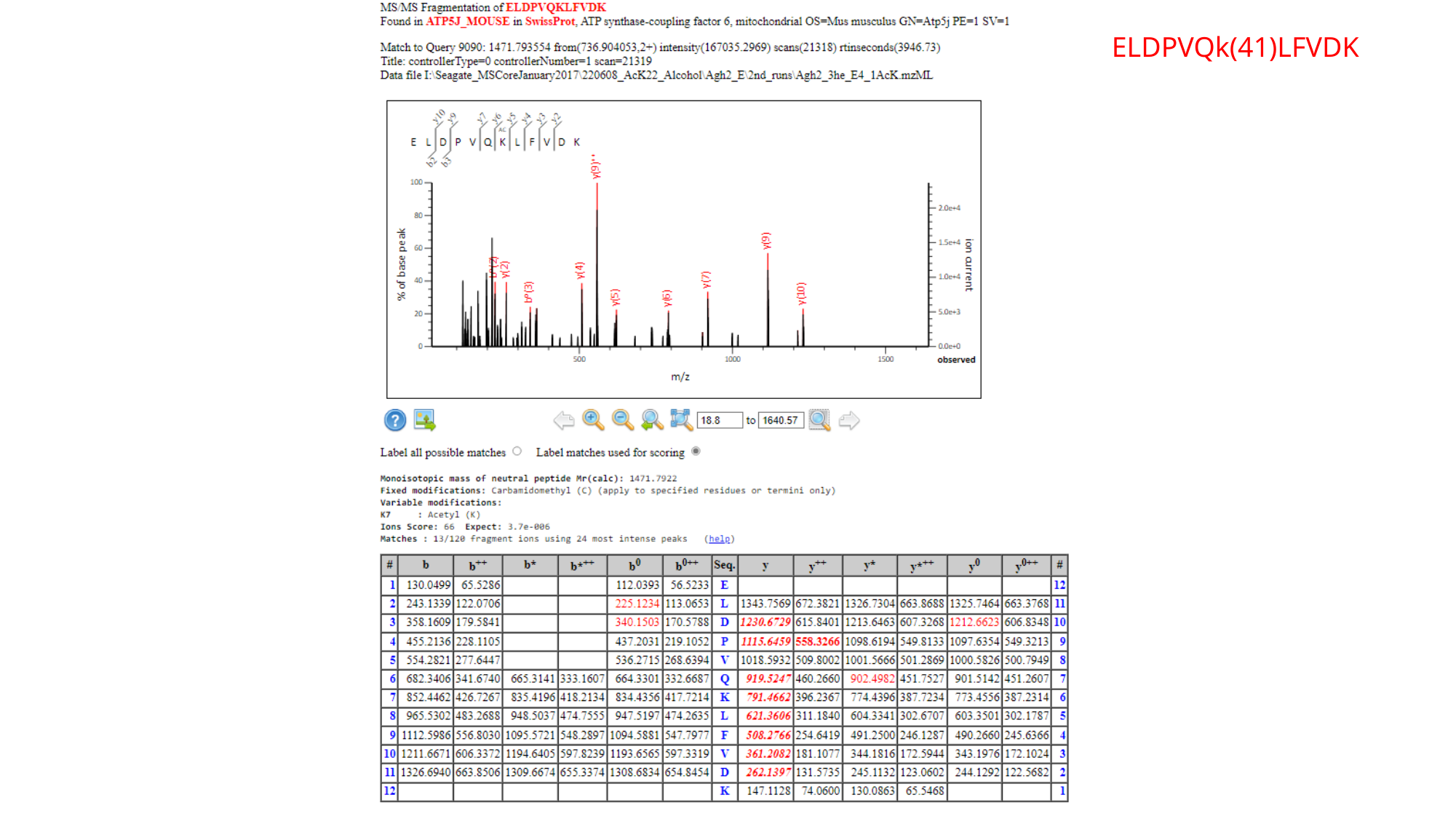

ELDPVQk(41)LFVDK

## Slide 13
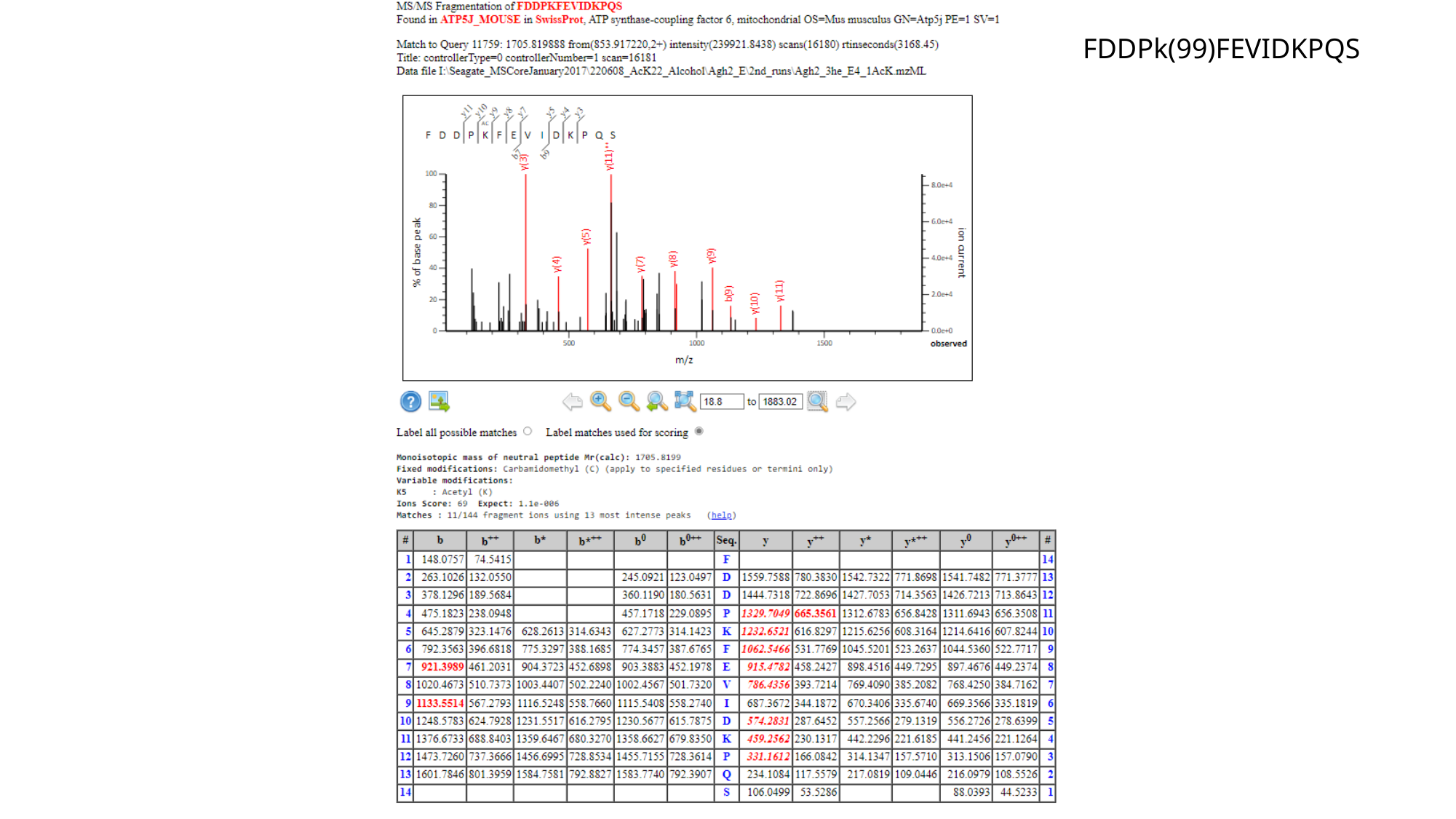

FDDPk(99)FEVIDKPQS

## Slide 14
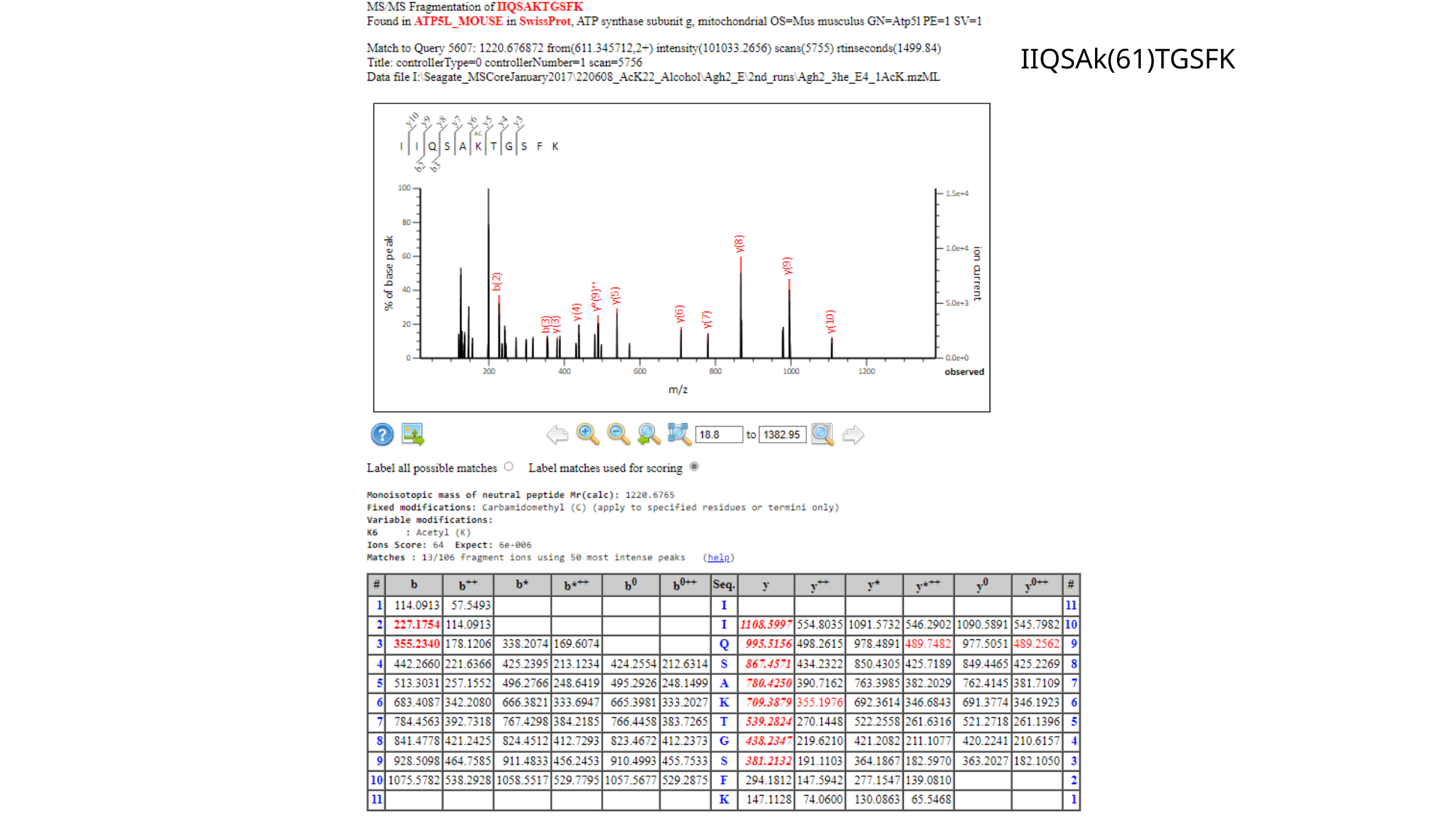

IIQSAk(61)TGSFK

## Slide 15
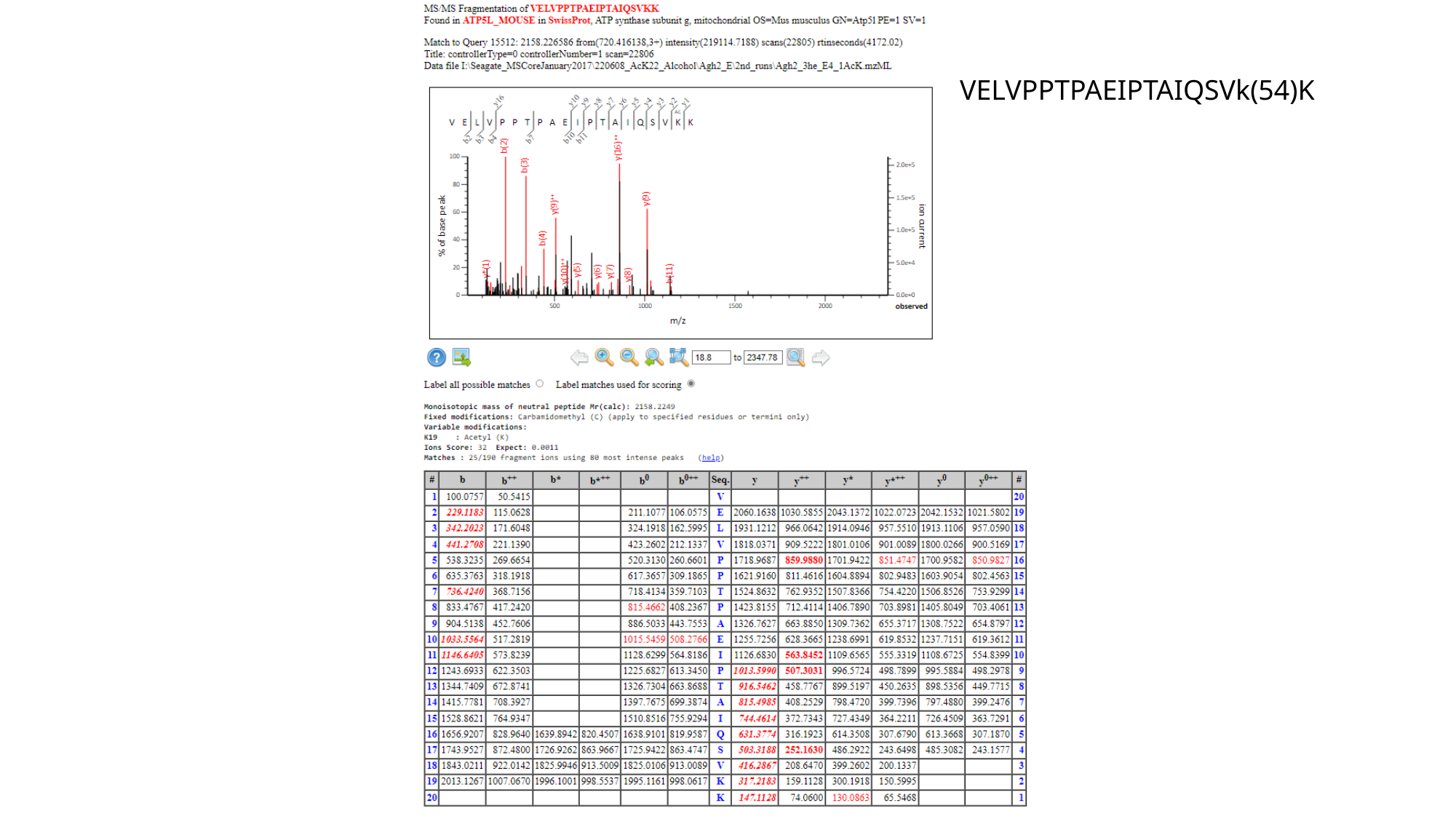

VELVPPTPAEIPTAIQSVk(54)K

## Slide 16
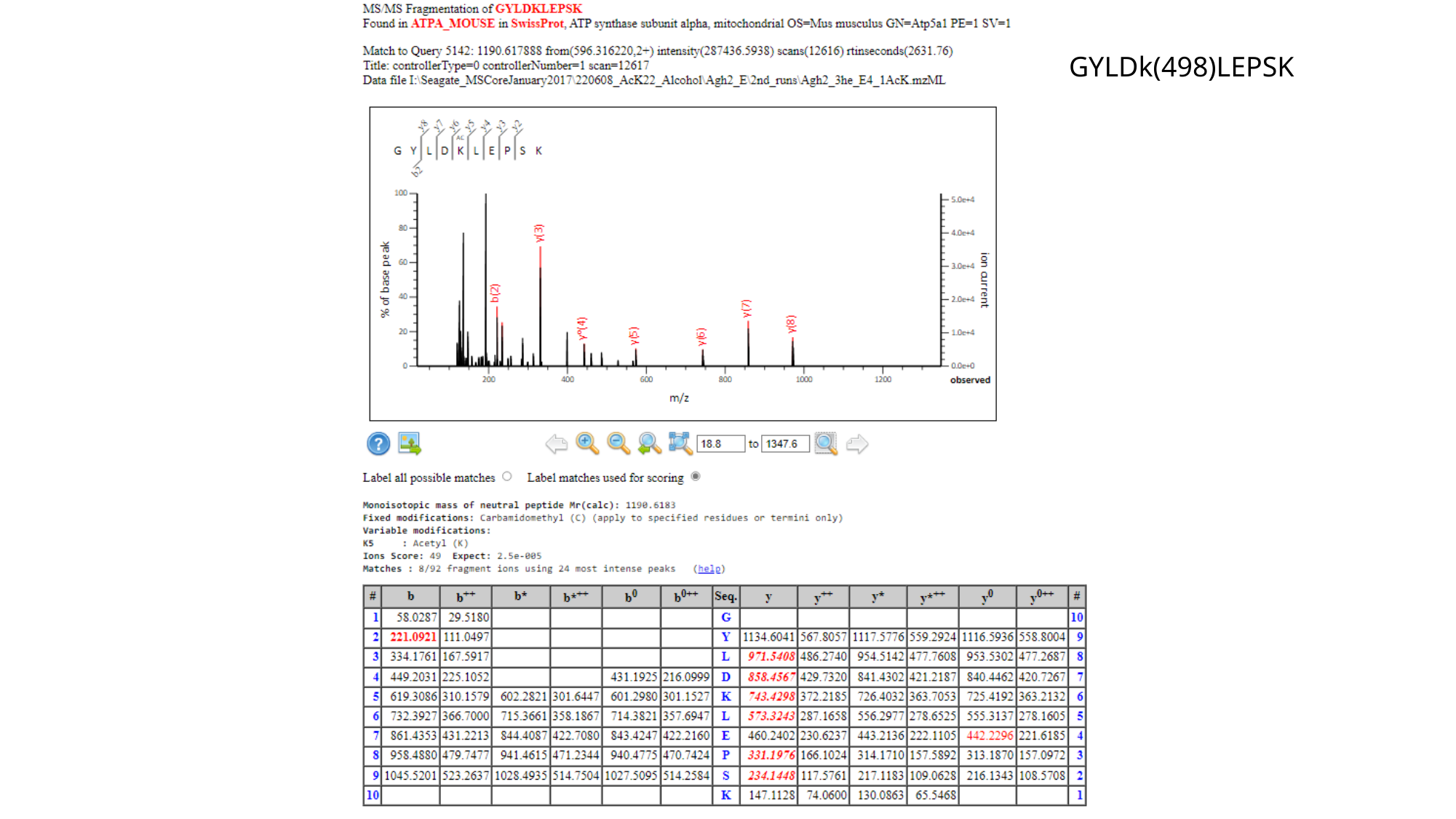

GYLDk(498)LEPSK

## Slide 17
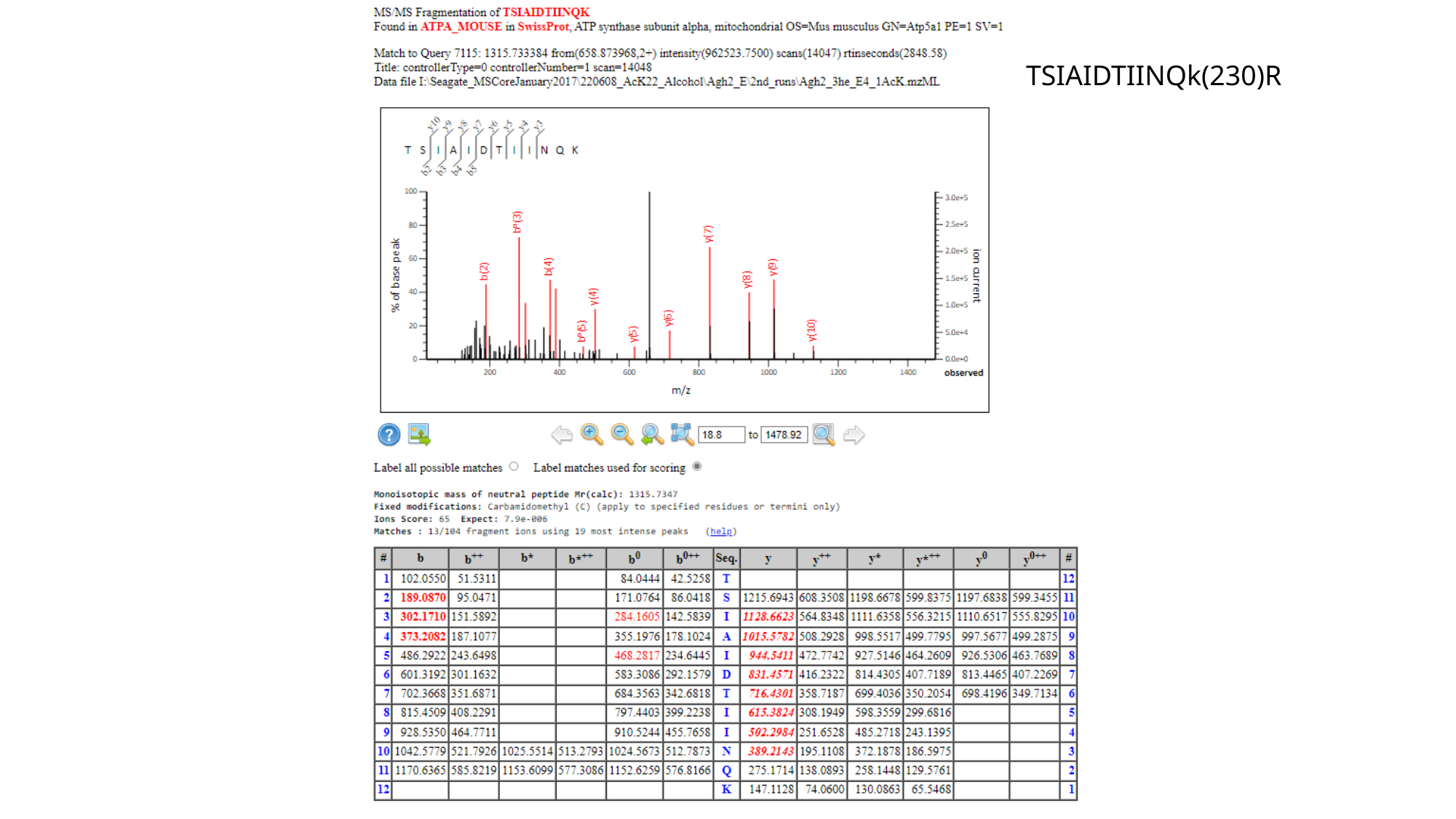

TSIAIDTIINQk(230)R

## Slide 18
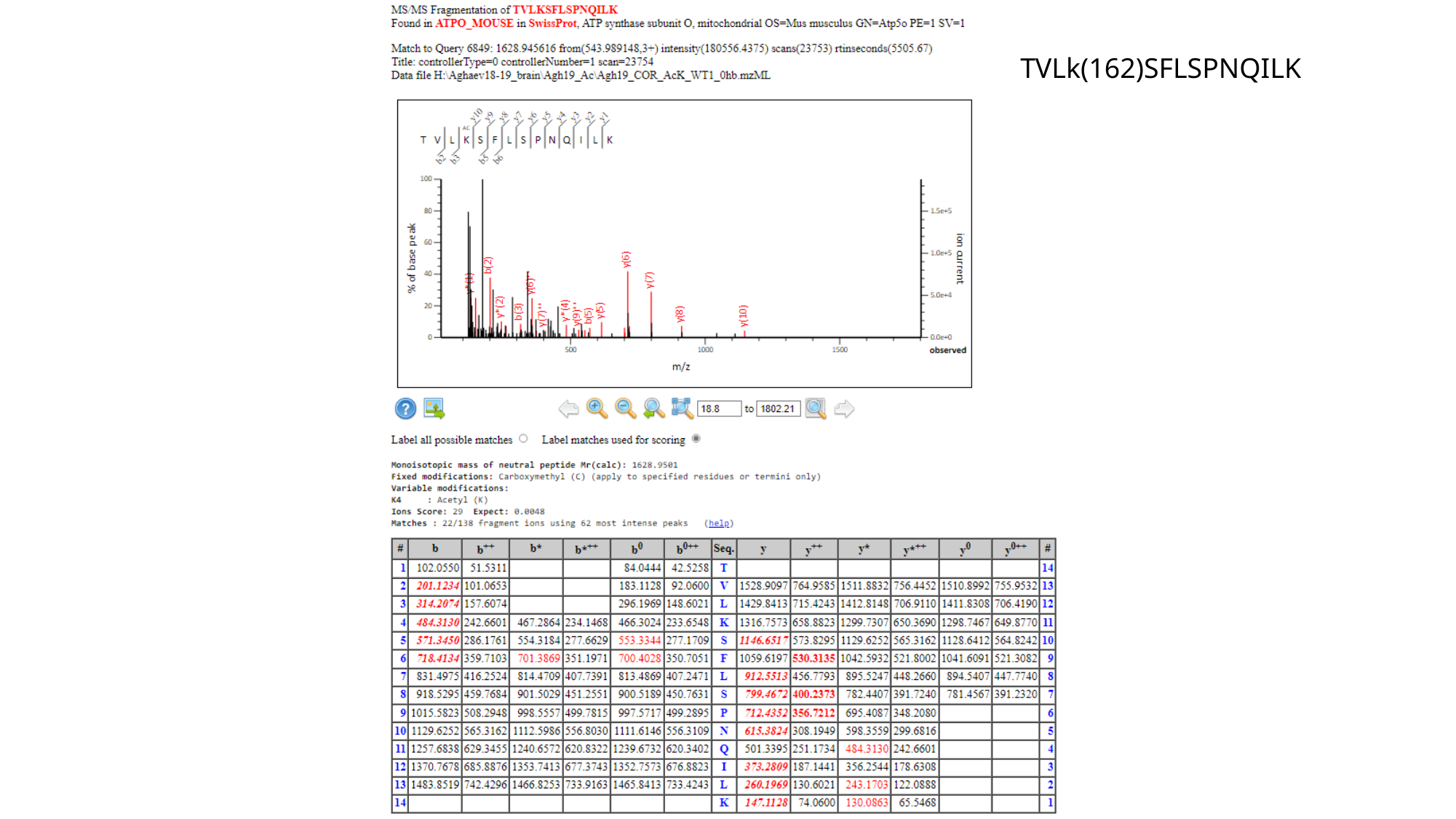

TVLk(162)SFLSPNQILK

## Slide 19
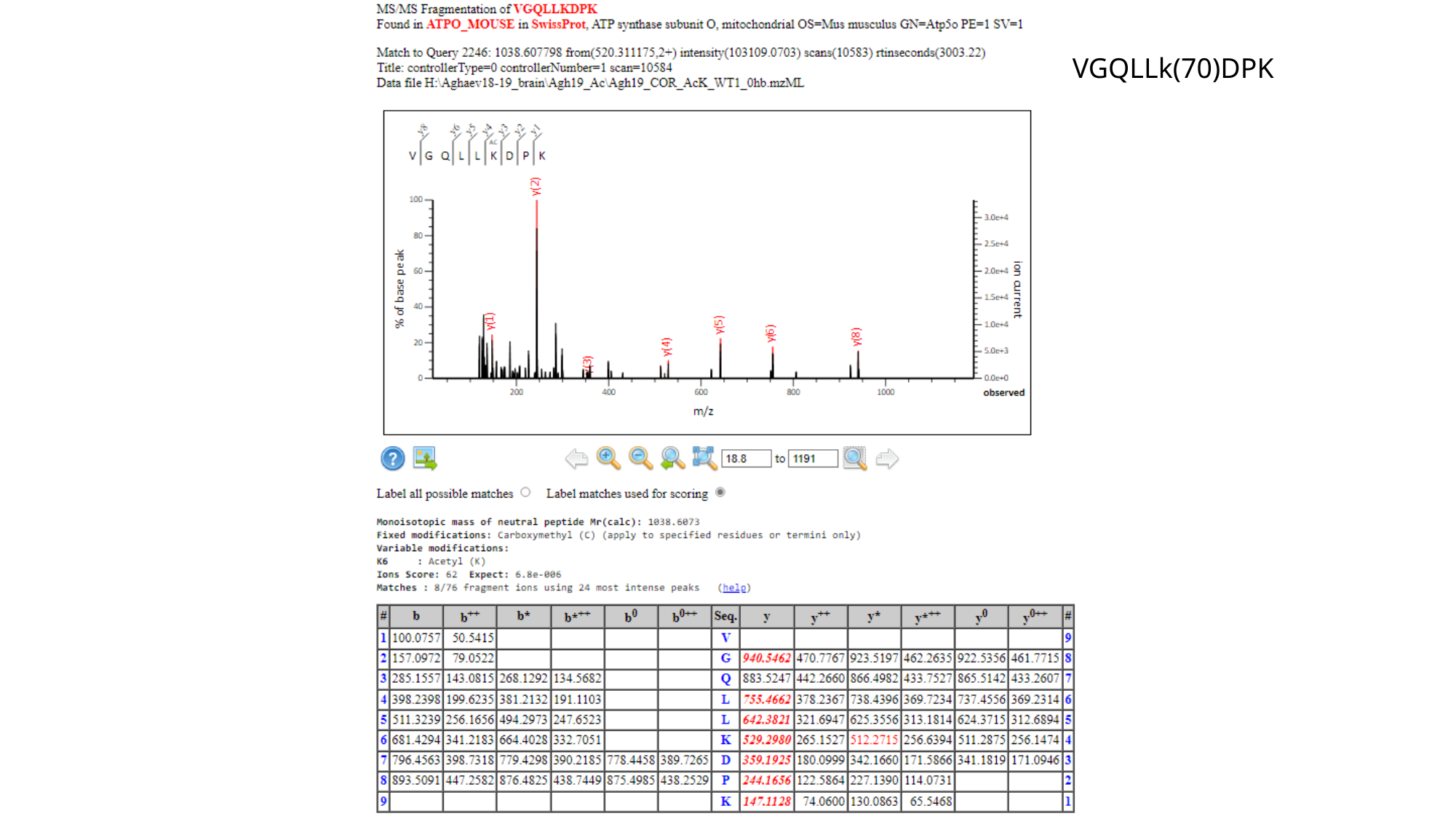

VGQLLk(70)DPK

## Slide 20
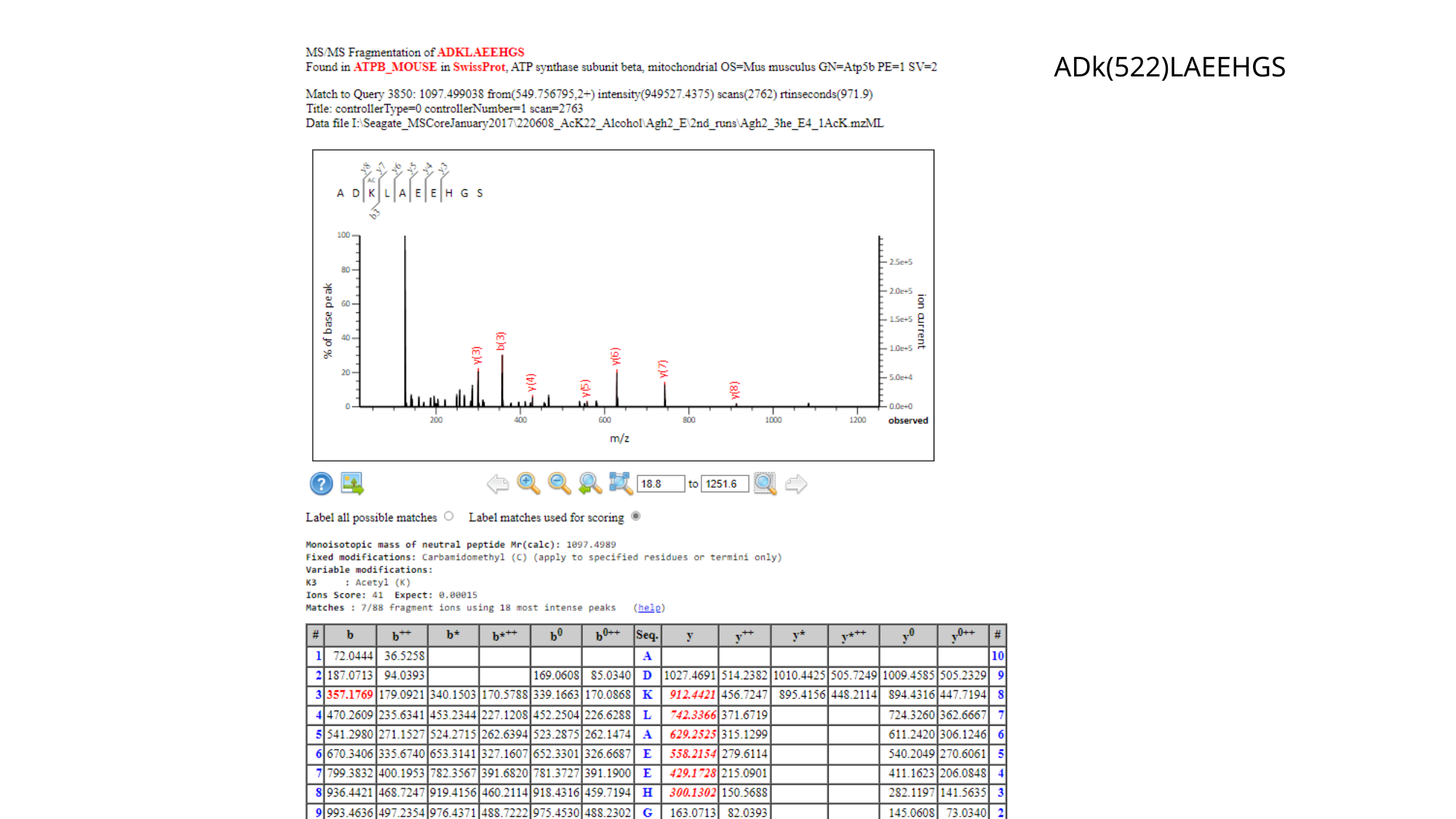

ADk(522)LAEEHGS

## Slide 21
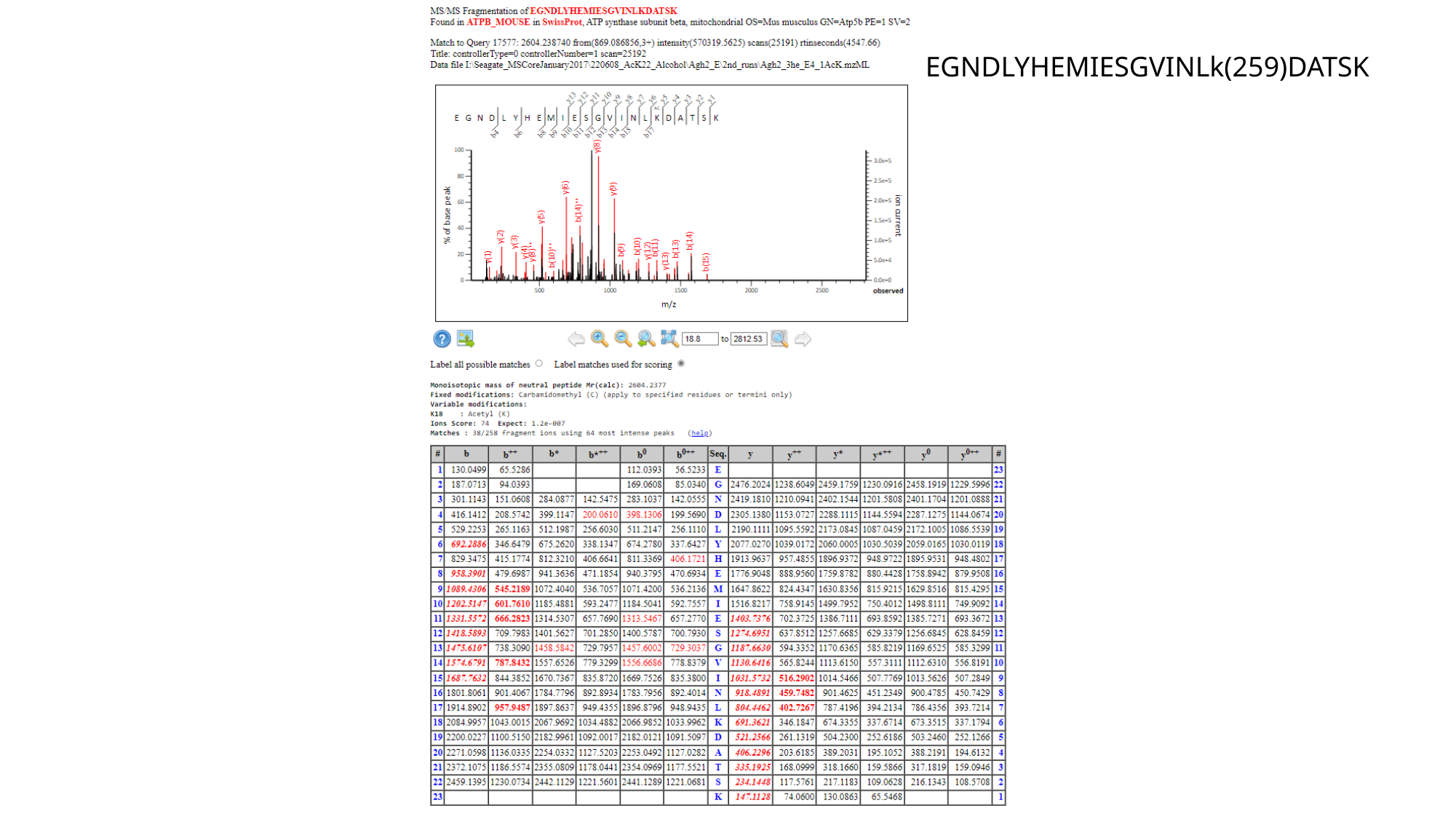

EGNDLYHEMIESGVINLk(259)DATSK

## Slide 22
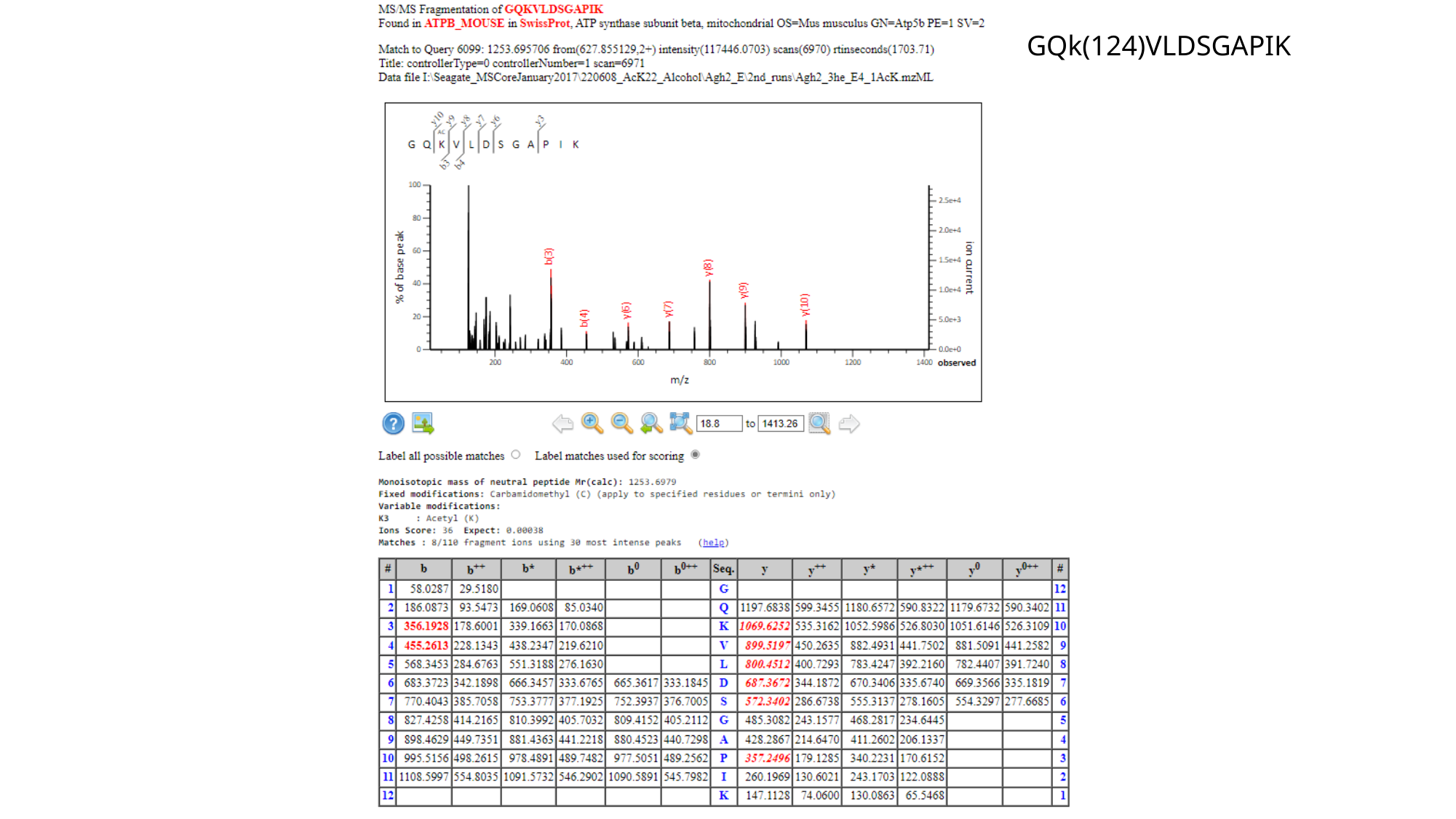

GQk(124)VLDSGAPIK

## Slide 23
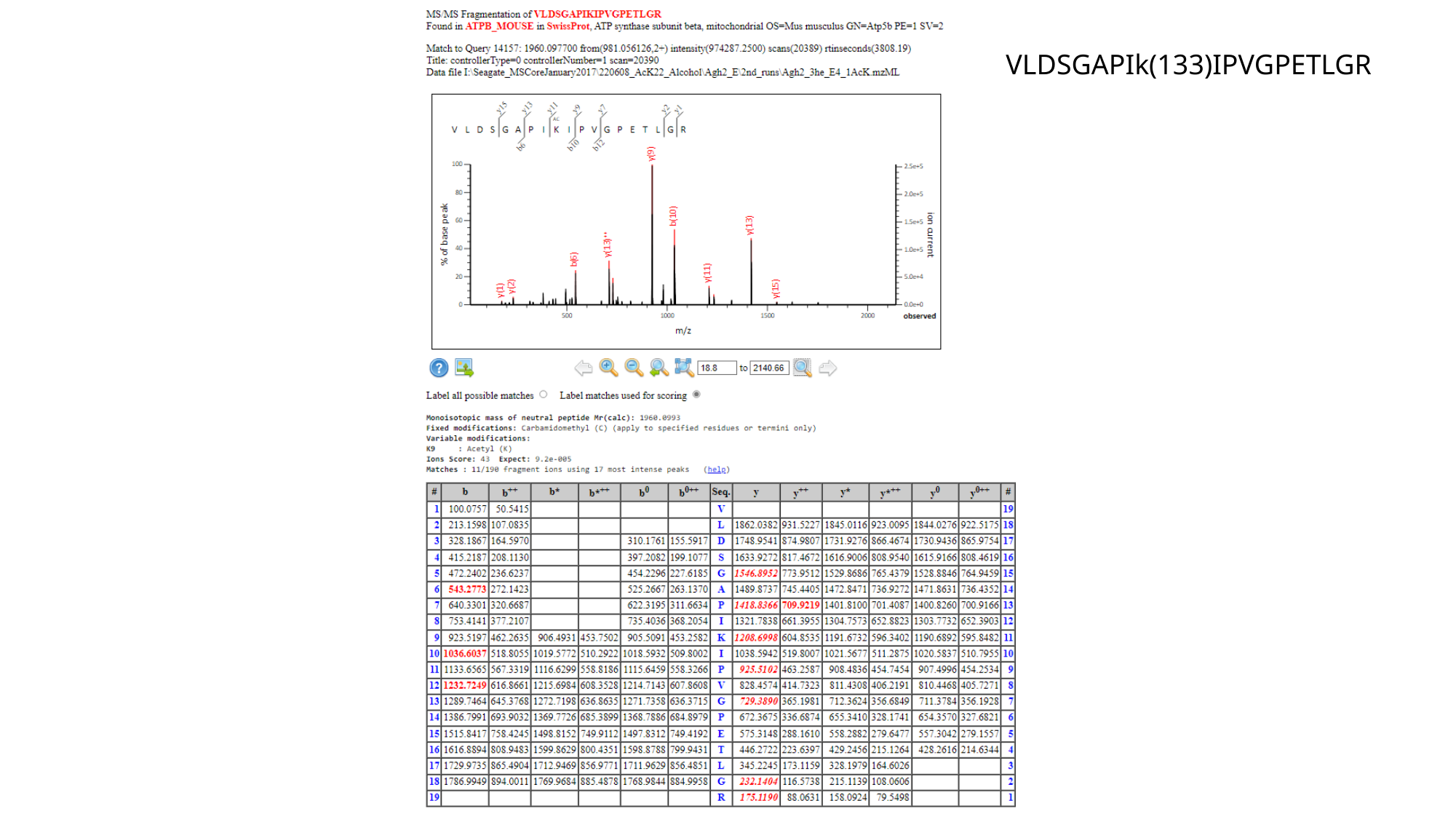

VLDSGAPIk(133)IPVGPETLGR

## Slide 24
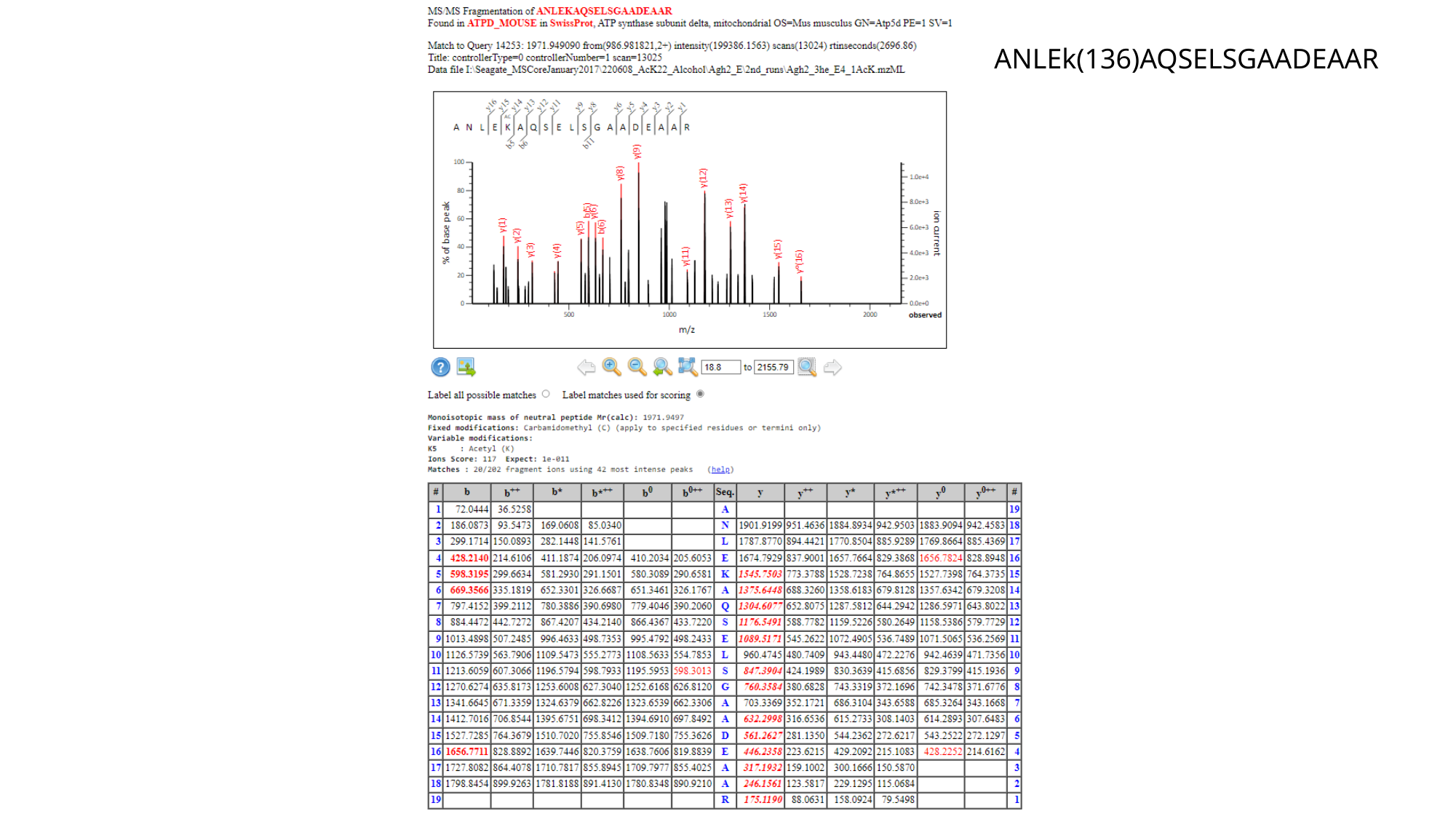

ANLEk(136)AQSELSGAADEAAR

## Slide 25
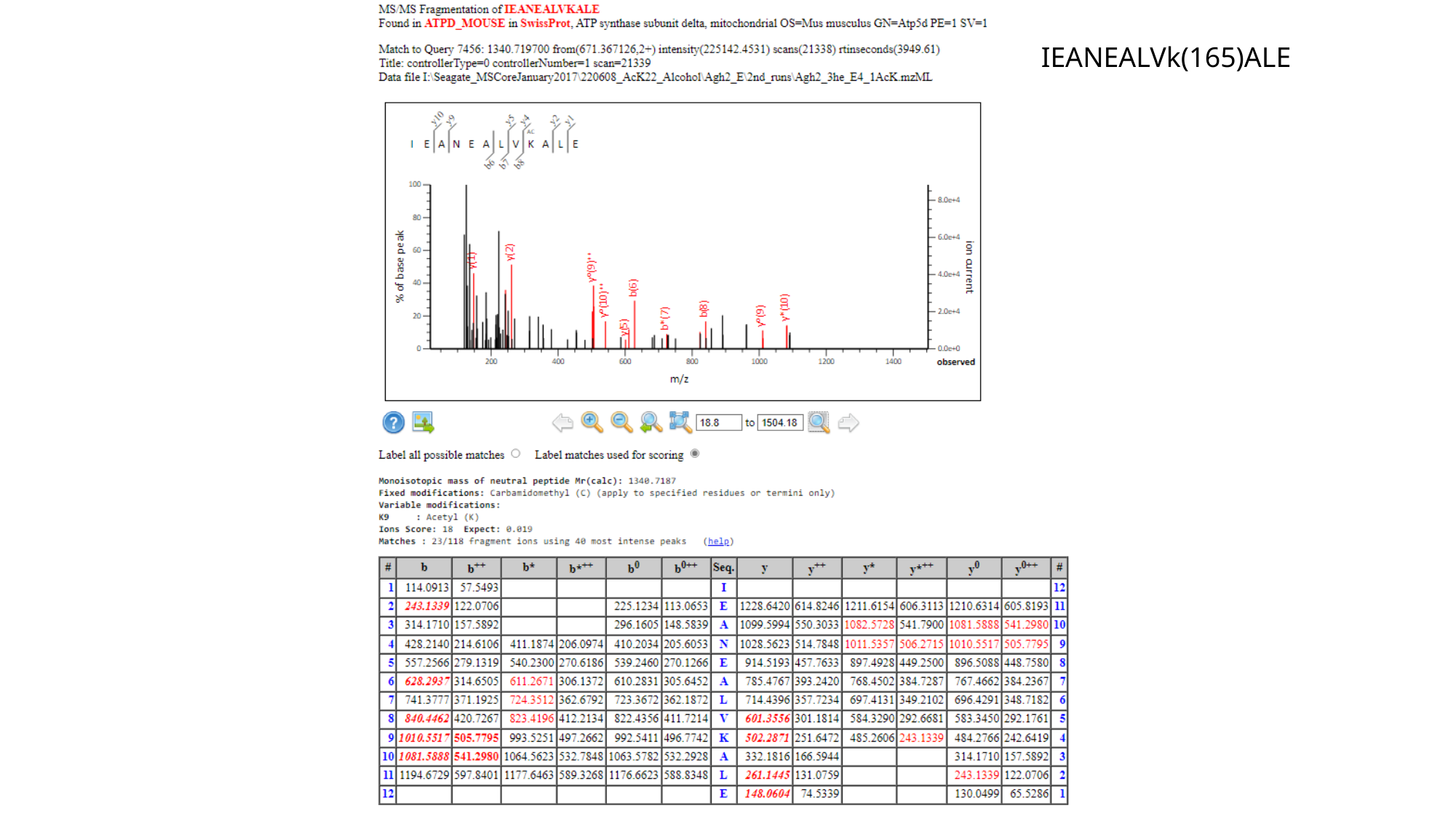

IEANEALVk(165)ALE

## Slide 26
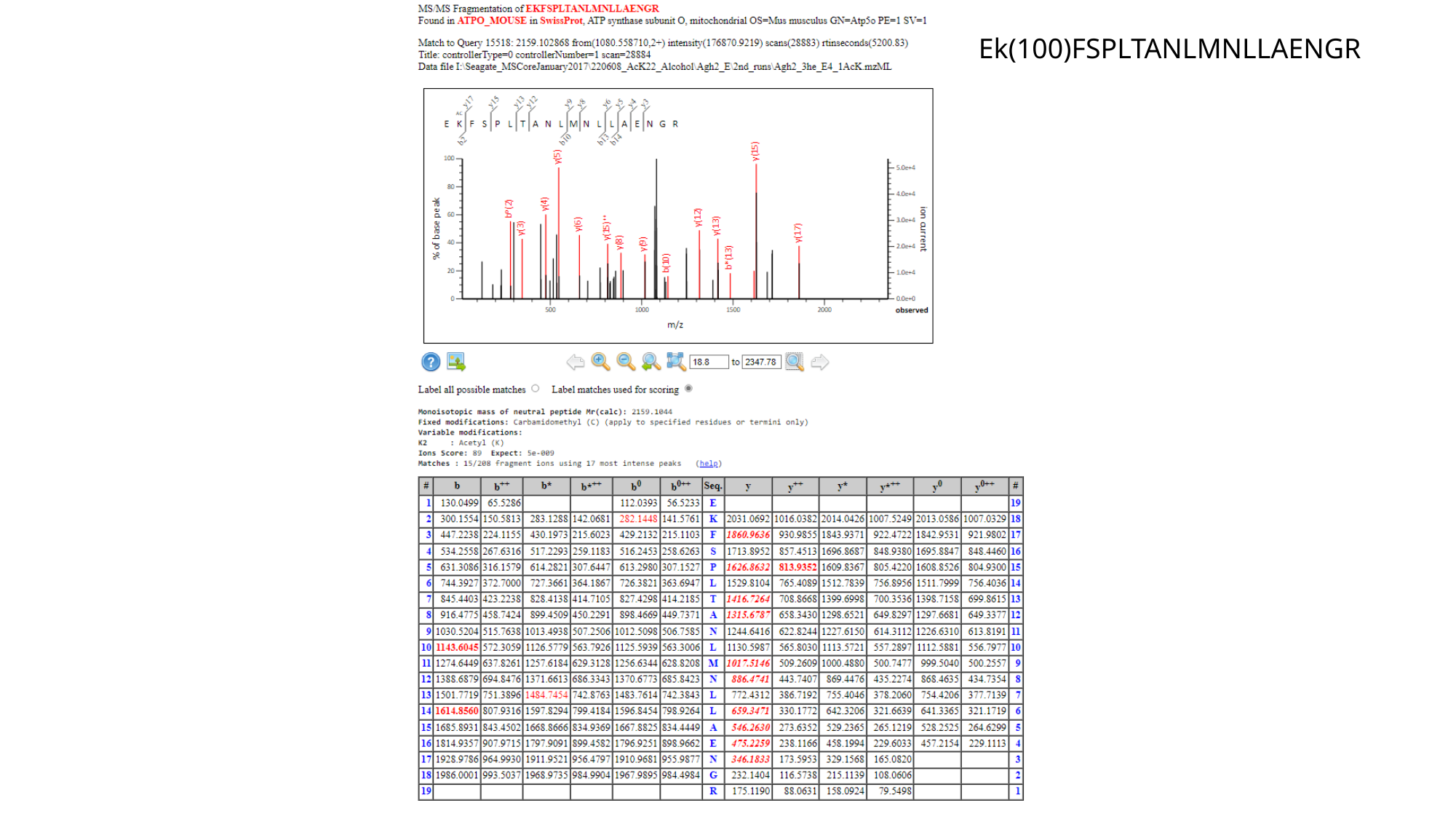

Ek(100)FSPLTANLMNLLAENGR

## Slide 27
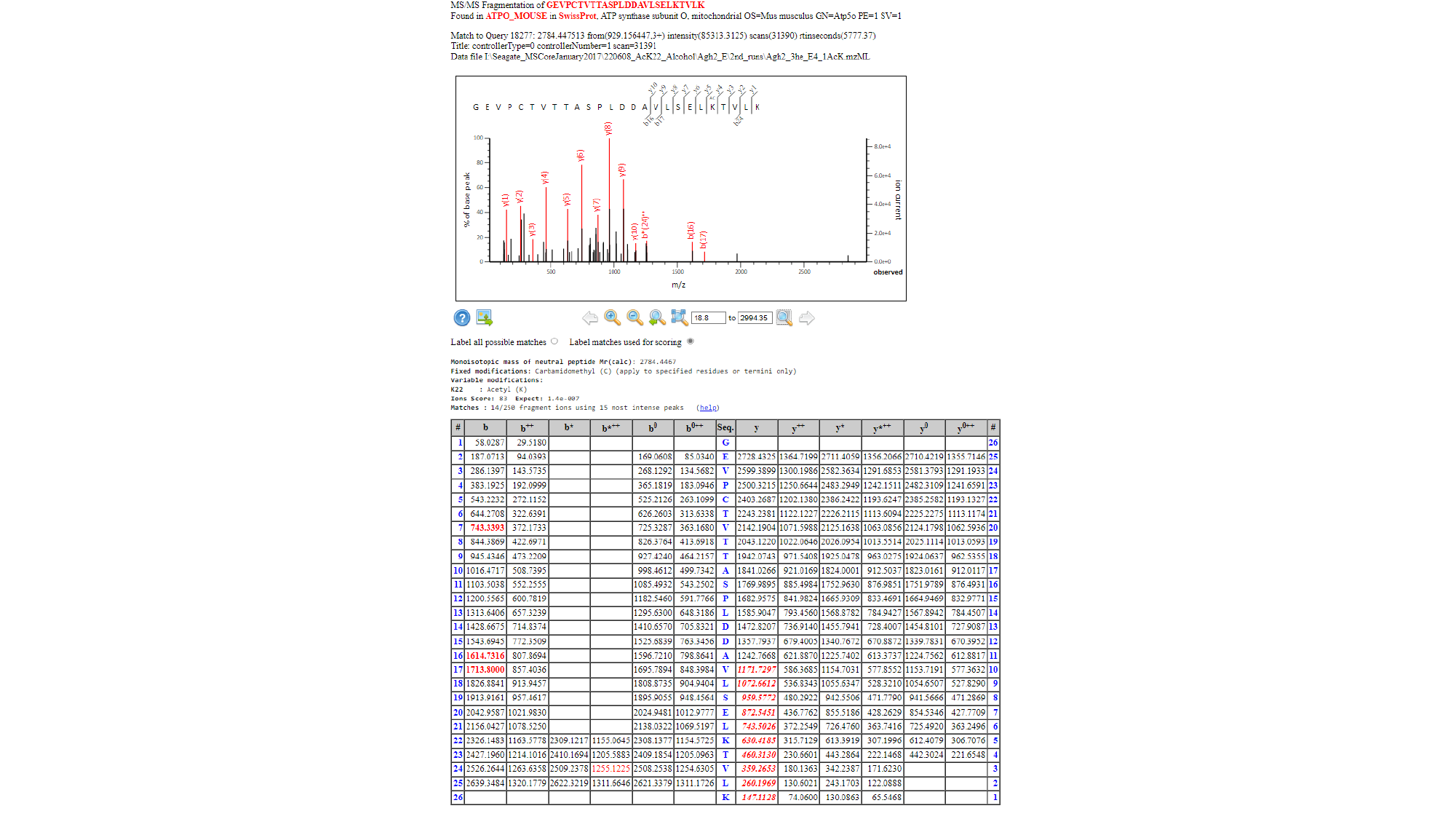

## Slide 28
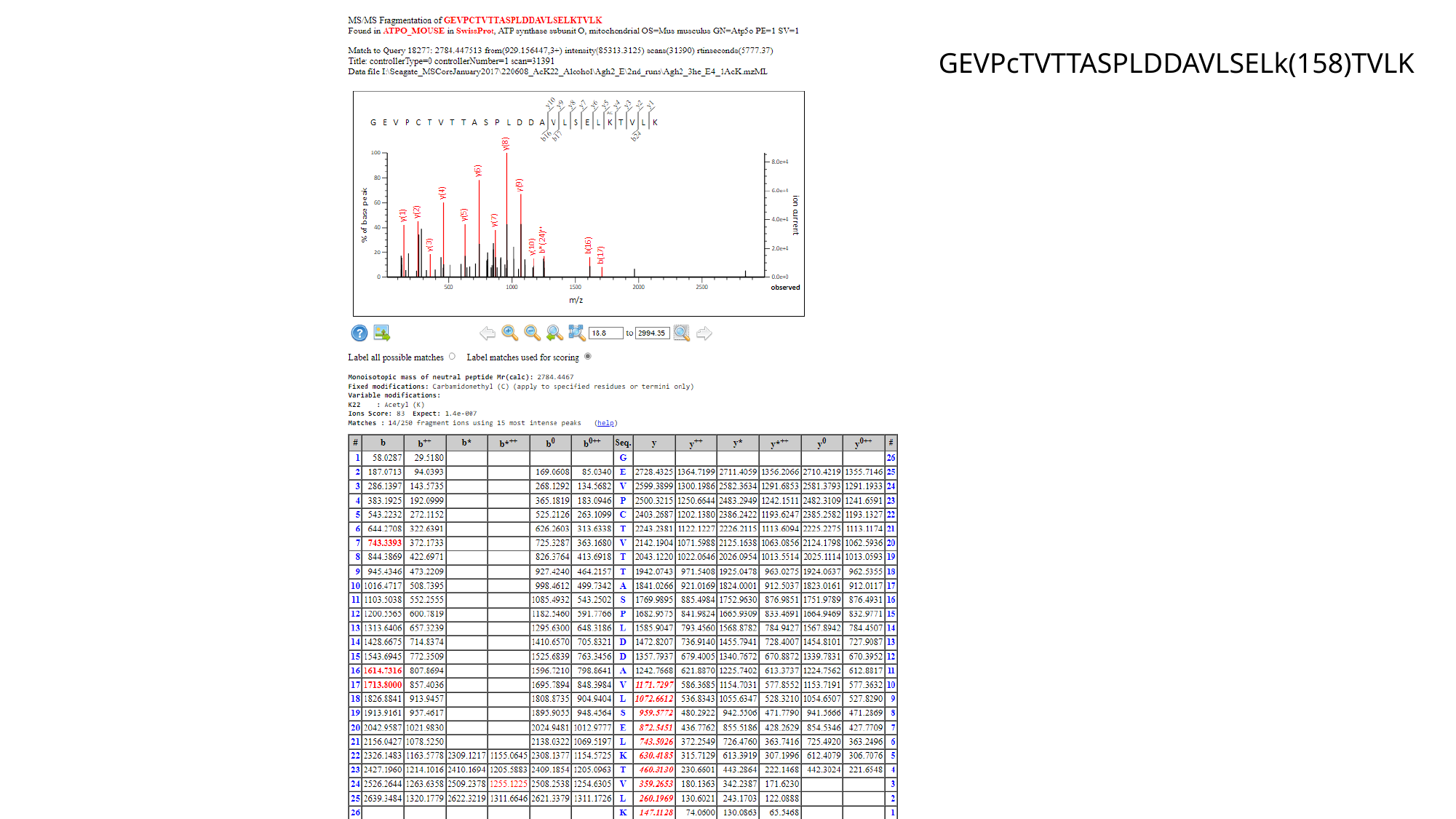

GEVPcTVTTASPLDDAVLSELk(158)TVLK

## Slide 29
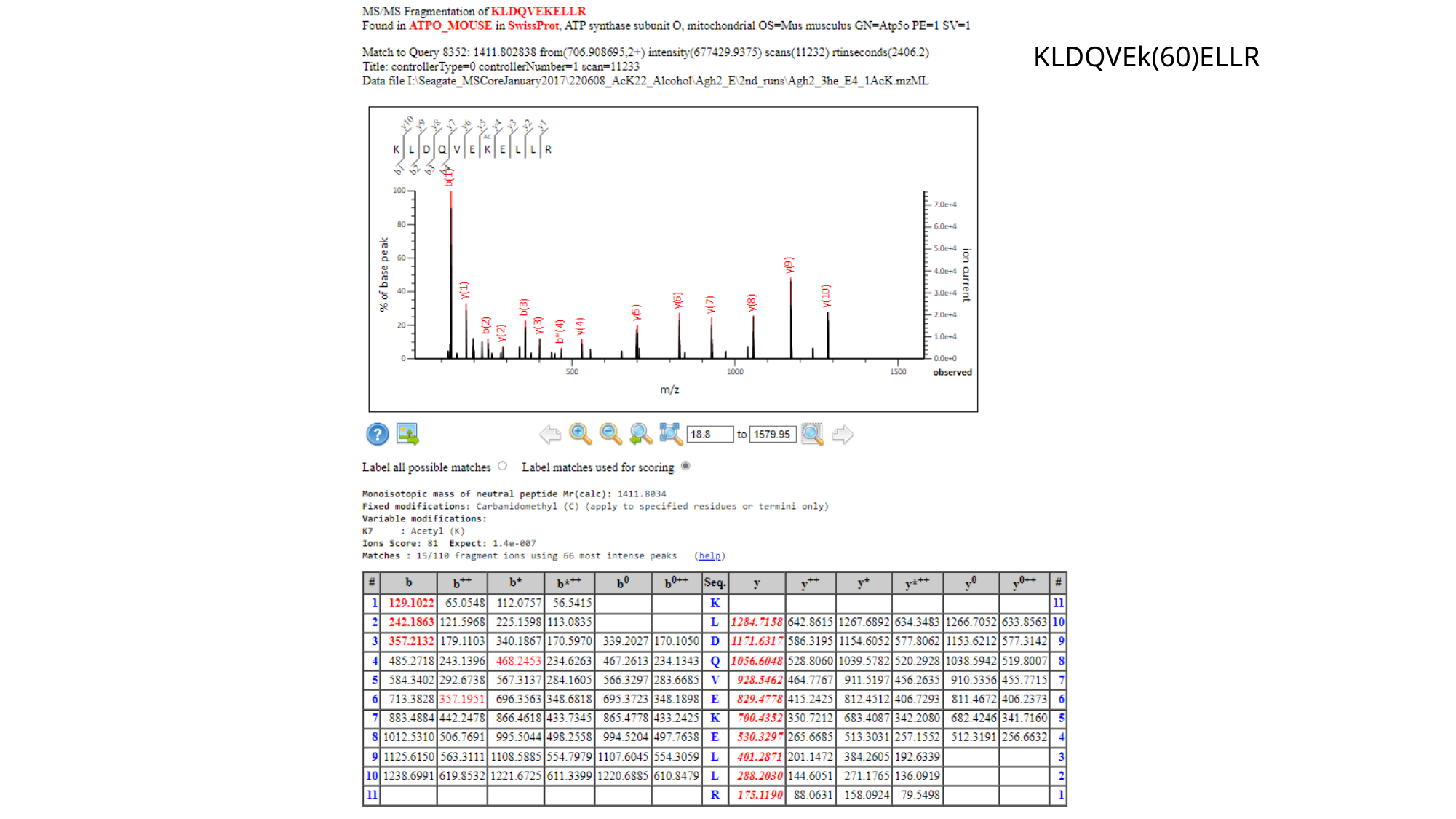

KLDQVEk(60)ELLR

## Slide 30
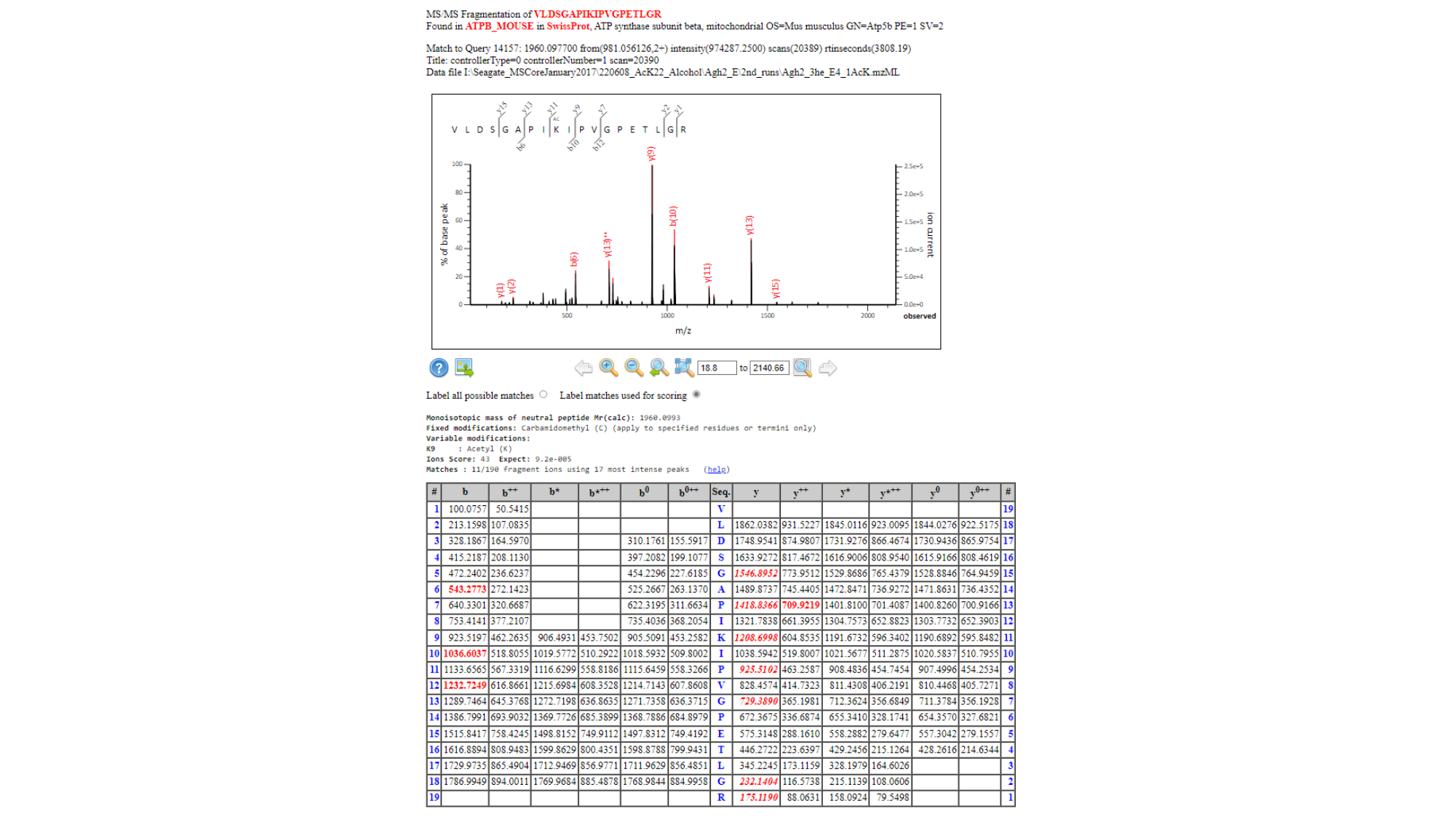

## Slide 31
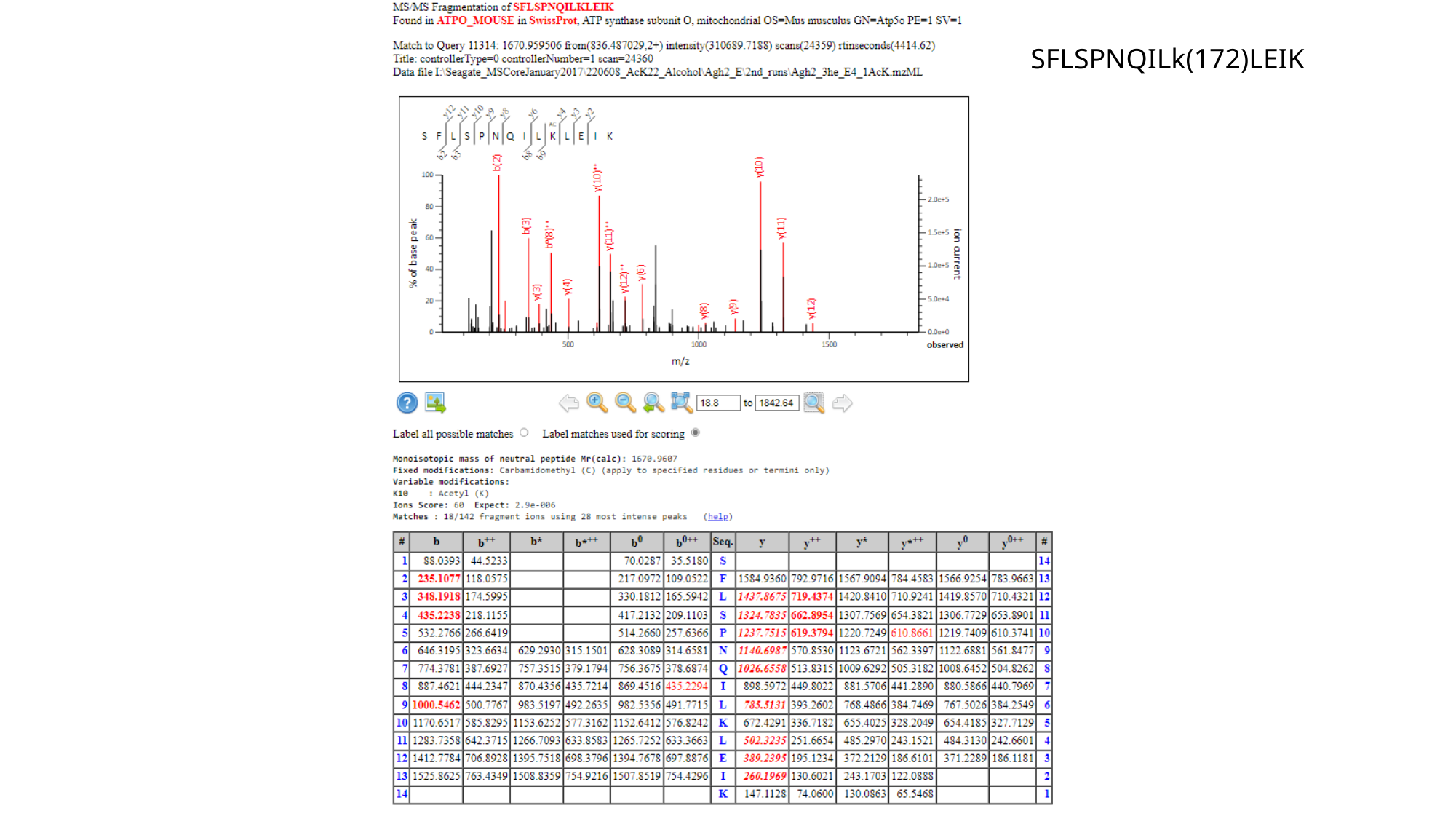

SFLSPNQILk(172)LEIK

## Slide 32
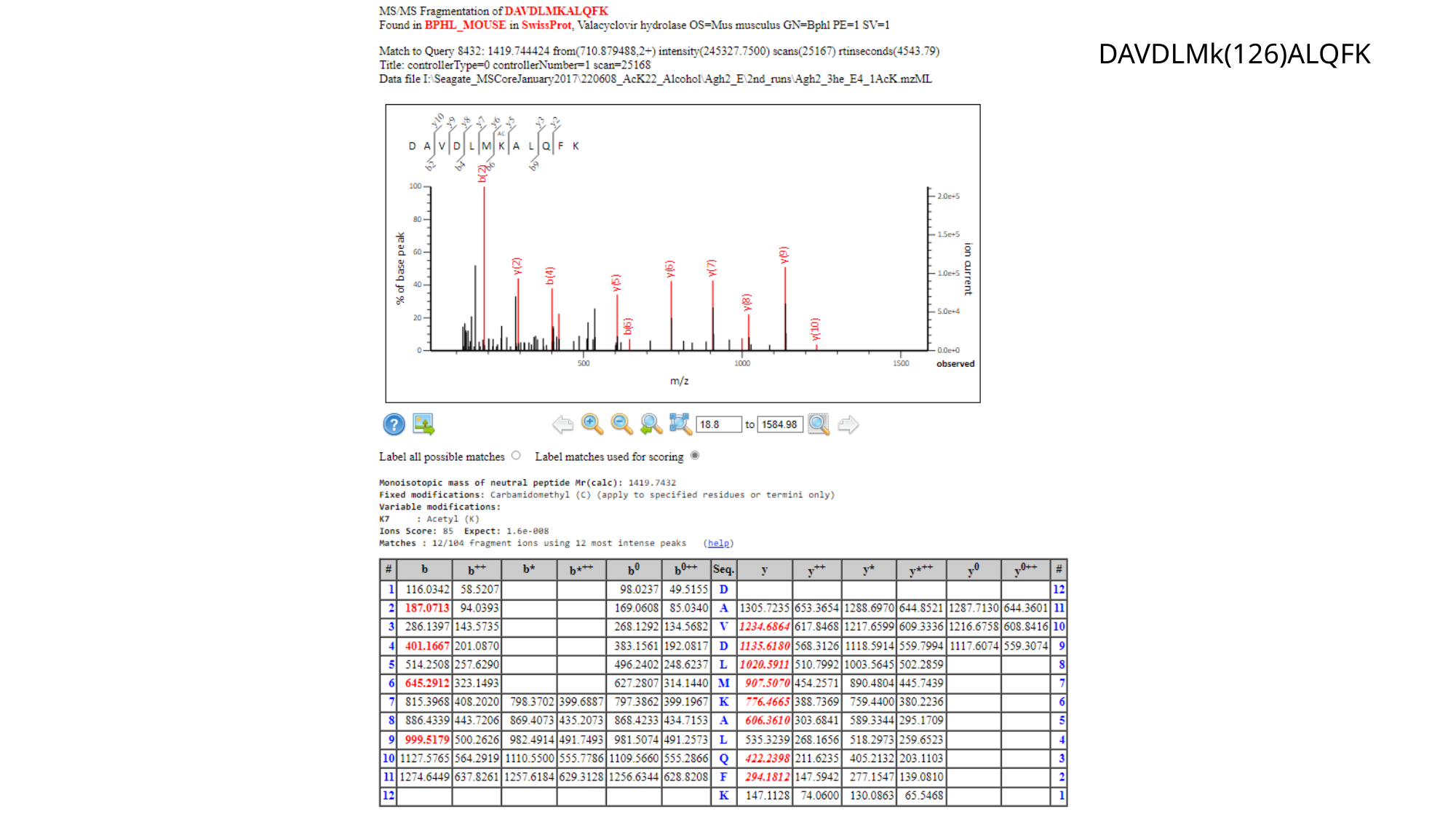

DAVDLMk(126)ALQFK

## Slide 33
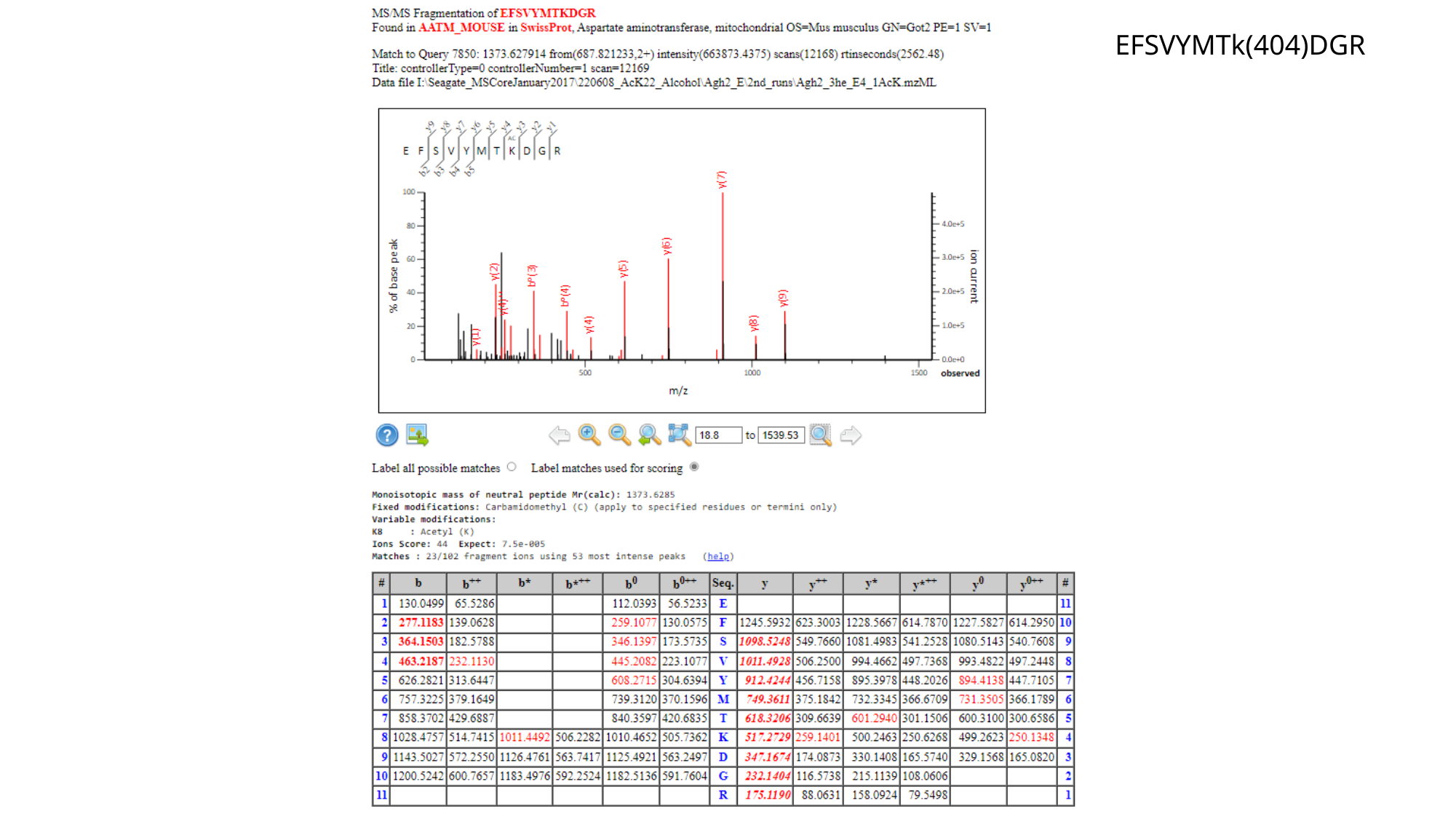

EFSVYMTk(404)DGR

## Slide 34
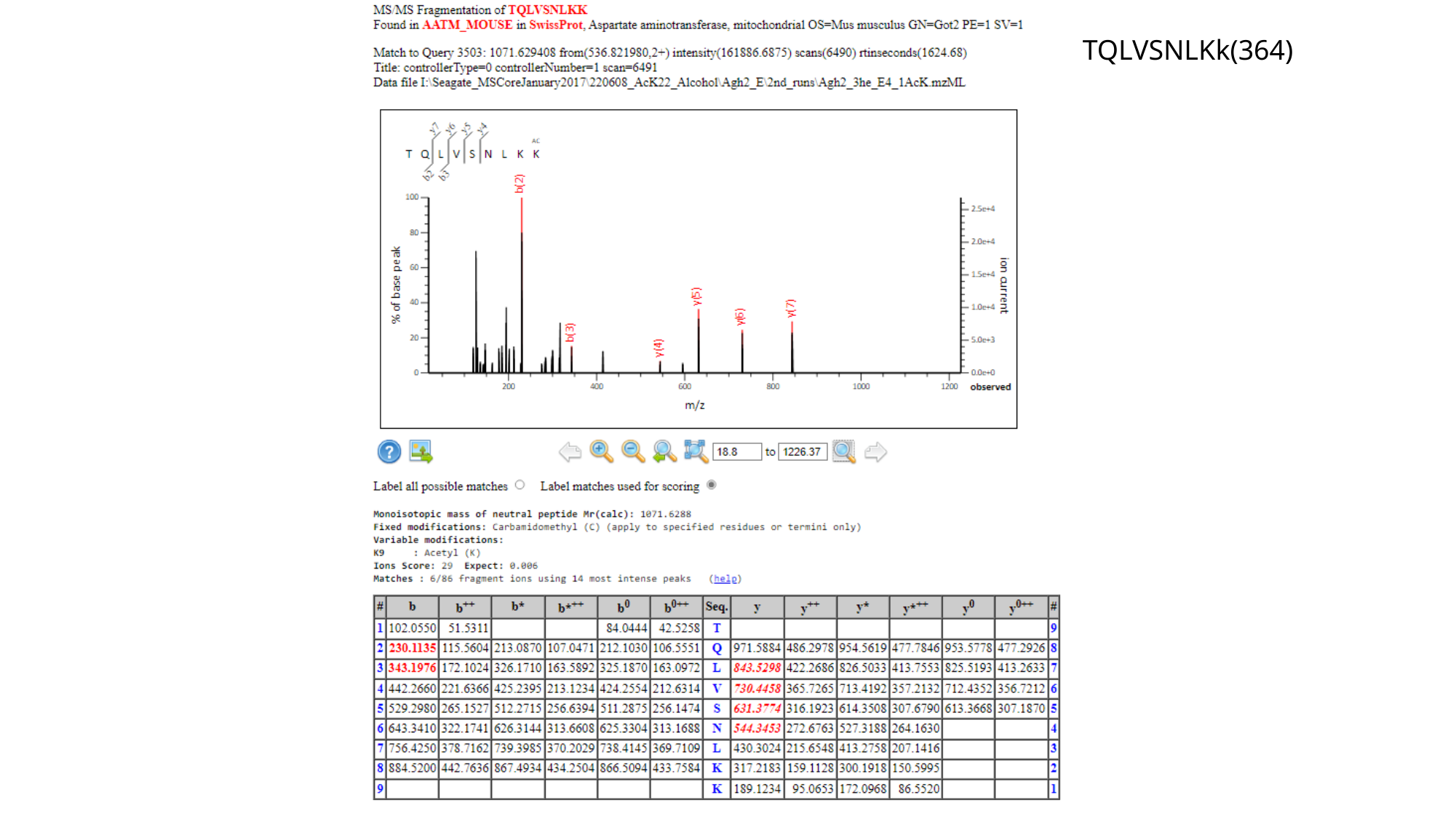

TQLVSNLKk(364)

## Slide 35
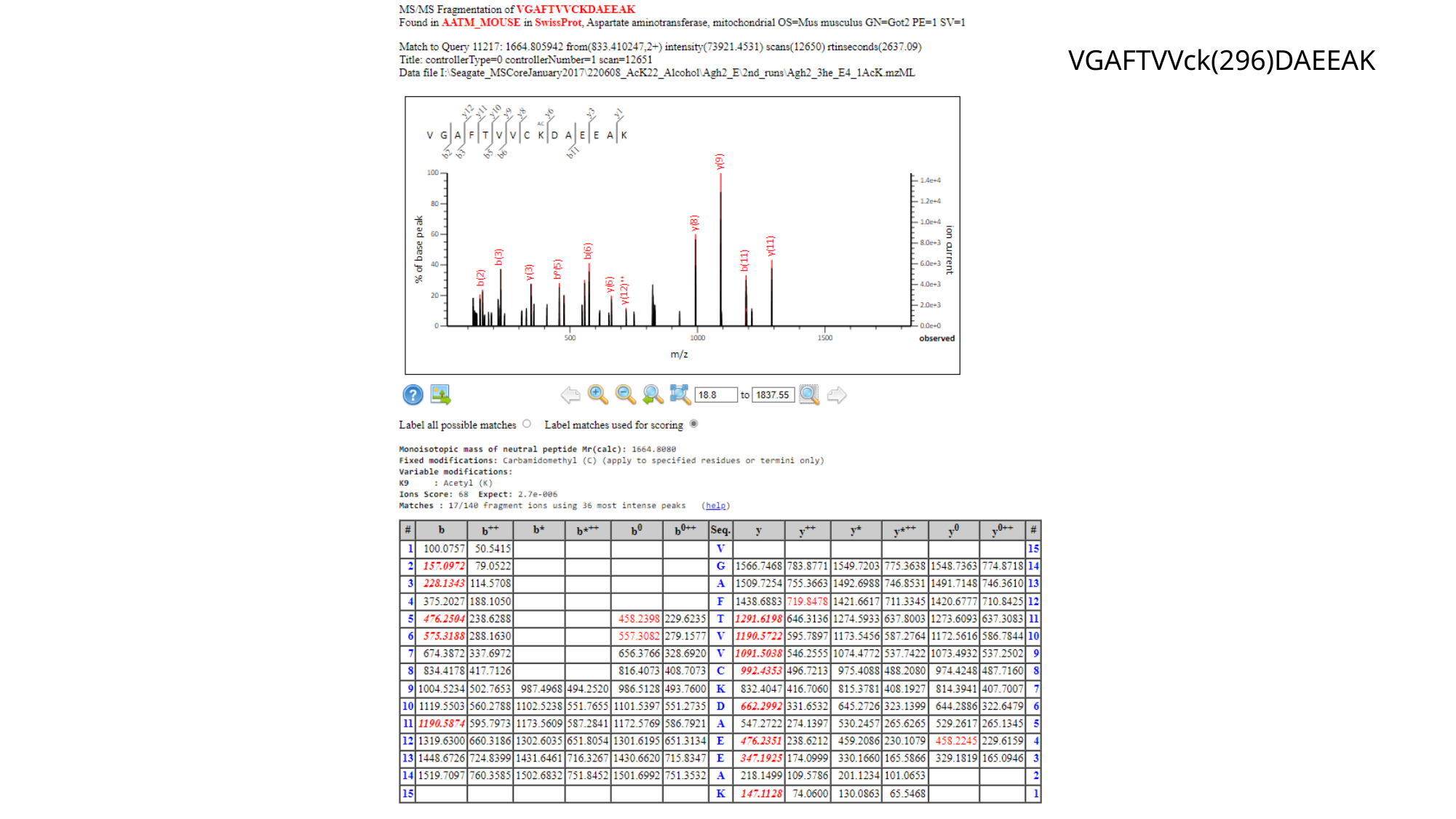

VGAFTVVck(296)DAEEAK

## Slide 36
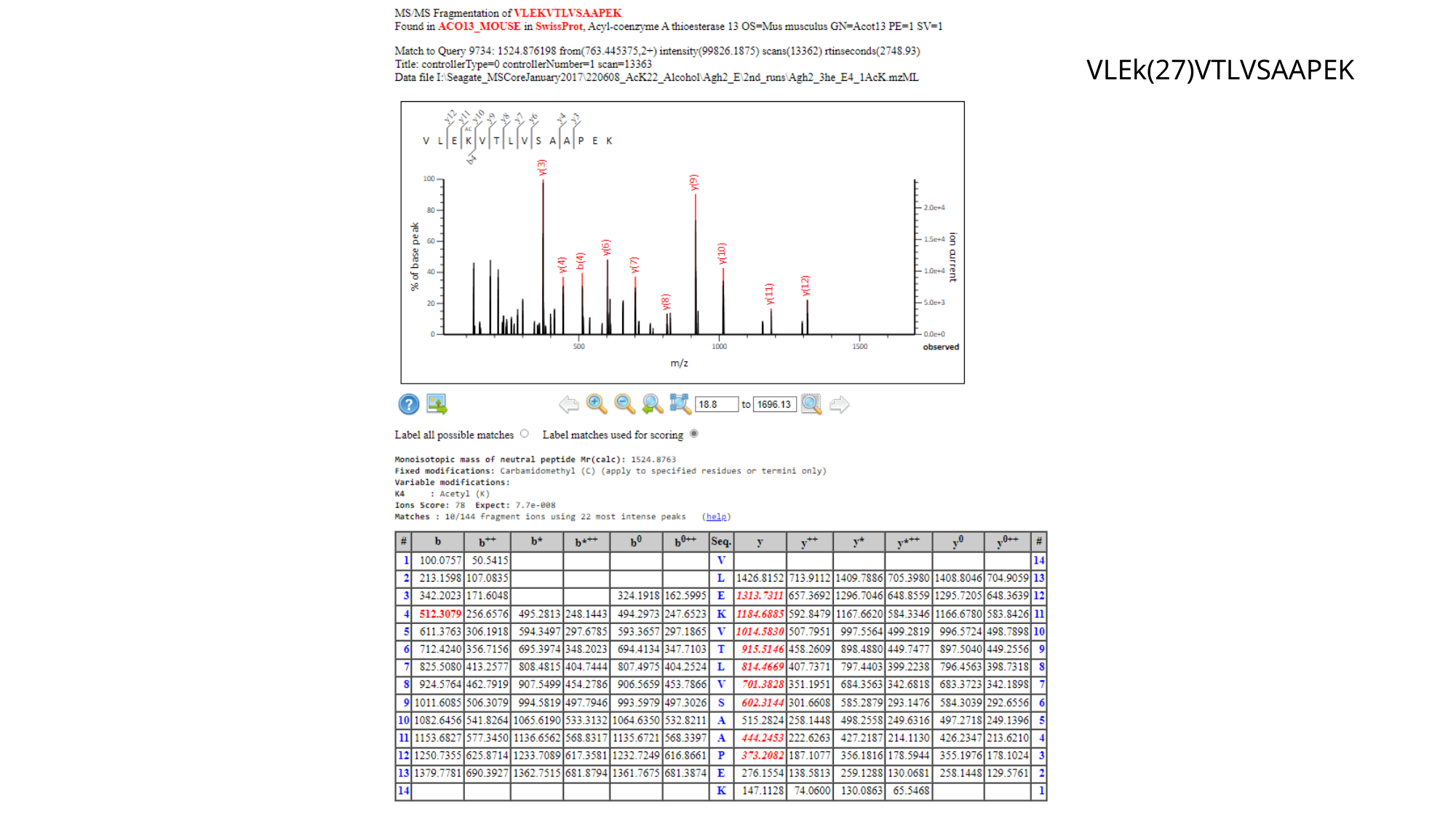

VLEk(27)VTLVSAAPEK

## Slide 37
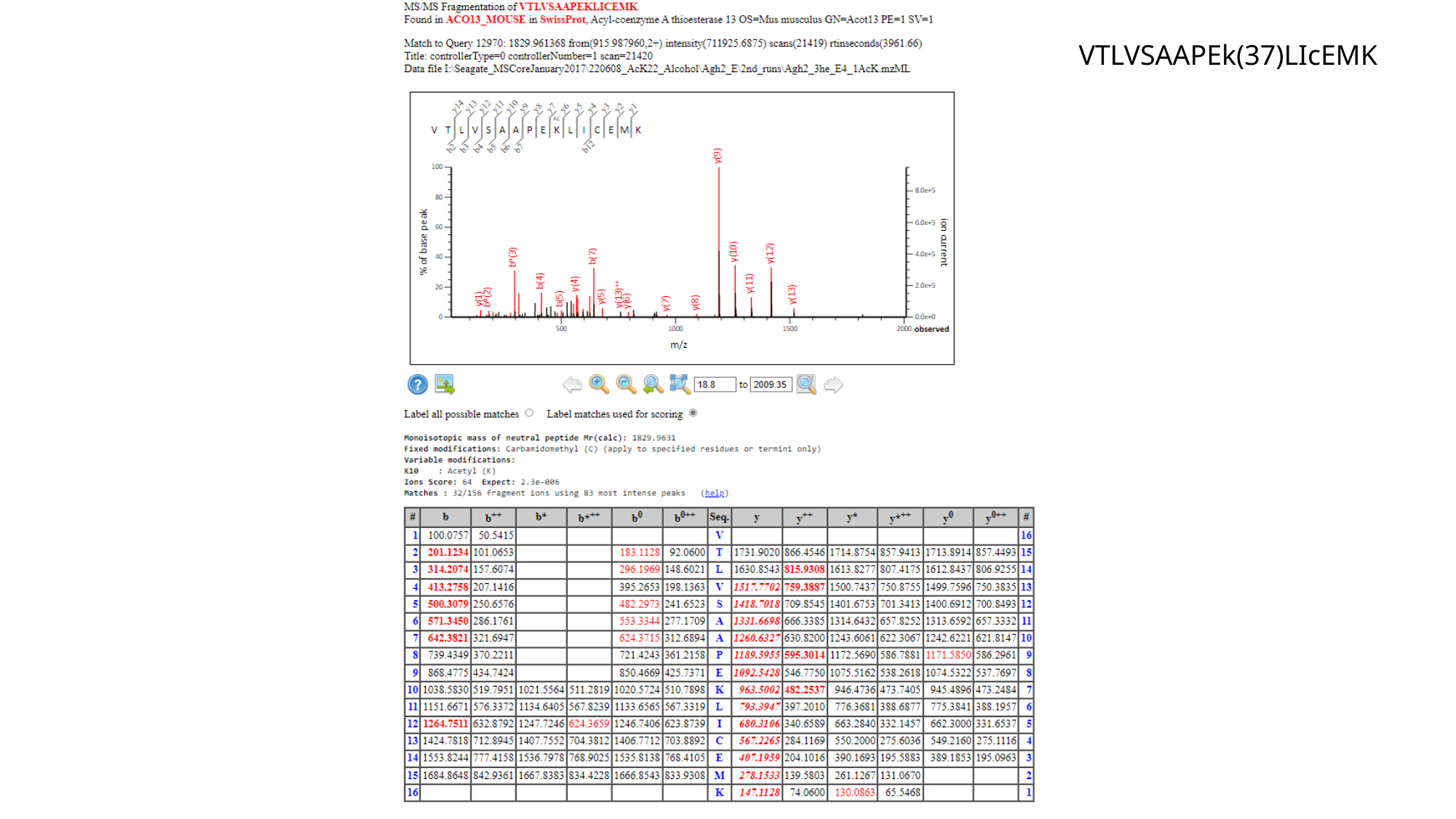

VTLVSAAPEk(37)LIcEMK

## Slide 38
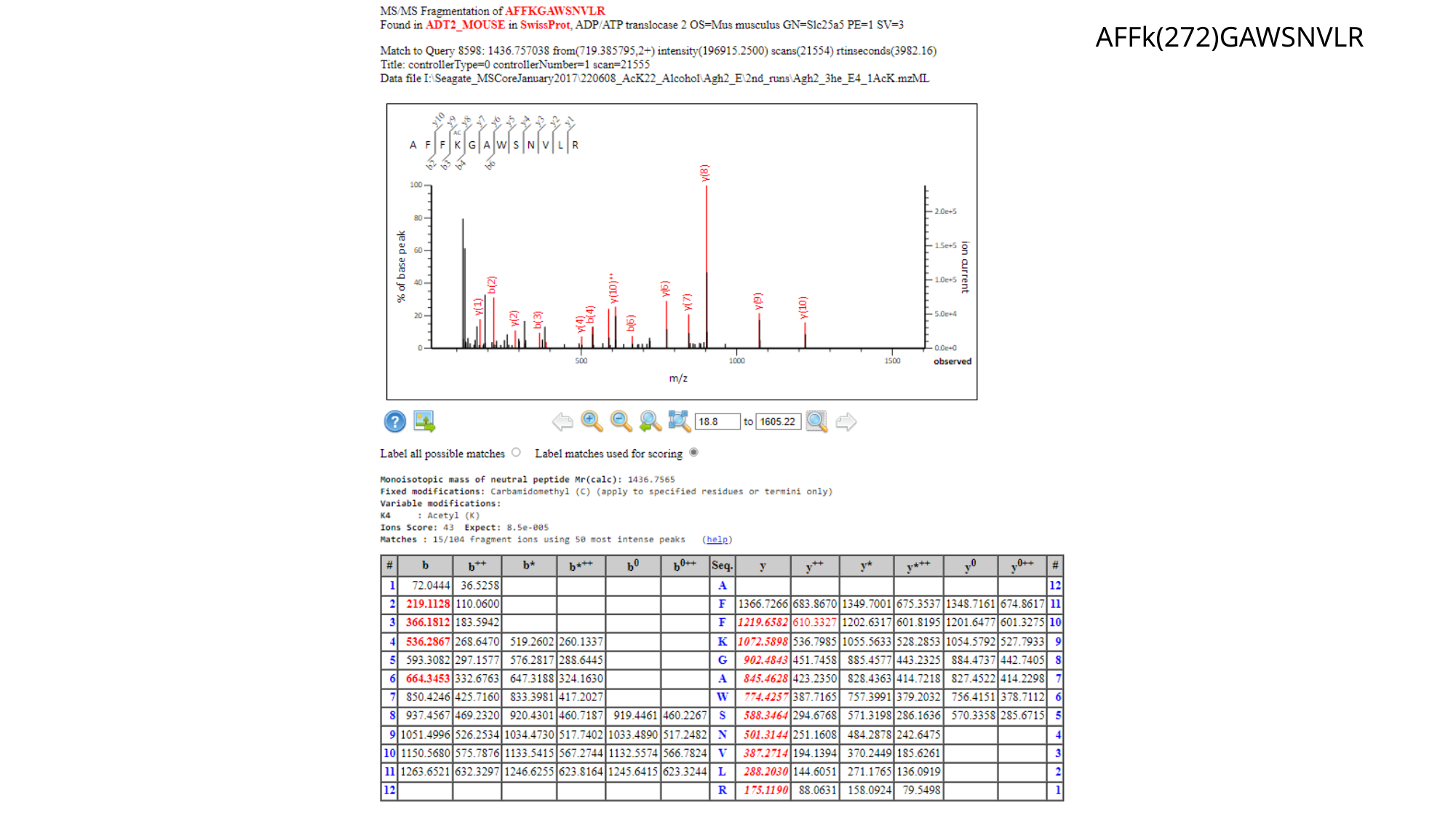

AFFk(272)GAWSNVLR

## Slide 39
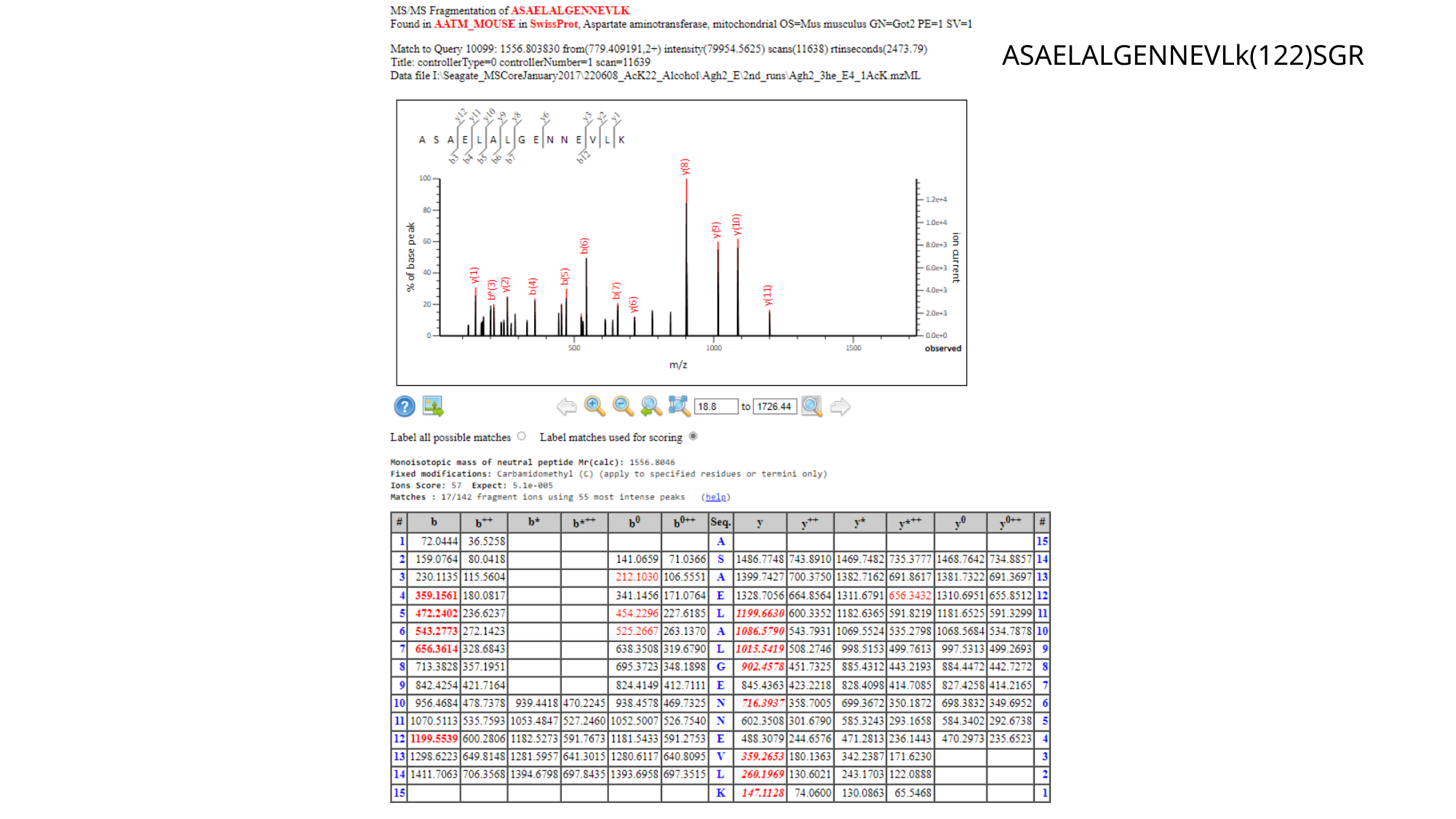

ASAELALGENNEVLk(122)SGR

## Slide 40
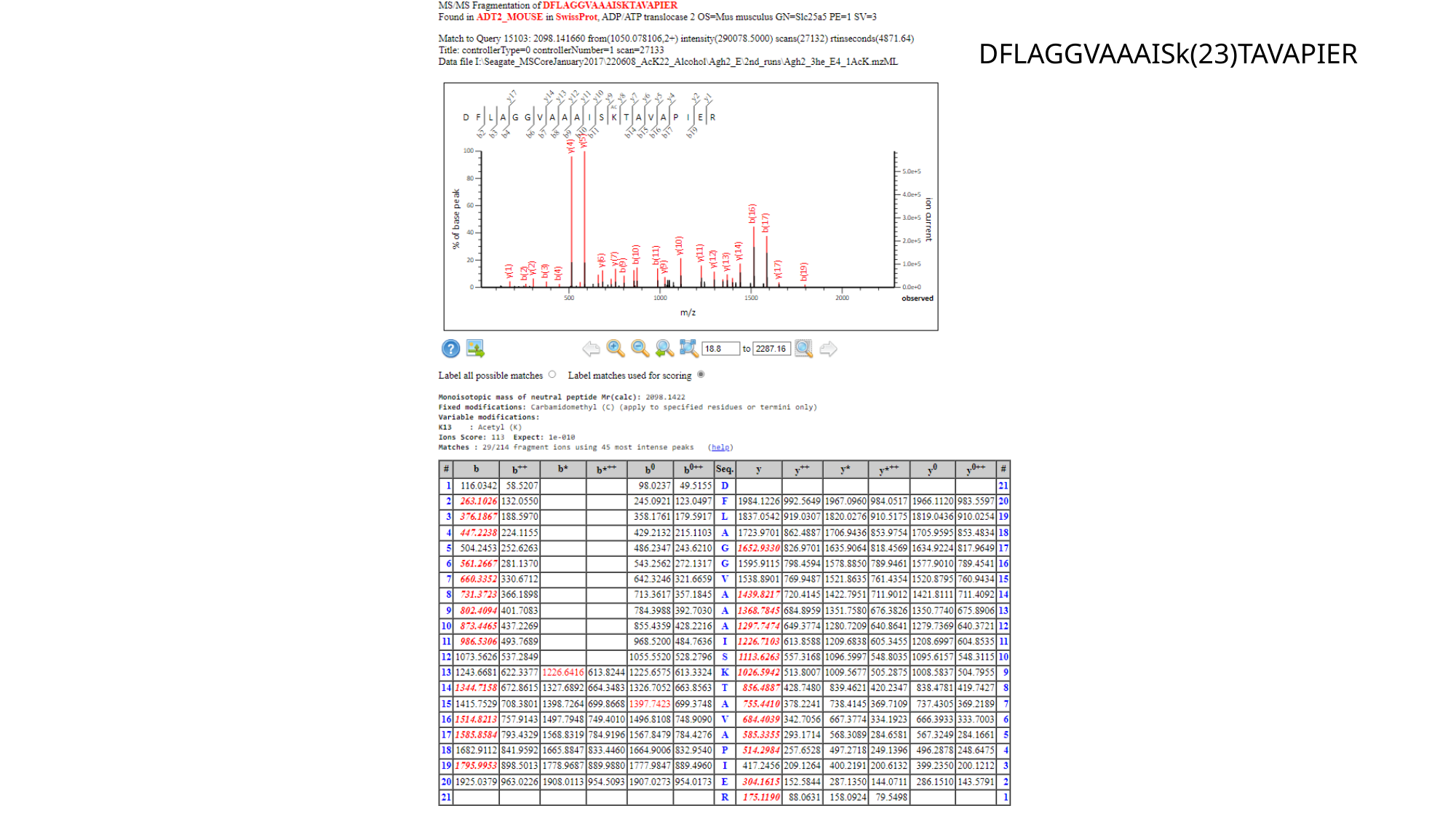

DFLAGGVAAAISk(23)TAVAPIER

## Slide 41
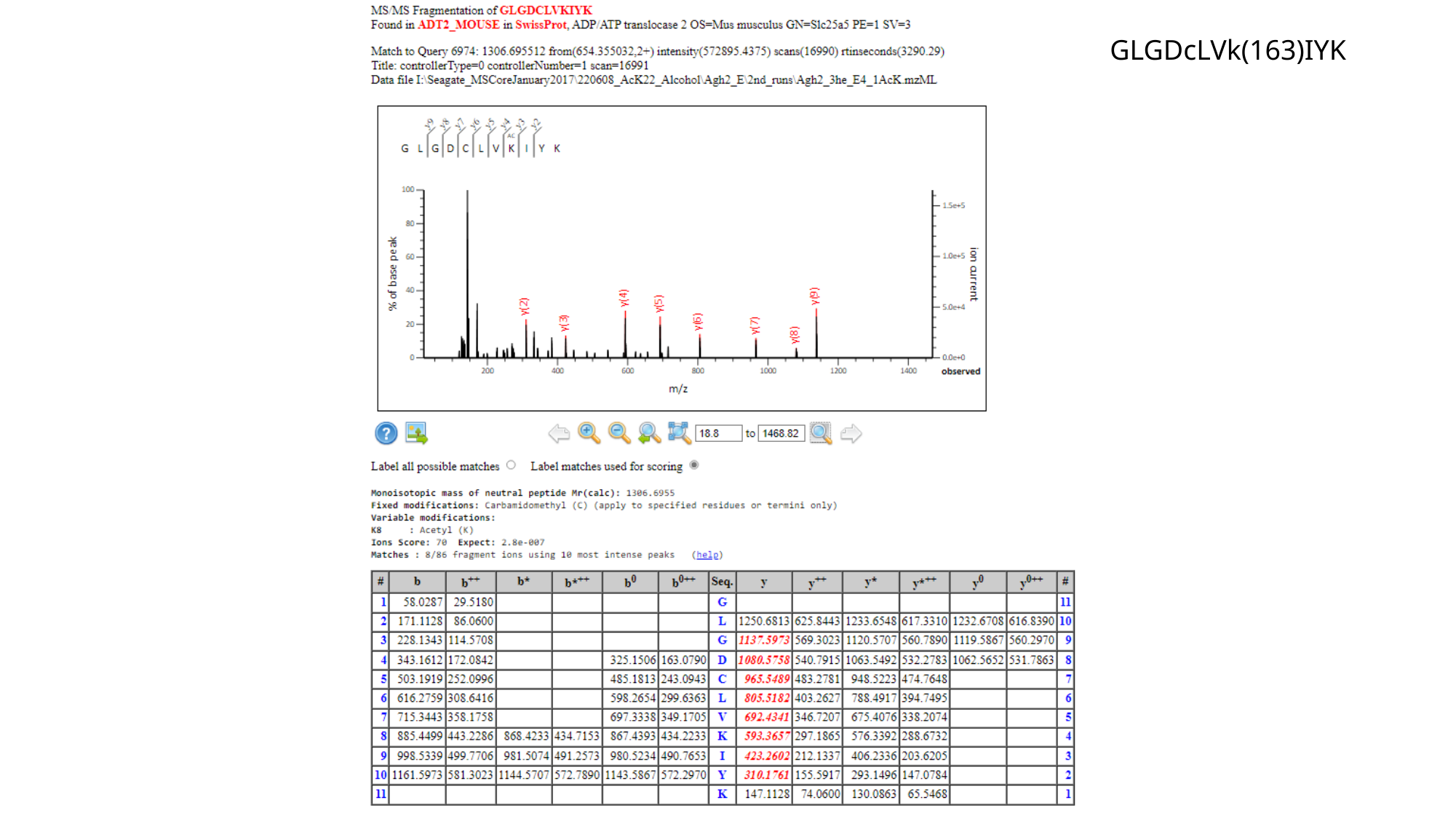

GLGDcLVk(163)IYK

## Slide 42
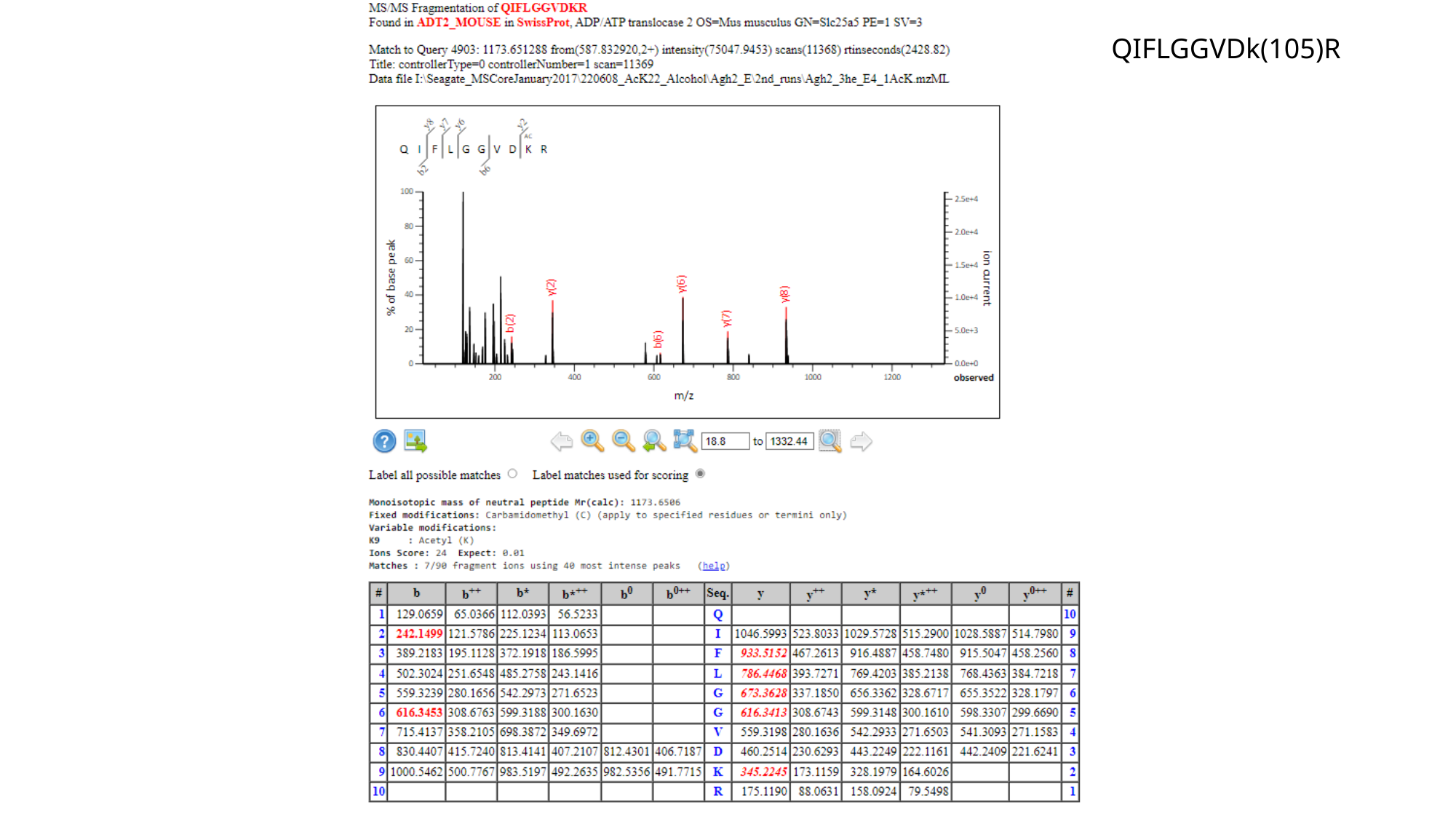

QIFLGGVDk(105)R

## Slide 43
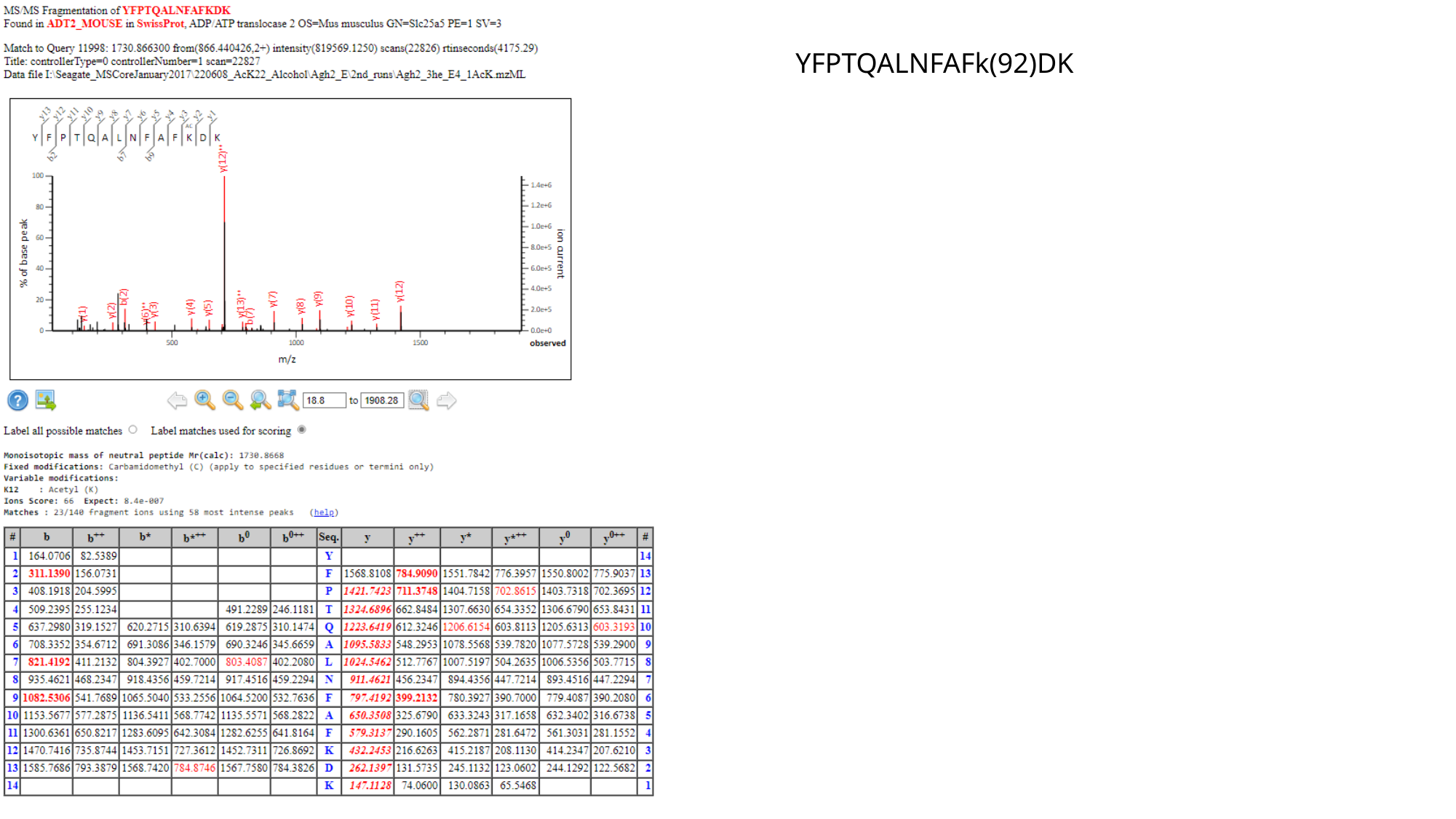

YFPTQALNFAFk(92)DK

## Slide 44
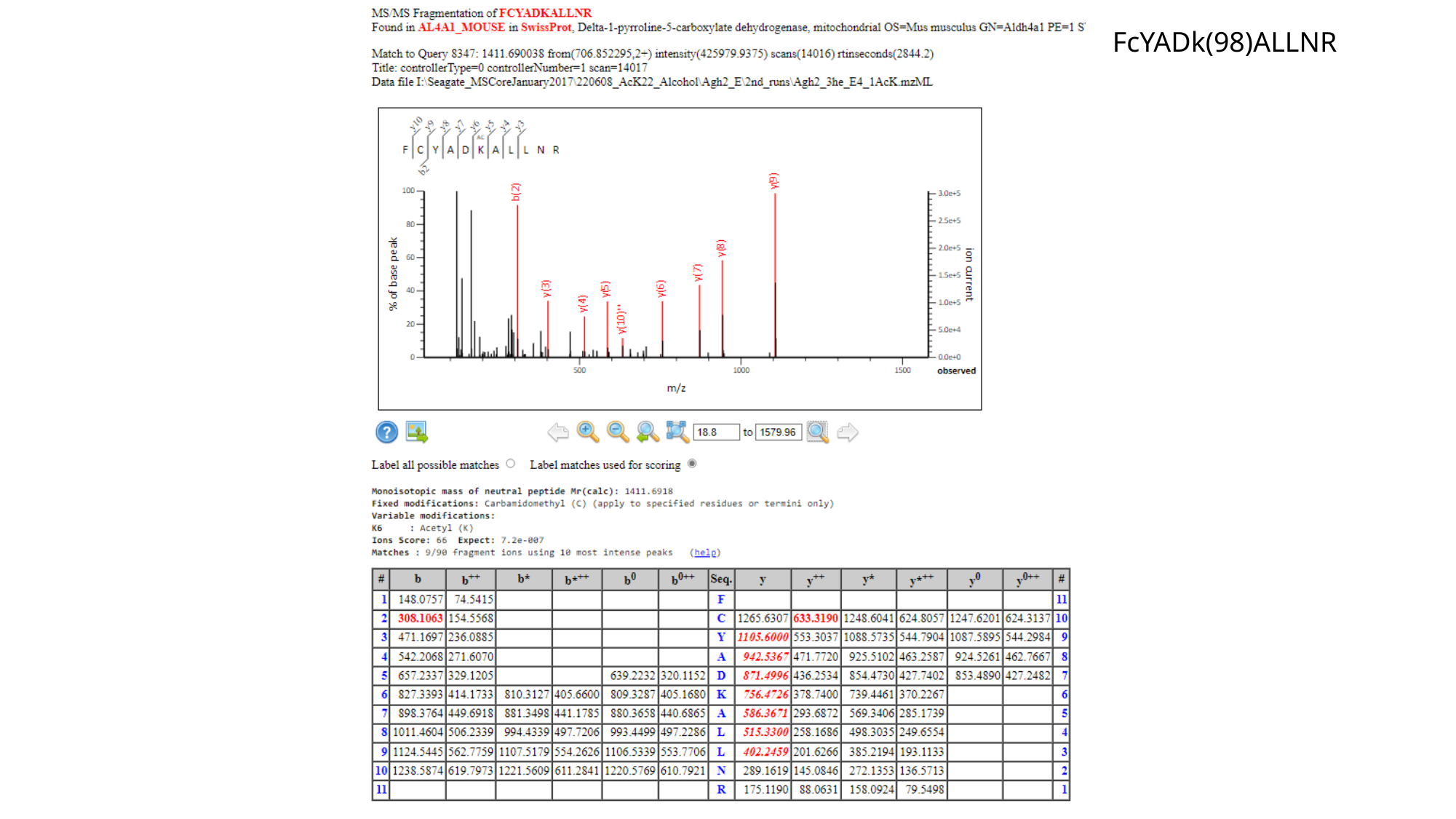

FcYADk(98)ALLNR

## Slide 45
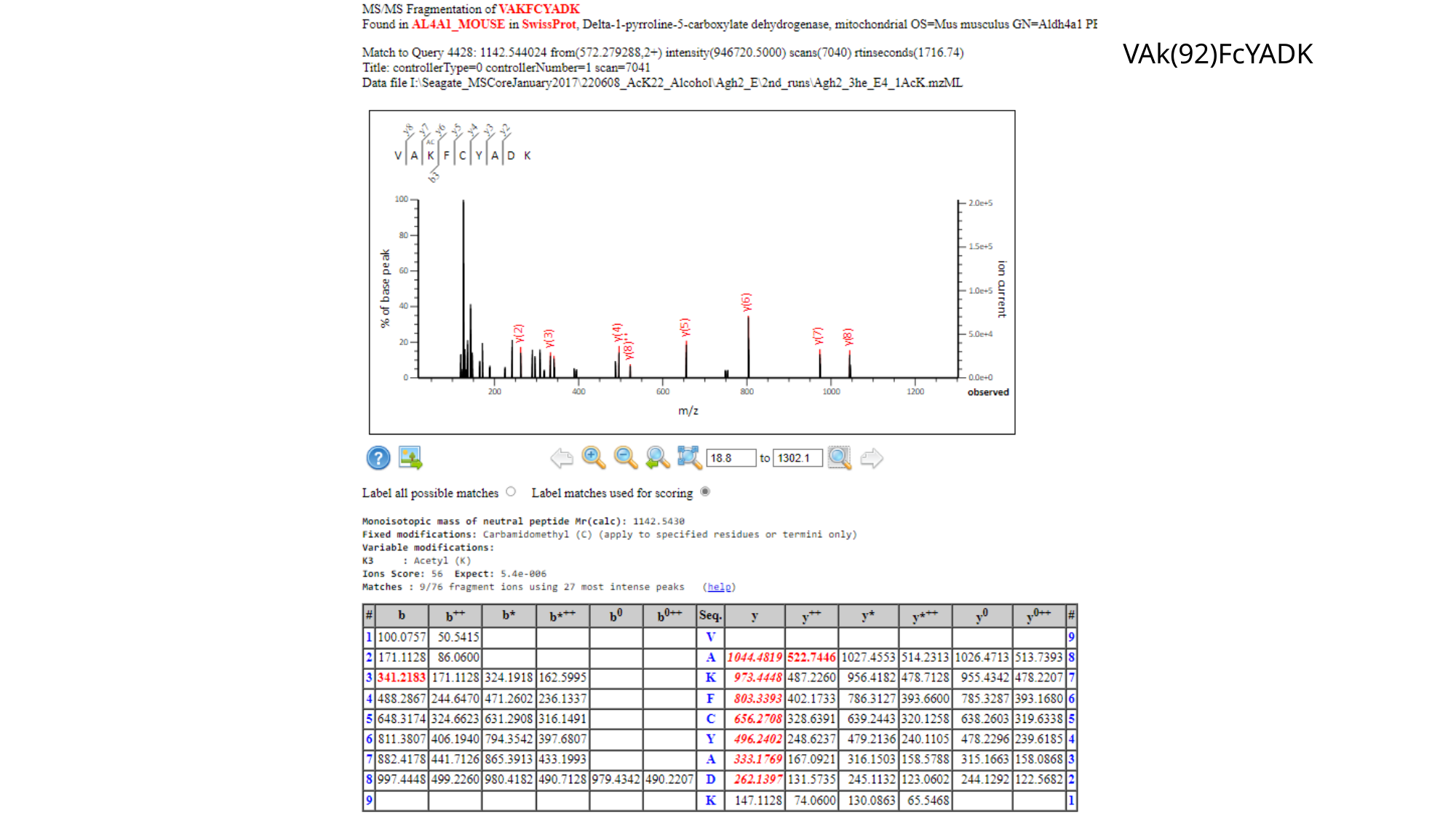

VAk(92)FcYADK

## Slide 46
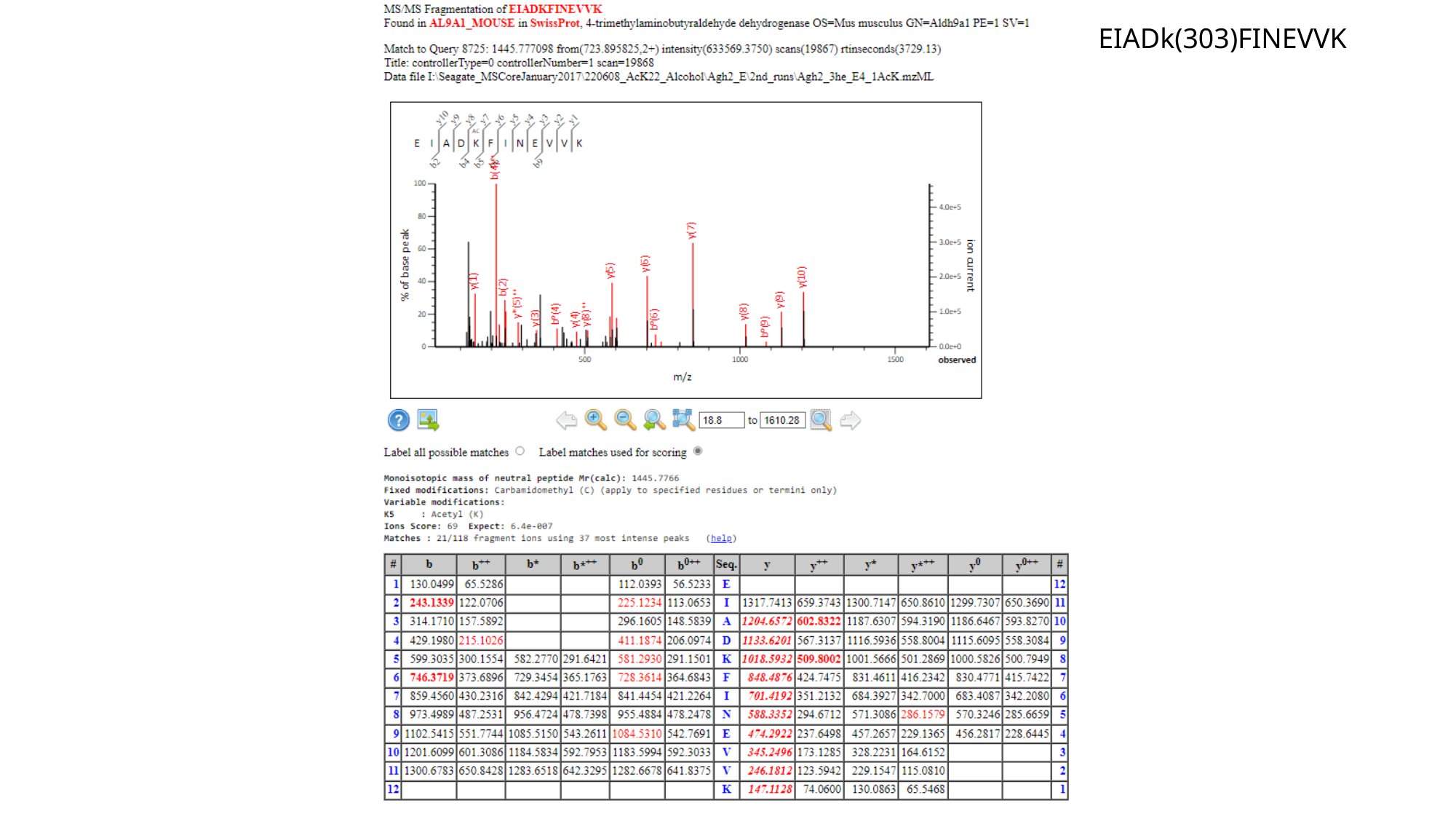

EIADk(303)FINEVVK

## Slide 47
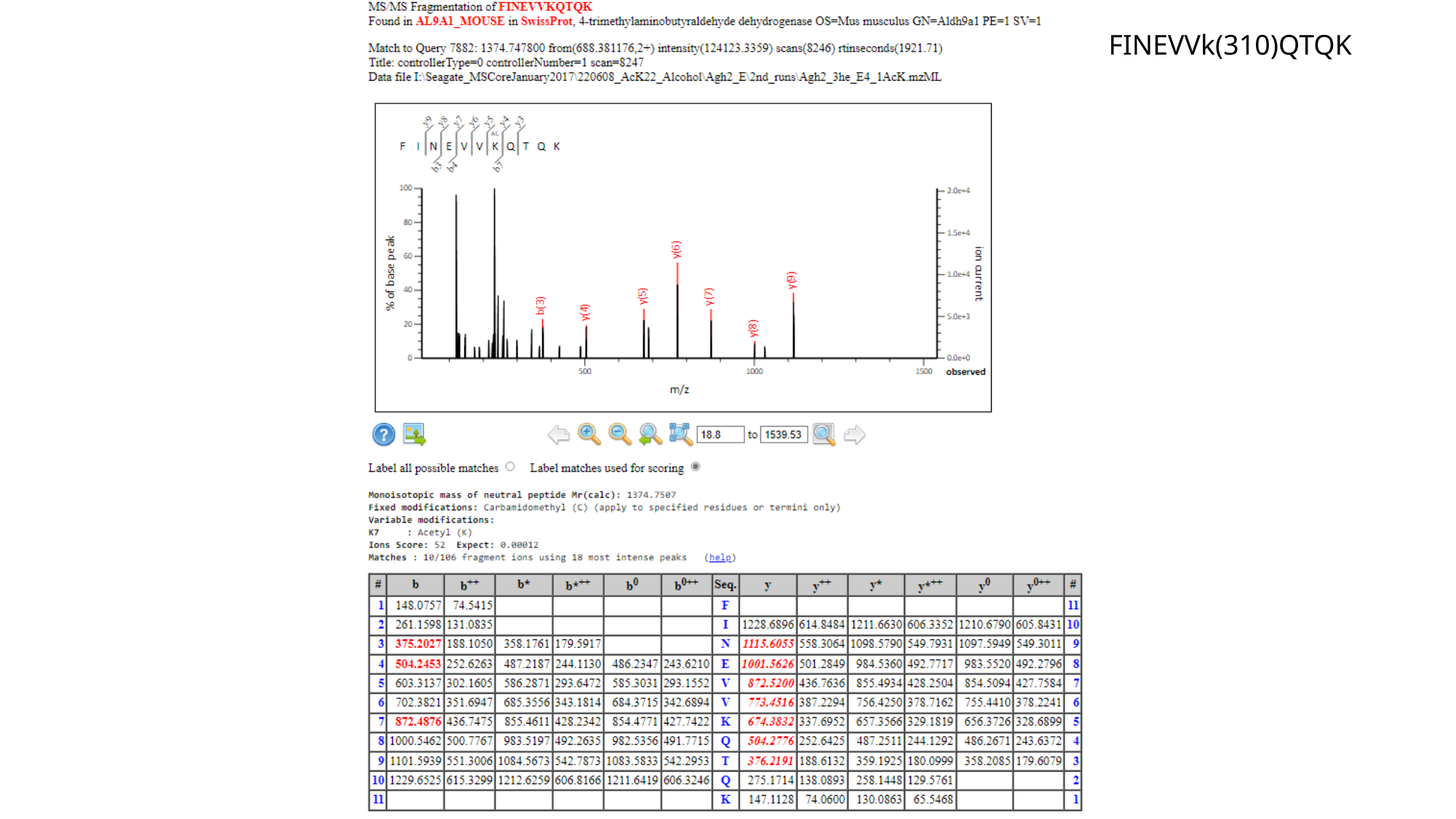

FINEVVk(310)QTQK

## Slide 48
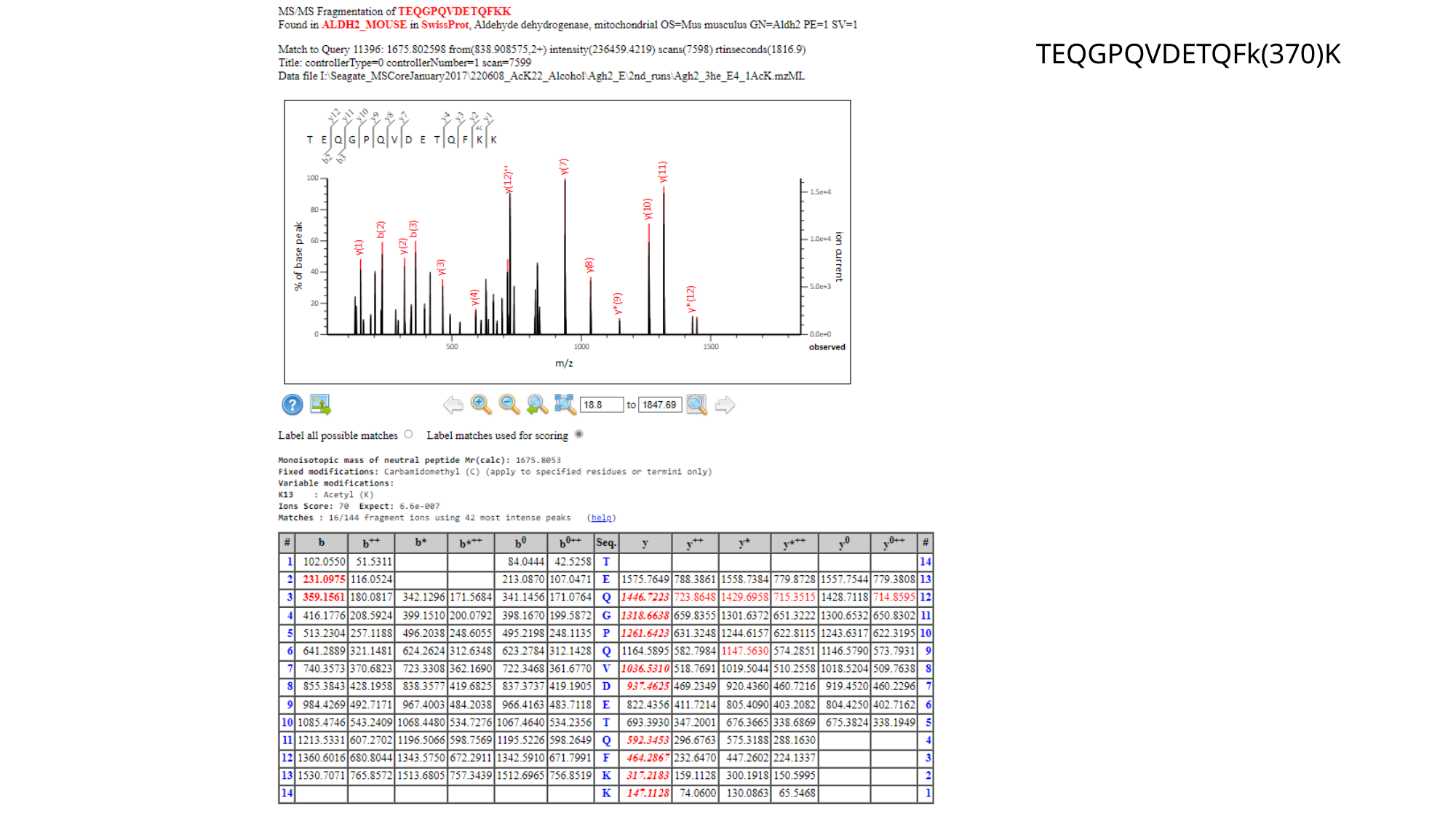

TEQGPQVDETQFk(370)K

## Slide 49
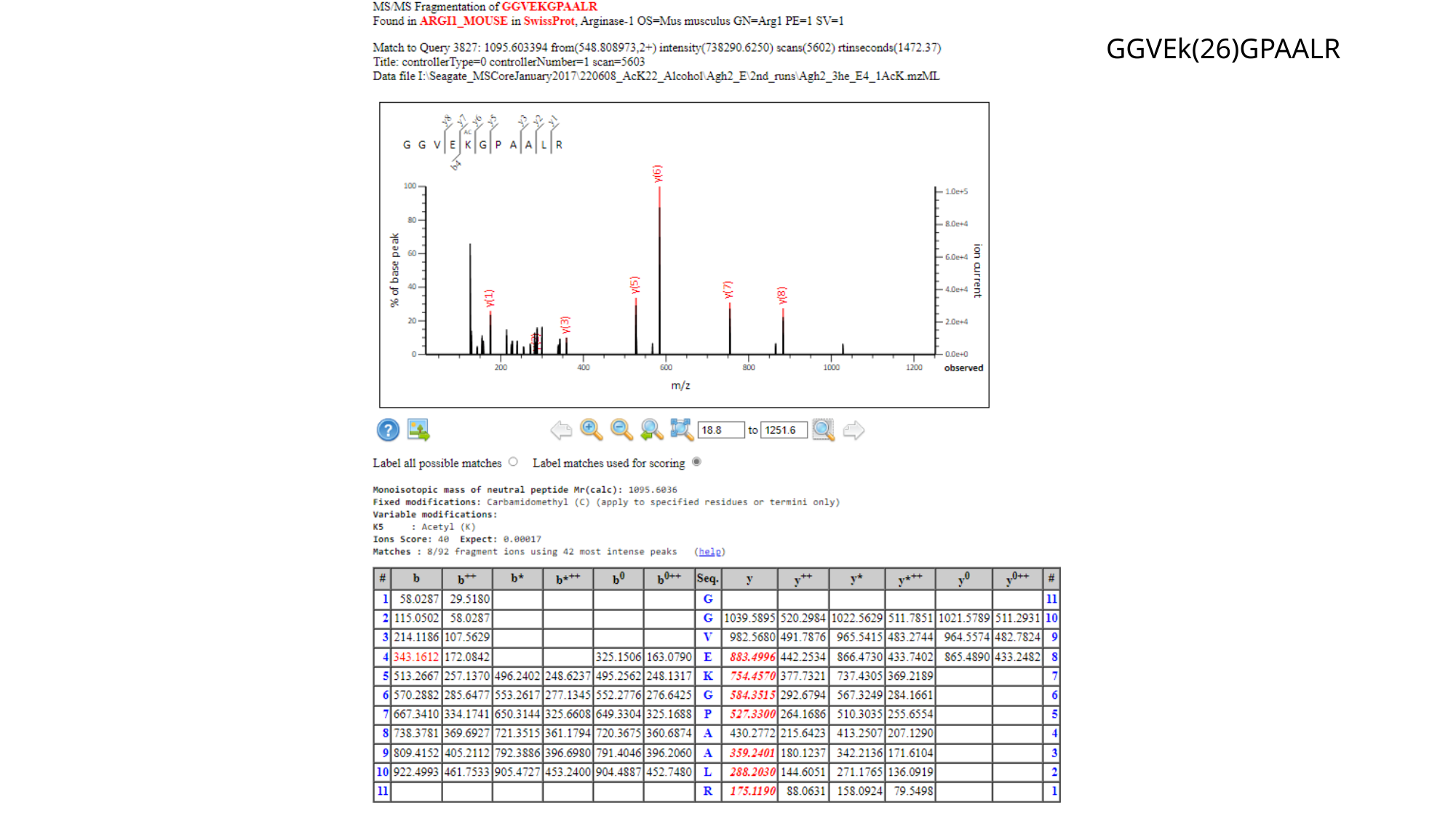

GGVEk(26)GPAALR

## Slide 50
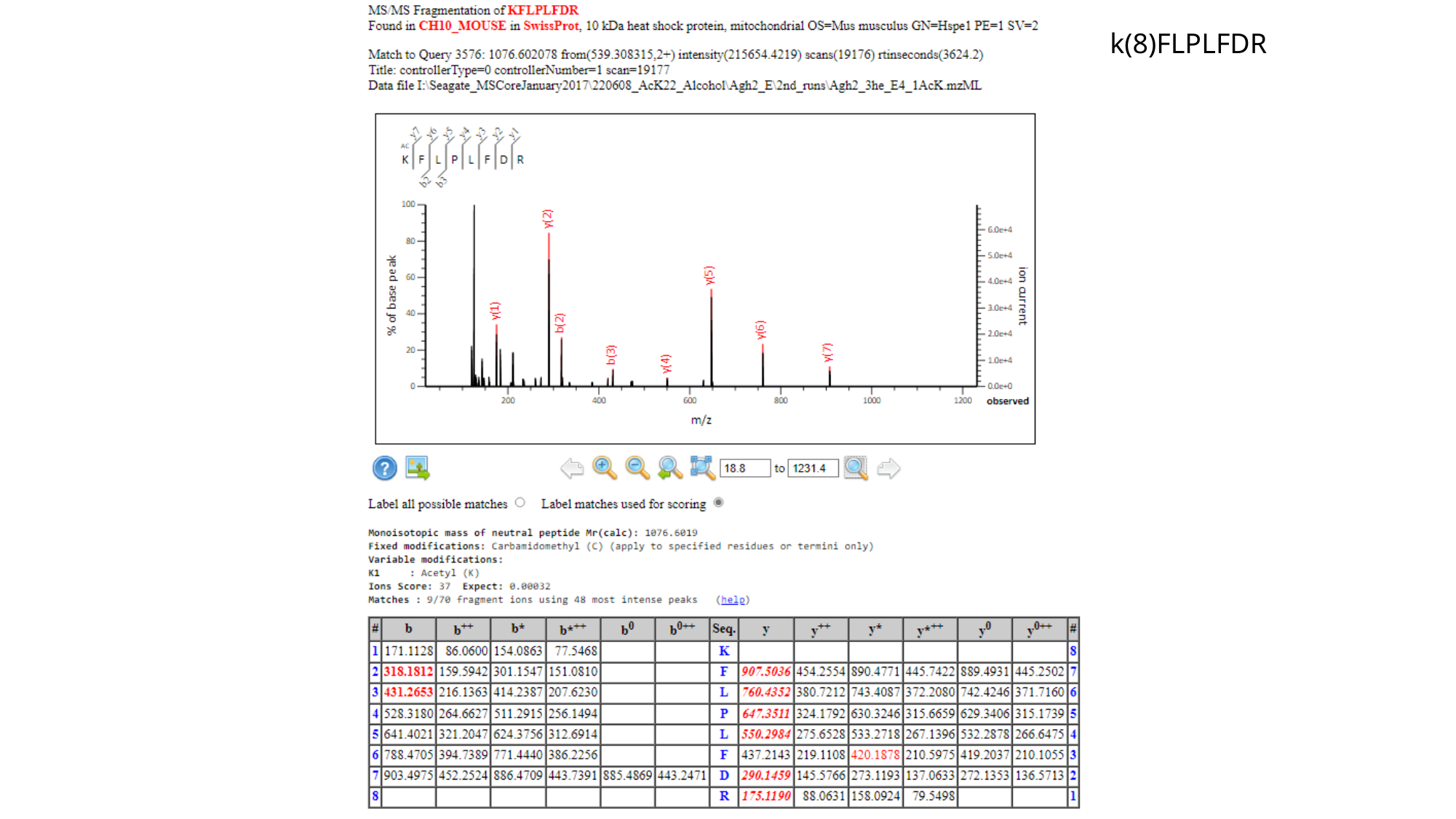

k(8)FLPLFDR

## Slide 51
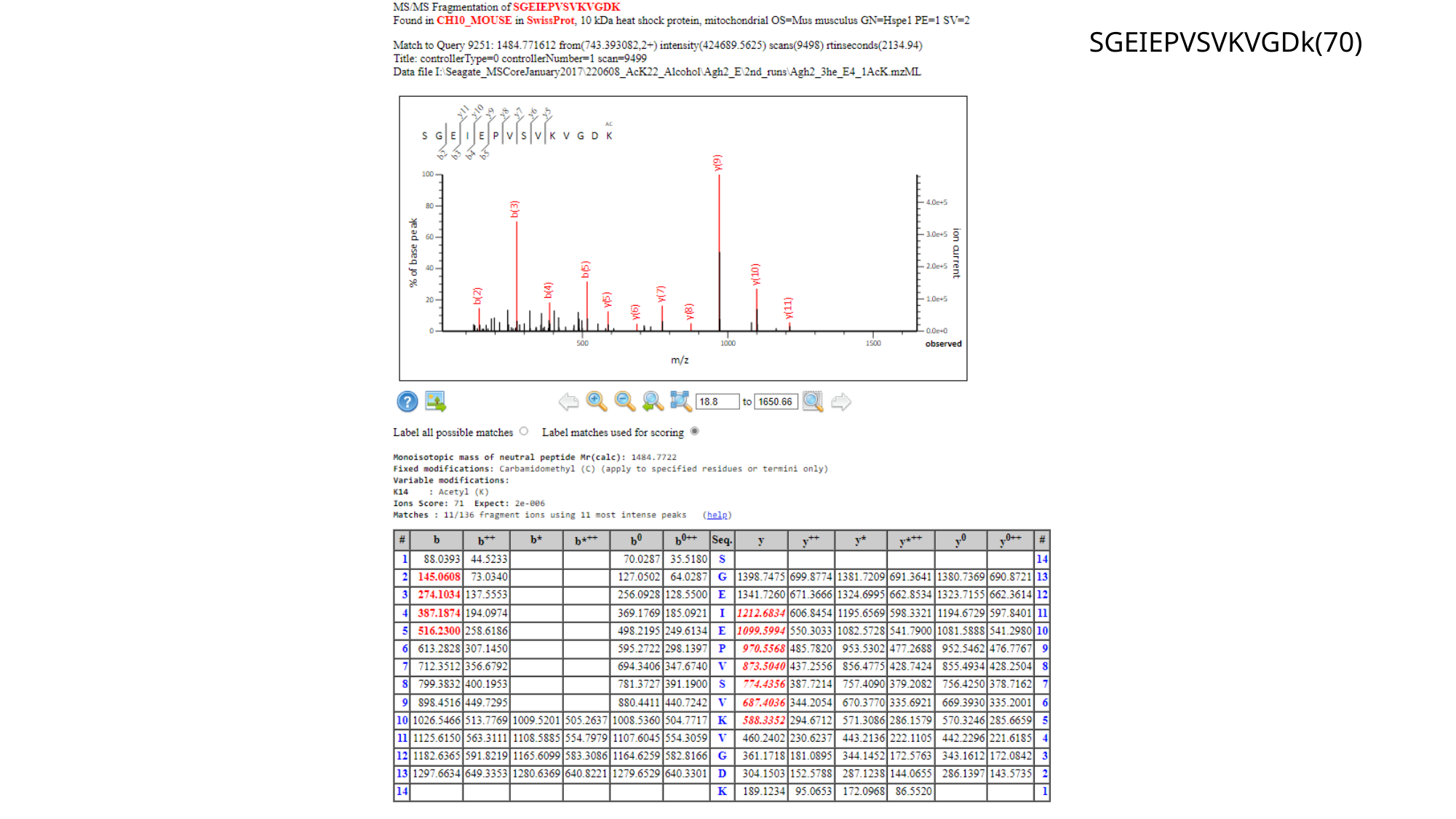

SGEIEPVSVKVGDk(70)

## Slide 52
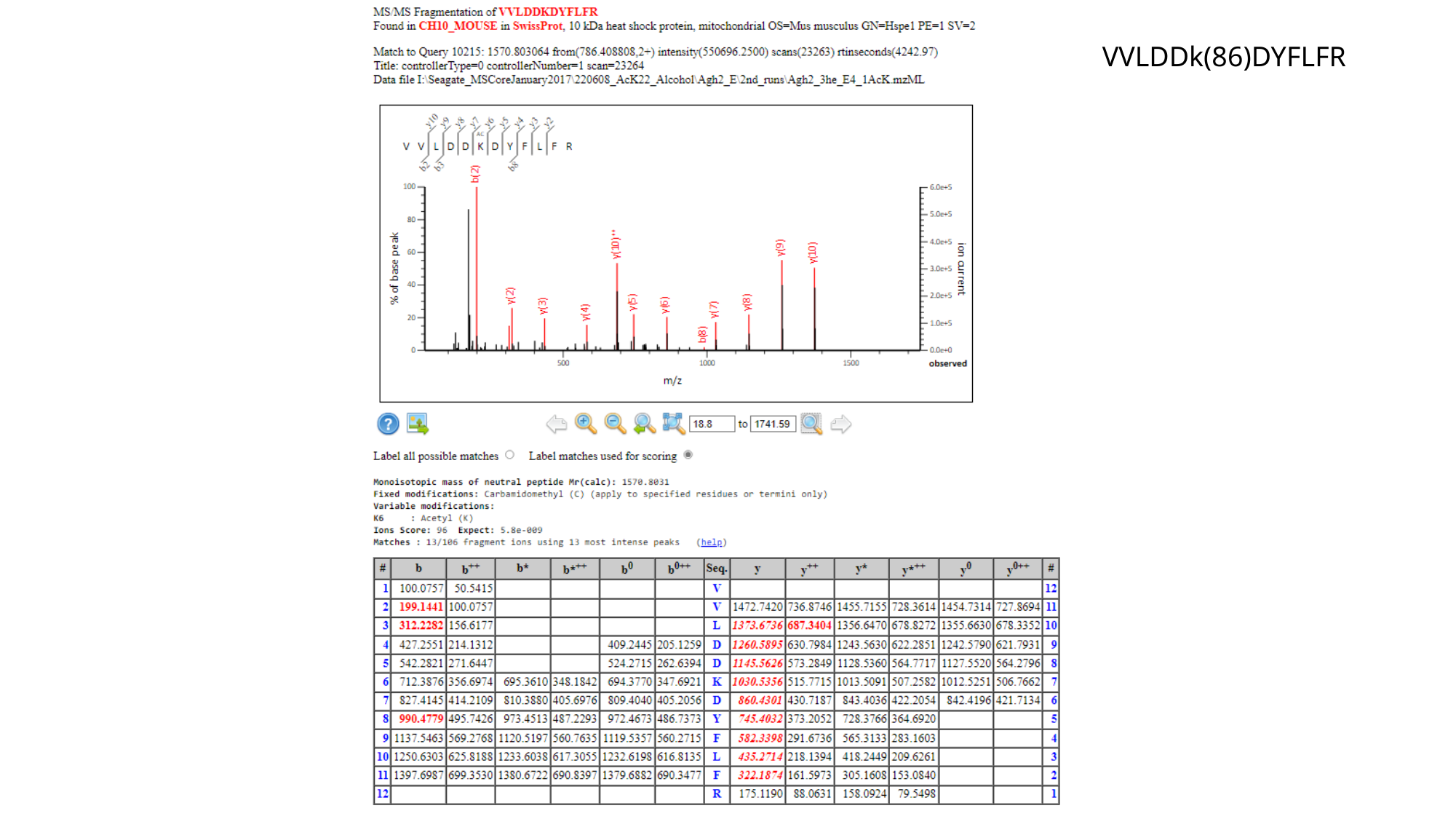

VVLDDk(86)DYFLFR

## Slide 53
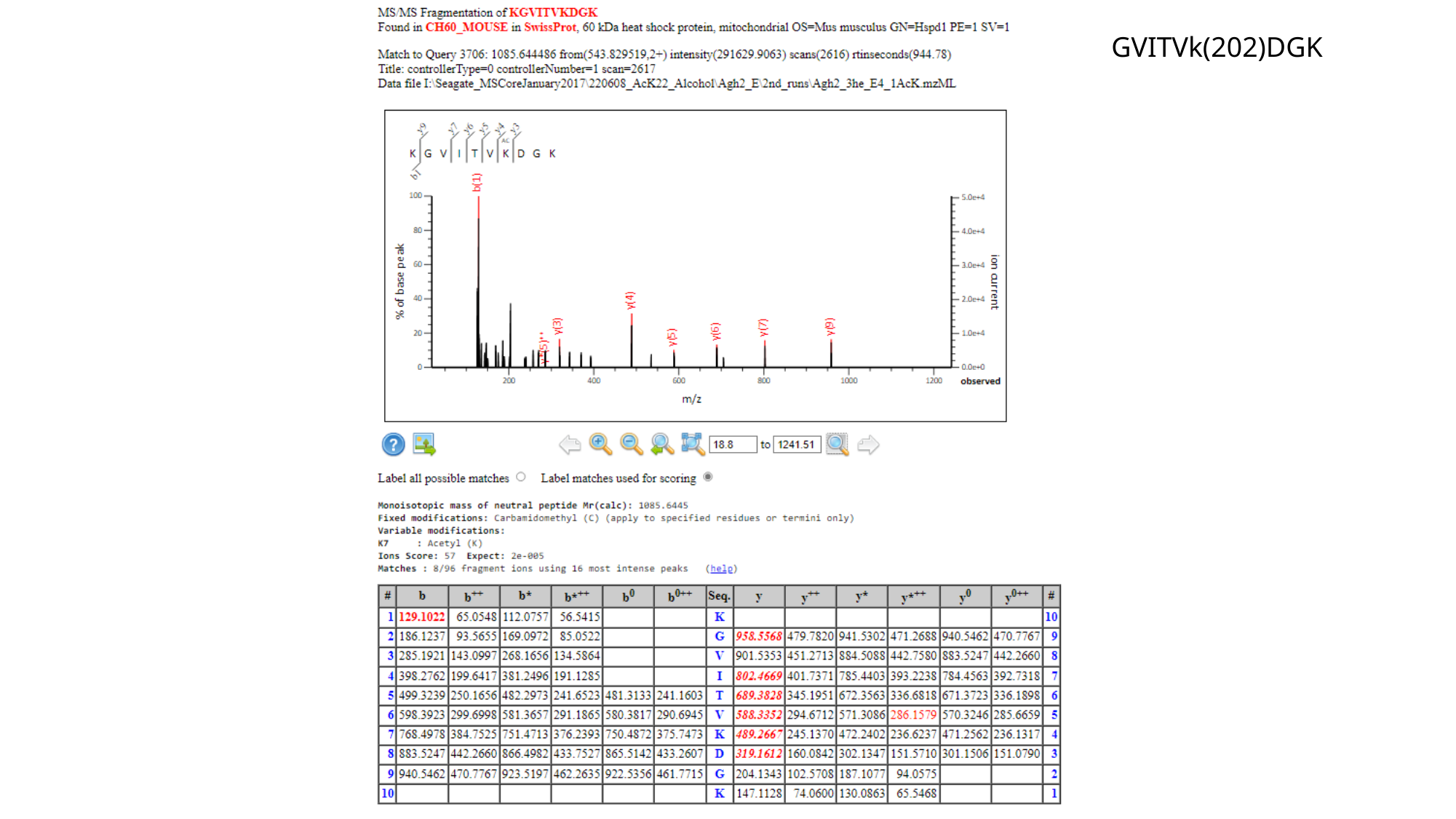

GVITVk(202)DGK

## Slide 54
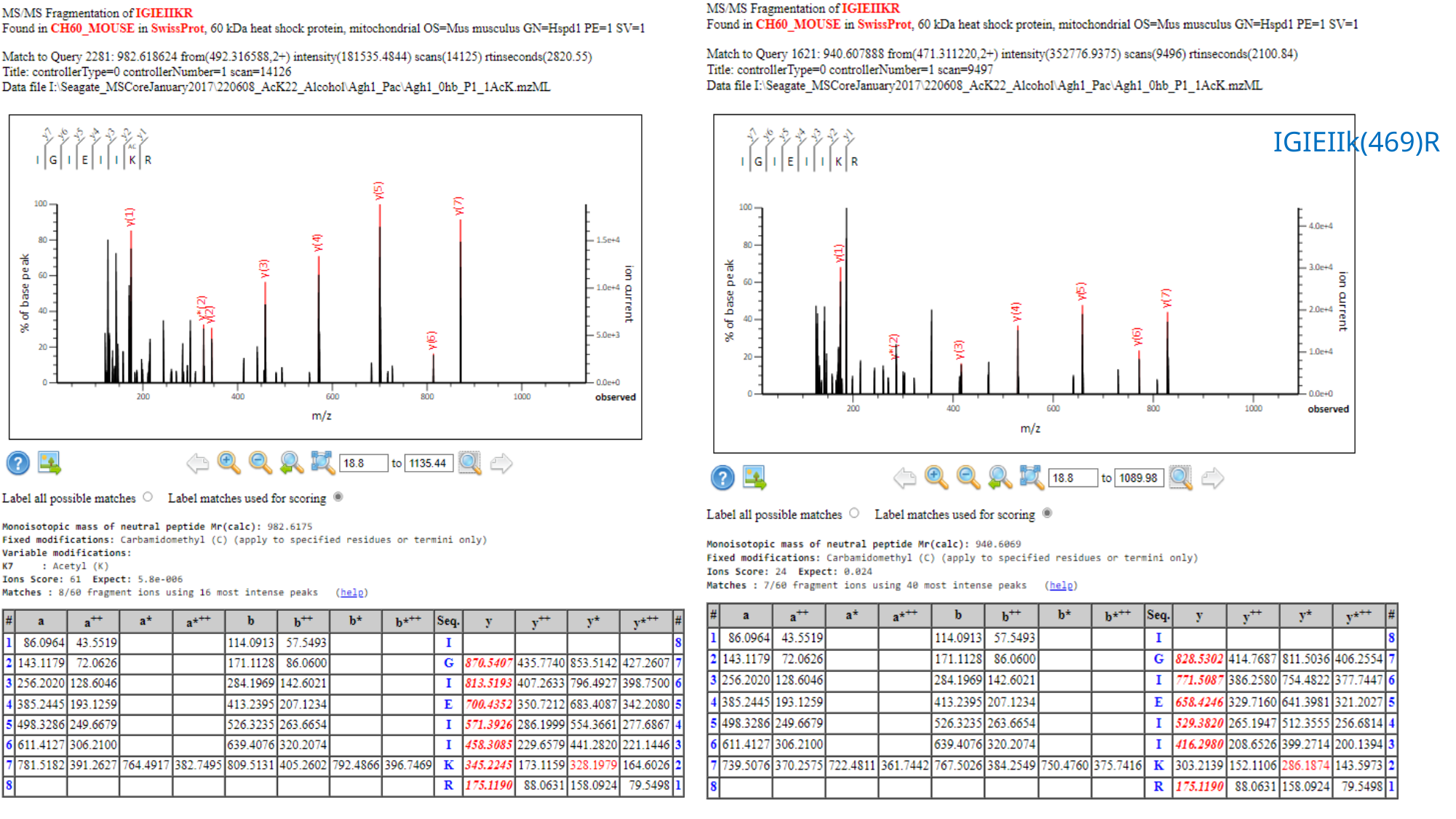

IGIEIIk(469)R

## Slide 55
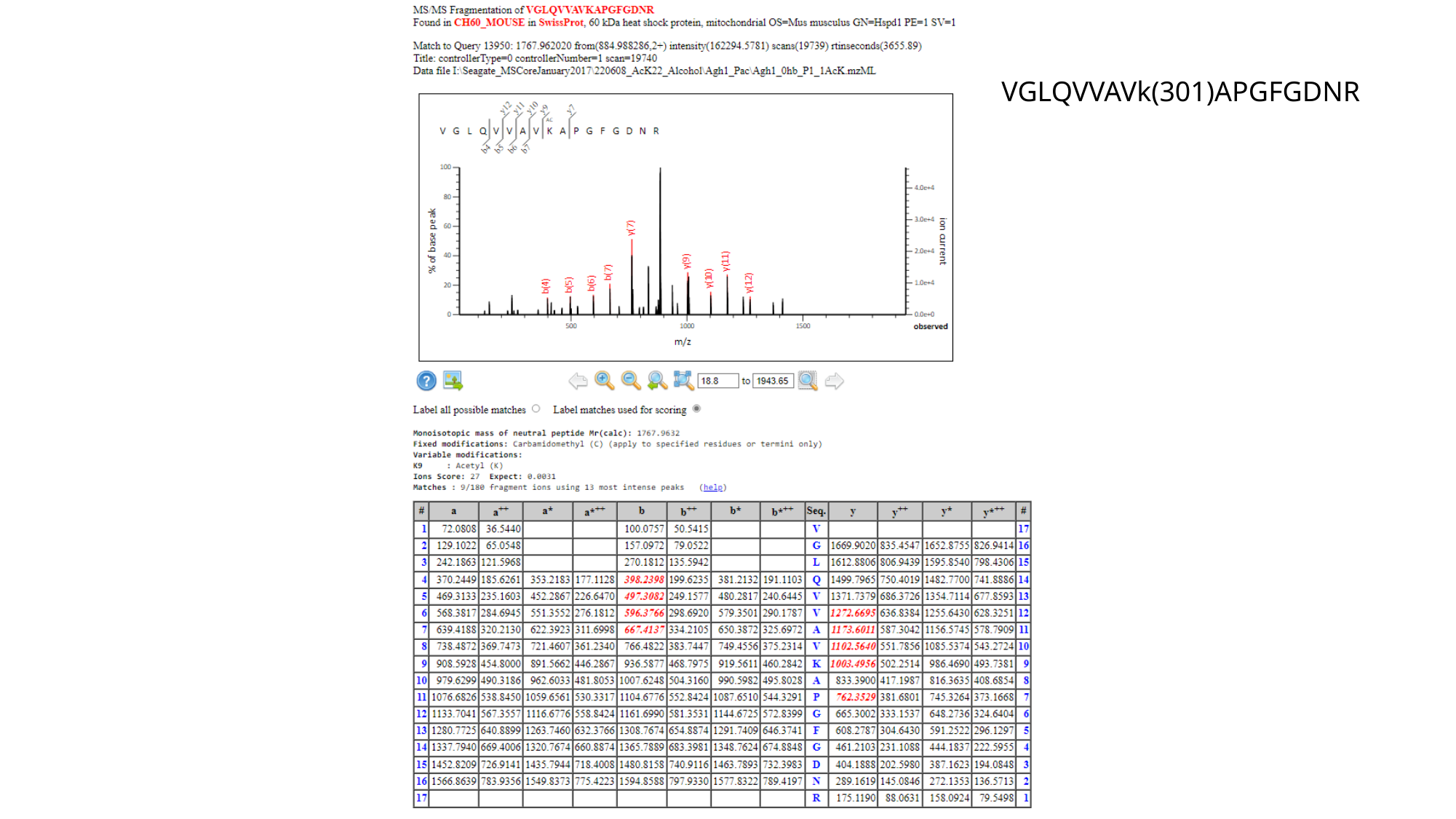

VGLQVVAVk(301)APGFGDNR

## Slide 56
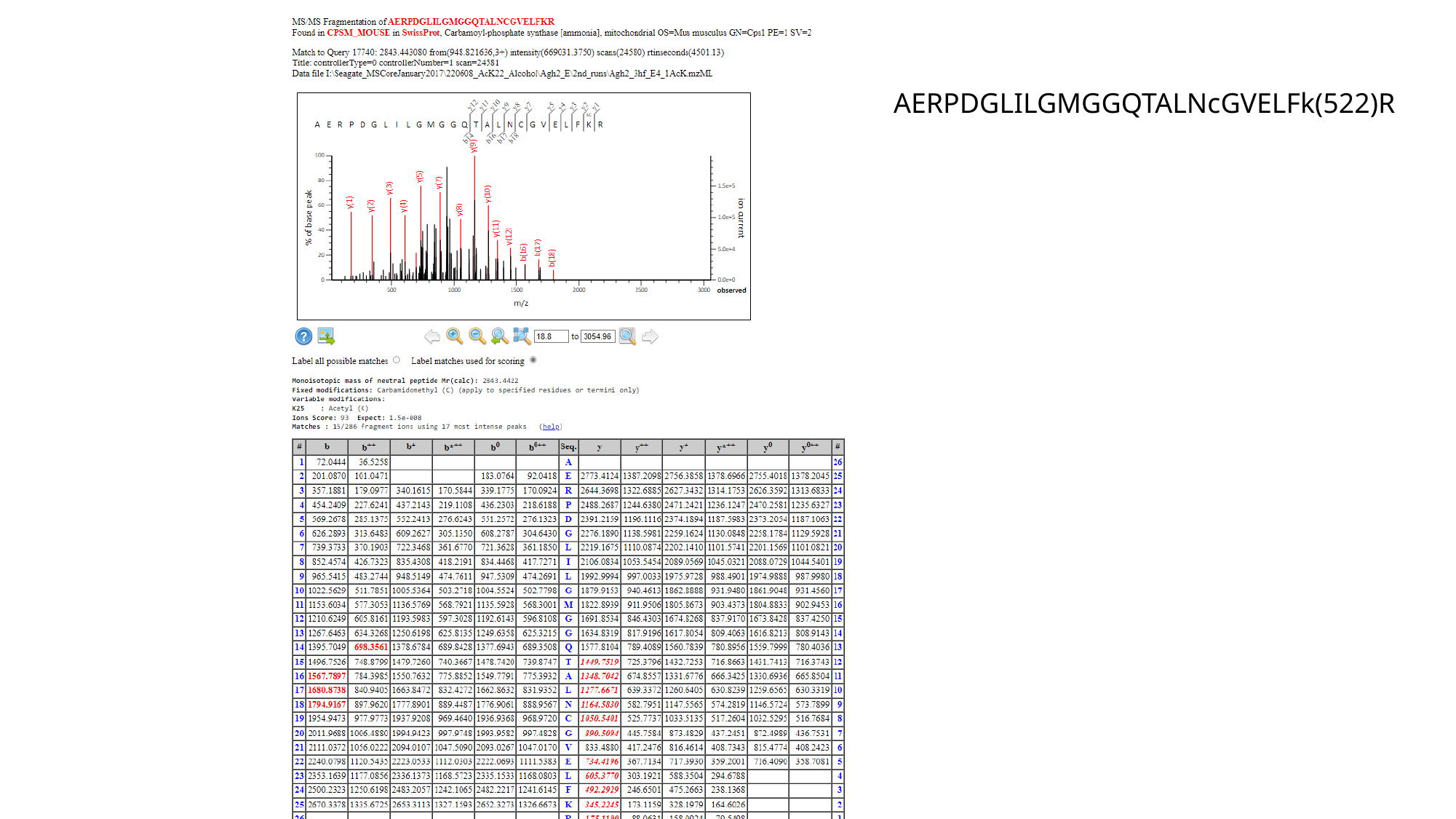

AERPDGLILGMGGQTALNcGVELFk(522)R

## Slide 57
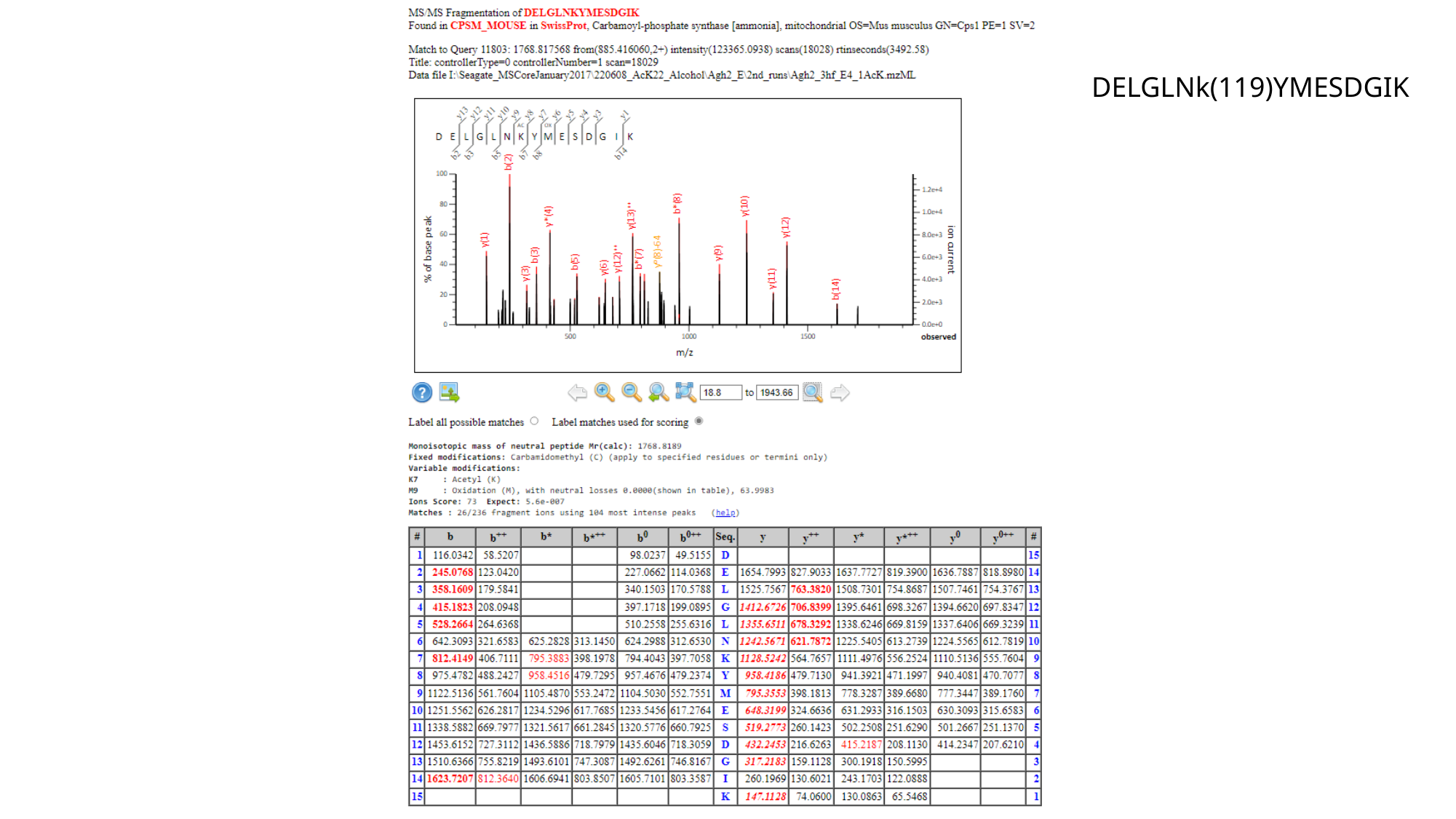

DELGLNk(119)YMESDGIK

## Slide 58
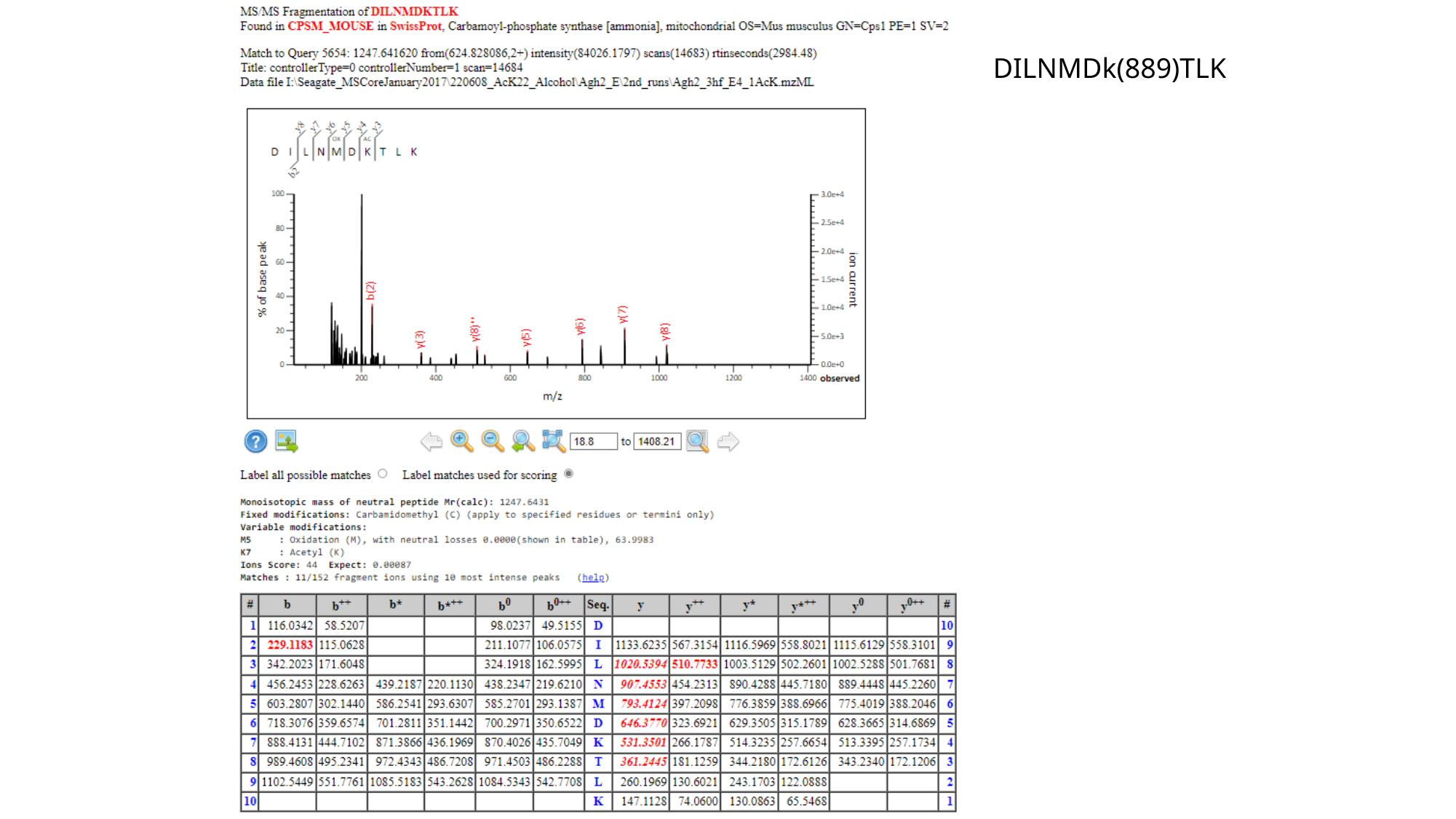

DILNMDk(889)TLK

## Slide 59
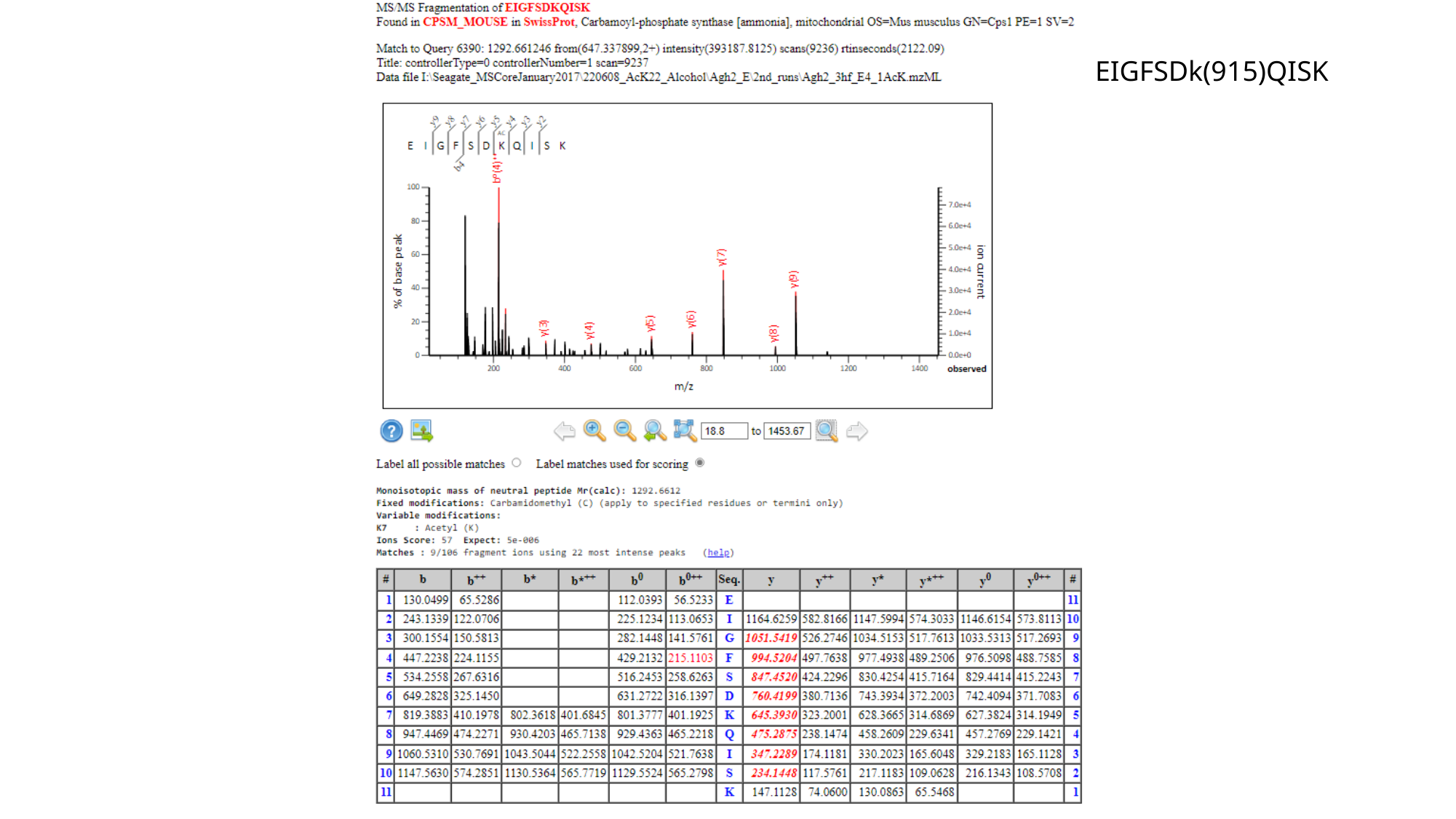

EIGFSDk(915)QISK

## Slide 60
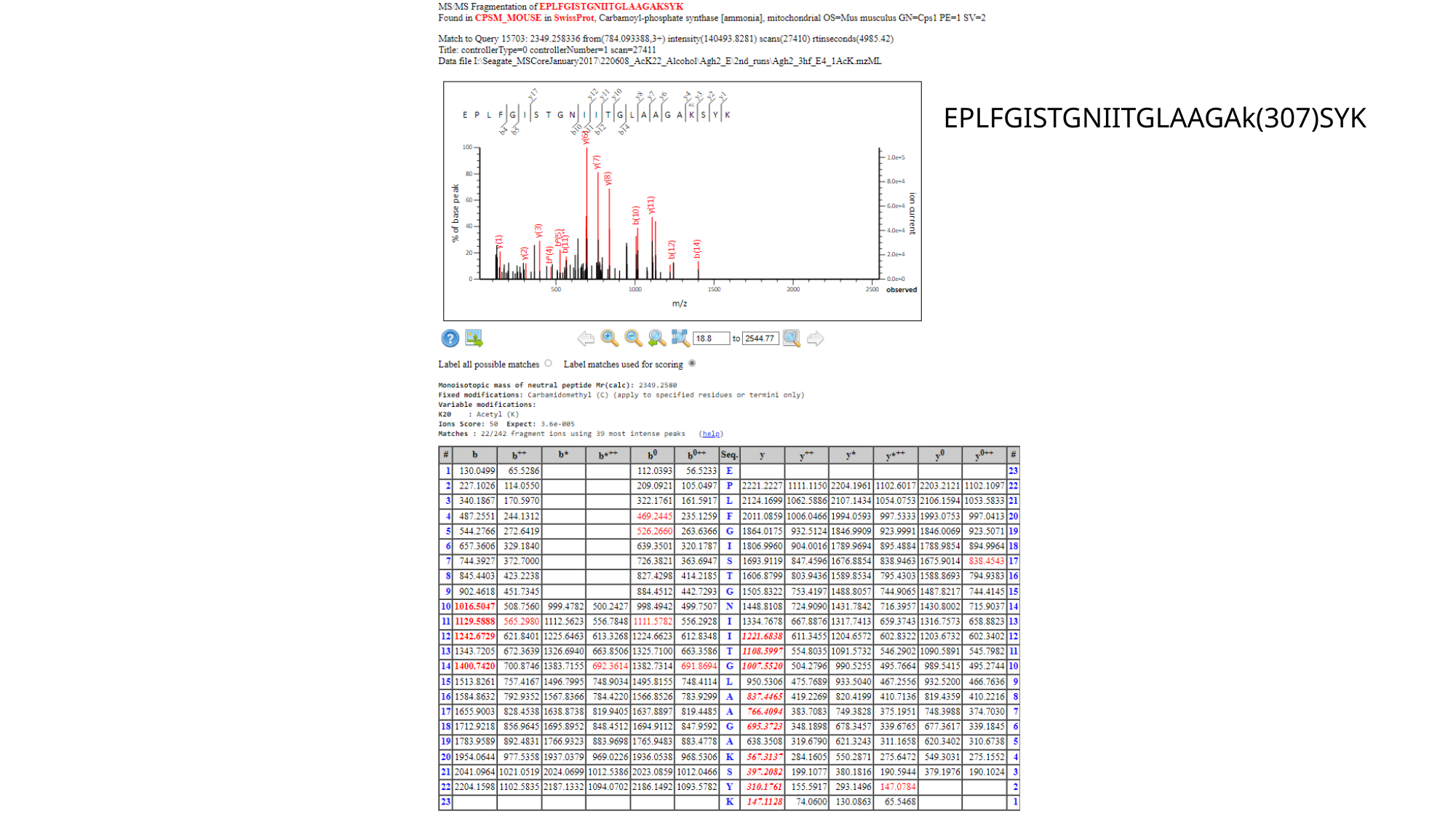

EPLFGISTGNIITGLAAGAk(307)SYK

## Slide 61
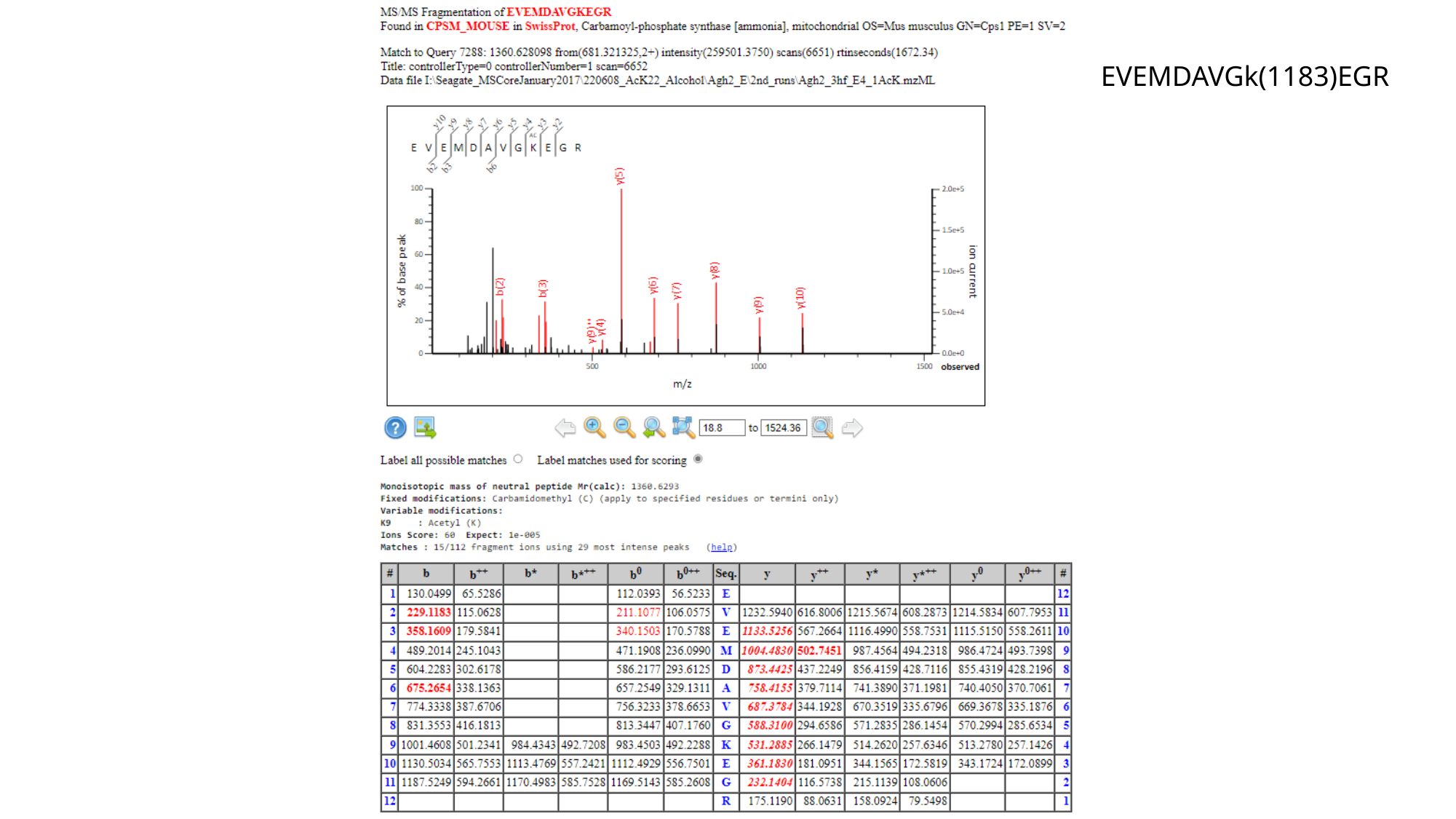

EVEMDAVGk(1183)EGR

## Slide 62
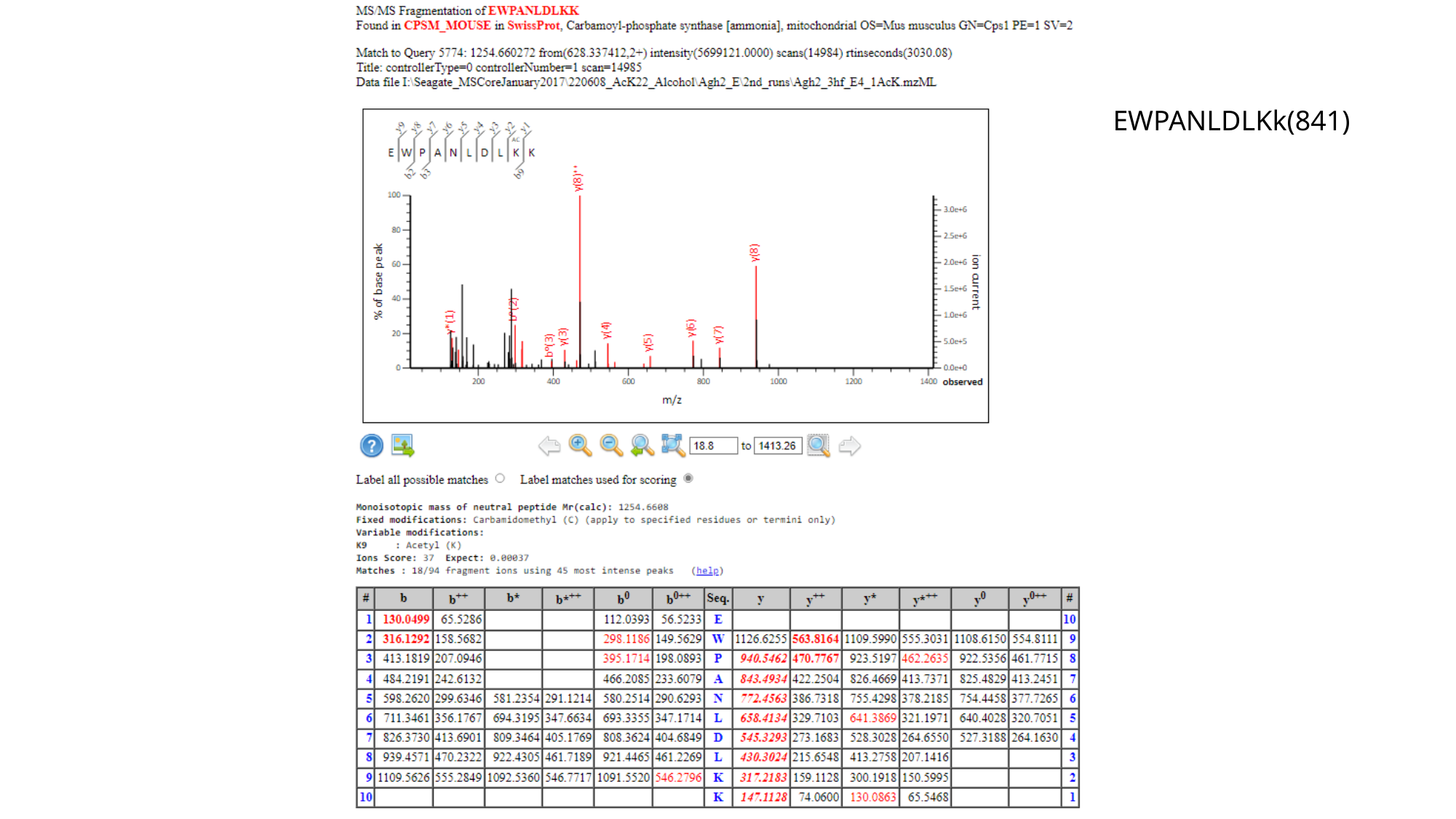

EWPANLDLKk(841)

## Slide 63
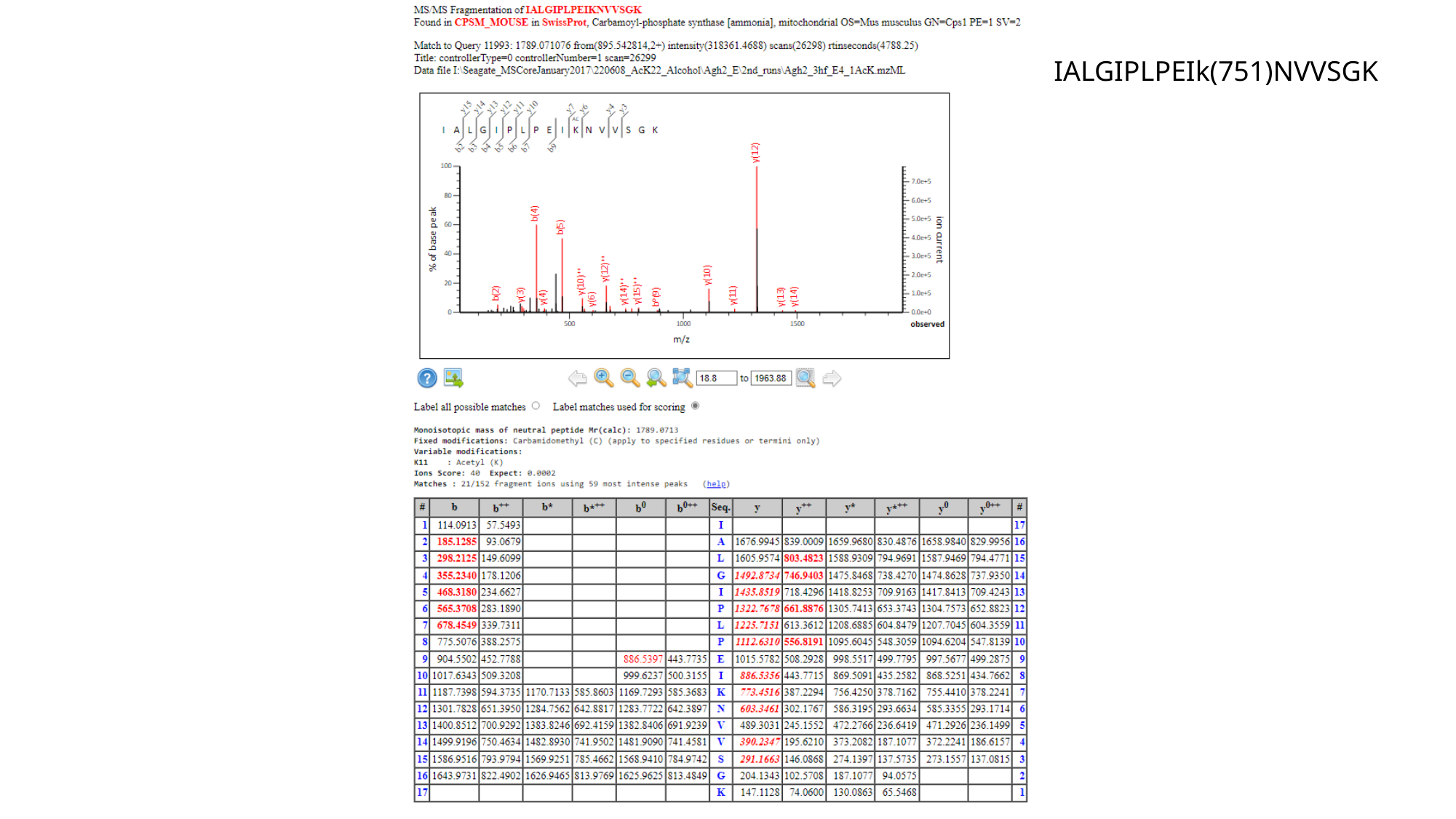

IALGIPLPEIk(751)NVVSGK

## Slide 64
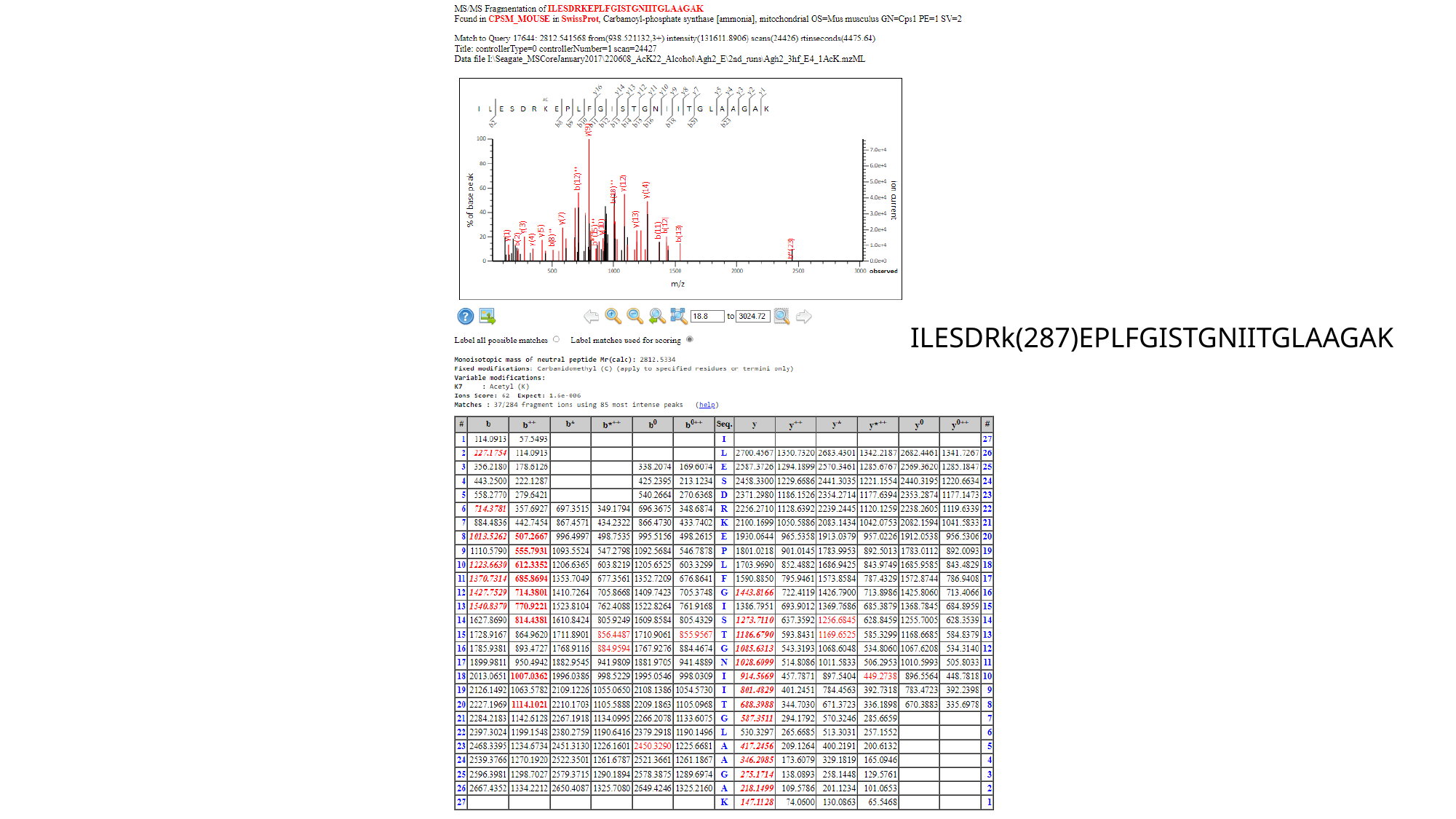

ILESDRk(287)EPLFGISTGNIITGLAAGAK

## Slide 65
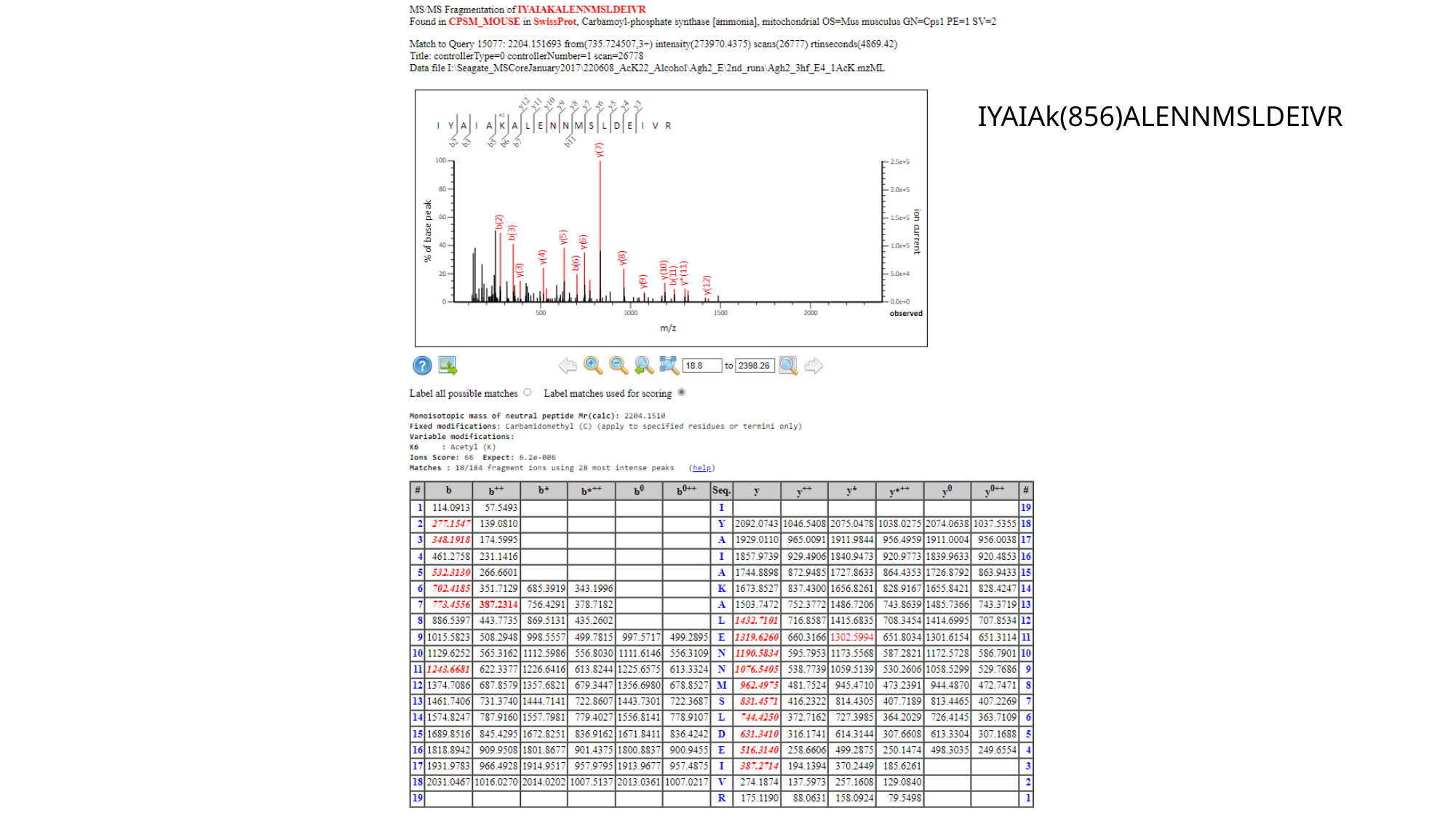

IYAIAk(856)ALENNMSLDEIVR

## Slide 66
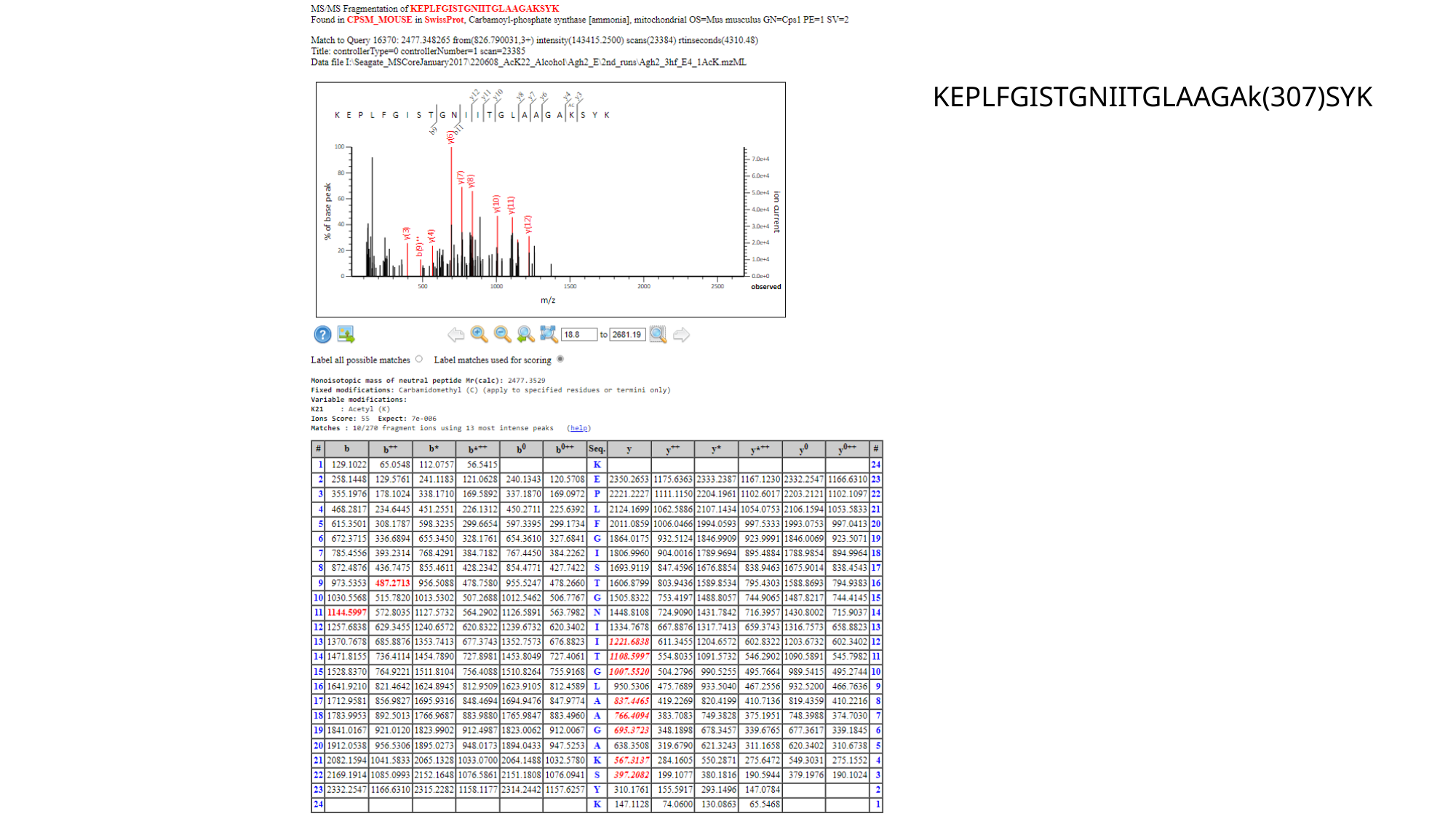

KEPLFGISTGNIITGLAAGAk(307)SYK

## Slide 67
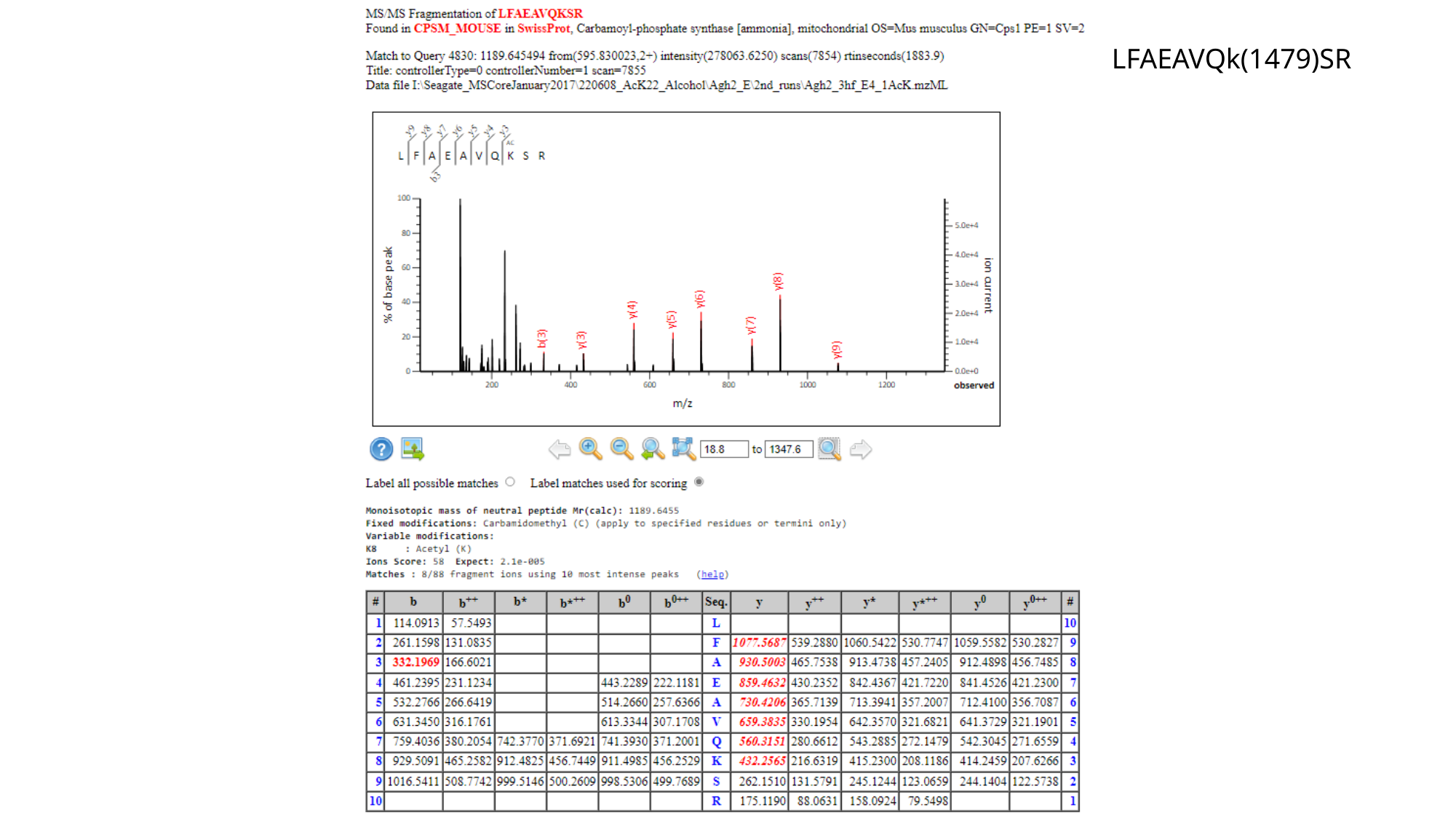

LFAEAVQk(1479)SR

## Slide 68
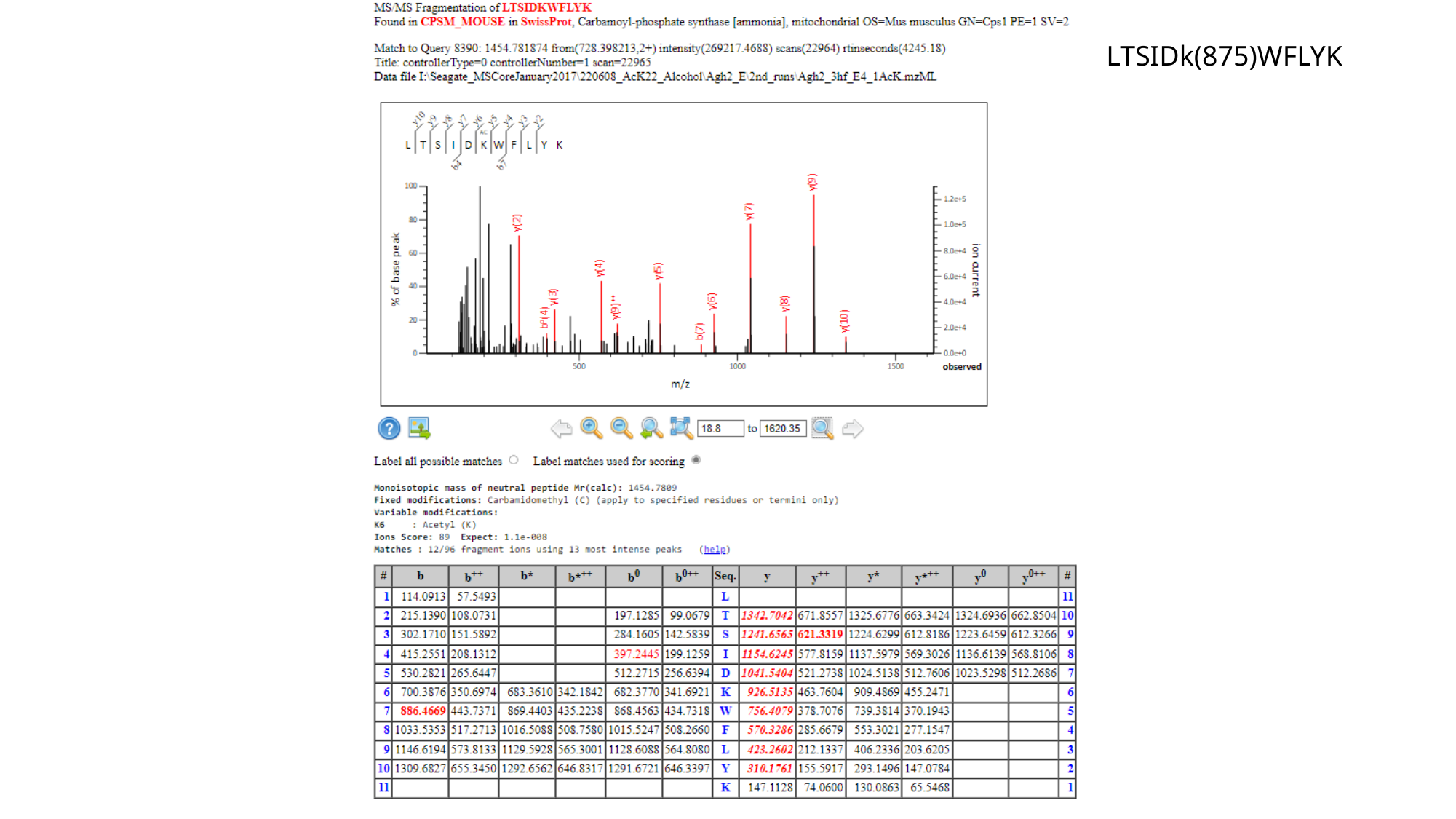

LTSIDk(875)WFLYK

## Slide 69
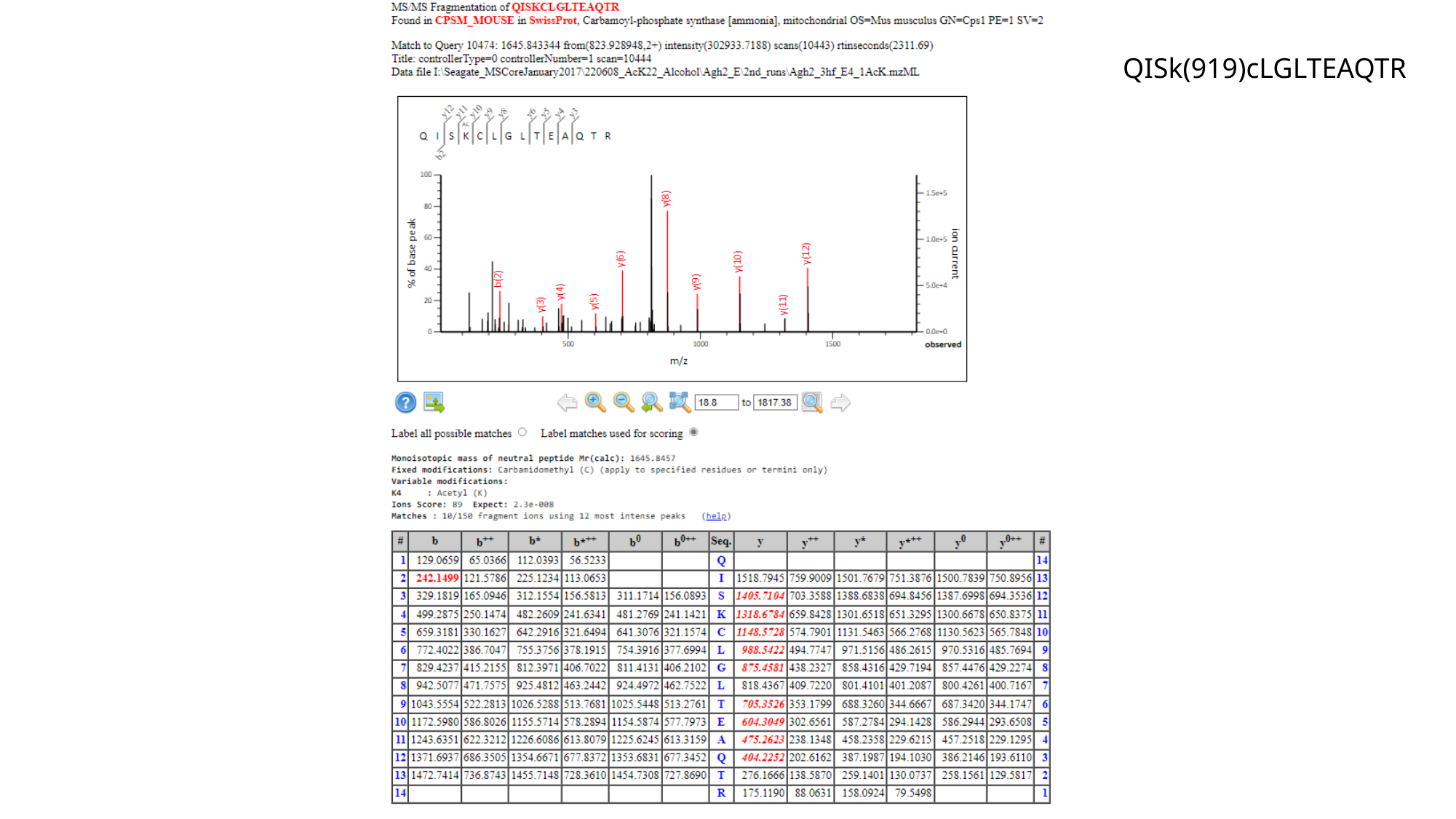

QISk(919)cLGLTEAQTR

## Slide 70
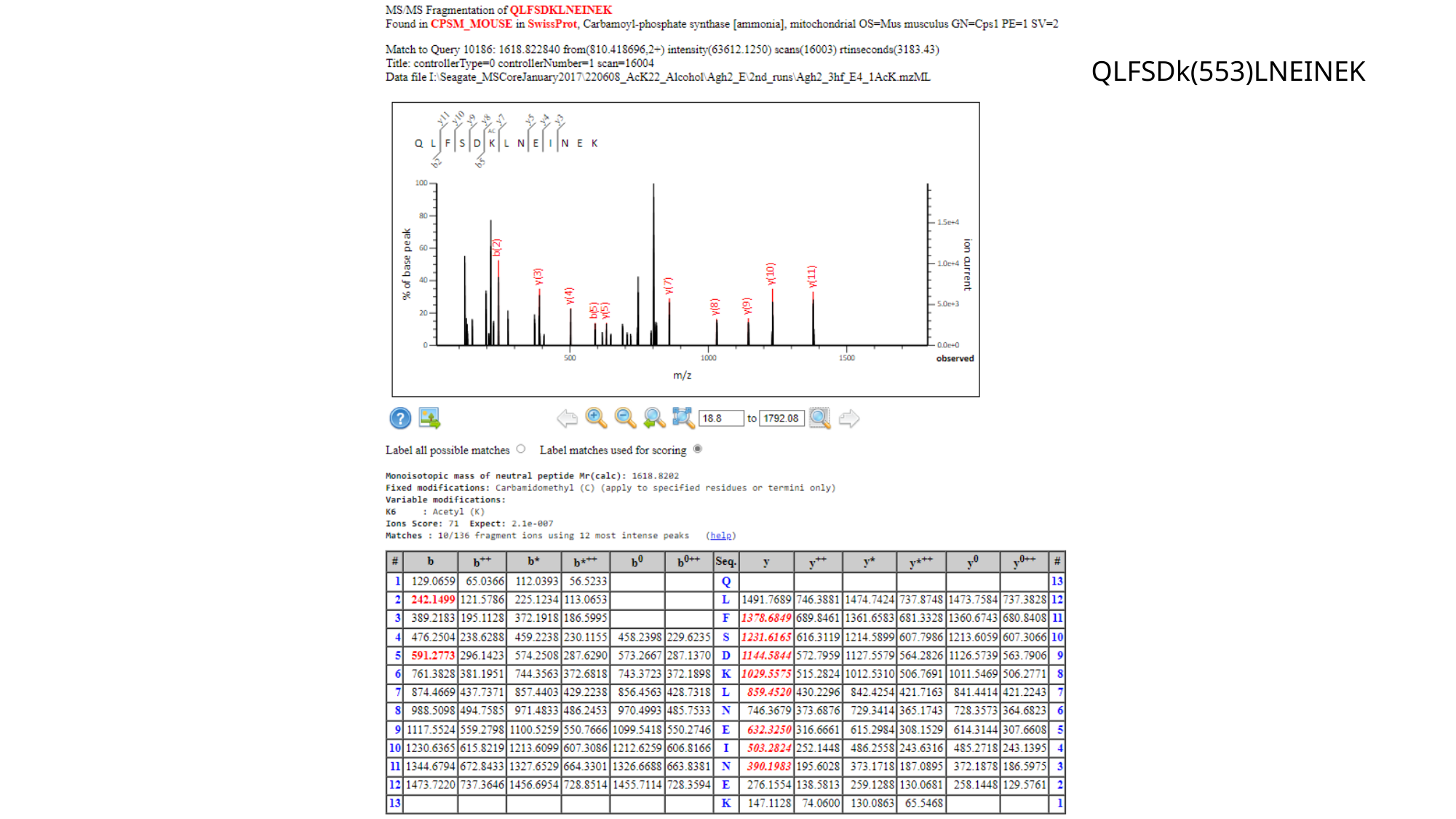

QLFSDk(553)LNEINEK

## Slide 71
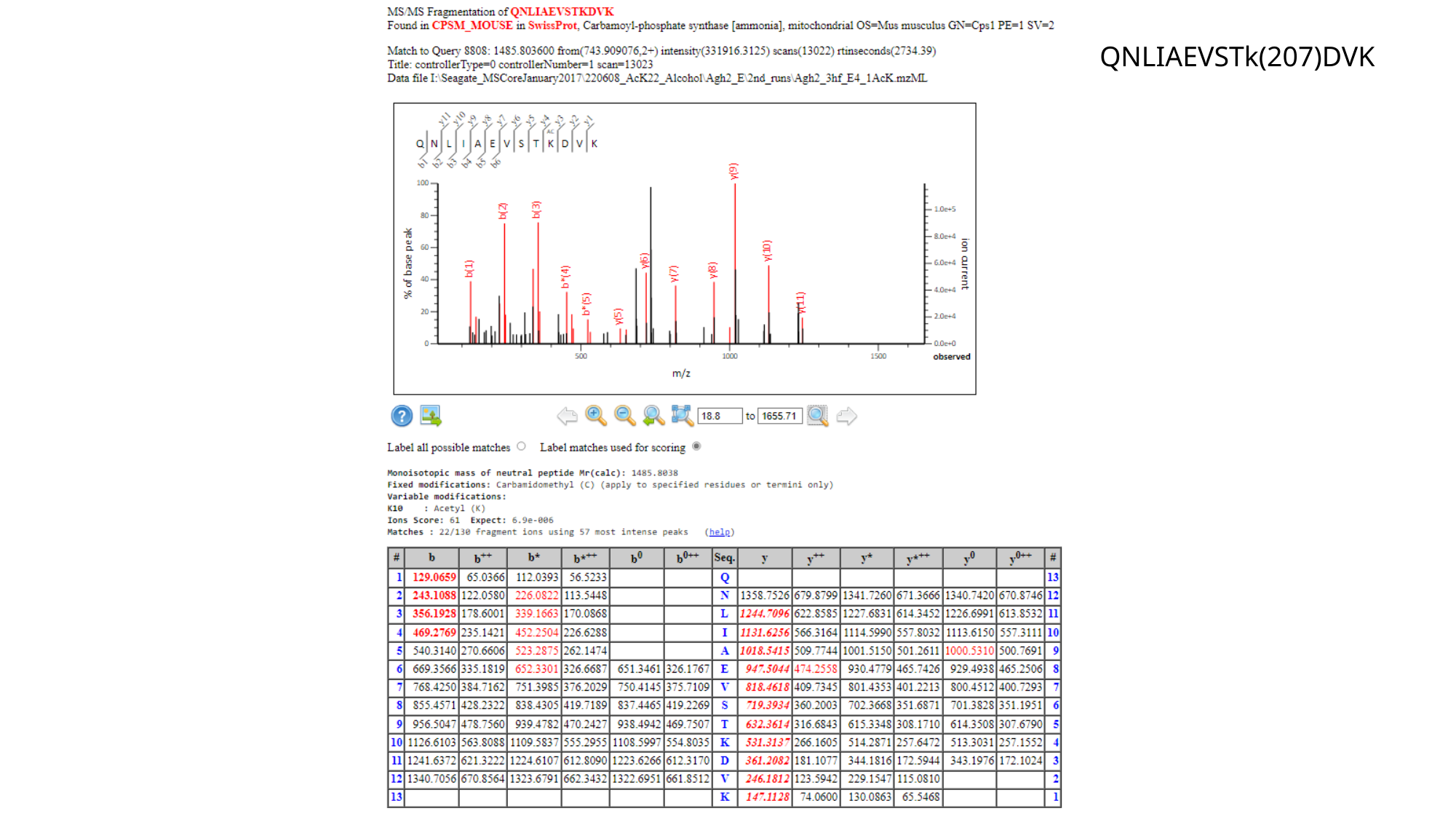

QNLIAEVSTk(207)DVK

## Slide 72
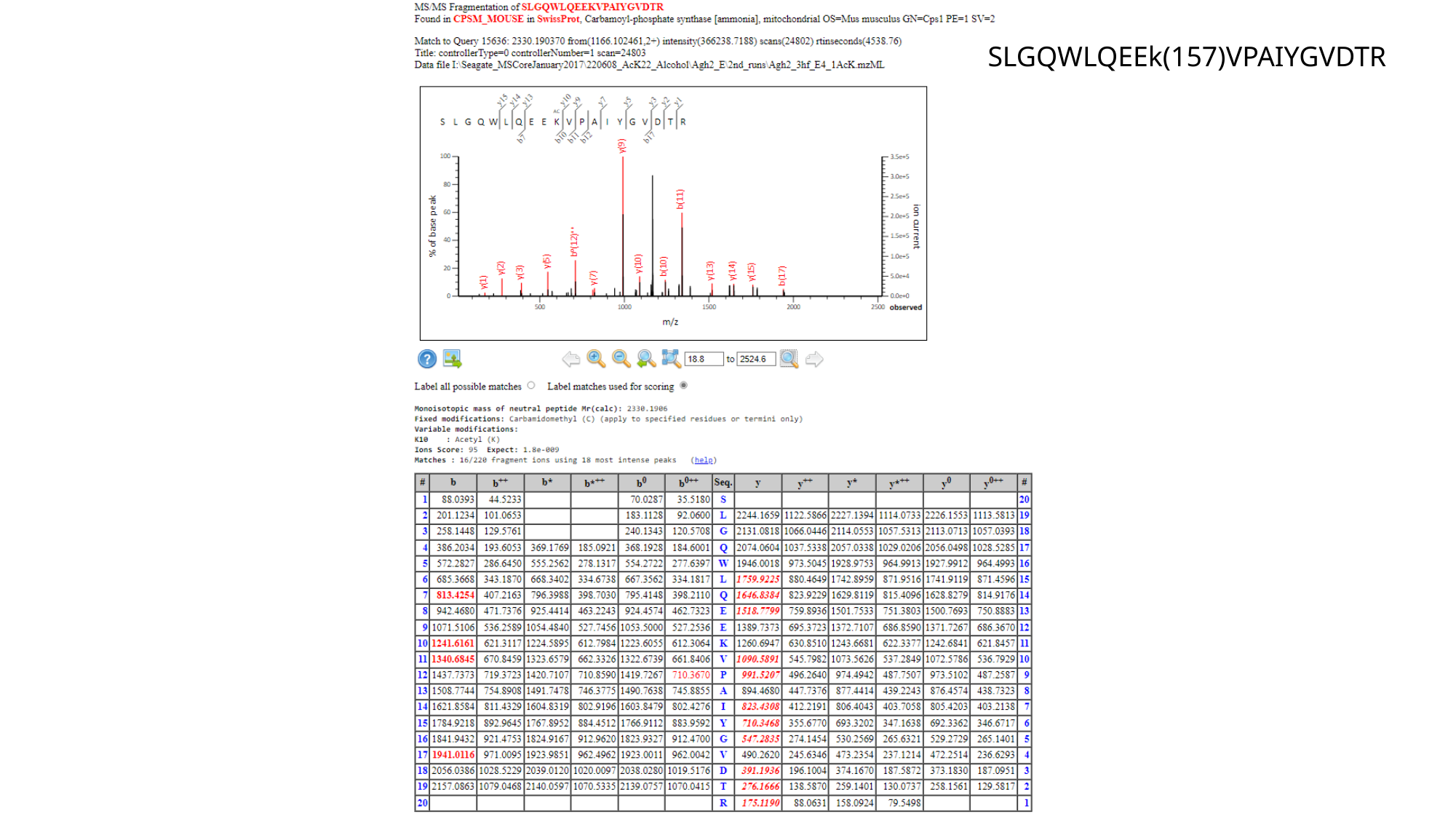

SLGQWLQEEk(157)VPAIYGVDTR

## Slide 73
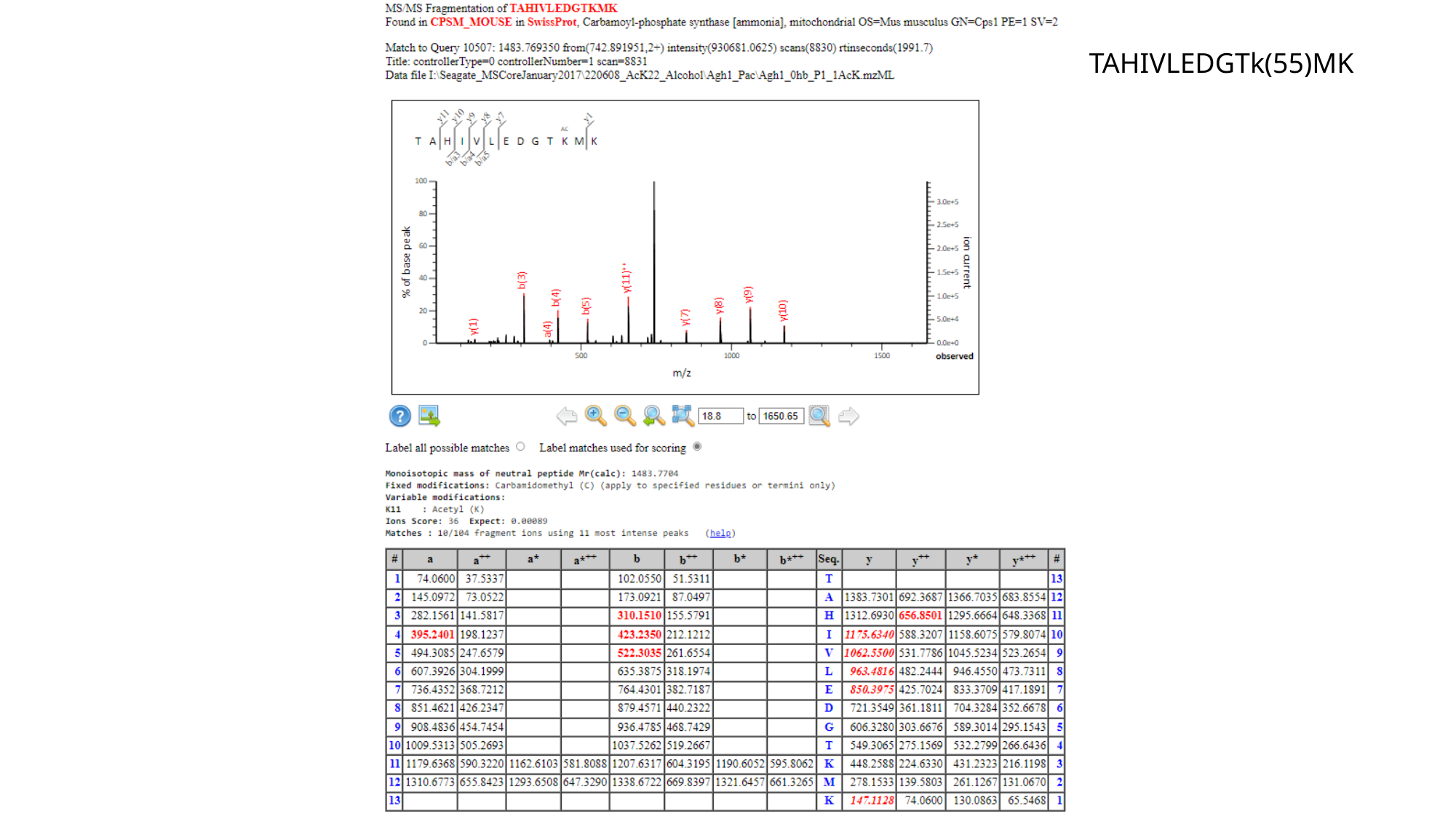

TAHIVLEDGTk(55)MK

## Slide 74
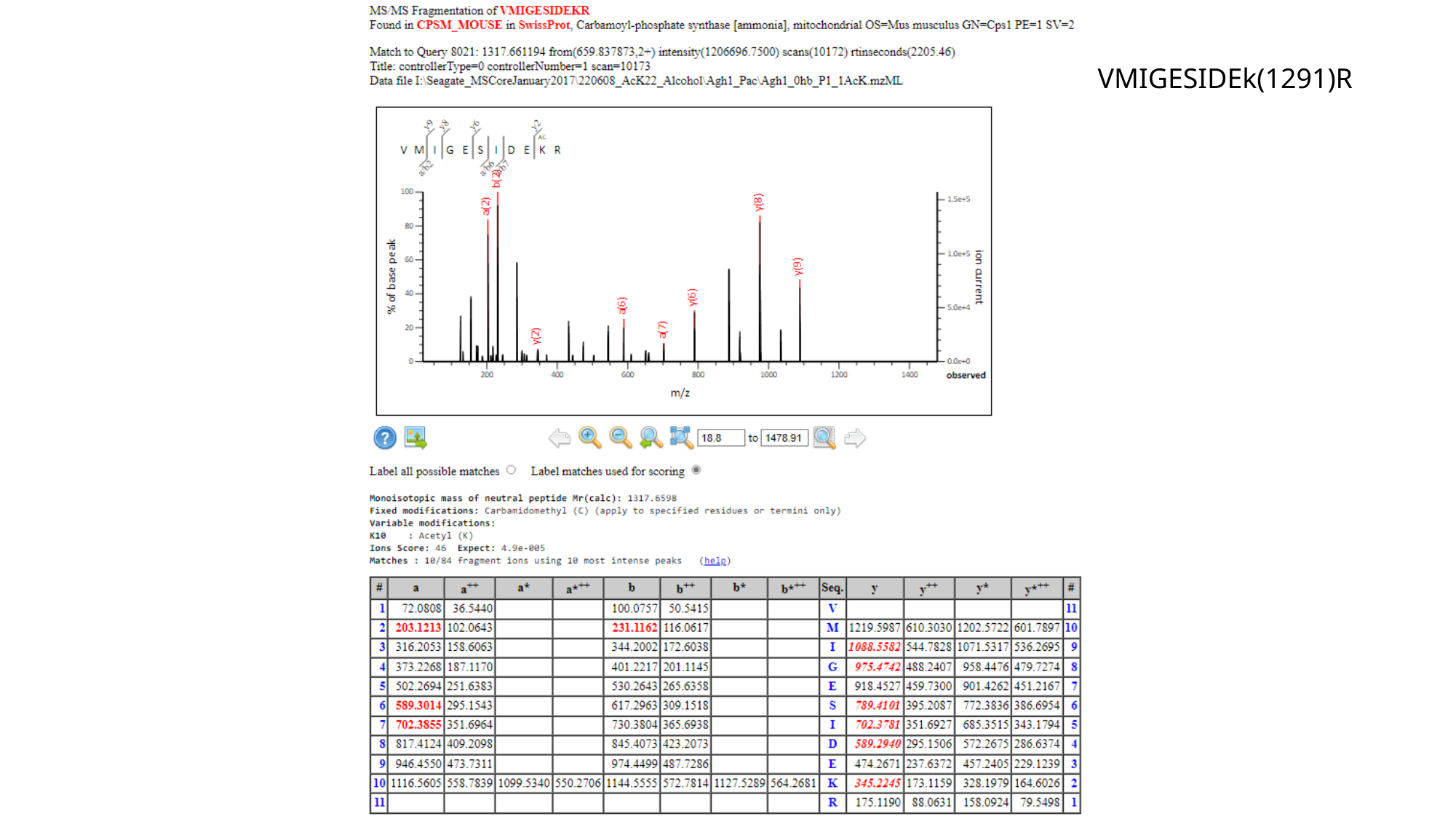

VMIGESIDEk(1291)R

## Slide 75
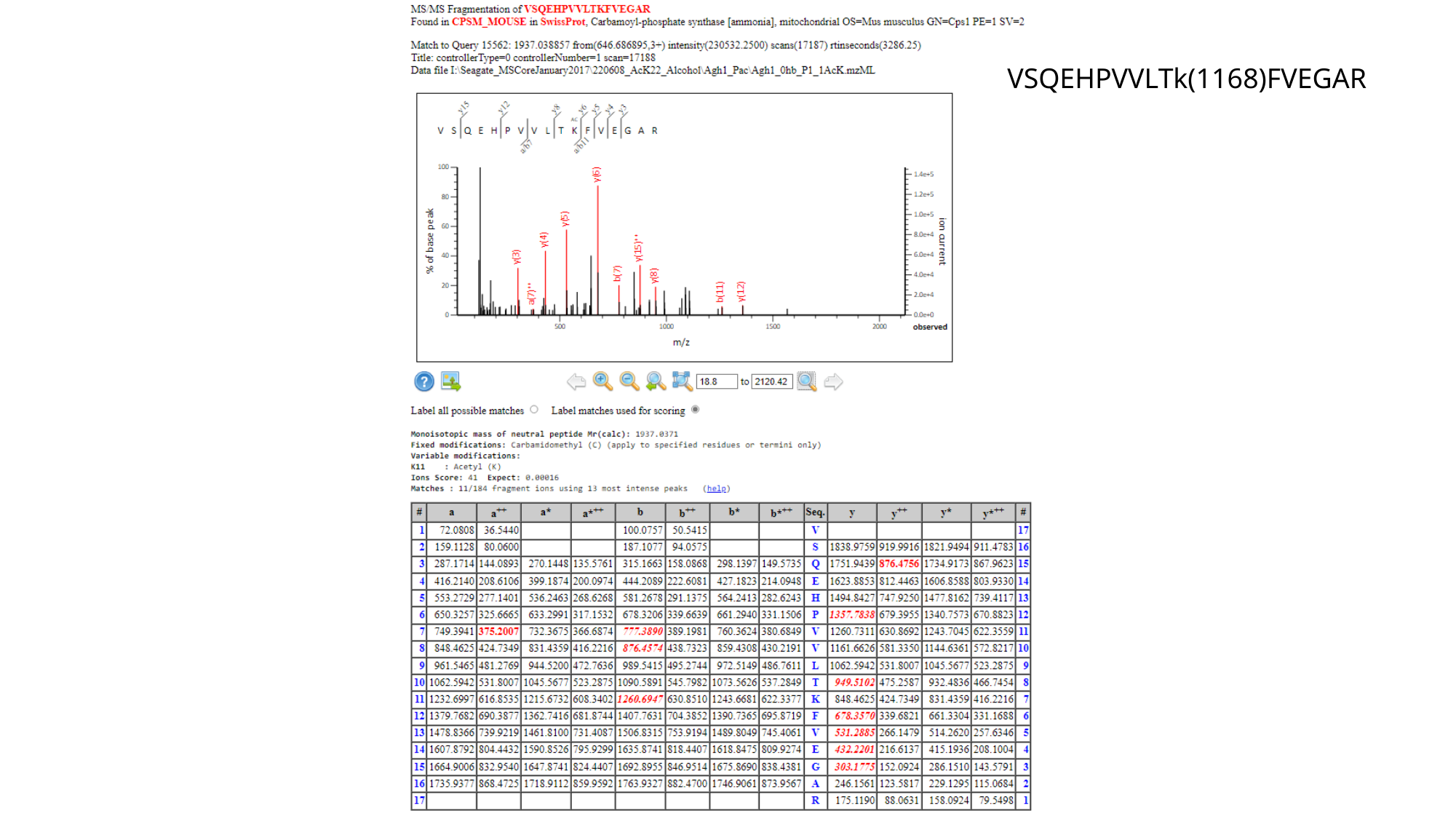

VSQEHPVVLTk(1168)FVEGAR

## Slide 76
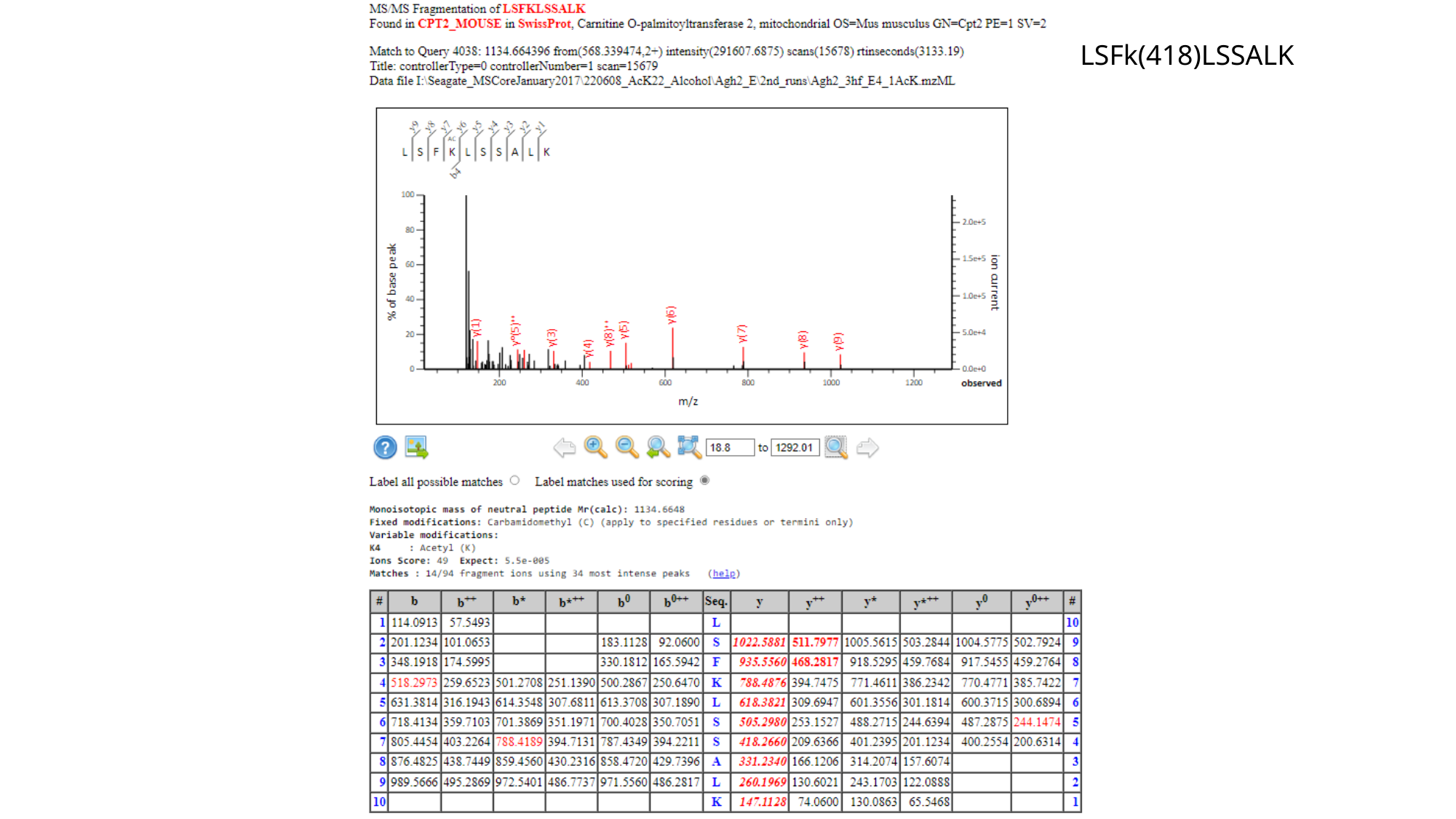

LSFk(418)LSSALK

## Slide 77
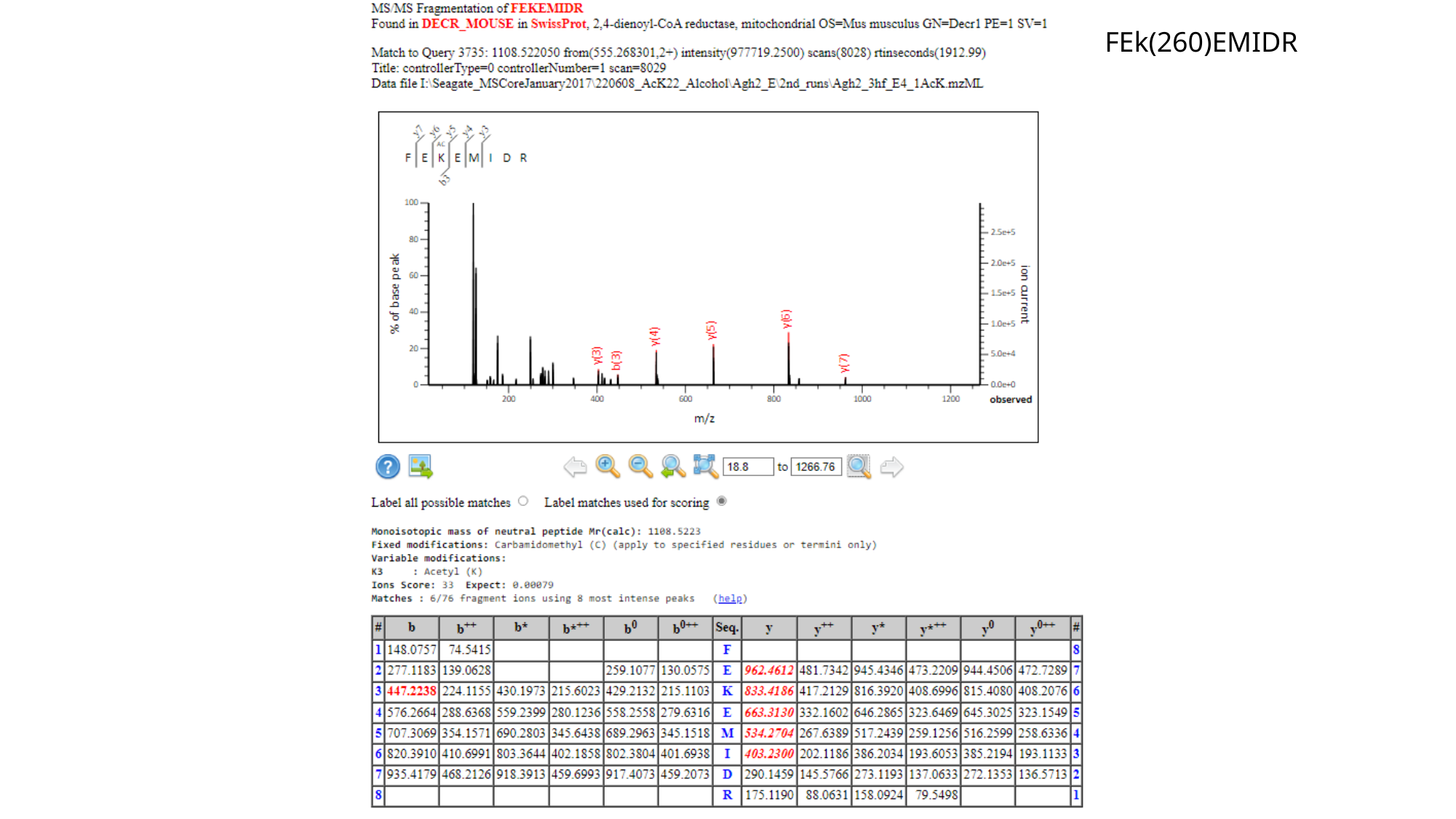

FEk(260)EMIDR

## Slide 78
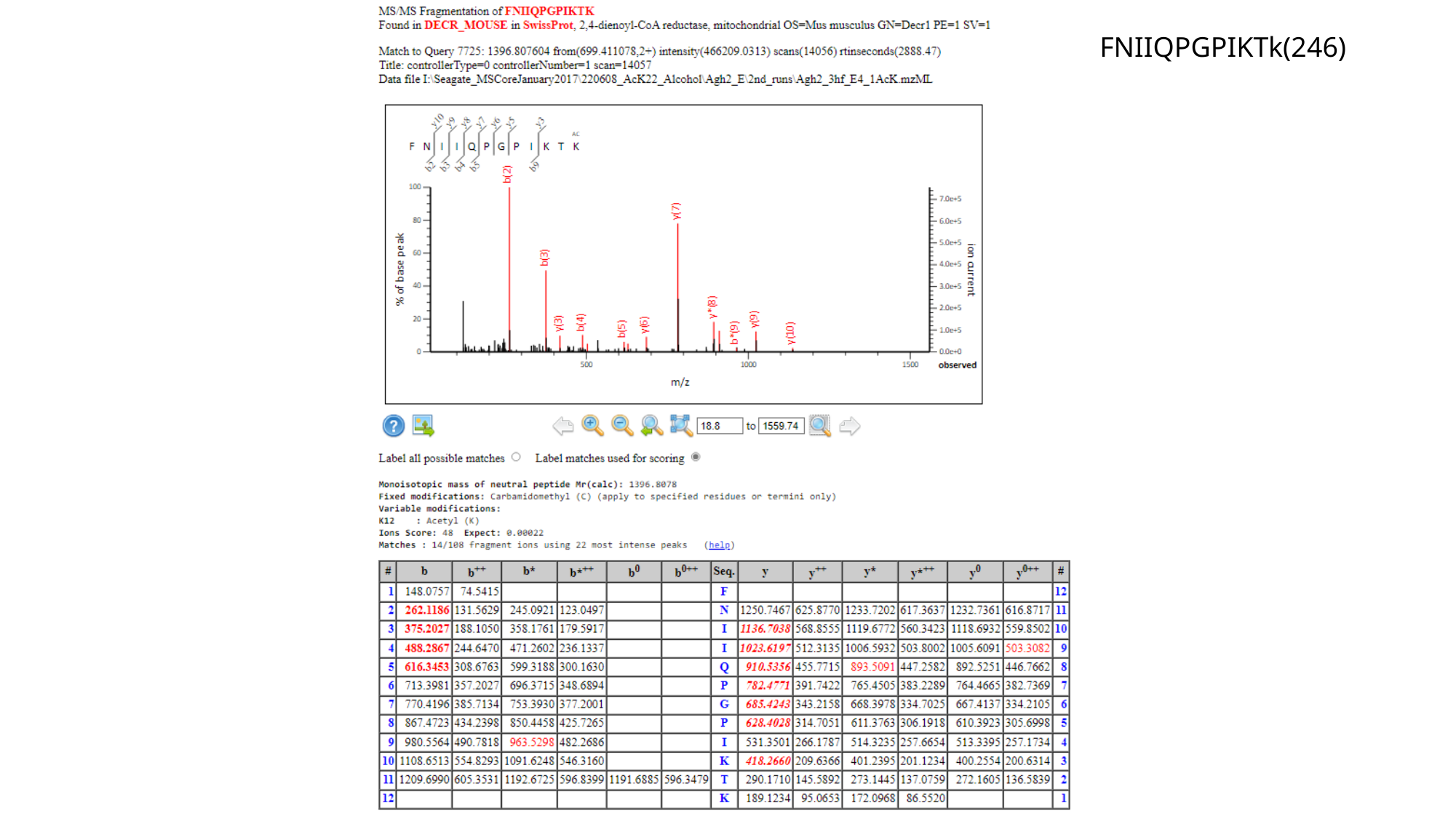

FNIIQPGPIKTk(246)

## Slide 79
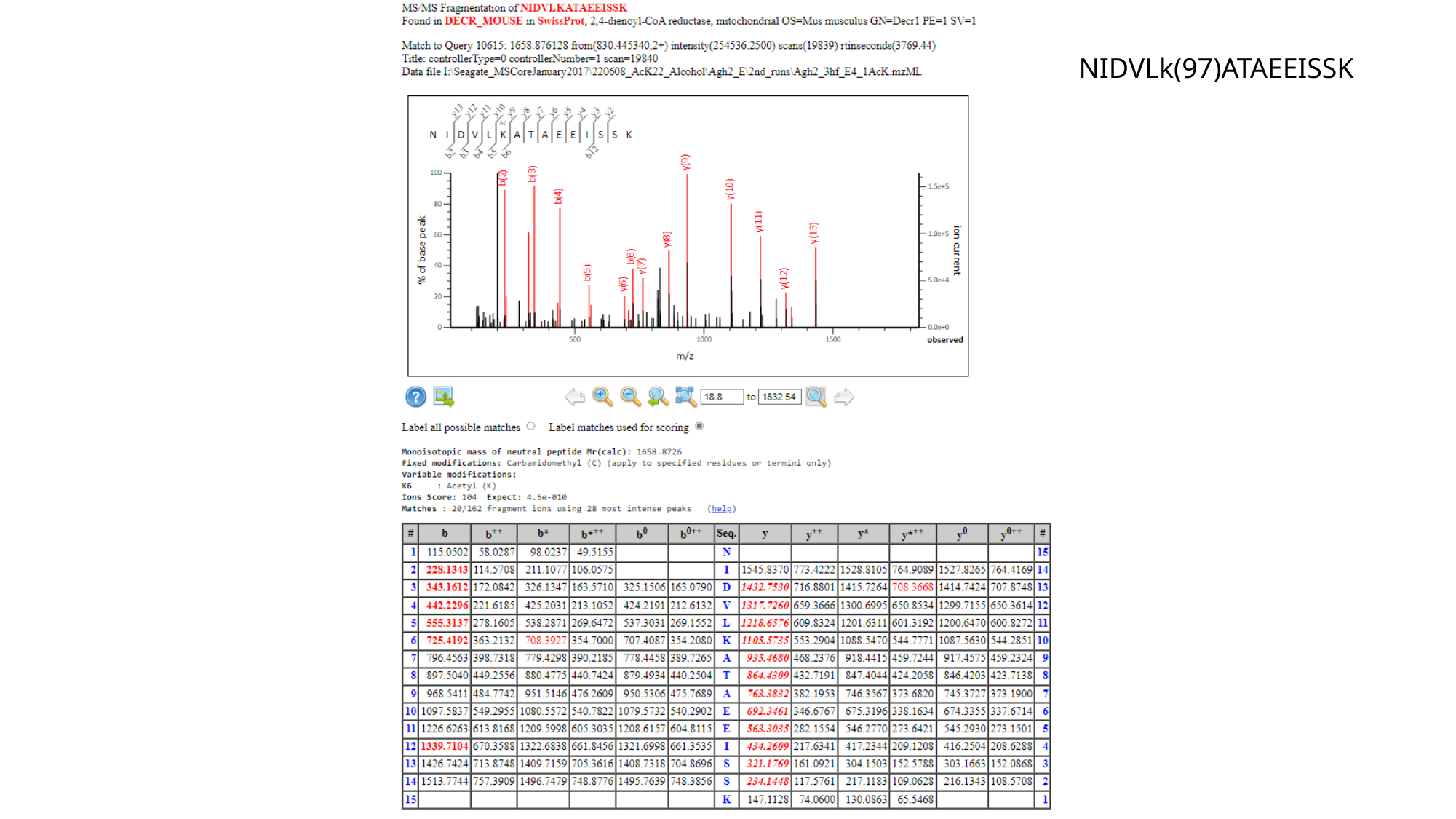

NIDVLk(97)ATAEEISSK

## Slide 80
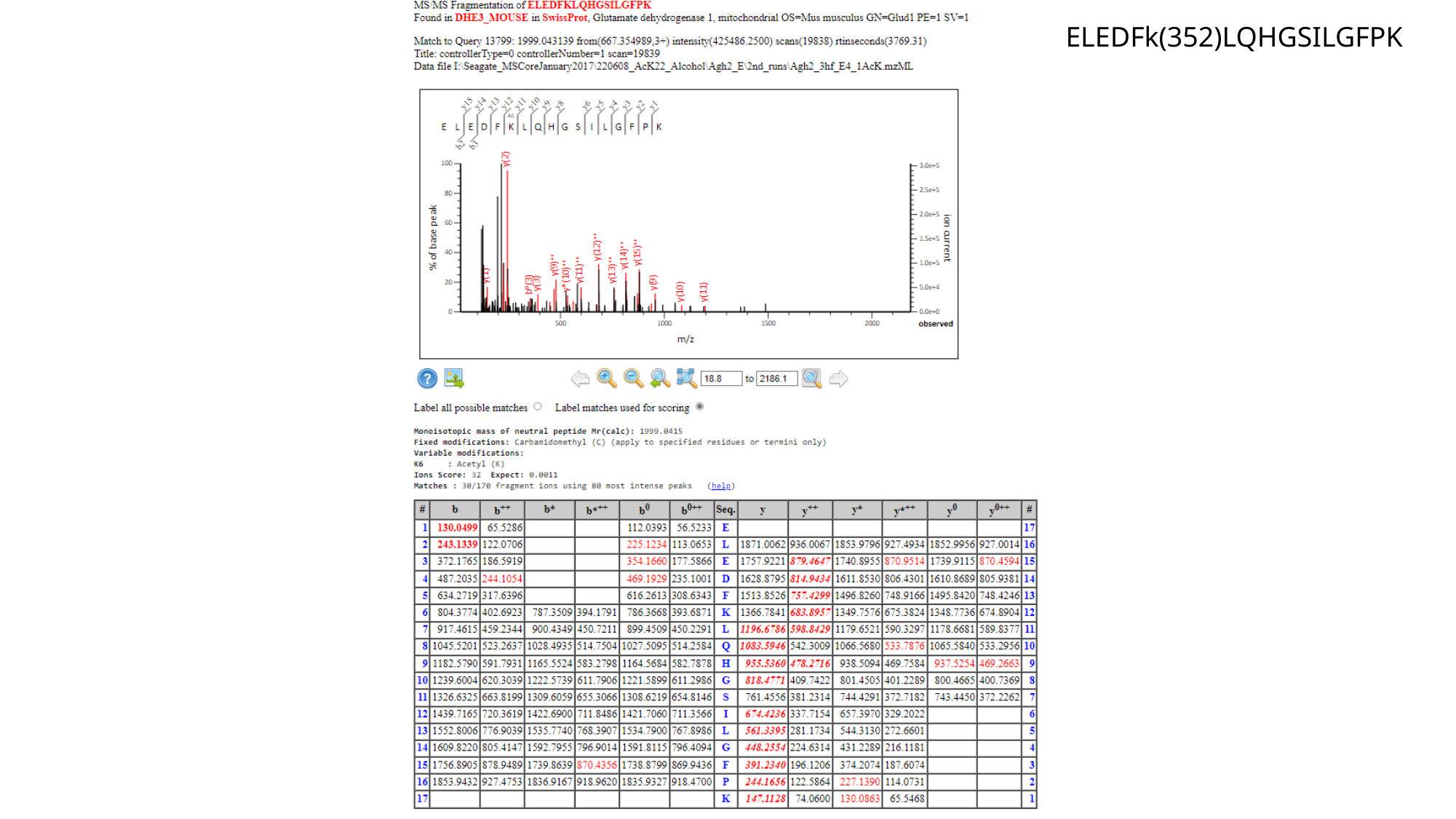

ELEDFk(352)LQHGSILGFPK

## Slide 81
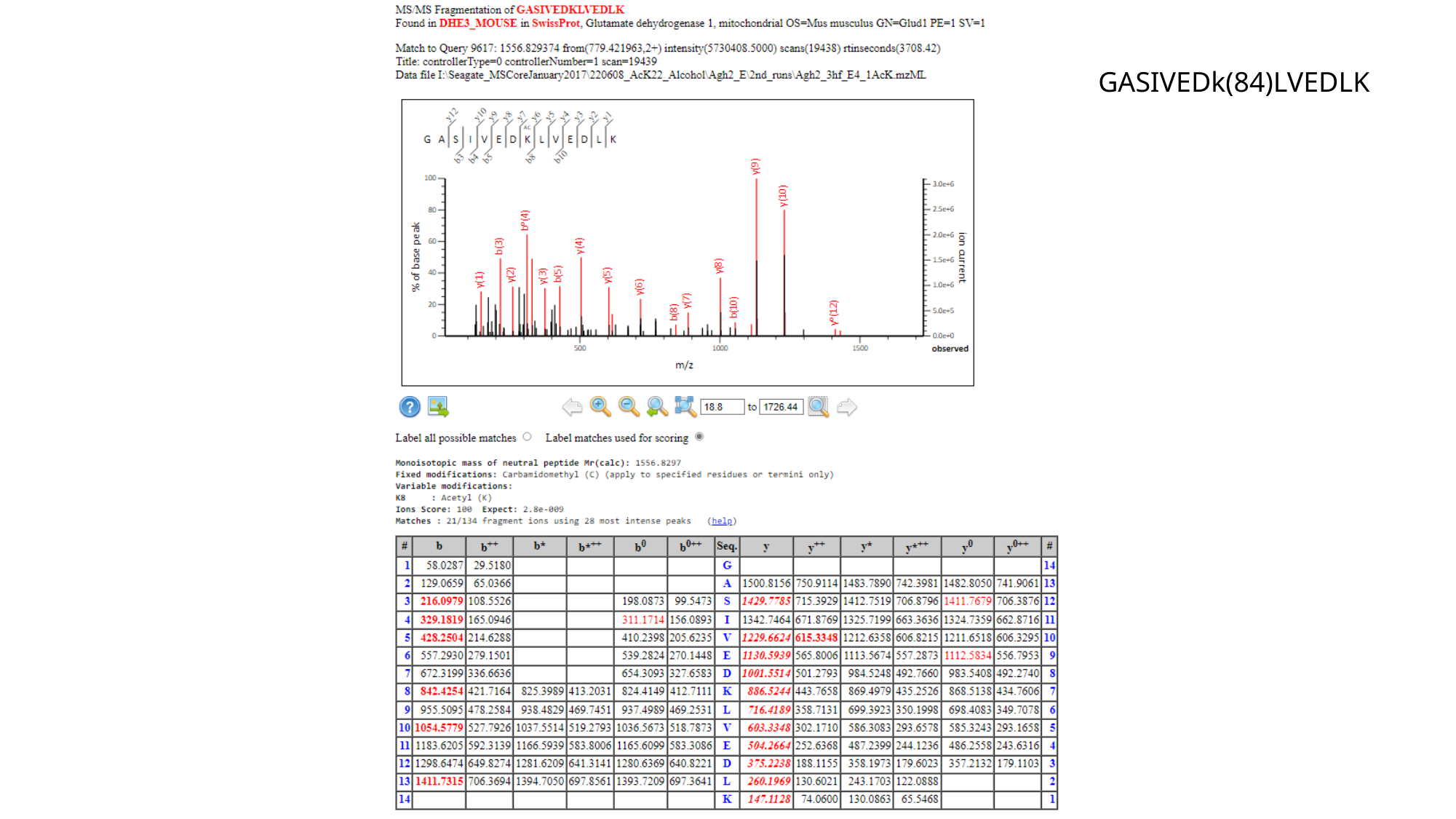

GASIVEDk(84)LVEDLK

## Slide 82
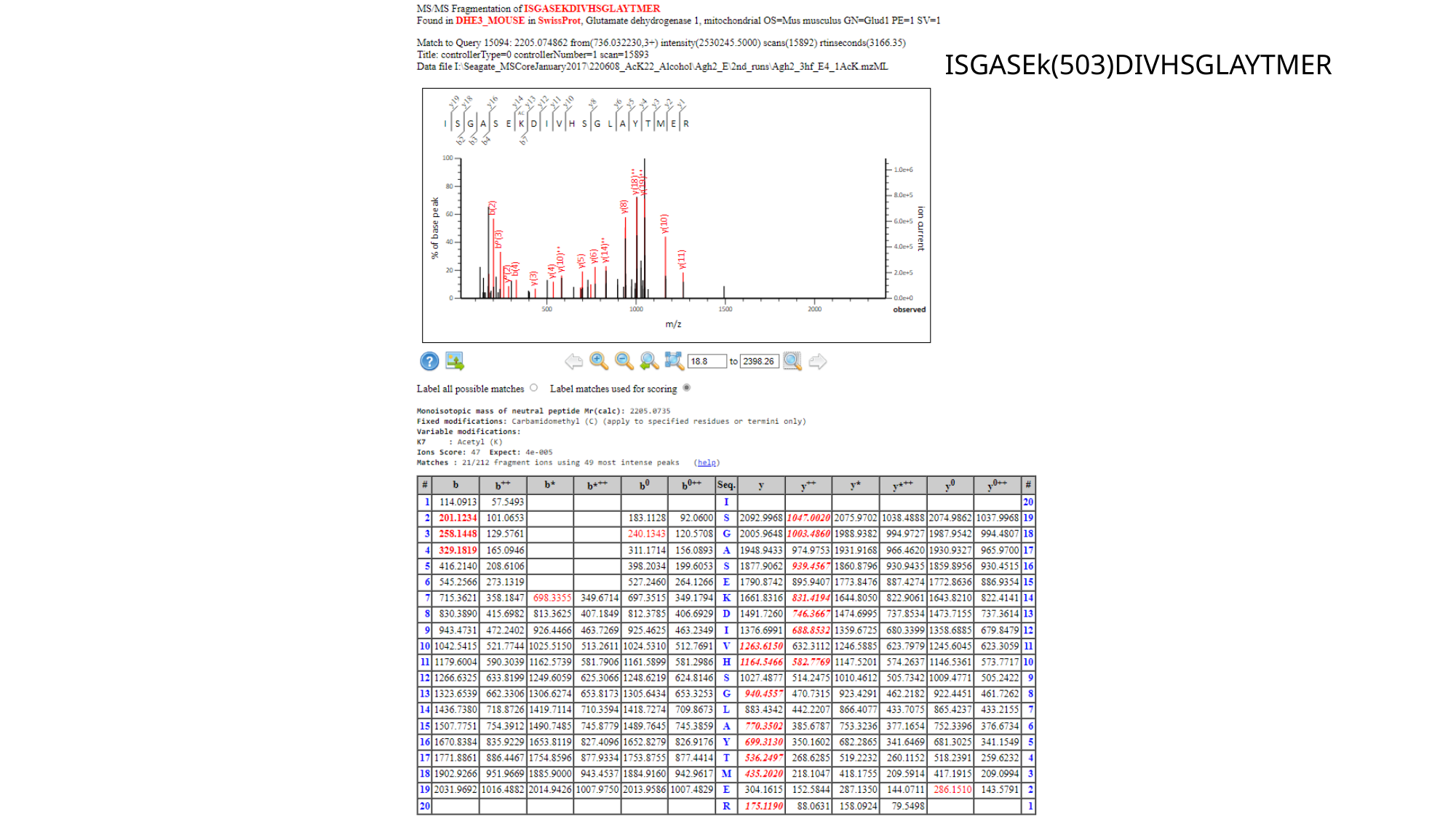

ISGASEk(503)DIVHSGLAYTMER

## Slide 83
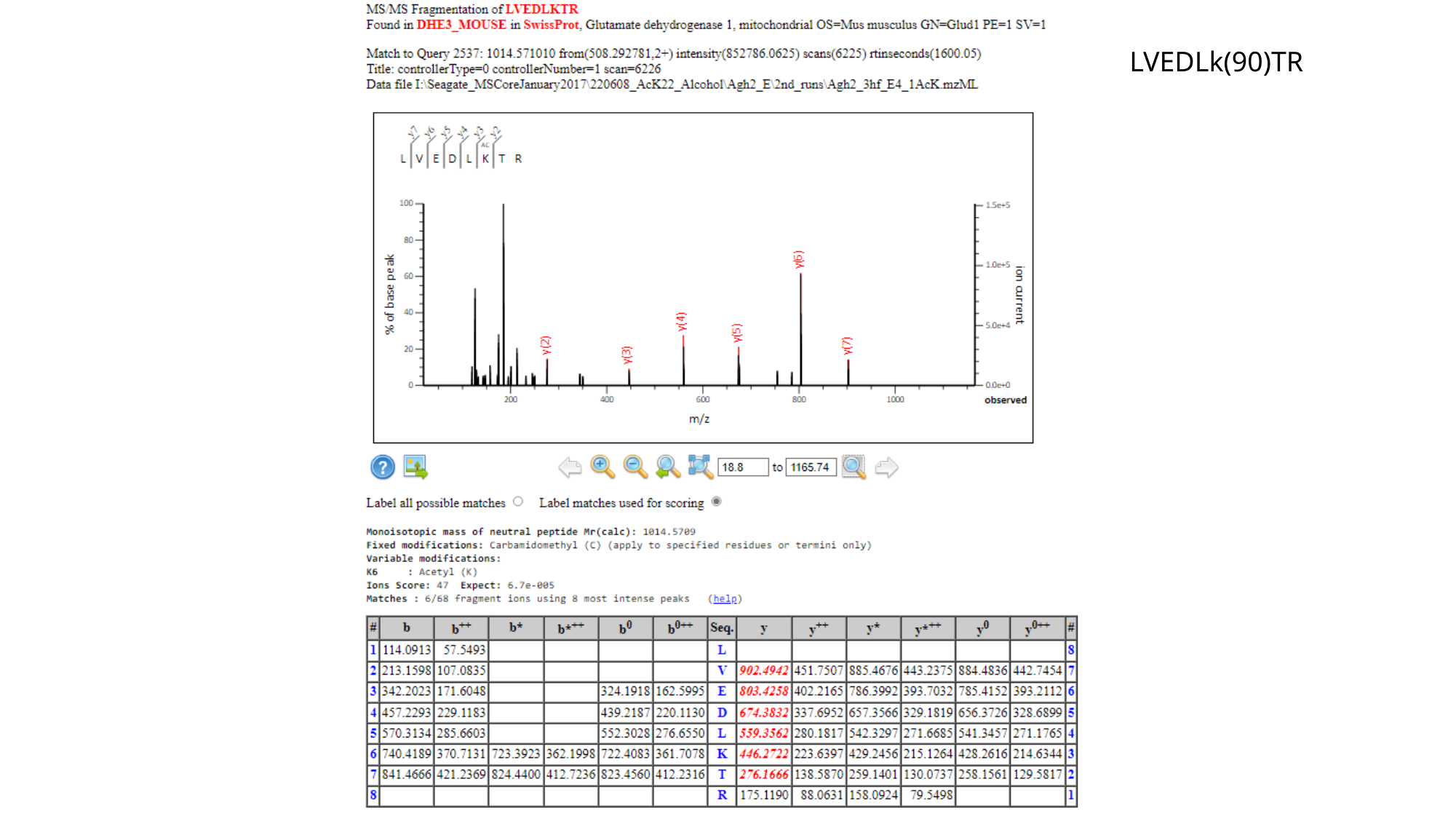

LVEDLk(90)TR

## Slide 84
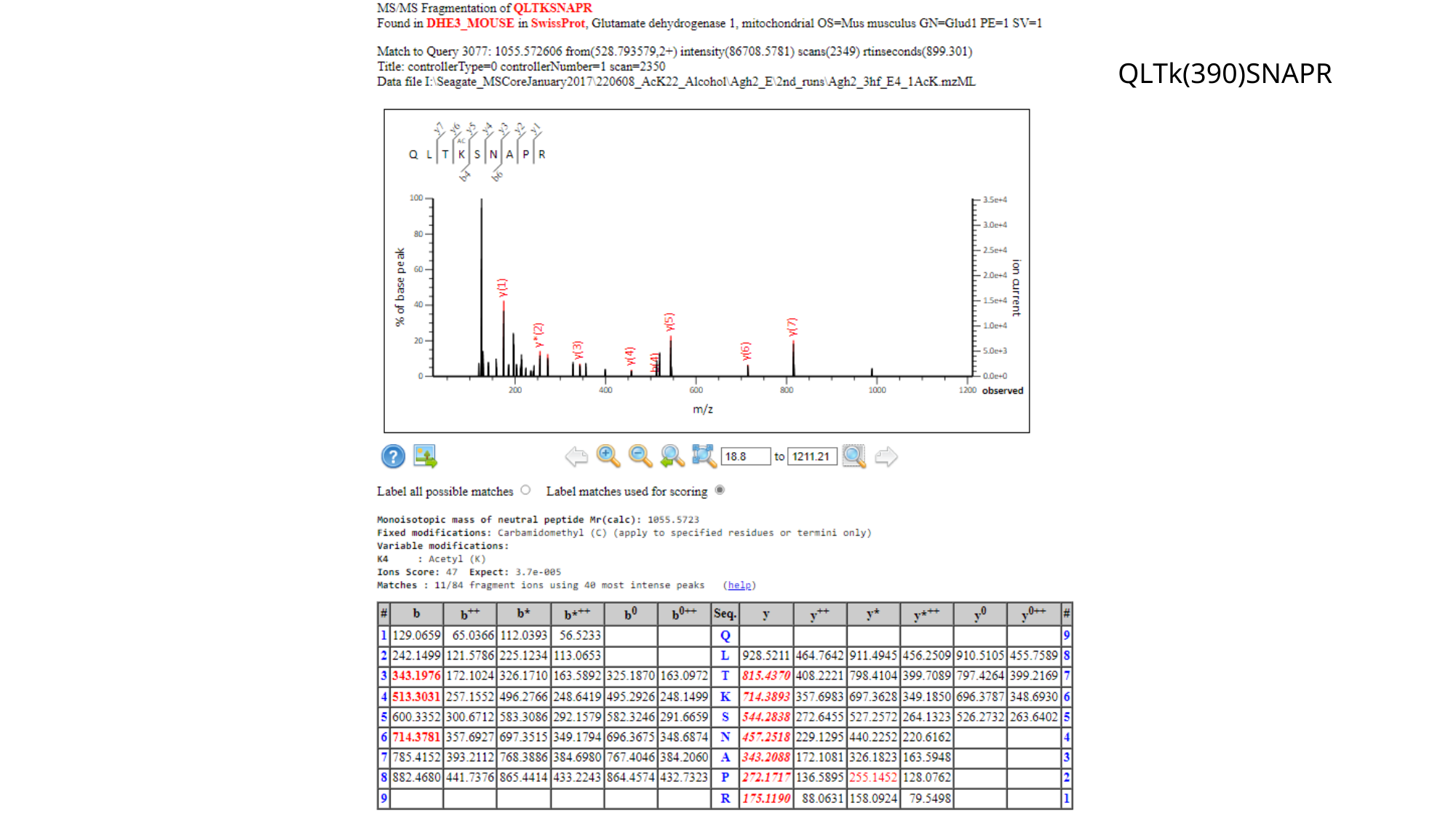

QLTk(390)SNAPR

## Slide 85
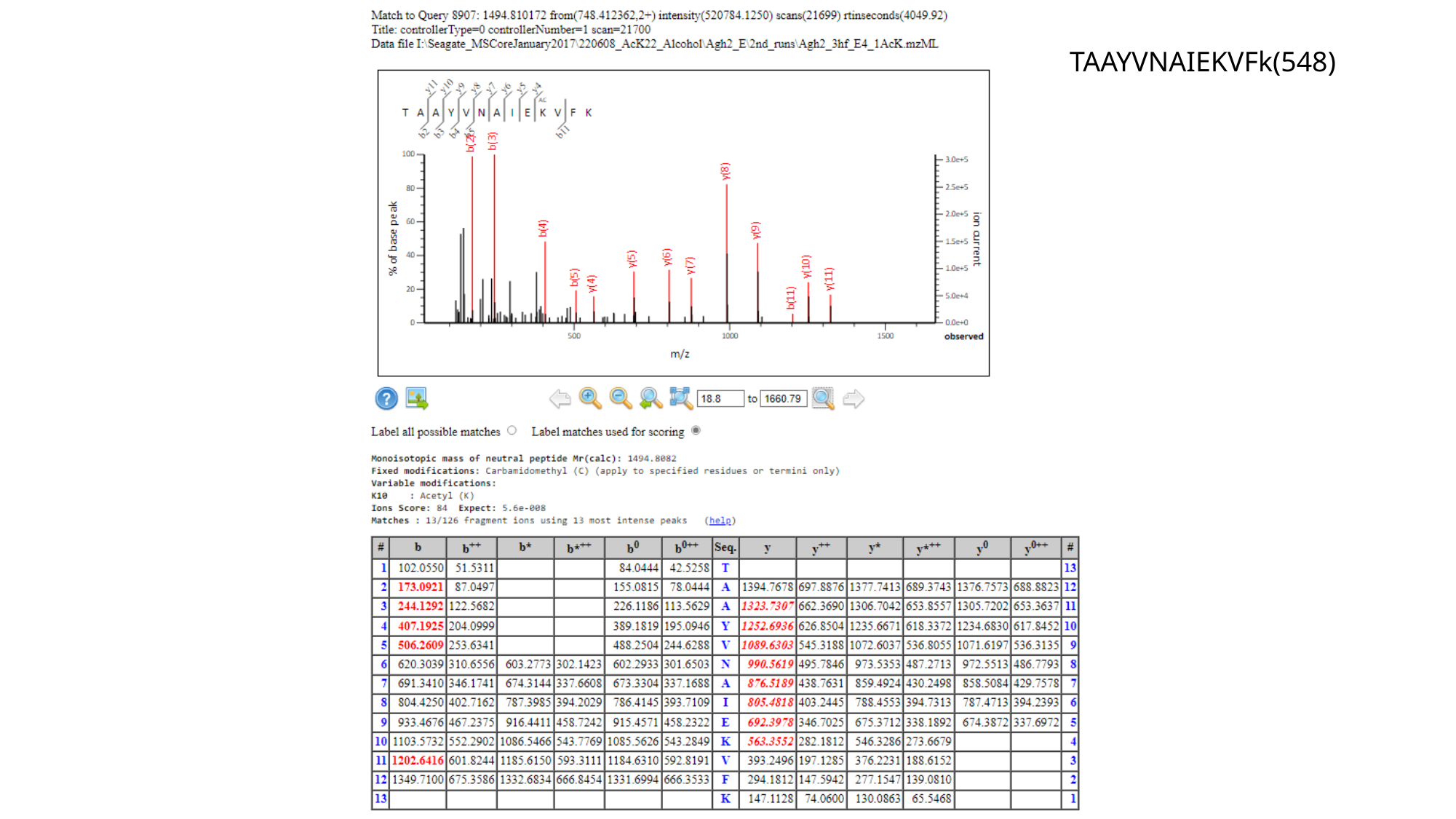

TAAYVNAIEKVFk(548)

## Slide 86
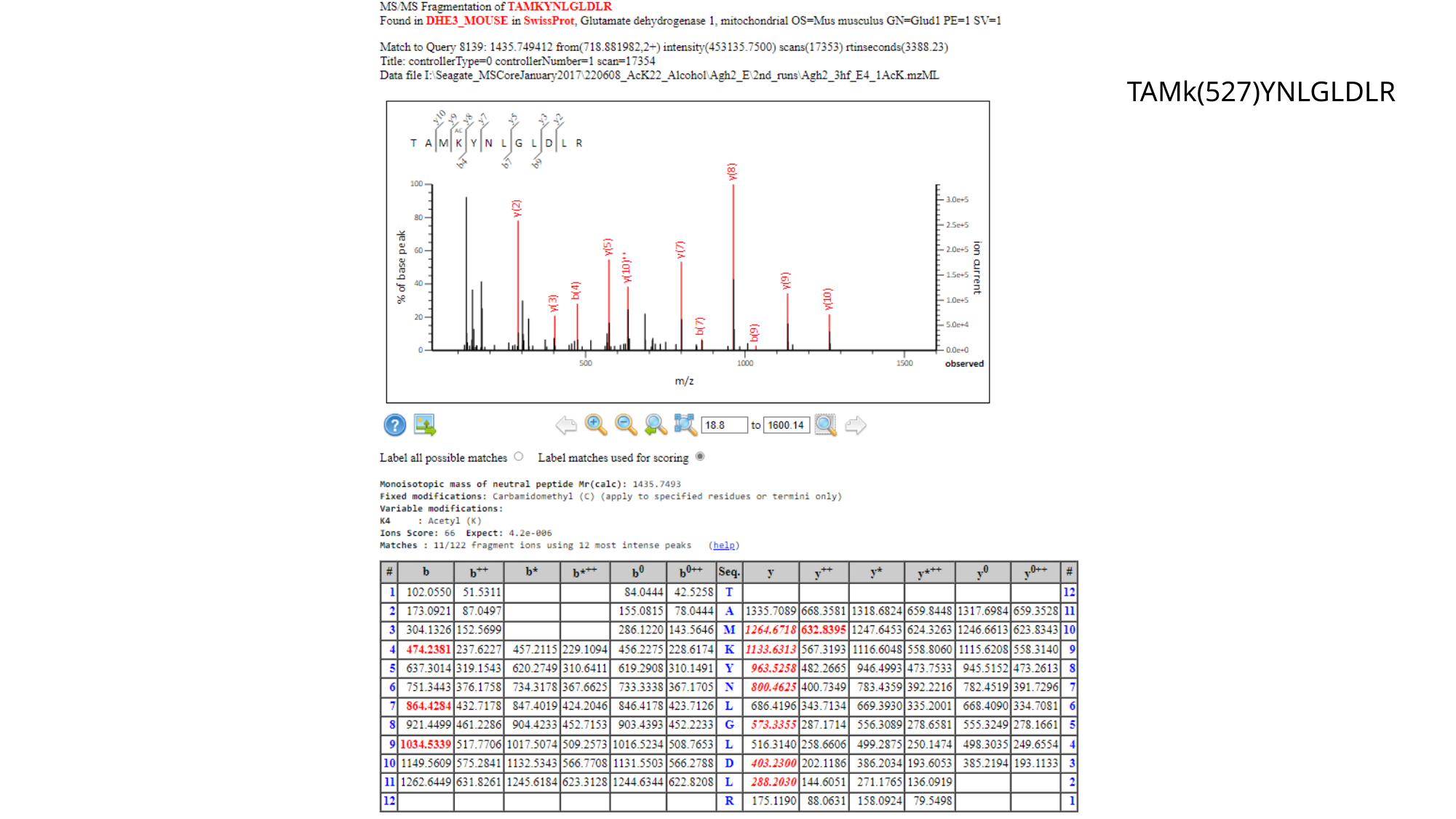

TAMk(527)YNLGLDLR

## Slide 87
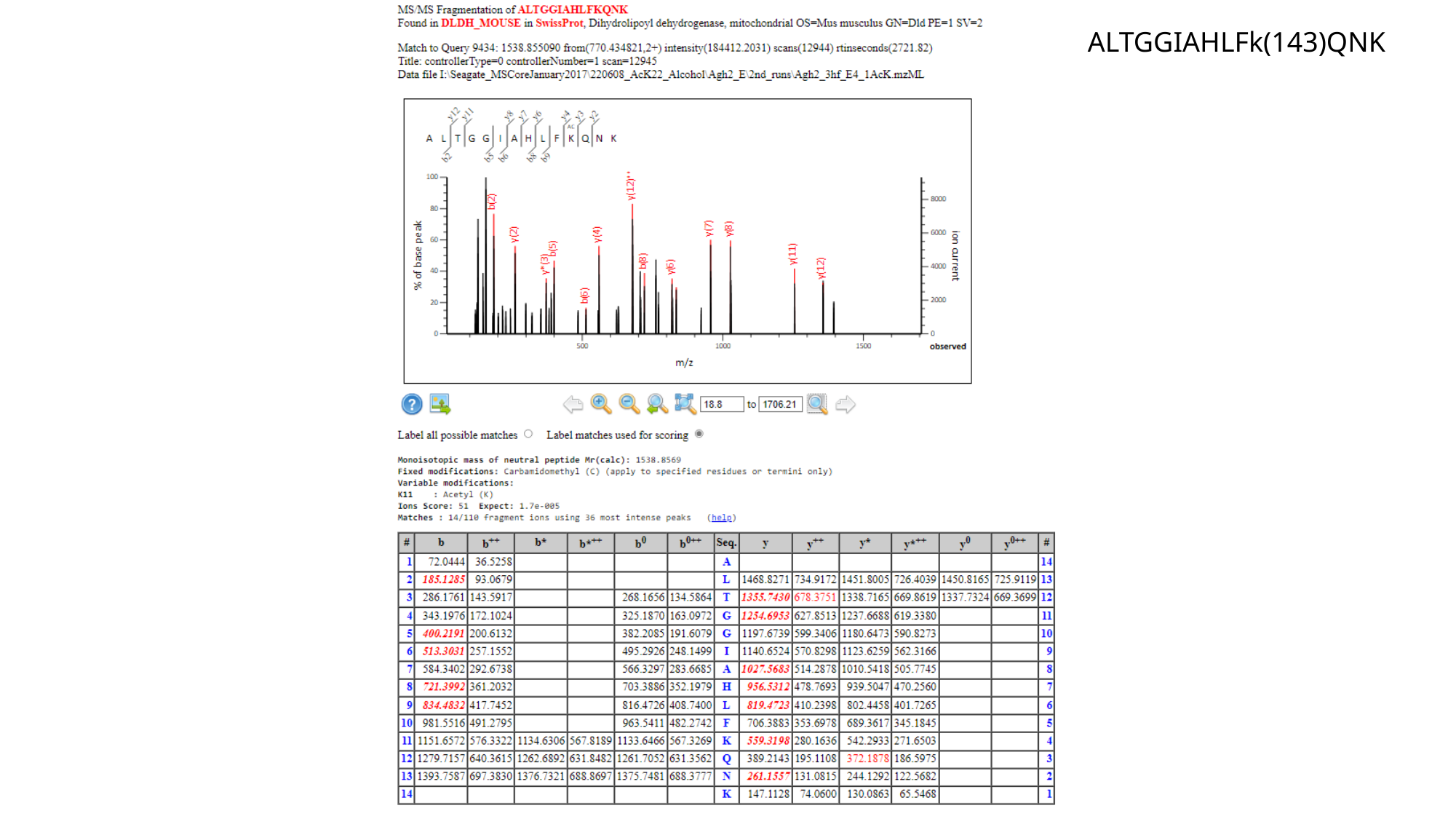

ALTGGIAHLFk(143)QNK

## Slide 88
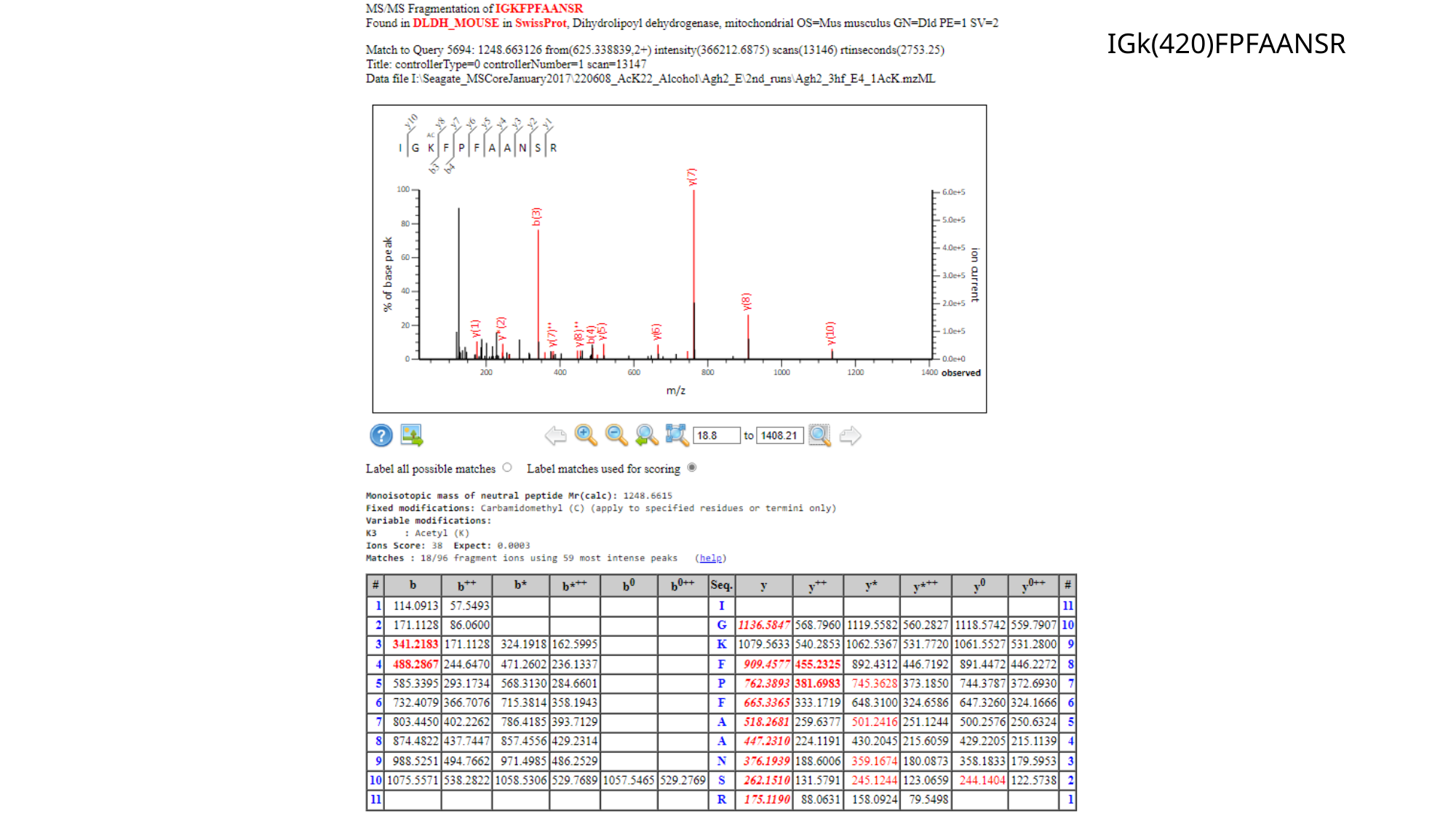

IGk(420)FPFAANSR

## Slide 89
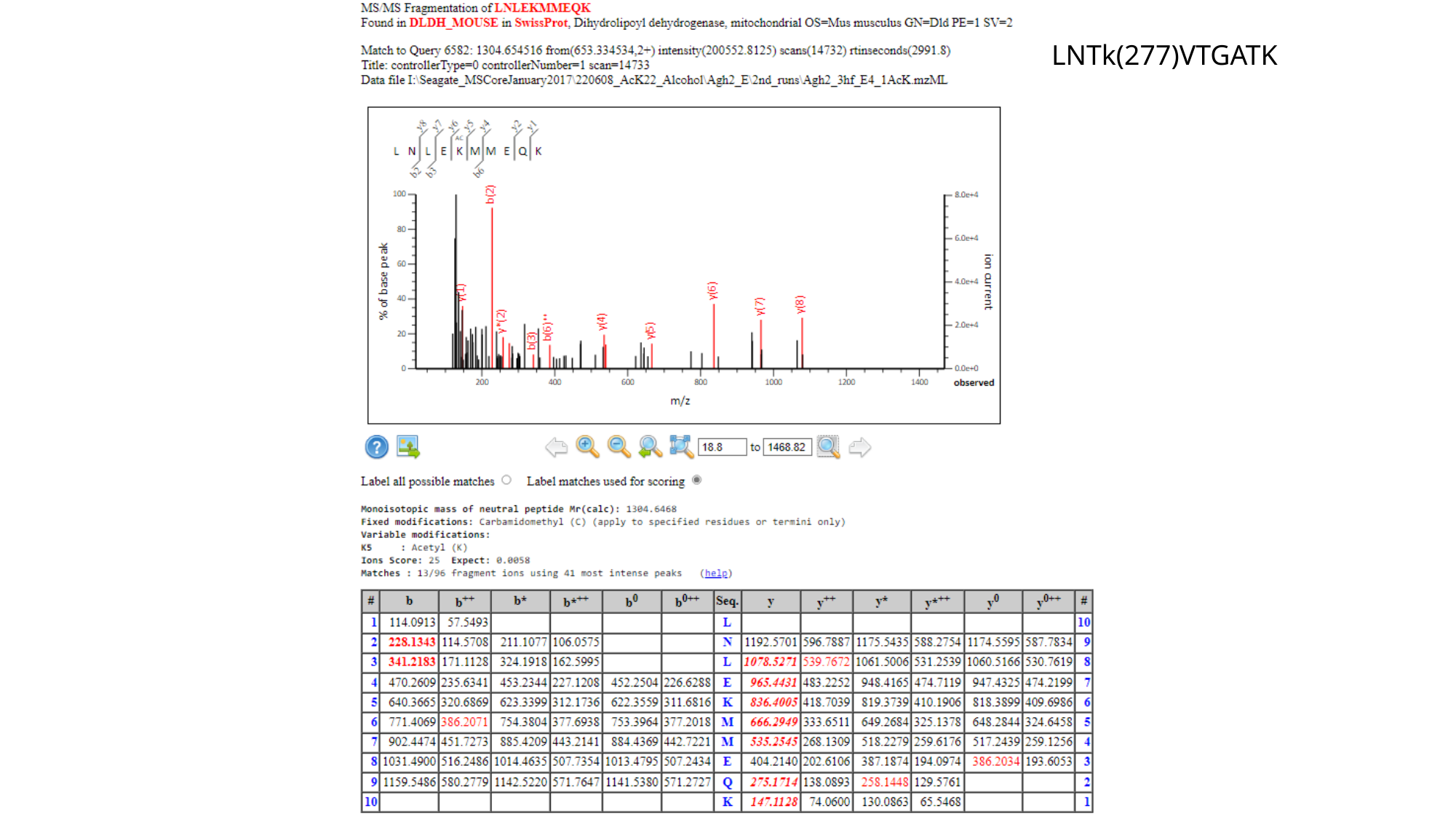

LNTk(277)VTGATK

## Slide 90
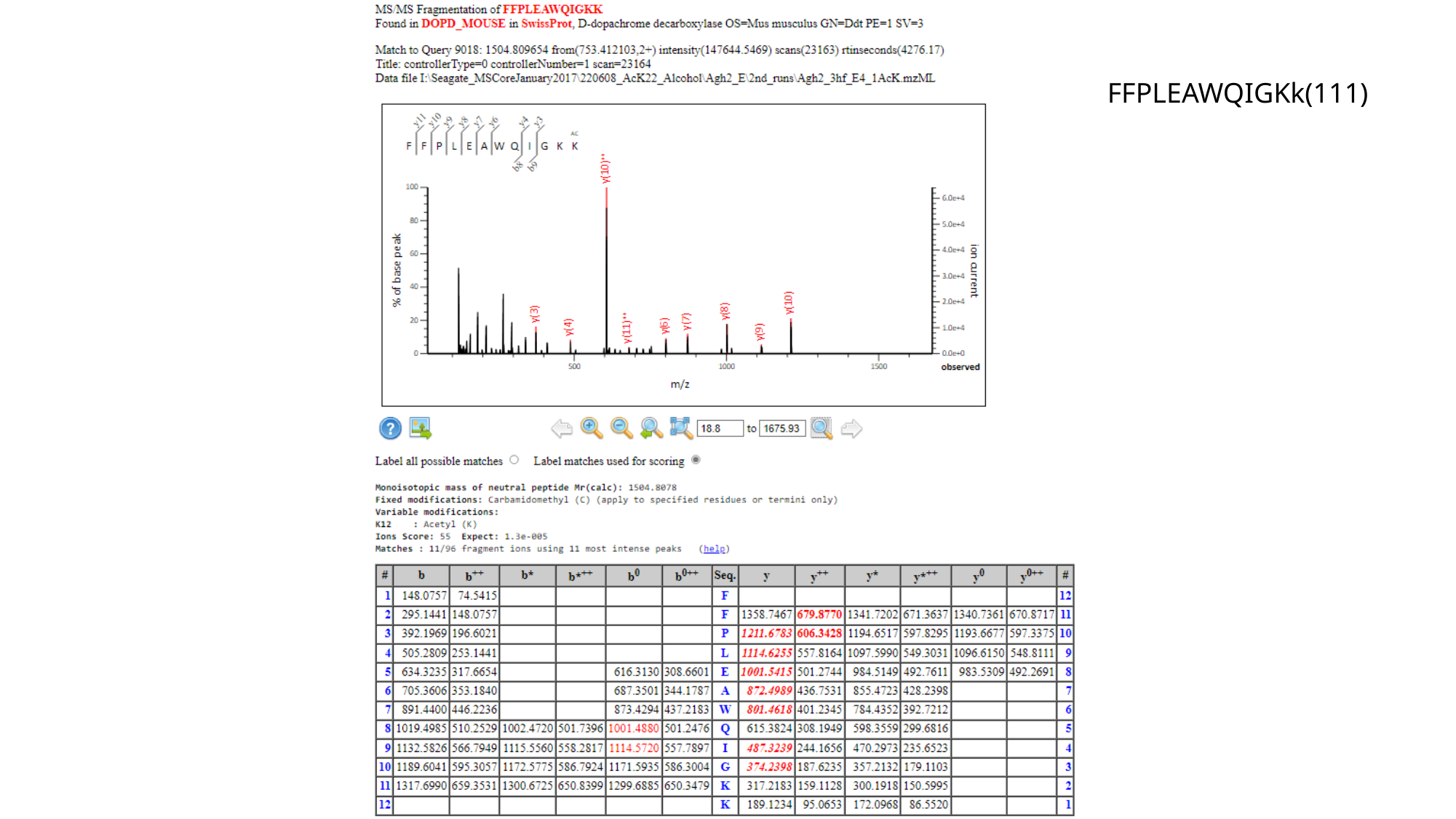

FFPLEAWQIGKk(111)

## Slide 91
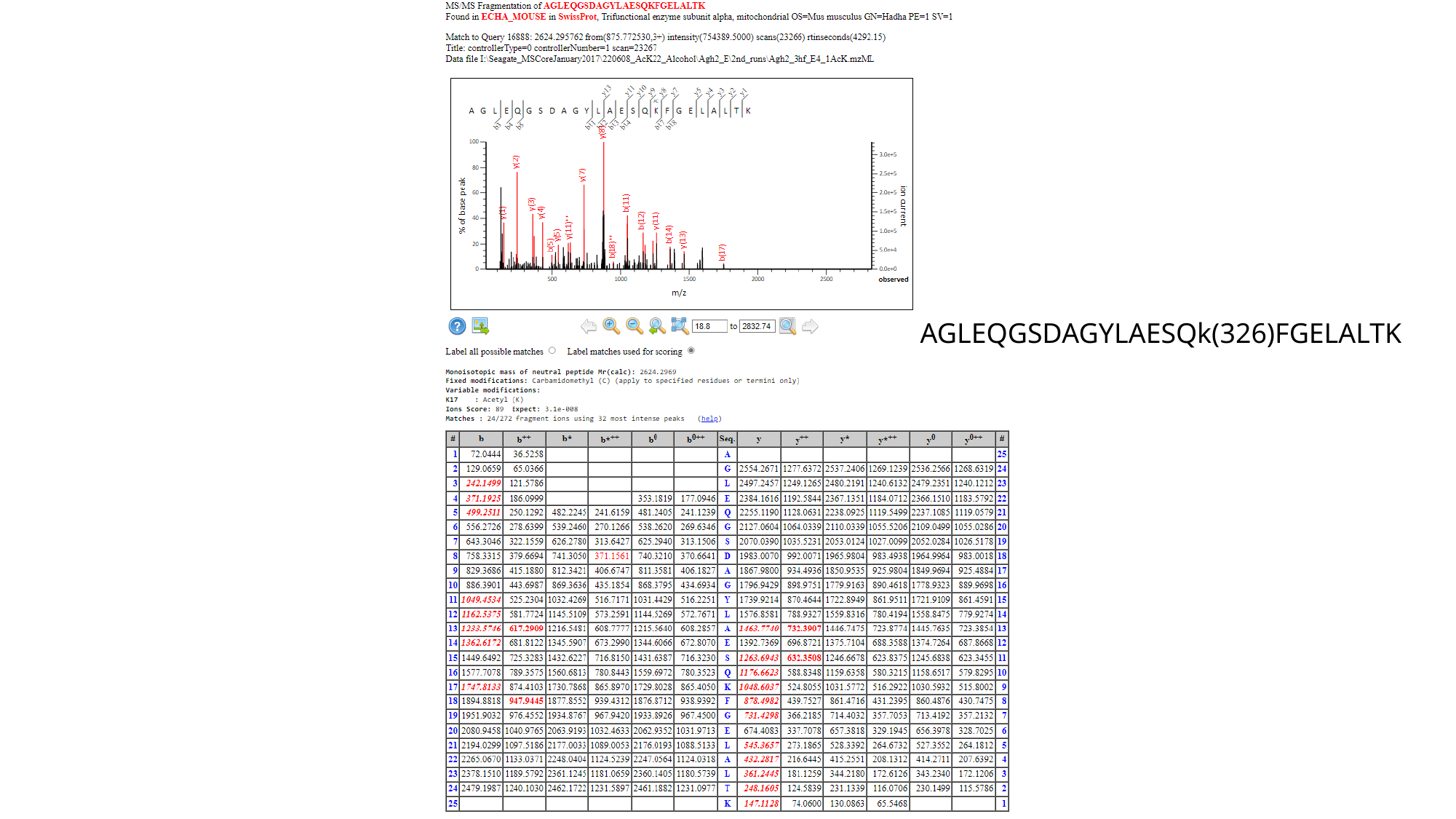

AGLEQGSDAGYLAESQk(326)FGELALTK

## Slide 92
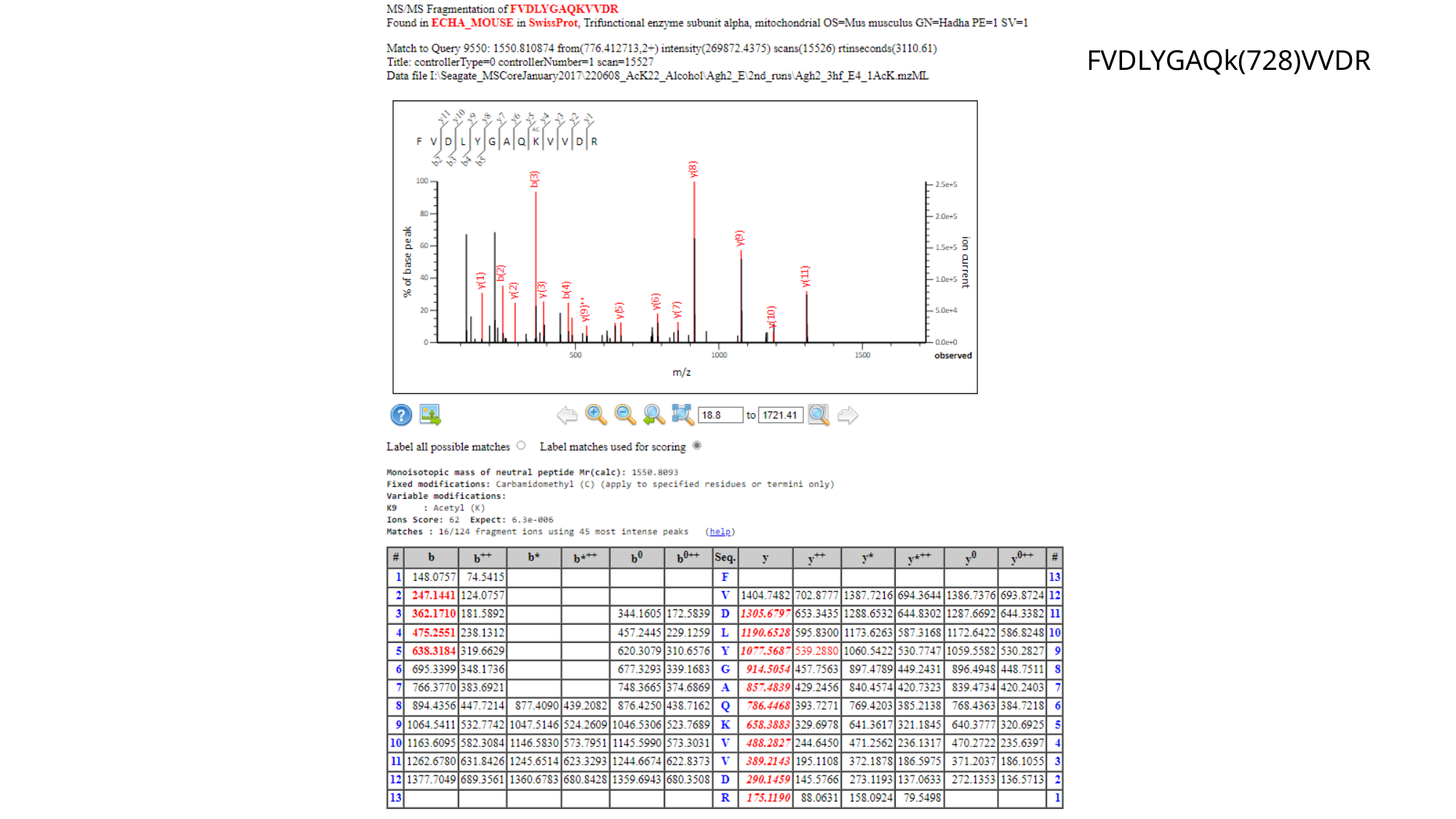

FVDLYGAQk(728)VVDR

## Slide 93
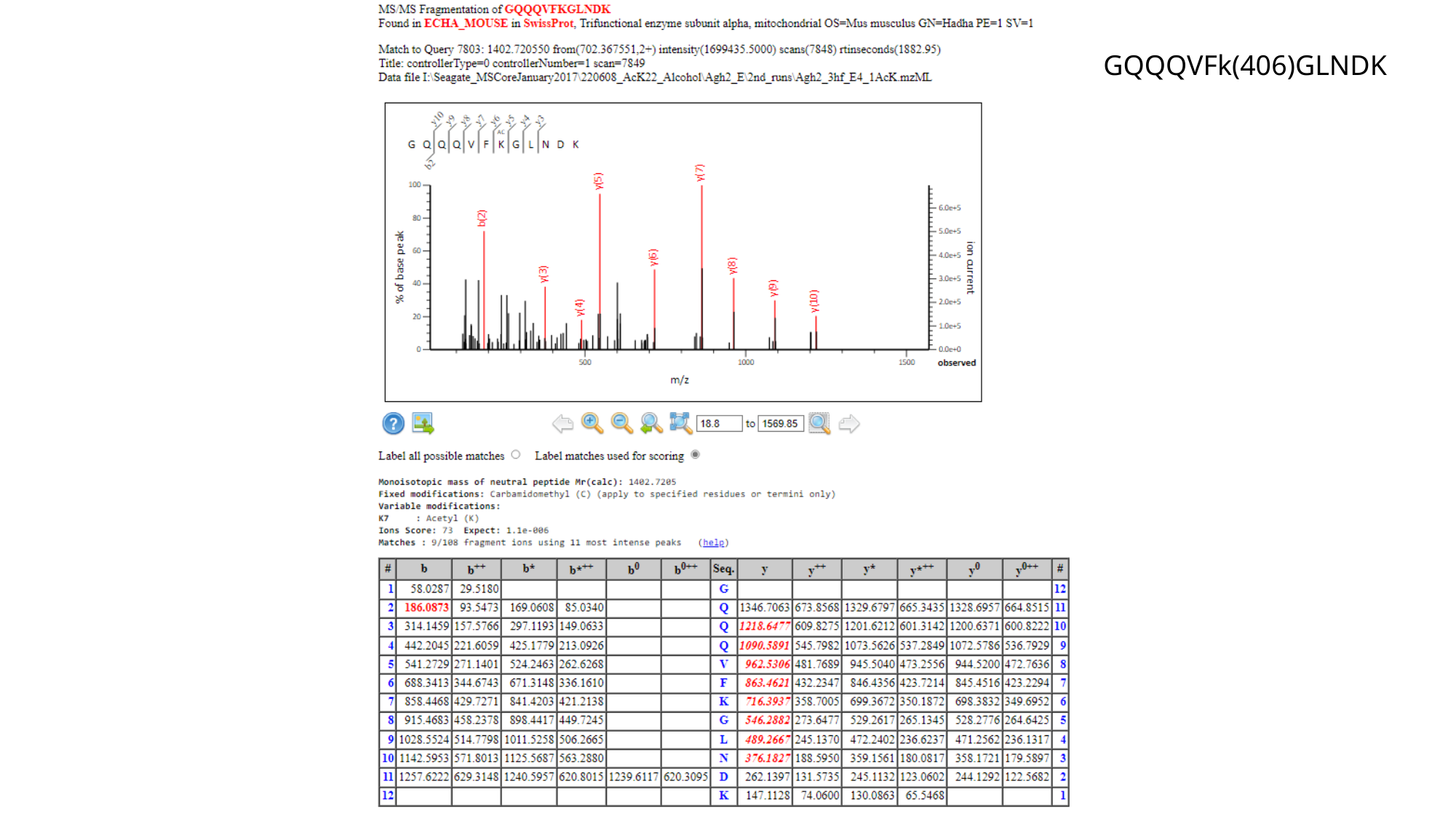

GQQQVFk(406)GLNDK

## Slide 94
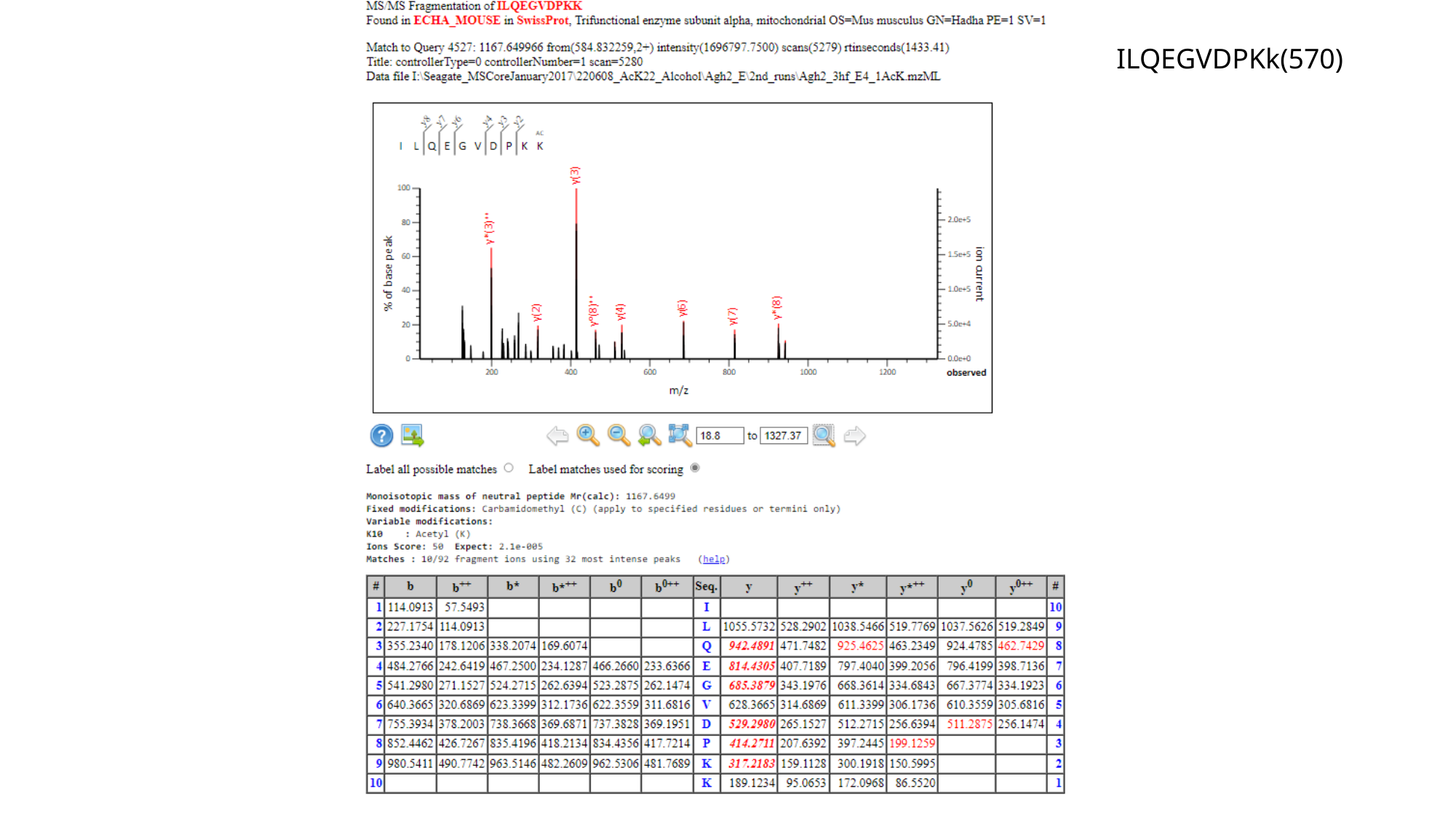

ILQEGVDPKk(570)

## Slide 95
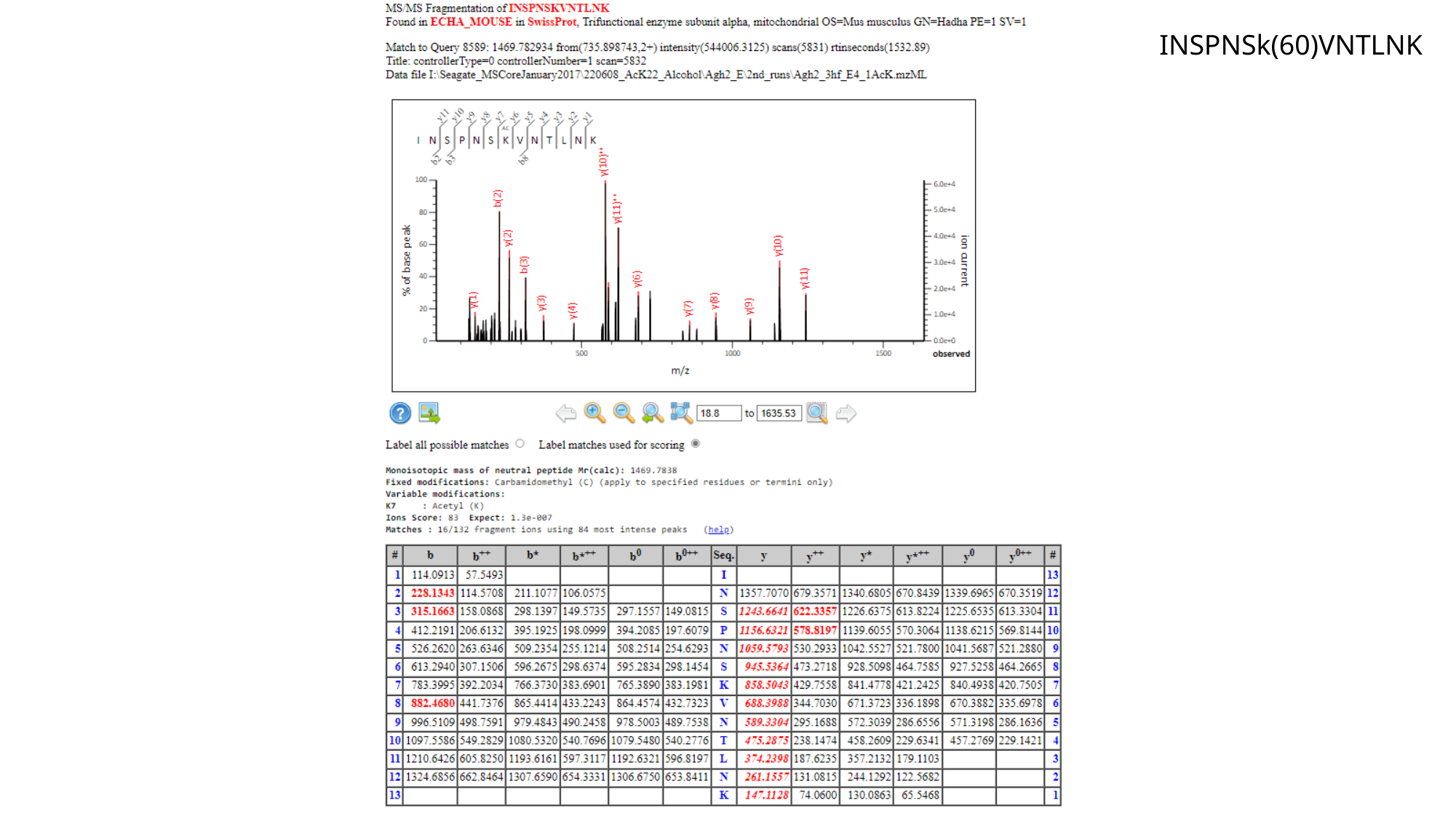

INSPNSk(60)VNTLNK

## Slide 96
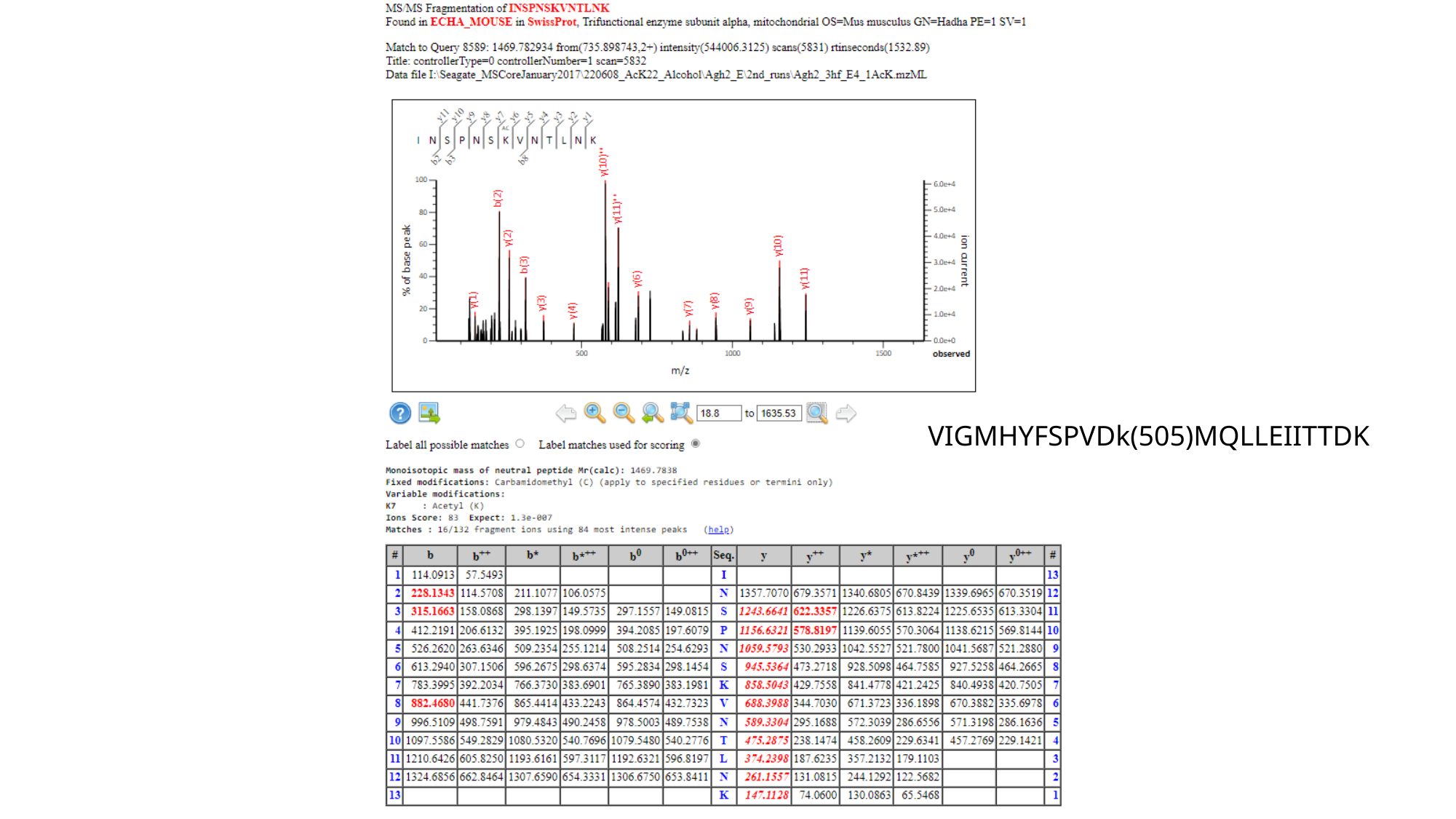

VIGMHYFSPVDk(505)MQLLEIITTDK

## Slide 97
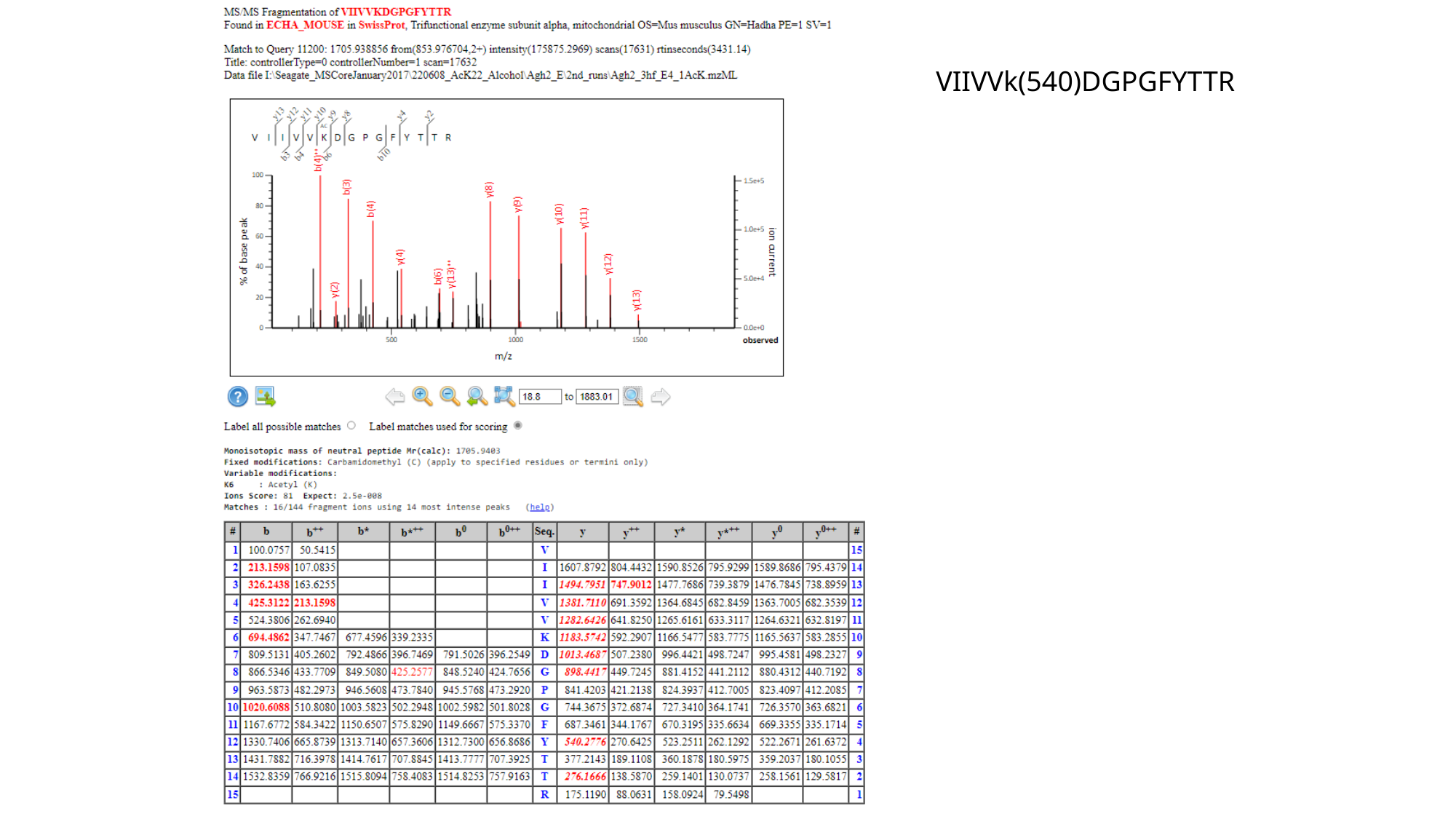

VIIVVk(540)DGPGFYTTR

## Slide 98
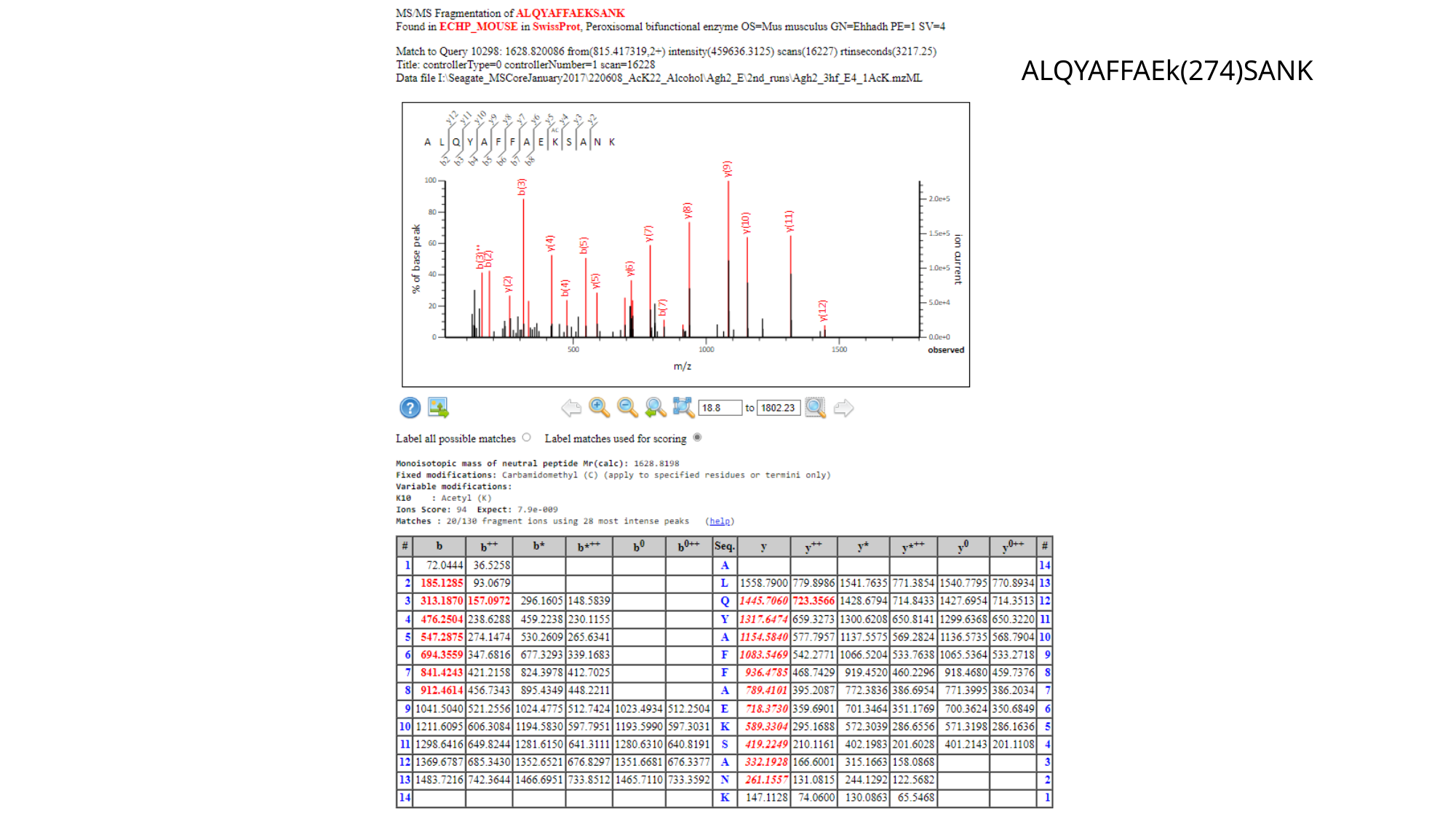

ALQYAFFAEk(274)SANK

## Slide 99
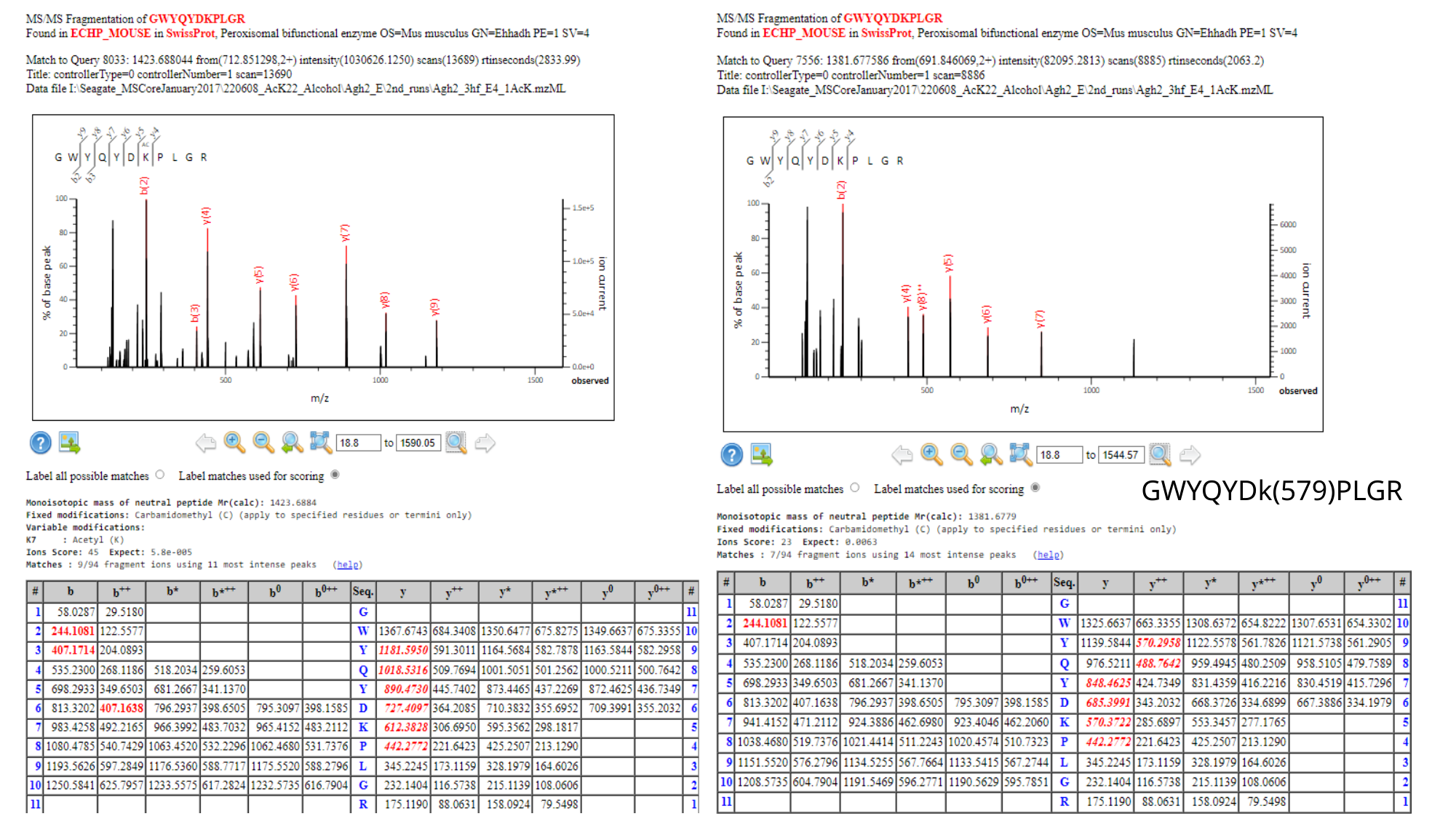

GWYQYDk(579)PLGR

## Slide 100
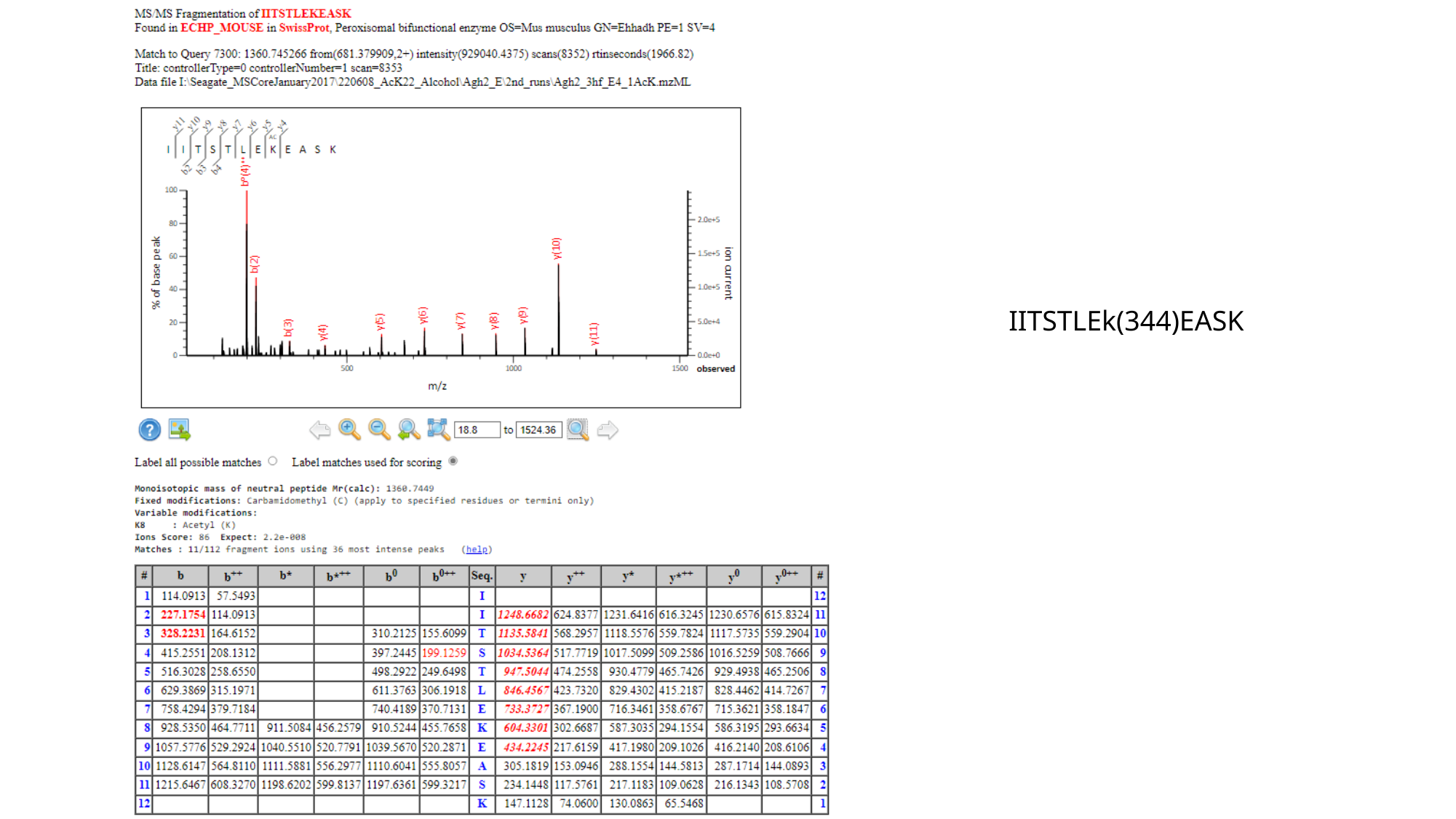

IITSTLEk(344)EASK

## Slide 101
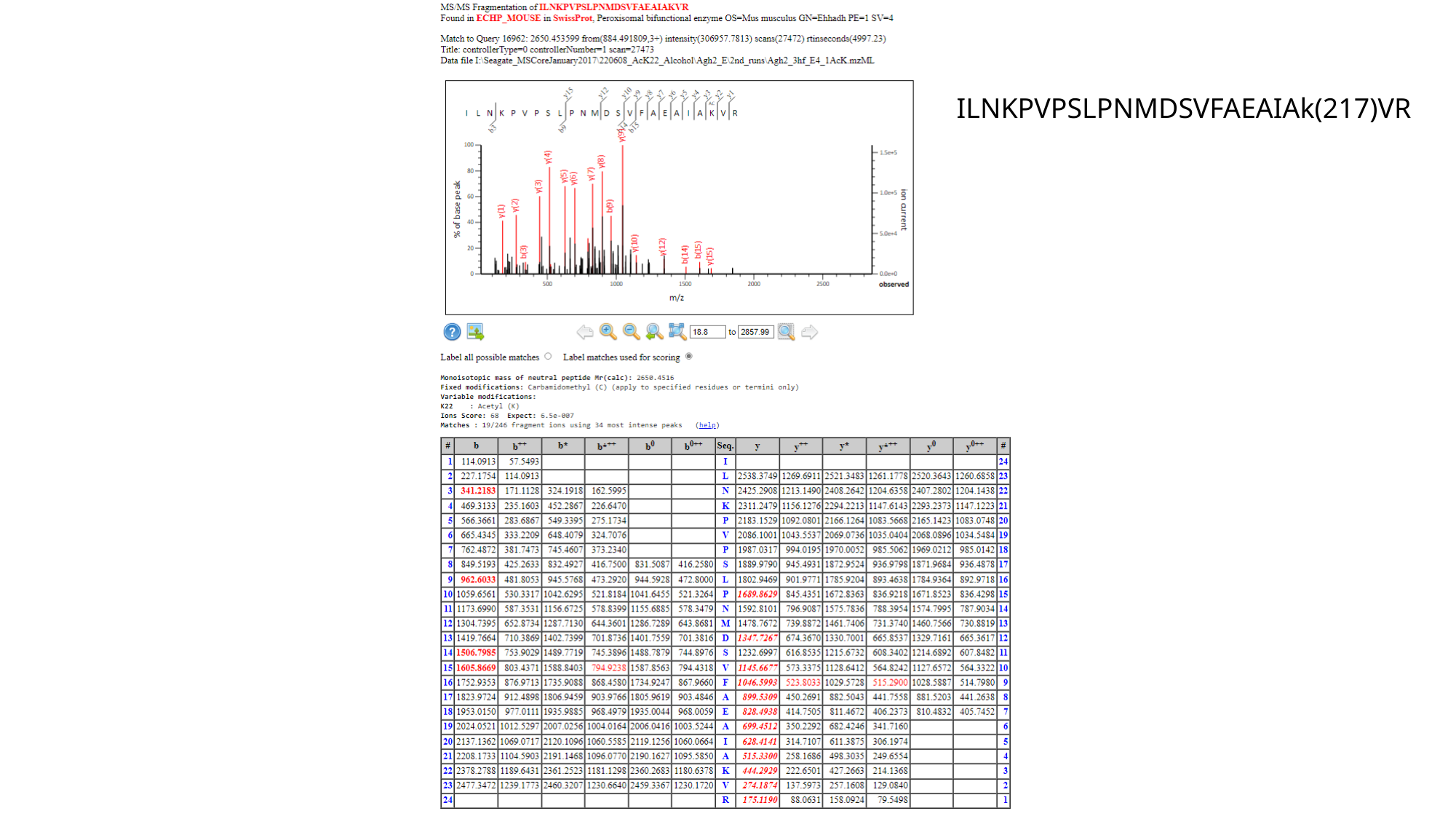

ILNKPVPSLPNMDSVFAEAIAk(217)VR

## Slide 102
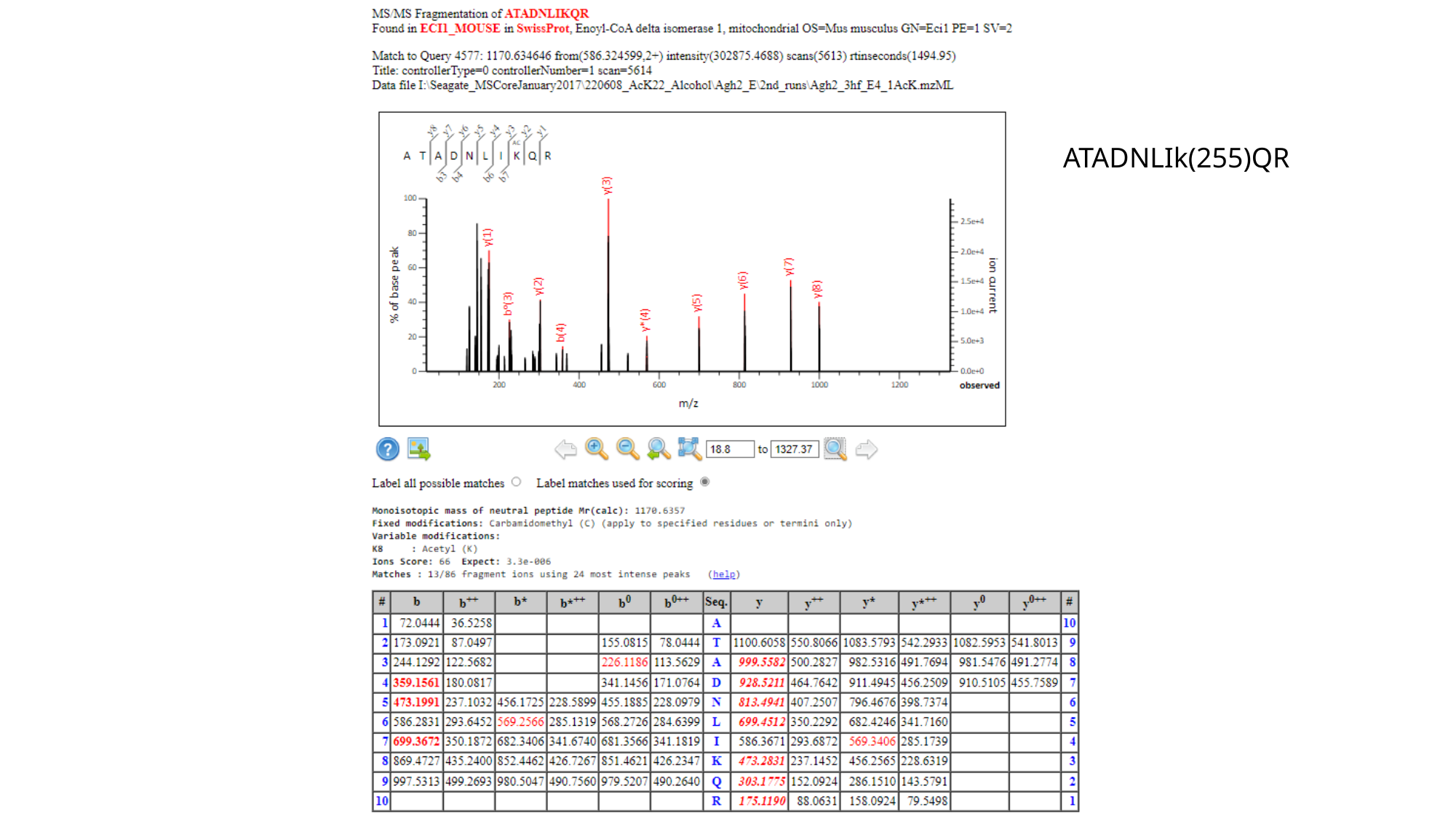

ATADNLIk(255)QR

## Slide 103
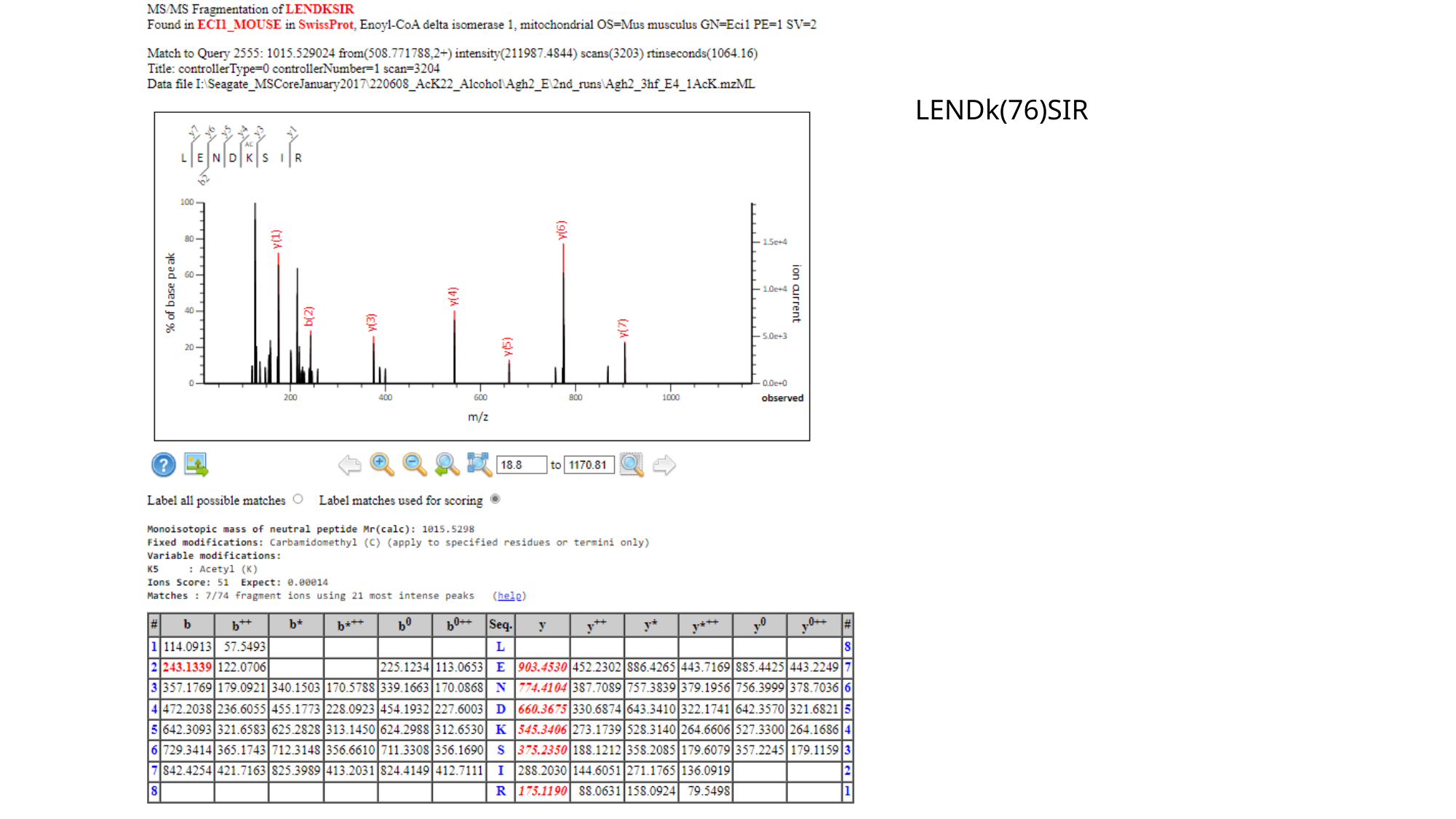

LENDk(76)SIR

## Slide 104
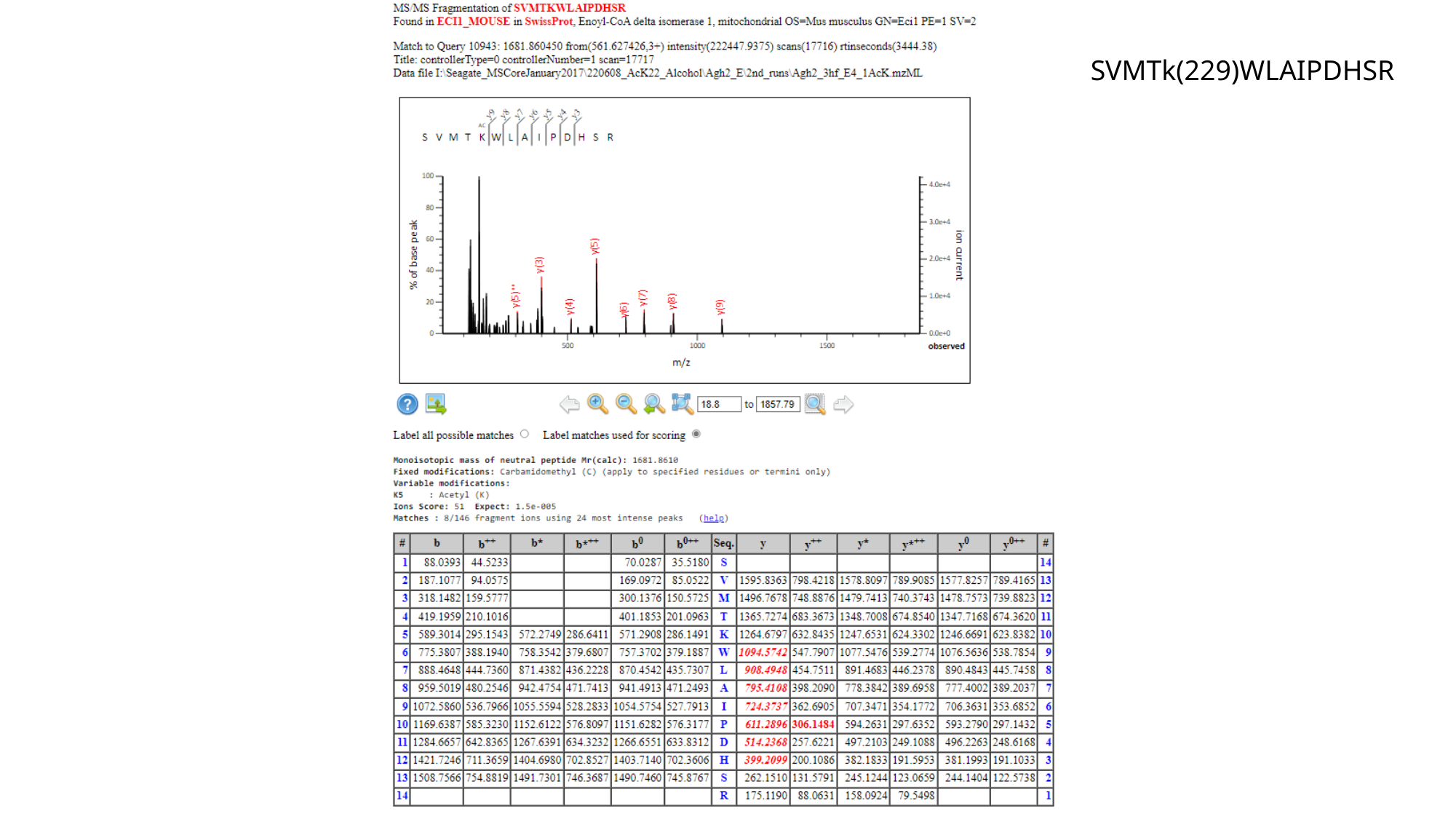

SVMTk(229)WLAIPDHSR

## Slide 105
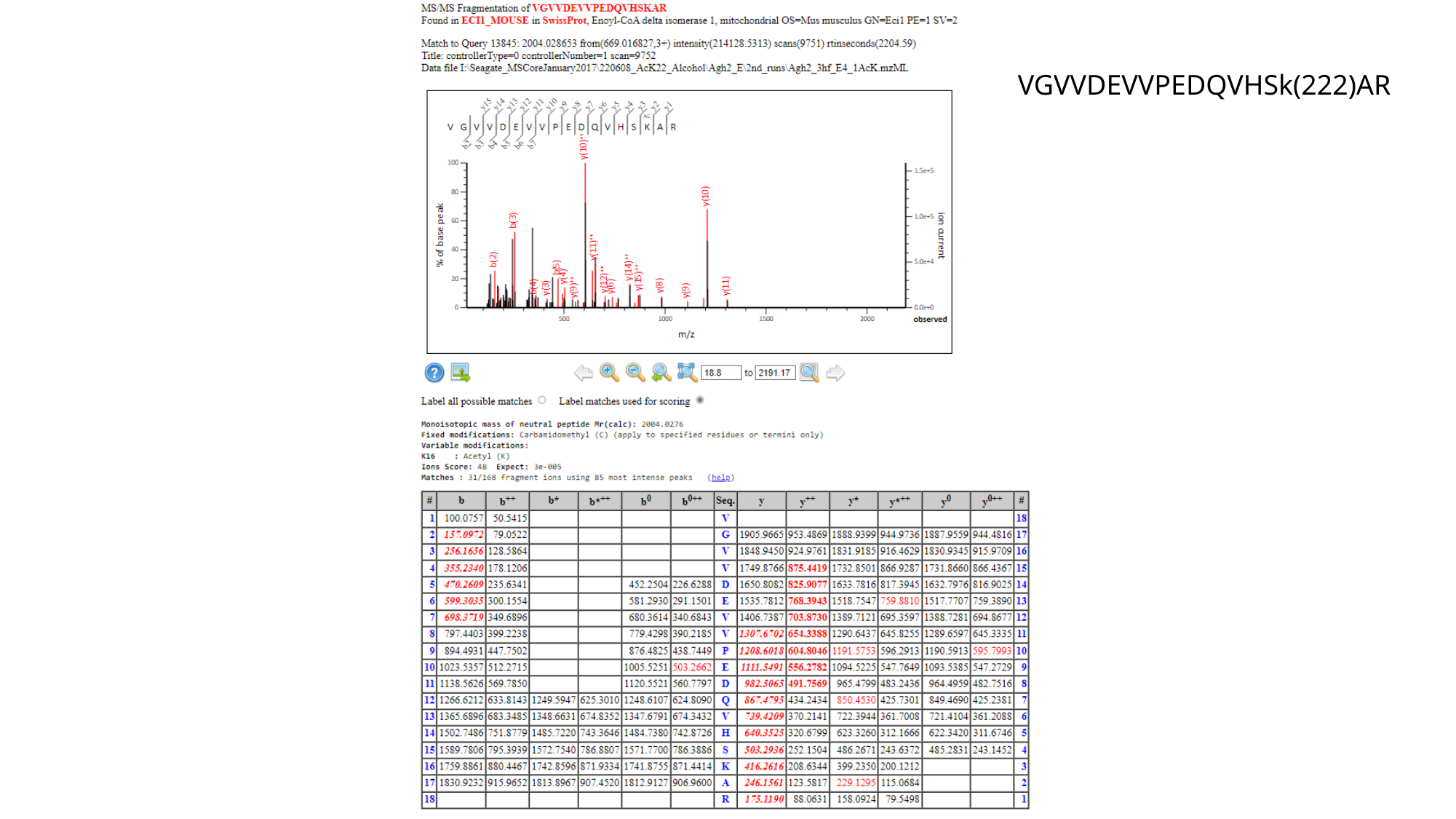

VGVVDEVVPEDQVHSk(222)AR

## Slide 106
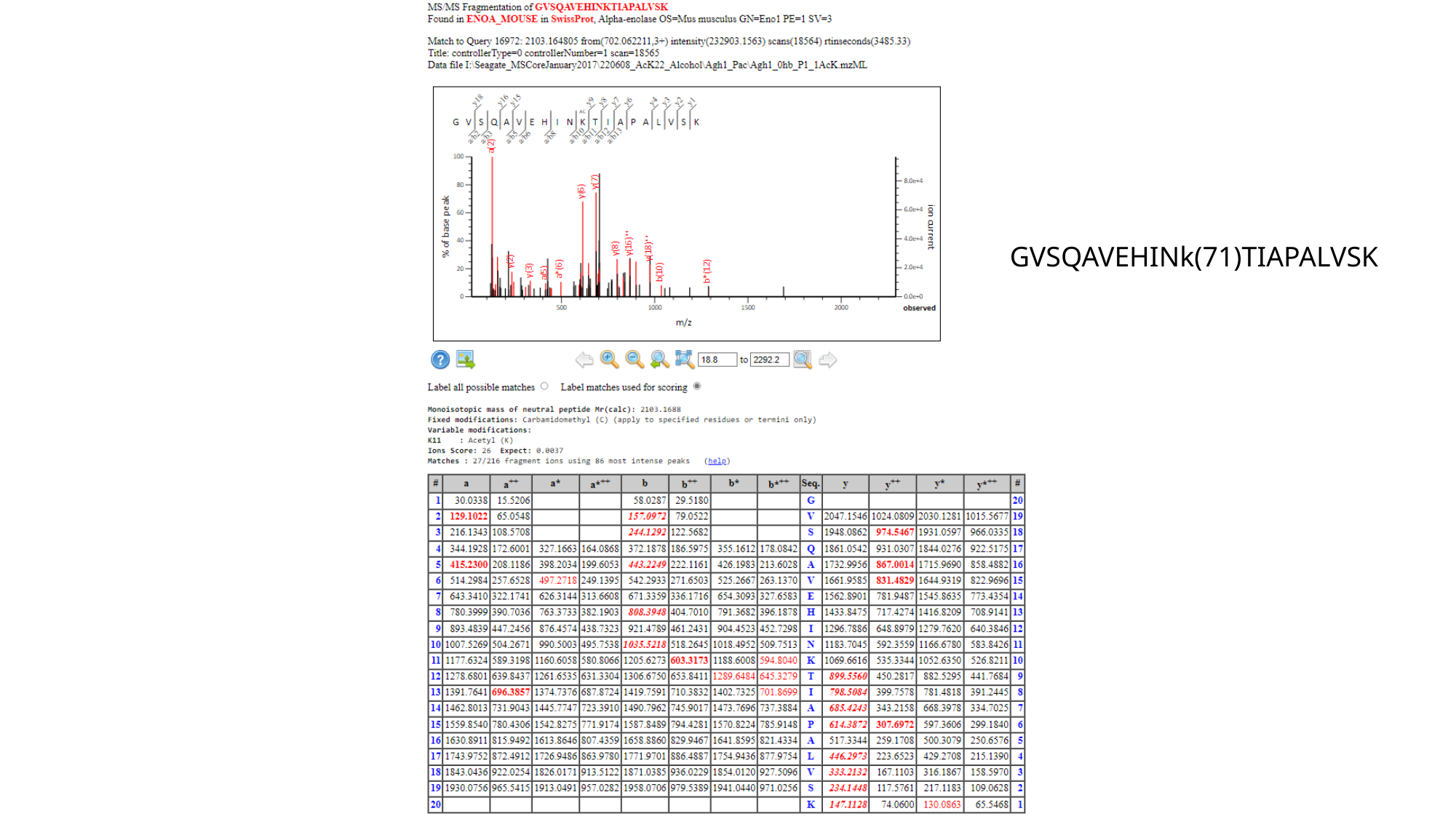

GVSQAVEHINk(71)TIAPALVSK

## Slide 107
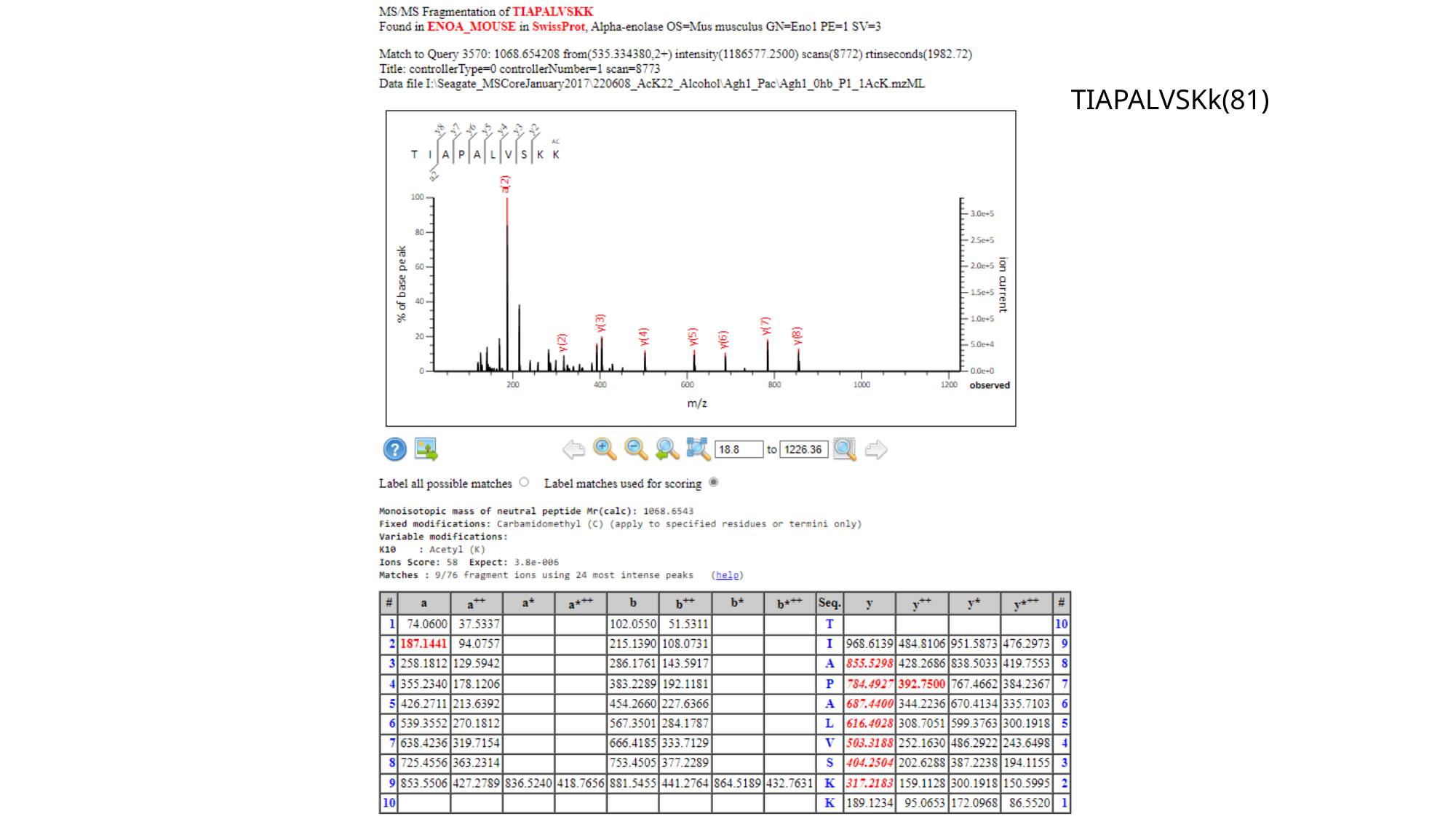

TIAPALVSKk(81)

## Slide 108
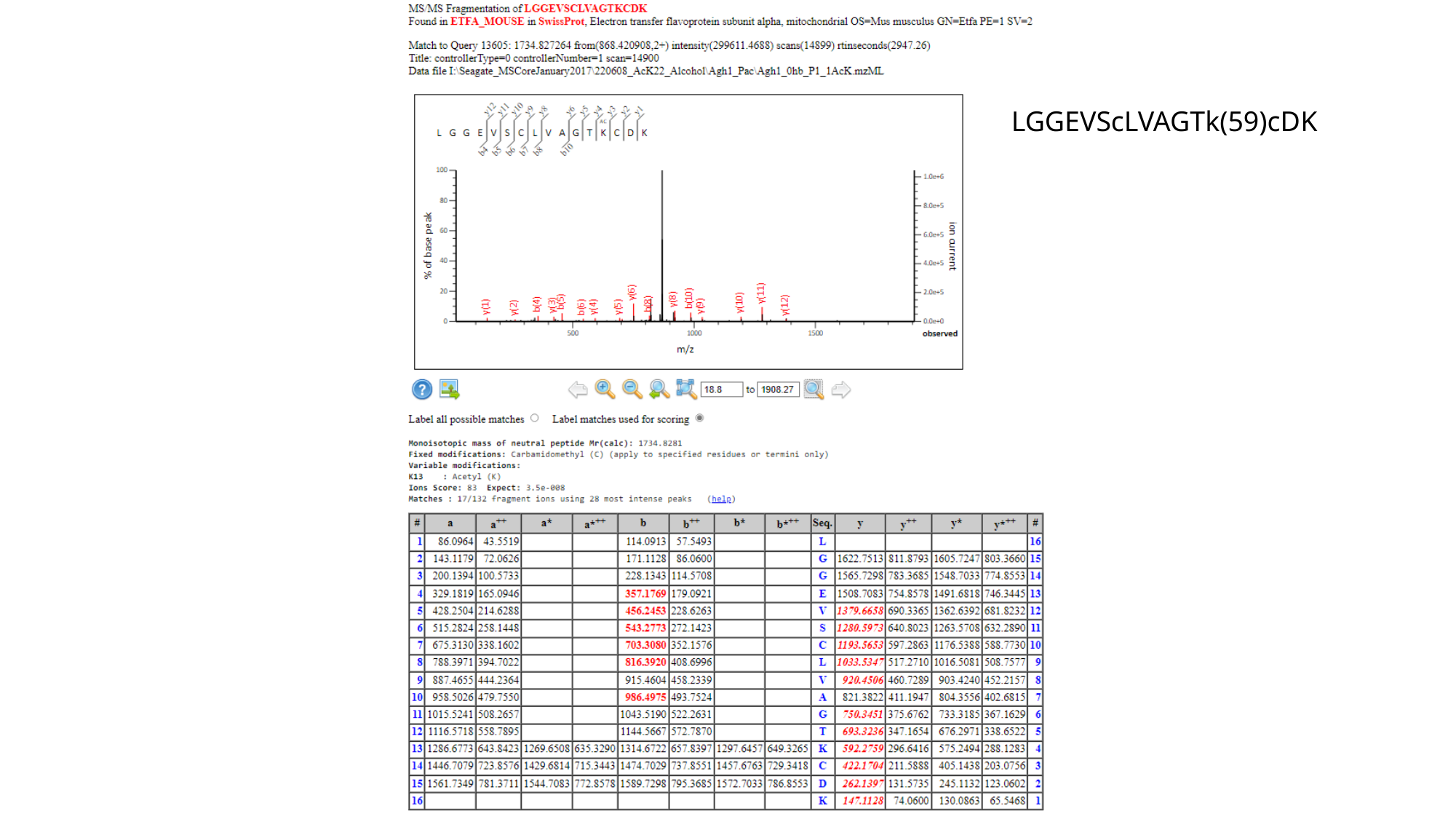

LGGEVScLVAGTk(59)cDK

## Slide 109
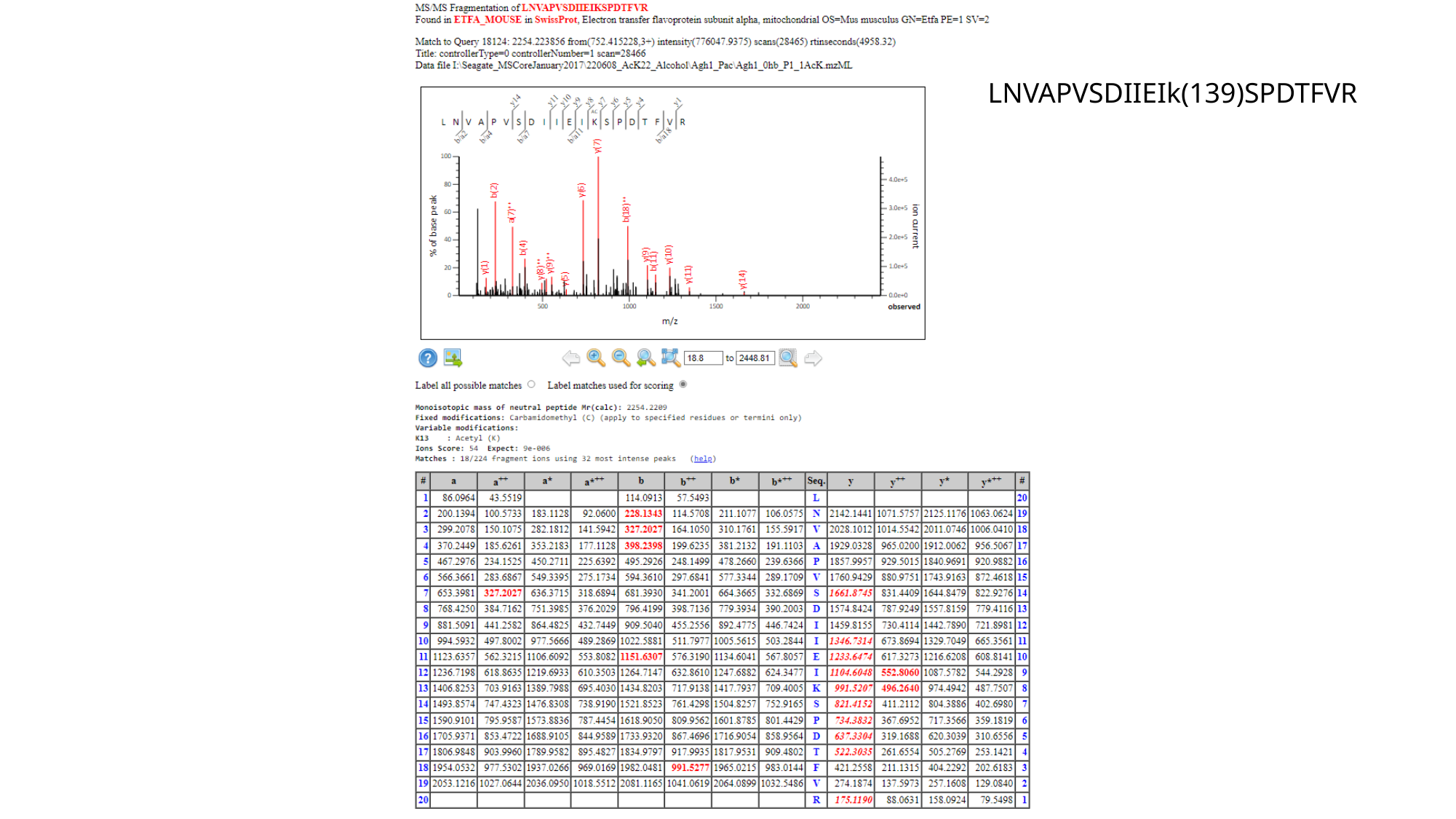

LNVAPVSDIIEIk(139)SPDTFVR

## Slide 110
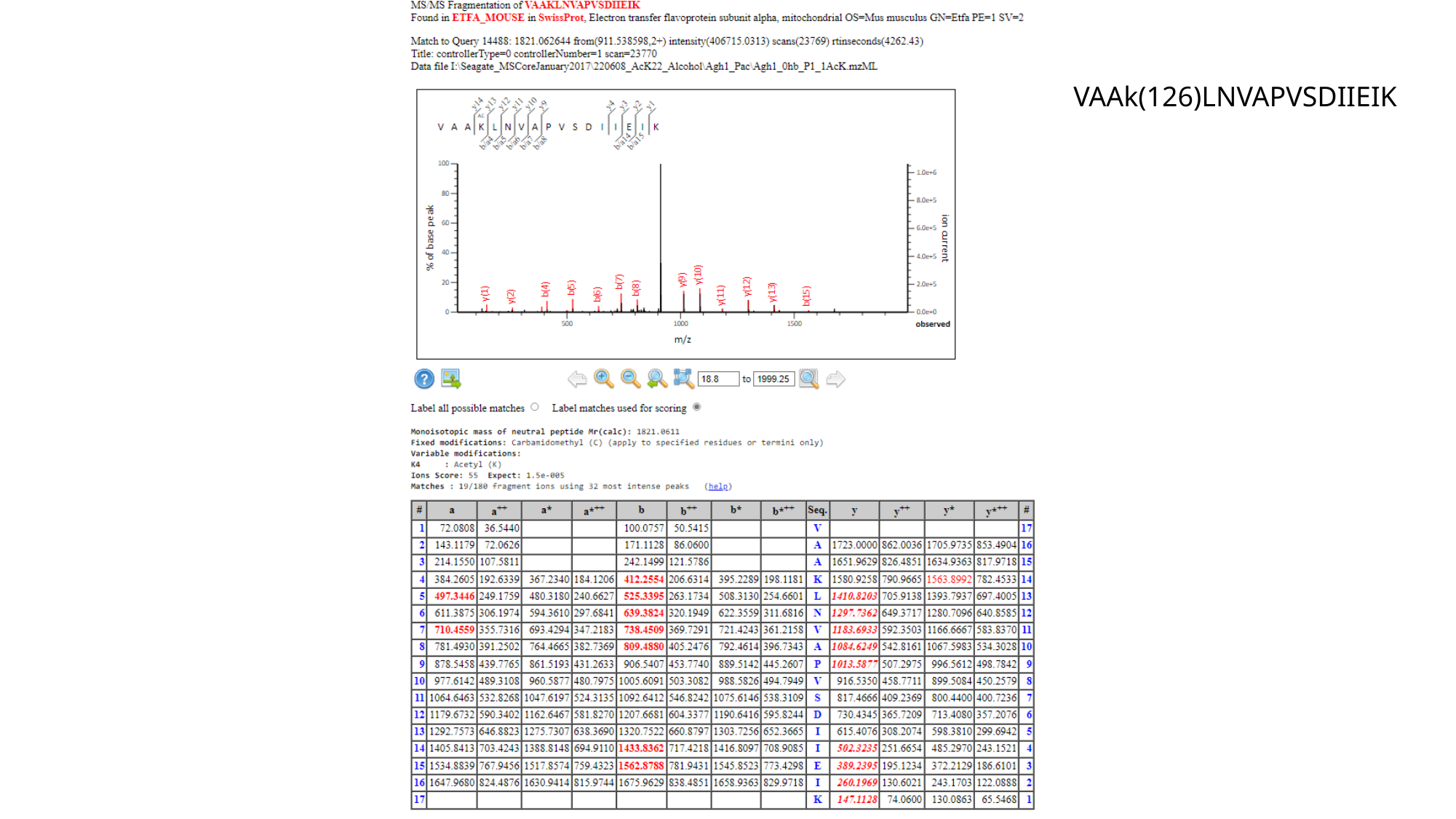

VAAk(126)LNVAPVSDIIEIK

## Slide 111
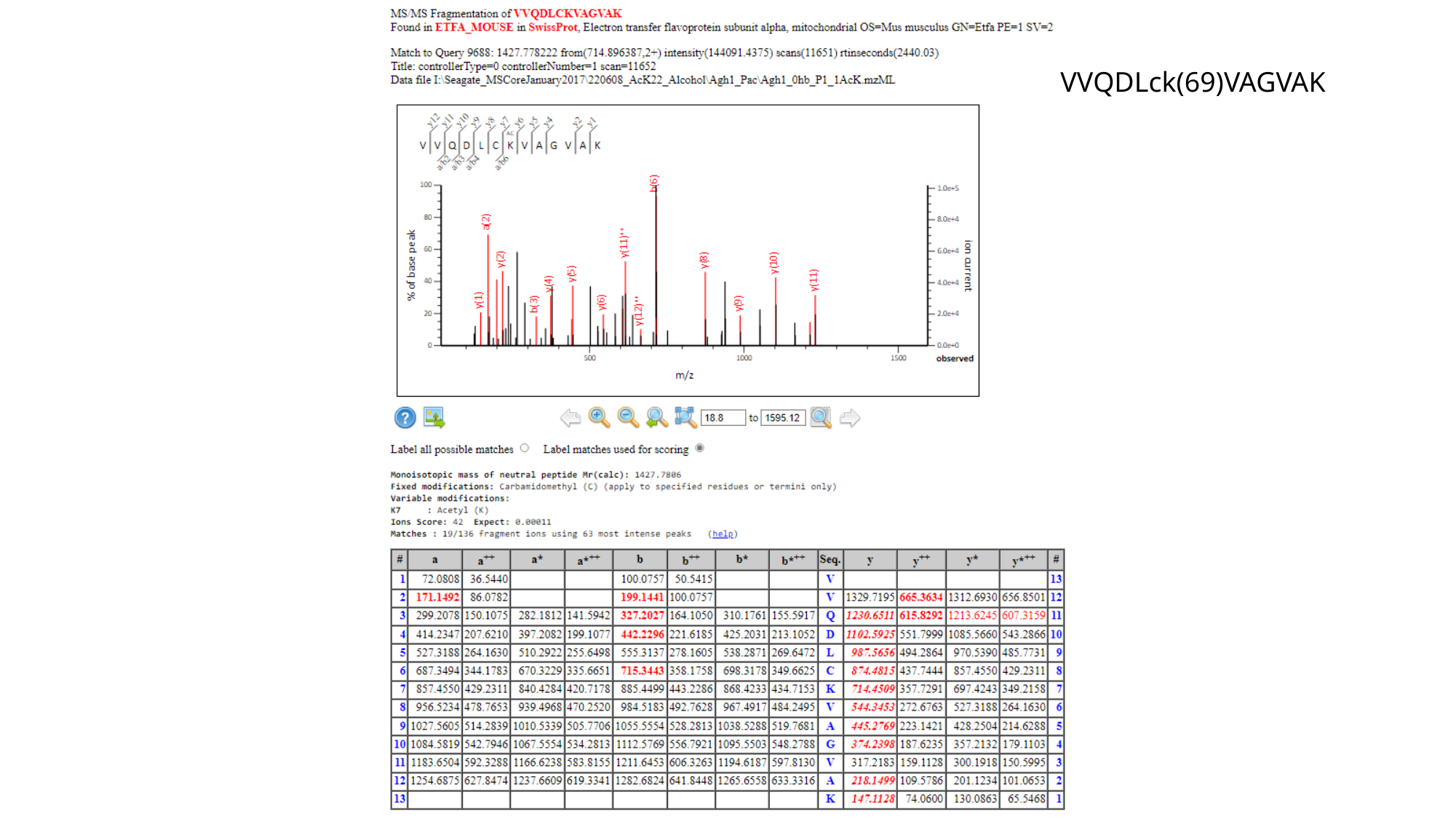

VVQDLck(69)VAGVAK

## Slide 112
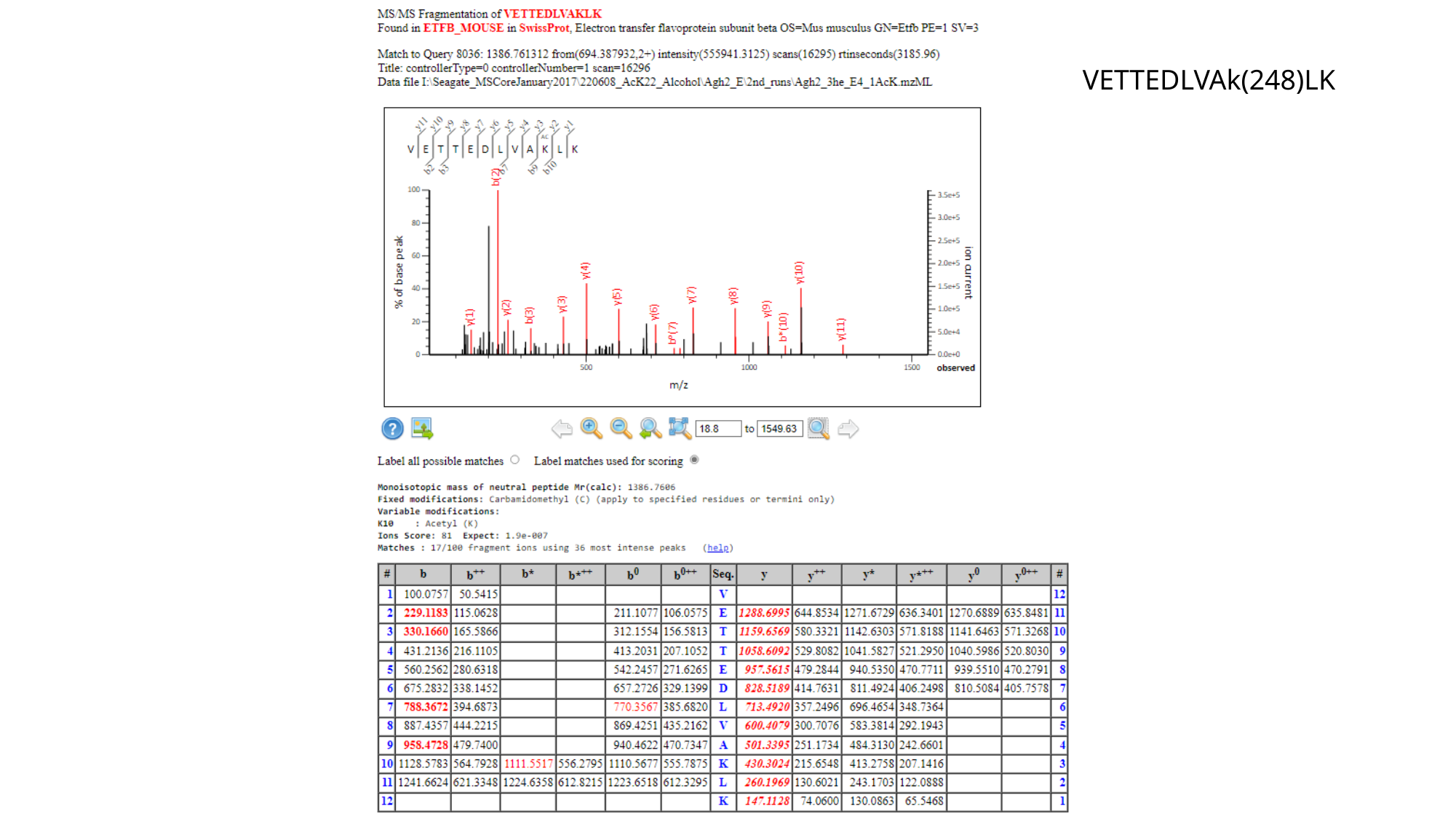

VETTEDLVAk(248)LK

## Slide 113
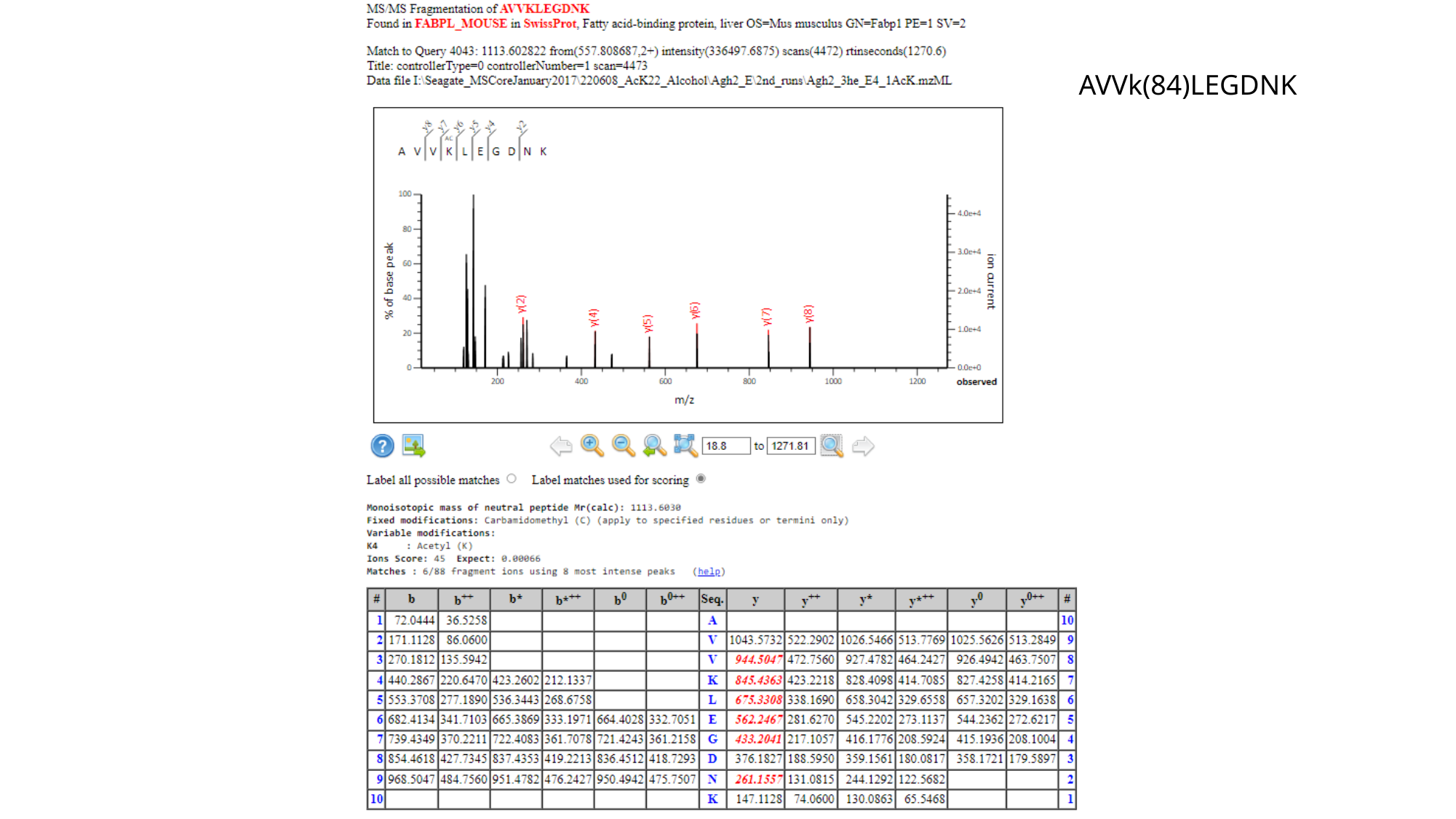

AVVk(84)LEGDNK

## Slide 114
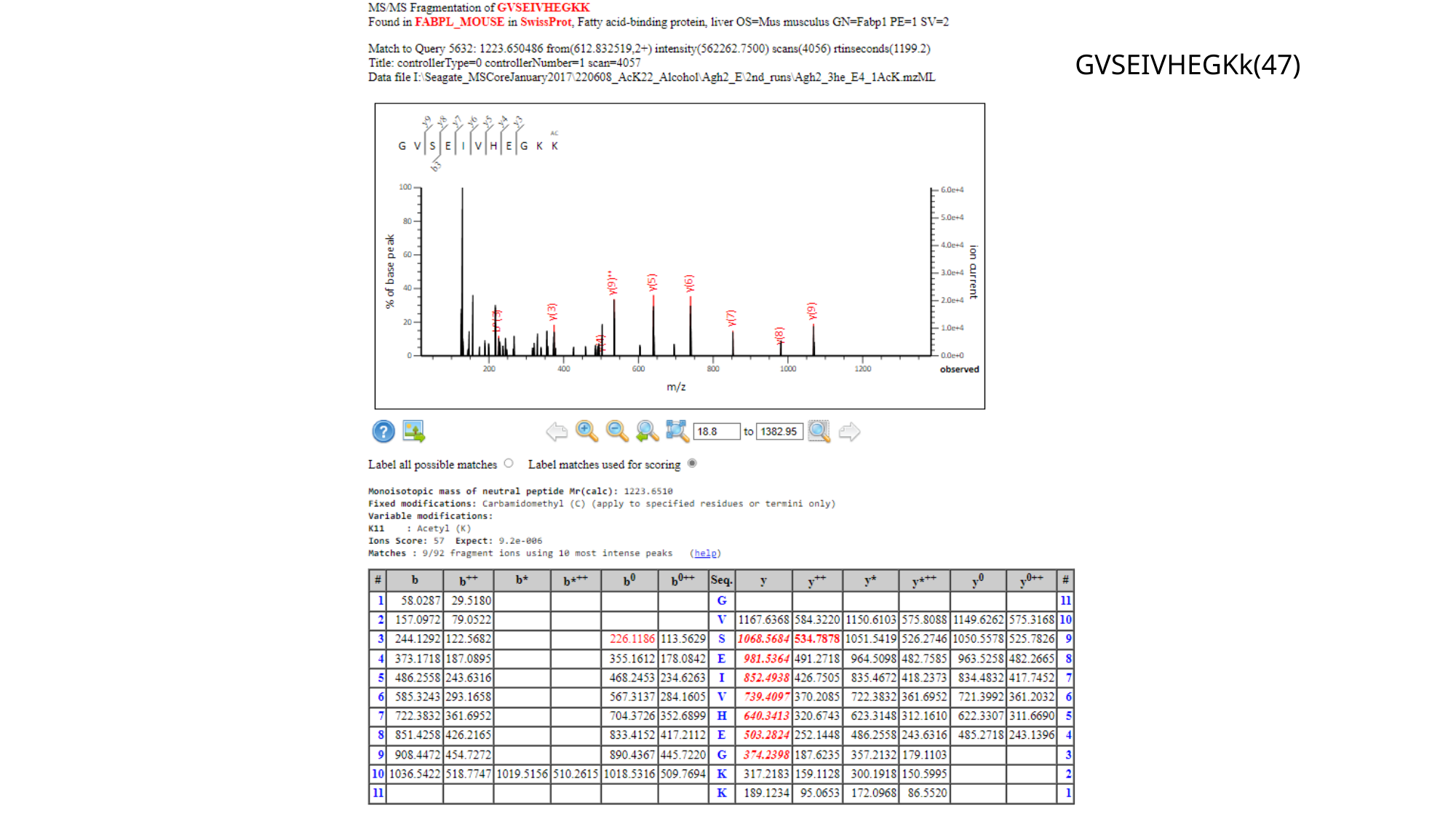

GVSEIVHEGKk(47)

## Slide 115
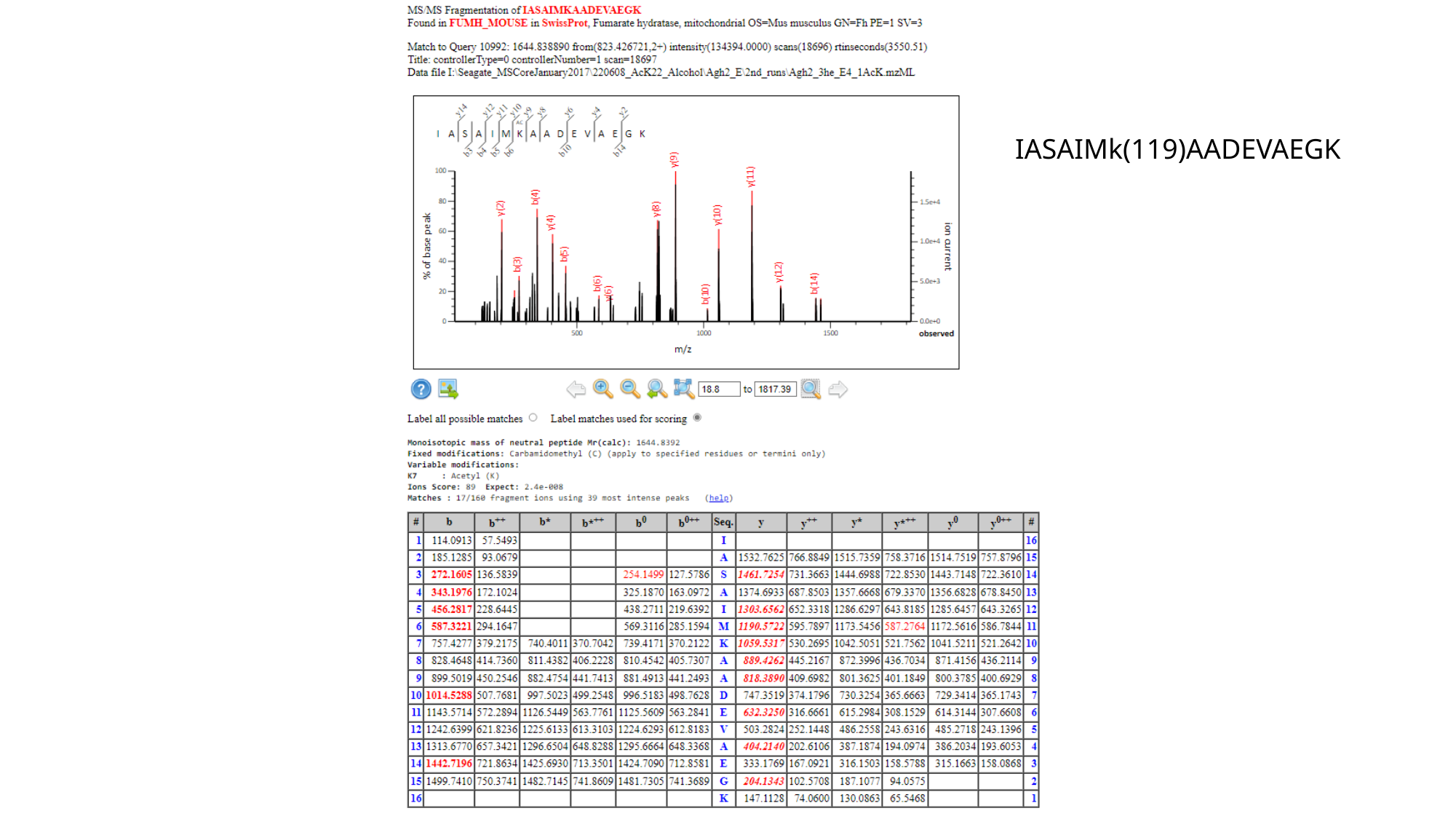

IASAIMk(119)AADEVAEGK

## Slide 116
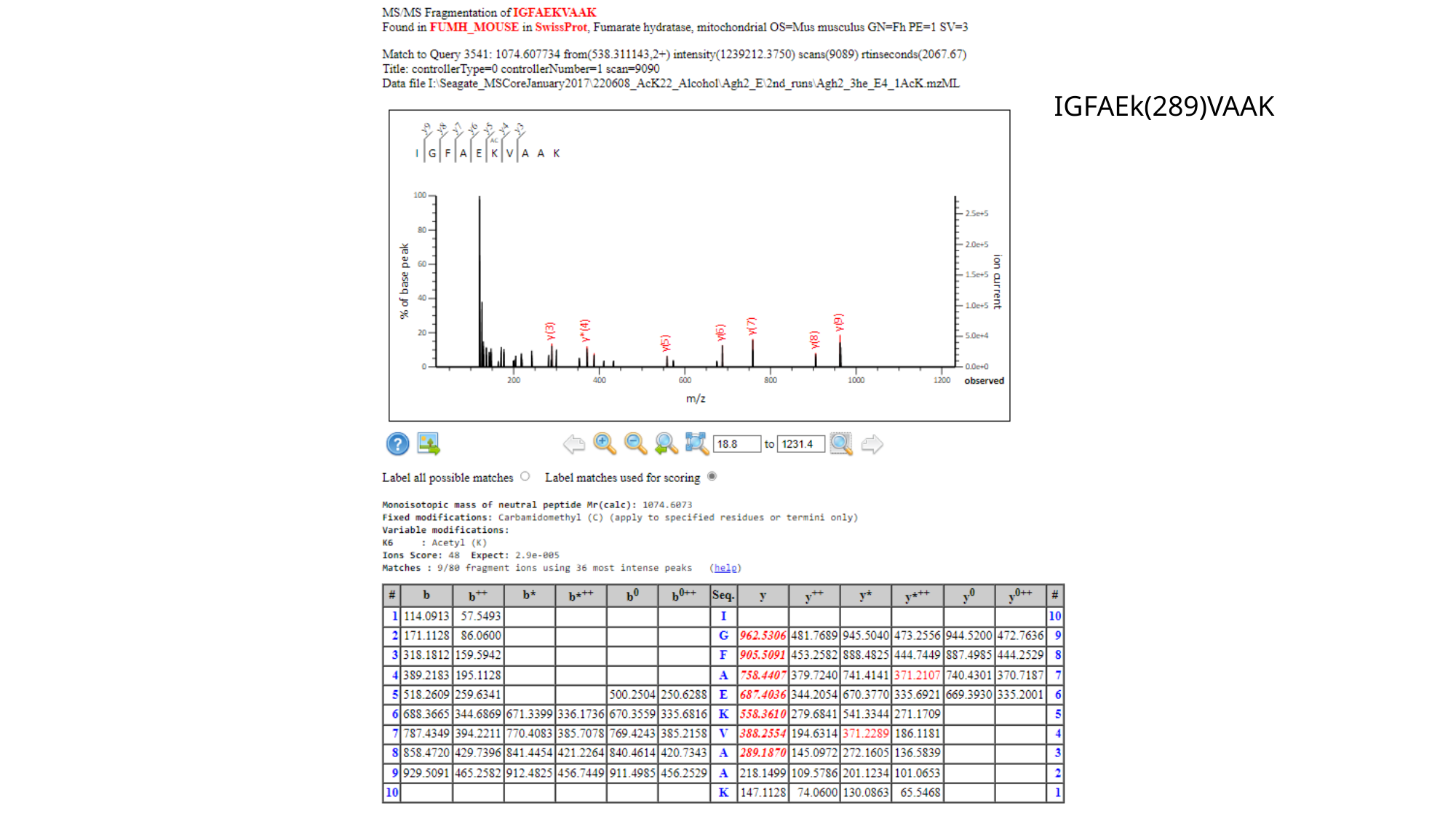

IGFAEk(289)VAAK

## Slide 117
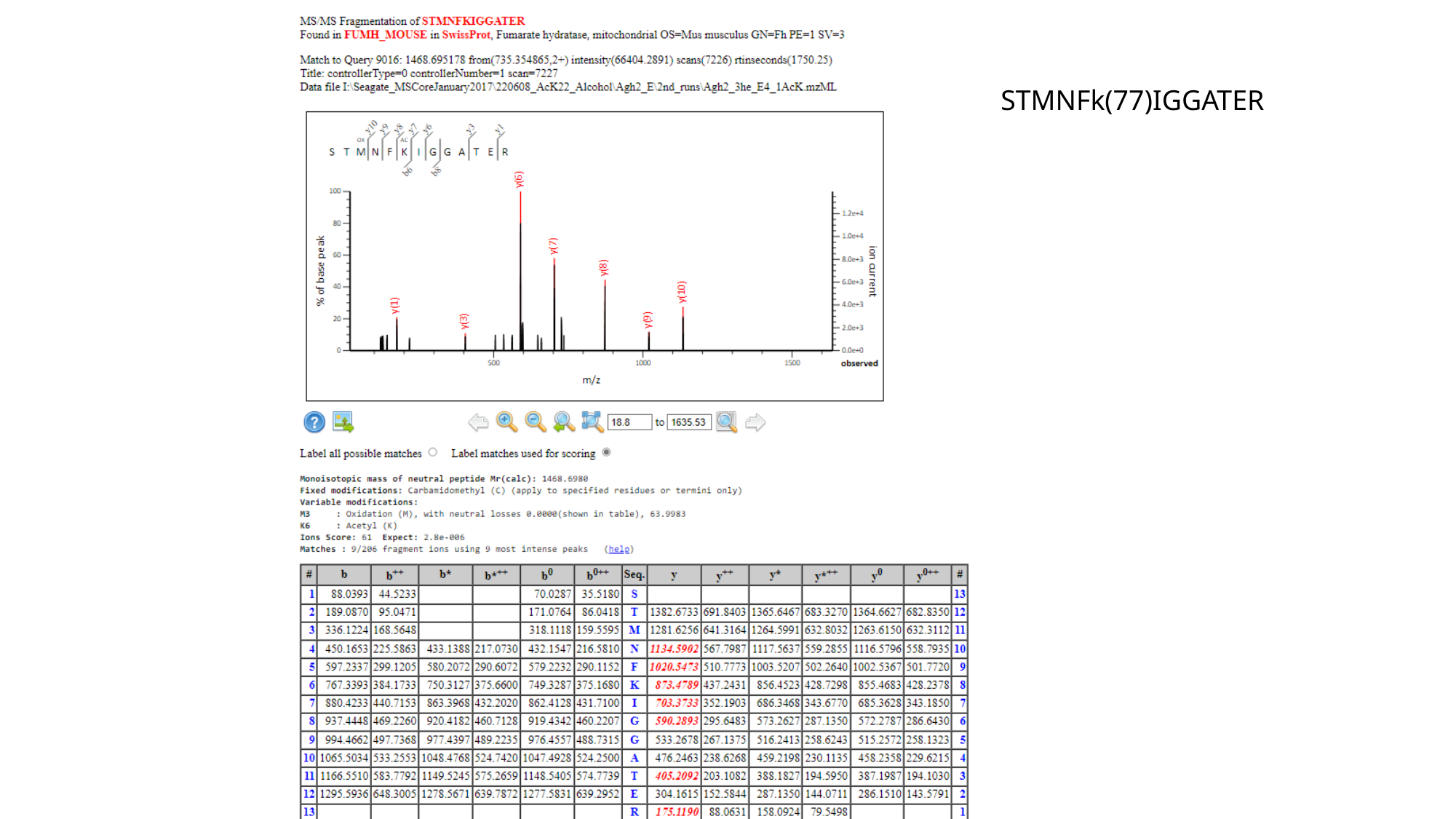

STMNFk(77)IGGATER

## Slide 118
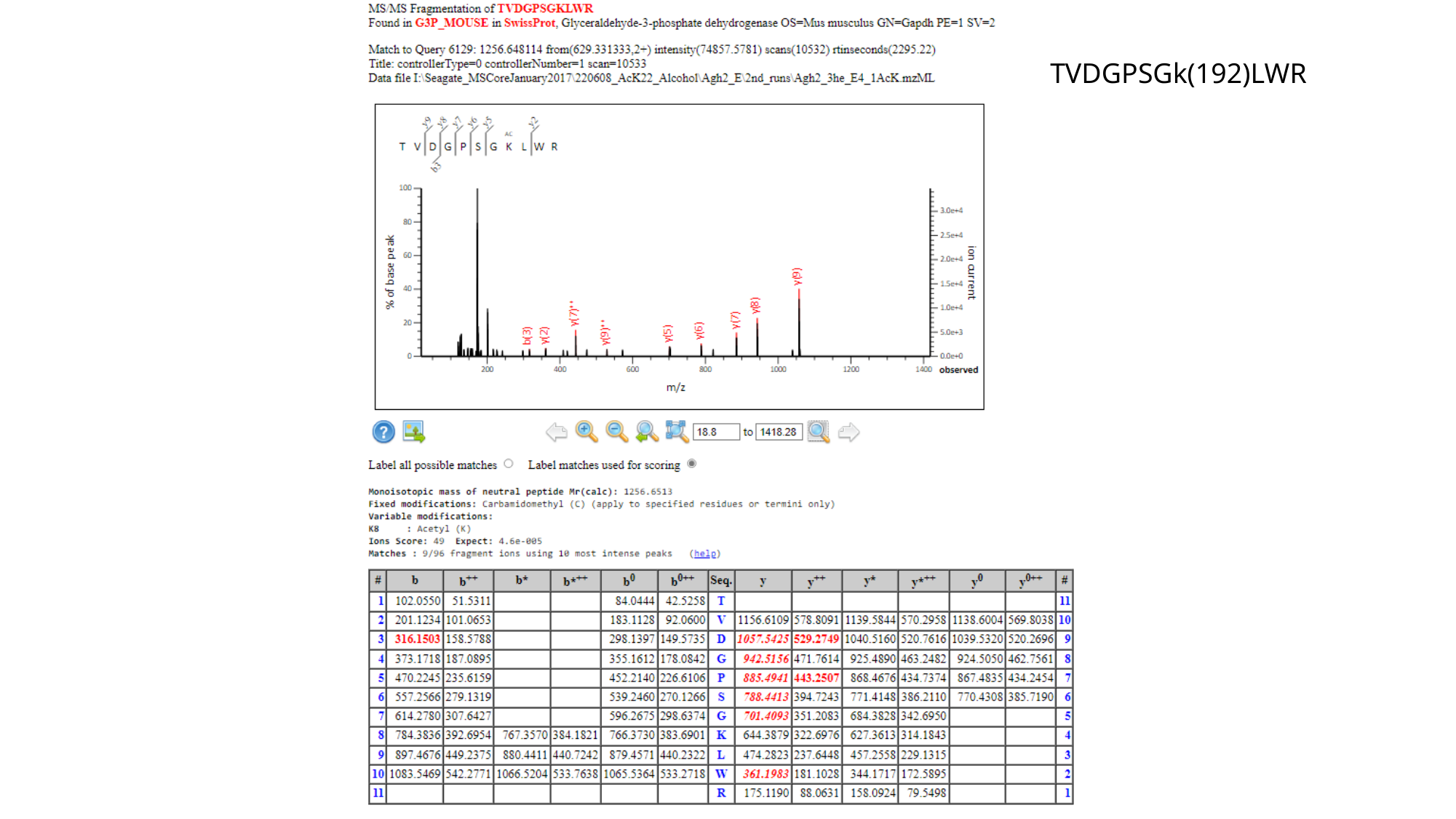

TVDGPSGk(192)LWR

## Slide 119
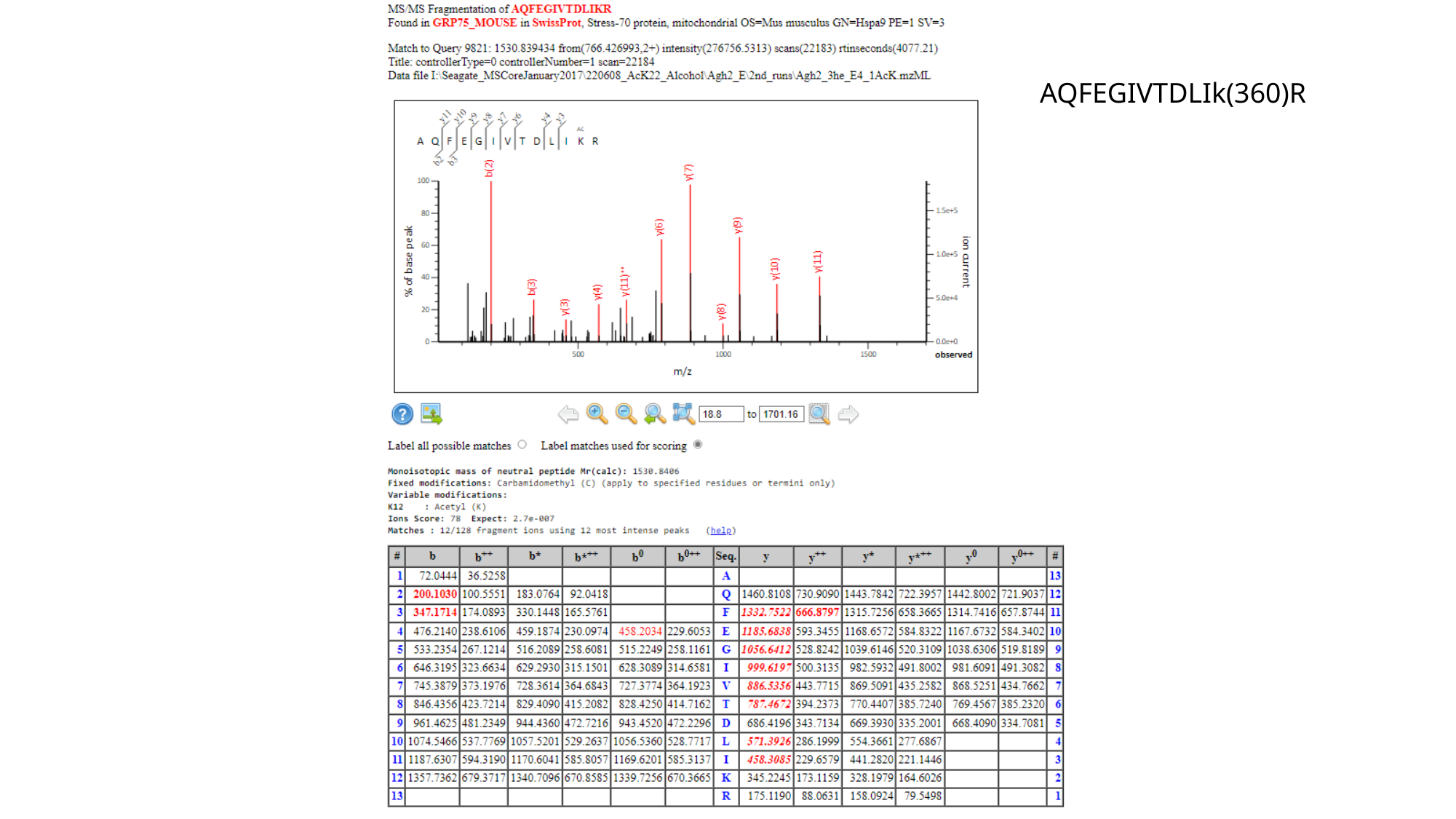

AQFEGIVTDLIk(360)R

## Slide 120
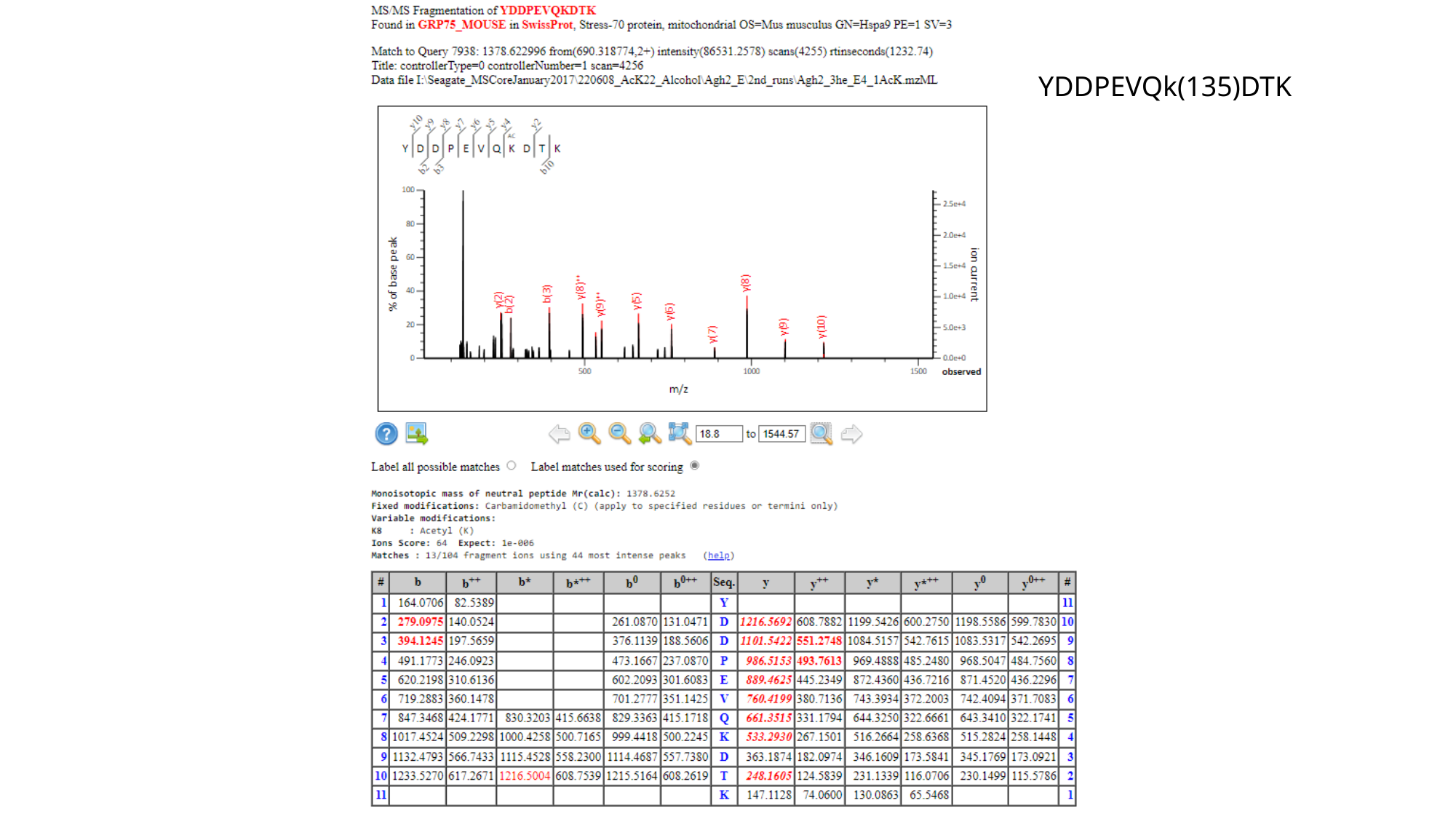

YDDPEVQk(135)DTK

## Slide 121
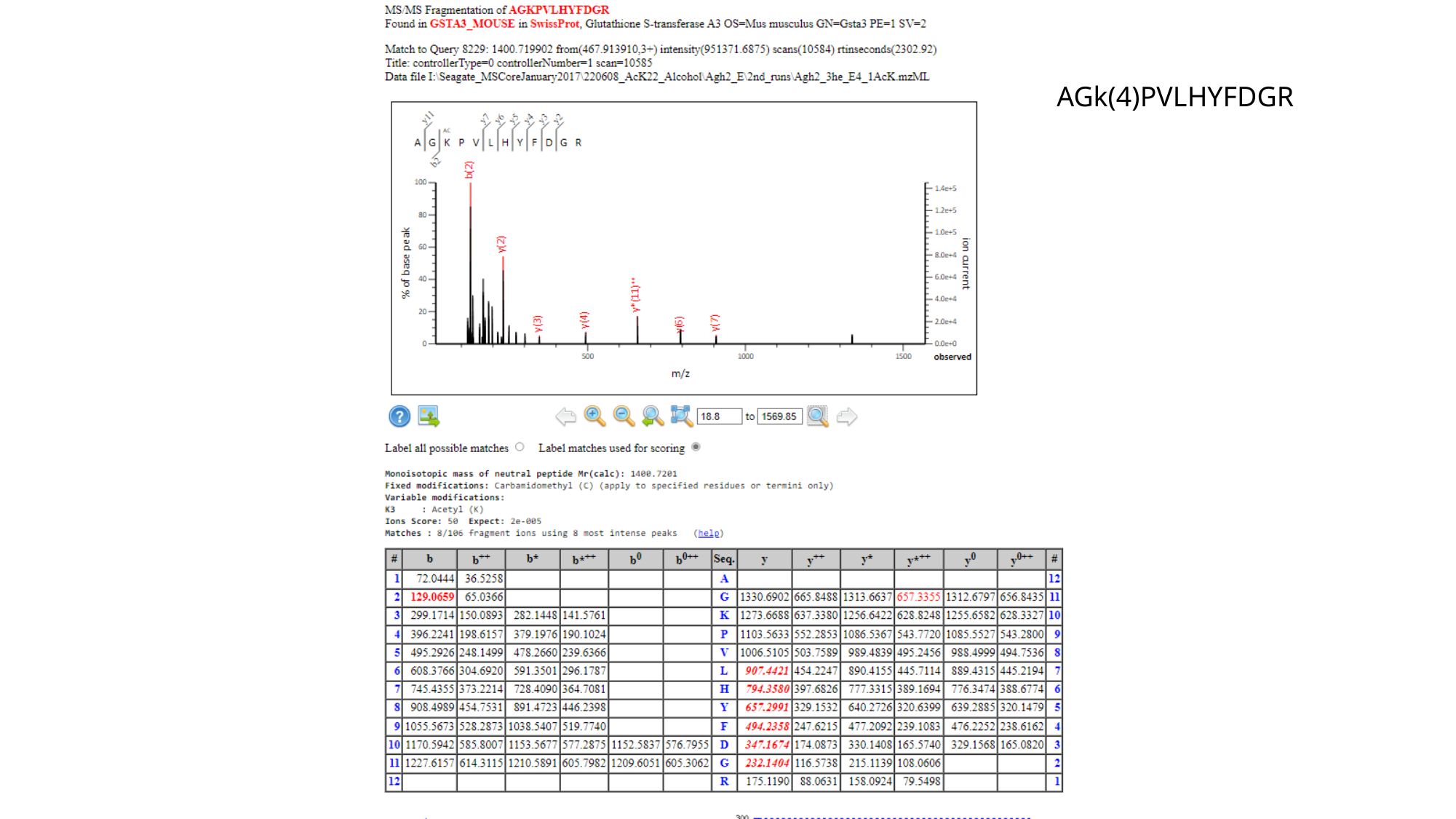

AGk(4)PVLHYFDGR

## Slide 122
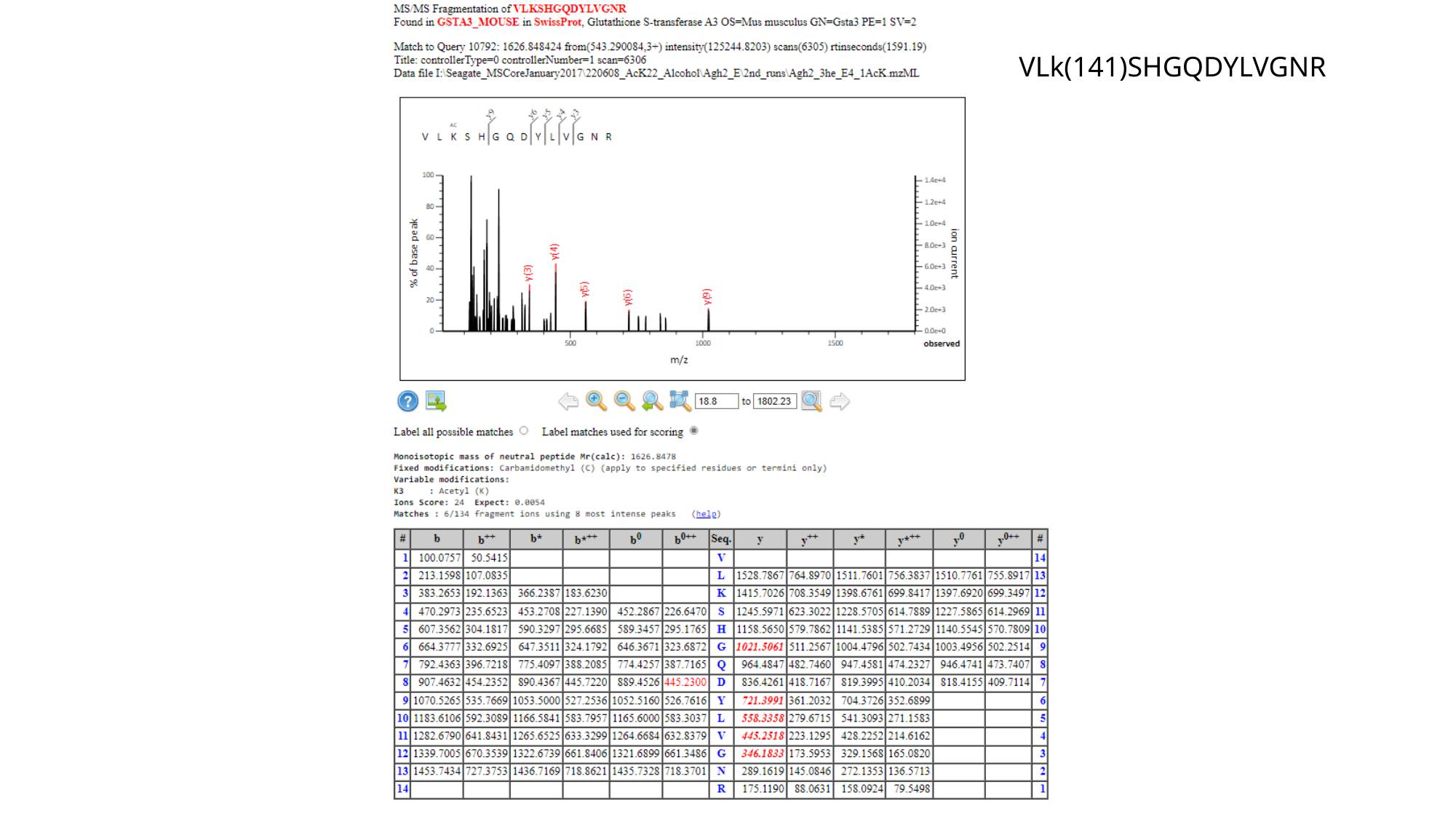

VLk(141)SHGQDYLVGNR

## Slide 123
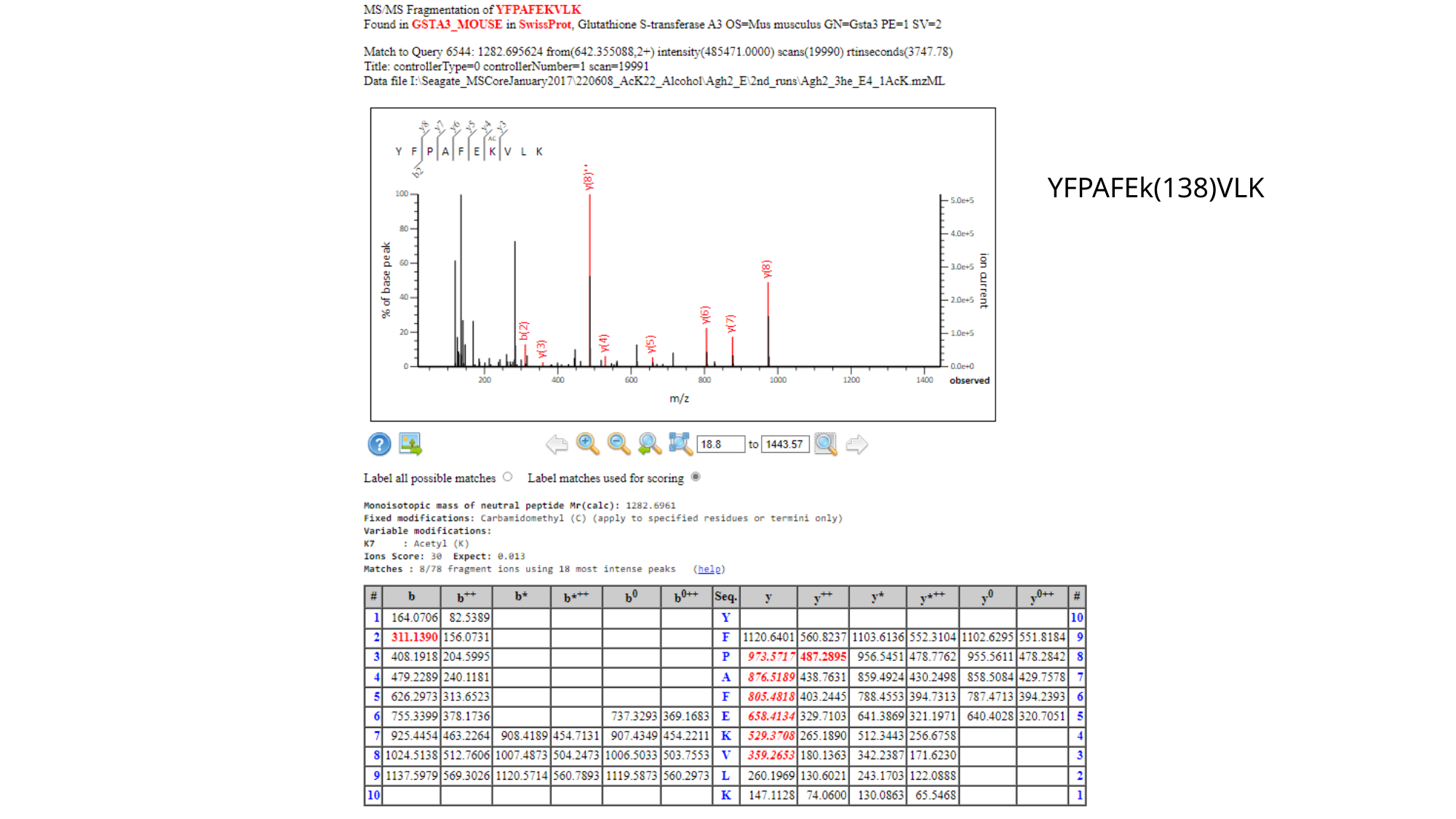

YFPAFEk(138)VLK

## Slide 124
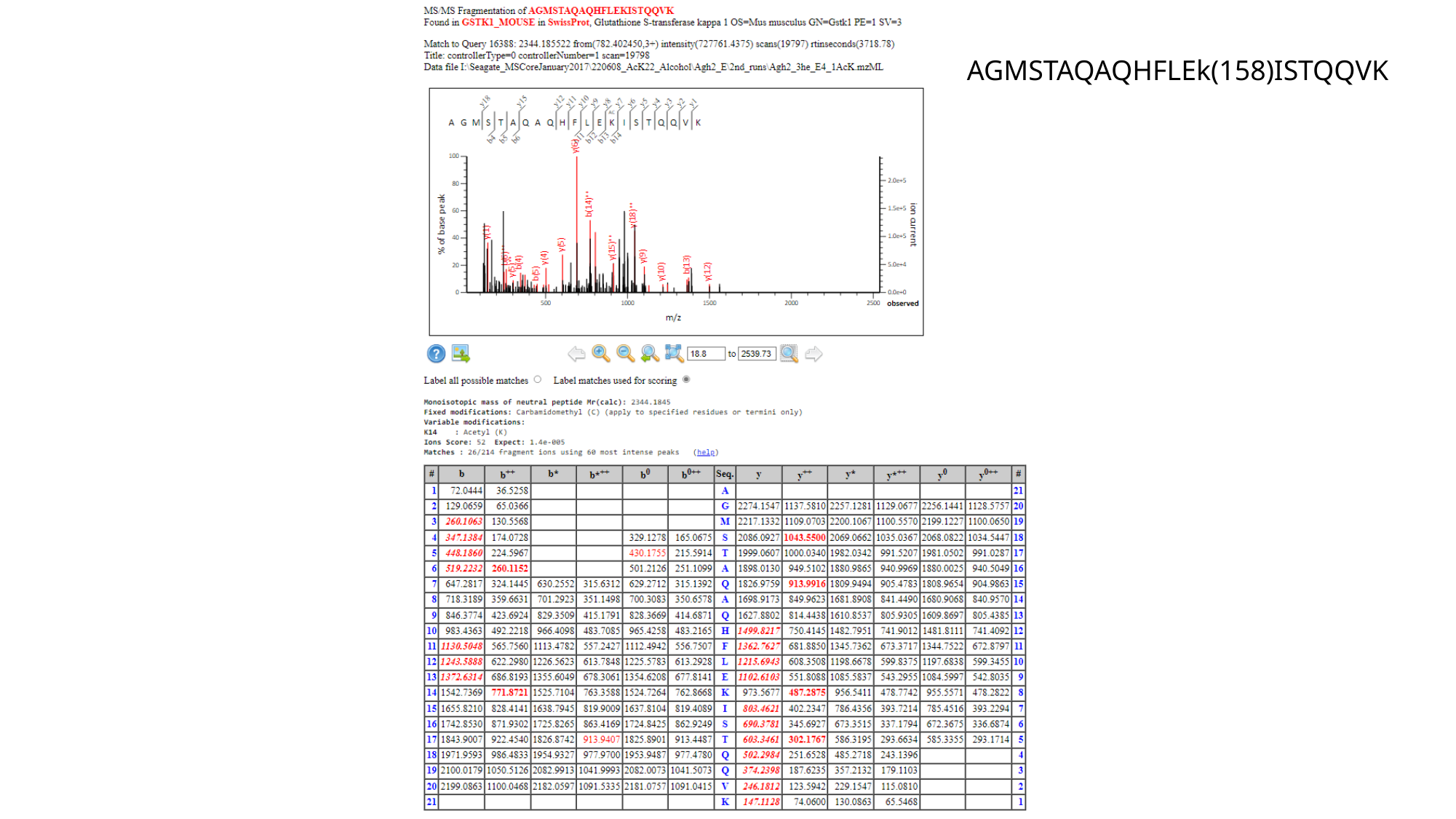

AGMSTAQAQHFLEk(158)ISTQQVK

## Slide 125
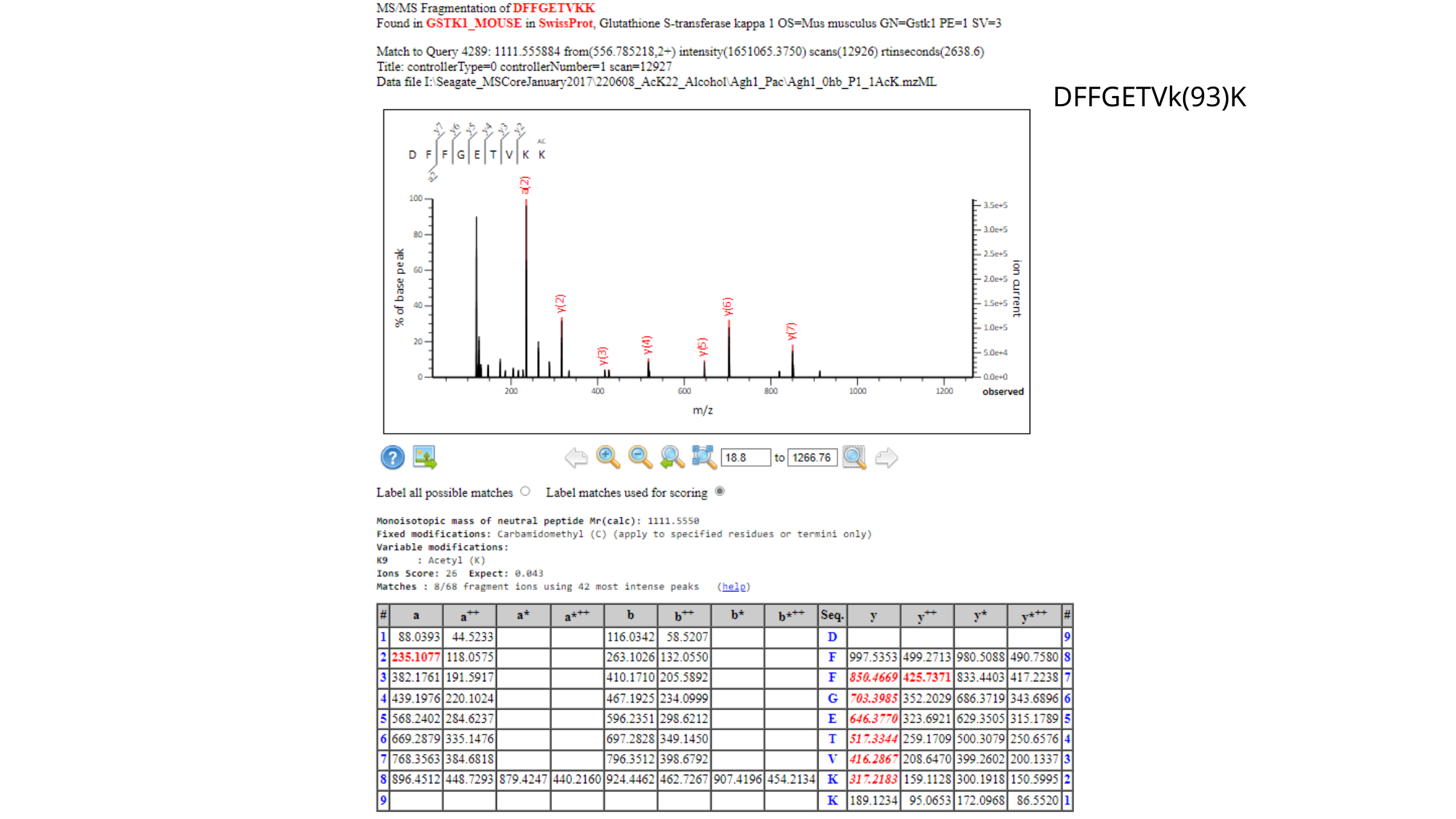

DFFGETVk(93)K

## Slide 126
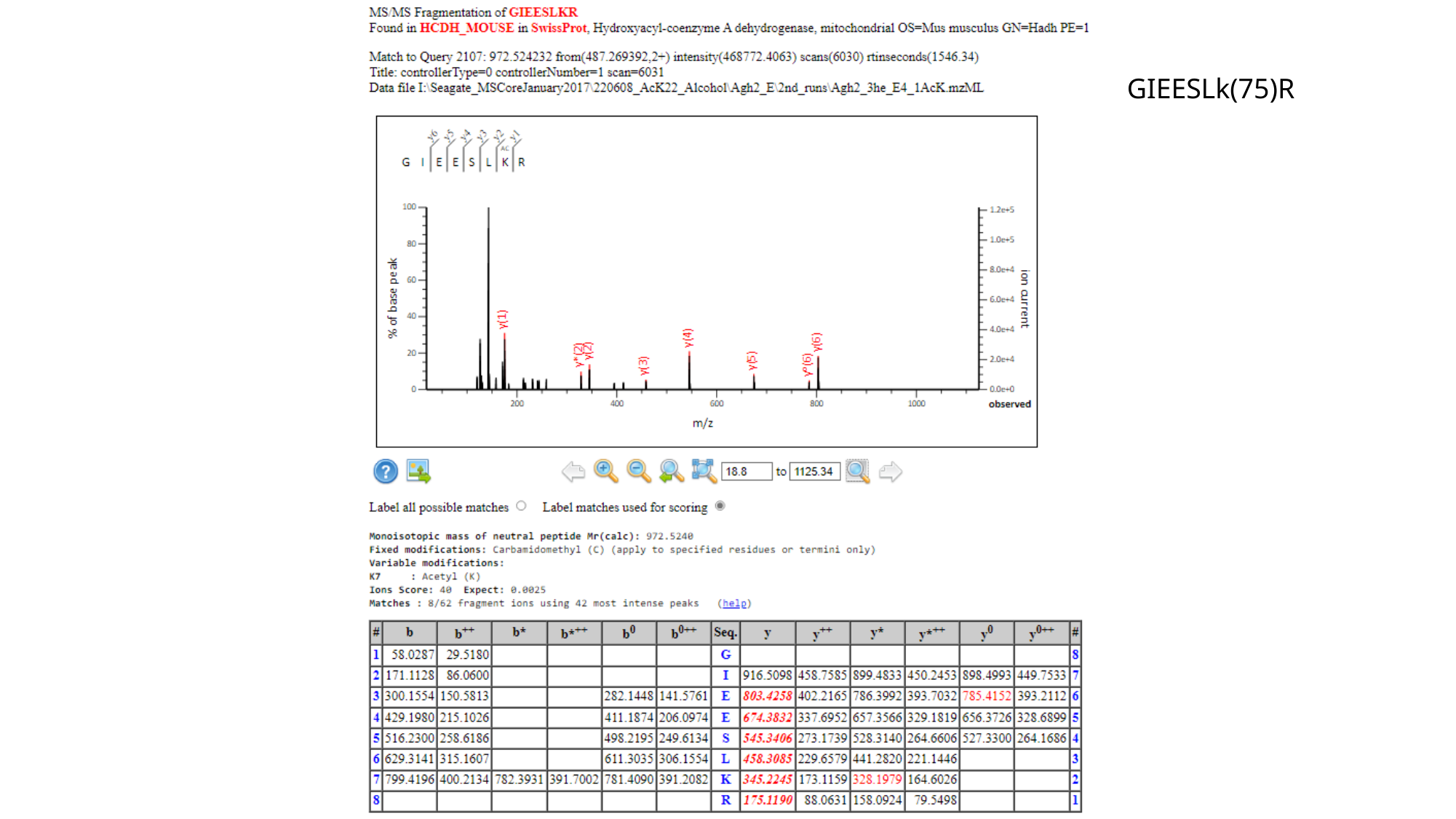

GIEESLk(75)R

## Slide 127
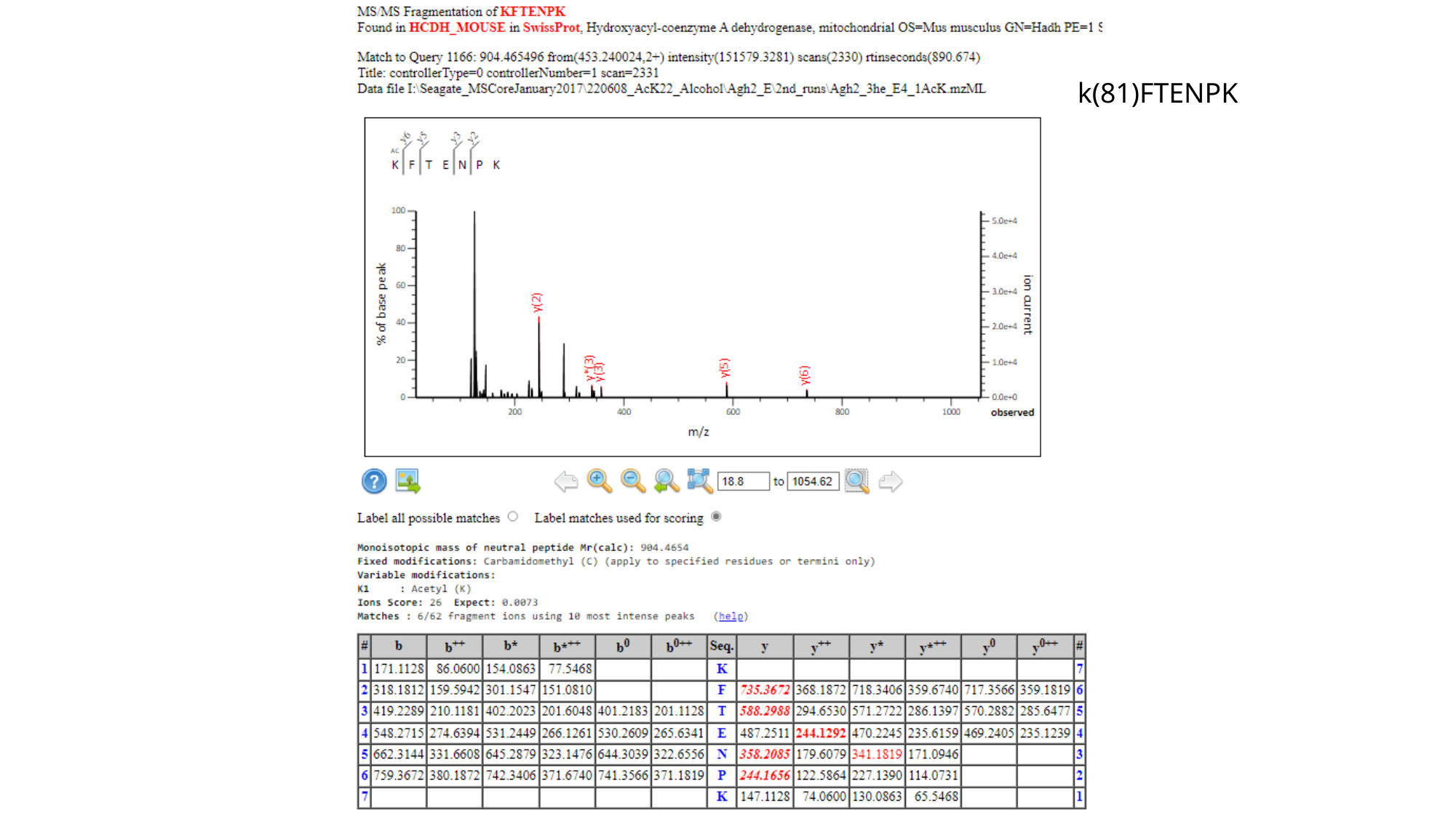

k(81)FTENPK

## Slide 128
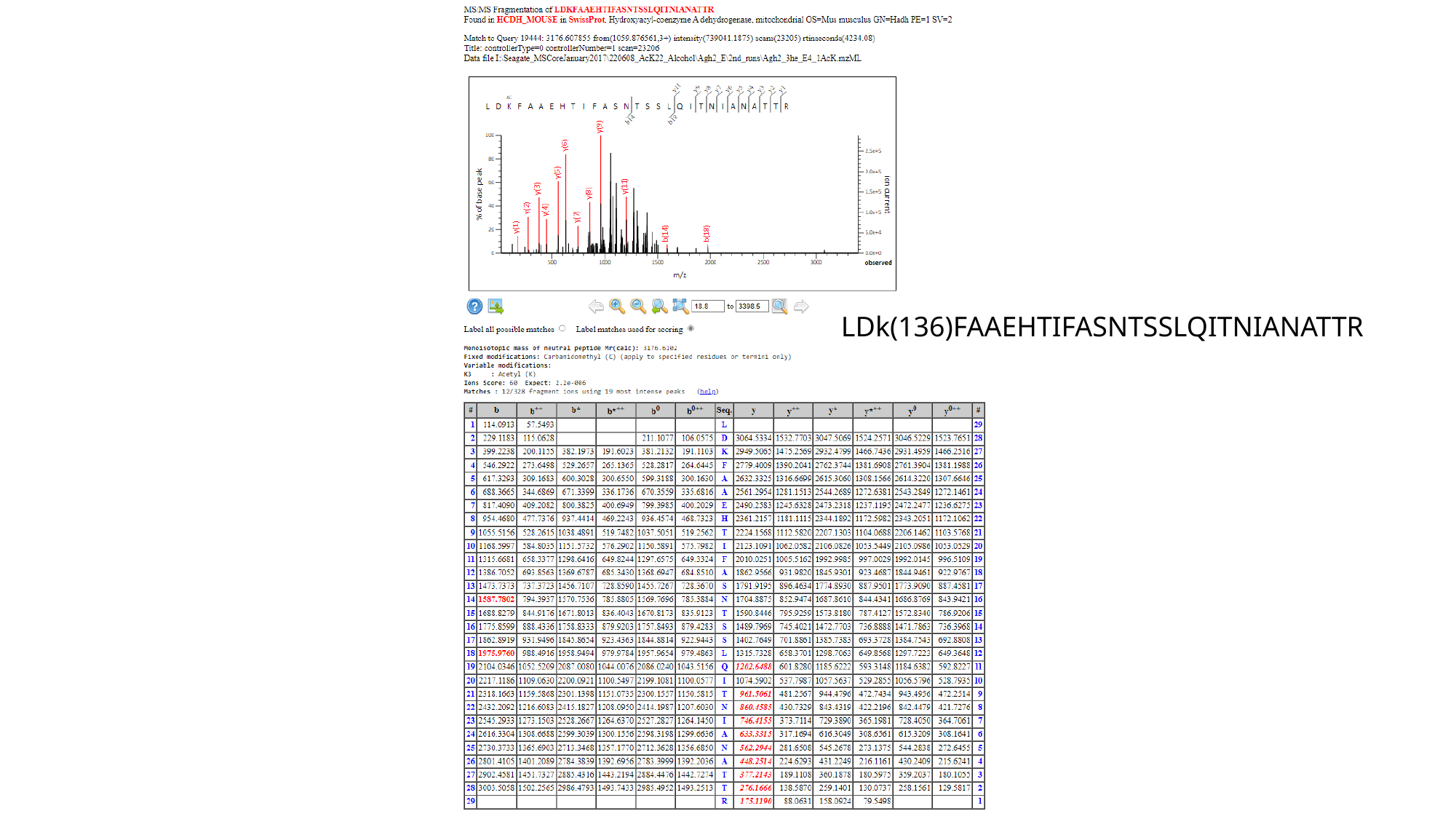

LDk(136)FAAEHTIFASNTSSLQITNIANATTR

## Slide 129
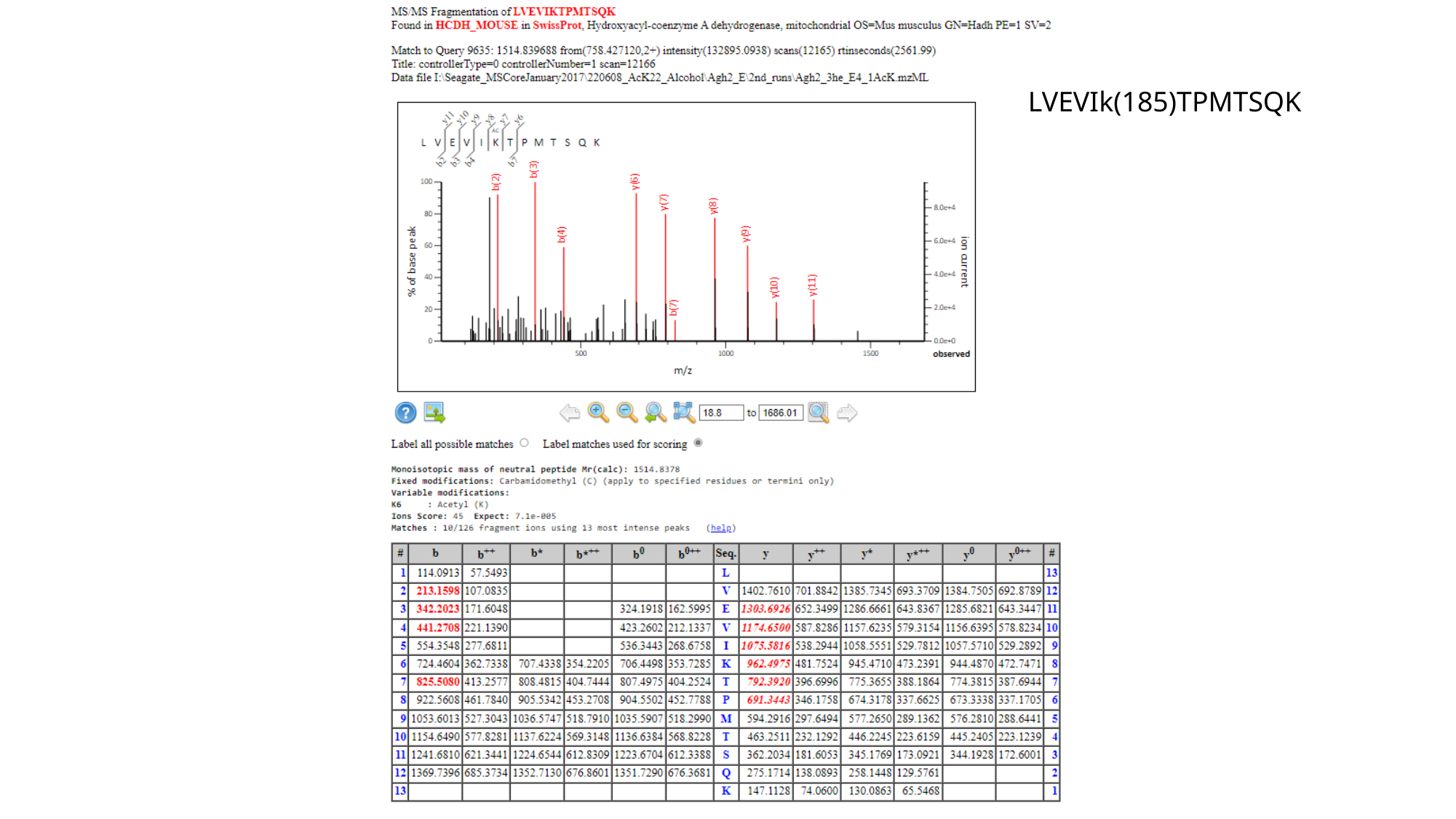

LVEVIk(185)TPMTSQK

## Slide 130
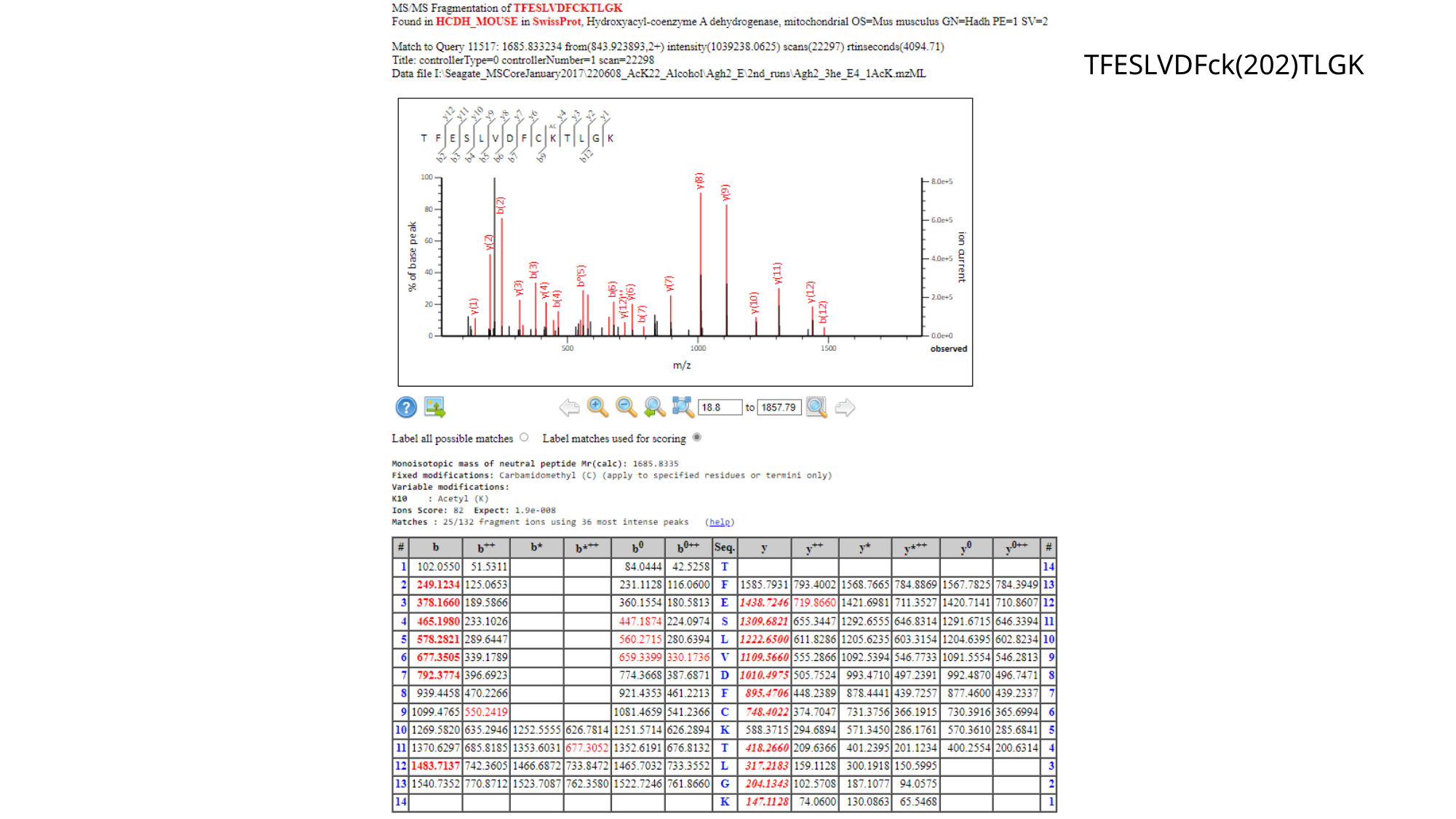

TFESLVDFck(202)TLGK

## Slide 131
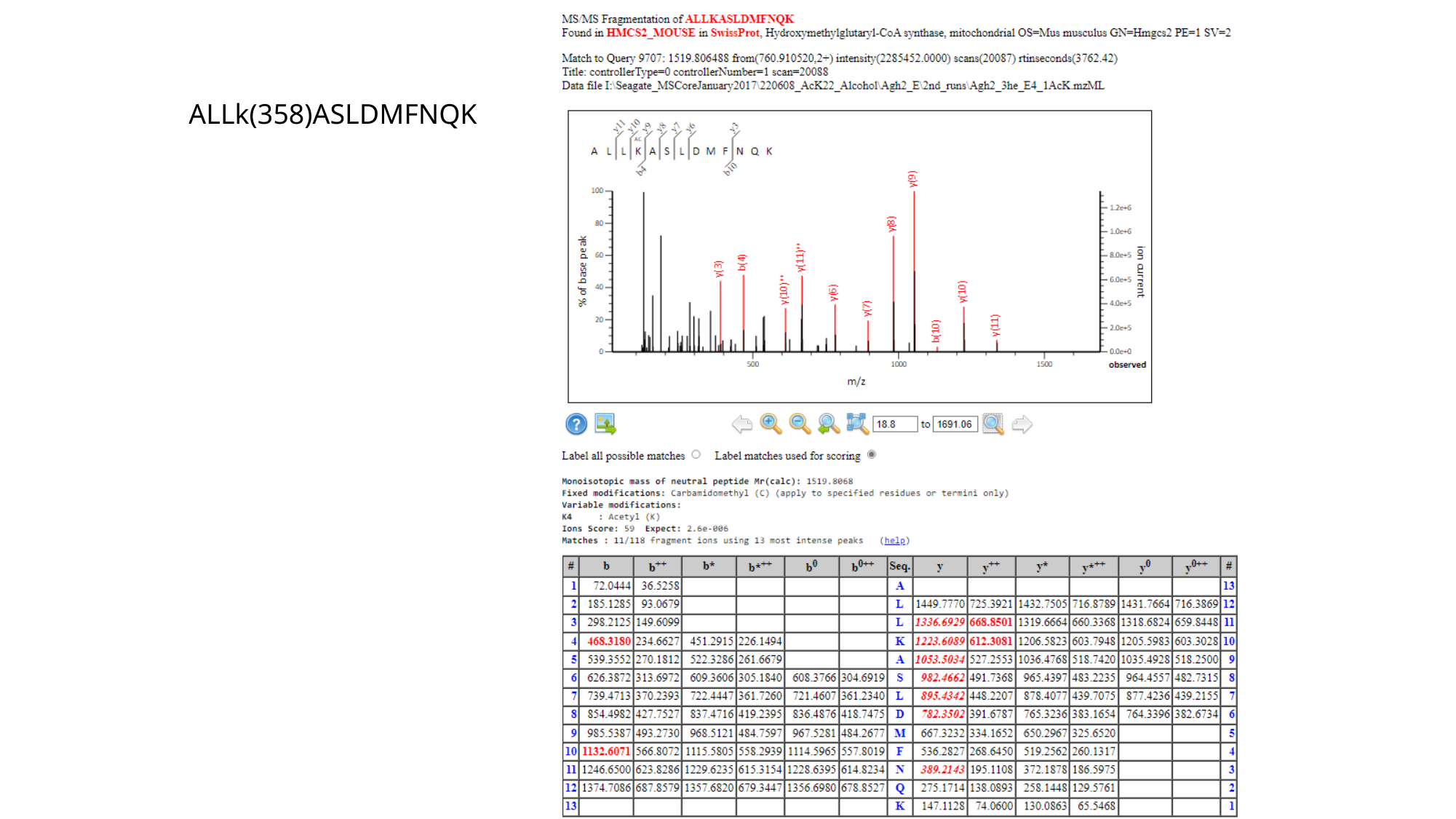

ALLk(358)ASLDMFNQK

## Slide 132
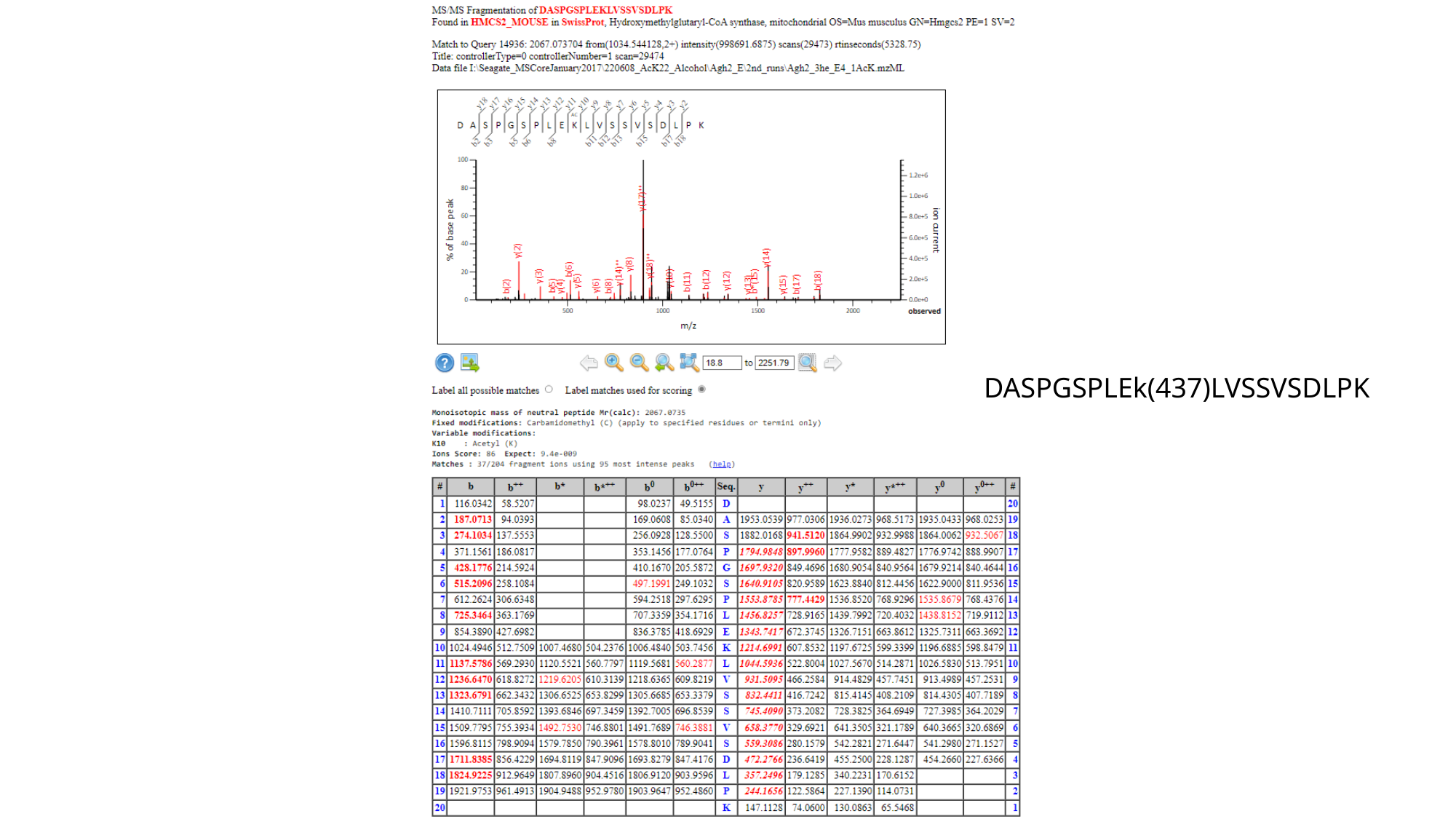

DASPGSPLEk(437)LVSSVSDLPK

## Slide 133
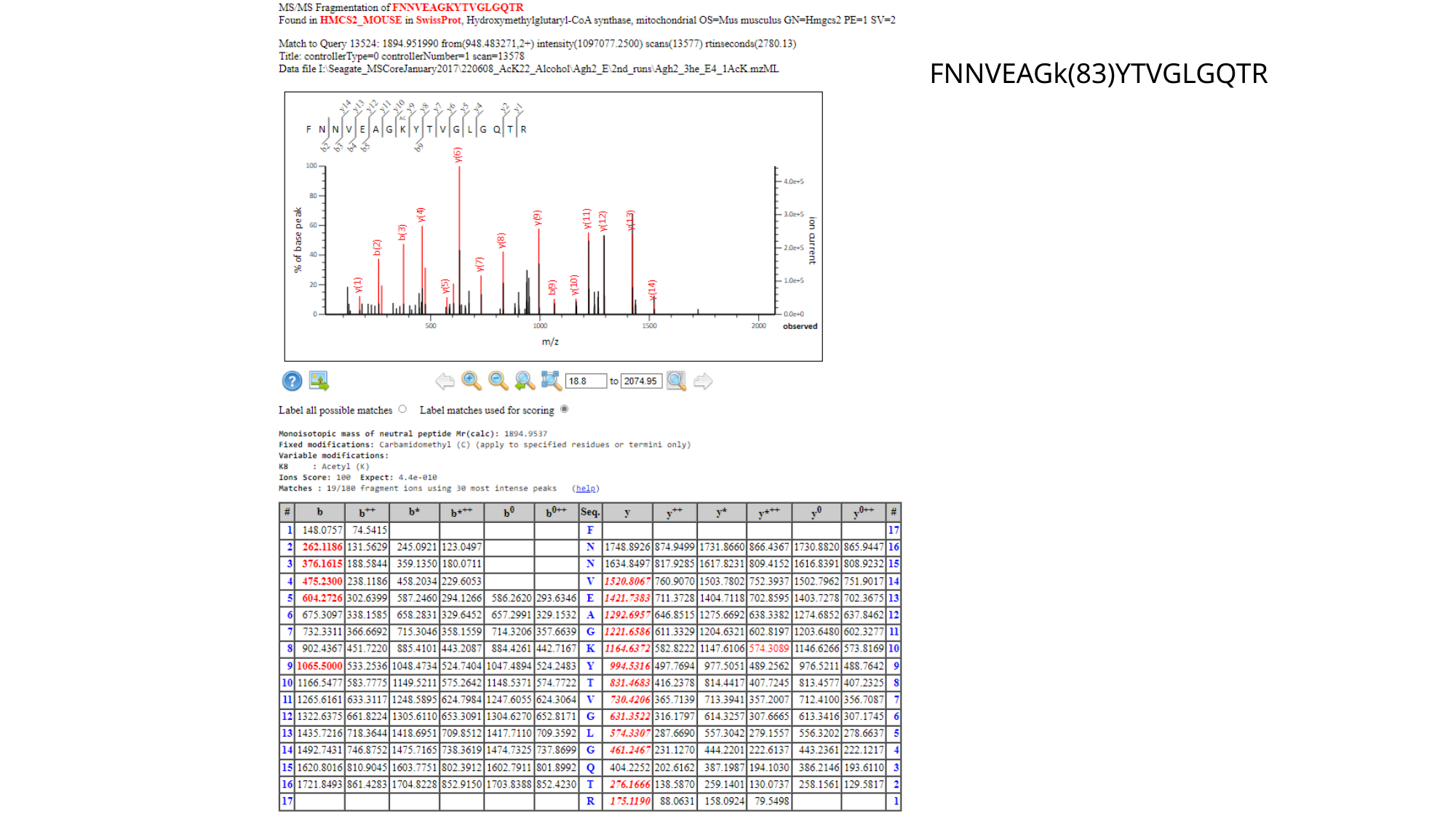

FNNVEAGk(83)YTVGLGQTR

## Slide 134
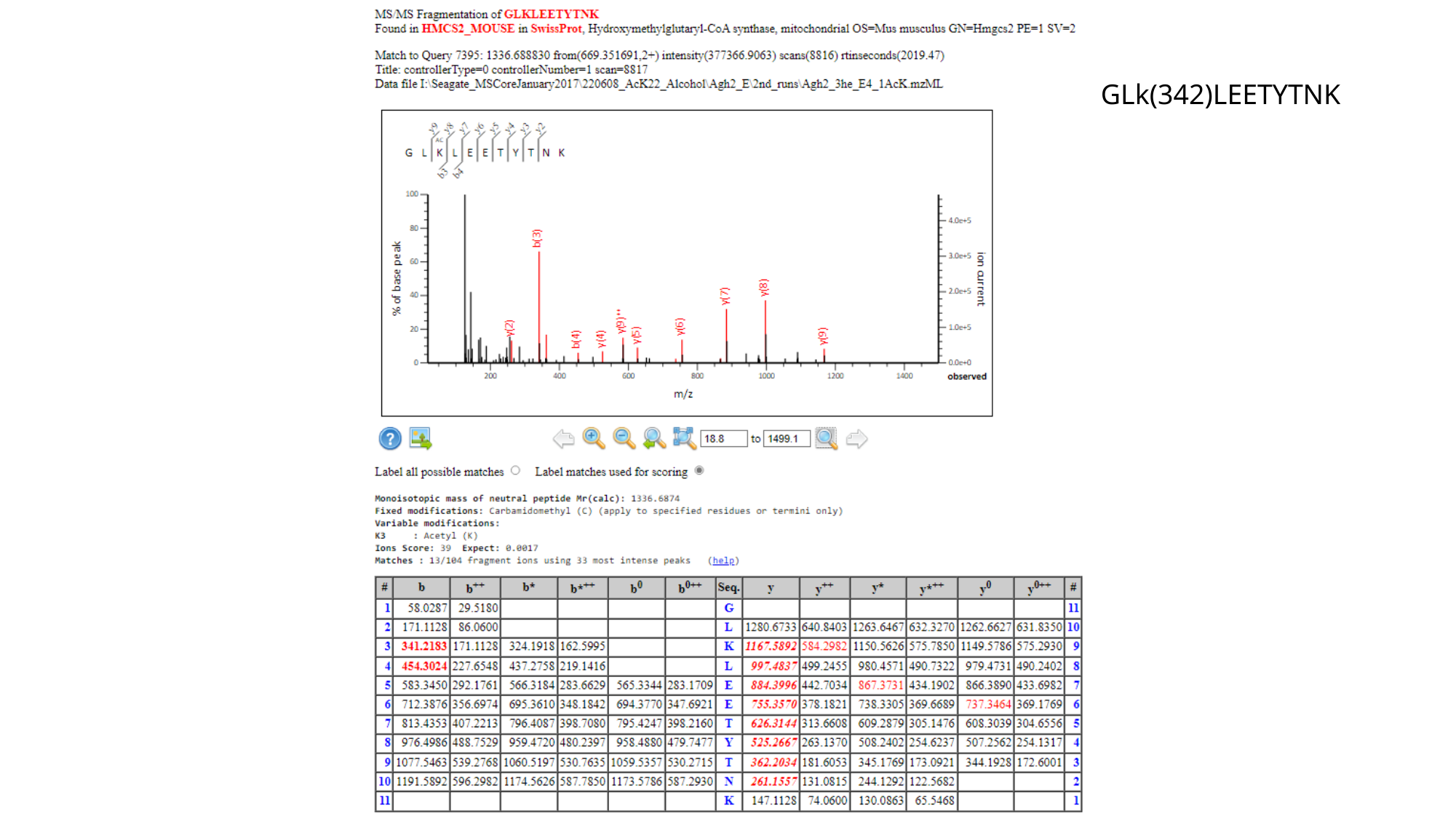

GLk(342)LEETYTNK

## Slide 135
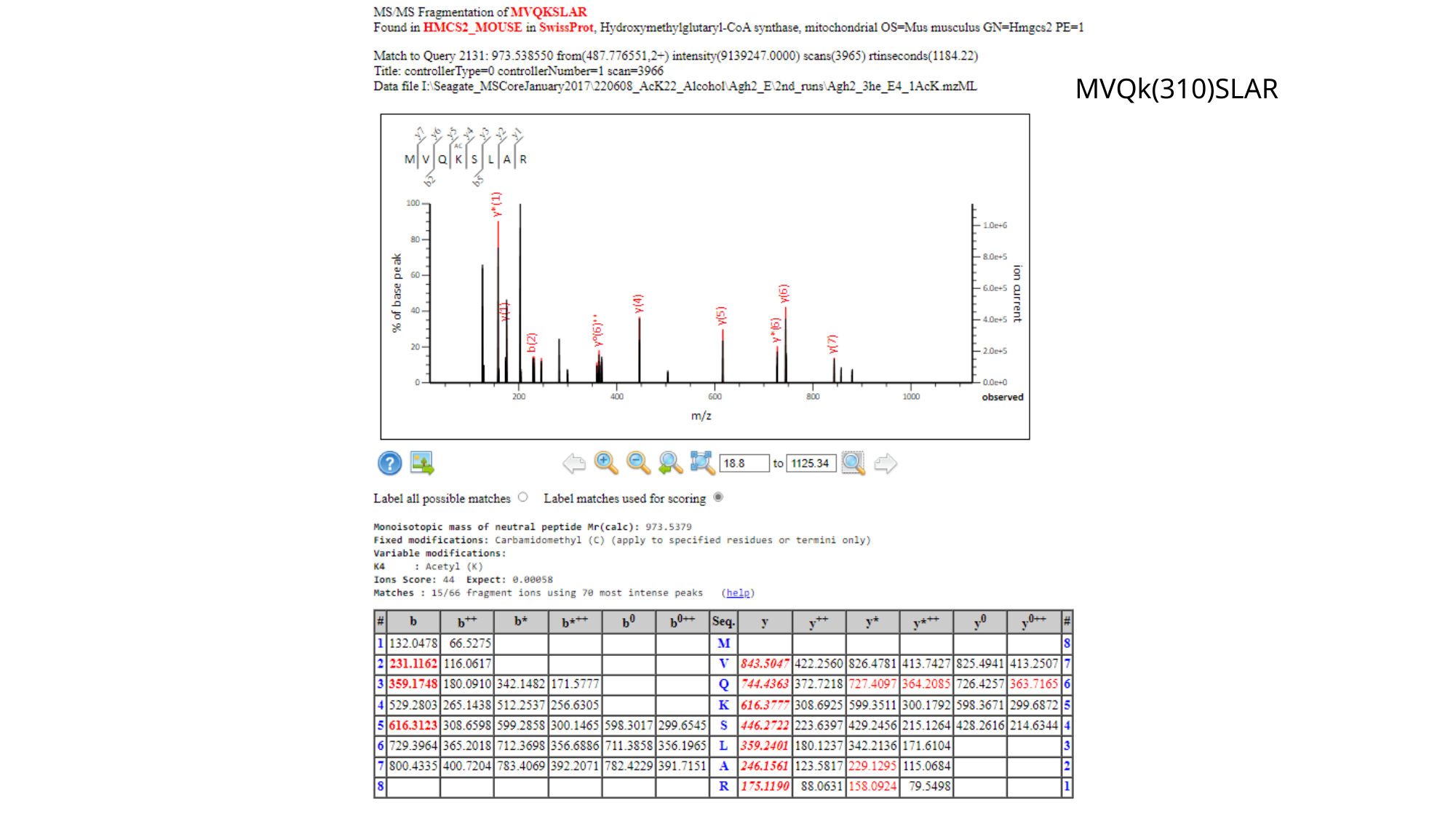

MVQk(310)SLAR

## Slide 136
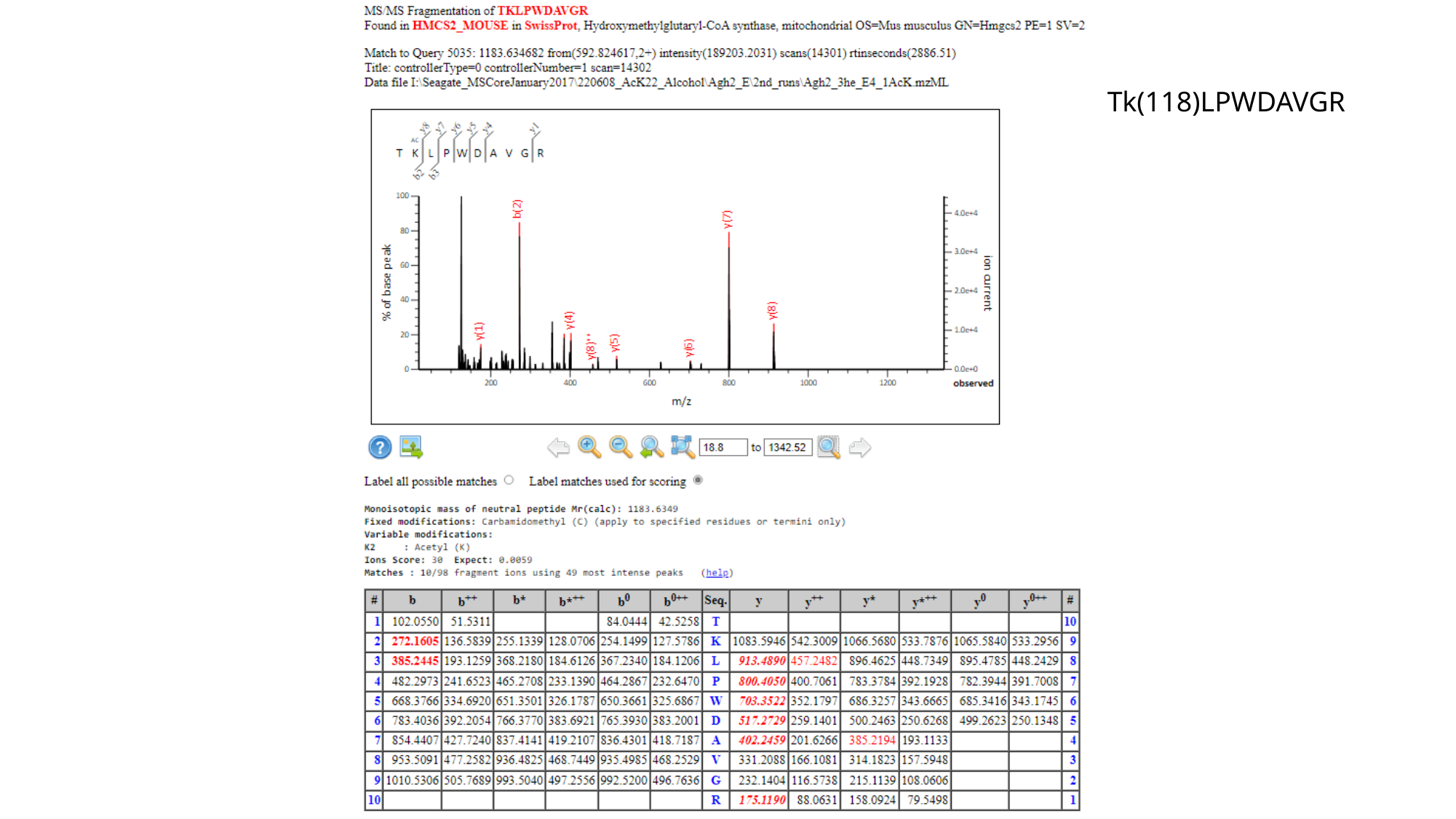

Tk(118)LPWDAVGR

## Slide 137
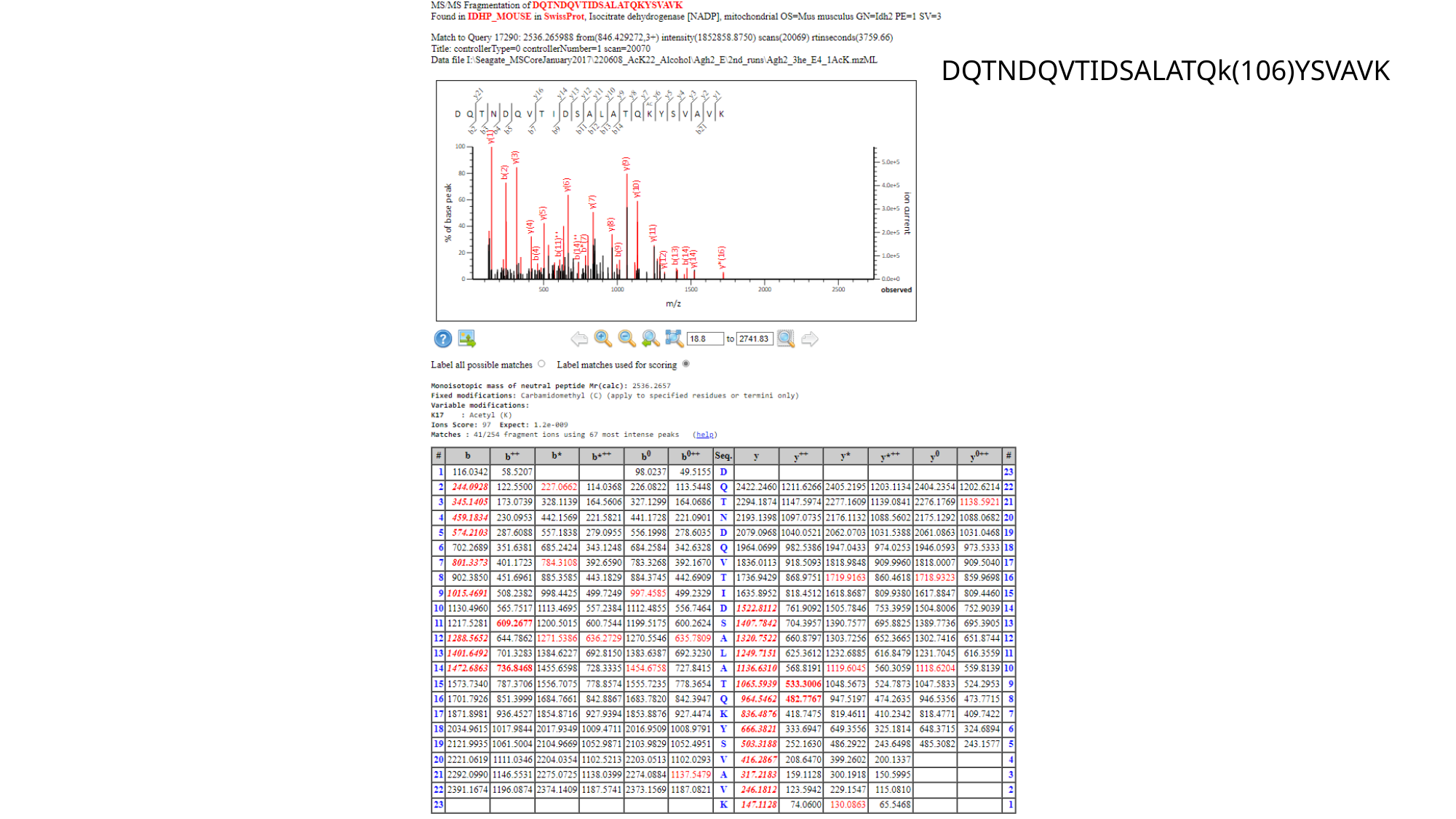

DQTNDQVTIDSALATQk(106)YSVAVK

## Slide 138
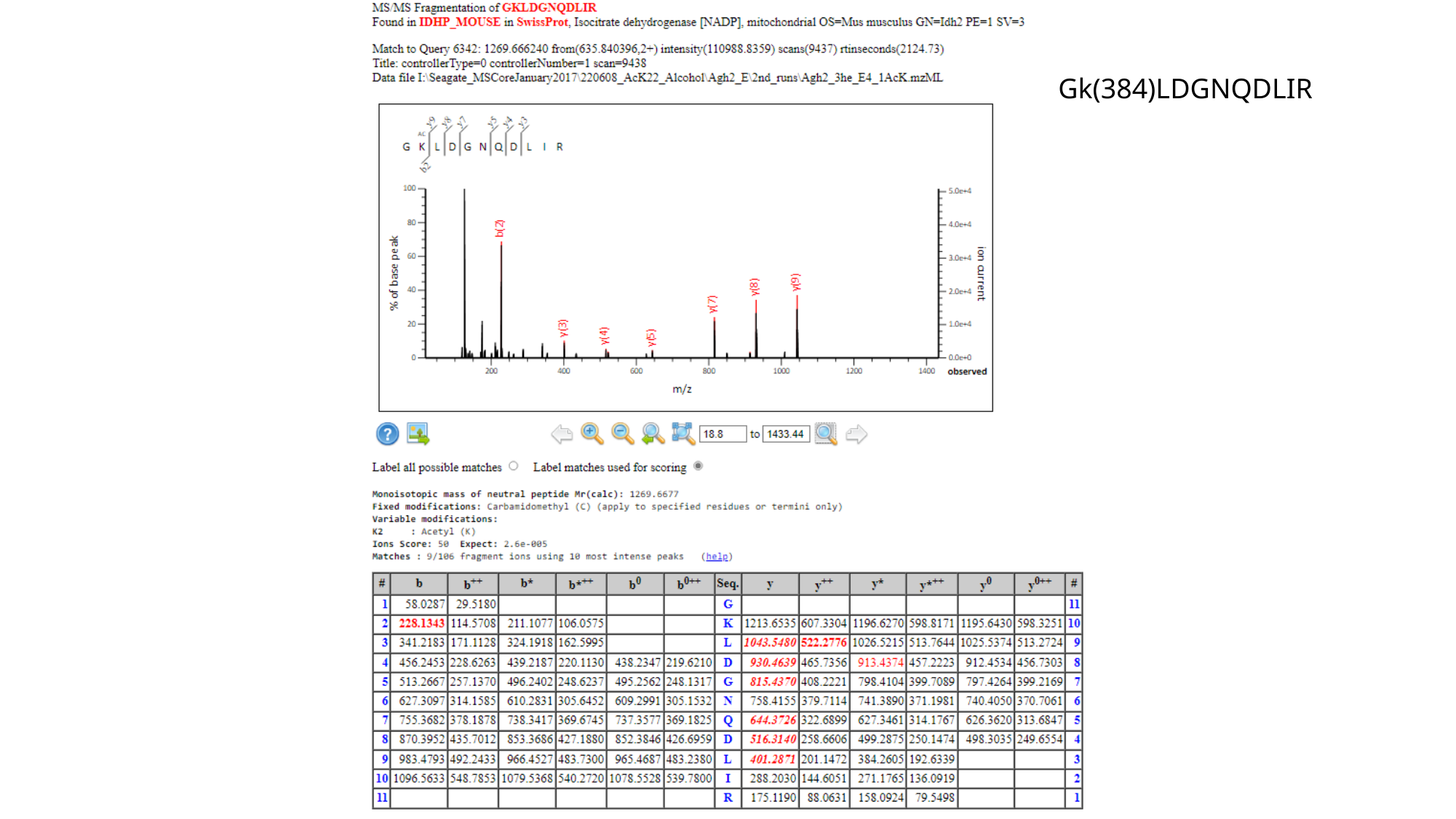

Gk(384)LDGNQDLIR

## Slide 139
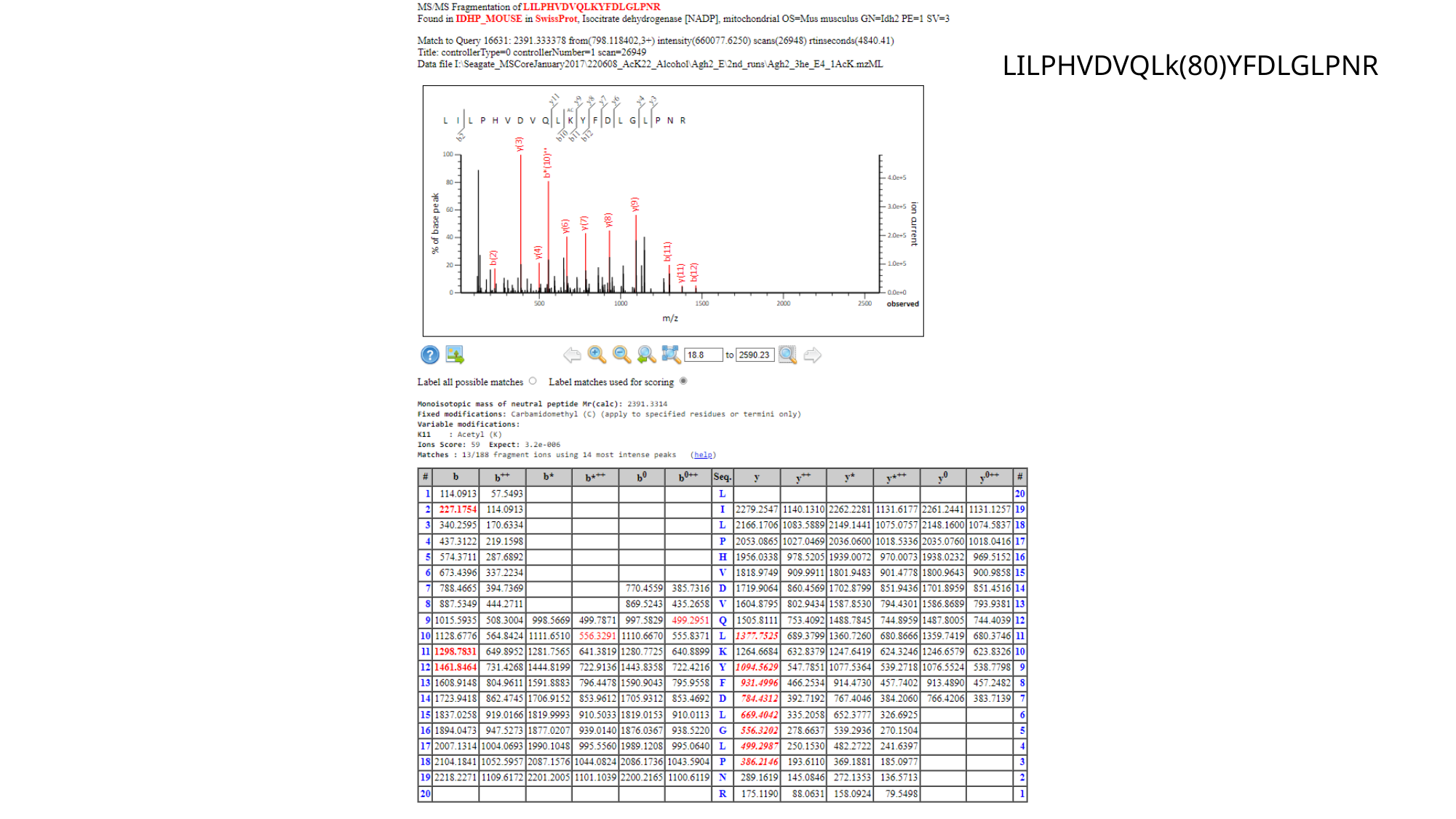

LILPHVDVQLk(80)YFDLGLPNR

## Slide 140
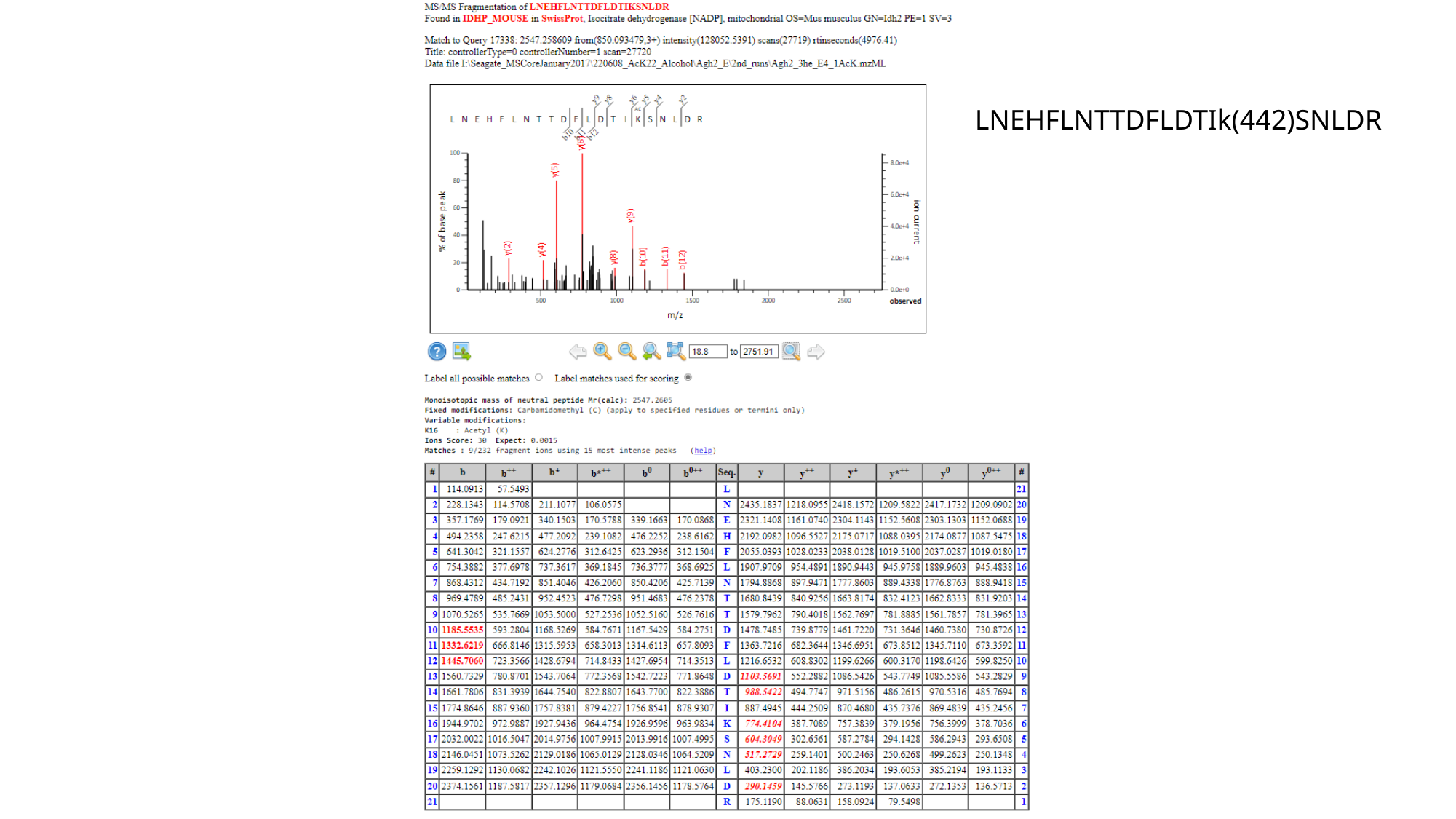

LNEHFLNTTDFLDTIk(442)SNLDR

## Slide 141
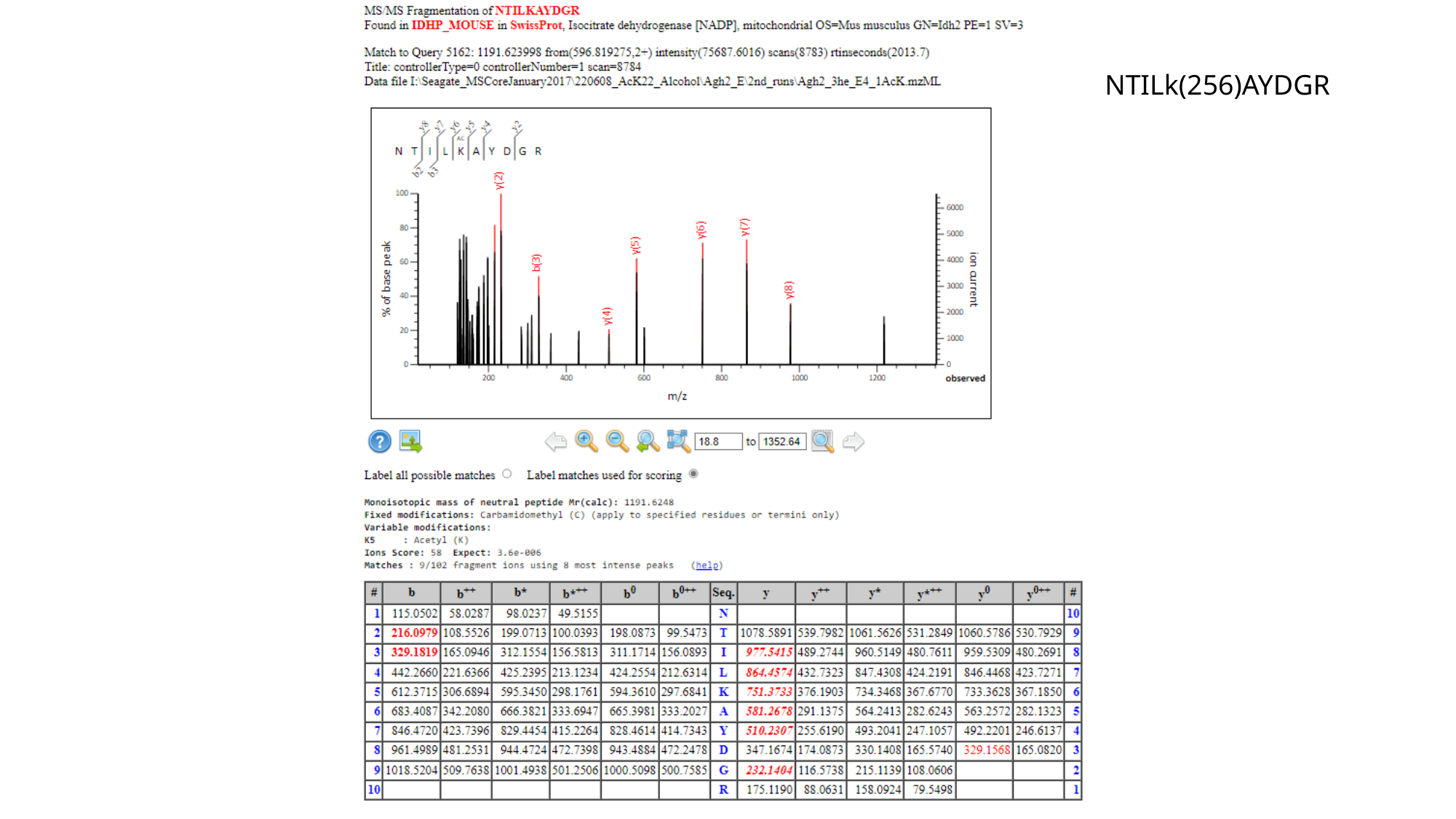

NTILk(256)AYDGR

## Slide 142
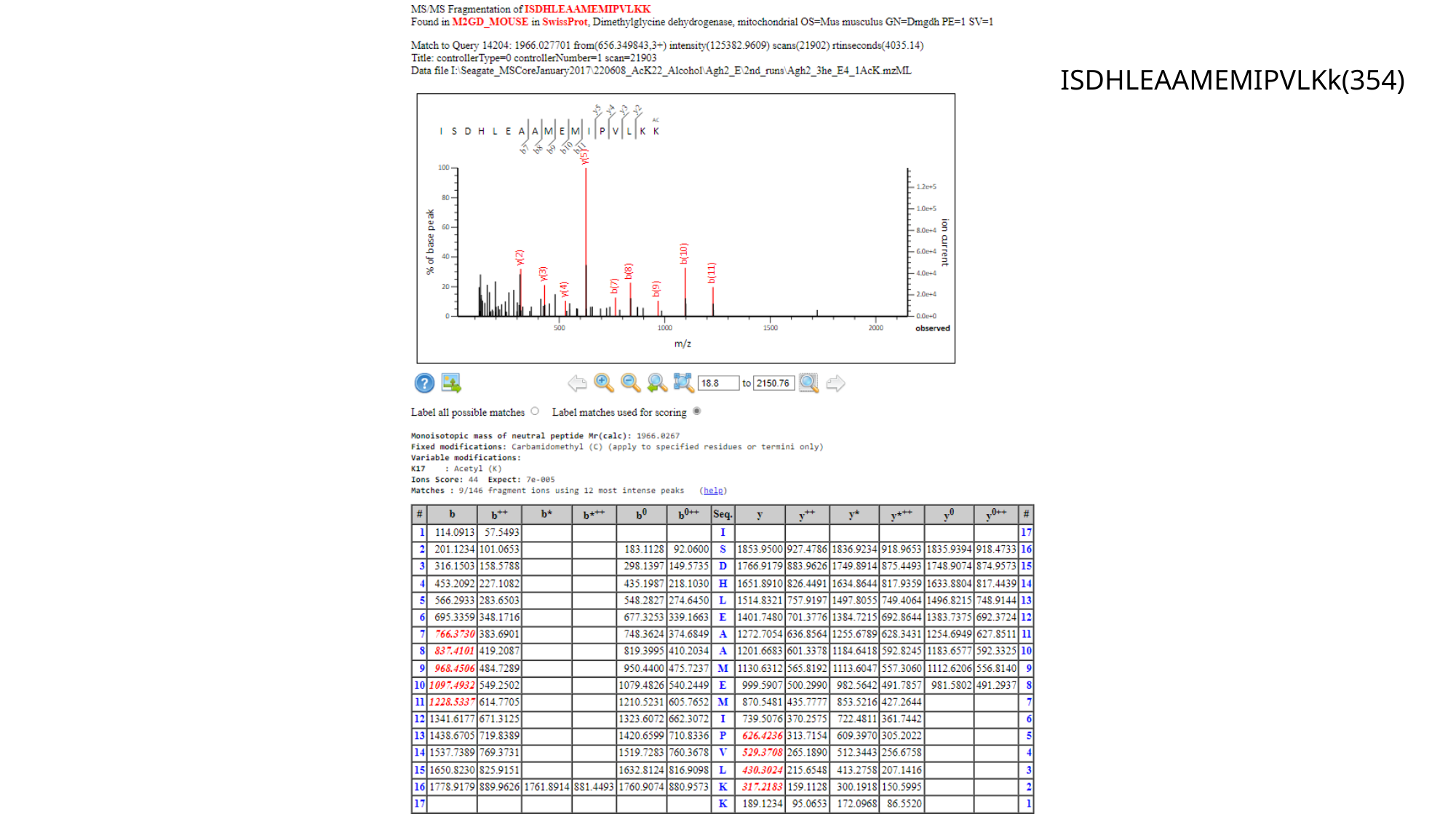

ISDHLEAAMEMIPVLKk(354)

## Slide 143
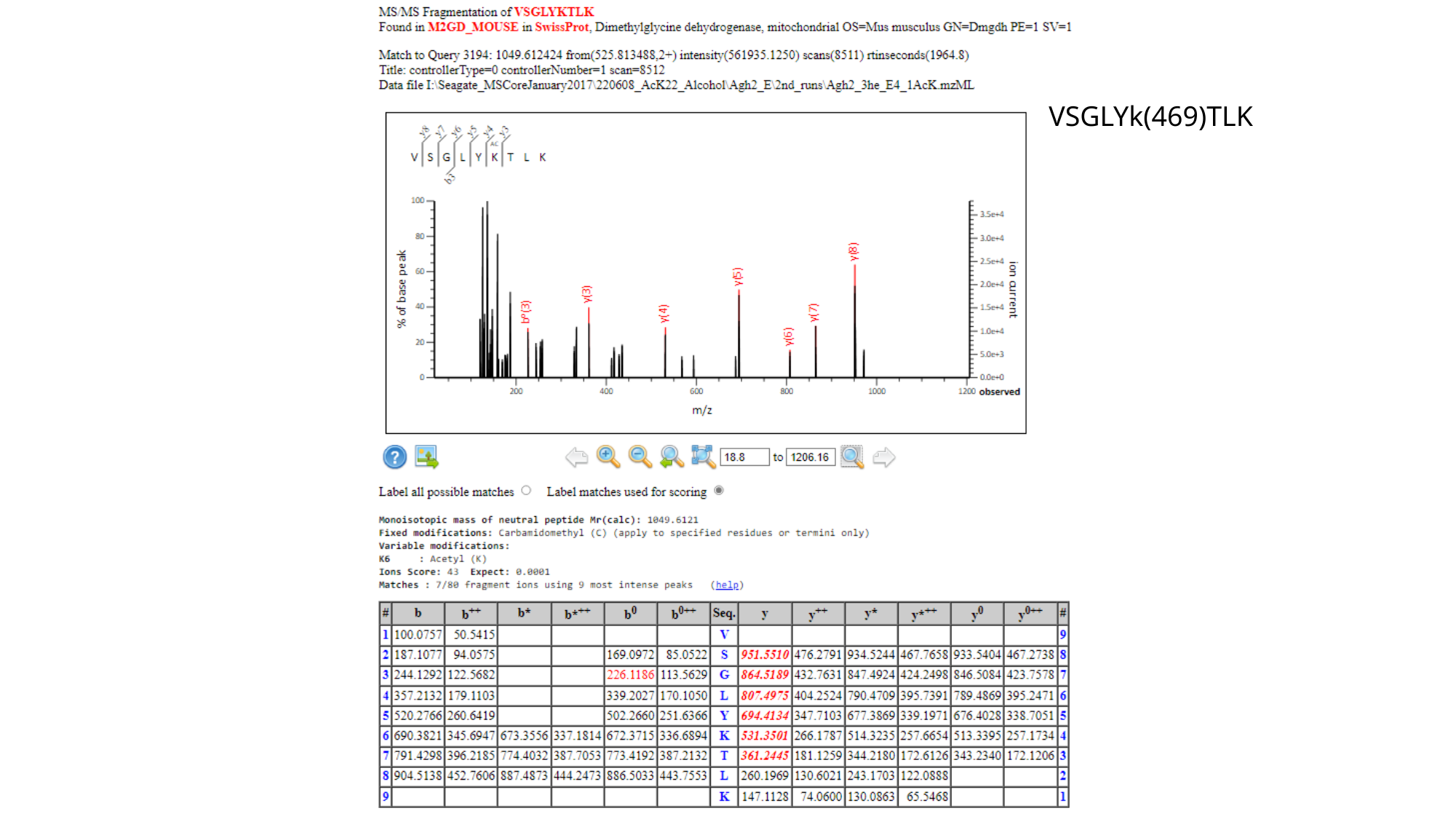

VSGLYk(469)TLK

## Slide 144
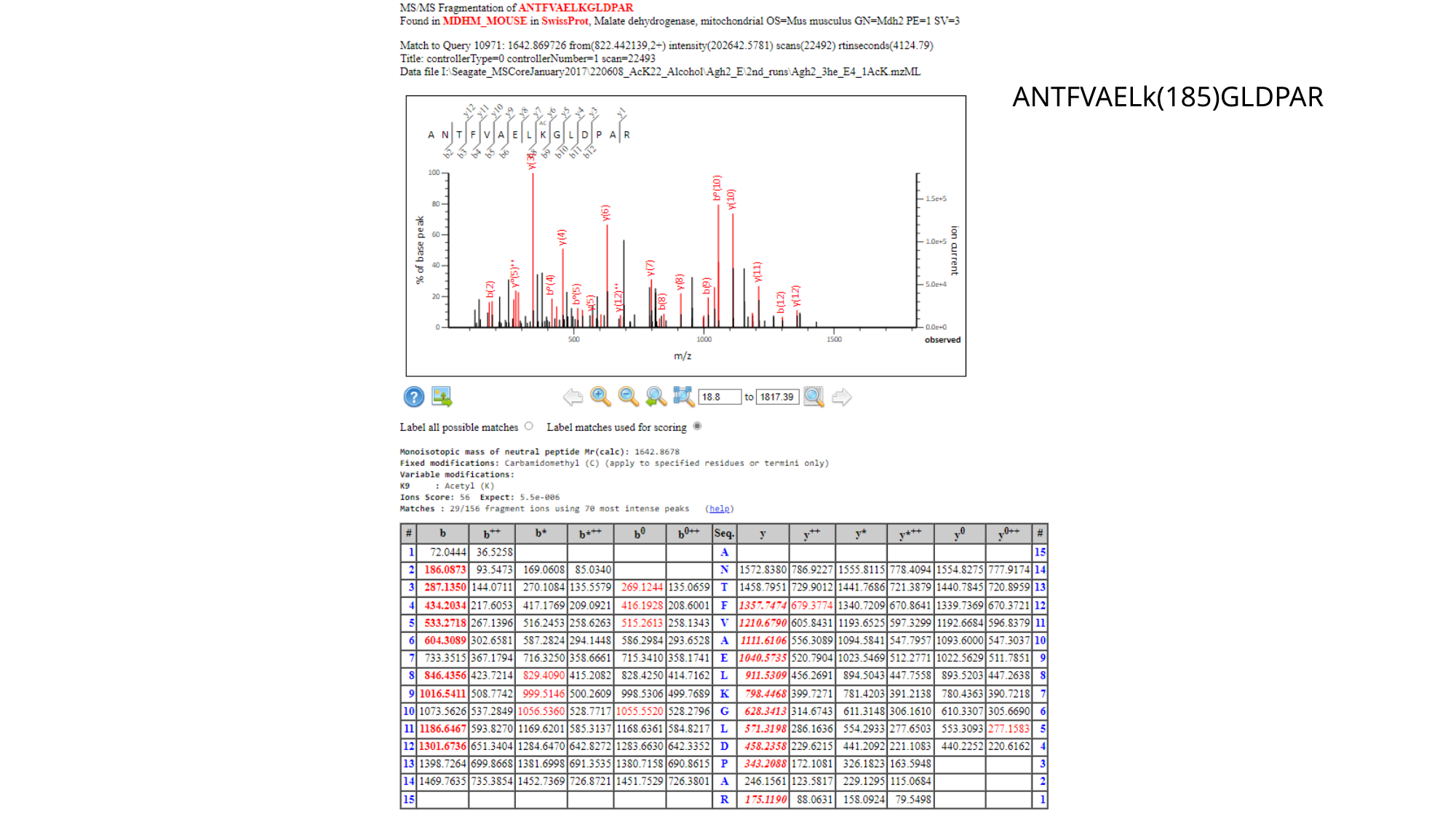

ANTFVAELk(185)GLDPAR

## Slide 145
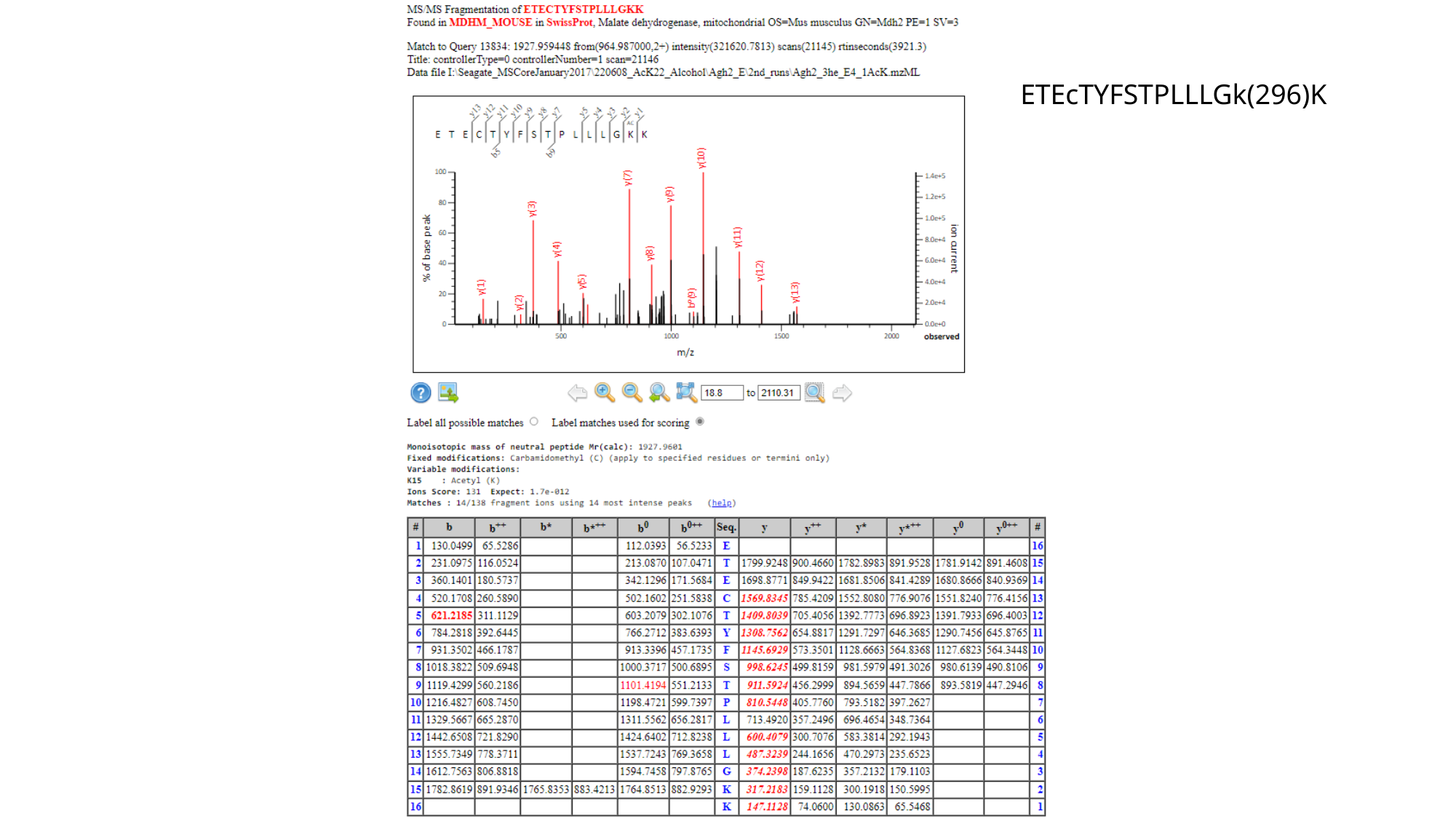

ETEcTYFSTPLLLGk(296)K

## Slide 146
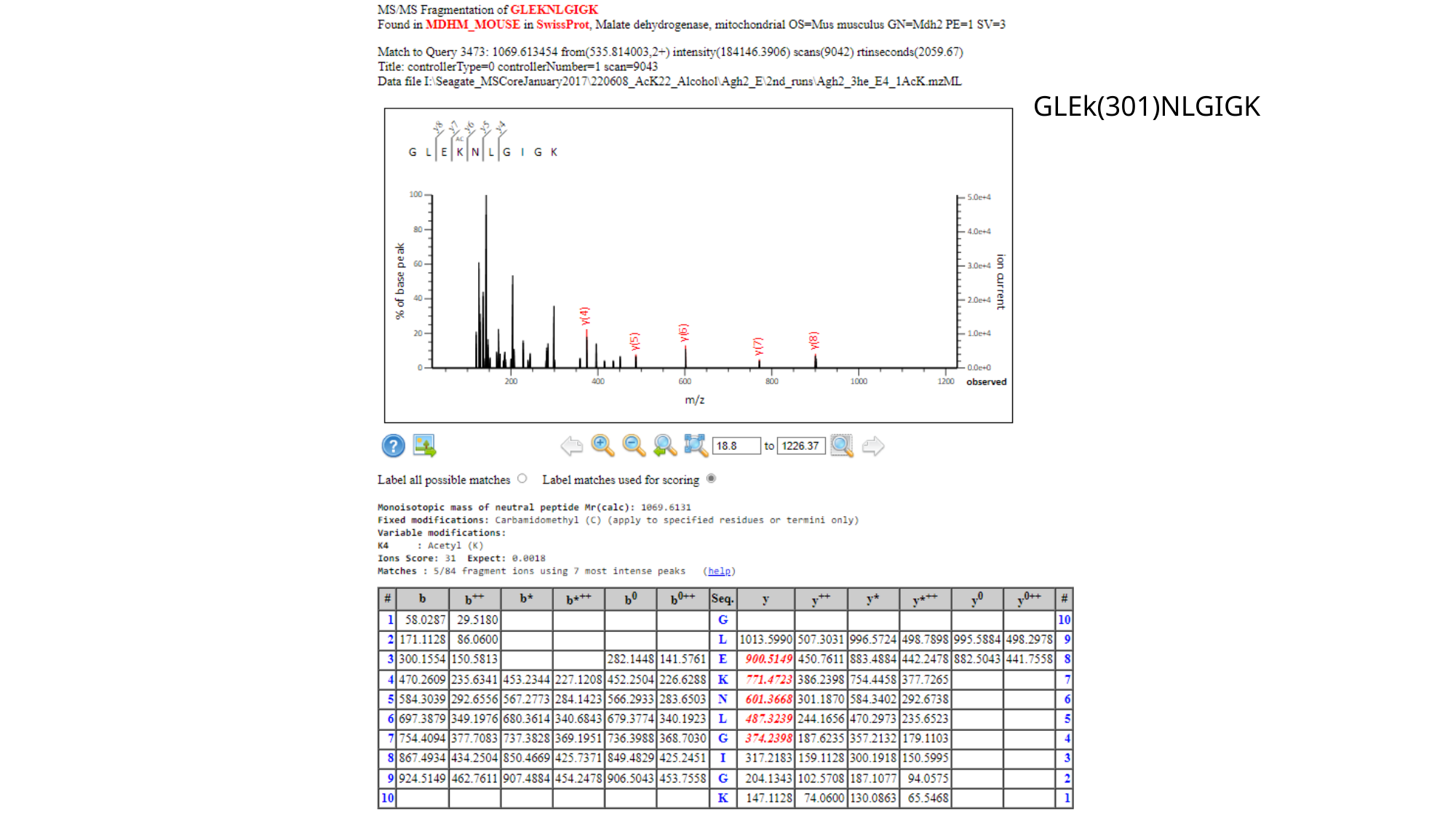

GLEk(301)NLGIGK

## Slide 147
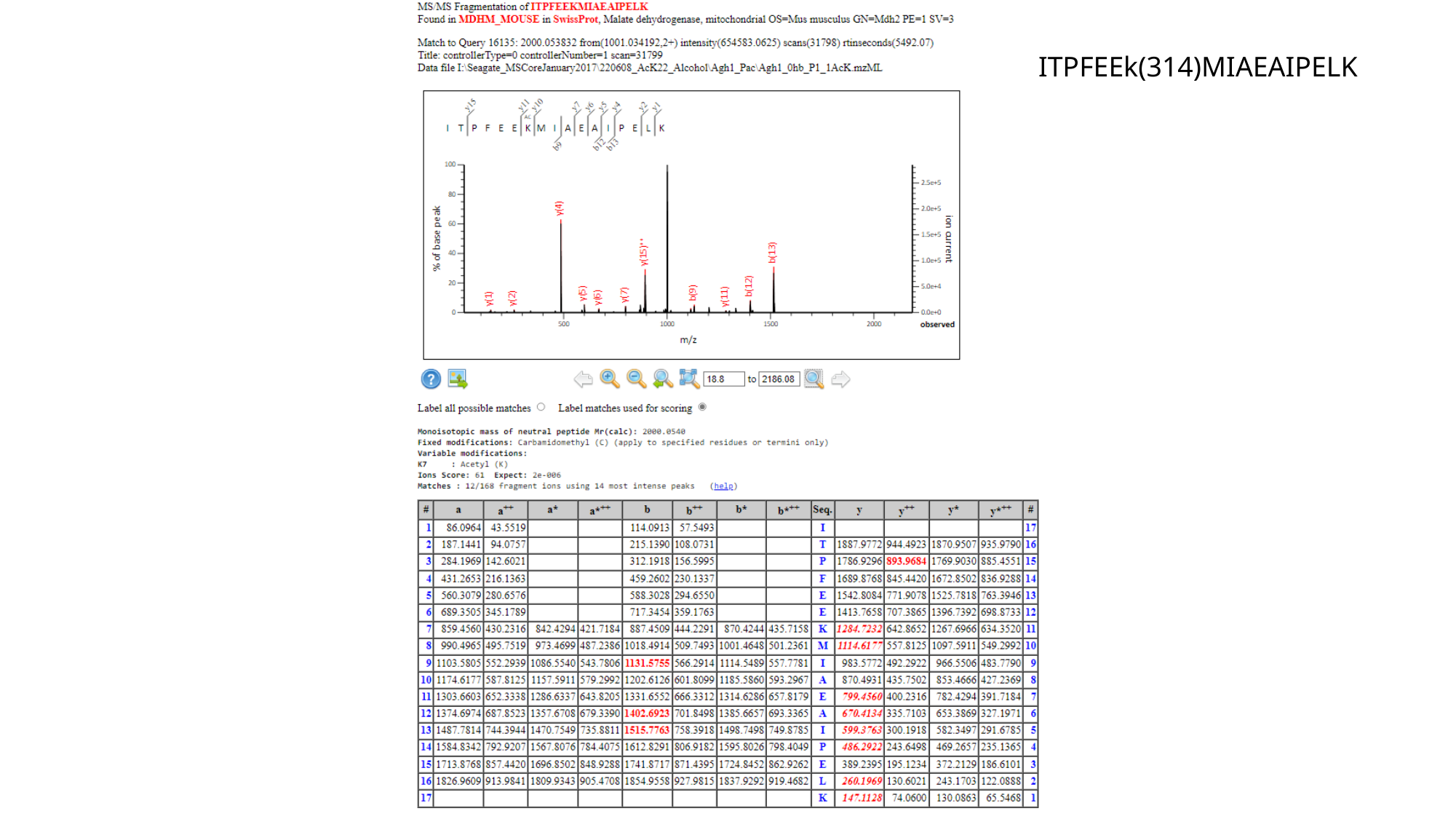

ITPFEEk(314)MIAEAIPELK

## Slide 148
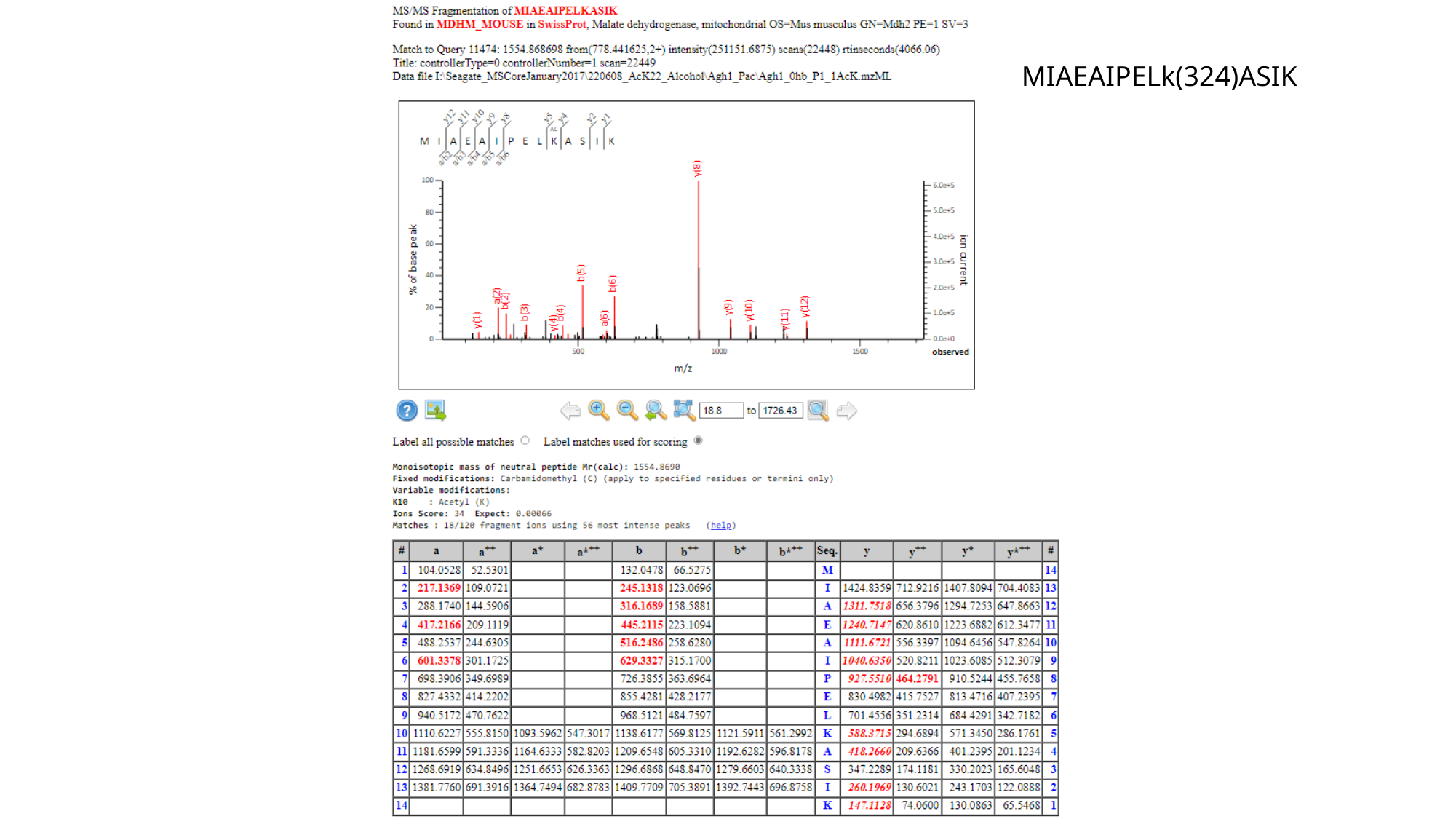

MIAEAIPELk(324)ASIK

## Slide 149
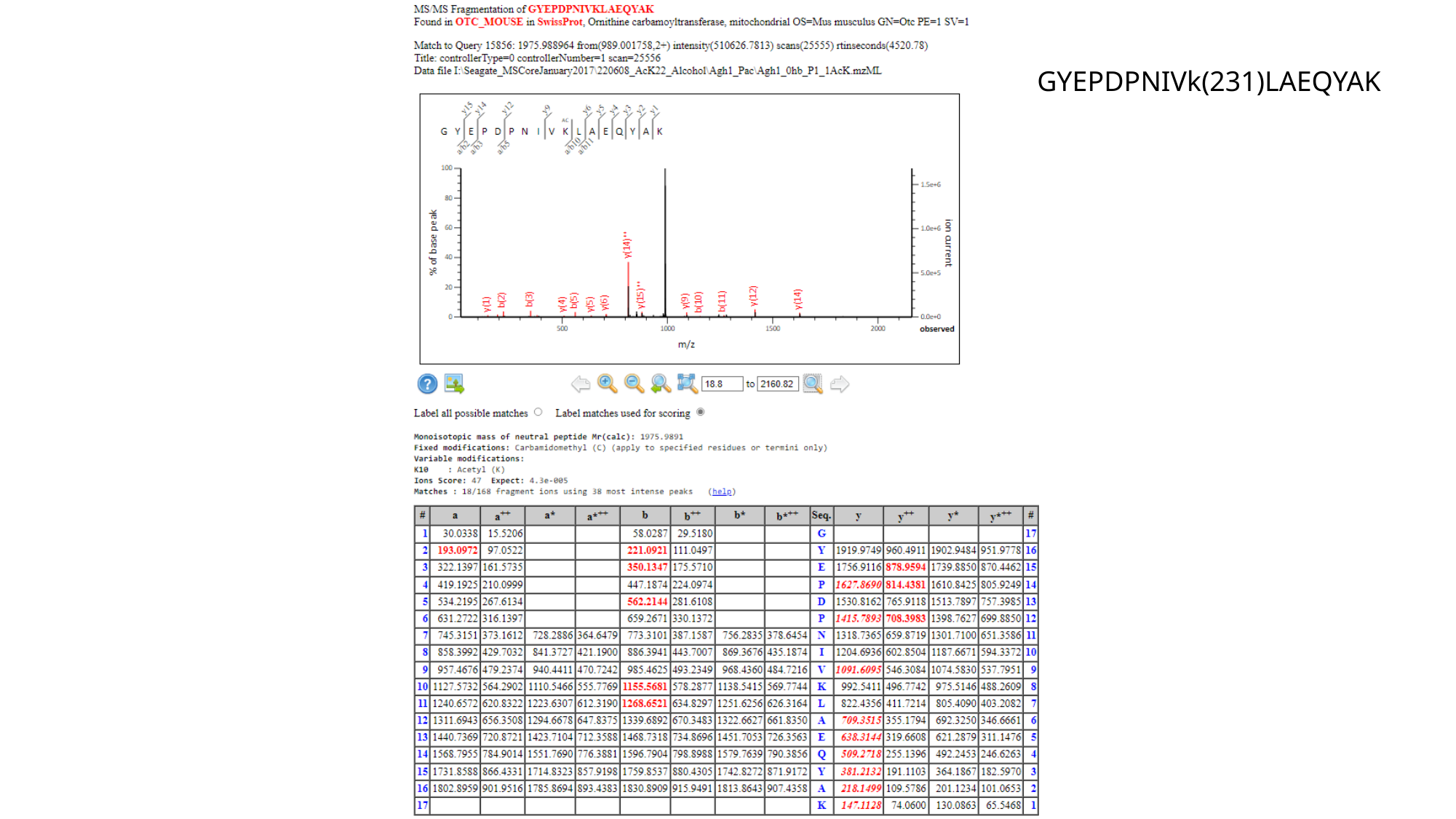

GYEPDPNIVk(231)LAEQYAK

## Slide 150
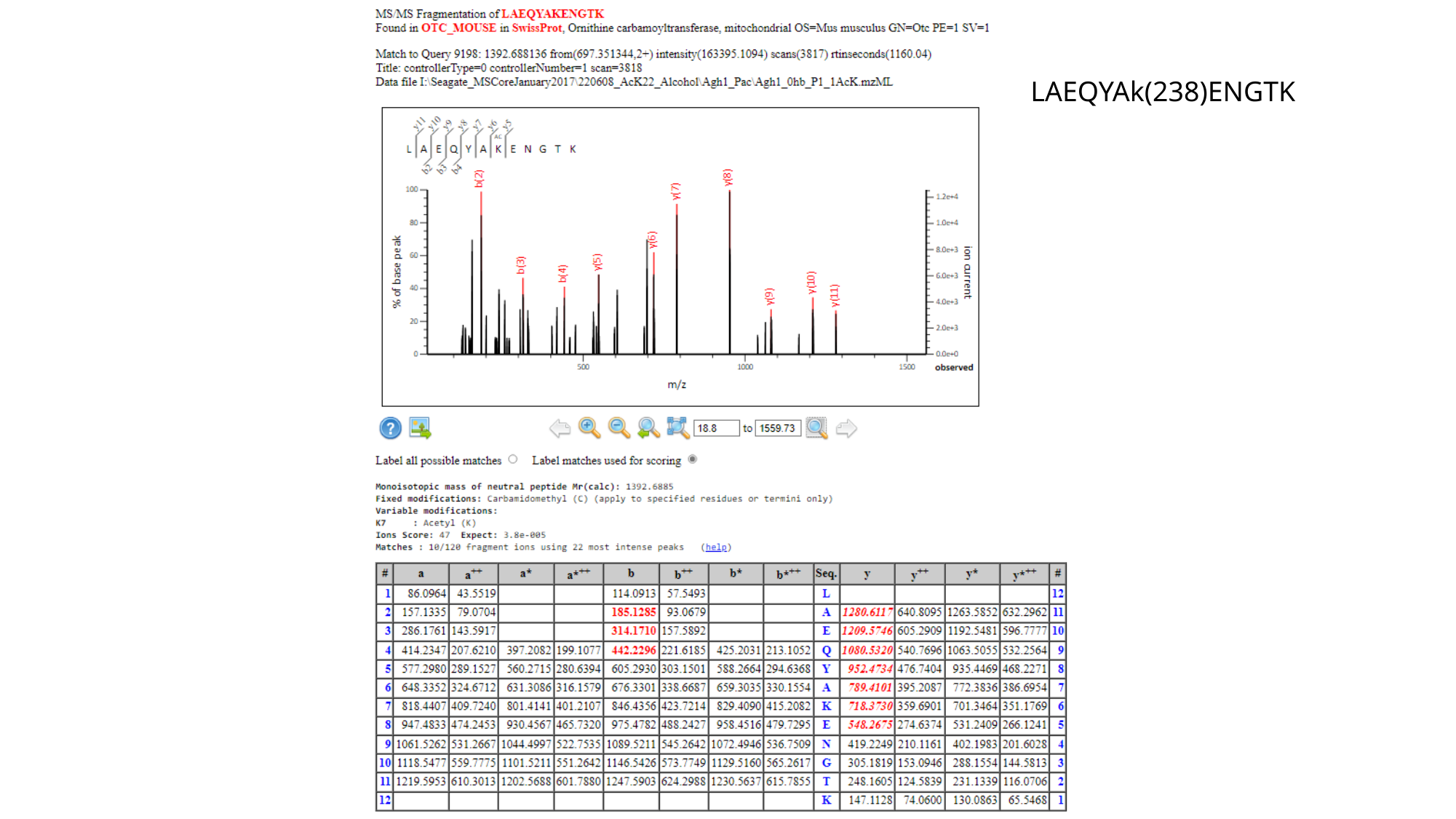

LAEQYAk(238)ENGTK

## Slide 151
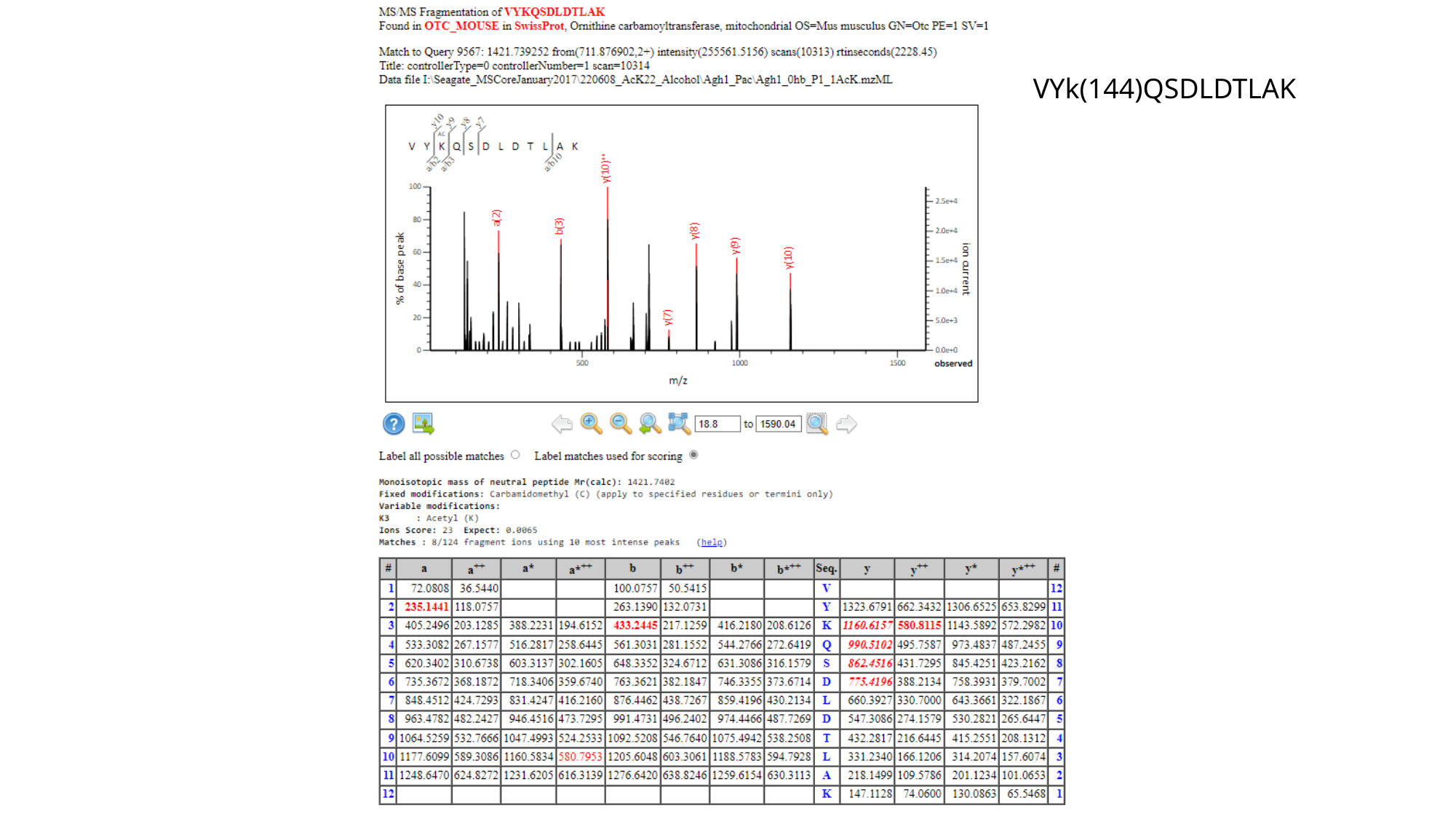

VYk(144)QSDLDTLAK

## Slide 152
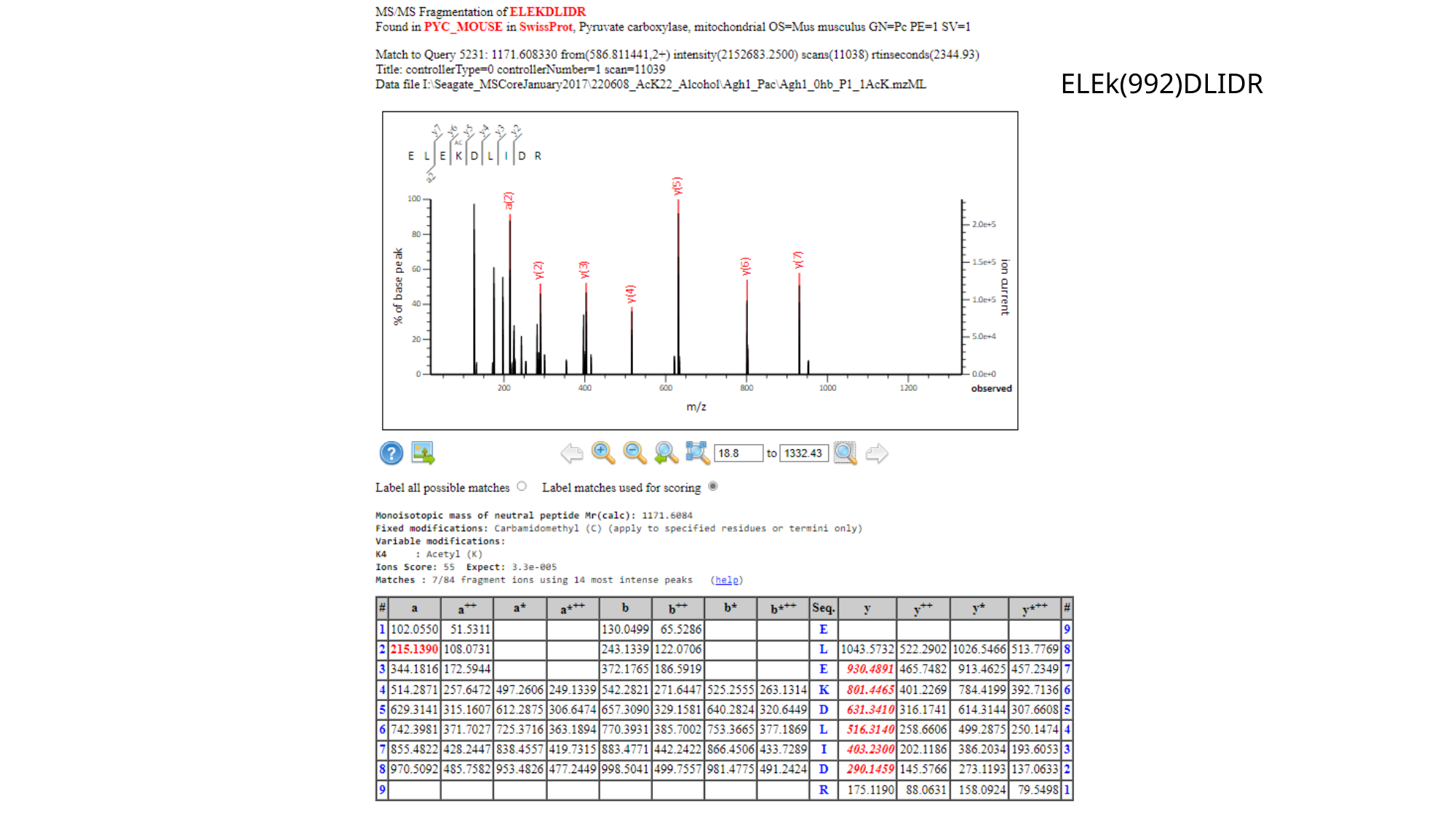

ELEk(992)DLIDR

## Slide 153
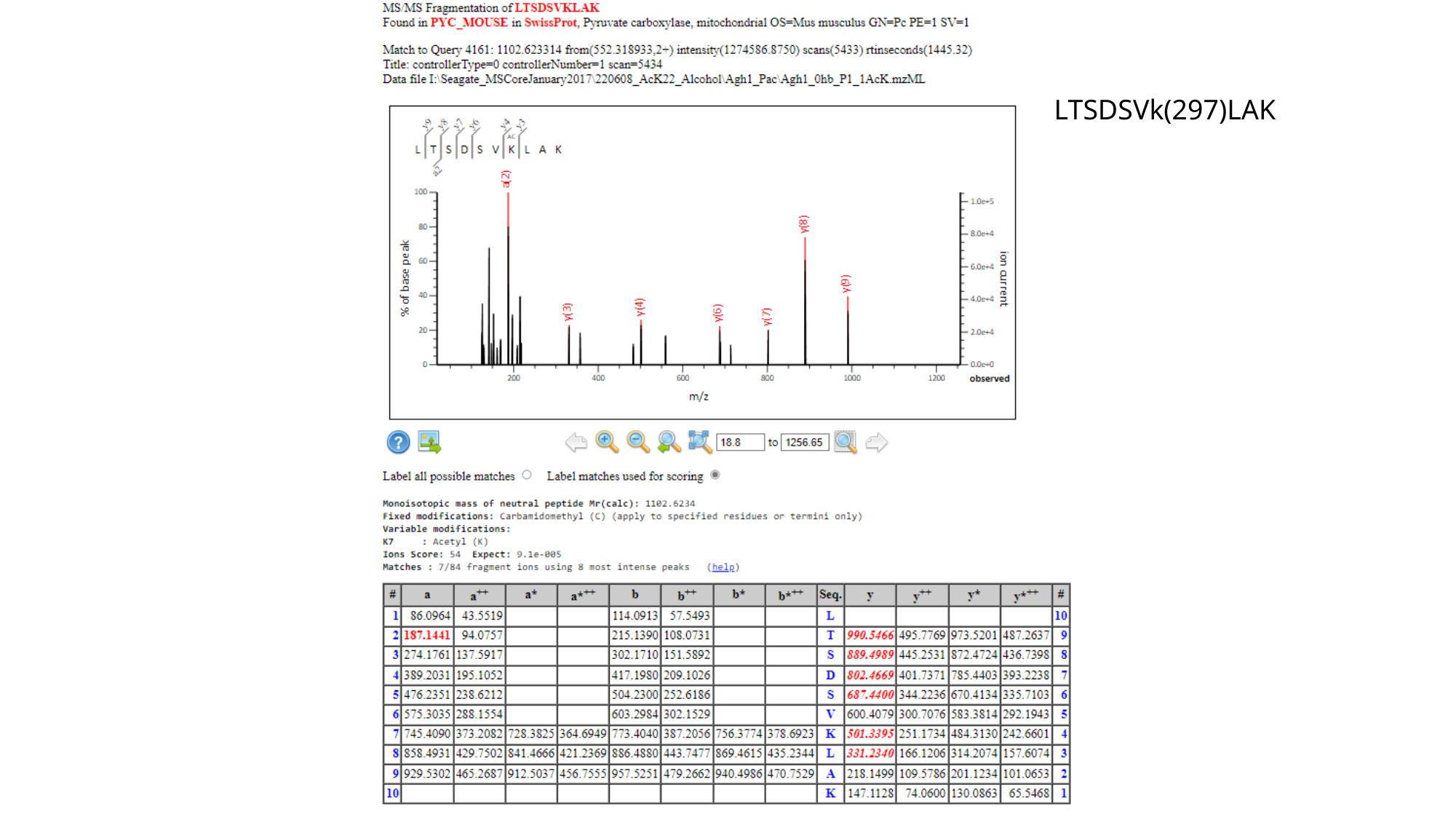

LTSDSVk(297)LAK

## Slide 154
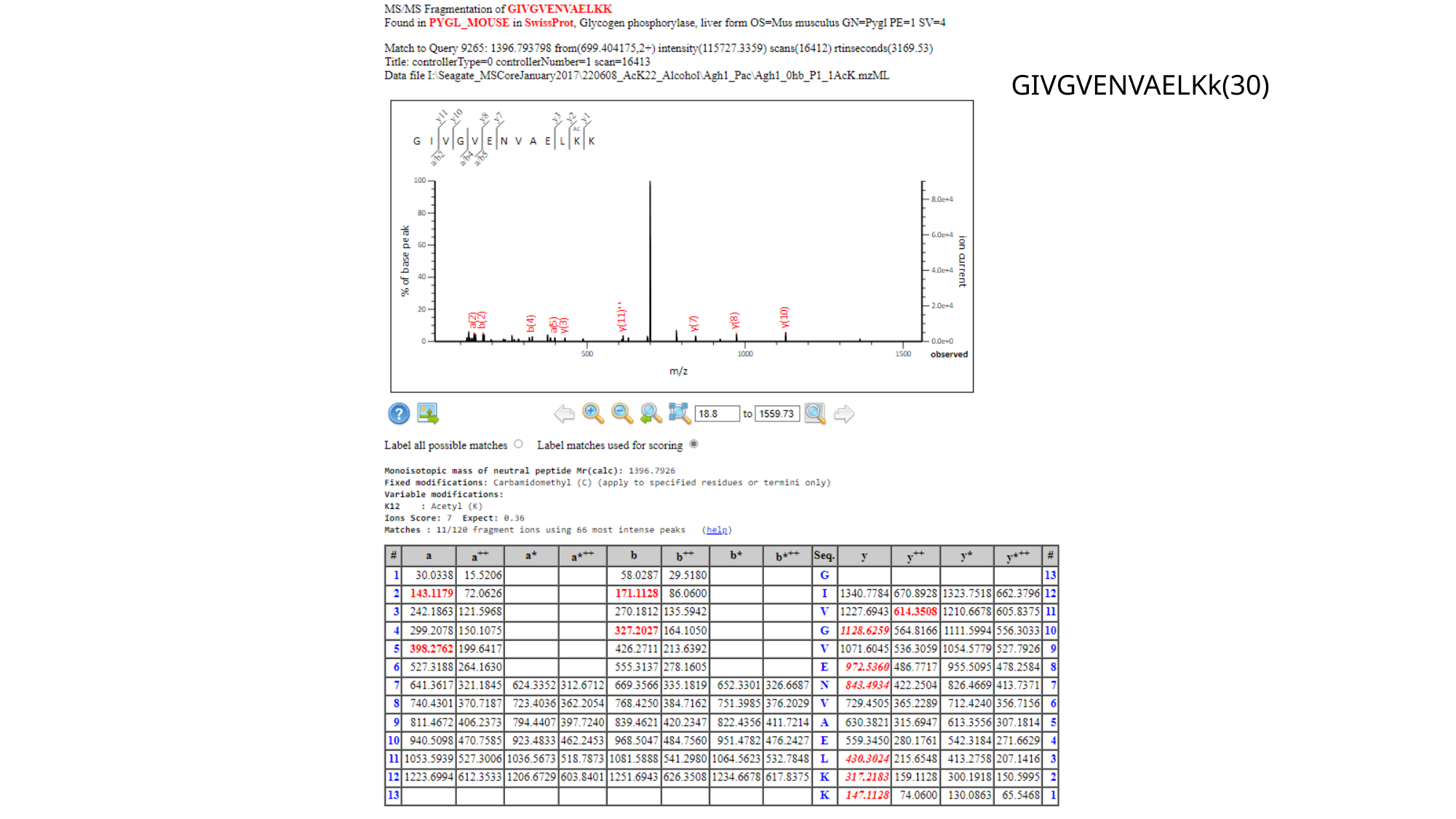

GIVGVENVAELKk(30)

## Slide 155
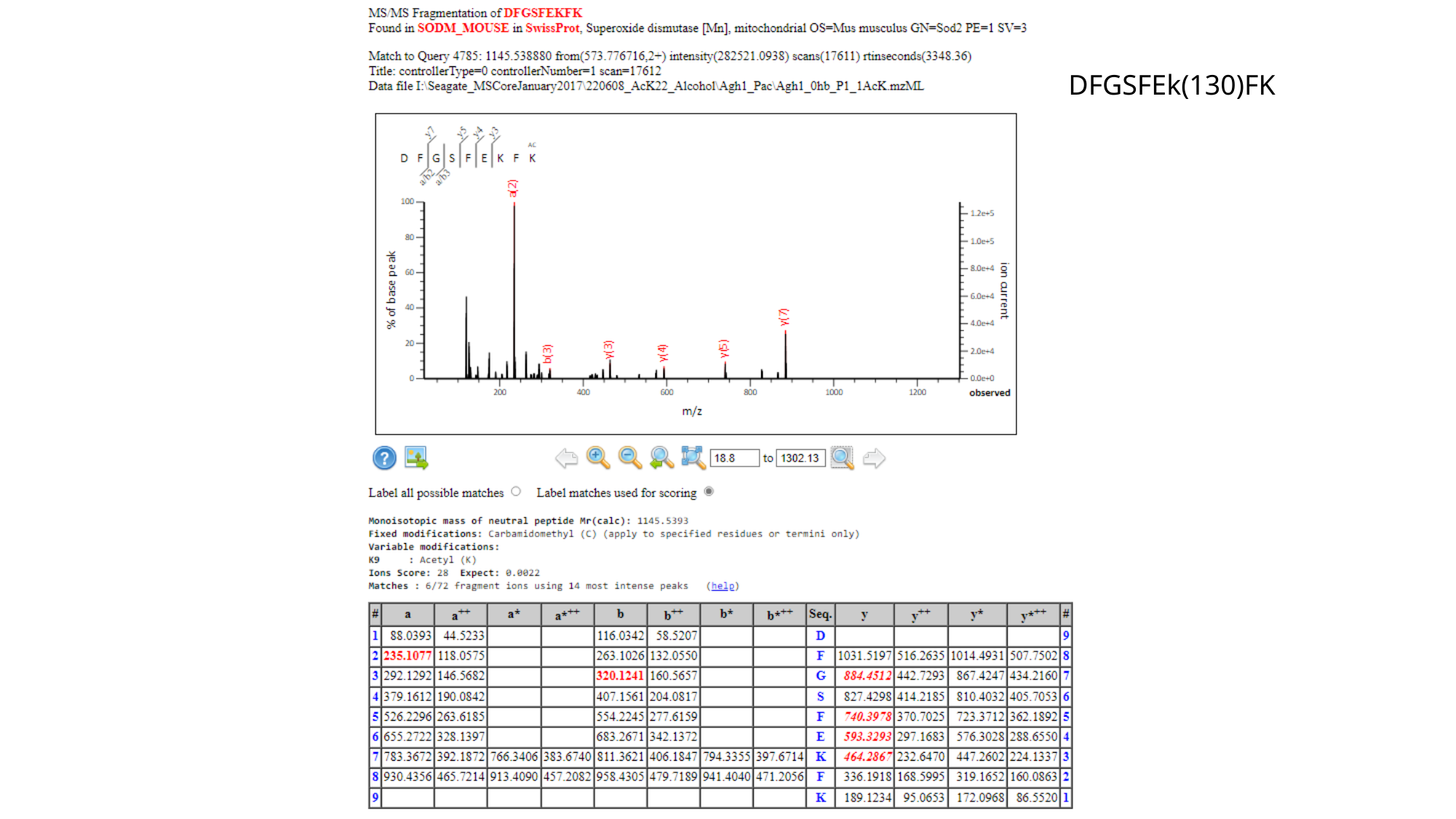

DFGSFEk(130)FK

## Slide 156
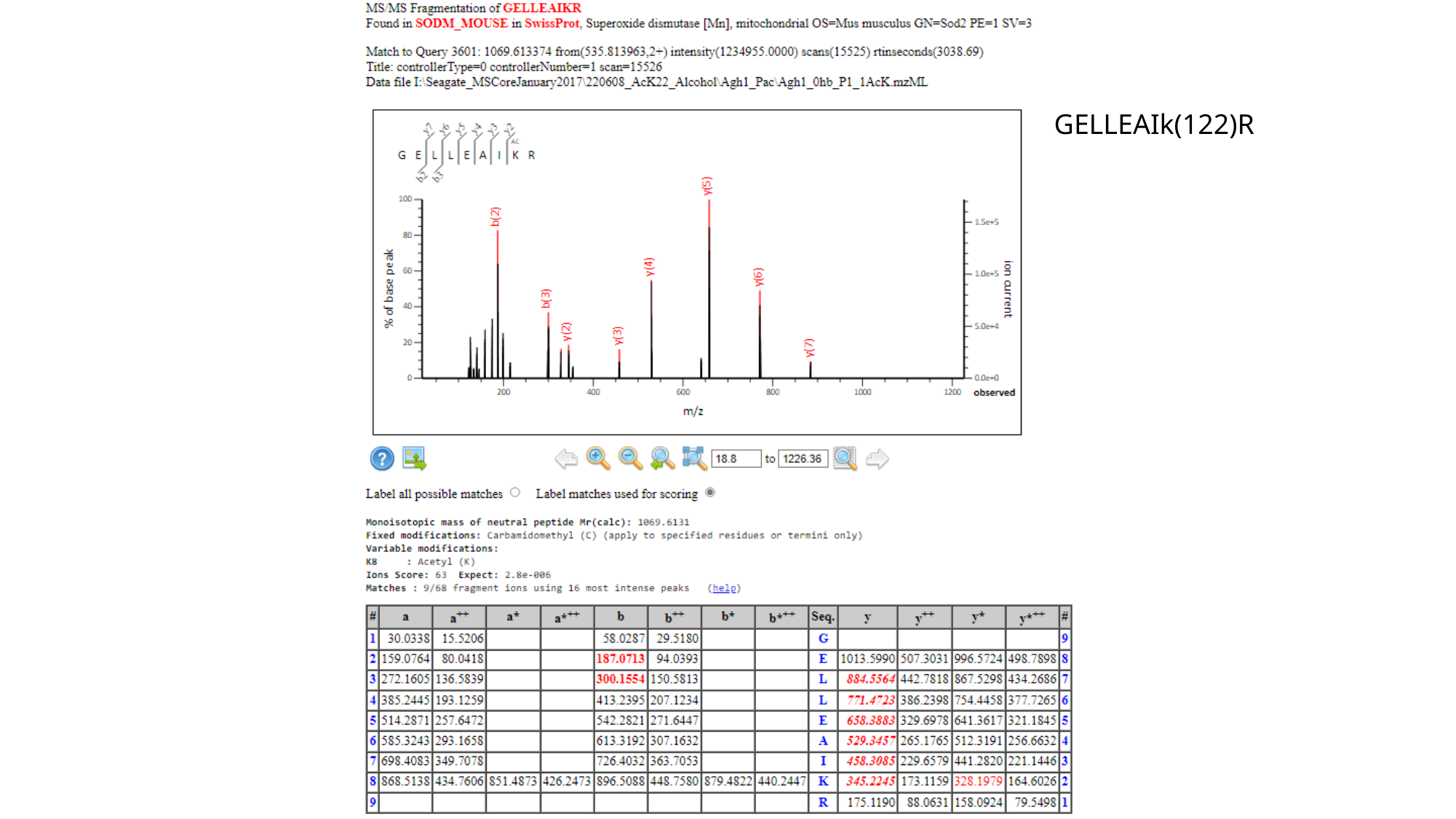

GELLEAIk(122)R

## Slide 157
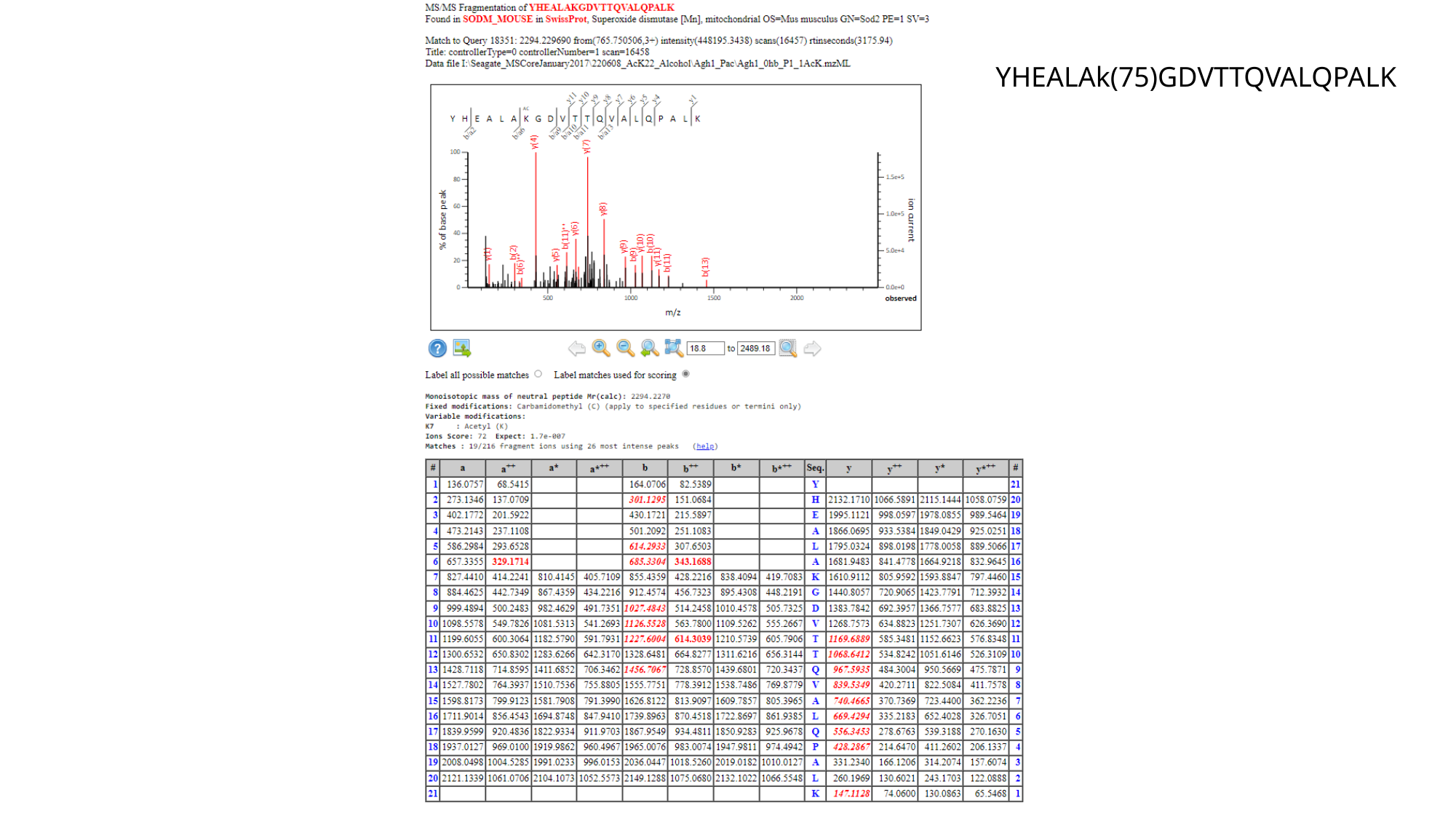

YHEALAk(75)GDVTTQVALQPALK

## Slide 158
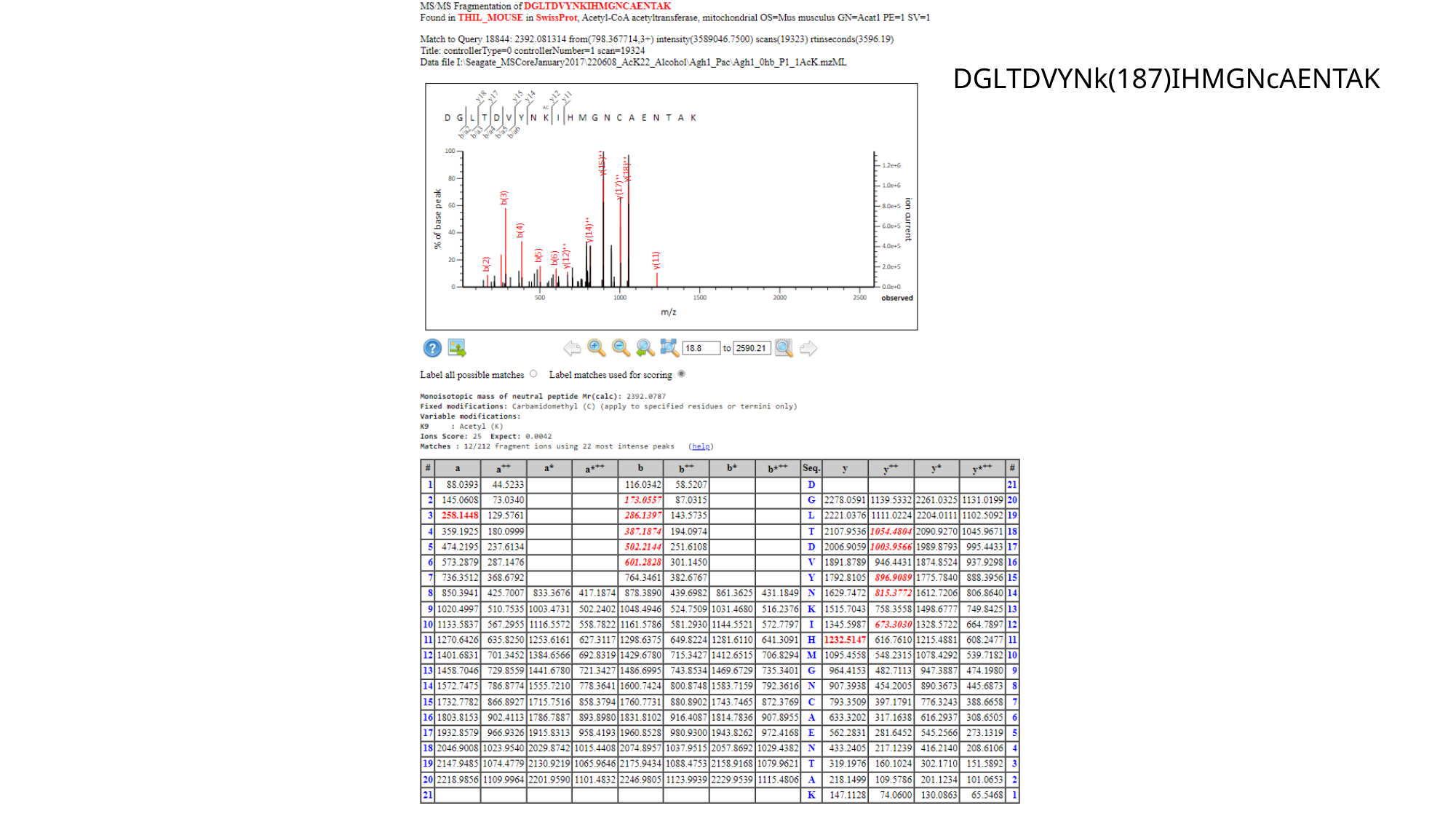

DGLTDVYNk(187)IHMGNcAENTAK

## Slide 159
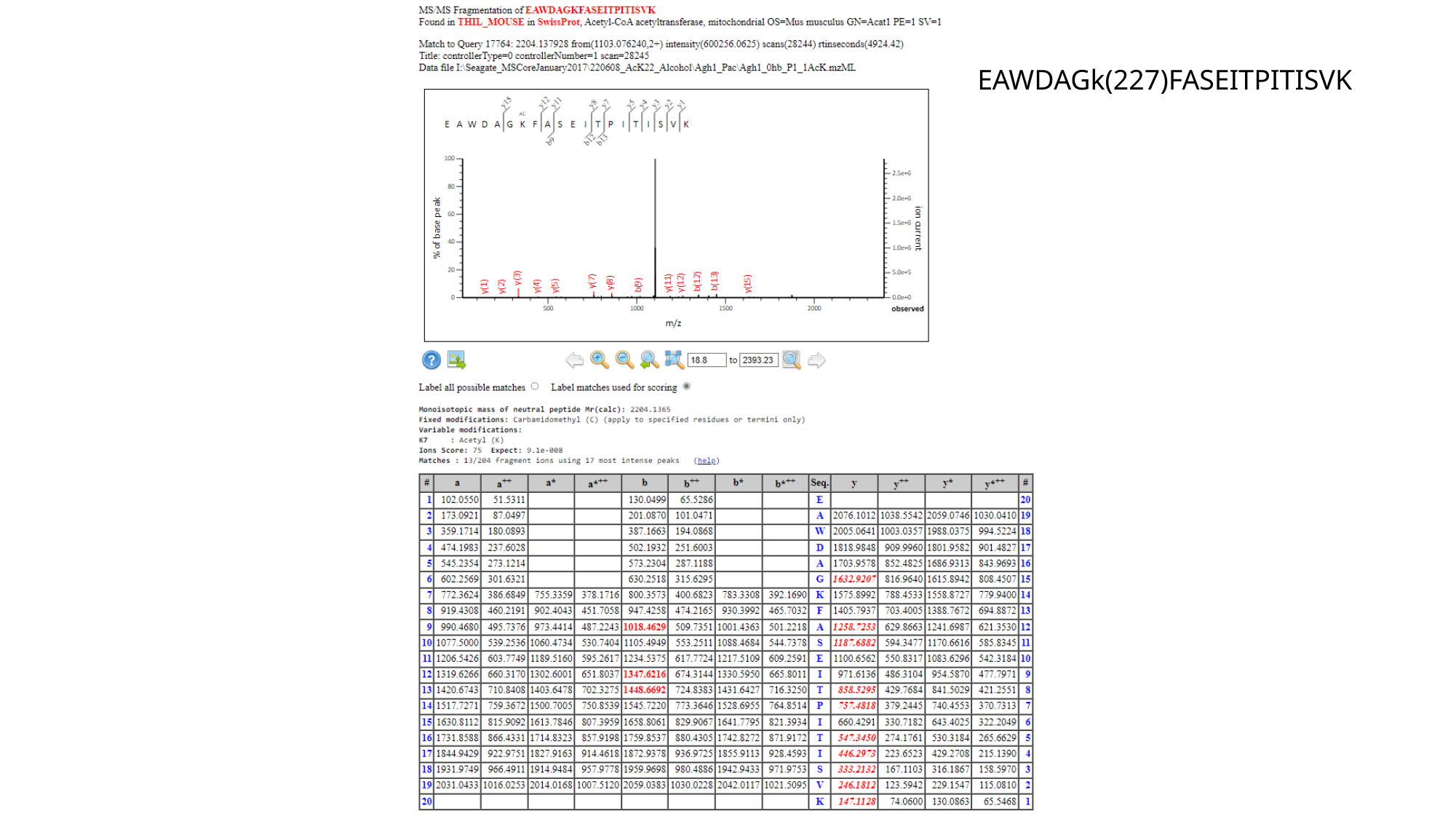

EAWDAGk(227)FASEITPITISVK

## Slide 160
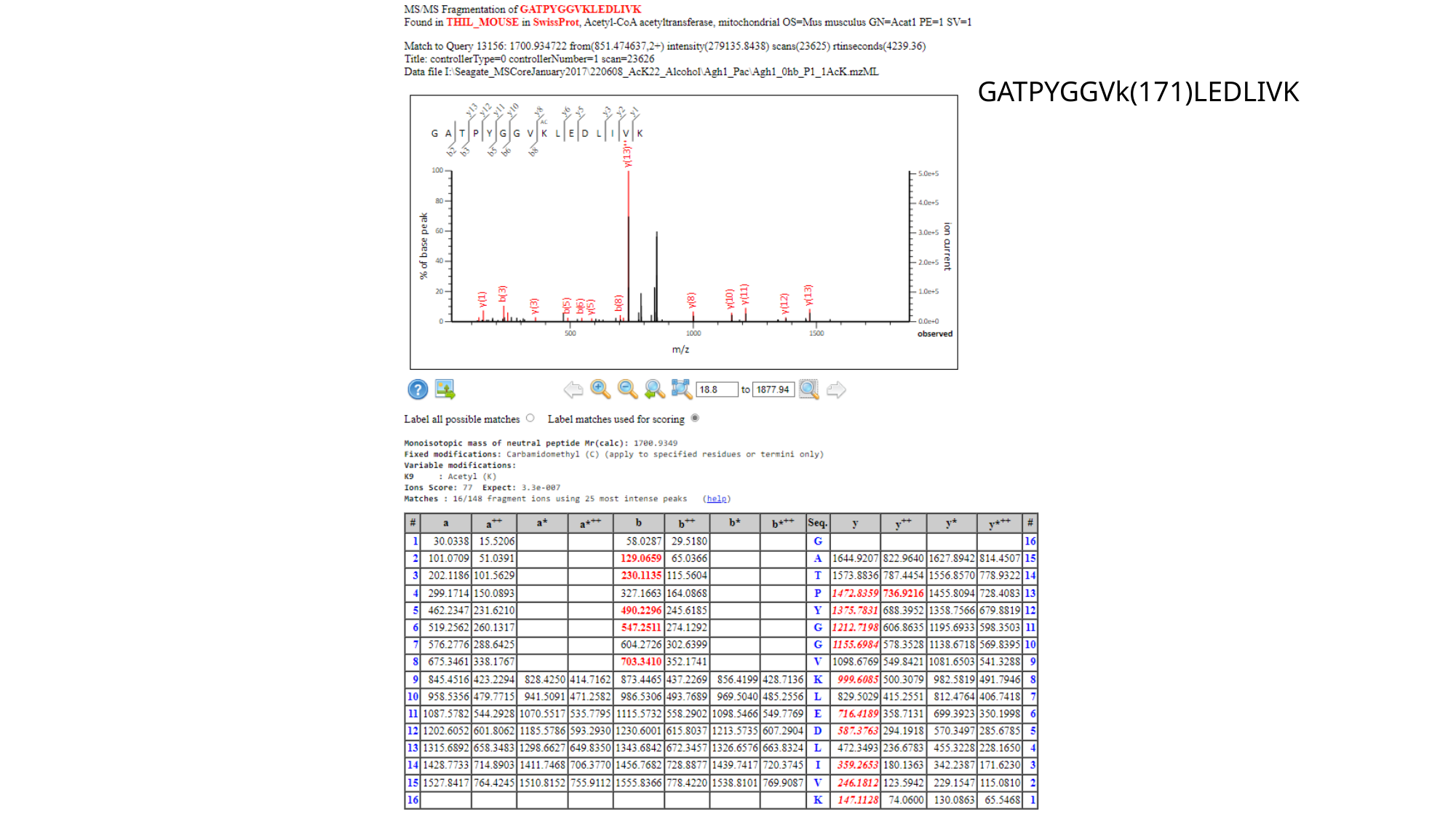

GATPYGGVk(171)LEDLIVK

## Slide 161
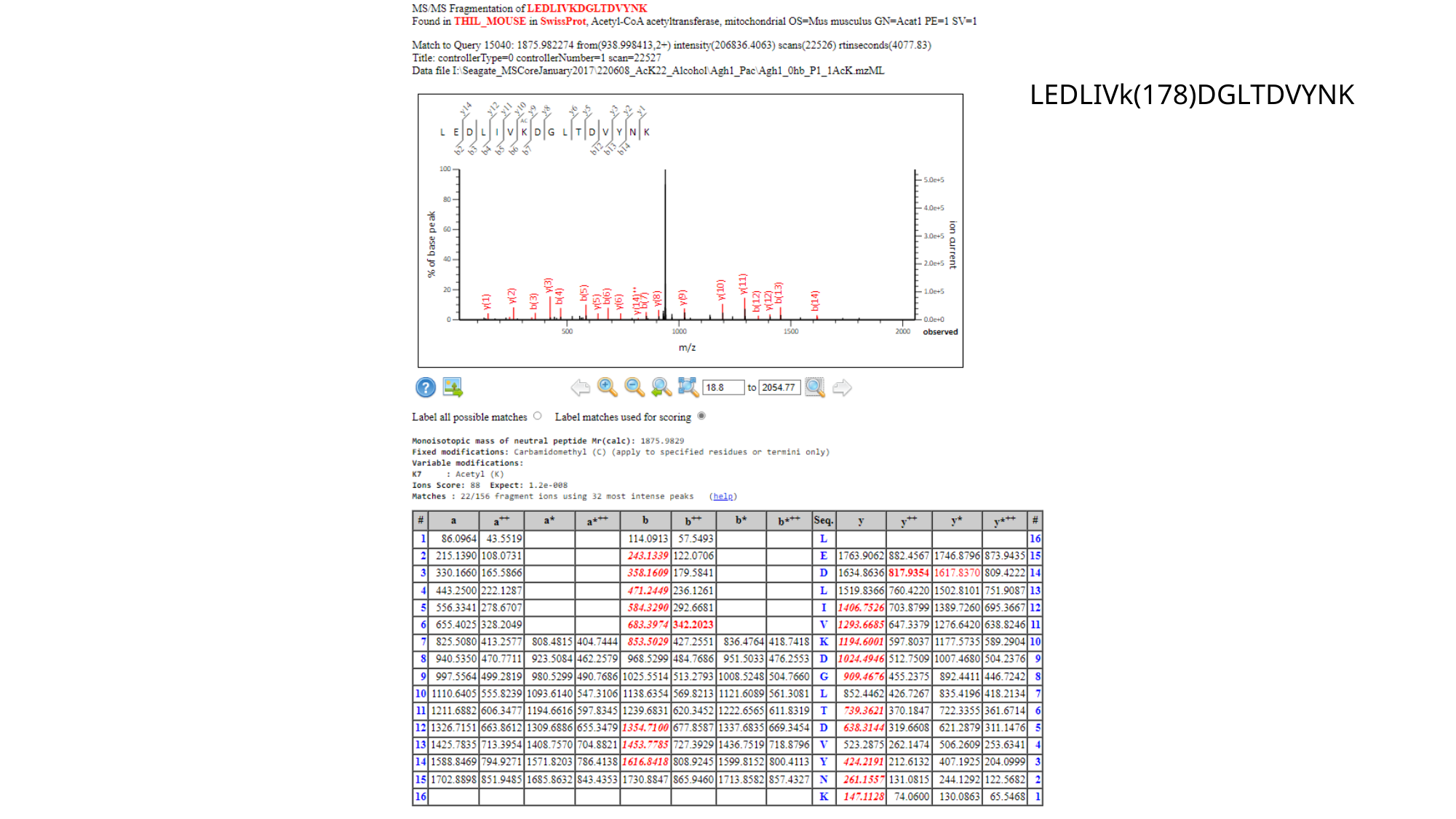

LEDLIVk(178)DGLTDVYNK

## Slide 162
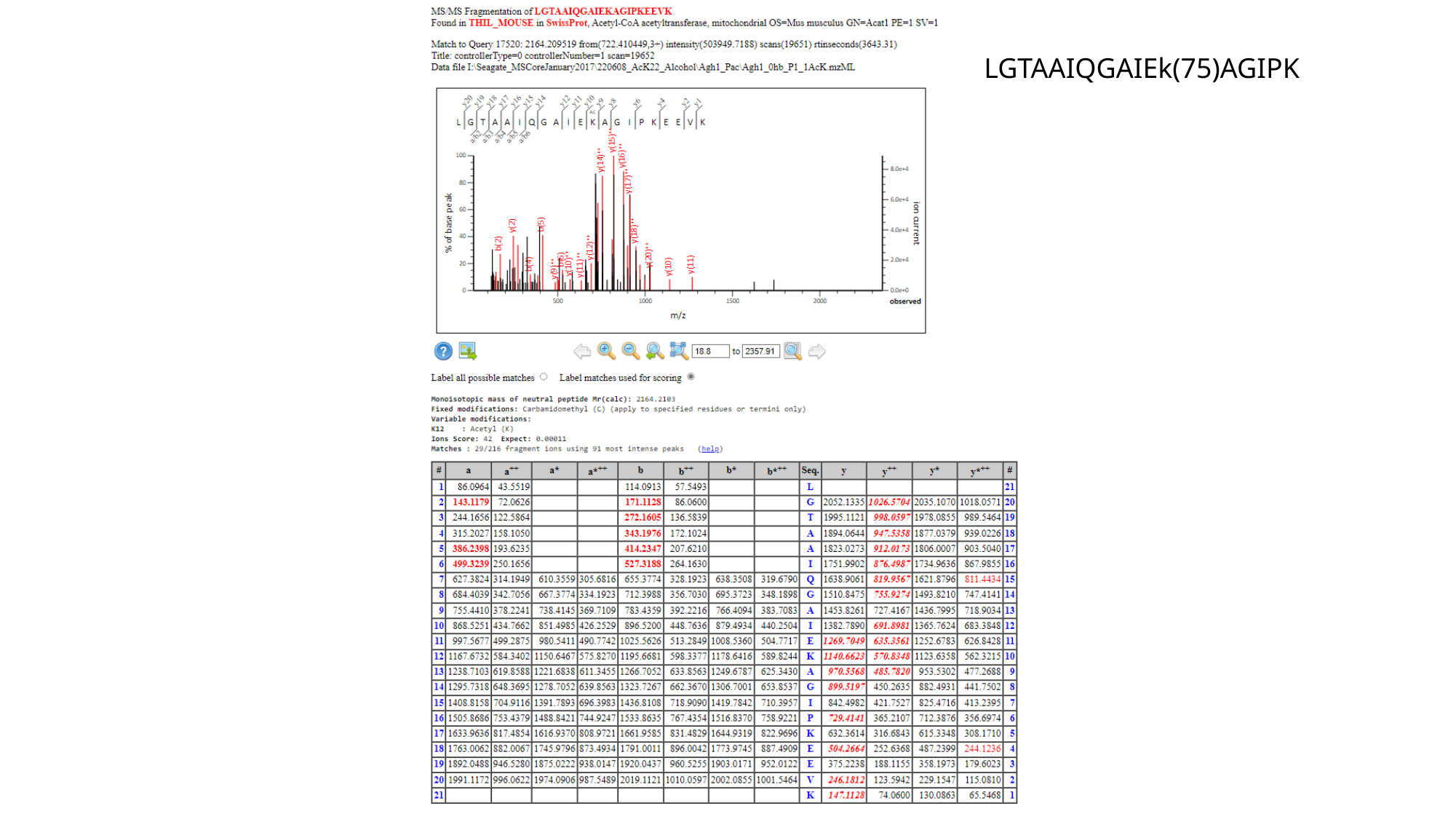

LGTAAIQGAIEk(75)AGIPK

## Slide 163
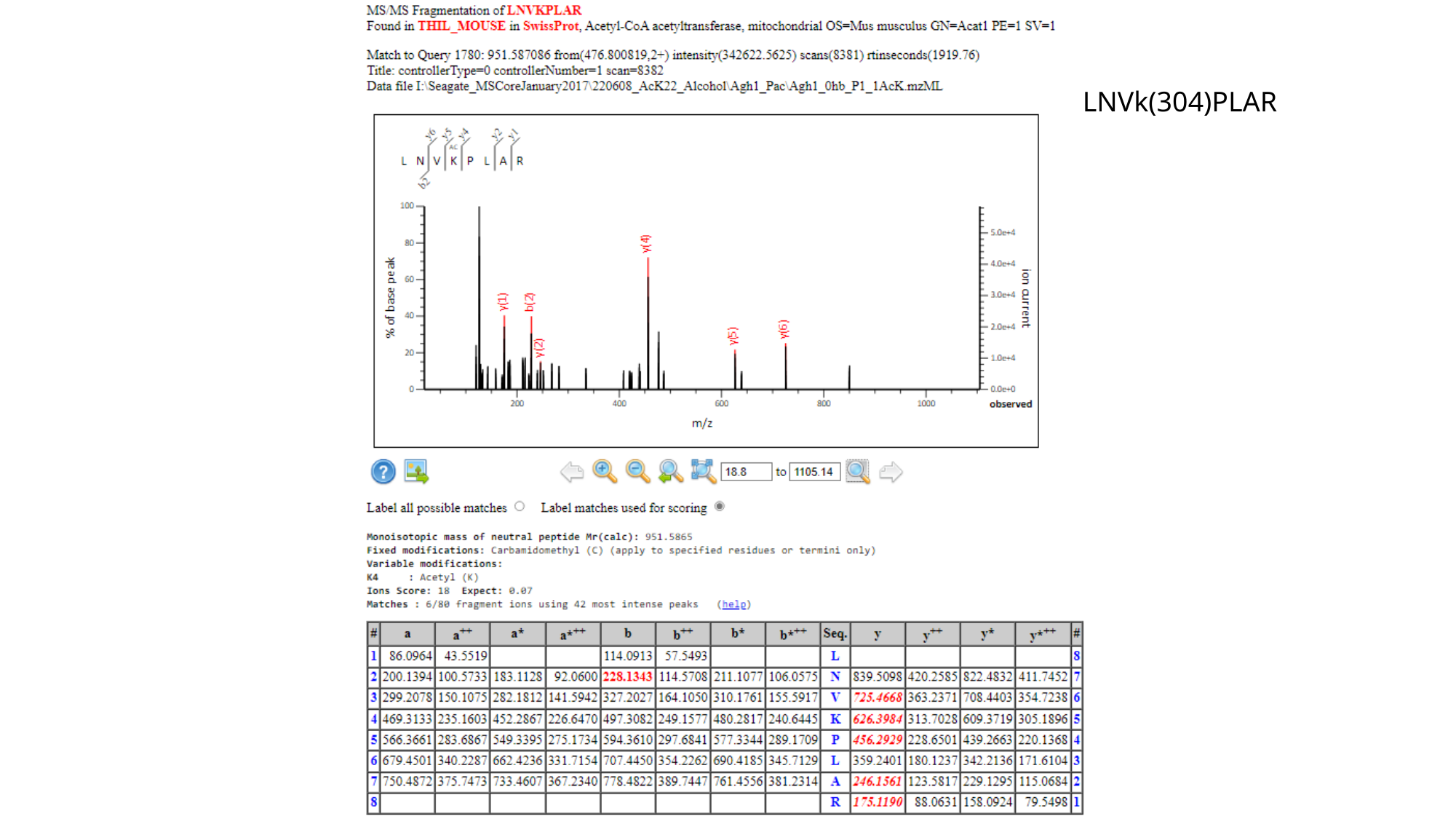

LNVk(304)PLAR

## Slide 164
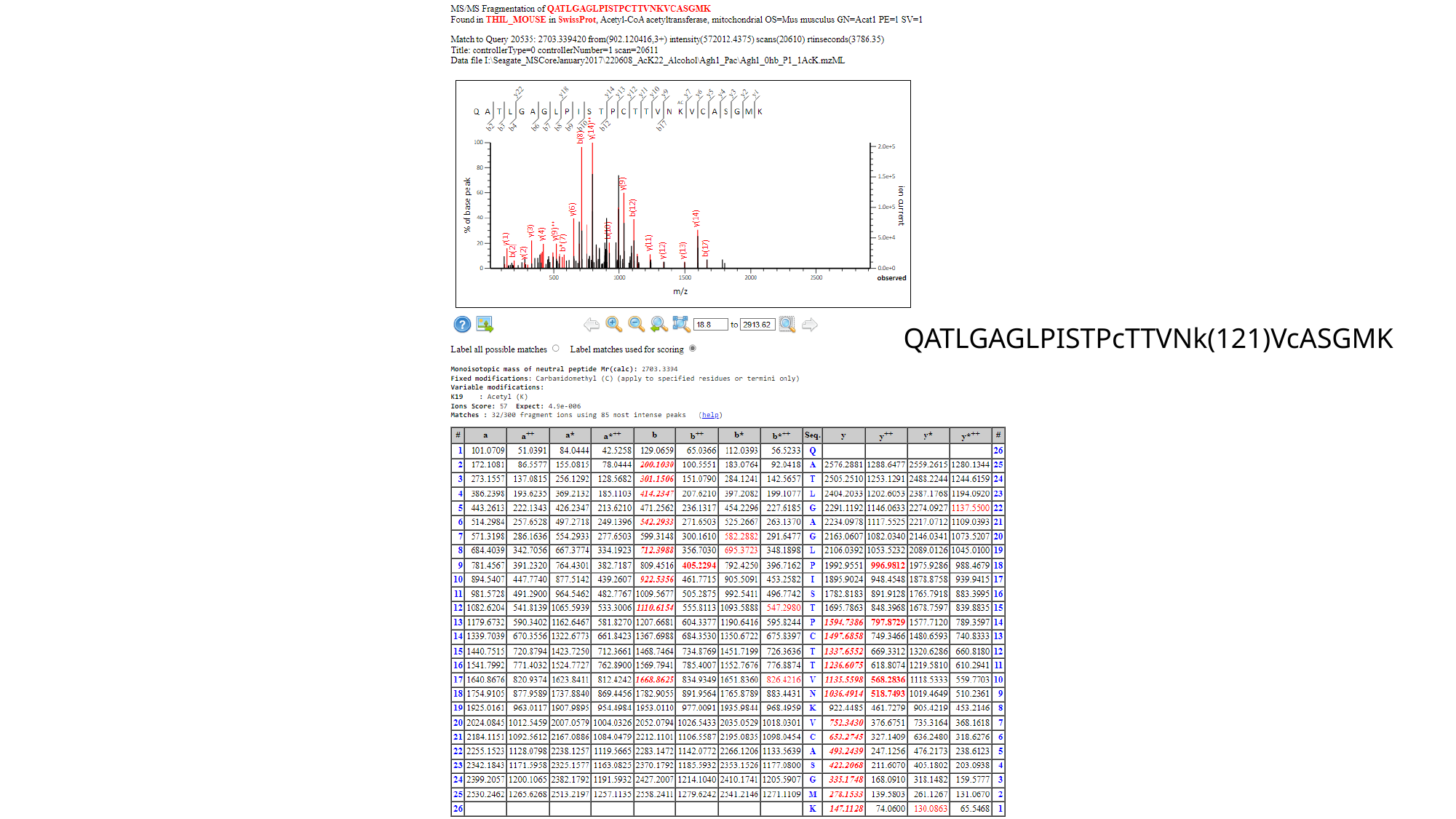

QATLGAGLPISTPcTTVNk(121)VcASGMK

## Slide 165
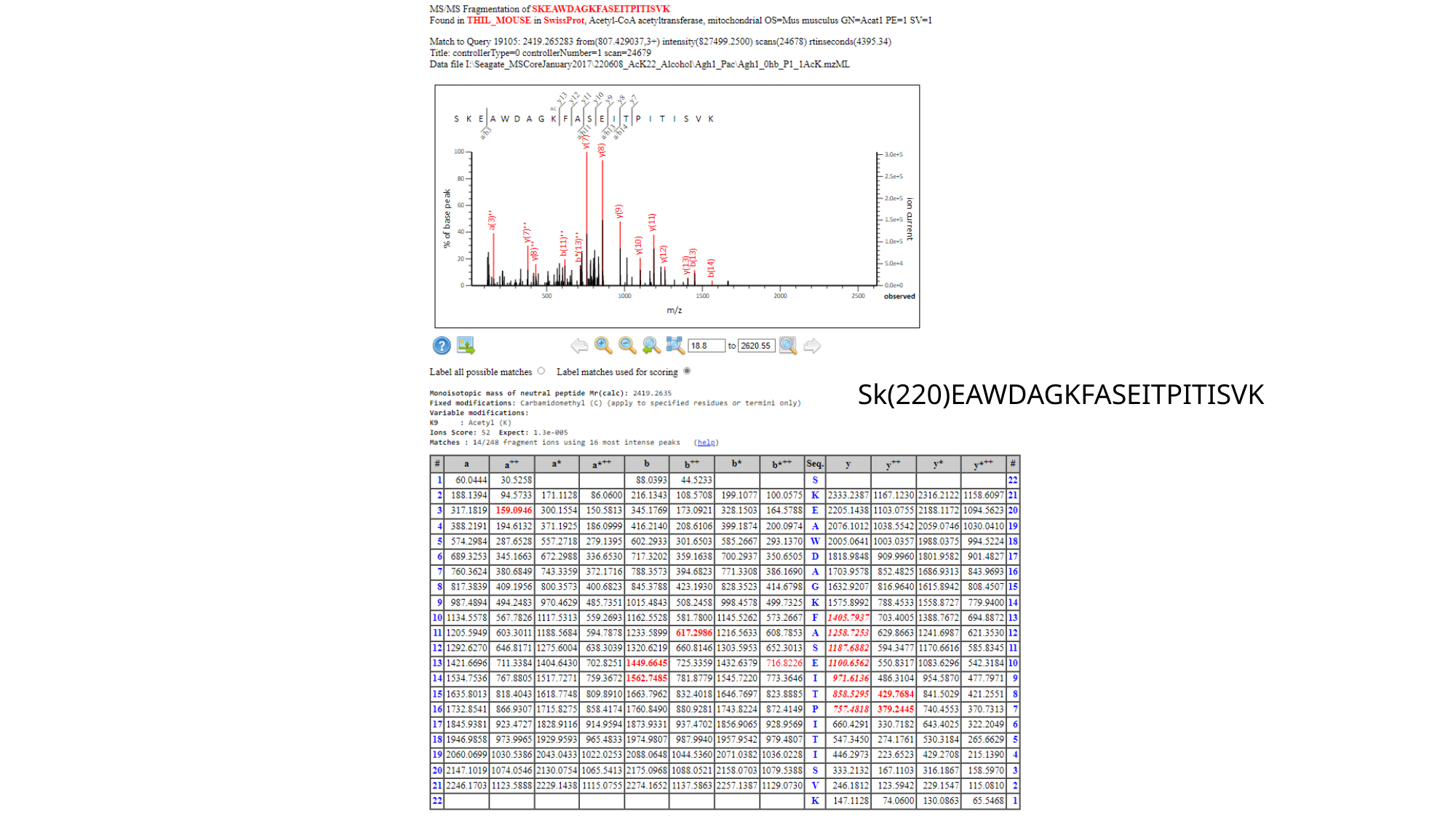

Sk(220)EAWDAGKFASEITPITISVK

## Slide 166
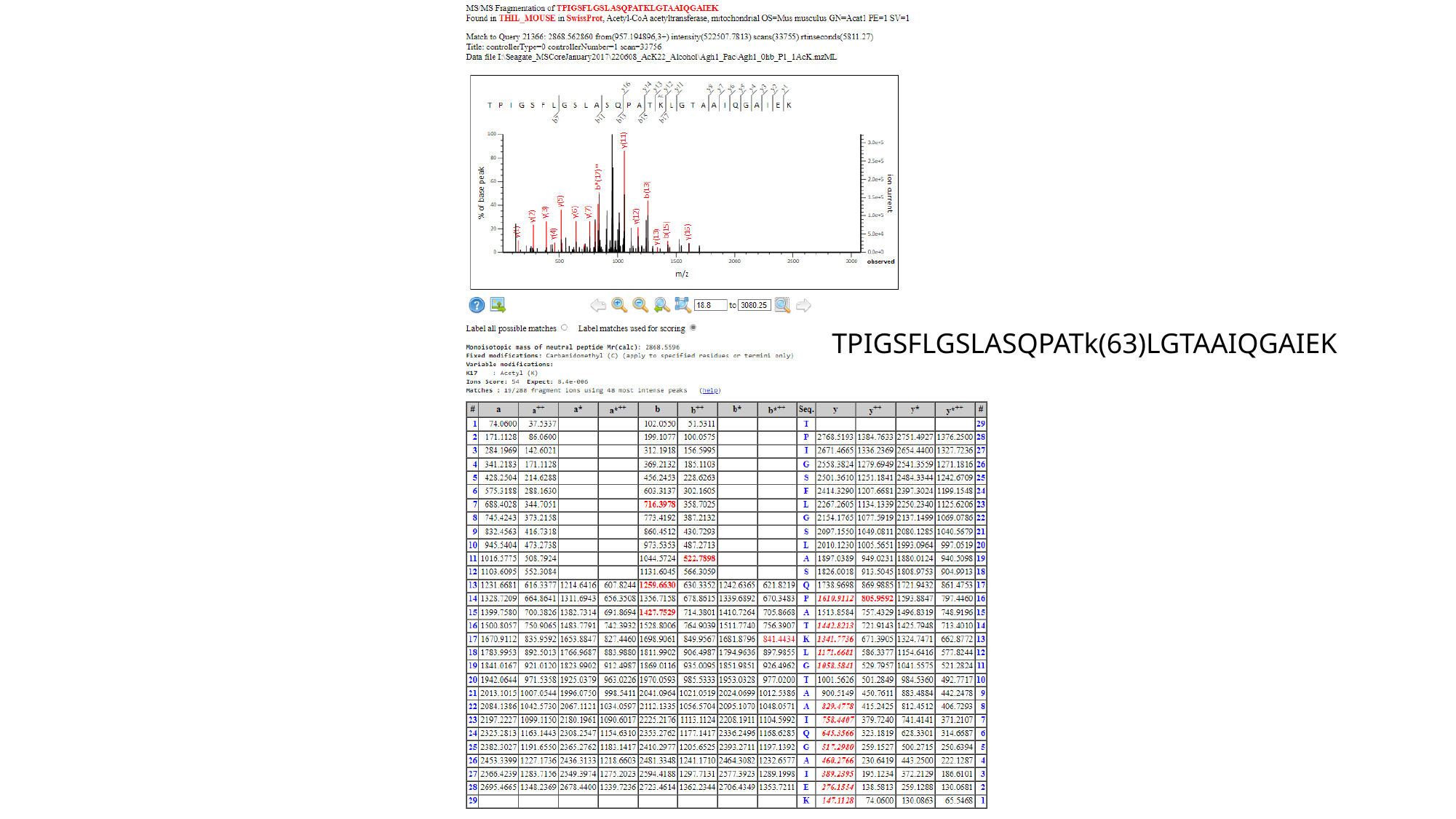

TPIGSFLGSLASQPATk(63)LGTAAIQGAIEK

## Slide 167
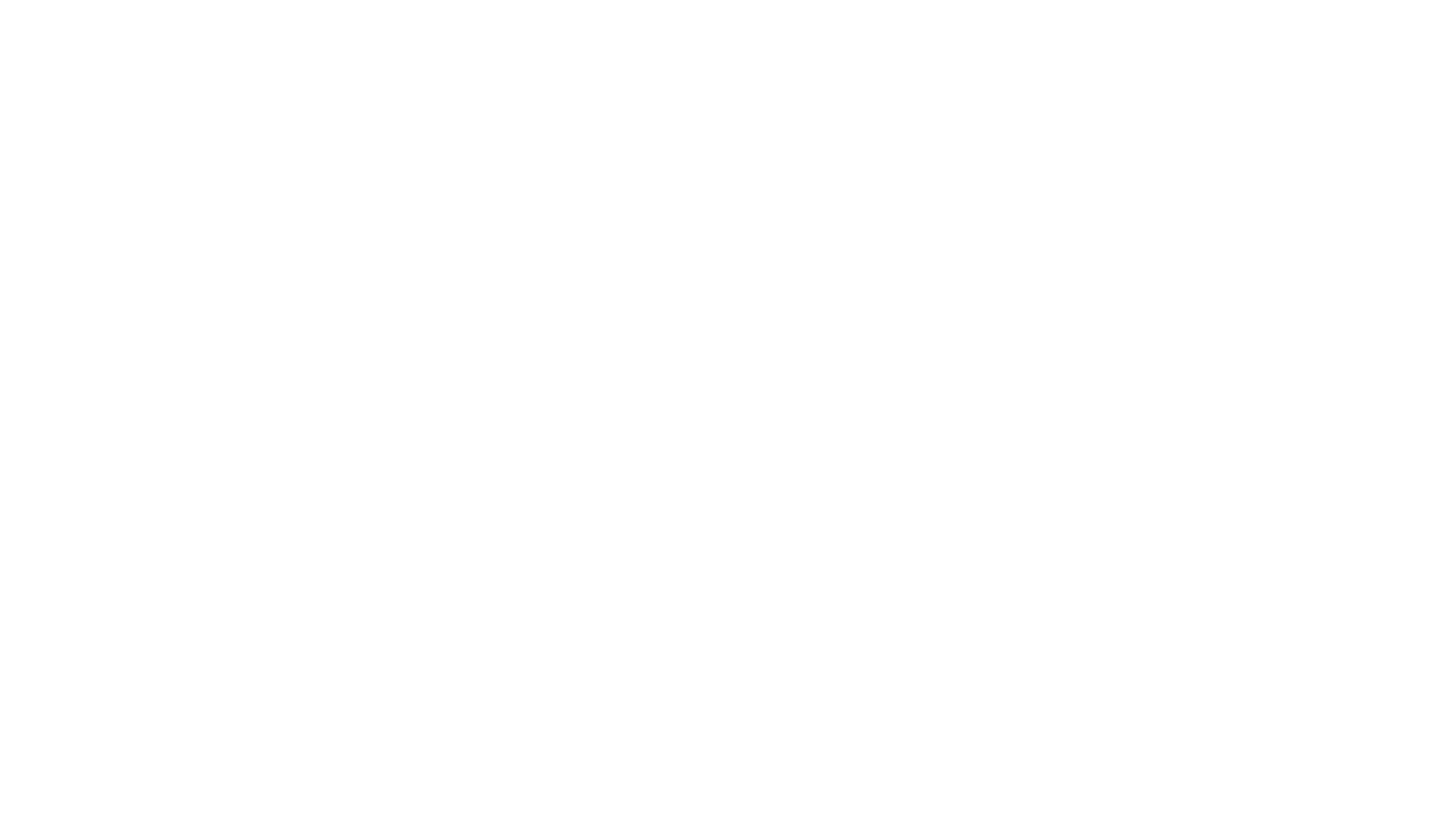

## Slide 168
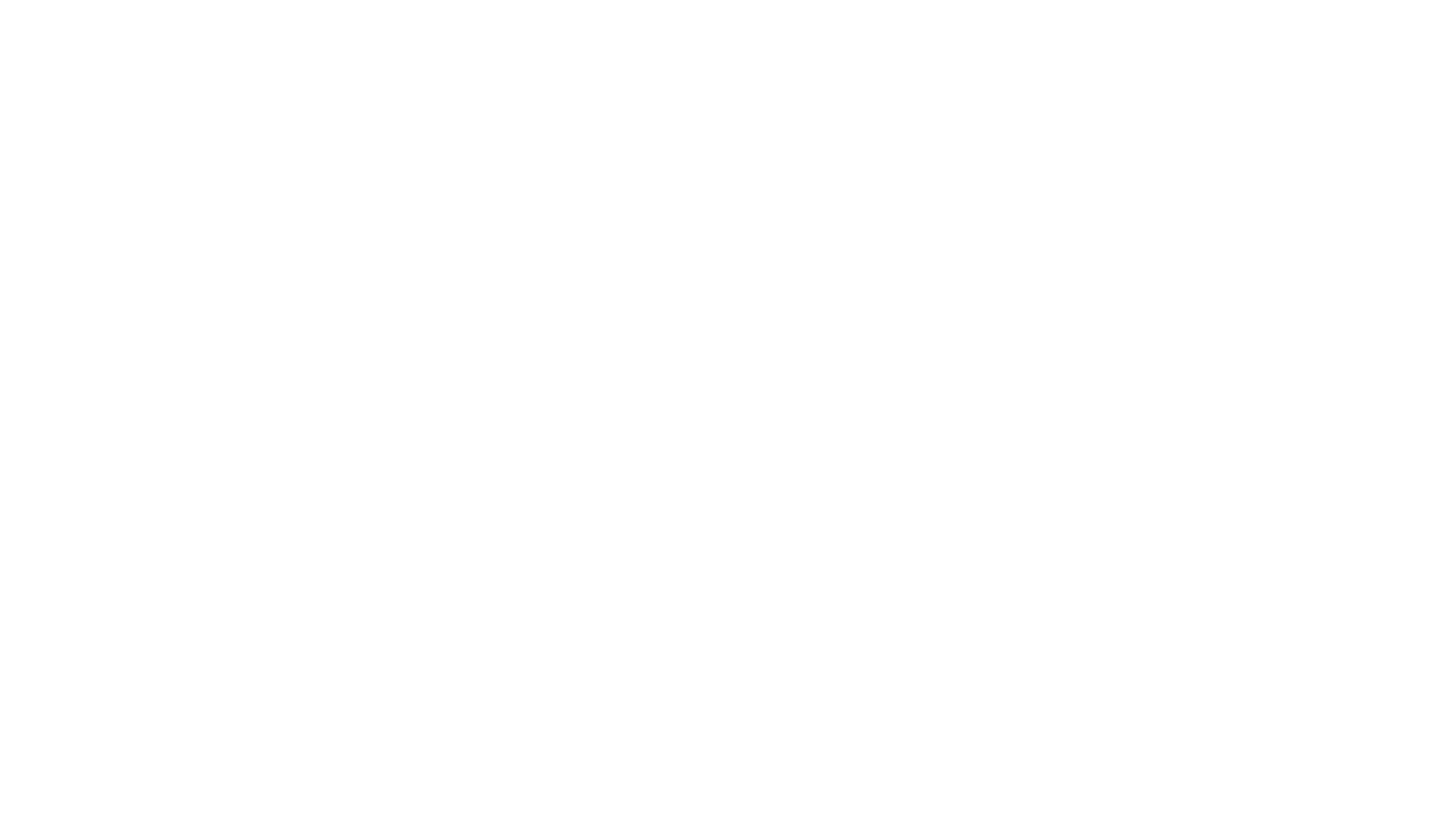

## Slide 169
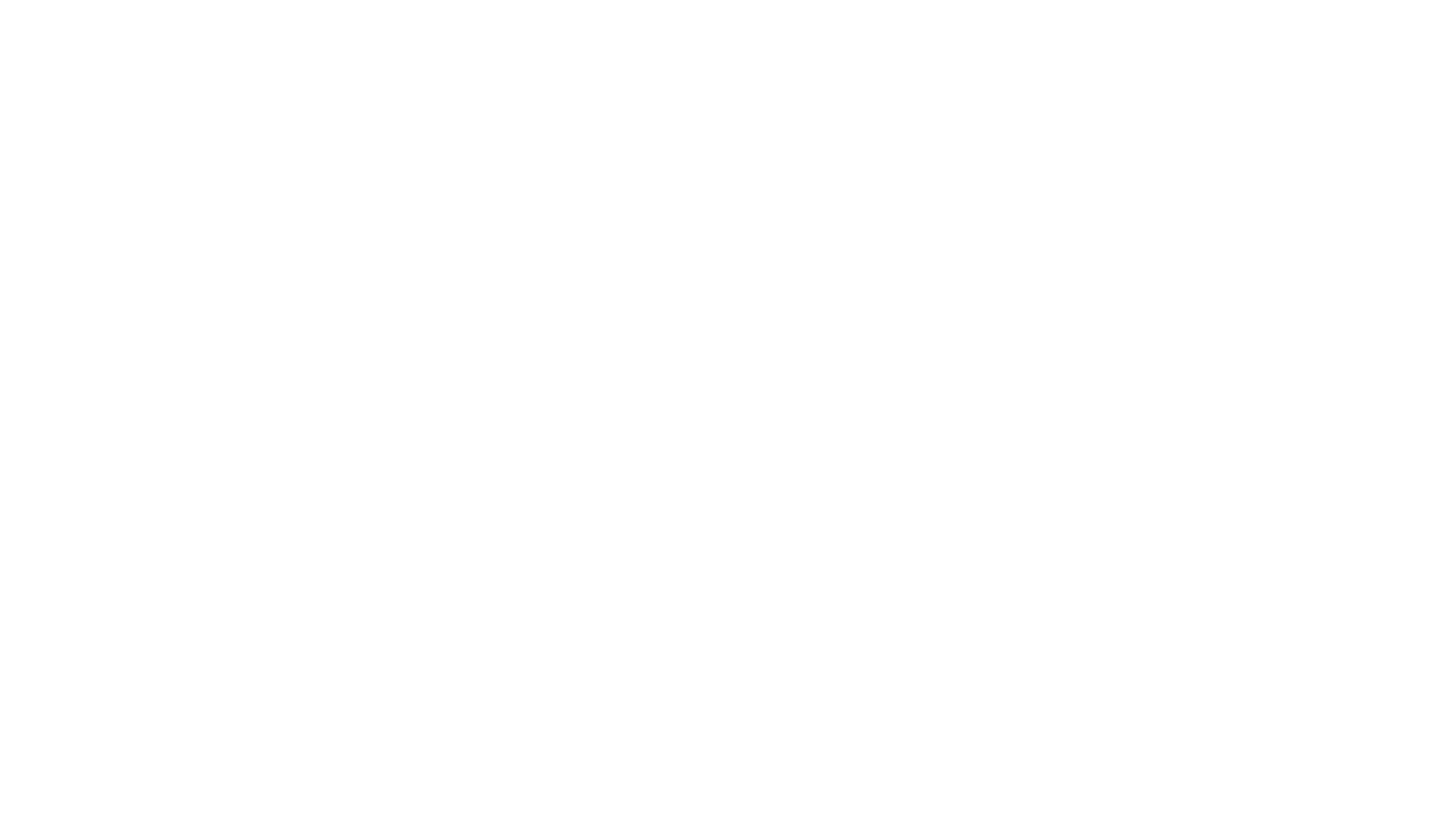

## Slide 170
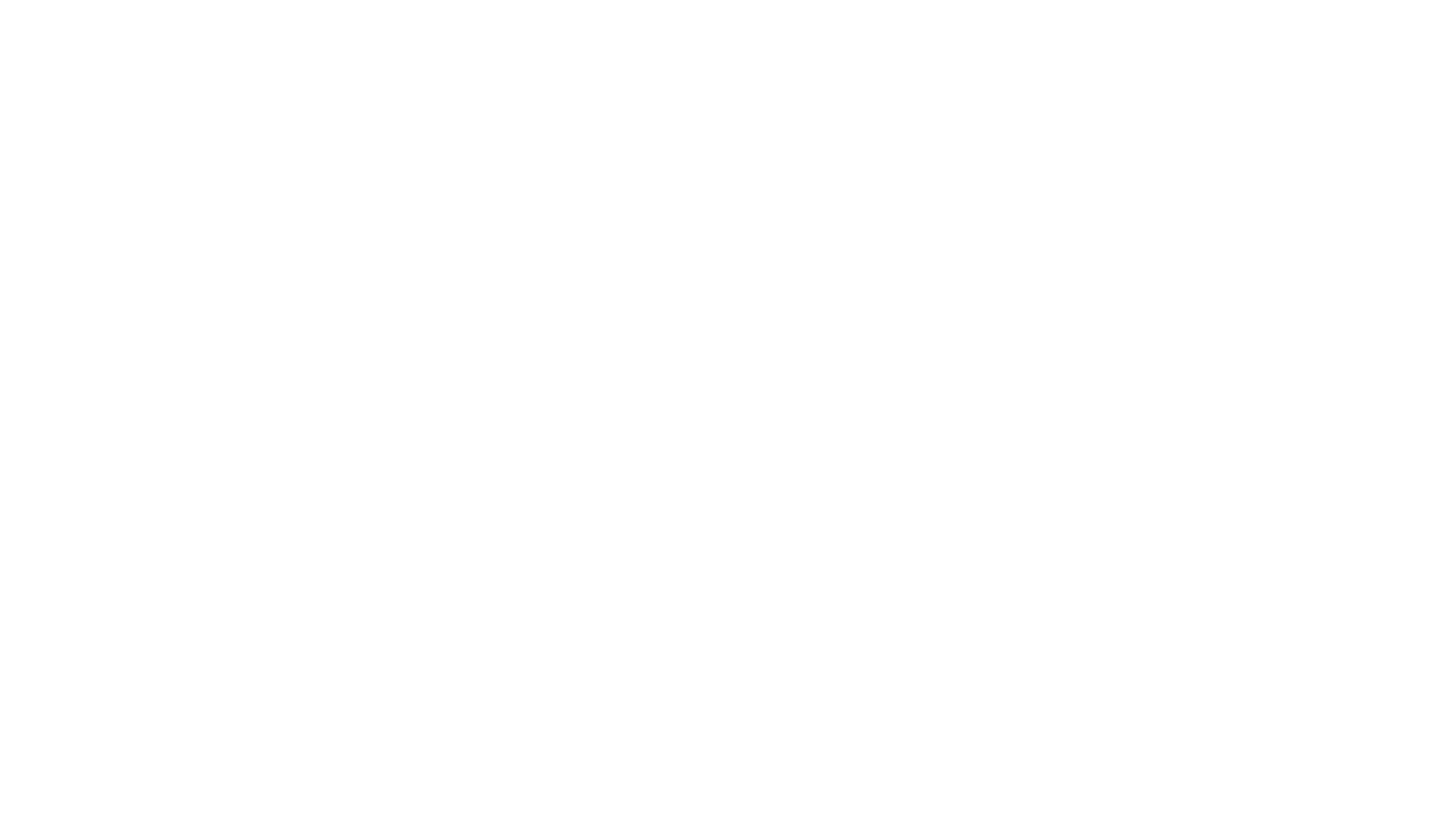

## Slide 171
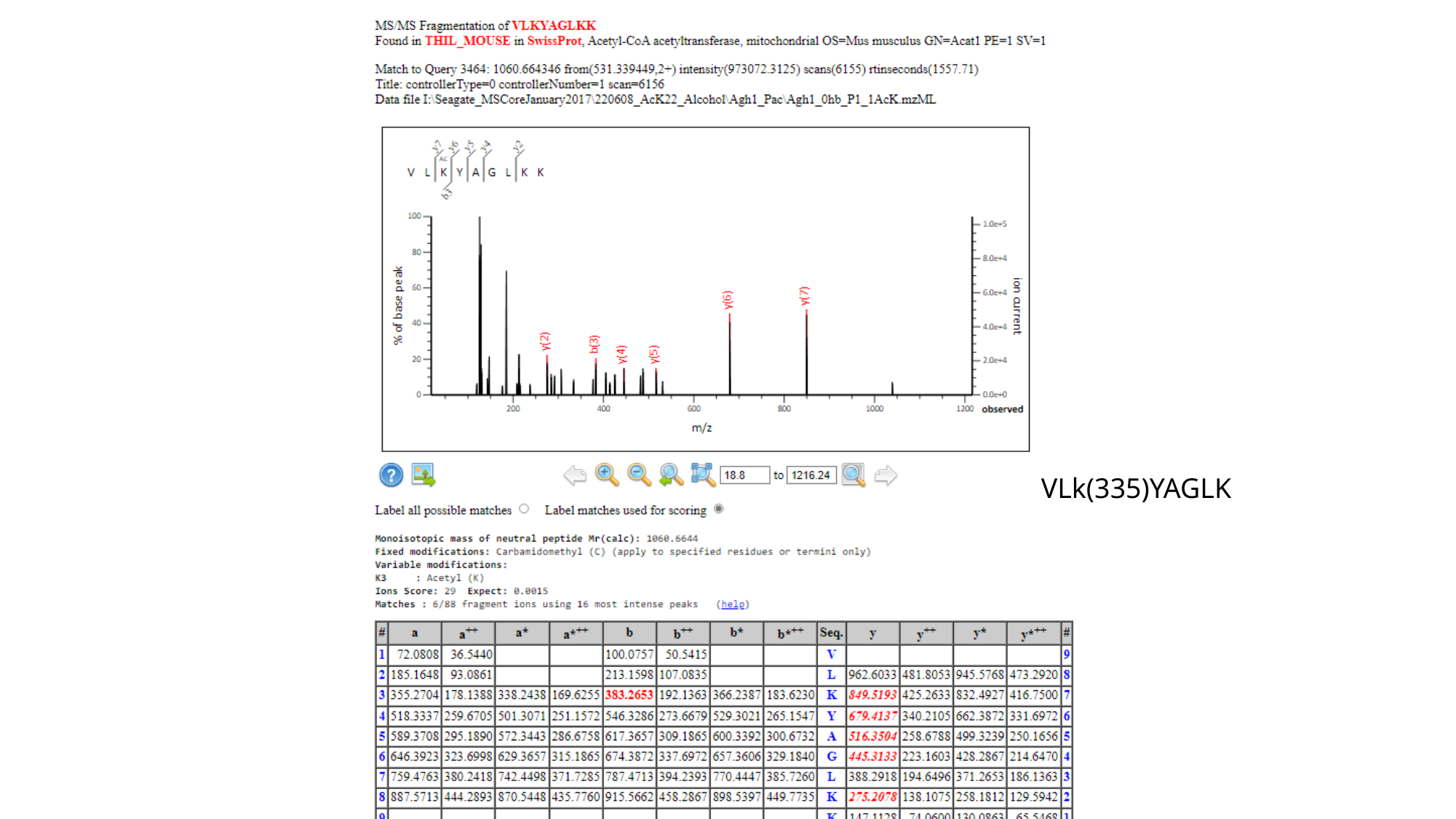

VLk(335)YAGLK
