# Supplementary material for: Chronic Alcohol Consumption Reprograms Hepatic Metabolism Through Organelle-Specific Acetylation in Mice
Source: Mol Cell Proteomics. 2025 May 12;24(6):100990. doi: 10.1016/j.mcpro.2025.100990 (PMC12289531; doi:10.1016/j.mcpro.2025.100990)
Supplement: Supplemental Figures [file mmc6.pptx]

## Slide 1
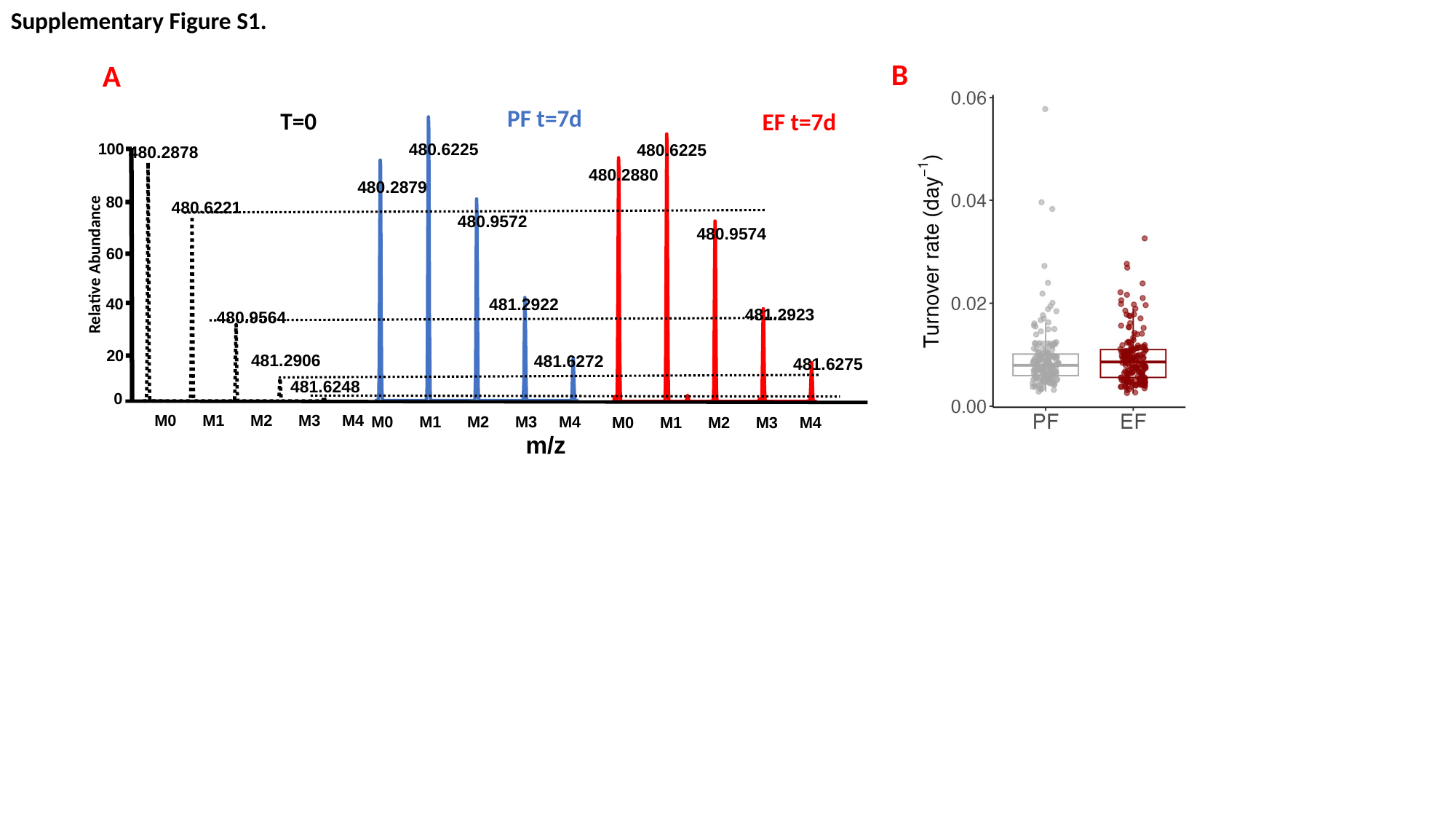

Supplementary Figure S1.
B
A
PF t=7d
T=0
EF t=7d
480.6225
480.2879
480.9572
481.2922
481.6272
480.6225
480.2880
480.9574
481.2923
481.6275
100
80
Relative Abundance
60
40
20
0
M0 M1 M2 M3 M4
m/z
480.2878
480.6221
480.9564
481.2906
481.6248
M0 M1 M2 M3 M4
M0 M1 M2 M3 M4

## Slide 2
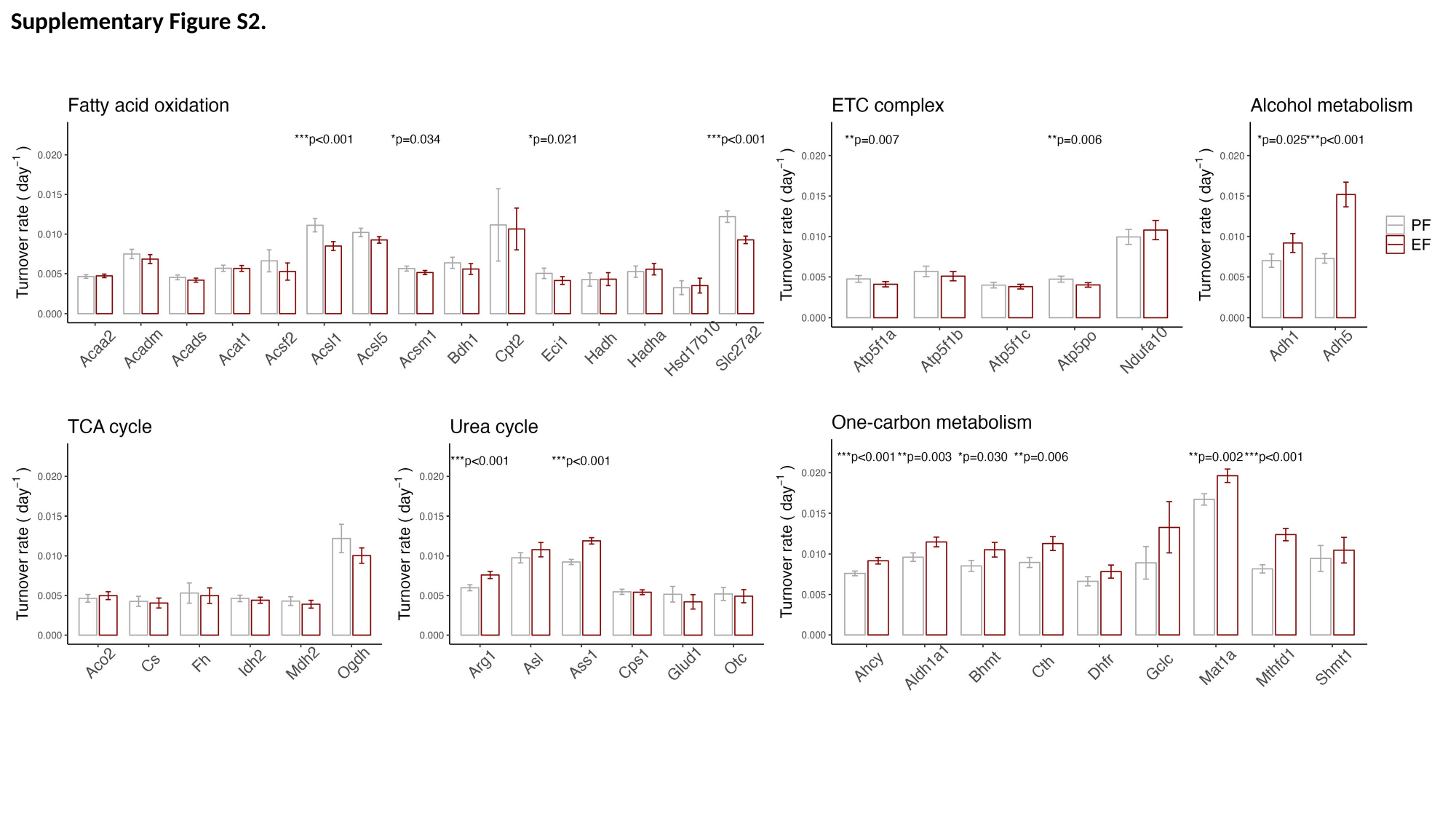

Supplementary Figure S2.

## Slide 3
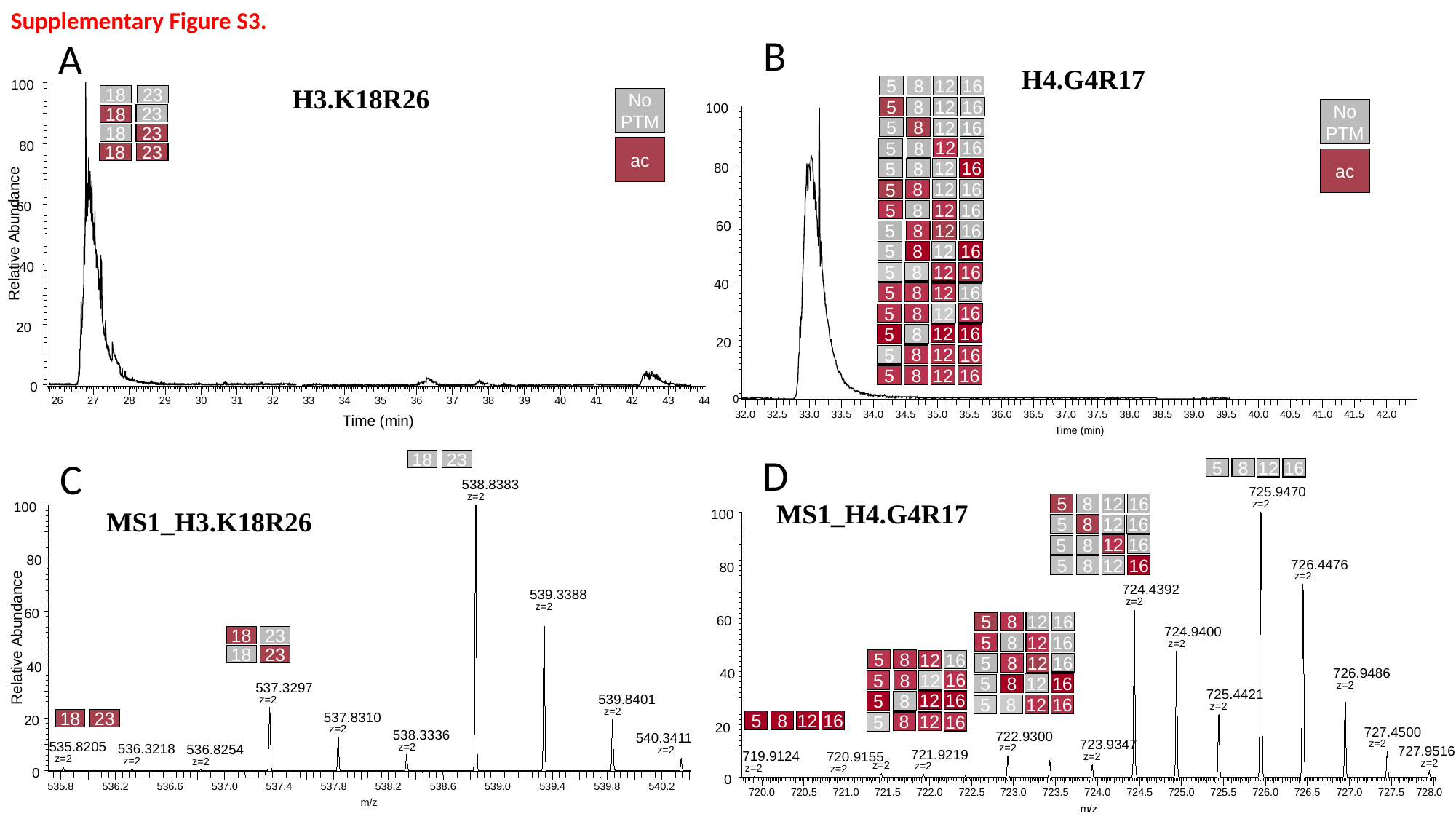

Supplementary Figure S3.
B
A
H4.G4R17
5
8
8
5
8
5
5
8
12
16
12
16
16
12
12
16
12
16
12
16
16
12
16
12
5
8
8
5
8
5
5
8
12
16
12
16
16
12
16
12
5
8
8
5
8
5
5
8
12
16
12
16
16
12
5
8
8
5
8
5
100
80
60
40
20
0
32.0
32.5
33.0
33.5
34.0
34.5
35.0
35.5
36.0
36.5
37.0
37.5
38.0
38.5
39.0
39.5
40.0
40.5
41.0
41.5
42.0
Time (min)
725.9470
z=2
100
0
720.0
720.5
721.0
721.5
722.0
722.5
723.0
723.5
724.0
724.5
725.0
725.5
726.0
726.5
727.0
727.5
728.0
m/z
726.4476
80
z=2
724.4392
z=2
60
724.9400
z=2
726.9486
40
z=2
725.4421
z=2
20
727.4500
722.9300
723.9347
z=2
z=2
727.9516
721.9219
719.9124
720.9155
z=2
z=2
z=2
z=2
z=2
z=2
5
8
12
16
12
16
16
12
12
16
12
16
8
5
8
5
5
8
5
8
12
16
16
12
16
12
8
5
8
5
5
8
12
16
12
16
5
8
8
5
8
5
5
8
16
12
16
12
12
16
12
16
5
8
8
5
5
8
12
16
100
26
27
28
29
30
31
32
33
34
35
36
37
38
39
40
41
42
43
44
Time (min)
80
60
Relative Abundance
40
20
0
538.8383
z=2
100
80
539.3388
z=2
60
Relative Abundance
40
537.3297
539.8401
z=2
z=2
537.8310
20
z=2
538.3336
540.3411
535.8205
536.3218
z=2
536.8254
z=2
z=2
z=2
z=2
0
535.8
536.2
536.6
537.0
537.4
537.8
538.2
538.6
539.0
539.4
539.8
540.2
m/z
H3.K18R26
18
23
23
18
23
18
18
23
No
PTM
ac
No
PTM
ac
D
C
18
23
MS1_H4.G4R17
MS1_H3.K18R26
23
18
23
18
18
23

## Slide 4
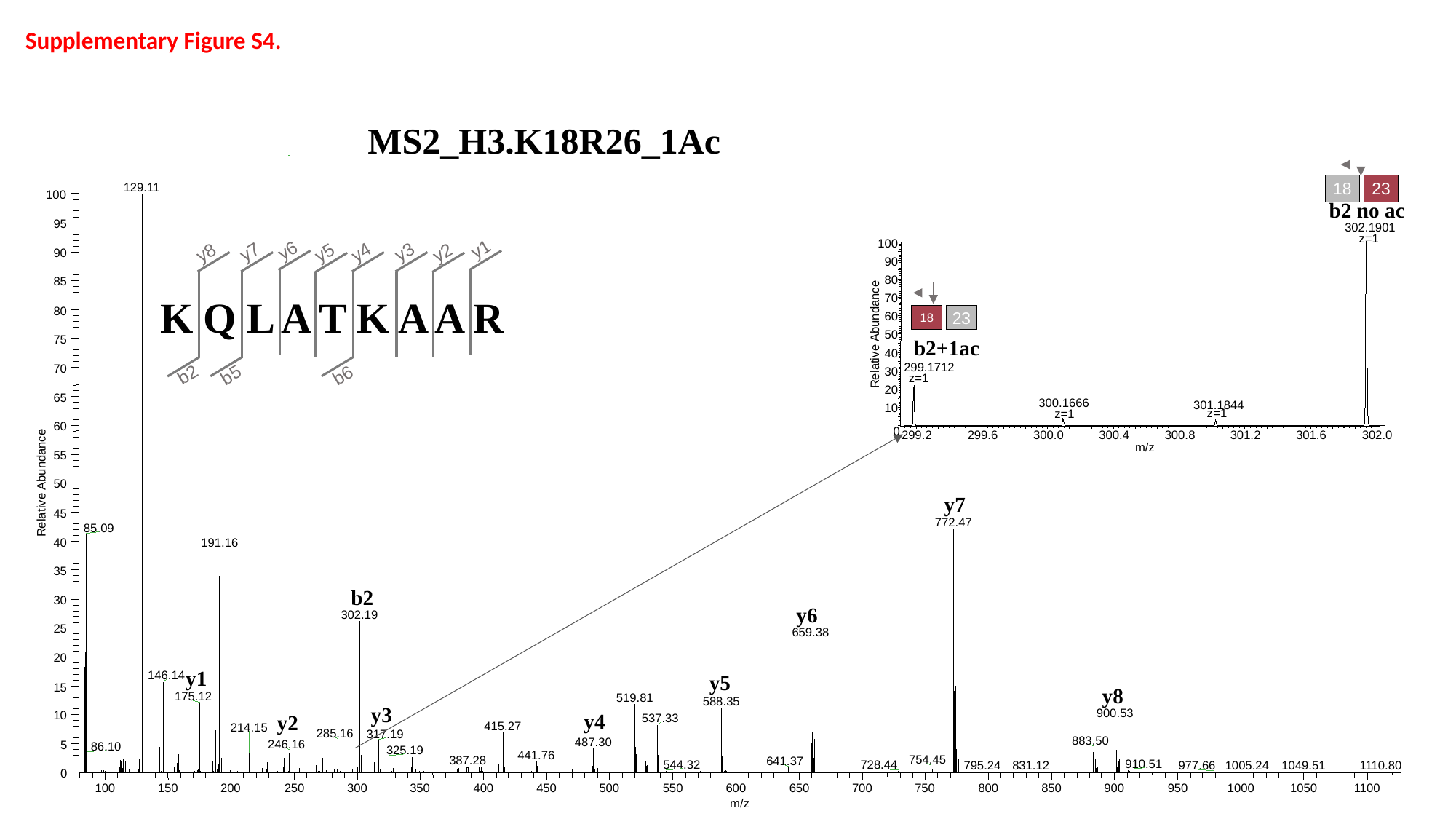

Supplementary Figure S4.
MS2_H3.K18R26_1Ac
100
150
200
250
300
350
400
450
500
550
600
650
700
750
800
850
900
950
1000
1050
1100
m/z
129.11
100
95
90
85
80
75
70
65
60
55
Relative Abundance
50
45
772.47
85.09
40
191.16
35
30
302.19
25
659.38
20
146.14
15
175.12
519.81
588.35
900.53
10
537.33
415.27
214.15
285.16
317.19
883.50
487.30
246.16
5
86.10
325.19
441.76
754.45
387.28
641.37
910.51
544.32
728.44
795.24
831.12
977.66
1005.24
1049.51
1110.80
0
23
18
b2 no ac
302.1901
z=1
100
90
80
70
60
50
Relative Abundance
40
299.1712
30
z=1
20
300.1666
301.1844
10
z=1
z=1
0
299.2
299.6
300.0
300.4
300.8
301.2
301.6
302.0
m/z
y1
y6
y7
y3
y4
y2
y5
y8
K Q L A T K A A R
b2
b5
b6
18
23
b2+1ac
y7
b2
y6
y1
y5
y8
y3
y4
y2

## Slide 5
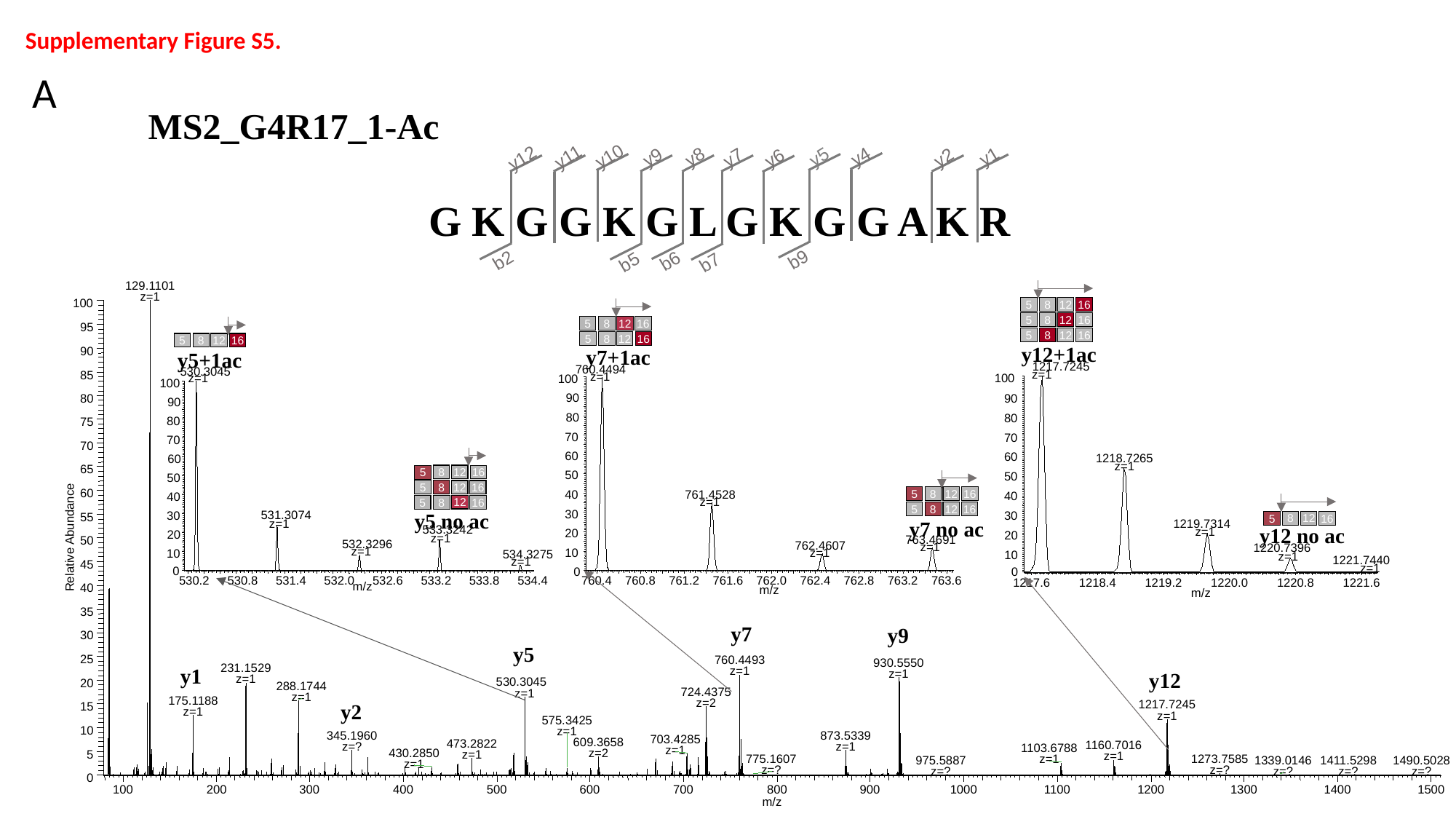

Supplementary Figure S5.
A
MS2_G4R17_1-Ac
y10
y1
y4
y11
y8
y5
y9
y2
y7
y12
y6
G K G G K G L G K G G A K R
b9
b2
b6
b5
b7
129.1101
z=1
100
0
100
200
300
400
500
600
700
800
900
1000
1100
1200
1300
1400
1500
m/z
95
90
85
80
75
70
65
60
55
Relative Abundance
50
45
40
35
30
25
760.4493
930.5550
231.1529
z=1
z=1
z=1
530.3045
20
288.1744
724.4375
z=1
z=1
175.1188
z=2
1217.7245
15
z=1
z=1
575.3425
10
z=1
345.1960
873.5339
703.4285
609.3658
473.2822
1160.7016
z=?
z=1
1103.6788
z=1
430.2850
z=2
5
z=1
z=1
775.1607
1273.7585
z=1
975.5887
1339.0146
1411.5298
1490.5028
z=1
z=?
z=?
z=?
z=?
z=?
z=?
5
8
12
16
5
8
12
16
5
8
12
16
y12+1ac
1217.7245
z=1
100
90
80
70
60
1218.7265
z=1
50
40
30
1219.7314
z=1
20
1220.7396
10
z=1
1221.7440
z=1
0
1217.6
1218.4
1219.2
1220.0
1220.8
1221.6
m/z
12
16
8
5
y12 no ac
12
16
12
16
5
8
5
8
y7+1ac
5
8
12
16
y5+1ac
760.4494
z=1
100
760.4
760.8
761.2
761.6
762.0
762.4
762.8
763.2
763.6
m/z
90
80
70
60
50
761.4528
40
z=1
30
20
763.4691
762.4607
z=1
10
z=1
0
530.3045
z=1
100
90
80
70
60
50
40
531.3074
30
z=1
533.3242
20
10
0
530.2
530.8
531.4
532.0
532.6
533.2
533.8
534.4
m/z
z=1
532.3296
z=1
534.3275
z=1
12
16
16
12
12
16
8
5
8
5
5
8
y5 no ac
12
16
16
12
8
5
8
5
y7 no ac
y7
y9
y5
y1
y12
y2

## Slide 6
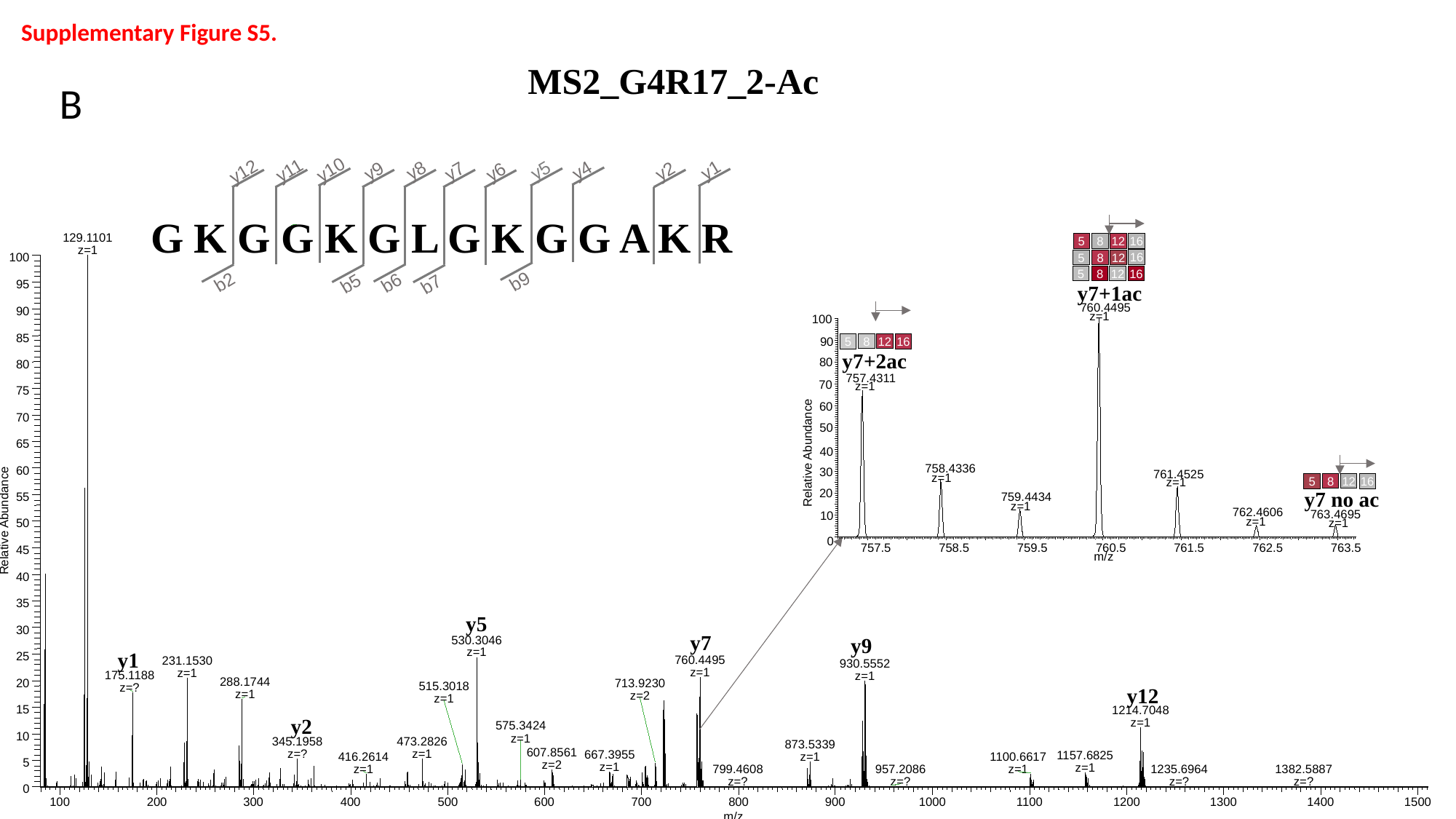

Supplementary Figure S5.
MS2_G4R17_2-Ac
B
y10
y1
y4
y11
y8
y5
y9
y2
y7
y12
y6
G K G G K G L G K G G A K R
b9
b2
b6
b5
b7
129.1101
z=1
100
0
100
200
300
400
500
600
700
800
900
1000
1100
1200
1300
1400
1500
m/z
95
90
85
80
75
70
65
60
55
Relative Abundance
50
45
40
35
30
530.3046
z=1
25
760.4495
231.1530
930.5552
z=1
z=1
175.1188
z=1
288.1744
20
713.9230
515.3018
z=?
z=1
z=2
z=1
15
1214.7048
z=1
575.3424
10
z=1
345.1958
473.2826
873.5339
607.8561
z=?
z=1
667.3955
1157.6825
416.2614
z=1
1100.6617
5
z=2
z=1
z=1
z=1
799.4608
957.2086
z=1
1235.6964
1382.5887
z=?
z=?
z=?
z=?
y7+1ac
760.4495
z=1
100
757.5
758.5
759.5
760.5
761.5
762.5
763.5
m/z
90
80
757.4311
70
z=1
60
50
40
Relative Abundance
758.4336
30
761.4525
z=1
z=1
20
759.4434
z=1
762.4606
763.4695
10
z=1
z=1
0
y7+2ac
y7 no ac
y5
y7
y9
y1
y12
y2
8
5
5
8
16
12
16
12
12
16
5
8
12
16
8
5
12
8
16
5

## Slide 7
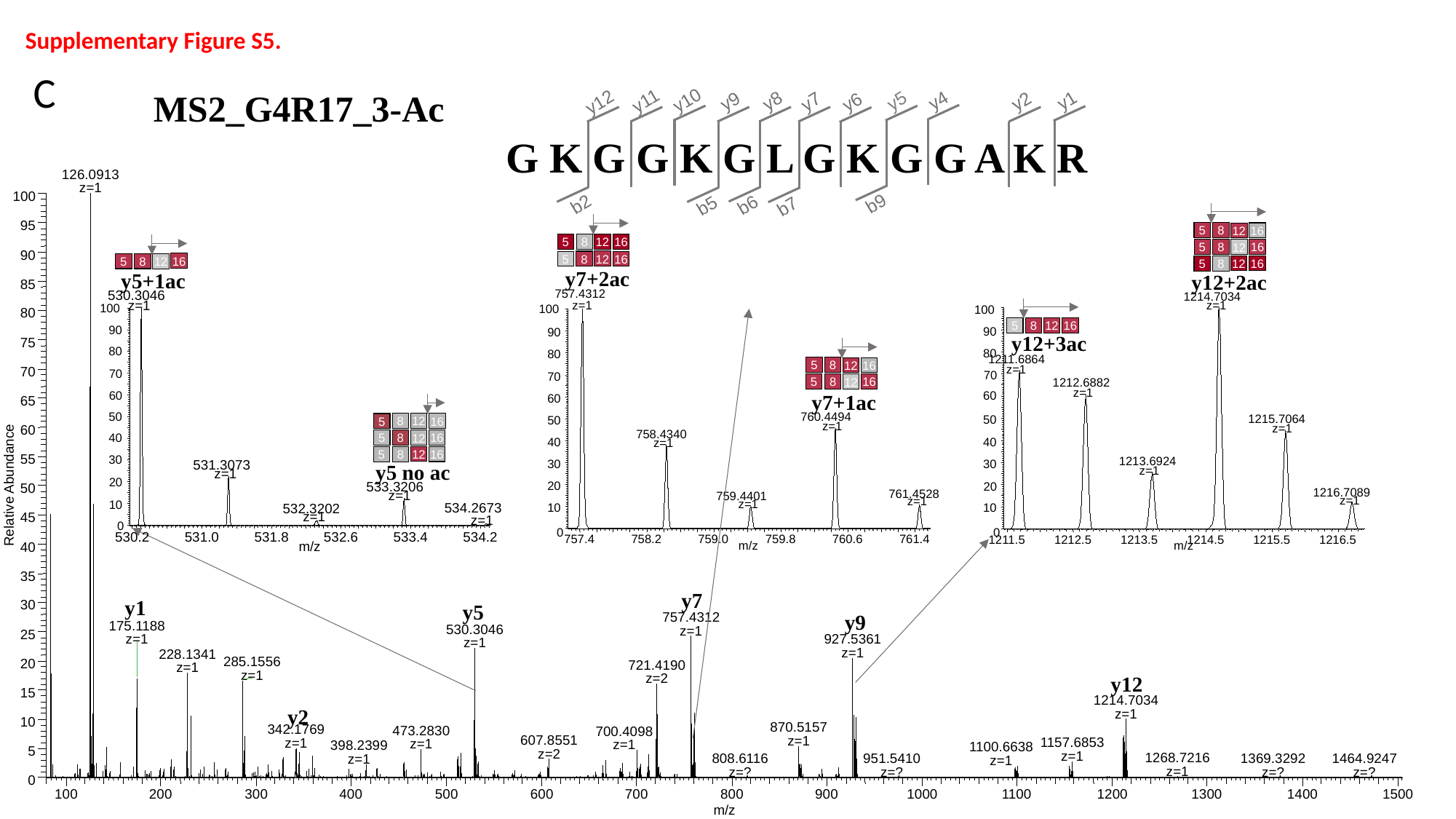

Supplementary Figure S5.
C
MS2_G4R17_3-Ac
y10
y1
y4
y11
y8
y5
y9
y2
y7
y12
y6
G K G G K G L G K G G A K R
b9
b2
b6
b5
b7
126.0913
z=1
100
5
0
100
200
300
400
500
600
700
800
900
1000
1100
1200
1300
1400
1500
m/z
95
90
85
80
75
70
65
60
55
Relative Abundance
50
45
40
35
30
757.4312
175.1188
530.3046
z=1
25
z=1
927.5361
z=1
z=1
228.1341
285.1556
20
721.4190
z=1
z=1
z=2
15
1214.7034
z=1
10
870.5157
342.1769
473.2830
700.4098
607.8551
z=1
1157.6853
z=1
z=1
z=1
398.2399
1100.6638
z=2
z=1
1268.7216
808.6116
951.5410
1369.3292
1464.9247
z=1
z=1
z=1
z=?
z=?
z=?
z=?
8
5
5
8
16
12
16
12
12
16
5
8
y12+2ac
1214.7034
z=1
100
90
80
1211.6864
z=1
70
1212.6882
z=1
60
1215.7064
50
z=1
40
1213.6924
30
z=1
20
1216.7089
z=1
10
0
1211.5
1212.5
1213.5
1214.5
1215.5
1216.5
m/z
12
16
8
5
y12+3ac
12
16
12
16
5
8
8
5
y7+2ac
757.4312
757.4
758.2
759.0
759.8
760.6
761.4
m/z
z=1
100
90
80
70
60
760.4494
50
z=1
758.4340
40
z=1
30
20
761.4528
759.4401
z=1
z=1
10
0
8
5
5
8
16
12
16
12
y7+1ac
16
12
5
8
y5+1ac
530.3046
z=1
100
90
80
70
60
50
40
30
531.3073
z=1
20
533.3206
z=1
10
534.2673
532.3202
z=1
z=1
0
530.2
531.0
531.8
532.6
533.4
534.2
m/z
12
16
16
12
12
16
8
5
8
5
5
8
y5 no ac
y7
y1
y5
y9
y12
y2

## Slide 8
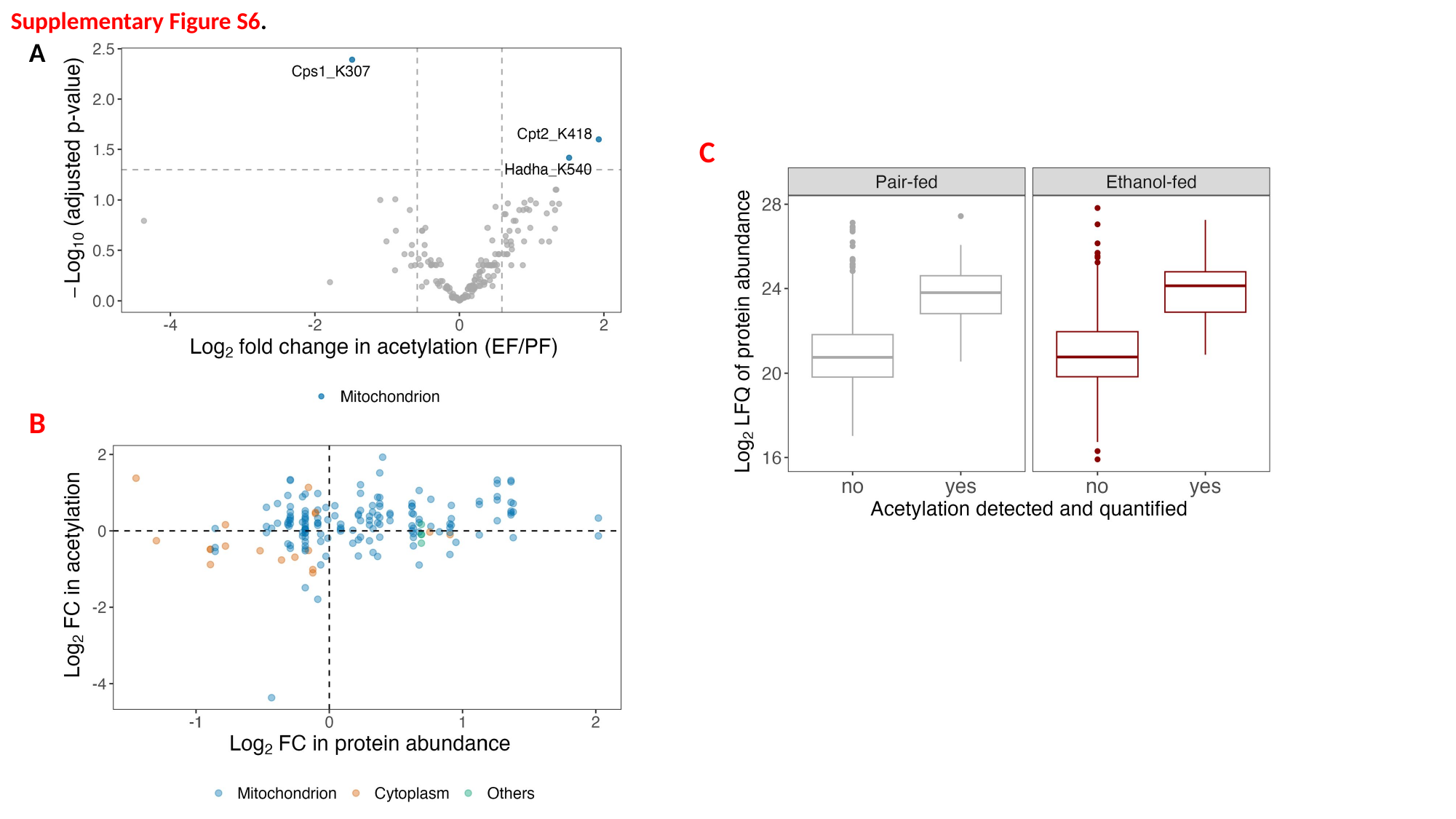

Supplementary Figure S6.
A
C
B

## Slide 9
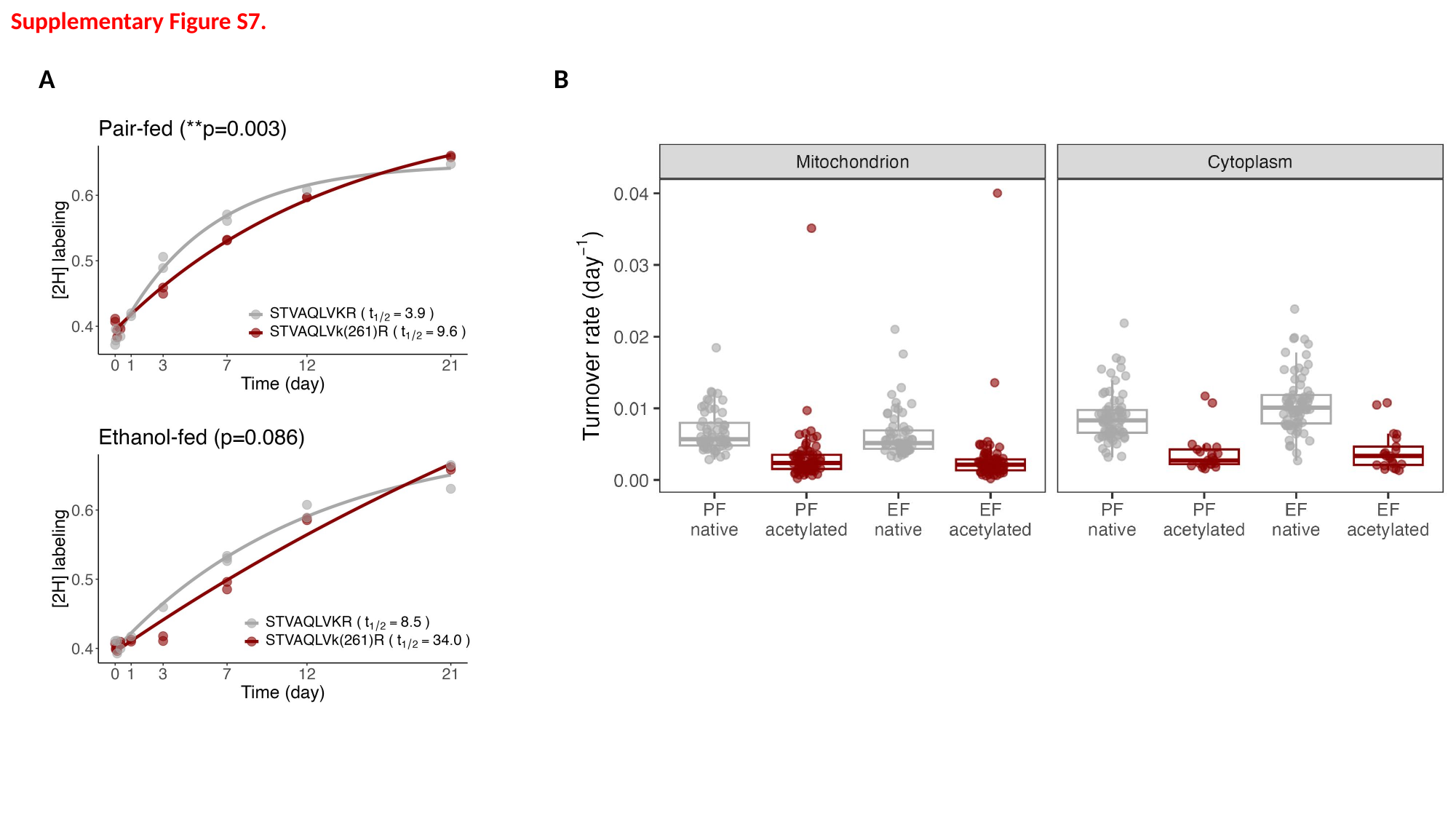

Supplementary Figure S7.
A
B

## Slide 10
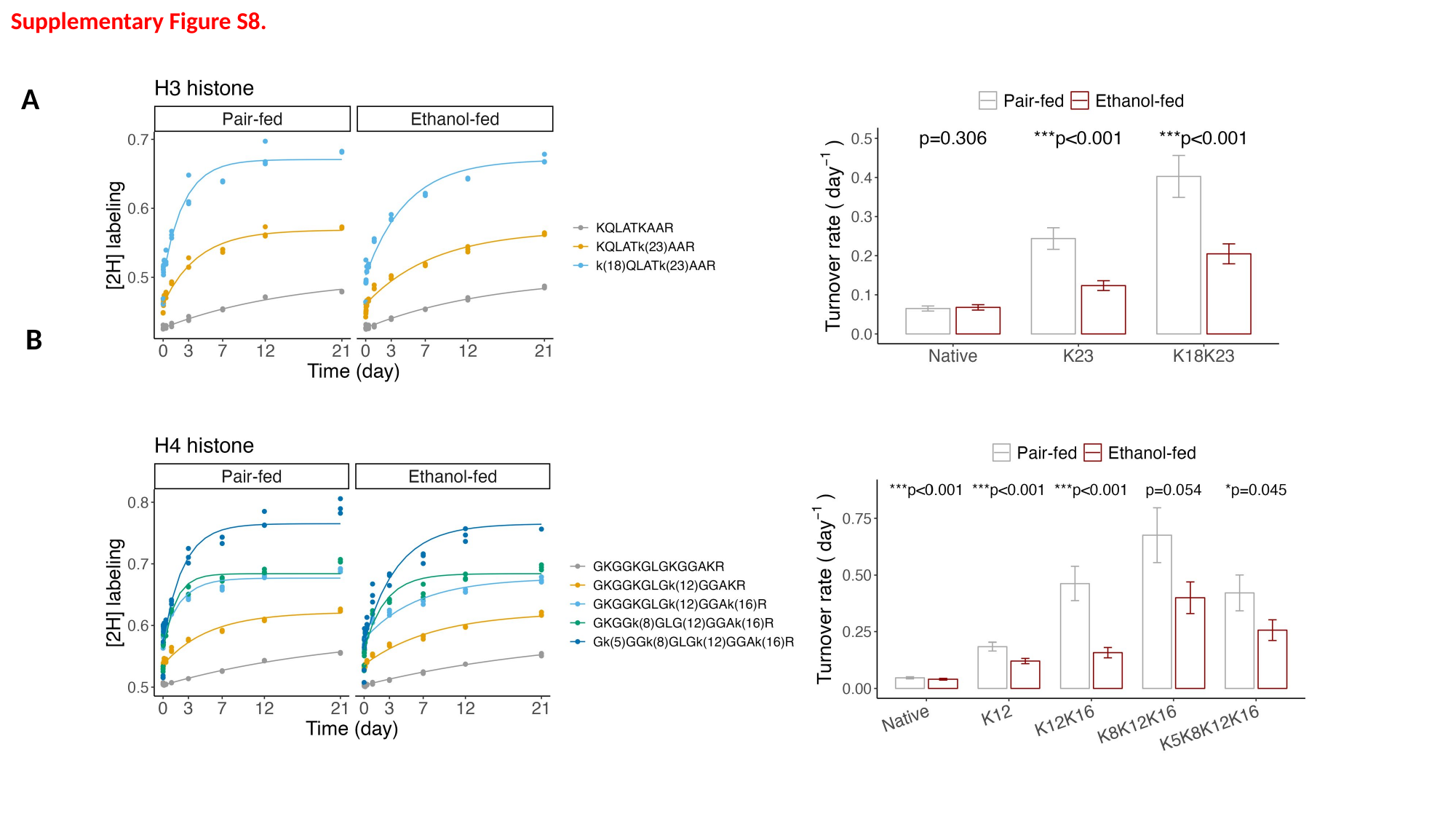

Supplementary Figure S8.
A
B

## Slide 11
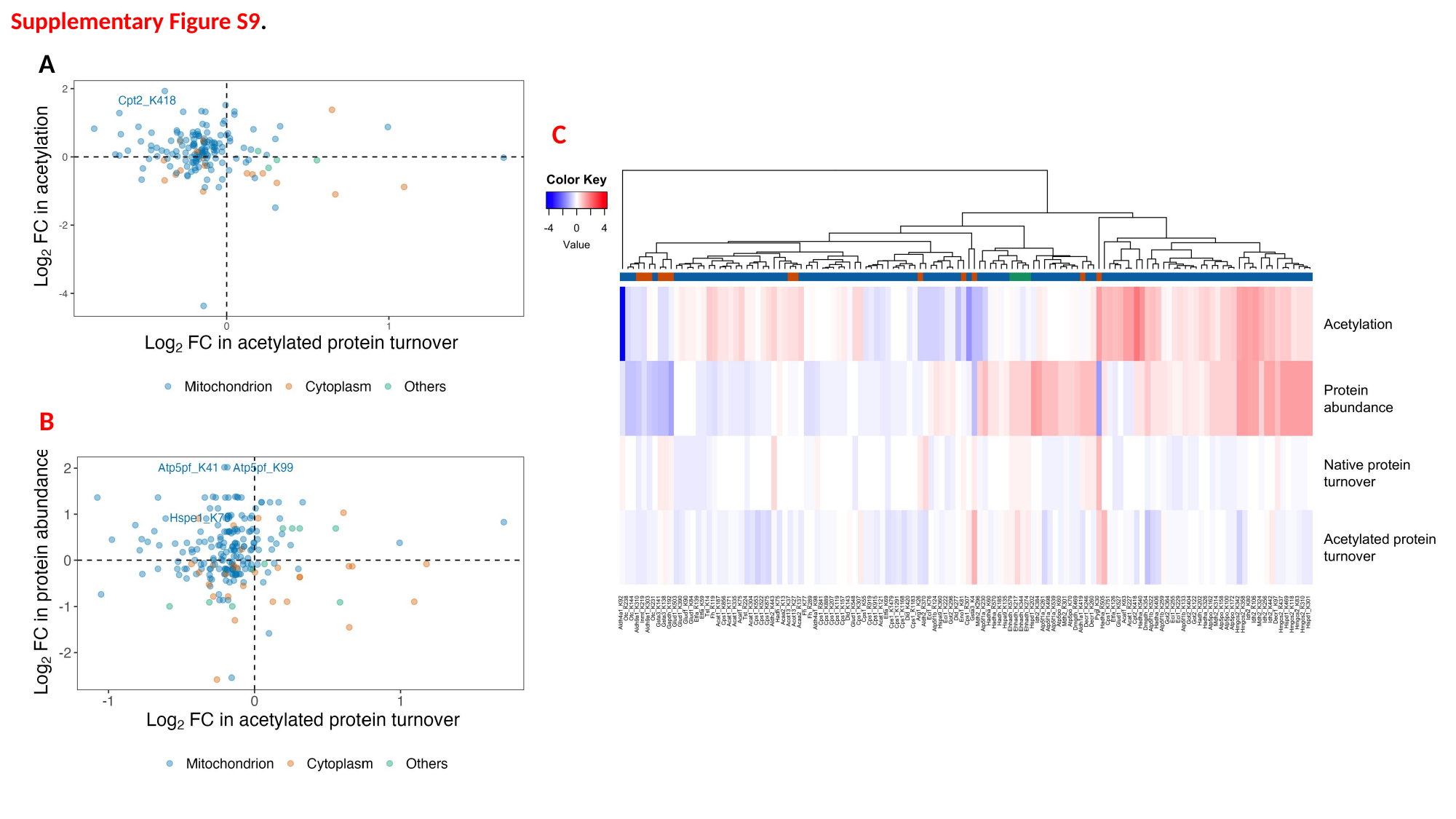

Supplementary Figure S9.
A
C
B

## Slide 12
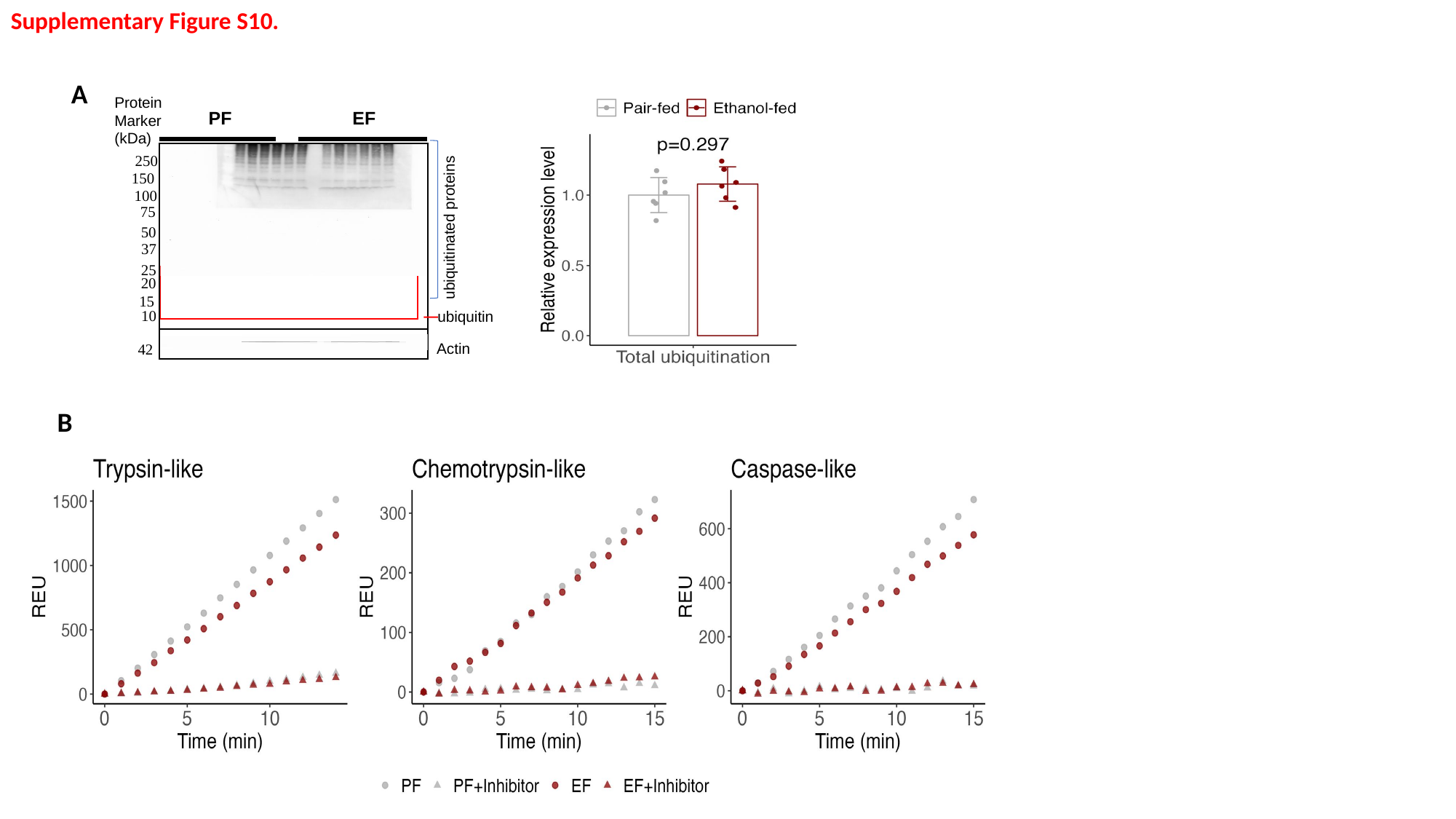

Supplementary Figure S10.
A
Protein
Marker (kDa)
PF
EF
250
150
100
75
50
37
25
20
15
10
ubiquitinated proteins
ubiquitin
Actin
42
B

## Slide 13
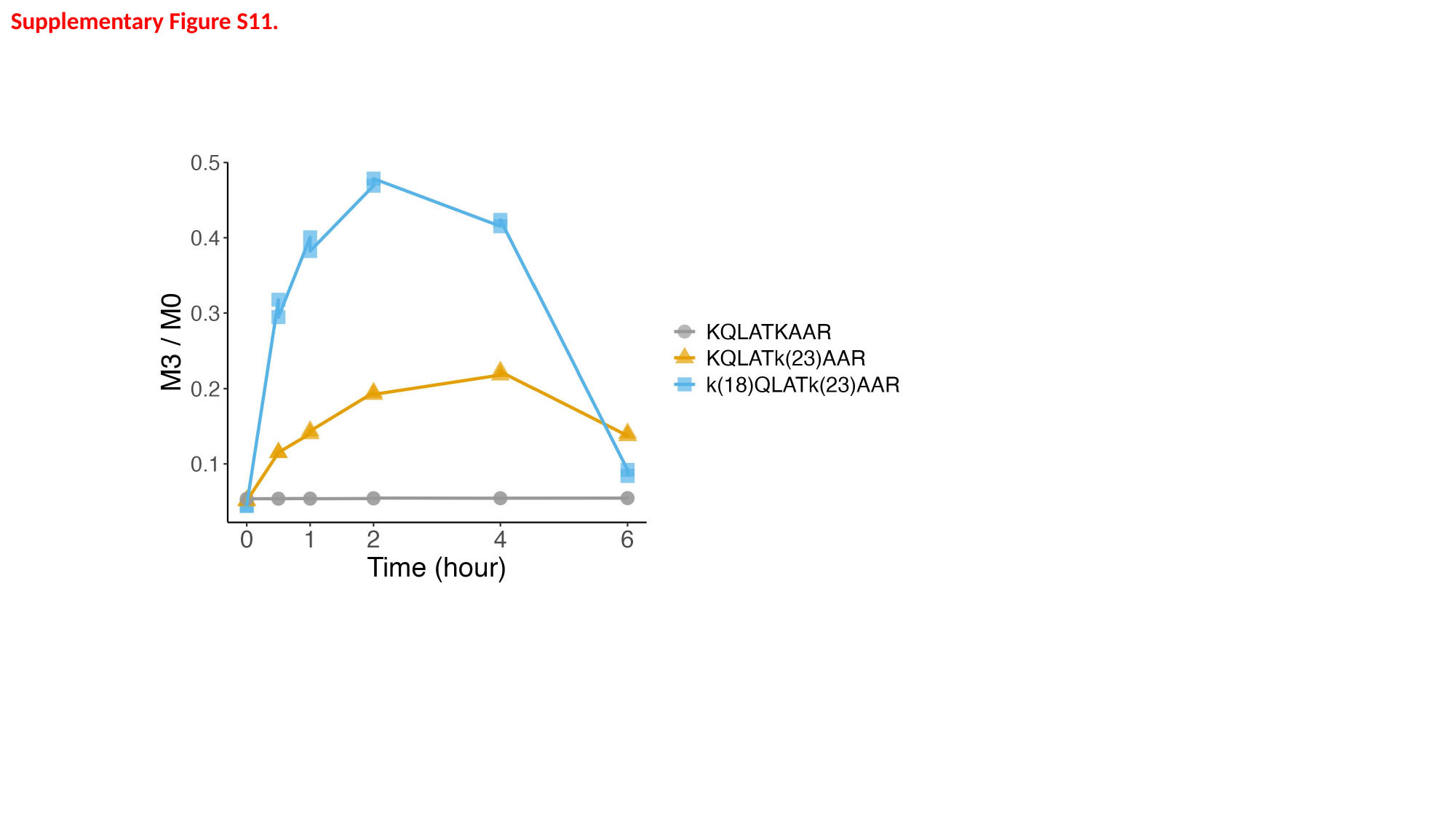

Supplementary Figure S11.

## Slide 14
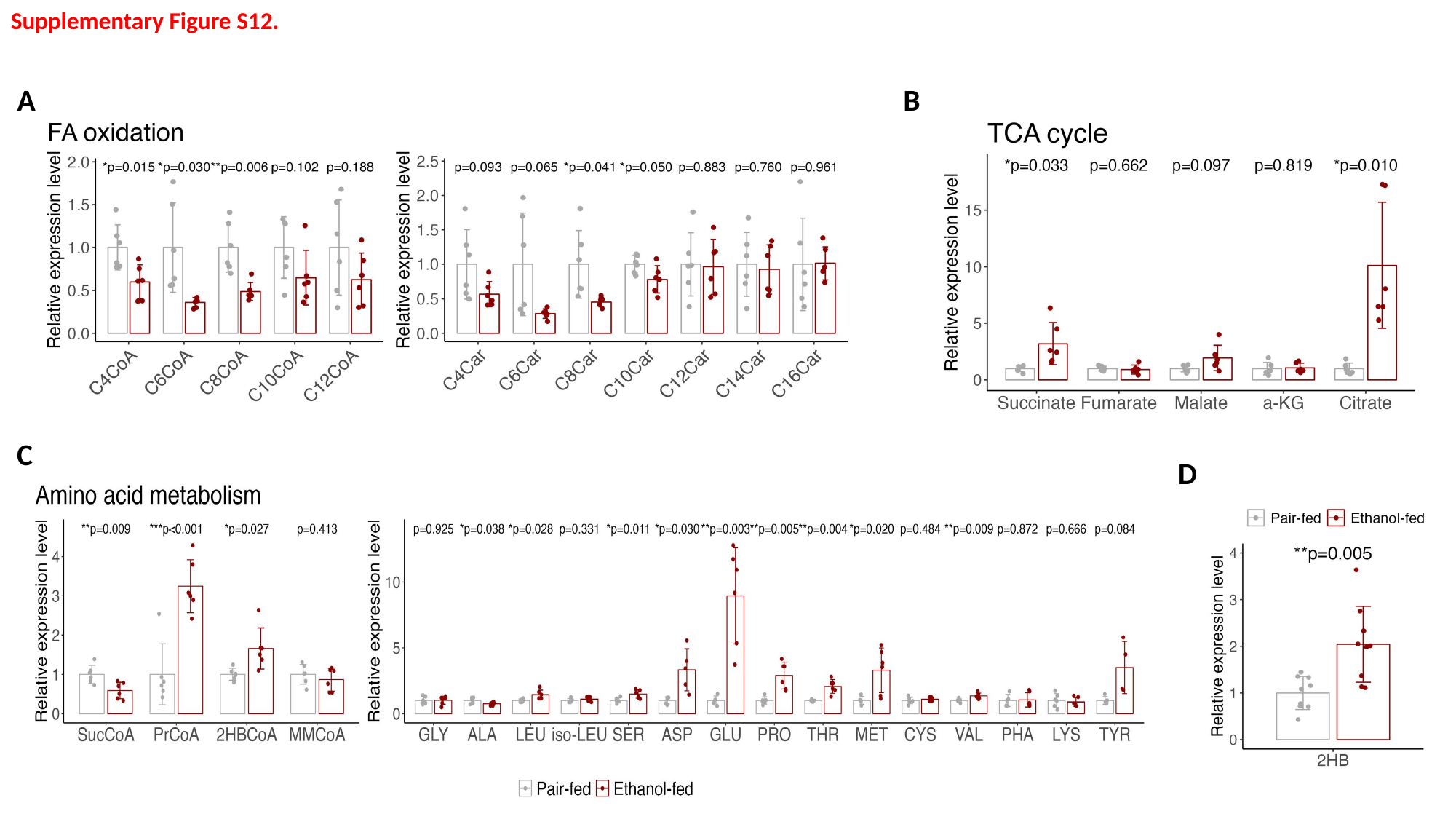

Supplementary Figure S12.
A
B
C
D
