# Supplementary material for: Chronic Alcohol Consumption Reprograms Hepatic Metabolism Through Organelle-Specific Acetylation in Mice
Source: Mol Cell Proteomics. 2025 May 12;24(6):100990. doi: 10.1016/j.mcpro.2025.100990 (PMC12289531; doi:10.1016/j.mcpro.2025.100990)
Supplement: Supplemental Material [file mmc8.docx]

**Chronic alcohol consumption reprograms hepatic metabolism through organelle-specific acetylation in mice.**

Mirjavid Aghayev^1^, Megan R. McMullen^2^, Sergei Ilchenko^1^, Andrea Arias-Alvarado^1^, Victor Lufi^1^, Jack Mathis^1^, Hannah Marchuk^3^, Tsung-Heng Tsai^4^, Guo-Fang Zhang^3^, Laura E. Nagy^2^, and Takhar Kasumov^1^

^1^Department of Pharmaceutical Sciences, College of Pharmacy, Northeast Ohio Medical University, Rootstown, OH 44272

^2^Division of Division of Endocrinology, Metabolism and Nutrition, Duke Molecular Physiology Institute, and Department of Medicine, Duke University, Durham NC 27701

^3^Departments of Inflammation and Immunity and Gastroenterology/Hepatology, Northern Ohio Alcohol Center, The Cleveland Clinic Foundation, Cleveland, OH 44195

^4^Department of Mathematical Sciences, Kent State University, Kent, OH 44242

**Supplementary Methods**

**Materials**

Male and female C57BL/6 wild type mice (18-20 g) were purchased from Jackson Labs. The Lieber-DeCarli ethanol diet and maltose dextrins were from BioServ (Frenchtown, NJ). HPLC grade solvents for nanoflow chromatography and sample preparation were purchased from Fluka (Milwaukee, MO). All other chemicals were from Sigma-Aldrich (St. Louis, MO). ^2^H_2_O (99.9 atom percent excess) and ethanol-d6 (99% percent ^2^H-isotopic and chemical purity) were purchased from Cambridge Isotope Laboratories (Cambridge, MA).

**Animals:**

***Chronic alcohol exposure and ^2^H_2_O-metabolic labeling***: Weight-matched 8 weeks old female mice were randomized into control pair-fed (**PF**) and ethanol-fed (**EF**) groups (n=9/group) and fed their respective liquid diets for 25 days^1^. In the **EF** group, ethanol levels gradually increased: 1% v/v (5.5% kcal in the diet) for 2 days, 2% (11% kcal) for 2 days, 4% (22% kcal) for 1 week, 5% (27% kcal) for 1 week, and 6% (32% kcal) for the remaining final week. In the PF group, maltose-dextrin replaced ethanol iso-calorically. To assess proteome and acetylome dynamics, ²H₂O treatment (25 μl IP bolus/g body weight, followed by a 6% ²H₂O liquid diet) started at intervals during ethanol feeding (0, 1h, 3h, 8h, 1d, 3d, 7d, 12d, and 21d before euthanasia). Early frequent and late infrequent sample collections captured turnover rates of metabolites and proteins with shorter and longer half-lives. This design ensured steady-state body water labeling (~3%) and equal ethanol exposure duration for all mice. Mice were not fasted, and euthanasia occurred between 12-2 PM to avoid circadian variability in acetylation^2^. After pentobarbital anesthesia, blood was collected by cardiac puncture and serum was saved. A portion of liver tissue was saved in 10% formalin for histology^3,4^, and the remaining liver tissue was freeze clamped and stored at -80ºC for future analyses.

***Tracing alcohol metabolites and acetylation with ethanol-d6***: To assess the impact of ethanol-derived acetate on liver protein acetylation and lipid biosynthesis, we administered ethanol-d6 (2.0 µl/g body weight) or saline intraperitoneally to non-fasted male mice. This dose of ethanol induces a twofold increase in blood acetate (~0.4 mM) within 30 minutes^5^. Serum and freeze-clamped tissue samples were collected at baseline, 0.5, 1, 2, 4, and 6 hours post-injection after pentobarbital anesthesia and stored at -80ºC. We measured ²H-labeling of acetate in serum, acetyl-CoA, and acetyl-carnitine in liver tissue to trace ethanol-d6 metabolism. Liver samples were also evaluated for ²H-acetate incorporation into histones, palmitate, and cholesterol. Isotopically labeled metabolites and acetylated histones were quantified using quantitative liquid chromatography-tandem mass spectrometry (LC-MS/MS) and gas chromatography-mass spectrometry (GC-MS) methods.

***Triglycerides analysis*:** Serum levels of total triglycerides (TG) were determined using the TG assay kit (Pointe Scientific, Canton, MI) using 5 µl of serum incubated with TG reagent (1:100 ratio) for 5 minutes. TG-derived glycerol was quantified after a series of enzymatic reactions by the production of a colored product at 500 nm.

***Hepatic oxidative stress*:** Lipid peroxidation products, thiobarbituric acid reactive substances (TBARS) including malonyl dialdehyde (MDA), were quantified as a marker of hepatic oxidative stress using a commercially available assay kit by Cayman Chemical Co (Ann Arbor, MI)). After incubating liver homogenate (10 mg liver in 100 µl of RIPA buffer) with 100 µl of 10% tricholoroacetic acid and 800 µl of a color reagent (37 mM thiobarbituric acid solubilized in 3.5 M acetic acid and 0.7 M sodium hydroxide) at 100°C for 1 hour, the formed malondialdehyde-thiobarbituric acid (MDA-TBA) adduct was measured calorimetrically at 535 nm using spectrophotometer.

***Protease activity assay:*** Hepatic protease activity in the liver homogenate was measured using fluorometric assay kit (UBPBio, Aurora, CO) according to the manufacturer’s protocol. Fluorescence-labelled peptide substrates, succinyl-LLVY-7-amido-4-methylcoumatin (AMC), Boc-LRR-AMC, and Z-LLE-AMC with and without MG132, a proteasome-specific inhibitor, were used for chymotrypsin-, trypsin- and caspase-like activity assays, respectively^6^. To verify the proteasome activity, MG132, a proteasome-specific inhibitor, was separately added to the assay solution at the final concentration of 100 µM for each protease assay. The released fluorophore product AMC was monitored for 15 min at 37°C (Ex/Em=360/460 nm). Proteasome activity was calculated as the difference between the total activity in liver homogenate and the residual activity in the presence of the proteasome inhibitor MG132.

***Total body water enrichment measurement*:** ^2^H-enrichment of body water was measured in serum using a modification of the acetone exchange method. Serum (5 μl), in parallel with a set of calibration curve samples containing 0-5% ^2^H_2_O, was incubated with 10 M potassium hydroxide (5 μl) and pure acetone (5 μl) at room temperature for 4 h. Acetone vapor from the headspace was directly injected for gas chromatography-mass spectrometry (GC-MS) analysis. Isotopic enrichment of acetone was determined using electron impact ionization and selected ion monitoring at *m/z* 58 (M_0_), 59 (M_1_) and 60 (M_2_). The regression equation of the calibration curve was used for ^2^H_2_O enrichment measurement in the biological sample.

***Citric acid cycle intermediates and 2-hydroxybutyrate***: Approximately 30 mg of tissue was spiked with mixed stable isotope-labeled metabolites as internal standards ([U-^13^C_4_]fumarate, ([1,4-^13^C_2_]succinate, ([U-^13^C_4_]malate, and ([1,5-^13^C_2_]citrate) and extracted using the Folch method with 500 μl methanol, 500 μl H₂O, and 500 μl chloroform. After centrifugation for 10 minutes, the upper phase (350 μl) was transferred to a fresh vial and evaporated under nitrogen gas. The dried residues were sequentially derivatized with 40 μl methoxylamine hydrochloride (2% w/v in pyridine) for 90 minutes at 40°C, and then with 60 μl TBDMS (1% tert-butylchlorodimethylsilane) for 1 hour at 80°C. The derivatized samples were centrifuged for 10 minutes at 12,000 × g, and the supernatants were transferred to GC vials for analysis using an Agilent 7890B GC system and 5977A mass spectrometer. A 1 μl aliquot was injected, with the GC temperature gradient starting at 80°C for 2 minutes, increasing at 7°C per minute to 280°C, and held at 280°C for a total run time of 40 minutes. Ionization was performed via electron impact (EI) at 70 eV, with helium flow at 1.1 ml/min. The source, MS quadrupole, interface, and inlet temperatures were maintained at 230°C, 150°C, 280°C, and 250°C, respectively. Analytes were recorded in selected ion monitoring (SIM) mode as we described^7^.

***Amino acids***: Relative levels of hepatic amino acids were analyzed as described^8^. Briefly, 30–50 mg liver tissue samples were spiked with 50 nmol of [¹³C₆]leucine and homogenized in 1 ml of 6% formic acid. After centrifugation, the supernatants were diluted with water and loaded on an anion-exchange column (AG 50W-X8 resin, hydrogen form). The column was washed with water (5 ml), and amino acids were eluted with 2 ml of 4 N ammonium hydroxide. The eluent was dried and derivatized with 60 µl of bis(trimethylsilyl)trifluoroacetamide + 10% trimethylchlorosilane (Pierce). The trimethylsilyl derivatives of amino acids were analyzed using GC-MS with electron impact ionization (70 eV) and SIM mode^8^.

***Palmitate and cholesterol:*** To quantify hepatic lipogenesis, liver samples (~30-40 mg) and calibration curve samples were spiked with 50 μL of 1 mM [^2^H_6_]-cholesterol and 100 μL of 1mM heptadecanoic acid (C17) solutions, homogenized in 0.5 ml of 1N NaCl and extracted by the Bligh-Dyer method ^9^. After solvent evaporation, lipids were saponified with 1N KOH/70% ethanol for 2 hours at 70 ºC, then evaporated to dryness and suspended in 150 μl of 1N HCl. Total lipids were extracted with 1.0 ml of pentane, evaporated, and derivatized with 65 μl of bis(trimethylsilyl) trifluoroacetamide + 1% trimethylchlorosilane at 70 ºC for 45 minutes. The ²H enrichment and concentrations of cholesterol and palmitate were determined using an Agilent GC-MS system. Cholesterol was analyzed in electron impact ionization mode (70 eV) with SIM of m/z 368-371 (M0-M3, endogenous cholesterol) and 374 (M6, [²H₆]-cholesterol internal standard). Palmitate was analyzed with SIM of m/z 313-316 (M0-M3, endogenous palmitate) and 327 (C17 internal standard) as described^3^.

***Acetate and acyl-CoAs analysis by LC-MS/MS:*** Ethanol-derived acetate in plasma was analyzed as described^10^. Briefly, a 30-µl plasma sample was mixed with 30 µl of 200 µM [2,2,2-²H₃-1,2-¹³C₂]acetate (M+5 acetate) internal standard. Acetonitrile (1 ml) was added to precipitate protein, and the supernatant was dried under nitrogen gas. The residue was resuspended in 50 µl HPLC water, 20 µl 3-nitrophenylhydrazine hydrochloride (120 mM), and 20 µl N-(3-dimethylaminopropyl)-N′-ethylcarbodiimide (200 mM) for derivatization at 40 °C for 30 minutes. After centrifugation, the supernatant (3 µl) was analyzed by LC-MS/MS using a Sciex QTRAP 6500+ MS connected with a Sciex AD UHPLC with an Agilent C18 column (Pursuit XRs C18 150 × 2.0 mm, 5 µm). A gradient with two mobile phases (A: 98% H₂O, 2% acetonitrile, 0.1% formic acid; B: 98% acetonitrile, 2% H₂O, 0.1% formic acid) with flow rate of 0.4 ml/min at room temperature was used. The gradient started at 2% B, increased to 90% over 8 minutes, maintained for 4.5 minutes, then returned to initial conditions. M0 acetate, M+3 acetate, and M+5 acetate were analyzed in negative mode using 194/151, 197/153, and 199/155 multiple reaction monitoring (MRM) ion transitions.

Acyl-CoAs and free CoA were analyzed using previously established method^11,12^. Approximately 75 mg of liver tissue was spiked with 0.2 nmol [²H₉]pentanoyl-CoA (internal standard) and homogenized in 1.5 ml extraction buffer (5% acetic acid in 50:50 MeOH/H₂O). The supernatant was processed on a 1 ml ion exchange cartridge with 100 mg of 2-(2-pyridyl)ethyl silica gel (Sigma, MO), pre-activated with methanol and equilibrated with extraction buffer. Acyl-CoAs were eluted with the sequential addition of (i) 1 ml 50 mM ammonium formate (pH 6.3) and methanol (1:1), (ii) 1 ml 50 mM ammonium formate (pH 6.3) and methanol (1:3), and (iii) 1 ml methanol. The combined effluent was dried under N_2_ gas and stored at -80°C until LC-MS/MS analysis using a UHPLC-6500 QTRAP+ mass spectrometer (Sciex) as described ^11,12^.

***Nuclei isolation and histone extraction*:** Histones were isolated from liver tissues by acid extraction as previously described with some modifications ^13,14^. Briefly, tissues tissues were homogenized on ice in Triton Extraction Buffer (TEB: PBS containing 0.5 % Triton X 100 (v/v), protease inhibitors cocktail and 5 mM sodium butyrate) at 100mg /ml using a polytron homogenizer and kept on ice for 5 to 10 minutes to allow cells to lyse and release nuclei. Samples were then centrifuged at 2000 rpm for 10 min at 4°C to precipitate nucleus. Supernatant containing mostly cytoplasm were discarded and the pellet containing cell nuclei were washed in half the volume of TEB and centrifuged as above. Histones were then acid extracted by resuspending nuclei pellets in 200 µl of 0.4 N H_2_SO_4_ and incubated overnight at 4°C^13^. Then centrifuged at 14.000 g for 15 minutes at 4°C and supernatant transferred to a clean tube. Two ml of cold acetone was then added and incubated at −20°C overnight to precipitate histones. Histones were then collected by centrifugation at 14,000 g for 15 min, washed again with acetone then air dried and resuspended in sterile deionized water. Protein concentration was measured using Bradford assay and samples were stored at -20°C until further analysis.

***Preparation of histone samples for proteomics analysis*:** Extracted histones were prepared for proteomic analyses as described^15^, with some modifications. Briefly, histones were first derivatized with acetic anhydride-d_6_ to block unmodified lysines. Briefly, 60 µg of acid extracted histones were dried down in a SpeedVac. Then were dissolved in 50 µL 0.5 M ammonium bicarbonate solution (pH = 8.0). Six µl of acetic anhydride-d_6_ was added to each sample, pH adjusted to ~8 using ammonium hydroxide (12N) and incubated at 4°C for 20 min. To remove solvent, the samples were evaporated for 30 minutes in a SpeedVac. The second round of chemical acetylation were performed for each sample to ensure near complete lysine acetylation. To revert possible O-acetylation dried samples were treated with 50 μl of 50% hydroxylamine and adjusted to pH 8.0 by the addition of NH4OH. The samples were tryptic digested at 37 °C for 4 hour and a second digestion performed by adding glu-C (Promega) and incubating at 37 °C overnight. The final chemical acetylation was performed to modify peptide n-terms. The volume of each sample was adjusted to 20 µL using 5% trifluoroacetic acid and 10 µL aliquot was purified and desalted using a C18 solid phase extraction column. Eluted samples were dried in a SpeedVac and reconstituted in 30 µL 2% acetonitrile and 0.1% formic acid solution and transferred to LC-MS vial for analysis.

***Acetylome sample preparation:*** Acetylated peptides from whole tissue lysates were prepared as described ^16^. Briefly, liver tissue samples (~150 mg) were homogenized in 2 ml of tissue lysis buffer (pH=7.4) for isolation of acetylated proteins ^16^. Two and half-milligram protein from whole tissue homogenate was reduced with 4.5 mM dithiothreitol (DTT) and alkylated with 10 mM of iodoacetamide. Proteins were digested with trypsin (Trypsin: Protein ratio of 1:50) overnight at 37ºC. Peptides were purified using solid-phase extraction C18 columns (Supelco DSC-18). The acetylated peptides were immunoenriched using PTMScan® kit for acetyl-lysine motif [Ac-K] according to the manufacturer’s protocol (#13416, Cell Signaling Technologies, Danvers, MA). Peptides were reconstituted in immunoaffinity purification (IAP) buffer (#993, Cell Signaling) and centrifuged at 14,000x*g* at 4 °C for 10 min to remove cell debris. Antibody beads were washed with PBS and incubated with peptides in IAP buffer at 4°C overnight with gentle rotation. After elution of the non-bound fraction with IAP buffer (2 x 1 mL), the acetylated peptides were eluted with 0.15% trifluoroacetic acid (TFA, 3x200 µL) for 10 min at room temperature with gentle mixing. Both unbound and immuno-isolated fractions containing native and acetylated peptides, respectively were subjected to small scale desalting using C18 ZipTips (Agilent), dried, and analyzed by nanospray LC-MS/MS after reconstitution in 2% acetonitrile and 0.1% formic acid solution.

***Metabolic labeling with ^2^H_2_O:*** Peptide turnover rates, both acetylated and native, were quantified using in vivo ^2^H_2_O-metabolic labeling approach^17^. Specialized software, compatible with Mascot 2.3, quantified ^2^H isotopic enrichments for native peptides^18^. Low-abundance acetylated peptides were analyzed manually using Xcalibur (Thermo). The criteria for kinetic analysis comprised the accuracy of precursor ion mass within 3 ppm, signal intensity > 10^4^, more than 5 MS1 scans, and the absence of overlapping signals in at least 3 mass isotopomers, where isotopomers represent isotope isomers of molecules. Turnover rates were determined through isotopomer distribution analysis of MS1 spectra. With ^2^H_2_O metabolic labeling, during protein synthesis, incorporation of ^2^H labeled amino acids into a peptide chain leads to redistribution of isotope pattern, showing elevated levels of heavy isotopomers (*M_1_*, *M_2_*, *M_3_*, etc.) compared to the *M_0_* light isotopomer.

**Quantification of H3 and H4 histone acetylation stoichiometry**

We chemically acetylated lysines that are not endogenously acylated using heavy acetic anhydride (d3-acetyl). This approach resulted in the co-elution of endogenously acetylated and native peptides during chromatography, complicating their quantification based on MS1 ions. However, this strategy enhances precision by providing similar ionization characteristics and increases confidence in identifying low-abundance acetylation sites through isotopic mass shifts.

Although we were unable to chromatographically separate the endogenously and chemically acetylated isoforms of H3 (18-26) peptides, MS1 spectra allowed for their detection due to differences in mass observed in liver sample histone preparations (Supplementary Figure C&D). The MS2 spectra successfully differentiated between single-acetylated H3K18ac and H3K23ac forms. We used the intensities of b2 ions of the single acetylated precursors to calculate their relative abundance as described^19^:

$$\boldsymbol{H}\boldsymbol{3.K}\boldsymbol{18}\boldsymbol{R}\boldsymbol{26\_noPTM}=\frac{MS1\_K18R26\_noPTM}{\sum(MS1\_K18R26\_noPTM,MS1\_K18R26\_1Ac,MS1\_K18R26\_2Ac)}$$

$$\boldsymbol{H}\boldsymbol{3.K}\boldsymbol{18}\boldsymbol{ac}=\frac{MS1\_K18R26\_1Ac}{\sum(MS1\_K18R26\_noPTM,MS1\_K18R26\_1Ac,MS1\_K18R26\_2Ac)}* \frac{MS1\_K18R26\_b2\_K18ac\_299}{\sum(MS1\_K18R26\_b2\_K18ac\_299 ,MS1\_K18R26\_b2\_K18NoAc\_302 )}$$

$$\boldsymbol{H}\boldsymbol{3.K}\boldsymbol{23}\boldsymbol{ac}=\frac{MS1\_K18R26\_1Ac}{\sum(MS1\_K18R26\_noPTM,MS1\_K18R26\_1Ac,MS1\_K18R26\_2Ac)}* \frac{MS1\_K18R26\_b2\_K18NoAc\_302}{\sum(MS1\_K18R26\_b2\_K18ac\_299 ,MS1\_K18R26\_b2\_K18NoAc\_302 )}$$

$$\boldsymbol{H}\boldsymbol{3.K}\boldsymbol{18}\boldsymbol{acK}\boldsymbol{23}\boldsymbol{ac\_}=\frac{MS1\_K18R26\_2Ac}{\sum(MS1\_K18R26\_noPTM,MS1\_K18R26\_1Ac,MS1\_K18R26\_2Ac)}$$

We also identified MS1 masses corresponding to mono-, di-, tri-, and tetra-acetylated forms of H4 (4-17) peptides. MS2 peptide sequencing enabled the identification and quantification of all positional isomers of these peptides based on their y5, y7, and y12, and corresponding mixed MS1 of mono-, di-, and tri-acetylated forms with the exception of the diacetylated species H4K5acK12ac, H4K5acK16ac, H4K8acK12ac, and H4K8acK16ac, for which we present combined abundance values.

$$\boldsymbol{H}\boldsymbol{4.G}\boldsymbol{4}\boldsymbol{R}\boldsymbol{17\_noPTM}=\frac{MS1\_G4R17\_noPTM}{\sum(MS1\_G4R17\_noPTM,MS1\_G4R17\_1Ac,MS1\_G4R17\_2Ac MS1\_G4R17\_3AcMS1\_G4R17\_4Ac)}$$

$$\boldsymbol{H}\boldsymbol{4.K}\boldsymbol{5}\boldsymbol{ac}=\frac{MS1G4R17\_1Ac}{\sum\left( MS1\_G4R17\_\mathrm{noPTM},MS1\_G4R17\_1Ac,MS1\_G4R17\_2AcMS1\_G4R17\_3AcMS1\_G4R17\_4Ac \right)}*$$

$(1- \frac{MS2\_G4R17\_1Ac\_y12\_K16K12K8.1.Ac\_1217}{\sum(MS2\_G4R17\_1Ac\_y12\_K16K12K8.1Ac\_1217 ,MS2\_G4R17\_1Ac\_y12\_K16K12K8.0Ac\_1220)}$)

$$\boldsymbol{H}\boldsymbol{4.K}\boldsymbol{8}\boldsymbol{ac}=\frac{MS1G4R17\_1Ac}{\sum\left( MS1\_G4R17\_\mathrm{noPTM},MS1\_G4R17\_1Ac,MS1\_G4R17\_2AcMS1\_G4R17\_3AcMS1\_G4R17\_4Ac \right)}$$

$$*( \frac{MS2\_G4R17\_1Ac\_y12\_K16K12K8.1Ac\_1217}{\sum\left( MS2\_G4R17\_1Ac\_y12\_K16K12K8.1Ac\_1217 ,MS2\_G4R17\_1Ac\_y12\_K16K12K8.0Ac\_1220 \right)} -$$

$\frac{MS2\_G4R17\_1Ac\_y7\_K16K12.1Ac\_760}{\sum\left( MS2\_G4R17\_1Ac\_y7\_K16K12.1Ac\_760 ,MS2\_G4R17\_1Ac\_y7\_K16K12.0Ac\_763 \right)}$)

$$\boldsymbol{H}\boldsymbol{4.K}\boldsymbol{12}\boldsymbol{ac}=\frac{MS1G4R17\_1Ac}{\sum\left( MS1\_G4R17\_\mathrm{noPTM},MS1\_G4R17\_1Ac,MS1\_G4R17\_2AcMS1\_G4R17\_3AcMS1\_G4R17\_4Ac \right)}$$

$*(\frac{MS2\_G4R17\_1Ac\_y7\_K16K12.1Ac\_760}{\sum\left( MS2\_G4R17\_1Ac\_y7\_K16K12.1Ac\_760 ,MS2\_G4R17\_1Ac\_y7\_K16K12.0Ac\_763 \right)}$ -

$\frac{MS2\_G4R17\_1Ac\_y5\_K16ac\_530}{\sum\left( MS2\_G4R17\_1Ac\_y5\_K16ac\_530 ,MS2\_G4R17\_1Ac\_y5\_K16NoAc\_533 \right)}$)

$$\boldsymbol{H}\boldsymbol{4.K}\boldsymbol{16}\boldsymbol{ac}=\frac{MS1\_G4R17\_1Ac}{\sum\left( MS1\_G4R17\_\mathrm{noPTM},MS1\_G4R17\_1Ac,MS1\_G4R17\_2AcMS1\_G4R17\_3AcMS1\_G4R17\_4Ac \right)}$$

$$*\frac{MS2\_G4R17\_1Ac\_y5\_K16ac\_530}{\sum\left( MS2\_G4R17\_1Ac\_y5\_K16ac\_530 ,MS2\_G4R17\_1Ac\_y5\_K16NoAc\_533 \right)}$$

$$\boldsymbol{H}\boldsymbol{4.K}\boldsymbol{5}\boldsymbol{acK}\boldsymbol{8}\boldsymbol{ac}=\frac{MS1G4R17\_2Ac}{\sum\left( MS1\_G4R17\_\mathrm{noPTM},MS1\_G4R17\_1Ac,MS1\_G4R17\_2AcMS1\_G4R17\_3AcMS1\_G4R17\_4Ac \right)}*$$

$$\frac{MS2\_G4R17\_1Ac\_y12\_K16K12K8.1.Ac\_1217}{\sum(MS2\_G4R17\_2Ac\_y7\_K16K12.0Ac\_763 ,MS2\_G4R17\_2Ac\_y7\_K16K12.1Ac\_760 ,MS2\_G4R17\_2Ac\_y7\_K16K12.2Ac\_757 )}$$

$$\boldsymbol{H}\boldsymbol{4.K}\boldsymbol{12}\boldsymbol{acK}\boldsymbol{16}\boldsymbol{ac}=\frac{MS1G4R17\_2Ac}{\sum\left( MS1\_G4R17\_\mathrm{noPTM},MS1\_G4R17\_1Ac,MS1\_G4R17\_2AcMS1\_G4R17\_3AcMS1\_G4R17\_4Ac \right)}*$$

$$\frac{MS2\_G4R17\_2Ac\_y7\_K16K12.2Ac\_757}{\sum(MS2\_G4R17\_2Ac\_y7\_K16K12.0Ac\_763 ,MS2\_G4R17\_2Ac\_y7\_K16K12.1Ac\_760 ,MS2\_G4R17\_2Ac\_y7\_K16K12.2Ac\_757 )}$$

$$\boldsymbol{H}\boldsymbol{4.K}\boldsymbol{5}\boldsymbol{acK}\boldsymbol{8}\boldsymbol{acK}\boldsymbol{12}\boldsymbol{ac}=\frac{MS1G4R17\_3Ac}{\sum\left( MS1\_G4R17\_\mathrm{noPTM},MS1\_G4R17\_1Ac,MS1\_G4R17\_2AcMS1\_G4R17\_3AcMS1\_G4R17\_4Ac \right)}*$$

$($ $1-\frac{MS2\_G4R17\_3Ac\_y5\_K16ac\_530}{\sum\left( MS2\_G4R17\_3Ac\_y5\_K16ac\_530 ,MS2\_G4R17\_1Ac\_y5\_K16NoAc\_533 \right)}$)

$$\boldsymbol{H}\boldsymbol{4.K}\boldsymbol{5}\boldsymbol{acK}\boldsymbol{8}\boldsymbol{acK}\boldsymbol{16}\boldsymbol{ac}=\frac{MS1G4R17\_3Ac}{\sum\left( MS1\_G4R17\_\mathrm{noPTM},MS1\_G4R17\_1Ac,MS1\_G4R17\_2AcMS1\_G4R17\_3AcMS1\_G4R17\_4Ac \right)}$$

$*($ $\frac{MS2\_G4R17\_1Ac\_y5\_K16ac\_530}{\sum\left( MS2\_G4R17\_3Ac\_y5\_K16ac\_530 ,MS2\_G4R17\_1Ac\_y5\_K16NoAc\_533 \right)}$-

$\frac{MS2\_G4R17\_3Ac\_y7\_K16K12.2Ac\_757}{\sum\left( MS2\_G4R17\_3Ac\_y7\_K16K12.1Ac\_757 ,MS2\_G4R17\_3Ac\_y7\_K16K12.2Ac\_760 \right)}$)

$$\boldsymbol{H}\boldsymbol{4.K}\boldsymbol{5}\boldsymbol{acK}\boldsymbol{12}\boldsymbol{acK}\boldsymbol{16}\boldsymbol{ac}=\frac{MS1G4R17\_3Ac}{\sum\left( MS1\_G4R17\_\mathrm{noPTM},MS1\_G4R17\_1Ac,MS1\_G4R17\_2AcMS1\_G4R17\_3AcMS1\_G4R17\_4Ac \right)}$$

$*($ $\frac{MS2\_G4R17\_3Ac\_y7\_K16K12.2Ac\_757}{\sum\left( MS2\_G4R17\_3Ac\_y7\_K16K12.1Ac\_757 ,MS2\_G4R17\_3Ac\_y7\_K16K12.2Ac\_760 \right)}$-$\frac{MS2\_G4R17\_3Ac\_y12\_K16K12K8.3Ac\_1211}{\sum\left( MS2\_G4R17\_3Ac\_y12\_K16K12K8.3Ac\_1211 ,MS2\_G4R17\_3Ac\_y12\_K16K12K8.2Ac\_1214 \right)}$

$$\boldsymbol{H}\boldsymbol{4.K}\boldsymbol{5}\boldsymbol{acK}\boldsymbol{12}\boldsymbol{acK}\boldsymbol{16}\boldsymbol{ac}=\frac{MS1G4R17\_3Ac}{\sum\left( MS1\_G4R17\_\mathrm{noPTM},MS1\_G4R17\_1Ac,MS1\_G4R17\_2Ac, MS1\_G4R17\_3AcMS1\_G4R17\_4Ac \right)}$$

*$\frac{MS2\_G4R17\_3Ac\_y12\_K16K12K8.3Ac\_1211}{\sum\left( MS2\_G4R17\_3Ac\_y12\_K16K12K8.3Ac\_1211 ,MS2\_G4R17\_3Ac\_y12\_K16K12K8.2Ac\_1214 \right)}$

$$\boldsymbol{H}\boldsymbol{4.K}\boldsymbol{5}\boldsymbol{acK}\boldsymbol{8}\boldsymbol{acK}\boldsymbol{12}\boldsymbol{acK}\boldsymbol{16}\boldsymbol{ac}=\frac{MS1G4R17\_4Ac}{\sum\left( MS1\_G4R17\_\mathrm{noPTM},MS1\_G4R17\_1Ac,MS1\_G4R17\_2AcMS1\_G4R17\_3AcMS1\_G4R17\_4Ac \right)}$$

**Statistical analysis of kinetic data**

The kinetic data were used to evaluate (i) the effect of alcohol on the turnover rate of the native proteins, and (ii) the effect of alcohol on the turnover rate of an acetylated peptide at the specific lysine site. The statistical model and analysis procedure to infer and test the turnover rate are detailed below. The analysis procedure implemented as R script can be found at https://github.com/tsunghengtsai/ald-turnover.

***Analysis and test for alcohol-related change in turnover rate of native protein*:** The turnover rates of native peptides are viewed as repeated measures of the turnover rate of their corresponding protein. Thus, the time-course data of native peptides corresponding to the same protein were modeled jointly to estimate the turnover rate of the native protein. For a particular protein, the total labeling of Peptide $j$ in Replicate $i$ at time $t$ in the PF group is expressed as

$E_{\text{PF}, ij}\left( t \right)=E_{j}^{\left( 0 \right)}+\left( E_{j}^{\left( ss \right)}-E_{j}^{\left( 0 \right)} \right)\times\left[ 1-\exp\left( -k_{\text{PF}}\cdot t \right) \right]+\varepsilon_{ij}(t)$,

where $E_{j}^{\left( 0 \right)}$ and $E_{j}^{\left( ss \right)}$ represent the baseline and plateau enrichments, respectively, for Peptide $j$, $k_{\text{PF}}$ is the turnover rate of the protein in the PF group, and $\varepsilon_{ij}\left( t \right)$ expresses the random variation unexplained by the nonlinear model, with a constant variance $\sigma^{2}$. Similarly, the total labeling of Peptide $j$ in Replicate $i'$ at time $t$ in the EF group is given by

$E_{\text{EF}, i'j}\left( t \right)=E_{j}^{\left( 0 \right)}+\left( E_{j}^{\left( ss \right)}-E_{j}^{\left( 0 \right)} \right)\times\left[ 1-\exp\left( -k_{\text{EF}}\cdot t \right) \right]+\varepsilon_{i'j}(t)$,

where $k_{\text{EF}}$ is the turnover rate of the protein in the EF group and $\varepsilon_{i'j}(t)$ shares the same variance $\sigma^{2}$ as $\varepsilon_{ij}\left( t \right)$. The nonlinear regression model was fit jointly to all labeling measurements for all peptides pertaining to the same protein in both conditions using the nls() R function^20^, where the joint model contains four parameters, ${\beta_{\text{full}}=(E}_{j}^{\left( 0 \right)}, E_{j}^{\left( ss \right)}, k_{\text{PF}}, k_{\text{EF}})$. The fitting function nls() determines the point estimates and standard errors for all the model parameters. Specifically, the parameters are estimated by minimizing the residual sum of squares using the Gaussian-Newton algorithm^20,21^. Once the parameter estimates are obtained, the variance estimate is estimated as the minimized residual sum of squares divided by the degrees of freedom, the number of data points minus the number of model parameters ($p=4$),

$s^{2}=\frac{{RSS}_{\text{full}}(\hat{\beta}_{\text{full}})}{n-p}$.

The standard errors of the parameter estimates are then estimated as the square roots of the diagonal elements in the estimated variance-covariance matrix

$$\hat{\text{Var}}\left( \hat{\beta}_{\text{full}} \right)=s^{2}\hat{B},$$

where $\hat{B}$ is the inverse of the second derivative of the log-likelihood with respect to the parameter $\beta_{\text{full}}$, evaluated at the parameter estimates $\hat{\beta}_{\text{full}}$ ^21^. For each protein, the inferential procedure gives one (and only one) turnover rate estimate in each condition, and the alcohol-related change in turnover rate is estimated as ${\hat{k}_{\text{EF}}-\hat{k}}_{\text{PF}}.$ To assess the significance of the turnover rate change, an *F*-test was performed to test the null hypothesis $H_{0}:k_{\text{PF}}=k_{\text{EF}}$ versus the alternative hypothesis $H_{1}:k_{\text{PF}}\neq k_{\text{EF}}$. The full model with separate turnover rates for both groups as described above was compared to a reduced model under the null hypothesis, with an identical turnover rate shared by both groups (i.e., $k_{\text{PF}}=k_{\text{EF}}=k_{\text{shared}}$). The reduced model has three parameters ${\beta_{\text{reduced}}=(E}_{j}^{\left( 0 \right)}, E_{j}^{\left( ss \right)}, k_{\text{shared}})$ and is contained in the full model. That is, the reduced model represents a submodel of the full model, when $k_{\text{PF}}=k_{\text{EF}}$. The two models are compared using the *F*-test to test the equality of turnover rates in both groups, with the test statistic:

$F=\frac{\left( {RSS}_{\text{reduced}}\left( \hat{\beta}_{\text{reduced}} \right)-{RSS}_{\text{full}}\left( \hat{\beta}_{\text{full}} \right) \right)/\left( {df}_{\text{reduced}}-{df}_{\text{full}} \right)}{{{RSS}_{\text{full}}\left( \hat{\beta}_{\text{full}} \right)}/{{df}_{\text{full}}}}$,

where ${RSS}_{\text{reduced}}\left( \hat{\beta}_{\text{reduced}} \right)$ and ${RSS}_{\text{full}}\left( \hat{\beta}_{\text{full}} \right)$are the residual sum of squares for the reduced and full models, respectively, and ${df}_{\text{reduced}}$ and ${df}_{\text{full}}$ are the residual degrees of freedom for the reduced and full models, respectively. The test statistic is compared against the *F*-distribution with degrees of freedom ${(df}_{\text{reduced}}-{df}_{\text{full}}=1)$ and ${df}_{\text{full}}$ to compute the *p*-value for the hypothesis test, where the difference in degrees of freedom between the reduced and full models is one because of the identical turnover rate parameter (rather than two individual parameters) shared by both groups.

***Analysis and test for alcohol-related change in turnover rate of acetylated lysine sites*:** For acetylated proteins, we evaluated the ethanol-related change in turnover rate in a site-specific manner. Each acetylated lysine site was analyzed individually and the time-course data of acetylated peptides covering the same site were modeled jointly as in the analysis of native proteins. In contrast to the analysis of native protein, the parameters $k_{\text{PF}}, k_{\text{EF}}$ represent site-level estimates of the turnover rates. The inferential and testing procedure can be applied even when a site is represented by only one acetylated peptide.

**Supplementary Figure Legend**.

**Supplementary Figure S1**. Proteome dynamics with ^2^H_2_O (heavy water) -based metabolic labeling. **A:** Isotope distribution of ATP synthase subunit alpha (ATP5A) peptide GIRPAINVGLSVSR at the baseline (T=0), and in PF (blue) and EF (EF) mice after 7 days of heavy water exposure. The horizontal dashed lines indicates the changes in heavy istopomers due to ^2^H incorporation. EF mice show less ^2^H incorporation than PF mice, reflecting slower turnover rate of ATP5A in the liver due to alcohol exposure. **B:** The average turnover rate of hepatic proteins (n=175) did not change significantly due to alcohol exposure.

**Supplementary Figure S2**. Ethanol (EtOH)-induced changes in hepatic protein turnover. EtOH inhibits turnover rates of mitochondrial enzymes in fatty acid oxidation, the TCA cycle, oxidative phosphorylation, and ureagenesis, while promoting turnover rates of cytosolic enzymes in alcohol, amino acid, and one-carbon metabolism.

**Supplementary Figure S3**. Representative LC-MS chromatograms illustrating the positional acetylated isomers of lysine acetylation sites in H3 and H4 histones. Panels A and B: Chemical acetylation with deuterated acetic anhydride results in the co-elution of all positional isomers. Panel C: The MS1 spectrum distinguishes between native and diacetylated H3 forms, despite the lack of separation between the positional isomers H3K18ac and H3K23ac. Panel D: MS1 signals differentiate native and tetra-acetylated H4 forms; however, mono-, di-, and tri-acetylated H4 forms cluster together, complicating their quantification based on MS1 spectra.

**Supplementary Figure S4.** MS2 spectra of the monoacetylated histone H3 peptide (18KQLATKAAR26), a mixture of K18 and K23, were obtained after chemical acetylation with acetic anhydride-d6. The inset displays magnified b2 fragment ions for endogenously acetylated (m/z 299.1712) and chemically acetylated (m/z 302.1901) forms, which were utilized to calculate the relative abundances of H3K18ac and H3K23ac. The arrows above different positions show the fragment ions corresponding to each mass.

**Supplementary Figure S5**. MS2 spectra of a mixture of mono-, di-, and tri-acetylated histone H4 peptide (4GKGGKGLGKGGAKR17) following chemical acetylation with acetic anhydride-d6, used for calculating different positional isomers. Arrows above each position indicate the fragment ions corresponding to their respective masses.

**A:** Quantification of positional monoacetylated isomers H4K5ac, H4K8ac, H4K12ac, and H4K16ac. The inset displays high-resolution spectra of y5, y7, and y12 for the endogenously monoacetylated forms after chemical acetylation with acetic anhydride-d6. Signals from light (endogenous acetylation) and heavy (chemical acetylation) ions were utilized to calculate the relative abundances of the endogenously monoacetylated positional isomers.

**B:** Quantification of positional diacetylated isomers H4K5acK8ac and H4K12K16ac. The inset presents high-resolution spectra of y7 fragment ions derived from endogenously diacetylated (m/z 757.4311), chemically monoacetylated (m/z 760.4495), and diacetylated (m/z 763.4695) heavy forms. Signals from light (endogenous acetylation) and heavy (chemical acetylation) ions were employed to calculate the relative abundances of the endogenously diacetylated positional isomers.

**C:** Quantification of positional triacetylated isomers H4K5acK8acK12ac and H4K5acK8acK16ac. The insets show high-resolution spectra of y5, y7, and y12 for the endogenously triacetylated forms following chemical acetylation with acetic anhydride-d6. Signals from light (endogenous acetylation) and heavy (chemical acetylation) ions were utilized to calculate the relative abundances of the endogenously triacetylated positional isomers.

**Supplementary Figure S6. A:** Label-free quantification of ethanol-induced changes in hepatic protein acetylation. Chronic ethanol intake significantly altered acetylation at 3 of 172 sites. Although not statistically significant, acetylation increased at 112 sites (on 40 proteins) and decreased at 60 sites (on 38 proteins). **B**: Association between changes in acetylation and protein abundance; each dot represents an acetylated site **C**: Boxplot of average with log_2_ total protein quantification, for proteins with and without acetylation form detected and quantified.

**Supplementary Figure S7.** Effect of EtOH-induced acetylation on protein turnover assessed using the ^2^H_2_O-metabolic labeling technique. **A**: Turnover rates of native and acetylated ATPA in pair-fed and ethanol-fed mice, with time-course 2H incorporation into native (STVAQLVKR) and acetylated (STVAQLVK_ac_R) ATPA peptides to evaluate acetylation impact on ATPA turnover and half-life. **B**: Effect of EtOH-induced acetylation on turnover rates of mitochondrial and cytosolic proteins.

**Supplementary Figure S8**. Impact of ethanol-induced acetylation on the turnover rates of selected histone proteins assessed using the ^2^H_2_O-metabolic labeling technique. **A**: H3, and **B**: H4.

**Supplementary Figure S9**. Association between ethanol-induced changes in acetylation, protein turnover, and abundance. **A**: Changes in acetylated protein turnover correlate with fold changes in acetylation. **B**: Changes in acetylated protein turnover correlate with fold changes in protein abundance. **C**: Heatmap showing differences in acetylation (LFQ), protein abundance (LFQ), native protein turnover (k), and acetylated protein turnover (k). Analyses are based on proteins with calculated k values. Ratios are log-scaled, with a value of one represented in white. Increased values in ethanol-fed are shown in red, and increased values in pair-fed are shown in blue. Proteins from mitochondria, cytosol, and other organelles are indicated by blue, orange, and green colors, respectively in the color bar between the heat map and dendrogram.

**Supplementary Figure S10**. Ethanol-induced changes in proteasomal protein degradation.

**A:** Western blot analysis of ubiquitinated proteins in liver samples from PF and EF mice. Bar graphs show normalized quantification relative to β-actin.

**B**: Proteasome activity was evaluated in the livers from PF and EF mice using fluorescent-tagged peptide substrates for trypsin, chymotrypsin, and caspase with and without MG132, a proteasome specific inhibitor. MG132 completely inhibited chymotrypsin-like (left) and caspase-like (right) activities without any significant change in trypsin-like (middle) activity.

**Supplementary Figure S11**. Contribution of ethanol-d6 to acetylation of histone H3 peptide KQLATKAAR. Comparison of ^2^H-labelings of native, monoacetylated, diacetylated histone H3 peptides.

**Supplementary Figure S12.** Chronic alcohol intake reprograms substrate metabolism in mouse liver.

**A**: Acyl-CoA and acyl-carnitine profiles. **B**: TCA cycle intermediates. **C**: Amino acid profiles.

**D**: Chronic alcohol consumption leads to elevated levels of 2-hydroxybutyrate, indicating reductive stress.

**References:**

(1) McCullough, R. L.; McMullen, M. R.; Sheehan, M. M.; Poulsen, K. L.; Roychowdhury, S.; Chiang, D. J.; Pritchard, M. T.; Caballeria, J.; Nagy, L. E. Complement Factor D protects mice from ethanol-induced inflammation and liver injury. *Am J Physiol Gastrointest Liver Physiol* **2018**, *315*, G66-G79.

(2) Gaucher, J.; Kinouchi, K.; Ceglia, N.; Montellier, E.; Peleg, S.; Greco, C. M.; Schmidt, A.; Forne, I.; Masri, S.; Baldi, P.; Imhof, A.; Sassone-Corsi, P. Distinct metabolic adaptation of liver circadian pathways to acute and chronic patterns of alcohol intake. *Proc Natl Acad Sci U S A* **2019**, *116*, 25250-25259.

(3) Kasumov, T.; Li, L.; Li, M.; Gulshan, K.; Kirwan, J. P.; Liu, X.; Previs, S.; Willard, B.; Smith, J. D.; McCullough, A. Ceramide as a mediator of non-alcoholic Fatty liver disease and associated atherosclerosis. *PLoS One* **2015**, *10*, e0126910.

(4) Lee, K.; Haddad, A.; Osme, A.; Kim, C.; Borzou, A.; Ilchenko, S.; Allende, D.; Dasarathy, S.; McCullough, A.; Sadygov, R. G.; Kasumov, T. Hepatic Mitochondrial Defects in a Nonalcoholic Fatty Liver Disease Mouse Model Are Associated with Increased Degradation of Oxidative Phosphorylation Subunits. *Mol Cell Proteomics* **2018**, *17*, 2371-2386.

(5) Mews, P.; Egervari, G.; Nativio, R.; Sidoli, S.; Donahue, G.; Lombroso, S. I.; Alexander, D. C.; Riesche, S. L.; Heller, E. A.; Nestler, E. J.; Garcia, B. A.; Berger, S. L. Alcohol metabolism contributes to brain histone acetylation. *Nature* **2019**, *574*, 717-721.

(6) Cui, Z.; Gilda, J. E.; Gomes, A. V. Crude and purified proteasome activity assays are affected by type of microplate. *Anal Biochem* **2014**, *446*, 44-52.

(7) Kasumov, T.; Cendrowski, A. V.; David, F.; Jobbins, K. A.; Anderson, V. E.; Brunengraber, H. Mass isotopomer study of anaplerosis from propionate in the perfused rat heart. *Arch Biochem Biophys* **2007**, *463*, 110-117.

(8) Li, L.; Willard, B.; Rachdaoui, N.; Kirwan, J. P.; Sadygov, R. G.; Stanley, W. C.; Previs, S.; McCullough, A. J.; Kasumov, T. Plasma proteome dynamics: analysis of lipoproteins and acute phase response proteins with 2H2O metabolic labeling. *Mol Cell Proteomics* **2012**, *11*, M111 014209.

(9) Bligh, E. G.; Dyer, W. J. A rapid method of total lipid extraction and purification. *Canadian journal of biochemistry and physiology* **1959**, *37*, 911-917.

(10) He, W.; Marchuk, H.; Koeberl, D.; Kasumov, T.; Chen, X.; Zhang, G. F. Fasting alleviates metabolic alterations in mice with propionyl-CoA carboxylase deficiency due to Pcca mutation. *Commun Biol* **2024**, *7*, 659.

(11) Li, Q.; Zhang, S.; Berthiaume, J. M.; Simons, B.; Zhang, G. F. Novel approach in LC-MS/MS using MRM to generate a full profile of acyl-CoAs: discovery of acyl-dephospho-CoAs. *J Lipid Res* **2014**, *55*, 592-602.

(12) Zhang, G. F.; Kombu, R. S.; Kasumov, T.; Han, Y.; Sadhukhan, S.; Zhang, J.; Sayre, L. M.; Ray, D.; Gibson, K. M.; Anderson, V. A.; Tochtrop, G. P.; Brunengraber, H. Catabolism of 4-hydroxyacids and 4-hydroxynonenal via 4-hydroxy-4-phosphoacyl-CoAs. *J Biol Chem* **2009**, *284*, 33521-33534.

(13) Shechter, D.; Dormann, H. L.; Allis, C. D.; Hake, S. B. Extraction, purification and analysis of histones. *Nat Protoc* **2007**, *2*, 1445-1457.

(14) Lin, S.; Garcia, B. A. Examining histone posttranslational modification patterns by high-resolution mass spectrometry. *Methods Enzymol* **2012**, *512*, 3-28.

(15) Evertts, A. G.; Zee, B. M.; Dimaggio, P. A.; Gonzales-Cope, M.; Coller, H. A.; Garcia, B. A. Quantitative dynamics of the link between cellular metabolism and histone acetylation. *J Biol Chem* **2013**, *288*, 12142-12151.

(16) Schilling, B.; Meyer, J. G.; Wei, L.; Ott, M.; Verdin, E. High-Resolution Mass Spectrometry to Identify and Quantify Acetylation Protein Targets. *Methods Mol Biol* **2019**, *1983*, 3-16.

(17) Aghayev, M.; Arias-Alvarado, A.; Ilchenko, S.; Lepp, J.; Scott, I.; Chen, Y. R.; Zhang, G. F.; Tsai, T. H.; Kasumov, T. A high-fat diet increases hepatic mitochondrial turnover through restricted acetylation in a NAFLD mouse model. *Am J Physiol Endocrinol Metab* **2023**, *325*, E83-E98.

(18) Sadygov, R. G.; Avva, J.; Rahman, M.; Lee, K.; Ilchenko, S.; Kasumov, T.; Borzou, A. d2ome, Software for in Vivo Protein Turnover Analysis Using Heavy Water Labeling and LC-MS, Reveals Alterations of Hepatic Proteome Dynamics in a Mouse Model of NAFLD. *J Proteome Res* **2018**, *17*, 3740-3748.

(19) Feller, C.; Forne, I.; Imhof, A.; Becker, P. B. Global and specific responses of the histone acetylome to systematic perturbation. *Mol Cell* **2015**, *57*, 559-571.

(20) Bates, D. M. a. C., J. M. . Nonlinear models. *Chapter 10 of Statistical Models in S eds J. M. Chambers and T. J. Hastie, Wadsworth & Brooks/Cole.* **1992**.

(21) Ritz, C.; Carl, J. Nonlinear Regression with R. . *Springer* **2008**.
